# Potassium Bisulfite’s Role in Developing a Robust Platform for Enantioenriched *N*‑Alkylpyridinium Salts as Piperidine Precursors

**DOI:** 10.1021/jacs.5c20464

**Published:** 2026-02-17

**Authors:** Jake D. Selingo, Jacob R. King, Barbara Pio, Andrew J. Neel, Yu-Hong Lam, Robert S. Paton, Matthew L. Maddess, Andrew McNally

**Affiliations:** † Department of Chemistry, 224023 Colorado State University, Fort Collins, Colorado 80523, United States; ‡ Department of Discovery Chemistry, 2793 Merck & Co. Inc., Rahway, New Jersey 07065, United States; § Department of Process Research and Development, 1438 Merck & Co. Inc., Boston, Massachusetts 02115, United States; ∥ Computational and Structural Chemistry, 2793 Merck & Co. Inc., Rahway, New Jersey 07065, United States; ⊥ Department of Discovery Chemistry, Merck & Co. Inc., Boston, Massachusetts 02115, United States

## Abstract

Piperidines are prominent scaffolds in medicinal chemistry. However, methods that incorporate chiral *N*-alkyl substituents on piperidine remain limited. Here, we report a platform for the synthesis of enantioenriched *N*-(α-chiral)­alkylpyridinium salts from commercially available pyridines and enantiopure primary amines; the resulting pyridinium salts serve as versatile precursors to stereoenriched *N*-(α-chiral)­alkylpiperidines via established reduction protocols. We discovered potassium metabisulfite as a reaction additive that significantly enhanced the robustness of the pyridinium formation reaction. Mechanistic and computational studies reveal that potassium metabisulfite deconjugates Zincke imines, enabling a lower-energy polar cyclization pathway to pyridinium formation compared to a pericyclic one. We performed high-throughput experimentation that demonstrated a broad scope for both coupling partners, providing a robust, general platform for generating libraries of piperidine precursors relevant to medicinal chemistry.

## Graphical abstract

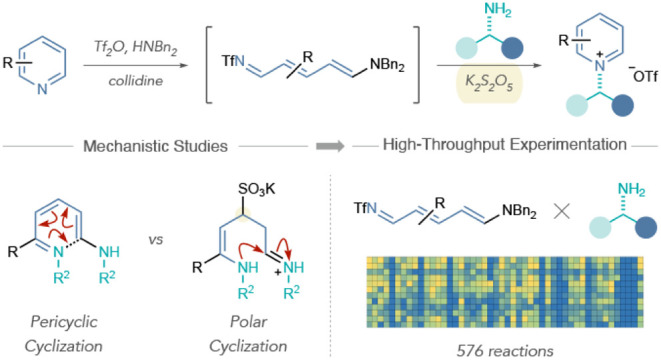

## Introduction

Piperidines are abundant components of bioactive molecules, represented in 20 different drug classes and ranking as the second most common *N*-heterocycle in FDA-approved pharmaceuticals.^1–4^ They are frequent targets of Structure Activity Relationship (SAR) studies, which can be challenging due to the lack of general methods for functionalizing their *sp* ^3^-hybridized framework and the limited commercial availability of chiral piperidine building blocks. In particular, accessing piperidines with *N*-enantioenriched carbon-bearing groups, as depicted in Figure 1A, is surprisingly challenging.^5–10^ Most modern methods for asymmetric C–N bond formation employ ketones, alkenes, or chiral organohalides and are more broadly applicable to primary amines compared to piperidines.^1,11–25^ Instead, practitioners commonly rely on more robust, unselective C–N bond-forming reactions, followed by chiral resolutions.^5–7^ Here, we report an orthogonal approach to piperidine *N*-functionalization using enantioenriched *N*-(α-chiral)­alkylpyridinium salts as key precursors. The process operates via a pyridine ring-opening, ring-closing sequence that couples abundant pyridines and enantiopure primary amines from commercial sources or pharmaceutical libraries.^26–28^ Well-established pyridinium reduction reactions or functionalization-reduction sequences can access the corresponding stereoenriched *N*-(α-chiral)­alkylpiperidines.^1,26,29–34^

**Figure 1:**
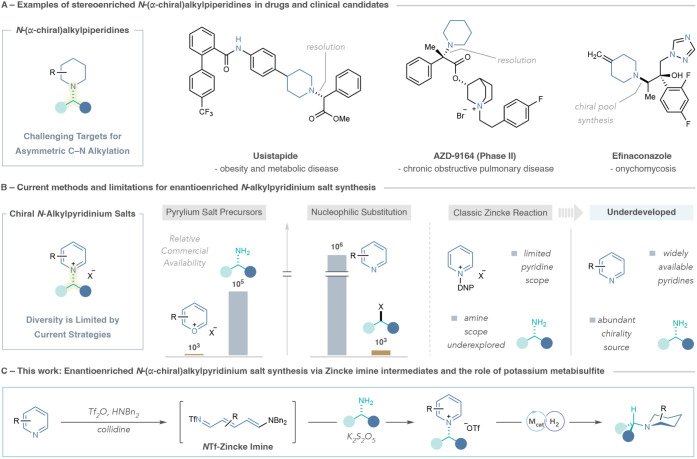
(A) Examples of pharmaceuticals and clinical candidates containing stereoenriched *N*-(α-chiral)­alkylpiperidines. (B) Common strategies for enantioenriched *N*-alkylpyridinium salt synthesis and limitations. (C) This work: enantioenriched *N*-(α-chiral)­alkylpyridinium salts synthesis via *N*Tf-Zincke imine intermediates with the discovery of potassium metabisulfite and elucidation of its mechanistic role.

The lack of a robust, general platform for synthesizing *N*-alkylpyridinium salts limits their use in synthesis,^35–41^ biology,^42–45^ materials,^46^ and as precursors to *N*-alkylpiperidines for drug discovery. Existing methods include reactions of pyrylium salts with amines and S_*N*_2 alkylations of pyridines (Figure 1B).^29,47^ The scarcity of pyrylium salt precursors and the poor reactivity of pyridines in S_*N*_2 reactions with more hindered electrophiles restrict these approaches. Alternatively, the classic Zincke reaction could be a platform that couples two abundant feedstocks, pyridines and enantiopure primary amines, and incorporates the stereochemistry of the amine into the pyridinium product.^30,48–51^ However, existing reports show that only a narrow set of pyridines function in this approach, with minimal demonstration of functional groups appended directly to the pyridine, and it does not tolerate 2-position substituents. Similarly, the scope of primary amines remains underexplored. Recently, Xiao reported a rhodium-catalyzed asymmetric transfer hydrogenation of *N*-ethylpyridinium salts with enantiopure amines that is highly efficient to access certain classes of piperidine with good levels of stereocontrol.^52^ Rather than targeting specific classes of piperidines, our goal was to develop a general method that enables broad variation of both *C*-substituents and *N*-groups.

We recently disclosed a protocol for synthesizing *N*-(hetero)­arylpyridinium salts via *N*Tf-Zincke imine intermediates, and we hypothesized that this pyridine ring-opening, ring-closing strategy could also serve as a general platform to construct enantioenriched *N*-alkylpyridinium salts using the vast collections of chiral amines (Figure 1C).^26^ Although conceptually simple, we did not achieve this goal until we discovered potassium metabisulfite as a critical additive that markedly enhances reaction generality. This report presents experimental and computational studies of metabisulfite’s effect on the reaction mechanism and its breadth, demonstrated by High-Throughput Experimentation (HTE).

## Results and Discussion

### Reaction Development

We began our study by developing a one-pot method for converting pyridines to pyridinium salts via *N*Tf-Zincke imine intermediates. Using 2-phenylpyridine, triflic anhydride, collidine, and dibenzylamine in EtOAc,^27^ we synthesized **1a** and added 1.5 equiv of amine **2a** to the same reaction vessel to study the formation of **3a** (Table 1, left). Pyridinium salt **3a** did not form at room temperature, and heating the reaction at 50 or 70 °C resulted in low yields (entries 1–3). Notably, increasing the temperature to 120 °C provided minimal yield improvement for **3a** and mainly resulted in decomposition of **1a** (entry 4). Conditions similar to our previous report, which employ AcOH in EtOAc, did not improve the yield of **3a** (entry 5).^26^ DABCO provided a significant improvement for pyridinium salt formation (entry 6). However, sulfite additives further increased the yield of **3a**, with potassium metabisulfite providing the highest yield (entries 7–9; see Supporting Information Section 2 for additional additives). Reducing the stoichiometry of metabisulfite to one equivalent did not adversely affect the reaction outcome (entry 10). We then extended our study to a small set of other amines using **2b–2d** (Table 1, right). While **2a–2c** form pyridiniums in good to excellent yields using the conditions from entry 9, we did not observe product formation with electron-deficient amine **2d**. Our previous report demonstrated MeOH can improve the reaction outcome with certain amines, so we employed it as a cosolvent for one-pot *N*-alkylpyridinium formation. While MeOH did not affect the reaction outcome with **2a** or **2b**, it improved the pyridinium salt yield with amino alcohol **2c**. Notably, including MeOH as a cosolvent enabled pyridinium formation with **2d**. These results suggest that including MeOH in the solvent mixture provides a more general set of reaction conditions.

**Table 1:**
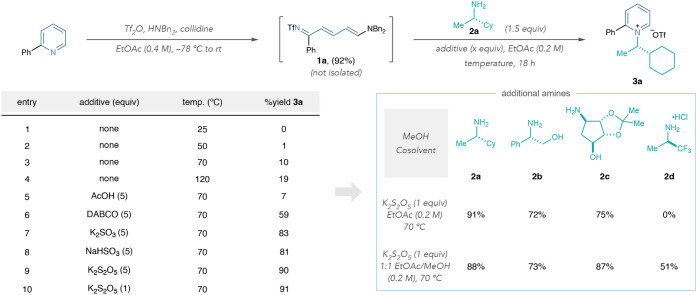
Ring-Closing Optimization Study

a Yields calculated by ^1^H NMR spectroscopy using Ph_3_CH as an internal standard.

Next, we explored the scope of the one-pot pyridinium salt formation process (Table 2). We developed Liquid–Liquid Extraction (LLE) and precipitation methods to isolate the pyridinium salts as the triflate or hexafluorophosphate salts. Salt **3b** demonstrated tolerance for enantioenriched 1-phenethylamines in pyridinium formation. The process also accommodated C2-alkyl substituted pyridines, such as **3c** derived from amino alcohol (−)-norephedrine. In general, pyridines with C2-alkyl substituents required AcOH and metabisulfite in EtOAc to form pyridinium salts in good yields (see Supporting Information Section 2). Nicotine-derived pyridinium salt **3d** formed a single diastereomer from an α-tertiary amino-ester. Salt **3e** formed in good yield but partially decomposed under the reaction conditions and the LLE stage. However, we observed a good yield of **3e** at shorter reaction times (see Supporting Information Section 6.2). Salts **3d** and **3e** were unstable under the reaction conditions with potassium metabisulfite, and excluding metabisulfite from the reaction improved the yields for both salts. Pyridinium salts derived from 2,3- and 3,4-disubstituted pyridines containing esters and halides were also accessible and efficiently coupled with fluorinated aminopiperidines and aminocyclopropanes in good yields, respectively (**3f** and **3g**).

**Table 2:**
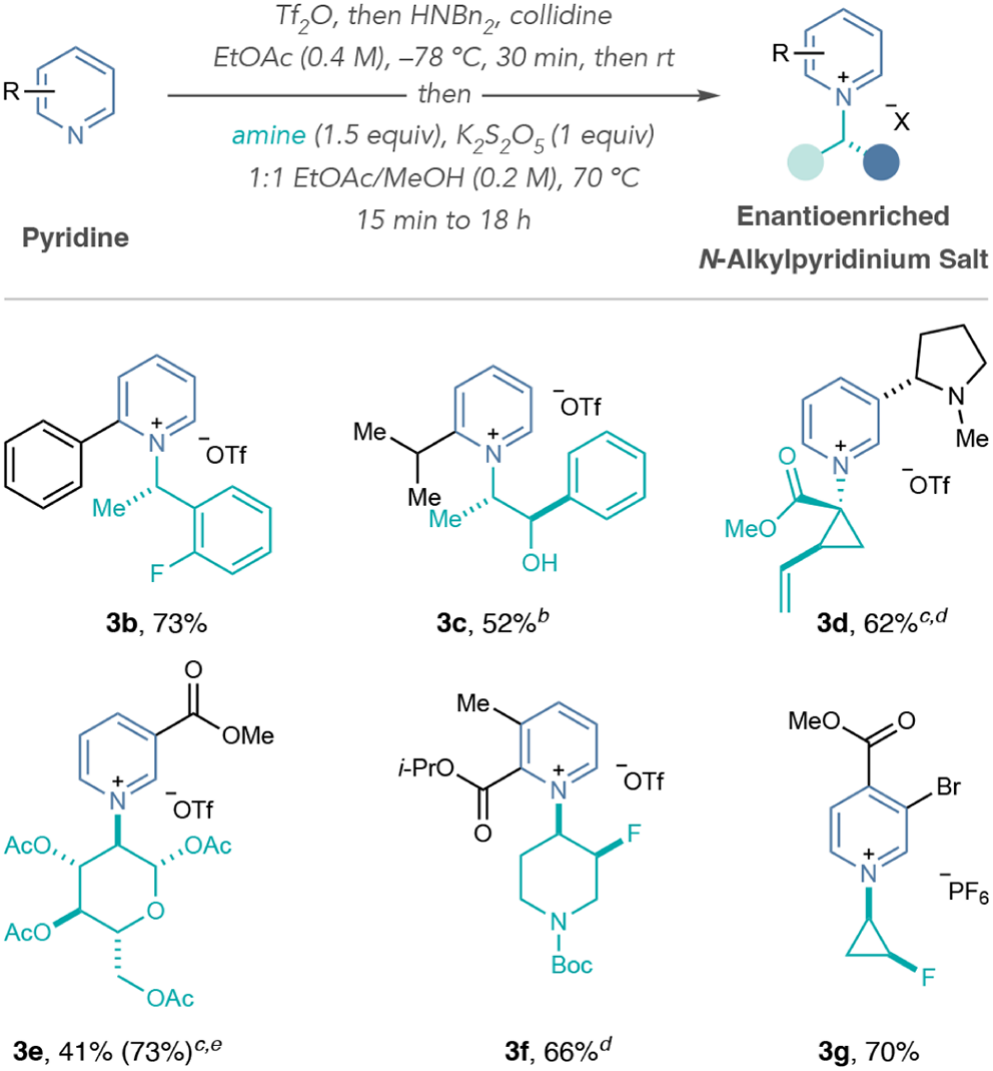
One-Pot Pyridinium Salt Formation Scope^a^ ^b^ ^c^ ^d^ ^e^

a Isolated yields are shown.

b Reaction used 10 equiv of AcOH, 1 equiv of K_2_S_2_O_5_ in EtOAc (0.2 M).

c K_2_S_2_O_5_ not used in reaction.

d Reaction ran at 50 °C.

e Yields calculated by ^1^H NMR spectroscopy using 1,3,5-trimethoxybenzene as an internal standard.

### Nucleophilic Additive Effects in Pyridinium Formation

The next phase of our study focused on the role of potassium metabisulfite as a reaction additive. We investigated the cyclization of isolated Zincke imine **1a** with amine **2a** independent from the ring-opening byproducts generated in the one-pot pyridinium formation. While EtOAc is necessary for generality in the ring-opening step, we found that using MeOH as the sole reaction solvent with isolated Zincke imines reproduced the yields obtained with potassium metabisulfite in Table 1 (see Supporting Information Section 7.1). In Scheme 1, we surveyed the reactivity of several sulfite-based reagents and compared them to common acids and bases. The data indicate that potassium metabisulfite, sodium bisulfite, and potassium sulfite all significantly improve the yields of pyridinium salt **3a–NHTf**, whereas acid and base additives did not. We postulate that the similar efficiency of these additives implies a common active species, and previous precedent describes that metabisulfite will form an equilibrium with bisulfite anions in the presence of nucleophiles (eq 1).^53,54^ 
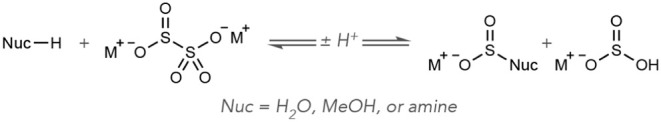


**Scheme 1:**
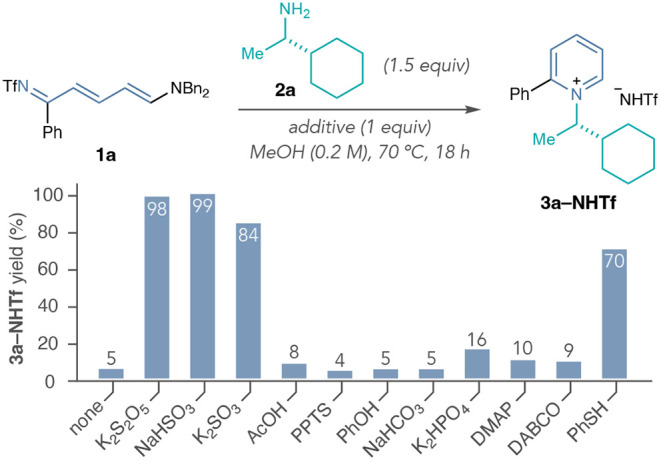
Comparison of Nucleophilic and Non-Nucleophilic Reaction Additives for the Recyclization of Isolated 1a^a^

a Yields calculated by ^1^H NMR spectroscopy using 1,3,5-trimethoxybenzene as an internal standard.

There is well-established precedent for bisulfite deconjugating polarized contiguous π-bonds in organic dyes and aromatic rings.^55–60^ We hypothesized that similar processes were operating in the cyclization of Zincke imines to pyridinium salts. However, other nucleophilic additives, such as DABCO, did not increase the yield of **3a–NHTf**, unlike the result reported in entry 5 of Table 1. This result emphasized the effect of the ring-opening byproducts on the downstream pyridinium formation steps (see Supporting Information Section 7.1). Interestingly, thiophenol did result in a substantial increase in the yield of **3a–NHTf** compared to the other acids, bases, and nucleophiles tested. We examined other electronically distinct thiophenols and observed that the nucleophilicity of the sulfur atom (Mayr’s nucleophilicity parameter, *N*) and the p*K*_a_ of the S–H bond had positive correlations to the yield of **3a–NHTf** (see Supporting Information Section 7.1).^61,62^ These trends for thiophenol additives and the analogous reactivity to bisulfite additives in pyridinium formation suggested that both the acidity and nucleophilicity of bisulfite are central to its reactivity.

### Proposed Mechanisms for Pyridinium Formation and the Role of Bisulfite

To determine bisulfite’s role in the mechanism, we first considered the key steps of pyridinium formation without exogenous additives (Scheme 2A). We propose that the process begins with a transamination step that incorporates the amine nucleophile into Zincke imine **1** to form **1–(NHR** ^**2**^)_**2**_. This process is observable by ^1^H NMR and low-resolution mass spectrometry (LRMS) at early time points of the reaction (vide infra). Next, *E/Z*-isomerization of the all-*trans*-configured **1–(NHR** ^**2**^)_**2**_ generates the requisite *cis*-isomer **Int-I**, which undergoes a disrotatory 6π-electrocyclization to yield the cyclized **Int-II**. Elimination of the exocyclic amine produces the product **3**.

**Scheme 2:**
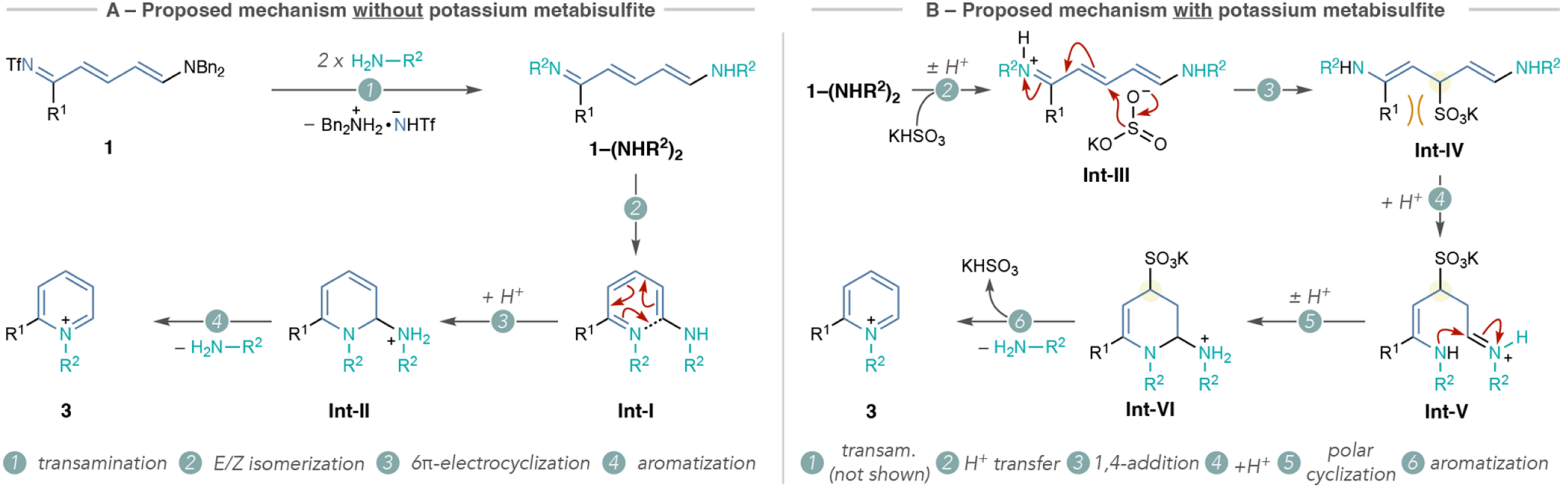
Proposed Mechanisms for Pyridinium Formation without Additives and with Potassium Metabisulfite[Fn sch2-fn2]

a Counterions are omitted for clarity.

We then considered potassium bisulfite’s role in promoting pyridinium formation and propose an alternative mechanism in Scheme 2B. We reasoned that bisulfite protonates **1–(NHR** ^**2**^)_**2**_ to facilitate 1,4-addition of sulfite to the protonated Zincke imine **Int-III** to generate **Int-IV**. This deconjugation process with bisulfite would obviate the *E/Z*-isomerization and 6π-electrocyclization steps outlined in Scheme 2A, providing an alternative, polar pathway to pyridinium formation. Protonation of the enamine followed by a 6-*exo*-trig cyclization through **Int-V** generates **Int-VI**. Elimination of the amine and subsequent E1cB elimination of bisulfite produces pyridinium **3**. Notably, the aromatization process regenerates potassium bisulfite. Using Zincke imine **1a** and amine **2a** with 20 mol % potassium metabisulfite produced a comparable yield of **3a–NHTf** to that obtained with a full equivalent. Other amines, such as **2d**, required a full equivalent of potassium metabisulfite to produce high yields of the corresponding salt. Therefore, we continued to use it as a stoichiometric additive for reaction generality (see Supporting Information Section 7.2).

### Investigation into Zincke Imine Deconjugation

Next, we investigated the deconjugation of Zincke imines with bisulfite additives (Scheme 3, top). Despite the arguments presented thus far, we did not observe **1a–SO**_**3**_ **K** when we treated **1a** with potassium metabisulfite in MeOH at 70 °C (see Supporting Information Section 7.3). Nevertheless, we assumed these species may transiently form in low concentrations and examined the deuteration of **1a** in CD_3_OD at 70 °C to study the deconjugation phenomenon independent of pyridinium formation (Scheme 3, bottom).^27,63^ Importantly, isotope incorporation did not occur without additives after 2 h. Brønsted acids, such as AcOH and pyridinium *p*-toluenesulfonate (PPTS), afforded varying degrees of deterium incorporation, but both reactions were indiscriminate between the C3- and C5-positions of ***d*** **-1a**. However, potassium metabisulfite, sodium bisulfite, and thiophenol all furnished high isotope incorporation of ***d-*** **1a** with considerable selectivity for the C5-position (see Supporting Information Section 7.4 for additional additives). We postulate that this outcome may arise from intermediate **1a–SO**_**3**_ **K**, as the more reactive *N*-dialkyl enamine would deuterate the C5-carbon preferentially over the C3-position within the *N*-triflyl enamine.

**Scheme 3:**
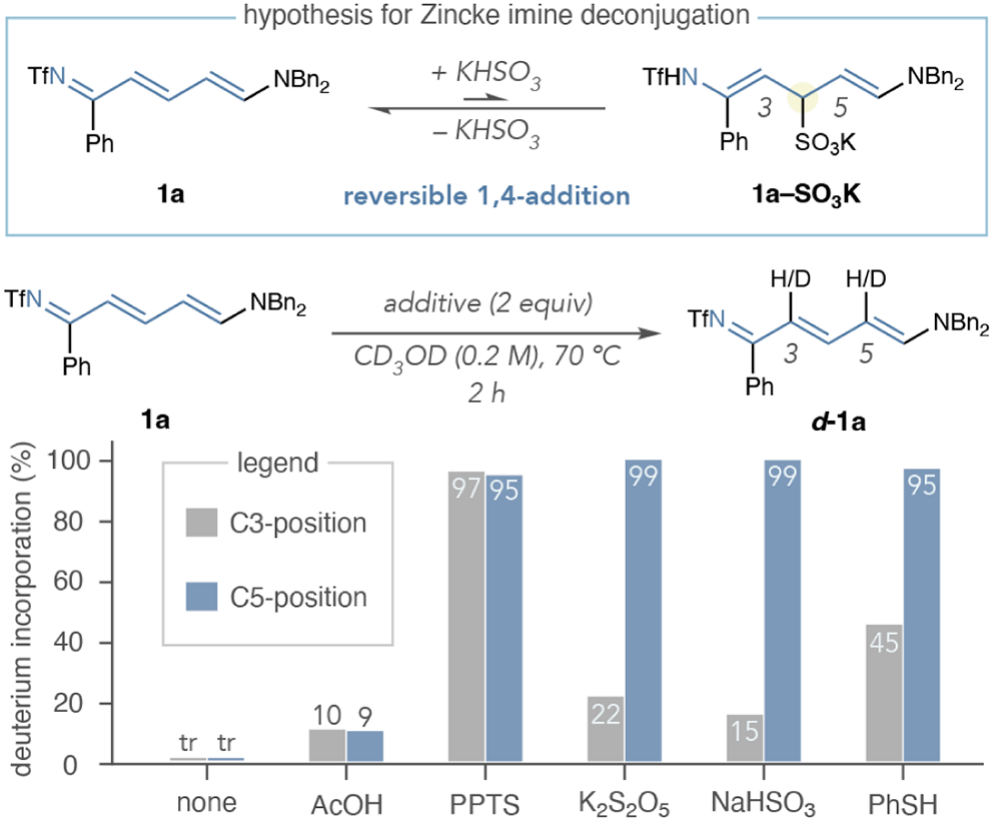
Hypothesis for Zincke Imine Deconjugation and Comparison of Additives in the Deuteration of 1a[Fn sch3-fn3] [Fn sch3-fn4]

a Deuterium incorporation calculated by ^1^H NMR spectroscopy using 1,3,5-trimethoxybenzene as an internal standard.
b tr = trace.

### Experimental Investigation of Mechanistic Steps with Potassium Metabisulfite

We then studied how bisulfite additives affect the mechanism of pyridinium salt formation using the reaction of Zincke imine **1a** and isopropylamine as a model system (Scheme 4). At room temperature, we observed an initial transamination event that formed a collection of new Zincke imines (***transaminated*** **–1a**) that are structurally similar by ^1^H NMR spectroscopy but distinguishable by LCMS analysis (Scheme 4A). It is conceivable that potassium metabisulfite accelerates this transamination step by deconjugating Zincke imine **1a**. Yet, at a 2-h time point, we saw minimal difference compared to a control reaction without the additive. We did, however, observe minor amounts of pyridinium salt **3h** when bisulfite was present.

**Scheme 4:**
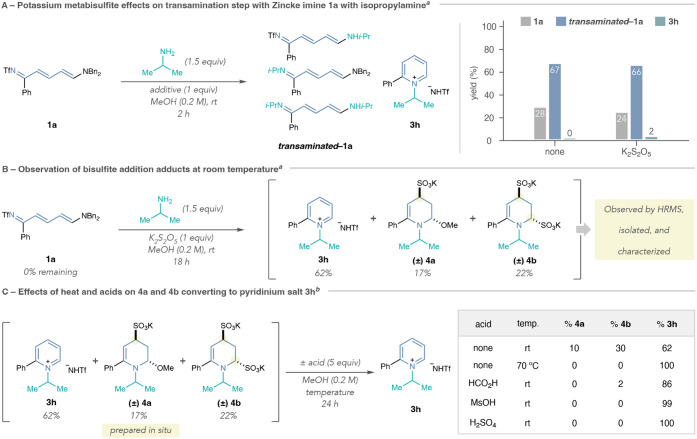
Investigation of Potassium Metabisulfite Effects on the Mechanism of Pyridinium Salt Formation[Fn sch4-fn5] [Fn sch4-fn6]

a Yields calculated using ^1^H NMR spectroscopy using 1,3,5-trimethoxybenzene as an internal standard.
b Yields calculated using ^1^H NMR spectroscopy using 1,3,5-trimethylbenzene as an internal standard.

In Scheme 4B, we prolonged the previous reaction with potassium metabisulfite at room temperature for 18 h and observed two distinct intermediates as well as appreciable amounts of pyridinium product **3h**. We separated the two new polar products from **3h** and isopropylamine, and structure elucidation using NMR spectroscopy and High-Resolution Mass Spectrometry (HRMS) supported the structures for monobisulfite adduct **4a** and bis-bisulfite adduct **4b**. Importantly, resubjecting **3h** to the reaction conditions does not decompose the salt nor form **4a** or **4b**, suggesting that **3h** forms irreversibly, and it is not the origin of **4a** or **4b** (see Supporting Information Section 7.6).

We hypothesized that **4a** and **4b** may form through the mechanism proposed in Scheme 2B via exchange of the exocyclic amine in **Int-VI** with methanol or bisulfite, respectively. Therefore, we examined their reactivity for pyridinium formation by subjecting the crude reaction mixture containing **3h**, **4a**, and **4b** to various conditions (Scheme 4C). Stirring the reaction mixture for an additional 24 h equilibrates **4a** to **4b**, but neither of the intermediates convert to **3h**. However, heating the reaction to 70 °C for 24 h forms **3h** exclusively, explaining why we do not observe either of these intermediates under the optimized reaction conditions. Acids tested in Scheme 4C also convert **4a** and **4b** to **3h** at room temperature. We suspect that acids promote the elimination of methanol and bisulfite from **4a** and **4b**, thereby facilitating the final aromatization step of the mechanism. The results of this study suggest that bisulfite may alter the reaction pathway for pyridinium formation via deconjugated Zincke imine and pyridinium intermediates that enable a distinct cyclization mechanism, as evidenced by **4a** and **4b**.

### Computational Investigation

We next employed density functional theory (DFT) to investigate the reaction mechanism with and without bisulfite additives at the ωB97M-V/def2-TZVPP//M06-2X­(D3)/6-31+G­(d,p) level of theory in methanol (Figure 2).^64^ Quantum chemical calculations were performed with Gaussian 16 revision C.01 and Orca 6.0.0.^65,66^ For full details of computations and references see Supporting Information. Importantly, in the proposed mechanism without bisulfite, the rate-limiting step is the disrotatory 6π-electrocyclization of **Int-VIII** through **TS5** (ΔG^‡^ = 23.9 kcal/mol; see Supporting Information Section 7.7.2). Based on the initial configuration of **Int-VIII**, the disrotatory motion generates the *syn* conformation in **TS5** and results in steric strain between the *N*-isopropyl and exocyclic amine substitutents. Therefore, we investigated our hypothesis regarding bisulfite’s role in the cyclization, with our computed energy surface starting from transaminated-Zincke imine **Int-VII**, since this step occurs independently of bisulfite (vide supra). After protonation of **Int-VII** (ΔG = −6.4 kcal/mol), potassium sulfite undergoes facile 1,4-addition to **Int-VIII** via **TS1,** generating the addition intermediate **Int-IX** (ΔG^‡^ = 12.7 kcal/mol, ΔG = −10.9 kcal/mol). **Int-IX** is protonated to generate **Int-X** (ΔG = −17.5 kcal/mol), which undergoes rapid 6-*exo*-trig cyclization via **TS2** to produce cyclic **Int-XI** (ΔG^‡^ = 5.8 kcal/mol, ΔG = 2.3 kcal/mol). Notably, the steric strain present in **TS5** is absent in **TS2**, as the *N*-isopropyl substituent is preferentially *anti* to the exocyclic amine, providing a facile polar cyclization. Following proton transfer, **Int-XI** converts to the more thermodynamically favored **Int-XII** (ΔG = −12.3 kcal/mol), which eliminates isopropylamine through **TS3** to form iminium **Int-XIII** as the rate-determining step (ΔG^‡^ = 19.9 kcal/mol, ΔG = 10.1 kcal/mol). Interestingly, we found **Int-XIII** to be an intermediary species in the formation of **3h**, as well as the off-pathway intermediates **4a** and **4b** (see Supporting Information Section 7.7.5). The remaining E1cB sequence from **Int-XIII** generates **3h**; this sequence commences with deprotonation of **Int-XIII** to produce **Int-XIV** (ΔG = −12.8 kcal/mol). Then, **Int-XIV** eliminates KSO_3_^–^ through **TS4** to produce **3h** (ΔG^‡^ = 17.7 kcal/mol, ΔG = −4.6 kcal/mol). The low-energy barrier for cyclization via **TS2** supports our hypothesis that bisulfite enables a facile polar cyclization process, and the rate-determining amine elimination via **TS3** demonstrates its role in reducing the overall kinetic barrier for pyridinium formation through a distinct mechanism.

**Figure 2:**
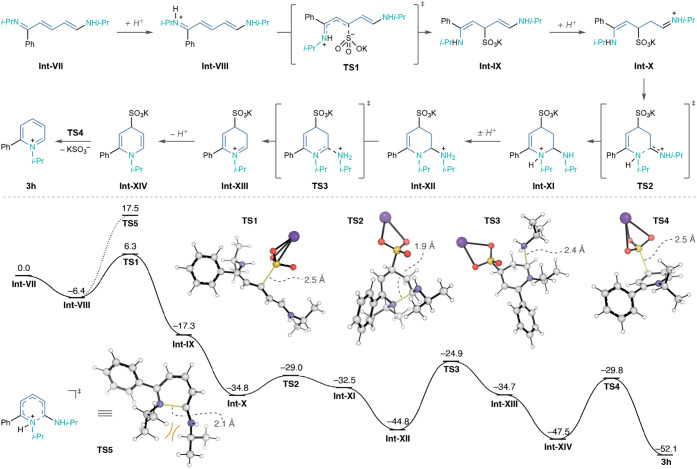
Proposed mechanism for the reaction pathway with potassium bisulfite (bold) and without (dashed line). Gibbs energy surface computed at the ωB97M-V/def2-TZVPP,CPCM­(methanol)//M06-2X­(D3)/6-31+G­(d,p), PCM­(methanol) level of theory. Counterions are omitted for clarity.

### Influence of Zincke Imine Substitution Patterns on Pyridinium Formation

Next, we systematically investigated the effects of potassium metabisulfite with other Zincke imine substitution patterns using amine **2a** as a representative nucleophile (Scheme 5). There was a significant impact on **3a** formation with metabisulfite, yet **3i** and **3j** formed in near quantitative yields without it. Pyridiniums with methyl groups at the C3-position were higher yielding with potassium metabisulfite in the reaction, and a larger effect was observed with an additional C2-phenyl group (**3k and 3l**). Potassium metabisulfite did not impact the yield of 2,4-disubstituted **3m**; however, it did significantly improve the formation of 2,5-disubstituted **3n**. These results suggest that the Zincke imine substitution pattern has a significant impact on pyridinium formation, showing that metabisulfite is not always necessary or effective in improving the reaction outcome. We suspect that C2-substituents on the Zincke imine can impede pyridinium formation; however, large substituents and/or substitution patterns that generate steric repulsion with coplanar substituents in the all *trans*-Zincke imine configuration, such as a C2-phenyl group interacting with a C4-methyl group in salt **3m**, promote pyridinium formation without involving metabisulfite (Figure 3).

**Scheme 5:**
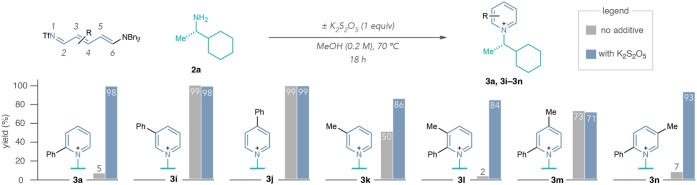
Potassium Metabisulfite Effects with Different Zincke Imine Substitution Patterns^a^ ^b^

a Yields calculated by ^1^H NMR spectroscopy using 1,3,5-trimethoxybenzene as an internal standard.
b Counterions are omitted for clarity.

**Figure 3:**
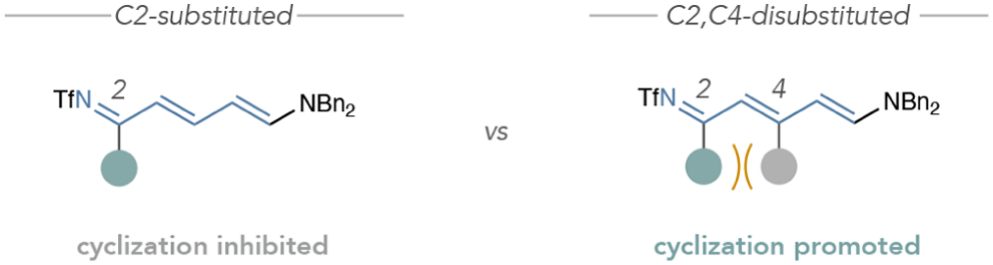
Hypothesis for steric interactions that promote pyridinium formation.

### HTE Screening

We then used HTE to assess the compatibility of a set of Zincke imines with an extensive range of chiral amines. We selected 48 enantioenriched (α-chiral)­amines **2a–2av** from the Merck & Co., Inc., Rahway, NJ, USA compound library, shown in Figure 4, and synthesized 12 substituted Zincke imines (**1a–1l**).^26,67,68^ First, we performed a control screen using Zincke imine **1a** and amines **2a–2av** without potassium metabisulfite (Table 3; see Supporting Information 8.2 for further details). We observed that most products did not form without the additive, and none formed in a yield of ≥20%. However, Zincke imines **1a–1l** were all successful in forming pyridinium salts under the reaction conditions with potassium metabisulfite, underscoring the importance of the additive for reaction robustness. Additionally, 37 out of the 48 amines were successful in forming ≥20% pyridinium product with at least three Zincke imines. These examples demonstrate that the reaction tolerates various amine classes, spanning from simple aliphatic amines to more complex (hetero)­benzylic amines, β-aryl amines, amino alcohols, amino heterocycles, protected diamines, and amino esters. Notably, most of the salts generated in this study retain the stereochemistry of the chiral amine. Pyridinium salts derived from heterobenzylic amines and ∝-amino esters epimerize under the reaction conditions to different degrees (see Supporting Information Section 8.4). Although the bulk of the amines were successful in pyridinium formation, **2e**, **2j**, **2o**, **2v**, **2ae**, **2ag**, **2ah**, **2am**, **2as**, **2at**, and **2au** formed <20% product with most of the Zincke imines tested. Our analysis of these reaction wells indicated this was often due to product decomposition under the reaction conditions or a poor transamination process, as evidenced by UPLC-MS detection of the parent pyridine and corresponding elimination byproducts, or the unreacted Zincke imine, respectively (see Supporting Information 8.5). The bottom of Table 3 presents examples of pyridiniums formed in the HTE screen. These representative examples demonstrate the method’s robustness across substantial variations in both Zincke imine and amine coupling partners, enabling access to libraries of diverse piperidine precursors.

**Figure 4:**
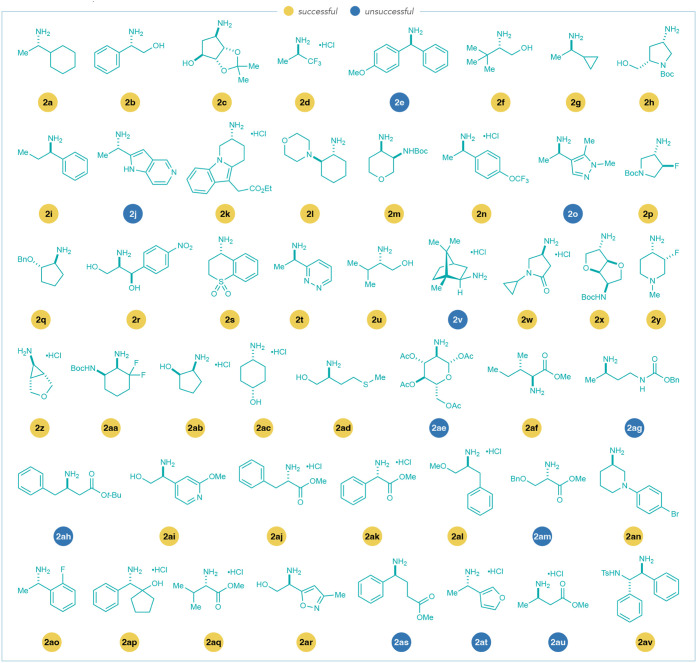
Scope enantioenriched (α-chiral)­amines (**2a–2av**) explored in high-throughput experimentation. Successful amines formed ≥ 20% product with at least 3 Zincke imines.^67^ Yields calculated by UPLC-MS-CAD analysis with noscapine as an external calibrant.

**Table 3:**
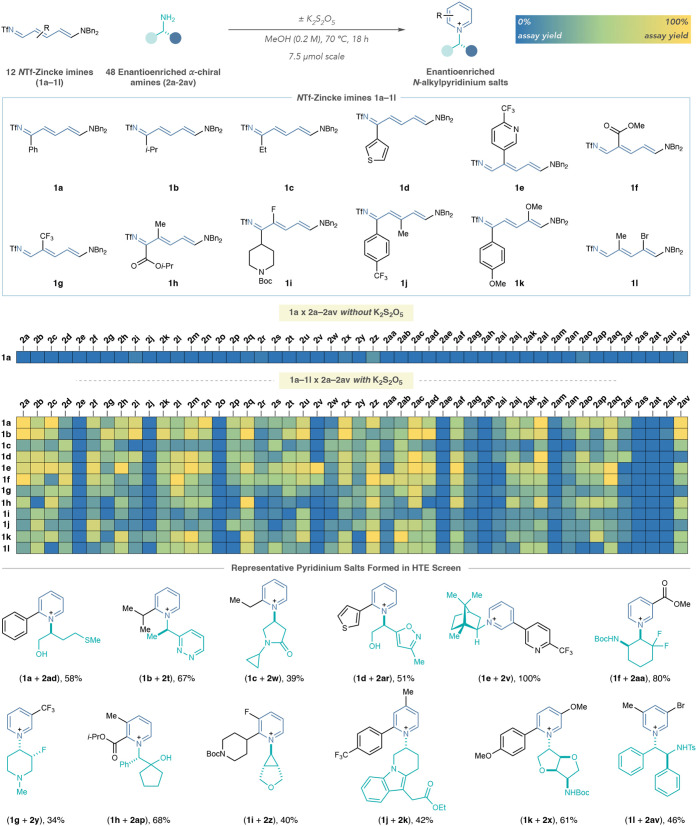
High-Throughput Experimentation for Pyridinium Salt Formation^a^ ^b^ ^c^

a 12 × 48 screen: 7.5 μmol **1a–1l**, 11.25 μmol **2a–2av**, 15 μmol K_2_S_2_O_5_, 0.2 M in MeOH.

b Yields calculated by UPLC-MS-CAD analysis with noscapine as an external calibrant.

c Counterions are omitted for clarity.

### HTE Validations and Reaction Improvements

Next, we validated the UPLC-CAD yields using quantitative ^1^H NMR analysis and isolated the products on preparative scale (Table 4). Although pyridiniums generally formed in higher yields on a larger reaction scale, the UPLC-CAD analysis provided reasonable estimates of reaction outcomes (see Supporting Information Section 8.3 for additional validations). Isolation via LLE and precipitation readily accessed the pyridinium salt products formed in the HTE screen on preparative scale (**3o–3t**).

**Table 4:**
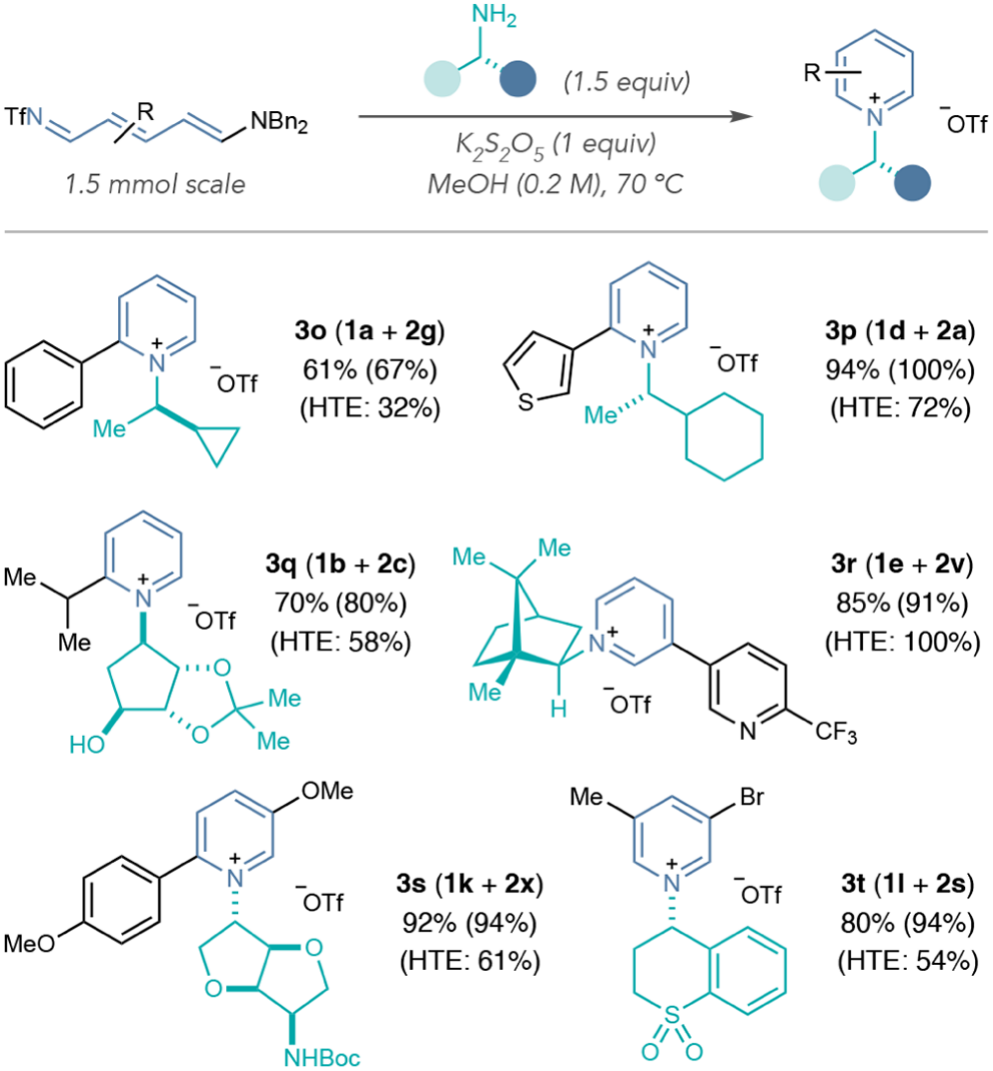
Preparative Scale ^1^H NMR Validations of Pyridinium Salts Formed in HTE Screening^a^ ^b^ ^c^

a Isolated yields on 1.5 mmol scale are shown.

b Yields in parentheses calculated by ^1^H NMR spectroscopy using 1,3,5-trimethoxybenzene as an internal standard.

c HTE yields calculated by UPLC-MS-CAD with noscapine as an external calibrant.

We then addressed some of the limitations observed in the HTE with adjustments to the reaction conditions. First, running the reaction at 50 °C instead of 70 °C improves the yields of pyridinium salts sensitive to elimination, such as **3o–NHTf** (Scheme 6A). Additional studies in Supporting Information Section 8.5 show general improvement for other pyridinium salts prone to elimination, including salts derived from β-amino esters. However, we still did not see pyridinium salt formation using amines **2e** and **2o**, even at room temperature. Second, Scheme 6B demonstrates that amino sugar **2ae** will form pyridinium salts without potassium metabisulfite at a lower temperature and shorter time (**3e–NHTf**). Removing this additive is therefore an option when using sensitive amines and Zincke imines without C2-substituents (vide supra). Third, adding acid and heating the reaction after the recyclization step can improve pyridinium salt formation with electron-deficient amines, such as **3u** in Scheme 6C, as well as amino alcohols and diamines, potentially by promoting the aromatization step of the reaction mechanism (see Supporting Information Section 8.5 for additional examples). Fourth, employing the amine as the limiting reagent can improve yields for pyridiniums with C2-alkyl substituents, such as **3v**, and provides practitioners with an alternate protocol when using valuable amines (Scheme 6D).

**Scheme 6:**
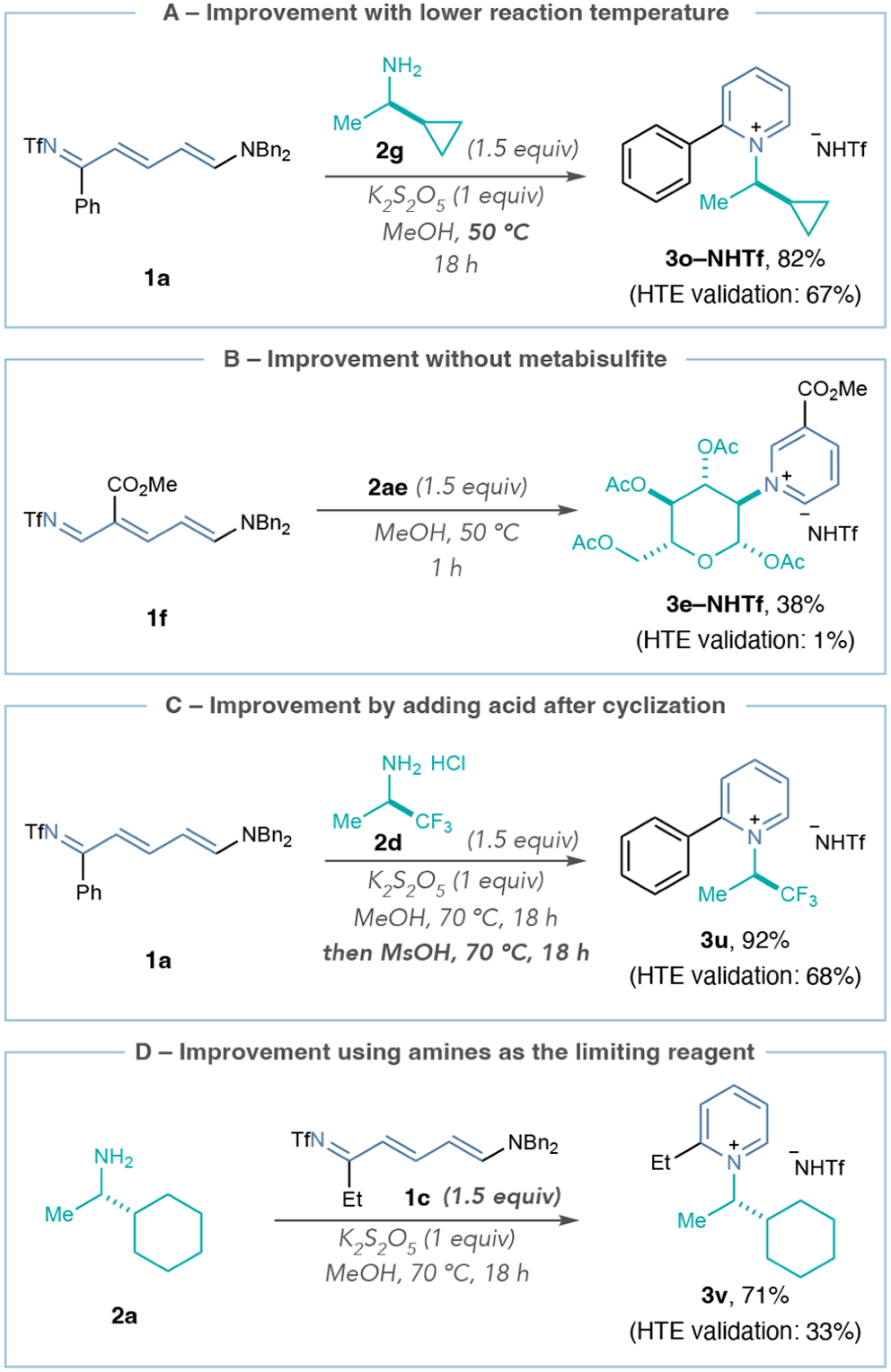
Reaction Improvements for Forming Pyridinium Salts^a^

a Yields calculated by ^1^H NMR spectroscopy using 1,3,5-trimethoxybenzene as an internal standard.

### Synthetic Applications

We next examined the robustness of one-pot pyridinium formation using 2-phenylpyridine and amine **2a** (Scheme 7A). Without needing to modify the reaction or isolation protocol, we obtained salt **3a** in high yield and 99% purity (see Supporting Information Section 9.1.). Furthermore, we demonstrated the synthetic utility for late-stage convergent coupling of complex pyridine and amine fragments (Scheme 7B). To this end, we performed the selective ring-opening of etoricoxib to couple the resulting Zincke imine with linagliptin under the one-pot conditions developed for C2-alkyl pyridines. Notably, LLE and precipitation provided salt **3af** in modest yield, demonstrating the breadth of this strategy in various synthetic stages for pharmaceutical development. Lastly, we demonstrated proof-of-concept for stereoenriched *N*-alkylpiperidine formation by subjecting three of the pyridinium salts generated in this study to platinum dioxide-catalyzed reduction (Scheme 7C).^29^ We observed that the pyridinium *N*-substituent can modestly influence the diastereoselectivity when C2- groups are present (**5a** and **5b**). Alternatively, C3-substituted pyridinium salts do not exhibit the same stereocontrol by the *N*-substituent, such as **5c**, which forms in good yield as a nearly 1:1 mixture of diastereomers. There are several other approaches to improving diastereocontrol during pyridinium salt reductions, including exploiting ligand effects in metal-catalyzed reductions, regent-based additions, and the use of designed *N*-substituents.^30,52,69,70^

**Scheme 7:**
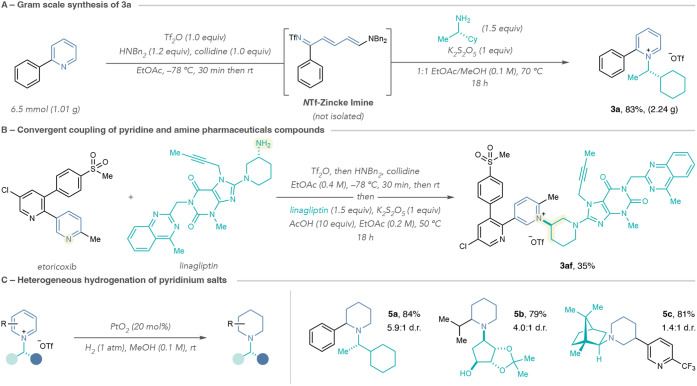
Synthetic Applications for Enantioenriched *N*-Alkylpyridinium Salt Formation^a^

a Isolated yields are shown.

## Conclusions

In summary, we have developed a robust platform for synthesizing enantioenriched *N*-alkylpyridinium salts as *N*-(α-chiral)­alkylpiperidine precursors. Mechanistic investigations revealed that potassium metabisulfite enhances the scope and yield of pyridinium salt formation through a distinct cyclization mechanism. This understanding led to additional improvements in reaction yields for select amines and could have implications in other classes of heterocycle-forming reactions. We employed HTE to assess diverse Zincke imine and enantioenriched amine collections in pyridinium formation and demonstrated their use as piperidine precursors. This strategy can generate diverse and complex libraries for SAR studies and will be directly applicable to medicinal chemists for accessing new piperidine chemical space.

## Supplementary Material



## Notes

Experimental procedures and characterization data for all reported compounds, protocols for high-throughput experimentation and details of the analysis methods, and descriptions of computational methods (PDF)

## Abbreviations

SAR: structure–activity relationship
THE: high-throughput experimentation
DNP: 2,4-dinitrophenyl
LLE: liquid–liquid extraction
PPTS: pyridinium *p*-toluenesulfonate
PhOH: phenol
PhSH: thiophenol
HRMS: high-resolution mass spectrometry
NMR: nuclear magnetic resonance
UPLC-MS-CAD: ultraperformance liquid chromatography–mass spectrometry-charged aerosol detection
DFT: density-functional theory
PES: potential energy surface
PCM: polarizable continuum model.
