# Supplementary material for: Potassium Bisulfite’s Role in Developing a Robust Platform for Enantioenriched N‑Alkylpyridinium Salts as Piperidine Precursors
Source: J Am Chem Soc. 2026 Feb 17;148(8):8621–33. doi: 10.1021/jacs.5c20464 (PMC12964407; doi:10.1021/jacs.5c20464)
Supplement: Supplementary file 1 [file ja5c20464_si_001.pdf]

Supporting Online Material for

Potassium Bisulfite's Role in Developing a Robust Platform for Enantioenriched *N*-Alkylpyridinium Salts as Piperidine Precursors

Jake D. Selingo[a], Jacob R. King[a], Barbara Pio[b], Andrew J. Neel[c], Yu-hong Lam[d], Robert S. Paton[a], Matthew L. Maddess[e]* and Andrew McNally[a]*

[a]Department of Chemistry, Colorado State University, Fort Collins, Colorado 80523, United States. [b]Department of Discovery Chemistry, Merck & Co., Inc., Rahway, New Jersey 07065, United States. [c]Department of Process Research and Development, Merck & Co., Inc., Boston, Massachusetts 02115, United States. [d]Computational and Structural Chemistry, Merck & Co., Inc., Rahway, New Jersey 07065, United States. [e]Department of Discovery Chemistry, Merck & Co., Inc., Boston, Massachusetts 02115, United States. *Corresponding authors. Emails: matthew_maddess@merck.com; andy.mcnally@colostate.edu

Table of Contents

1. General Information *S3*

2. One-Pot Optimization Studies and Limitations *S4*

3. Preparation of Starting Materials *S8*

4. Preparation of Zincke Imines *S9*

5. General Procedures for Preparation of Enantioenriched *N*-Alkylpyridinium Salts *S18*

6. Characterization Data for Enantioenriched *N*-Alkylpyridinium Salts *S22*

6.1. Enantioenriched *N*-Alkylpyridinium Salts from Optimization Studies *S22*

6.2. Enantioenriched *N*-Alkylpyridinium Salts Prepared in One Pot from Pyridines *S24*

6.3. Enantioenriched *N*-Alkylpyridinium Salts from Mechanism Studies *S28*

6.4. Enantioenriched *N*-Alkylpyridinium Salts Prepared from Zincke Imines (HTE Validation) *S31*

7. Mechanism Studies *S39*

7.1. Examination of Reaction Additive Effects in Pyridinium Formation *S39*

7.2. Sub-stoichiometric Metabisulfite Loading *S43*

7.3. Hypotheses and Control Experiments *S44*

7.4. Deuterium Labeling Experiments *S47*

7.5. Transamination Time Study with Potassium Metabisulfite *S56*

7.6. Reaction Intermediate Characterizations and Control Experiments *S57*

7.7. Computation Details *S76*

7.7.1. General Information *S76*

7.7.2. Evaluation of Methods *S77*

7.7.3. Proposed Mechanism without Bisulfite *S77*

7.7.4. Protonation Equlibria *S78*

7.7.5. Formation of Bisulfite Adducts *S79*

7.7.6. Thermochemical Data *S81*

7.7.7. Structural Data *S87*

7.8. Effects of Potassium Metabisulfite with Zincke Imine Substitution Patterns *S162*

7.9. *N*-(Heteroaryl)pyridinium Salt Yield Improvements with Acid *S163*

8. HTE for Enantioenriched *N*-Alkylpyridinium Salt Formation *S164*

8.1. Procedure and Assay for HTE Screens *S164*

8.2. Results and Validations for HTE of Enantioenriched *N*-Alkylpyridinium Salt Formation Without Metabisulfite *S165*

8.3. Results and Validations for 12 Zincke imines x 48 Enantioenriched (α-Chiral)amines Screen *S167*

8.4. Validation of Amine Stereoretention *S171*

8.5. Limitations and Yield Improvements for Enantioenriched Pyridinium Salt Formation *S172*

9. Synthetic Applications *S168*

9.1. Gram-Scale Enantioenriched *N*-Alkylpyridinium Salt Synthesis *S177*

9.2. Convergent Coupling of Etoricoxib and Linagliptin *S178*

9.3. Heterogeneous Hydrogenation of Enantioenriched *N*-Alkylpyridinium Salts *S178*

10. References *S180*

11. NMR Data (^{1}H, ^{13}C, ^{19}F spectra) *S183*

1. General Information

Proton nuclear magnetic resonance (^{1}H NMR) spectra were recorded at ambient temperature on a Varian 400 MR spectrometer (400 MHz), an Agilent Inova 400 (400 MHz) spectrometer, or a Bruker AV-111 400 (400 MHz) spectrometer. Chemical shifts (δ:) are reported in ppm and quoted to the nearest 0.1 ppm relative to the residual protons in $CDCl_3$ (7.26 ppm), CD_3OD (3.31 ppm), $(CD_3)_2CO$ (2.05 ppm), CD_3CN (1.94 ppm), D_2O (4.79 ppm), or $(CD_3)_2SO$ (2.50 ppm) and coupling constants (J) are quoted in Hertz (Hz). Data are reported as follows: Chemical shift (multiplicity, coupling constants, number of protons). Coupling constants were quoted to the nearest 0.1 Hz and multiplicity reported according to the following convention: s = singlet, d = doublet, t = triplet, q = quartet, qn = quintet, sext = sextet, sp = septet, m = multiplet, br = broad. Where coincident coupling constants have been observed, the apparent (app) multiplicity of the proton resonance has been reported. Carbon nuclear magnetic resonance (^{13}C NMR) spectra were recorded at ambient temperature on an Agilent Inova 400 (100 MHz) spectrometer or a Bruker AV-111 400 (100 MHz) spectrometer. Chemical shift (δ:) was measured in ppm and quoted to the nearest 0.01 ppm relative to the residual solvent peaks in $CDCl_3$ (77.16 ppm), CD_3OD (49.00 ppm), $(CD_3)_2CO$ (29.84 ppm), CD_3CN (1.32 ppm), or $(CD_3)_2SO$ (39.52 ppm). Two-dimensional experiments (COSY, NOESY, HSQC, HMBC) were used to support assignments where appropriate.

Hexane, diethyl ether (Et_2O), and dichloromethane (CH_2Cl_2) were dried and distilled using standard methods. 1,2-dichloroethane (DCE), chloroform ($CHCl_3$), ethanol (EtOH), ethyl acetate (EtOAc), *iso*-propyl acetate (IPAc), methanol (MeOH), isopropyl alcohol (IPA), and acetone were purchased anhydrous from Thermo Fisher Scientific and used as received. All reagents were purchased at the highest commercial quality and used without further purification. Reactions were carried out under an atmosphere of nitrogen unless otherwise stated. All reactions were ^{1}H NMR spectra taken from reaction samples or liquid chromatography mass spectrometry (LCMS) using an Agilent 6310 Quadrupole Mass Spectrometer. Low-resolution mass spectra (LRMS) were measured on an Agilent 6310 Quadrupole Mass Spectrometer. Infared (IR) spectra were recorded on a Bruker Tensor 27 FT-IR spectrometer as either solids or neat films, either through direct application or deposited in CHCl3, with absorptions reported in wavenumbers (cm-1).. Infrared (IR) spectra were recorded on a Nicolet IS-50 FT-IR spectrometer as either solids or neat films, either through direct application or deposited in $CHCl_3$, with absorptions reported in wavenumbers (cm^{-1}). Analytical thin layer chromatography (TLC) was performed using pre-coated Silicycle glass backed silica gel plates (Silicagel 60 F254). Manual flash column chromatography was undertaken on Silicycle silica gel Siliaflash P60 40-63 mm (230-400 mesh) under a positive pressure of air unless otherwise stated. Automated flash column chromatography was undertaken using a Teledyne Isco CombiFlash NextGen 300+ using 12 g RediSep Gold Normal-Phase Silica cartridges. Visualization was achieved using ultraviolet light (254 nm) and chemical staining with a chamber of I_2 in SiO_2, ceric ammonium molybdate, or basic potassium permanganate solutions as appropriate. Melting points (mp) were recorded using a Büchi B-450 melting point apparatus and are reported uncorrected.

Platinum dioxide (PtO_2), sodium trifluoromethanesulfonate (NaOTf), potassium hexafluorophosphate (KPF_6), 1,3,5-trimethoxybenzene and 1,3,5-trimethylbenzene were purchased from Oakwood and used without further purification. Trifluoromethanesulfonic anhydride (Tf_2O), dibenzylamine ($HNBn_2$), and 2,4,6-trimethylpyridine (collidine) were purchased from Oakwood and used without further purification and were routinely stored in a –20 ºC fridge. d_6-Methanol (CD_3OD, >99%) was purchased from Cambridge Isotope Laboratories and used as received in deuteration experiments. Triphenylmethane (TPM) was purchased from Ambeed and used without further purification. Potassium metabisulfite ($K_2S_2O_5$) was purchased from Thermo Scientific as 97% extra pure and used without further purification or manipulation. Potassium metabisulfite was pulverized prior to use in high throughput experimentation screens.

2. One-Pot Optimization Studies and Limitations

Procedure for Screening One-Pot Pyridinium Formation:

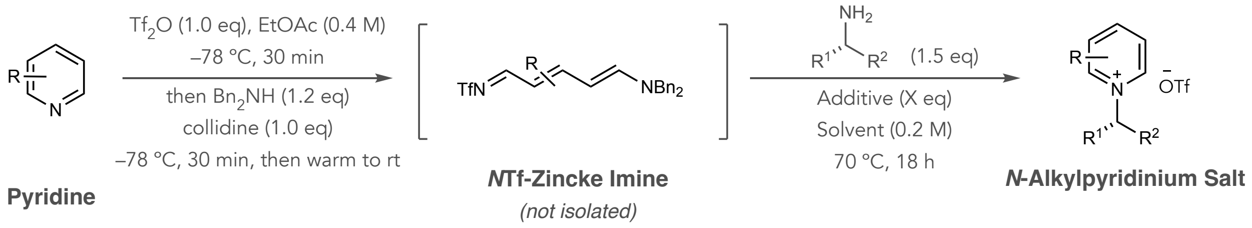

An oven dried 16 mL vial equipped with a stir bar was charged with pyridine (1.0 equiv.) and subjected to three vacuum/nitrogen backfill cycles before being placed under a nitrogen atmosphere. EtOAc (0.4 M) was added, the reaction vessel was cooled to –78 ºC, and Tf_2O (1.0 equiv.) was added dropwise. The reaction was stirred for 30 minutes before collidine (2,4,6-trimethylpyridine) (1.0 equiv.) was added followed by dropwise addition of dibenzylamine (1.2 or 1.0 equiv.). The reaction was stirred for a further 30 minutes at –78 ºC before the cooling bath was removed and the reaction was allowed to warm to room temperature while stirring. Triphenylmethane (1.0 equiv.) was added to the reaction as an internal standard for ^{1}H NMR analysis, the total reaction volume and concentration were determined, and 0.1 mmol aliquots were taken from the reaction and added to 8 mL vials equipped with a stir bar; the remaining material was used for ^{1}H NMR analysis of the Zincke imine formation step (in CD_3CN). The reaction vials were diluted to 0.2 M with EtOAc, charged with the additive (X equiv.) and amine (1.5 equiv.), capped, and heated to 70 ºC. The reactions were cooled to room temperature, and a direct aliquot was taken from each reaction and diluted with CD_3OD for ^{1}H NMR analysis of the crude reaction.

Assay Notes:

- The transamination of Zincke imines leads to complex mixtures of mono- and bis-transaminated Zincke imines that sometimes overlap with the starting Zincke imine. The mixture of starting and transaminated Zincke imines is reported as a combined yield in the tables below. The presence of transaminated Zincke imines was validated by LRMS.

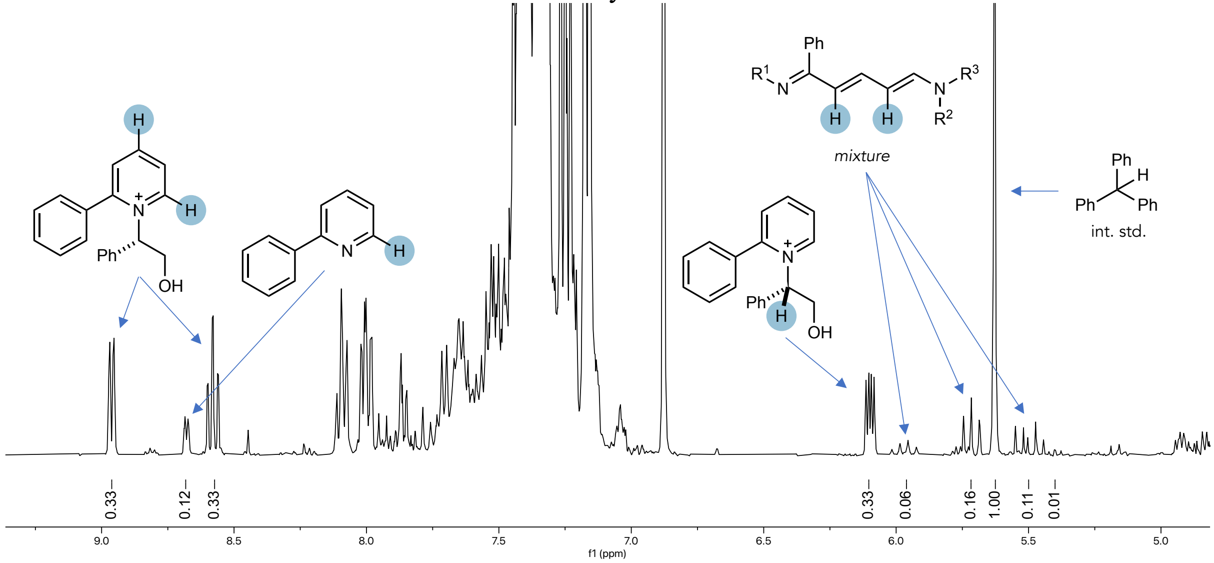

Figure S1. Representative crude ^{1}H NMR (in CD_3OD) of reaction between Zincke imine **1a** and amine **2b** without an additive at 70 ºC (entry 1 in Table S1).

Additive Screening:

Table S1. Additive screen for ring-closing of 1a with amines 2a, 2b, and 2aw.[a]

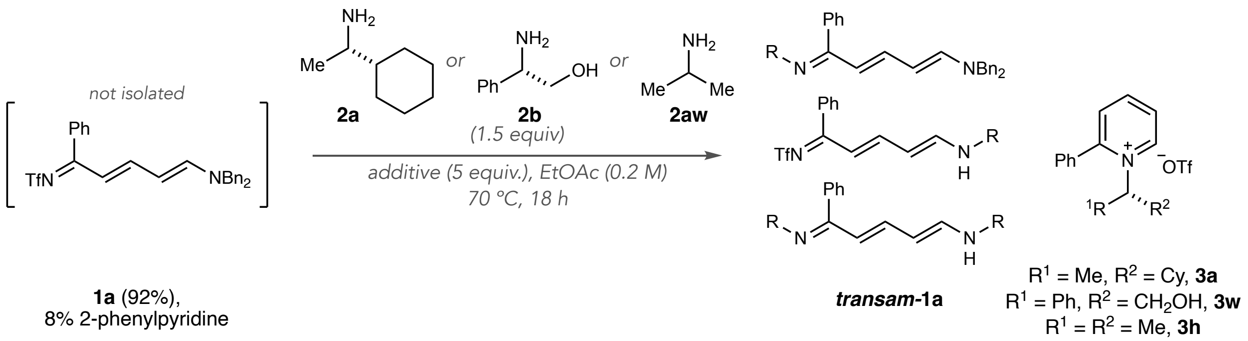

entry	additive	using 2a: % 1a +*transam*-1a	using 2a: % 3a	using 2b: % 1a +*transam*-1a	using 2b: % 3w	using 2aw: % 1a +*transam*-1a	using 2aw: % 3h
1	none	39	**10**	22	**33**	34	**28**
2	$K_2S_2O_5$	0	**90**	0	**72**	0	**89**
3	$Na_2S_2O_5$	-	-	0	**62**	0	**82**
4	K_2SO_3	0	**83**	3	**61**	0	**90**
5	Na_2SO_3	-	-	18	**40**	0	**77**
6	$NaHSO_3$	0	**81**	3	**65**	0	**86**
7	$NaHSO_4$	-	-	11	**3**	5	**6**
8	Na_2CO_3	7	**42**	0	**56**	0	**79**
9	$NaHCO_3$	-	-	10	**44**	0	**74**
10	K_2CO_3	-	-	47	**4**	13	**46**
11	KH_2PO_4	-	-	18	**39**	4	**57**
12	K_2HPO_4	5	**38**	11	**53**	0	**76**
13	K_3PO_4	-	-	0	**0**	56	**10**
14	Na_2HPO_4	-	-	19	**39**	0	**57**
15	NaOAc	-	-	19	**45**	0	**66**
16	DABCO	0	**59**	0	**60**	7	**82**
17	NEt_3	2	**34**	5	**57**	0	**78**
18	TMP	-	-	2	**44**	0	**80**
19	DBU	-	-	0	**0**	39	**6**
20	AcOH	54	**7**	29	**50**	40	**38**
21	Benzoic Acid	56	**9**	25	**46**	35	**28**

[a]Yields determined by ^1H NMR using triphenylmethane as an internal standard in CD_3CN. Dashes indicate the reaction was not run. Maximum yield of pyridinium salts is assumed to be 92%.

Optimization for 2-alkyl pyridines:

Table S2. Optimization of solvent and AcOH equivalents for the ring-closing of 1b with amines 2a and 2b.[a]

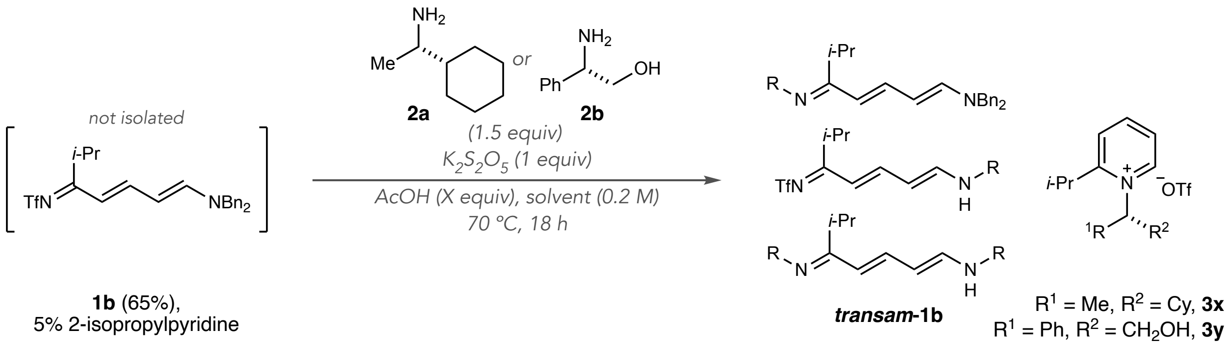

			using 2a		using 2b	
entry	solvent	AcOH equiv	% **1b** +*transam*-**1b**	% **3x**	% **1b** +*transam*-**1b**	% **3y**
1	1:1 EtOAc/MeOH	none	6	**20**	0	**25**
2	1:1 EtOAc/MeOH	1.5	8	**25**	-	-
3	1:1 EtOAc/MeOH	2.0	10	**25**	-	-
4	1:1 EtOAc/MeOH	2.5	10	**26**	-	-
5	1:1 EtOAc/MeOH	5.0	10	**29**	-	-
6	1:1 EtOAc/MeOH	10	0	**29**	0	**42**
7	EtOAc	none	0	**19**	0	**34**
8	EtOAc	1.5	14	**19**	-	-
9	EtOAc	2.0	12	**25**	-	-
10	EtOAc	2.5	8	**28**	-	-
11	EtOAc	5.0	5	**27**	-	-
12	EtOAc	10	0	**35**	0	**57**

[a]Yields determined by ^{1}H NMR using triphenylmethane as an internal standard in CD_3CN. Dashes indicate the reaction was not run. Maximum yield of pyridinium salts is assumed to be 65%.

Exploration of Other Heterocycles in One-Pot Azinium Formation

- We used the procedure described at the beginning of this section to assess various heterocycles in the one-pot azinium formation reaction. We found that pyridine, pyrazine, and isoquinoline are amenable this procedure. Currently, pyrimidine and quinoline are limitations in this procedure.

Table S3. Exploration of heterocycles in one-pot ring-opening, ring-closing sequence to form azinium salts.[a]

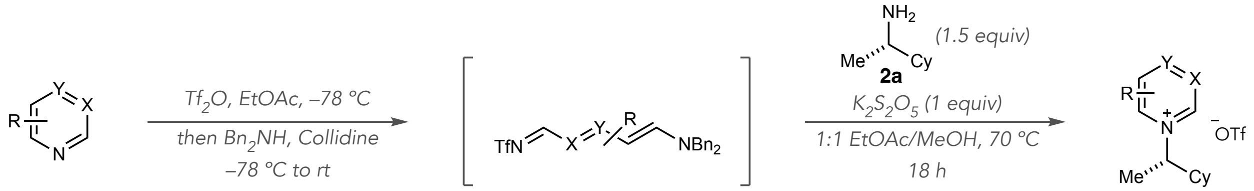

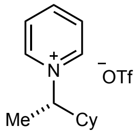

Azine Starting Material	Azinium
37%	63%

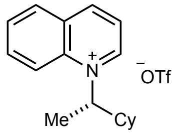

Azine Starting Material	Azinium
40%	0%

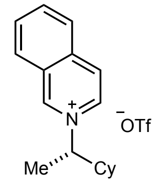

Azine Starting Material	Azinium
47%	56%

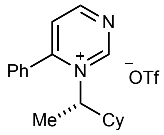

Azine Starting Material	Azinium
97%	0%

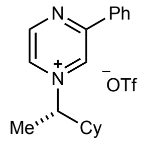

Azine Starting Material	Azinium
14%	37%

[a]Yields determined by ^{1}H NMR using 1,3,5-trimethylbenzene as an internal standard in CD_3OD.

Limitations and Challenges for Pyridines and Azines in One-Pot Pyridinium Formation

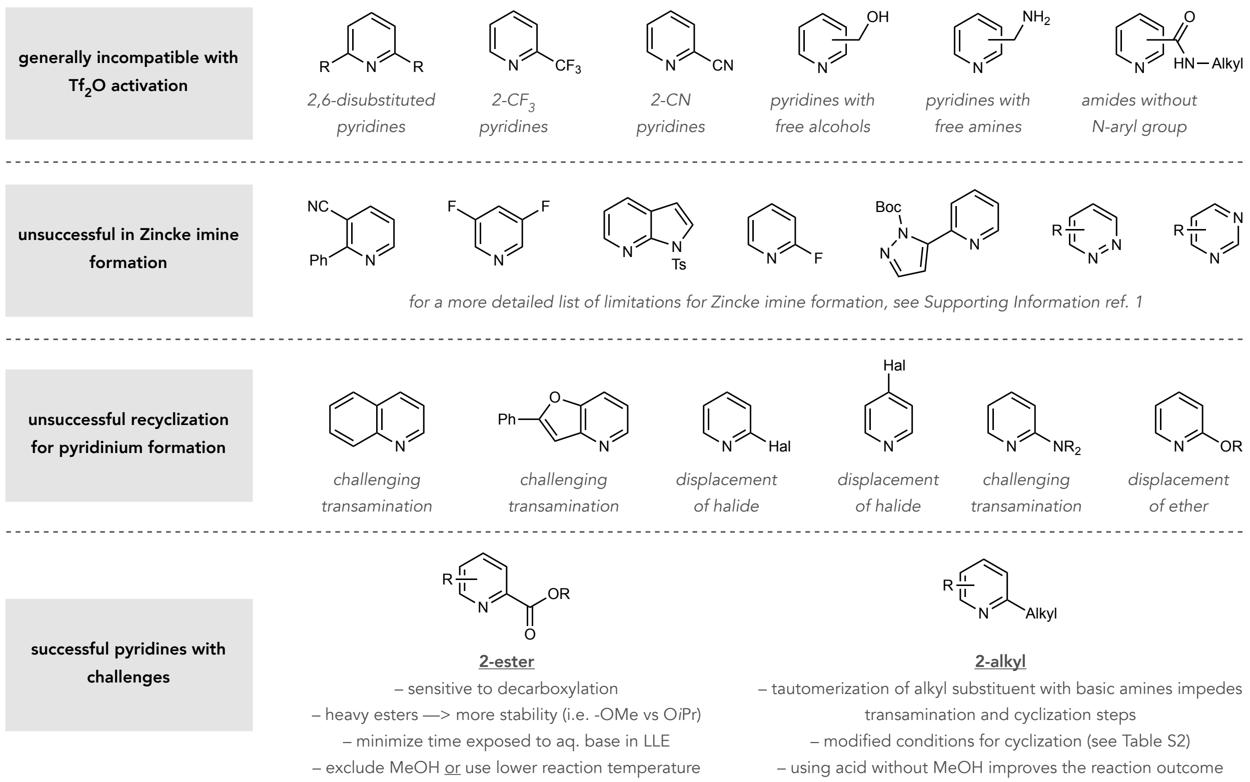

Figure S2. Limitations and challenges for pyridines and other azines in one-pot pyridinium formation via Zincke imine intermediates.

3. Preparation of Starting Materials

tert-Butyl 4-(3-fluoropyridin-2-yl)piperidine-1-carboxylate

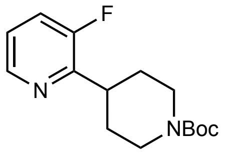

An oven dried 350 mL pressure tube equipped with a stir bar was charged with 2-bromo-3-fluoropyridine (2.02 mL, 20.0 mmol), *tert*-butyl 4-(4,4,5,5-tetramethyl-1,3,2-dioxaborolan-2-yl)-3,6-dihydropyridine-1(2H)-carboxylate (6.80 g, 22.0 mmol), Na_2CO_3 (10.6 g, 100 mmol). The reaction vessel was placed under a nitrogen atmosphere and tetrakis(triphenylphosphine)palladium(0) was added (231 mg, 0.200 mmol). Degassed 1,2-dimethoxyethane (65.0 mL) and degassed H_2O (33.0 mL) were added, the reaction vessel was sealed, and the mixture was heated to 110 ºC for 18 h. The reaction was cooled to room temperature and diluted with H_2O and EtOAc. The layers were separated, and the aqueous layer was extracted with EtOAc (x2). The combined organic extractions were dried over Na_2SO_4, filtered, and concentrated *in vacuo*. The residue was subjected to automated flash chromatography (silica gel: 20% EtOAc in hexanes). The resulting impure residue was dissolved in MeOH (100 mL, 0.2 M) and transferred to an oven dried 250 mL round bottom flask equipped with a stir bar. The mixture was charged with Pd/C (10 mol%, 2.00 mmol), the reaction vessel was sealed, and a double-skinned balloon of hydrogen gas was bubbled through the

solution while stirring (550 rpm). After the balloon was depleted, it was replaced with a fresh, double-skinned balloon filled with hydrogen gas hovering in the headspace of the reaction flask. The reaction was monitored by LCMS until full conversion of the starting material was observed. The balloon was removed, and the reaction mixture was filtered through Celite and concentrated *in vacuo*. The residue was dissolved in CH_2Cl_2 and washed with 1.0 M NaOH (x2), dried, filtered, and concentrated *in vacuo* to afford the title compound as a white solid (4.92 g, 17.6 mmol, 88% yield). mp 45 – 48 ºC; ^{1}H NMR (400 MHz, $CDCl_3$) δ: 8.36 (dt, J = 4.7, 1.5 Hz, 1H), 7.33 (ddd, J = 9.9, 8.3, 1.4 Hz, 1H), 7.15 (dt, J = 8.5, 4.4 Hz, 1H), 4.26 (br. s, 2H), 3.41 – 3.06 (m, 1H), 2.86 (br. s, 2H), 1.99 – 1.70 (m, 4H), 1.47 (s, 9H); ^{13}C NMR (100 MHz, $CDCl_3$) δ: 157.32 (d, J = 256.2 Hz), 154.83, 152.33 (d, J = 14.1 Hz), 144.99 (d, J = 5.4 Hz), 122.82 (d, J = 19.8 Hz), 122.66 (d, J = 3.8 Hz), 79.46, 37.73, 37.71, 30.22, 28.59; ^{19}F NMR (375 MHz, $CDCl_3$) δ: –126.75; *m/z* LRMS (ESI + APCI) found $[M + H]^+$ 281.2, $C_{15}H_{22}FN_2O_2^+$ requires 281.2.

(*R*)-2-(4-(But-3-yn-2-yloxy)phenyl)pyridine

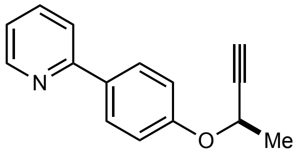

An oven dried 250 mL round bottom flask equipped with a stir bar was charged with 4-(pyridine-2-yl)phenol (5.65 g, 33.0 mmol) and triphenylphosphine (8.66 g, 33.0 mmol) and placed under a nitrogen atmosphere. THF (150 mL, 0.2 M) and (*S*)-but-3-yn-2-ol (2.36 mL, 30.0 mmol) and the reaction vessel was placed in a 0 ºC ice bath. Diisopropyl azodicarboxylate (DIAD) (6.50 mL, 33.0 mmol) was added dropwise, the ice bath was removed, and the reaction was allowed to warm to room temperature while stirring overnight. The reaction was concentrated *in vacuo*, and the resulting residue was dissolved in EtOAc. The organic layer was washed with 1.0 M NaOH (x1), brine (x1), dried over Na_2SO_4, filtered, and concentrated *in vacuo*. Automated flash chromatography (silica gel: 0% to 20% EtOAc in hexanes) afforded the title compound as a tan solid (5.38 g, 24.1 mmol, 80% yield). mp 55 – 58 ºC; IR ν_{max}/cm^{-1} (film): 3296, 3062, 2992, 1602, 1465, 1234, 1037, 775; ^{1}H NMR (400 MHz, $CDCl_3$) δ:: 8.66 (ddd, J = 4.8, 1.8, 1.0 Hz, 1H), 7.96 (d, J = 8.9 Hz, 2H), 7.80 – 7.63 (m, 2H), 7.18 (ddd, J = 7.1, 4.8, 1.4 Hz, 1H), 7.11 (d, J = 8.8 Hz, 2H), 4.95 (qd, J = 6.6, 2.0 Hz, 1H), 2.49 (d, J = 2.0 Hz, 1H), 1.70 (d, J = 6.6 Hz, 3H); ^{13}C NMR (100 MHz, $CDCl_3$) δ:: 158.32, 157.11, 149.55, 136.95, 132.69, 128.25, 121.66, 120.08, 116.03, 82.85, 74.18, 63.64, 22.26; *m/z* LRMS (ESI + APCI) found $[M + H]^+$ 224.1, $C_{15}H_{14}NO^+$ requires 224.1.

4. Preparation of Zincke Imines

General Procedure A (Preparation and Isolation of Zincke Imines)

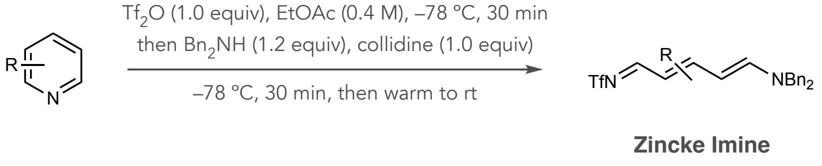

An oven dried round bottom flask equipped with a stir bar was charged with heterocycle (1.0 equiv.) and subjected to three vacuum/nitrogen backfill cycles before being placed under a nitrogen atmosphere. EtOAc (0.2 M or 0.4 M) was added, the reaction vessel was cooled to –78 ºC, and Tf_2O (1.0 equiv.) was added dropwise. The reaction was stirred for 30 minutes before collidine (2,4,6-trimethylpyridine) (1.0 equiv.) was added followed by dropwise addition of dibenzylamine (1.2 equiv.). The reaction was stirred for a

further 30 minutes at –78 ºC before the cooling bath was removed and the reaction was allowed to warm to room temperature while stirring. The reaction was diluted with EtOAc then washed with H_2O (x2), sat. Na_2CO_3 (x3), and brine (x1). The organic extract was dried over Na_2SO_4, filtered, and concentrated *in vacuo*. The crude residue was redissolved in minimal CH_2Cl_2 and added dropwise to hexanes (approx. 50 mL per 1.0 mmol) and stirred at room temperature overnight. If product was an oil, the hexanes was decanted off and the oil was dissolved in CH_2Cl_2 and concentrated *in vacuo* to provide the pure "Zincke imine." If product was a precipitate, it was collected via filtration and dried *in vacuo* to provide the pure "Zincke imine."

Reaction Notes:

- The reaction is sensitive to excess Tf_2O (use of 1.2 equiv. results in substantial yield loss).
- Stirring is critical to achieve consistent yields; recommended stirring 500-750 rpm.
- Higher reaction concentrations (0.4 M) are more likely to form gels at –78 ºC after adding Tf_2O. After adding the collidine and dibenzylamine, the reaction can be temporarily removed from the bath and swirled/mixed until it becomes homogenous and is able to stir. Once stirring, return the reaction flask to the –78 ºC ice bath.
- Adding collidine before dibenzylamine in the reaction sequence has either provided higher yields for some substrates or is otherwise the same for others as adding dibenzylamine first. Pyridines with a 2- and/or 4-alkyl substituent are an exception; add dibenzylamine before collidine in these instances.
- Substrates without a 2-substituent (and unsubstituted pyridine) are susceptible to "bis-adduct" formation and care should be taken prior to precipitation. Concentrating *in vacuo* without heat can minimize yield loss to Zincke iminiums.
- Substrates without a 2-substituent (and unsubstituted pyridine) can be run with 1.0 equiv. of Bn_2NH to minimize potential for Zincke iminium product formation.
- If Zincke iminium is observed, a short plug of silica (2-3 inches) and appropriate solvent eluent can remove the impurity.

N-((1*Z*,2*E*,4*E*)-5-(Dibenzylamino)-1-phenylpenta-2,4-dien-1-ylidene)-1,1,1-trifluoromethanesulfonamide (1a)

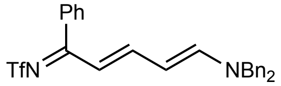

Prepared according to general procedure A using 2-phenylpyridine (7.14 mL, 50.0 mmol), EtOAc (125 mL, 0.4 M), Tf_2O (8.41 mL, 50.0 mmol), collidine (6.60 mL, 50.0 mmol), and dibenzylamine (11.5 mL, 60.0 mmol). Washing with H_2O (x2), sat. Na_2CO_3 (x1), brine (x1) and precipitating from hexanes (2.50 L) afforded the title compound (21.7 g, 44.8 mmol, 90% yield) as a red/brown solid. ^{1}H NMR (400 MHz, $CDCl_3$) δ:: 7.62 – 7.56 (m, 2H), 7.53 – 7.46 (m, 1H), 7.46 – 7.26 (m, 10H), 7.15 (dd, *J* = 7.6, 1.9 Hz, 4H), 6.71 (d, *J* = 13.7 Hz, 1H), 5.84 (t, *J* = 12.1 Hz, 1H), 4.44 (s, 4H); *m/z* LRMS (ESI + APCI) found [M + H]+ 485.2, $C_{26}H_{24}F_3N_2O_2S^+$ requires 485.2.

These data are consistent with those previously reported in the literature.[1]

N-((1*E*,2*E*,4*E*)-5-(Dibenzylamino)-2-phenylpenta-2,4-dien-1-ylidene)-1,1,1-trifluoromethanesulfonamide (1m)

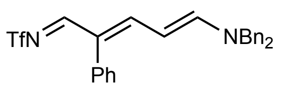

Prepared according to general procedure A using 3-phenylpyridine (2.85 mL, 20.0 mmol), EtOAc (50.0 mL, 0.4 M), Tf_2O (3.37 mL, 20.0 mmol), collidine (2.64 mL, 20.0 mmol), and dibenzylamine (3.85 mL, 20.0 mmol). Washing with H_2O (x2), sat. Na_2CO_3 (x1), brine (x1) and precipitating from hexanes (1.00 L) afforded the title compound as a yellow solid (8.23 g, 17.0 mmol, 85% yield). ^{1}H NMR (400 MHz, CD_3CN) δ: 8.19 (s, 1H), 7.92 (d, *J* = 11.9 Hz, 1H), 7.68 (d, *J* = 13.0 Hz, 1H), 7.46 – 7.36 (m, 3H), 7.36 – 7.27 (m, 8H), 7.13 – 6.99 (m, 4H), 5.73 (t, *J* = 12.5 Hz, 1H), 4.72 (s, 2H), 4.43 (s, 2H); *m/z* LRMS (ESI + APCI) found $[M + H]^+$ 485.1, $C_{26}H_{24}F_3N_2O_2S^+$ requires 485.2.

These data are consistent with those previously reported in the literature.[1]

***N*-((1*E*,2*Z*,4*E*)-5-(Dibenzylamino)-3-phenylpenta-2,4-dien-1-ylidene)-1,1,1-trifluoromethanesulfonamide (1n)**

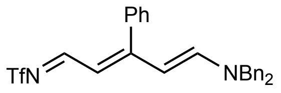

Prepared according to general procedure A using 4-phenylpyridine (1.55 g, 10.0 mmol), EtOAc (50.0 mL, 0.2 M), Tf_2O (1.68 mL, 10.0 mmol), collidine (1.32 mL, 10.0 mmol), and dibenzylamine (1.92 mL, 10.0 mmol). Washing with H_2O (x2), sat. Na_2CO_3 (x1), brine (x1) and precipitating from hexanes (500 mL) afforded the title compound as an orange solid (3.87 g, 7.99 mmol, 80% yield). mp 149 – 152 ºC; IR ν_{max}/cm^{-1} (film): 3033, 1633, 1534, 1504, 1314, 1173, 1147, 1094; ^{1}H NMR (400 MHz, CD_3CN) δ: 7.61 (d, *J* = 11.9 Hz, 1H), 7.55 – 7.20 (m, 14H), 7.13 (br. s, 2H), 6.24 (d, *J* = 12.1 Hz, 2H), 4.67 (s, 2H), 4.50 (s, 2H); ^{13}C NMR (100 MHz, CD_3CN) δ: 177.16, 170.72, 160.88, 135.87, 135.13, 130.68, 130.49, 129.98, 129.94, 129.63, 129.54, 129.47, 129.34, 129.12, 128.44, 121.15 (q, *J* = 322.6 Hz), 116.81, 107.92, 61.09, 53.46; ^{19}F NMR (375 MHz, CD_3CN) δ: –79.35; *m/z* LRMS (ESI + APCI) found $[M + H]^+$ 485.2, $C_{26}H_{24}F_3N_2O_2S^+$ requires 485.2.

***N*-((1*E*,2*E*,4*E*)-5-(Dibenzylamino)-2-methylpenta-2,4-dien-1-ylidene)-1,1,1-trifluoromethanesulfonamide (1o)**

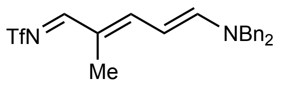

Prepared according to general procedure A using 3-methylpyridine (490 μL, 5.00 mmol), EtOAc (25.0 mL, 0.2 M), Tf_2O (840 μL, 5.00 mmol), collidine (660 μL, 5.00 mmol), and dibenzylamine (960 μL, 5.00 mmol). Washing with H_2O (x2), sat. Na_2CO_3 (x1), brine (x1) and precipitating from hexanes (250 mL) afforded the title compound as a yellow solid (1.51 g, 3.57 mmol, 72% yield). mp 132 – 134 ºC; IR ν_{max}/cm^{-1} (film): 3073, 1625, 1573, 1512, 1454, 1320, 1179, 1109; ^{1}H NMR (400 MHz, CD_3CN) δ: 8.02 (s, 1H), 7.86 (d, *J* = 11.8 Hz, 1H), 7.55 (d, *J* = 12.8 Hz, 1H), 7.47 – 7.27 (m, 8H), 7.27 – 7.18 (m, 2H), 6.01 (dd, *J* = 12.9, 11.9 Hz, 1H), 4.66 (s, 2H), 4.63 (s, 2H), 1.78 (s, 3H); ^{13}C NMR (100 MHz, CD_3CN) δ: 172.19, 167.92, 163.03, 135.59, 135.26, 129.99, 129.87, 129.60, 129.38, 129.07, 128.52, 121.86, 121.27 (q, *J* = 323.0 Hz), 103.57, 61.16, 52.95, 10.48; ^{19}F NMR (377 MHz, CD_3CN) δ: –79.35; *m/z* LRMS (ESI + APCI) found $[M + H]^+$ 423.2, $C_{21}H_{22}F_3N_2O_2S^+$ requires 423.1.

N-((1*Z*,2*E*,4*E*)-5-(Dibenzylamino)-2-methyl-1-phenylpenta-2,4-dien-1-ylidene)-1,1,1-trifluoromethanesulfonamide (1p)

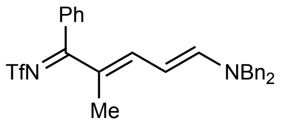

Prepared according to general procedure A using 3-methyl-2-phenylpyridine (1.60 mL, 10.0 mmol), EtOAc (25.0 mL, 0.2 M), Tf_2O (1.68 mL, 10.0 mmol), collidine (1.32 mL, 10.0 mmol), and dibenzylamine (2.31 mL, 12.0 mmol). Washing with H_2O (x2), sat. Na_2CO_3 (x1), brine (x1) and precipitating from hexanes (500 mL) afforded the title compound as a yellow solid (4.53 g, 9.09 mmol, 91% yield). ^{1}H NMR (400 MHz, CD_3CN) δ:: 7.60 – 7.41 (m, 4H), 7.40 – 7.28 (m, 8H), 7.28 – 7.17 (m, 4H), 7.13 (d, *J* = 12.5 Hz, 1H), 5.87 (t, *J* = 12.2 Hz, 1H), 4.53 (d, *J* = 9.7 Hz, 4H), 1.90 (s, 3H); *m/z* LRMS (ESI + APCI) found $[M + H]^+$ 499.3, $C_{27}H_{26}F_3N_2O_2S^+$ requires 499.2.

These data are consistent with those previously reported in the literature.[2]

N-((1*Z*,2*E*,4*E*)-5-(Dibenzylamino)-3-methyl-1-phenylpenta-2,4-dien-1-ylidene)-1,1,1-trifluoromethanesulfonamide (1q)

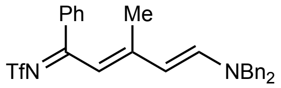

Prepared according to general procedure A using 4-methyl-2-phenylpyridine (238 mg, 1.40 mmol), EtOAc (7.00 mL, 0.2 M), Tf_2O (240 μL, 1.40 mmol), collidine (190 μL, 1.40 mmol), and dibenzylamine (320 μL, 1.68 mmol). Washing with H_2O (x2), sat. Na_2CO_3 (x1), brine (x1) and precipitating from hexanes (150 mL) afforded the title compound as a red solid (297 mg, 0.596 mmol, 43% yield). ^{1}H NMR (400 MHz, CD_3CN) δ:: 8.21 (d, *J* = 12.6 Hz, 1H), 7.53 – 7.48 (m, 2H), 7.46 – 7.32 (m, 10H), 7.26 (brs, 4H), 5.71 (s, 1H), 4.58 (brs, 4H), 2.36 (s, 3H); *m/z* LRMS (ESI + APCI) found $[M + H]^+$ 499.3, $C_{27}H_{26}F_3N_2O_2S^+$ requires 499.2.

These data are consistent with those previously reported in the literature.[2]

N-((1*Z*,2*E*,4*E*)-5-(Dibenzylamino)-4-methyl-1-phenylpenta-2,4-dien-1-ylidene)-1,1,1-trifluoromethanesulfonamide (1r)

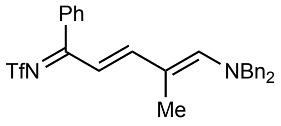

Prepared according to general procedure A using 5-methyl-2-phenylpyridine (1.64 mL, 10.0 mmol), EtOAc (25.0 mL, 0.4 M), Tf_2O (1.68 mL, 10.0 mmol), collidine (1.32 mL, 10.0 mmol), and dibenzylamine (2.31 mL, 12.0 mmol). Washing with H_2O (x2), sat. Na_2CO_3 (x1), brine (x1) and precipitating from hexanes (500 mL) afforded the title compound as an orange solid (2.10 g, 4.21 mmol, 42% yield). mp 129 – 132 ºC; IR ν_{max}/cm^{-1} (film): 3030, 1614, 1540, 1450, 1301, 1171, 1095, 1020; ^{1}H NMR (400 MHz, DMSO) δ: 8.19 (s, 1H), 7.66 – 7.45 (m, 6H), 7.44 – 7.22 (m, 10H), 6.47 (d, *J* = 13.1 Hz, 1H), 4.79 (s, 4H), 1.88 (s, 3H); ^{13}C NMR (100 MHz, DMSO) δ: 165.49, 164.20, 138.67, 135.30, 130.54, 129.02, 129.02, 128.92, 128.84,

128.29, 128.07, 127.29, 119.89 (d, J = 323.0 Hz), 112.81, 108.87, 38.97, 11.70; ^{19}F NMR (377 MHz, DMSO) δ: –78.21; *m/z* LRMS (ESI + APCI) found $[M + H]^+$ 499.6, $C_{27}H_{26}F_3N_2O_2S^+$ requires 499.6.

***N*-((3*E*,4*E*,6*E*)-7-(Dibenzylamino)-2-methylhepta-4,6-dien-3-ylidene)-1,1,1-trifluoromethanesulfonamide (1b)**

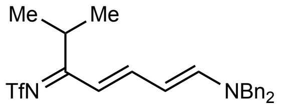

Prepared according to general procedure A using 2-isopropylpyridine (1.32 mL, 10.0 mmol), EtOAc (25.0 mL, 0.4 M), Tf_2O (1.68 mL, 10.0 mmol), dibenzylamine (2.31 mL, 12.0 mmol), and collidine (1.32 mL, 10.0 mmol). Washing with H_2O (x2), sat. Na_2CO_3 (x1), brine (x1) and precipitating from hexanes (500 mL) afforded the title compound (3.84 g, 8.52 mmol, 85% yield) as a yellow solid. ^{1}H NMR (400 MHz, CD_3CN) δ:: 7.84 (t, J = 12.9 Hz, 1H), 7.75 (d, J = 12.1 Hz, 1H), 7.45 – 7.32 (m, 6H), 7.29 (d, J = 7.3 Hz, 2H), 7.22 (d, J = 7.4 Hz, 2H), 6.23 (d, J = 13.7 Hz, 1H), 5.88 (t, J = 12.1 Hz, 1H), 4.57 (d, J = 30.3 Hz, 4H), 3.33 (p, J = 6.7 Hz, 1H), 1.12 (d, J = 6.5 Hz, 6H); *m/z* LRMS (ESI + APCI) found $[M + H]^+$ 451.3, $C_{23}H_{26}F_3N_2O_2S^+$ requires 451.2.

These data are consistent with those previously reported in the literature.[2]

***N*-((3*E*,4*E*,6*E*)-7-(Dibenzylamino)hepta-4,6-dien-3-ylidene)-1,1,1-trifluoromethanesulfonamide (1c)**

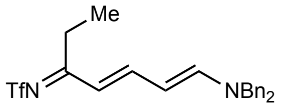

Prepared according to general procedure A using 2-ethylpyridine (3.43 mL, 30.0 mmol), EtOAc (75.0 mL, 0.4 M), Tf_2O (5.04 mL, 30.0 mmol), dibenzylamine (6.92 mL, 36.0 mmol), and collidine (3.96 mL, 30.0 mmol). Washing with H_2O (x2), sat. Na_2CO_3 (x1), brine (x1) and precipitating from hexanes (1.50 mL) afforded the title compound (10.9 g, 25.0 mmol, 83% yield) as an orange solid. mp 117 – 120 ºC; IR ν_{max}/cm^{-1} (film): 3062, 2940, 1577, 1496, 1431, 1236, 1180, 776; ^{1}H NMR (400 MHz, CD_3CN) δ: 7.93 – 7.70 (m, 2H), 7.47 – 7.17 (m, 10H), 6.15 (d, J = 13.6 Hz, 1H), 5.88 (t, J = 12.2 Hz, 1H), 4.60 (s, 2H), 4.53 (s, 2H), 2.65 (q, J = 7.5 Hz, 2H), 1.17 (t, J = 7.5 Hz, 3H); ^{13}C NMR (100 MHz, CD_3CN) δ: 184.00, 161.46, 158.86, 136.14, 135.69, 129.94, 129.82, 129.43, 129.25, 128.89, 128.31, 120.65 (q, J = 319.8 Hz), 113.06, 104.09, 60.87, 52.64, 29.97, 13.50; ^{19}F NMR (375 MHz, CD_3CN) δ: –80.63; *m/z* LRMS (ESI + APCI) found $[M + H]^+$ 437.1, $C_{22}H_{24}F_3N_2O_2S^+$ requires 437.2.

***N*-((1*Z*,2*E*,4*E*)-5-(Dibenzylamino)-1-(thiophen-3-yl)penta-2,4-dien-1-ylidene)-1,1,1-trifluoromethanesulfonamide (1d)**

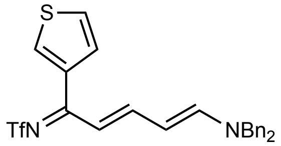

Prepared according to general procedure A using 2-(thiophen-3-yl)pyridine (806 mg, 5.00 mmol), EtOAc (12.5 mL, 0.4 M), Tf_2O (840 μL, 5.00 mmol), collidine (660 μL, 5.00 mmol), and dibenzylamine (1.15 mL, 6.00 mmol). Washing with H_2O (x2), sat. Na_2CO_3 (x1), brine (x1) and precipitating from hexanes (250 mL) afforded the title compound (2.02 g, 4.12 mmol, 82% yield) as a red solid. ^{1}H NMR (400 MHz, CD_3CN) δ:: 7.91 – 7.74 (m, 3H), 7.47 (dd, *J* = 5.1, 3.0 Hz, 1H), 7.42 – 7.33 (m, 7H), 7.29 (d, *J* = 8.0 Hz, 2H), 7.22 (d, *J* = 7.4 Hz, 2H), 6.54 (d, *J* = 13.2 Hz, 1H), 6.09 (t, *J* = 12.2 Hz, 1H), 4.59 (d, *J* = 22.3 Hz, 4H); *m/z* LRMS (ESI + APCI) found $[M + H]^+$ 491.2, $C_{24}H_{22}F_3N_2O_2S_2^+$ requires 491.1.

These data are consistent with those previously reported in the literature.[2]

***N*-((1*E*,2*E*,4*E*)-5-(Dibenzylamino)-2-(6-(trifluoromethyl)pyridin-3-yl)penta-2,4-dien-1-ylidene)-1,1,1-trifluoromethanesulfonamide (1e) (major and minor)**

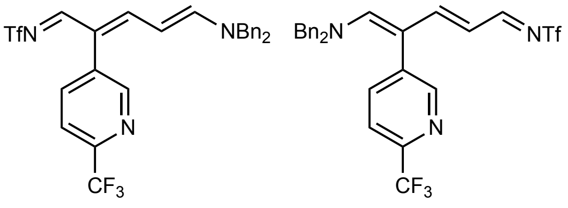

14.3 *1.0*

Prepared according to general procedure A using 6-(trifluoromethyl)-3,3'-bipyridine (1.12 g, 5.00 mmol), EtOAc (25 mL, 0.2 M), Tf_2O (840 μL, 5.00 mmol), dibenzylamine (960 μL, 5.00 mmol, 1.0 M in EtOAc), and collidine (661 μL, 5.00 mmol). Washing with H_2O (x2), saturated aqueous $NaHCO_3$, and precipitating dropwise from hexanes (500 mL) afforded the title compound in a mixture of regioisomers (14.3:1) as an orange solid (2.59 g, 4.68 mmol, 94% yield). ^{1}H NMR (major, 400 MHz, CD_3CN) δ:: 8.50 (s, 1H), 8.28 (s, 1H), 7.99 (d, *J* = 11.6 Hz, 1H), 7.88 – 7.60 (m, 3H), 7.45 – 7.38 (m, *J* = 6.8 Hz, 3H), 7.36 – 7.29 (m, 5H), 7.09 – 7.01 (m, 2H), 5.76 (t, *J* = 12.5 Hz, 1H), 4.72 (s, 2H), 4.50 (s, 2H); *m/z* LRMS (ESI + APCI) found $[M + H]^+$ 554.2, $C_{26}H_{22}F_6N_3O_2S^+$ requires 554.1.

These data are consistent with those previously reported in the literature.[2]

Methyl (2*Z*,4*E*)-5-(dibenzylamino)-2-((*E*)-(((trifluoromethyl)sulfonyl)imino)methyl)penta-2,4-dienoate (1f)

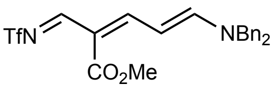

Prepared according to general procedure A using methyl nicotinate (686 mg, 5.00 mmol), EtOAc (12.5 mL, 0.4 M), Tf_2O (840 μL, 5.00 mmol), collidine (660 μL, 5.00 mmol), and dibenzylamine (960 μL, 5.00 mmol). Washing with H_2O (x2), sat. Na_2CO_3 (x1), brine (x1) and precipitating from hexanes (250 mL) provided an impure residue. Flash chromatography (silica gel: 0% to 1% MeOH in CH_2Cl_2) afforded the title compound as a yellow solid (1.34 g, 2.87 mmol, 58% yield). mp 108 – 111 ºC; IR ν_{max}/cm^{-1} (film): 2999, 1685, 1611, 1586, 1486, 1167, 1116, 844; ^{1}H NMR (400 MHz, CD_3CN) δ: 8.78 (s, 1H), 8.31 (d, *J* = 11.6 Hz, 1H), 8.16 (d, *J* = 14.3 Hz, 1H), 7.49 – 7.32 (m, 10H), 7.30 – 7.20 (m, 2H), 4.82 (s, 2H), 4.70 (s, 2H), 3.71 (s, 3H); ^{13}C NMR (100 MHz, CD_3CN) δ: 170.12, 167.87, 158.18, 134.17, 134.15, 130.12, 130.04, 130.00, 129.83, 129.60, 129.46, 128.74, 121.03 (q, *J* = 322.2 Hz), 110.61, 107.61, 62.75, 54.27, 51.97; ^{19}F NMR (375 MHz, CD_3CN) δ: –79.38; *m/z* LRMS (ESI + APCI) found $[M + H]^+$ 467.6, $C_{22}H_{22}F_3N_2O_4S^+$ requires 467.5.

N-((1*E*,2*E*,4*Z*)-5-(Dibenzylamino)-4-(trifluoromethyl)penta-2,4-dien-1-ylidene)-1,1,1-trifluoromethanesulfonamide (1g) (major and minor)

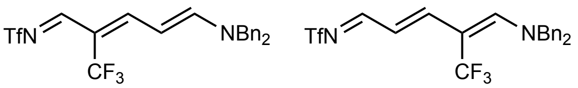

3.2 *1.0*

Prepared according to general procedure A using 3-(trifluoromethyl)pyridine (580 μL, 5.00 mmol), EtOAc (12.5 mL, 0.4 M), Tf_2O (840 μL, 5.00 mmol), collidine (660 μL, 5.00 mmol), and dibenzylamine (960 μL, 5.00 mmol). Washing with H_2O (x2), sat. Na_2CO_3 (x1), brine (x1) and precipitating from hexanes (250 mL) afforded the title compound (2.02 g, 4.12 mmol, 82% yield) as a yellow solid. ^{1}H NMR (major and minor, 400 MHz, CD_3CN) δ: 8.32 (d, *J* = 11.2 Hz, 1H, major), 8.17 (s, 1H, major), 8.01 (d, *J* = 11.6 Hz, 1H, minor), 7.51 – 7.31 (m, 10H, major and minor), 7.29 – 7.18 (m, 2H, major and minor), 4.82 (s, 2H, major and minor), 4.73 (s, 2H, major and minor); *m/z* LRMS (ESI + APCI) found $[M + H]^+$ 477.1, $C_{21}H_{19}F_6N_2O_2S^+$ requires 477.1.

These data are consistent with those previously reported in the literature.[3]

iso-Propyl (2*Z*,3*E*,5*E*)-6-(ibenzylamino)-3-methyl-2-(((trifluoromethyl)sulfonyl)imino)hexa-3,5-dienoate (1h)

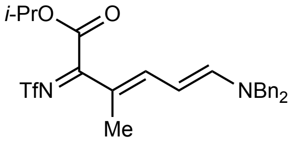

Prepared according to general procedure A using isopropyl 3-methylpicolinate (1.79 mL, 10.0 mmol), EtOAc (50 mL, 0.2 M), Tf_2O (1.68 mL, 10.0 mmol), dibenzylamine (1.93 mL, 10.0 mmol, 1.0 M in EtOAc), and collidine (1.32 mL, 10.0 mmol). Washing with H_2O (x2), saturated aqueous $NaHCO_3$, and precipitating dropwise from hexanes (1.0 L) afforded the title compound (3.60 g, 7.08 mmol, 71% yield) as a yellow solid. ^{1}H NMR (400 MHz, CD_3CN) δ:: 8.02 (d, *J* = 11.6 Hz, 1H), 7.50 (d, *J* = 12.9 Hz, 1H), 7.45 – 7.28 (m, 8H), 7.23 (d, *J* = 8.1 Hz, 2H), 6.17 (t, *J* = 12.2 Hz, 1H), 5.19 (p, *J* = 6.3 Hz, 1H), 4.68 (s, 4H), 1.80 (s, 3H), 1.35 (d, *J* = 6.3 Hz, 6H); *m/z* LRMS (ESI + APCI) found $[M + H]^+$ 509.3, $C_{25}H_{28}F_3N_2O_4S^+$ requires 509.2.

These data are consistent with those previously reported in the literature.[2]

Tert-butyl 4-((1*E*,2*Z*,4*E*)-5-(Dibenzylamino)-2-fluoro-1-(((trifluoromethyl)sulfonyl)imino)penta-2,4-dien-1-yl)piperidine-1-carboxylate (1i).

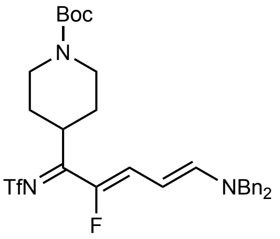

Prepared according to general procedure A using *tert*-butyl 4-(3-fluoropyridin-2-yl)piperidine-1-carboxylate (1.40 g, 5.00 mmol), EtOAc (12.5 mL, 0.4 M), Tf_2O (840 μL, 5.00 mmol), dibenzylamine (1.15 mL, 6.00 mmol), and collidine (660 μL, 5.00 mmol). Washing with H_2O (x2), sat. Na_2CO_3 (x1), brine (x1) and precipitating from hexanes (250 mL) afforded the title compound as a yellow solid (806 mg, 1.32 mmol, 26% yield). mp 169 – 172 ºC; IR ν_{max}/cm^{-1} (film): 2978, 1688, 1580, 1476, 1442, 1152, 1087, 942; ^1H NMR (400 MHz, CD_3CN) δ: 7.82 (d, *J* = 12.1 Hz, 1H), 7.60 (dd, *J* = 31.7, 12.4 Hz, 1H), 7.49 – 7.28 (m, 8H), 7.25 (d, *J* = 6.6 Hz, 2H), 5.94 (t, *J* = 12.2 Hz, 1H), 4.67 (s, 2H), 4.64 (s, 2H), 4.10 (d, *J* = 13.3 Hz, 2H), 2.98 – 2.89 (m, 1H), 2.81-2.71 (s, 2H), 1.70 – 1.49 (m, 4H), 1.43 (s, 9H); ^{13}C NMR (100 MHz, CD_3CN) δ: 167.22 (d, *J* = 14.6 Hz), 161.00, 155.43, 140.75 (d, *J* = 17.1 Hz), 135.60 (d, *J* = 28.0 Hz), 130.02, 129.87, 129.60, 129.51, 129.42, 129.04, 128.41, 122.08 (d, *J* = 321.0 Hz), 120.74 (q, *J* = 320.2 Hz), 98.45, 79.87, 61.31, 53.20, 52.83, 41.02, 31.39, 28.49; ^{19}F NMR (375 MHz, CD_3CN) δ: –81.00, –131.46; *m/z* LRMS (ESI + APCI) found $[M + H]^+$ 610.3, $C_{30}H_{36}F_4N_3O_4S^+$ requires 610.2.

N-((1*Z*,2*E*,4*E*)-5-(Dibenzylamino)-3-methyl-1-(4-(trifluoromethyl)phenyl)penta-2,4-dien-1-ylidene)-1,1,1-trifluoromethanesulfonamide (1j)

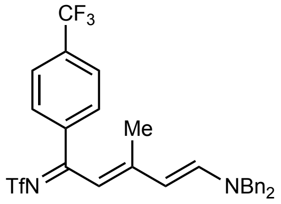

Prepared according to general procedure A using 4-methyl-2-(4-(trifluoromethyl)phenyl)pyridine (1.19 g, 5.00 mmol), EtOAc (12.5 mL, 0.4 M), Tf_2O (840 μL, 5.00 mmol), collidine (660 μL, 5.00 mmol), and dibenzylamine (1.15 mL, 6.00 mmol). Washing with H_2O (x2), sat. Na_2CO_3 (x1), brine (x1) and precipitating from hexanes (250 mL) afforded the title compound as a red solid (1.53 g, 2.70 mmol, 54% yield). mp 116 – 118 ºC; IR ν_{max}/cm^{-1} (film): 3033, 1597, 1498, 1296, 1096, 1005, 822, 697; ^1H NMR (400 MHz, CD_3CN) δ: 8.32 (d, *J* = 12.5 Hz, 1H), 7.70 (d, *J* = 8.4 Hz, 2H), 7.65 (d, *J* = 8.3 Hz, 2H), 7.44 – 7.34 (m, 6H), 7.32 – 7.22 (m, 5H), 5.64 (s, 1H), 4.73 – 4.56 (m, 4H), 2.39 (s, 3H); ^{13}C NMR (100 MHz, CD_3CN) δ: 170.10, 161.27, 147.44, 131.43 (q, *J* = 32.4 Hz), 130.63, 129.91, 129.68, 129.61, 129.49, 129.37, 129.35, 129.23, 128.55, 128.52, 125.82 (q, *J* = 3.8 Hz), 125.15 (q, *J* = 271.2 Hz), 120.85 (q, *J* = 320.6 Hz), 115.30, 108.46, 61.24, 52.41, 21.17; ^{19}F NMR (375 MHz, CD_3CN) δ: –63.16, –80.59; *m/z* LRMS (ESI + APCI) found $[M + H]^+$ 567.2, $C_{28}H_{25}F_6N_2O_2S^+$ requires 567.2.

N-((1*Z*,2*E*,4*Z*)-5-(Dibenzylamino)-4-methoxy-1-(4-methoxyphenyl)penta-2,4-dien-1-ylidene)-1,1,1-trifluoromethanesulfonamide (1k)

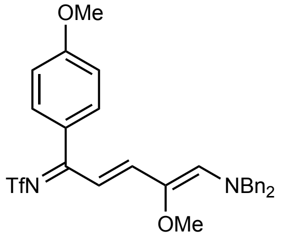

Prepared according to general procedure A using 5-methoxy-2-(4-methoxyphenyl)pyridine (1.08 g, 5.00 mmol), EtOAc (12.5 mL, 0.4 M), Tf_2O (840 μL, 5.00 mmol), collidine (660 μL, 5.00 mmol), and dibenzylamine (1.15 mL, 6.00 mmol). The reaction was diluted with EtOAc and washed with H_2O (x2),

sat. Na_2CO_3 (x1), and brine (x1). Flash chromatography (silica gel: 0% to 2% MeOH in CH_2Cl_2) afforded the title compound as a dark red oil, which was sonicated in room temperature pentanes for 20 minutes to afford the title compound as a white solid (1.14 g, 2.09 mmol, 42% yield). mp 123 – 125 ºC; IR ν_{max}/cm^{-1} (film): 3003, 1603, 1537, 1422, 1306, 1193, 1087, 874; ^1H NMR (400 MHz, DMSO) δ: 7.91 (s, 1H), 7.56 (d, *J* = 8.8 Hz, 2H), 7.46 – 7.21 (m, 11H), 7.06 (d, *J* = 8.8 Hz, 2H), 6.52 (d, *J* = 13.4 Hz, 1H), 4.89 (s, 2H), 4.56 (s, 2H), 3.84 (s, 3H), 3.53 (s, 3H); ^{13}C NMR (100 MHz, DMSO) δ: 172.58, 161.83, 153.36, 153.30, 137.19, 135.50, 135.30, 135.09, 135.01, 131.28, 130.35, 128.88, 128.20, 127.84, 127.66, 127.30, 127.14, 119.74 (q, *J* = 322.2 Hz), 113.86, 107.01, 60.54, 55.47; ^{19}F NMR (375 MHz, DMSO) δ: –78.42; *m/z* LRMS (ESI + APCI) found $[M + H]^+$ 545.1, $C_{28}H_{28}F_3N_2O_4S^+$ requires 545.2.

N-((1*E*,2*E*,4*Z*)-4-Bromo-5-(dibenzylamino)-2-methylpenta-2,4-dien-1-ylidene)-1,1,1-trifluoromethanesulfonamide (1l)

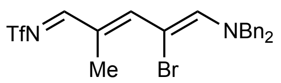

Prepared according to general procedure A using 3-methylpyridine (490 μL, 5.00 mmol), EtOAc (50.0 mL, 0.1 M), Tf_2O (840 μL, 5.00 mmol), collidine (660 μL, 5.00 mmol), and dibenzylamine (960 μL, 5.00 mmol). After warming to room temperature, *N*-bromosuccinimide (890 mg, 5.00 mmol) was added to the reaction mixture, and the reaction was stirred for 1 h at room temperature.[1] After 1 h, sat. $Na_2S_2O_3$ was added, and the reaction was diluted with EtOAc and H_2O. The layers were separated, and the organic was washed with H_2O (x1), sat. Na_2CO_3 (x1) and brine (x1). The organic extract was dried over Na_2SO_4, filtered, and concentrated *in vacuo* without heat. The resulting residue was dissolved in minimal Eåt$_2$O and added dropwise to *n*-pentane (250 mL) and stirred at –20 ºC overnight. The solid was filtered and the filtrate was concentrated *in vacuo* without heat. The resulting residue was subjected to automated flash chromatography (silica gel: 10% EtOAc/Hexanes) afforded the title compound as a brown oil, which was sonicated in *n*-pentane for 5 minutes to afford the title compound as a yellow solid (1.42 g, 2.83 mmol, 57% yield). mp 131 – 134 ºC; IR ν_{max}/cm^{-1} (film): 3033, 1495, 1398, 1226, 1191, 1147, 898, 738; ^1H NMR (400 MHz, $CDCl_3$) δ: 7.40 – 7.36 (m, 4H), 7.35 – 7.27 (m, 4H), 7.25 – 7.19 (m, 2H), 6.56 (s, 1H), 6.45 (s, 1H), 5.39 (s, 1H), 3.82 – 3.51 (m, 4H), 1.84 (s, 3H); ^{13}C NMR (100 MHz, CDCl3) δ: 138.03, 130.62, 129.21, 128.36, 127.47, 122.49, 120.25 (q, *J* = 318.6 Hz), 118.08, 114.73, 75.84, 51.45, 17.61; ^{19}F NMR (375 MHz, $CDCl_3$) δ: –74.29; *m/z* LRMS (ESI + APCI) found $[M + H]^+$ 501.0, $C_{21}H_{21}BrF_3N_2O_2S^+$ requires 501.1.

N-((1*Z*,2*E*,4*E*)-1-(4-(((*R*)-But-3-yn-2-yl)oxy)phenyl)-5-(dibenzylamino)penta-2,4-dien-1-ylidene)-1,1,1-trifluoromethanesulfonamide (1s)

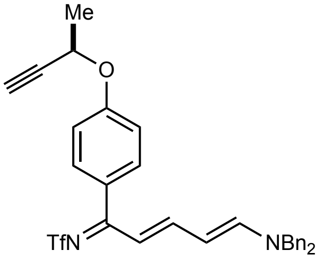

Prepared according to general procedure A using (*R*)-2-(4-(but-3-yn-2-yloxy)phenyl)pyridine (4.47 g, 20.0 mmol), EtOAc (50.0 mL, 0.4 M), Tf_2O (3.37 mL, 20.0 mmol), collidine (2.64 mL, 20.0 mmol), and dibenzylamine (4.61 mL, 24.0 mmol). Washing with H_2O (x2), sat. Na_2CO_3 (x1), brine (x1) and

precipitating from hexanes (1.00 L) afforded the title compound as a red solid (9.81 g, 17.8 mmol, 89% yield). mp 59 – 62 ºC; IR ν_{max}/cm^{-1} (film): 3283, 1615, 1556, 1470, 1433, 1163, 1093, 861; ^1H NMR (400 MHz, CD_3CN) δ:: 7.79 (d, *J* = 11.9 Hz, 1H), 7.67 – 7.53 (m, 3H), 7.37 (dq, *J* = 11.6, 5.9 Hz, 6H), 7.32 – 7.26 (m, 2H), 7.22 (d, *J* = 7.3 Hz, 2H), 7.06 (d, *J* = 8.7 Hz, 2H), 6.51 (d, *J* = 13.3 Hz, 1H), 6.05 (t, *J* = 12.2 Hz, 1H), 5.08 (qd, *J* = 6.6, 2.1 Hz, 1H), 4.59 (s, 2H), 4.55 (s, 2H), 2.82 (d, *J* = 2.0 Hz, 1H), 1.63 (d, *J* = 6.5 Hz, 3H); ^{13}C NMR (100 MHz, CD_3CN) δ:: 175.61, 162.66, 162.50, 160.70, 135.81, 135.45, 132.42, 132.22, 129.93, 129.85, 129.50, 129.29, 128.98, 128.34, 120.96 (q, *J* = 320.3 Hz), 116.07, 113.33, 105.59, 83.39, 75.60, 64.42, 61.03, 52.66, 22.36; ^{19}F NMR (375 MHz, CD_3CN) δ:: –80.19; *m/z* LRMS (ESI + APCI) found $[M + H]^+$ 553.1, $C_{30}H_{28}F_3N_2O_3S^+$ requires 553.2.

5. General Procedures for Preparation of Enantioenriched *N*-Alkylpyridinium Salts

General Procedure B (Preparation of Pyridinium Salts in One-Pot from Pyridines)

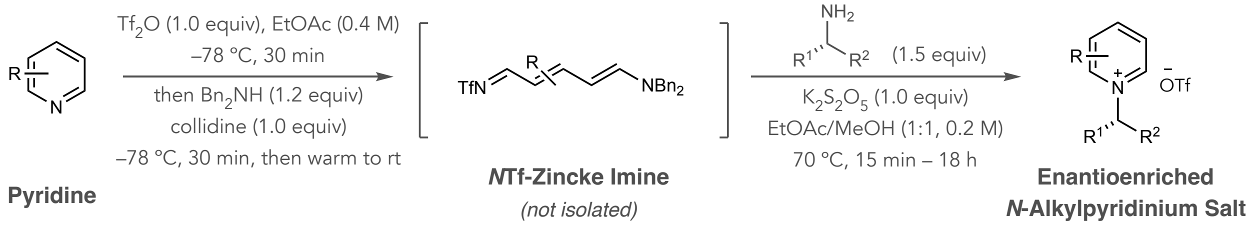

An oven dried 8 mL vial (< 1.0 mmol), 16 mL vial (≤1.60 mmol), or a round bottom flask (> 1.60 mmol) equipped with a stir bar was charged with heterocycle (1.0 equiv.) and subjected to three vacuum/nitrogen backfill cycles before being placed under a nitrogen atmosphere. EtOAc (0.2 or 0.4 M) was added, the reaction vessel was cooled to –78 ºC, and Tf_2O (1.0 equiv.) was added dropwise. The reaction was stirred for 30 minutes before collidine (2,4,6-trimethylpyridine) (1.0 equiv.) was added followed by dropwise addition of dibenzylamine (1.2 or 1.0 equiv.). The reaction was stirred for a further 30 minutes at –78 ºC before the cooling bath was removed and the reaction was allowed to warm to room temperature while stirring. The reaction was diluted with MeOH (one reaction volume, 1:1 with EtOAc) and potassium metabisulfite ($K_2S_2O_5$) (1.0 equiv.) was added followed by the enantioenriched (α-chiral)amine (1.5 equiv.). The reaction vessel was sealed and heated to 70 ºC or 50 ºC for 15 min – 18 h. The reaction was cooled to room temperature, 1,3,5-trimethylbenzene (1.0 equiv.) was added for ^1H NMR analysis of the crude reaction mixture, and the pyridinium salt was isolated according to general isolation procedure B1 or B2.

Reaction Notes:

- See reaction notes for pyridine ring-opening in General Procedure A
- Potassium metabisulfite can cause decomposition of the pyridinium salt product in select examples. If this occurred, metabisulfite was not added to the reaction.
- The reaction can be run at 50 ºC or room temperature if the pyridinium salt product decomposes at 70 ºC, but this may result in incomplete conversion of the Zincke imine intermediate. In this case, general isolation procedure B2 was used to separate the pyridinium product from the intermediate Zincke imine.
- See Supporting Information Sections 2 and 8.5 for additional details on troublesome substrates.

General Isolation Procedure B1

- **This was used as the primary procedure for isolating pyridinium salts from the one-pot pyridinium salt formation from pyridines.**

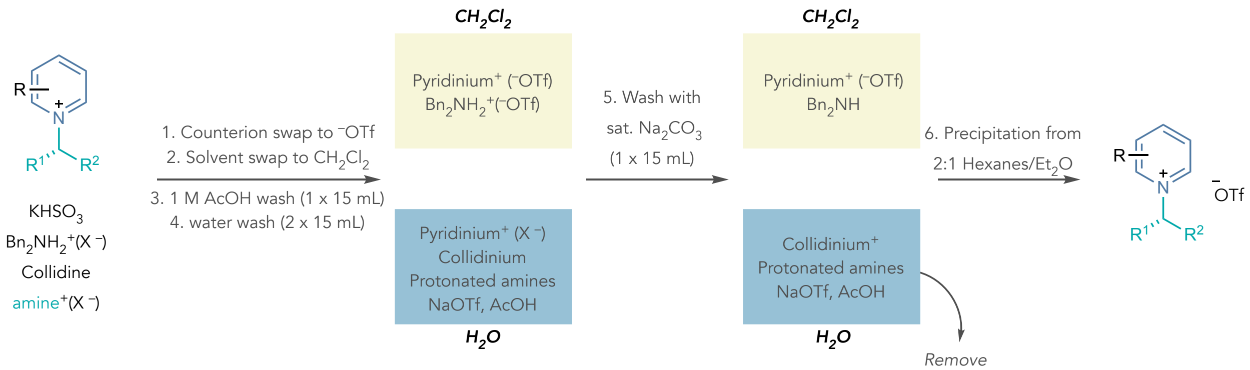

The crude mixture was diluted with MeOH (0.5 reaction volumes), NaOTf was added (6.0 equiv.), and the mixture was stirred until homogenous. Then, the mixture was concentrated *in vacuo*, dissolved in CH_2Cl_2, and transferred to a separatory funnel. The remaining NaOTf in the reaction vessel was rinsed into the separatory funnel with 1.0 M AcOH and the layers were separated. The organic layer was washed with water (x2) and sat. Na_2CO_3 (x1). The organic layer was dried over Na_2SO_4, filtered, and concentrated *in vacuo*. The residue was redissolved in minimal CH_2Cl_2, added dropwise to 2:1 hexanes/Et_2O (100 mL per 1 mmol) and stirred at room temperature overnight. The 2:1 hexanes/Et_2O were decanted off, and the free flowing solid or oily residue was collected to afford the pure pyridinium salt.

Isolation notes for general isolation procedures B1:

- Success of the isolation procedures can be dependent on the solvent amounts used. The amounts are specified for each substrate and vary depending on reaction scale.
- Emulsions formed during the liquid-liquid extraction (LLE) procedure can significantly reduce the pyridinium product yield obtained using the procedure. The separatory funnel was swirled to mix the organic and aqueous layers, rather than shaken, to avoid emulsions.
- Decomposition of the pyridinium product can occur while concentrating the crude reaction or organic layer after the LLE procedure. Concentration of the reaction and organic layer was performed *in vacuo* without heating.
- 3:1 CH_2Cl_2:IPA can be used in place of CH_2Cl_2 if the pyridinium salt displays higher aqueous solubility.
    - If the enantioenriched (α-chiral)amine was used as a salt form (i.e. hydrochloride salt), then CH_2Cl_2 should be used to avoid coprecipitation of protonated dibenzylamine or the enantioenriched (α-chiral)amine with the pyridinium product.
    - If 3:1 CH_2Cl_2:IPA is used, excess NaOTf may be retained in the organic layer. After concentrating the organic layer, the pyridinium can be separated from excess NaOTf by dissolving the residue in CH_2Cl_2 and filtering to remove the NaOTf prior to precipitating the pyridinium product.
- LCMS and ^1H NMR aliquots were used to monitor the presence of pyridinium salts in each layer
- NaOTf and KPF_6 can be used interchangeably for isolating pyridinium salts. Some pyridinium triflate salts can display higher aqueous solubility than pyridinium hexafluorophosphate salts; in these cases, KPF_6 was employed in the LLE procedure to obtain the pyridinium salt in the organic layer.

General Isolation Procedure B2:

- **This procedure was used when the pyridinium salts displayed higher aqueous solubility or were isolated with impurities using general isolation procedure B1.**

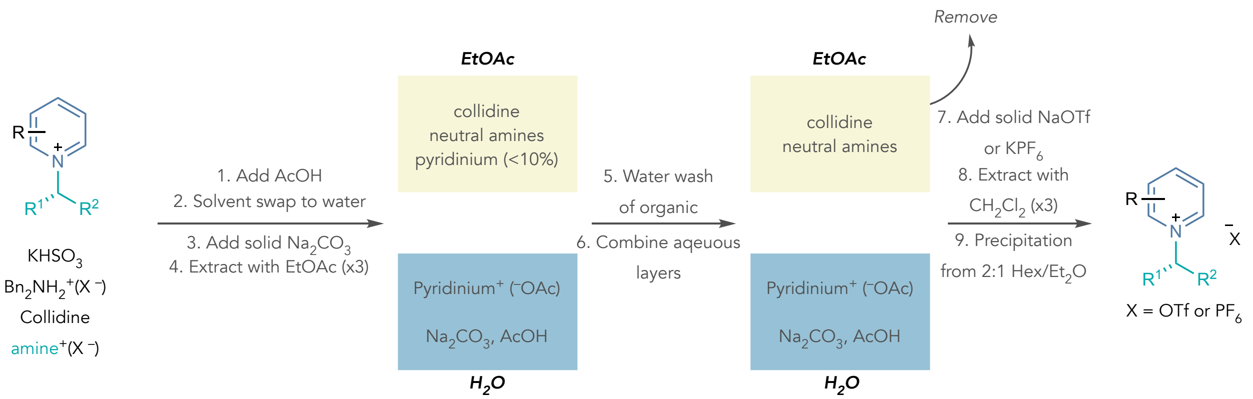

AcOH (10 equiv.) was added to the crude reaction mixture and then concentrated *in vacuo*. The resulting residue was dissolved in H_2O and transferred to a separatory funnel. Na_2CO_3 (5.0 equiv.) was added, the separatory funnel was swirled until the solution was homogenous, and the aqueous layer was extracted with EtOAc (x3). The combined organic extracts were washed with water (x2) and NaOTf or KPF_6 (9.0 equiv) was added to the combined aqueous layer. The aqueous layer was extracted with CH_2Cl_2 (x3), and the combined CH_2Cl_2 extractions were dried over Na_2SO_4, filtered, and concentrated *in vacuo*. The residue was redissolved in minimal CH_2Cl_2, added dropwise to 2:1 hexanes/Et_2O (100 mL per 1 mmol) and stirred at room temperature overnight. The 2:1 hexanes/Et_2O were decanted off, and the free flowing solid or oily residue was collected to afford the pure pyridinium salt.

Isolation notes for general isolation procedures B2:

- See isolation notes for general isolation procedure B1.

General Procedure C (Preparation of Pyridinium Salts from Isolated Zincke Imines)

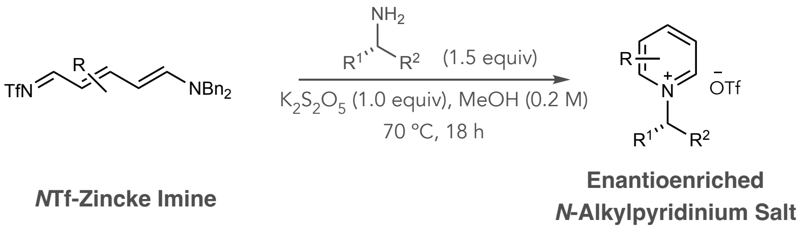

An oven dried 8 mL vial (< 1.0 mmol), 16 mL vial (≤1.60 mmol), or a round bottom flask (> 1.60 mmol) equipped with a stir bar was charged with Zincke imine (1.0 equiv.), MeOH (0.2 M), potassium metabisulfite ($K_2S_2O_5$) (1.0 equiv), and enantioenriched (α-chiral)amine (1.5 equiv.). The reaction vessel was sealed and heated to 70 ºC for 18 h. The reaction was cooled to room temperature, 1,3,5-trimethylbenzene (1.0 equiv) was added for ^{1}H NMR analysis of the crude reaction mixture, and the pyridinium salt was isolated according to general isolation procedure C1 or C2.

Reaction notes:

- Potassium metabisulfite can cause decomposition of the pyridinium salt product in select examples. If this occurred, metabisulfite was not added to the reaction.

- The reaction can be run at 50 ºC or room temperature if the pyridinium salt product decomposes at 70 ºC, but this may result in incomplete conversion of the Zincke imine intermediate. Use general isolation procedure C2 to separate the pyridinium product from the Zincke imine.
- See Supporting Information Sections 2 and 8.5 for additional details on troublesome substrates.

General Isolation Procedure C1

- **This procedure was used as the primary isolation procedure for isolating pyridinium salts prepared from isolated Zincke imines.**

The crude mixture was diluted with MeOH, NaOTf was added (6.0 equiv.) and then stirred until homogenous. The mixture was then concentrated *in vacuo*, dissolved in CH_2Cl_2, and transferred to a separatory funnel. The remaining NaOTf in the reaction vessel was rinsed into the separatory funnel with water and the layers were separated. The organic layer was washed with water (x2). The combined aqueous layers were extracted with CH_2Cl_2 (x2), and the organic layers were combined. The combined organic layers were washed with sat. Na_2CO_3 (x1), dried over Na_2SO_4, filtered, and concentrated *in vacuo*. The residue was redissolved in minimal CH_2Cl_2, added dropwise to room temperature 2:1 hexanes/Et_2O (100 mL per 1 mmol) and stirred at room temperature overnight. The 2:1 hexanes/Et_2O were decanted off, and the free flowing solid or oily residue was collected to afford the pure pyridinium salt.

General Isolation Procedure C1 Workflow:

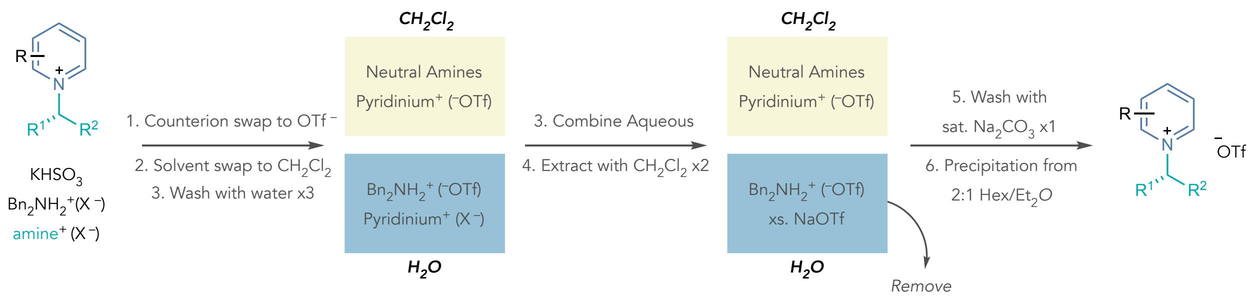

General Isolation Procedure C2

- **This procedure was used when the pyridinium salts displayed higher aqueous solubility or were isolated with impurities using general isolation procedure C1.**

The crude mixture was concentrated *in vacuo*, dissolved in water and transferred to a separatory funnel. Sodium carbonate (5.0 equiv.) was added, the separatory funnel was swirled until the solution was homogenous, and the aqueous layer was extracted with EtOAc (x3). The combined organic extracts were washed with water (x1) and sodium triflate or potassium hexafluorophosphate (9.0 equiv.) was added to the combined aqueous layers. The separatory funnel was swirled until the solution was homogenous. The aqueous layer was extracted with CH_2Cl_2 (x3). The combined CH_2Cl_2 extractions were dried over sodium sulfate, filtered and concentrated *in vacuo*. The resulting residue was redissolved in minimal CH_2Cl_2, added dropwise to room temperature 2:1 hexanes/Et_2O (100 mL per 1 mmol) and stirred at room temperature overnight. The 2:1 hexanes/Et_2O were decanted off, and the free flowing solid or oily residue was collected to afford the pure pyridinium salt.

General Isolation Procedure C2 Workflow:

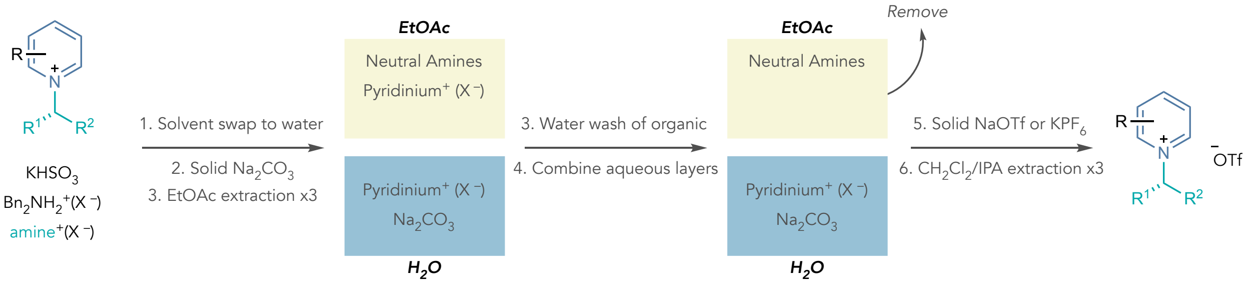

Isolation notes for general isolation procedures C1 and C2:

- See notes for general isolation procedures B1 and B2.
- When using general isolation procedure C2, the pyridinium salt may be recovered in the EtOAc extractions with the other reaction byproducts.
    - If this occurred, the EtOAc extractions were diluted with MeOH, NaOTf (2 equiv.) was added, and the mixture was concentrated *in vacuo*. The resulting residue was dissolved in CH_2Cl_2 and filtered to remove the excess NaOTf. The filtrate was then added dropwise to room temperature 2:1 hexanes/Et_2O and stirred at room temperature overnight. The resulting solid or oily residue was collected to afford the pure pyridinium product.

6. Characterization Data for Enantioenriched *N*-Alkylpyridinium Salts

6.1. Enantioenriched *N*-Alkylpyridinium Salts from Optimization Studies

(*S*)-1-(1-Cyclohexylethyl)-2-phenylpyridin-1-ium trifluoromethanesulfonate (3a)

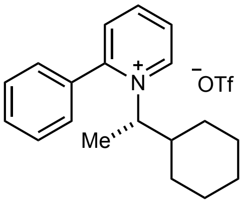

Prepared according to general procedure B using 2-phenylpyridine (71.0 µL, 0.500 mmol), EtOAc (1.25 mL, 0.4 M), Tf_2O (84.0 µL, 0.500 mmol), collidine (66.0 µL, 0.500 mmol), dibenzylamine (120 µL, 0.600 mmol), MeOH (1.25 mL, one reaction volume), potassium metabisulfite (111 mg, 0.500 mmol), (*S*)-1-cyclohexylethan-1-amine (**2a**) (110 µL, 0.750 mmol), and heated to 70 ºC for 18 h. Isolated according to general isolation procedure B1 using NaOTf (516 mg, 3.00 mmol), CH_2Cl_2 (30 mL), 1.0 M AcOH wash (1 x 15 mL), H_2O washes (2 x 15 mL), sat. Na_2CO_3 wash (1 x 15 mL), and 2:1 hexanes/Et_2O (50 mL) to afford the title compound as a light-yellow solid (168 mg, 0.404 mmol, 81% yield). mp 135 – 138 ºC; IR ν_{max}/cm^{-1} (film): 3095, 2927, 2857, 1622, 1486, 1256, 1150, 1030, 637; ^{1}H NMR (400 MHz, CD_3OD) δ: 9.17 (dd, *J* = 6.4, 1.4 Hz, 1H), 8.61 (td, *J* = 7.8, 1.4 Hz, 1H), 8.20 (ddd, *J* = 7.8, 6.4, 1.6 Hz, 1H), 8.04 (dd, *J* = 8.0, 1.6 Hz, 1H), 7.77 – 7.64 (m, 3H), 7.58 (dd, *J* = 7.9, 1.8 Hz, 2H), 4.54 (dq, *J* = 9.9, 6.8 Hz, 1H), 2.04 – 1.85 (m, 2H), 1.78 – 1.70 (m, 4H), 1.68 – 1.56 (m, 2H), 1.29 (qt, *J* = 12.8, 3.6 Hz, 1H), 1.19 – 0.92 (m, 3H), 0.78 (tdd, *J* = 12.7, 11.0, 3.6 Hz, 1H), 0.63 (qd, *J* = 12.2, 3.5 Hz, 1H); ^{13}C NMR (100 MHz, CD_3OD) δ: 158.00, 146.50, 143.40, 133.39, 132.59, 131.79, 130.08, 129.11, 121.81 (q, *J* = 318.6 Hz), 70.46, 45.52,

30.76, 30.55, 26.66, 26.44, 19.26; ^{19}F NMR (375 MHz, CD_3OD) δ: –80.06; *m/z* LRMS (ESI + APCI) found $[M – OTf]^+$ 266.2, $C_{19}H_{24}N^+$ requires 266.2.

(*S*)-1-(2-Hydroxy-1-phenylethyl)-2-phenylpyridin-1-ium trifluoromethanesulfonate (3w)

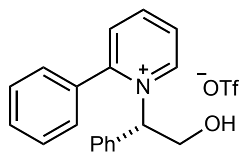

Prepared according to general procedure B using 2-phenylpyridine (71.0 μL, 0.500 mmol), EtOAc (1.25 mL, 0.4 M), Tf_2O (84.0 μL, 0.500 mmol), collidine (66.0 μL, 0.500 mmol), dibenzylamine (120 μL, 0.600 mmol), MeOH (1.25 mL, one reaction volume), potassium metabisulfite (111 mg, 0.500 mmol), (*S*)-2-amino-2-phenylethan-1-ol (**2b**) (103 mg, 0.750 mmol), and heated to 70 ºC for 18 h. Isolated according to general isolation procedure B1 using NaOTf (516 mg, 3.00 mmol), 3:1 CH_2Cl_2:IPA (30 mL), 1.0 M AcOH wash (1 x 15 mL), H_2O wash (1 x 15 mL), sat. Na_2CO_3 wash (1 x 15 mL), and 2:1 hexanes/Et_2O (50 mL) to afford the title compound as an orange solid (144 mg, 0.338 mmol, 81% yield). The O–H proton was exchanged with deuterium from CD_3OD during characterization. mp 83 – 85 ºC; IR ν_{max}/cm^{-1} (film): 3389, 3062, 1622, 1484, 1248, 1151, 1027, 635; ^{1}H NMR (400 MHz, CD_3OD) δ: 9.24 (d, *J* = 6.4 Hz, 1H), 8.66 (td, *J* = 7.8, 1.4 Hz, 1H), 8.18 (t, *J* = 6.9 Hz, 1H), 8.10 (dd, *J* = 8.0, 1.6 Hz, 1H), 7.82 – 7.48 (m, 4H), 7.49 – 7.24 (m, 4H), 7.20 (dd, *J* = 6.7, 2.9 Hz, 2H), 6.14 (dd, *J* = 8.8, 4.4 Hz, 1H), 4.48 (dd, *J* = 12.5, 8.7 Hz, 1H), 4.29 (dd, *J* = 12.5, 4.4 Hz, 1H); ^{13}C NMR (100 MHz, CD_3CN) δ: 157.92, 146.78, 144.50, 135.31, 132.67, 132.18, 131.87, 130.49, 130.21, 129.41, 128.61, 128.40, 128.18, 122.09 (q, *J* = 320.9 Hz), 71.72, 63.27; ^{19}F NMR (375 MHz, CD_3OD) δ: –77.94; *m/z* LRMS (ESI + APCI) found $[M – OTf]^+$ 276.2, $C_{19}H_{18}NO^+$ requires 276.1.

1-((3a*S*,4*R*,6*S*,6a*R*)-6-Hydroxy-2,2-dimethyltetrahydro-4*H*-cyclopenta[*d*][1,3]dioxol-4-yl)-2-phenylpyridin-1-ium trifluoromethanesulfonate (3z)

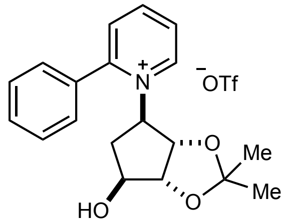

Prepared according to general procedure B using 2-phenylpyridine (71.0 μL, 0.500 mmol), EtOAc (1.25 mL, 0.4 M), Tf_2O (84.0 μL, 0.500 mmol), collidine (66.0 μL, 0.500 mmol), dibenzylamine (120 μL, 0.600 mmol), MeOH (1.25 mL, one reaction volume), potassium metabisulfite (111 mg, 0.500 mmol), (3a*R*,4*S*,6*R*,6a*S*)-6-amino-2,2-dimethyltetrahydro-4*H*-cyclopenta[*d*][1,3]dioxol-4-ol (**2d**) (130 mg, 0.750 mmol), and heated to 70 ºC for 18 h. Isolated according to general isolation procedure B1 using NaOTf (516 mg, 3.00 mmol), 3:1 CH_2Cl_2:IPA (30 mL), 1.0 M AcOH wash (1 x 15 mL), H_2O washes (2 x 15 mL), sat. Na_2CO_3 wash (1 x 15 mL), and 2:1 hexanes/Et_2O (50 mL) to afford the title compound as a yellow solid (121 mg, 0.262 mmol, 52% yield). The O–H proton was exchanged with deuterium from CD_3OD during characterization. mp 94 – 97 ºC; IR ν_{max}/cm^{-1} (film): 3404, 2989, 2937, 1625, 1488, 1251, 1152, 1028, 636; ^{1}H NMR (400 MHz, CD_3OD) δ: 9.58 (dd, *J* = 6.6, 1.4 Hz, 1H), 8.58 (td, *J* = 7.8, 1.4 Hz, 1H), 8.18 (ddd, *J* = 7.9, 6.4, 1.6 Hz, 1H), 8.04 (dd, *J* = 8.1, 1.6 Hz, 1H), 7.78 – 7.58 (m, 5H), 5.15 – 5.05 (m, 2H), 4.67 (dt, *J* = 5.4, 1.5 Hz, 1H), 4.33 (ddd, *J* = 5.4, 2.5, 1.4 Hz, 1H), 2.83 (ddd, *J* = 15.1, 9.3, 5.4 Hz, 1H), 2.25 – 2.14

(m, 1H), 1.28 (s, 3H), 1.20 (s, 3H); ^{13}C NMR (100 MHz, CD_3OD) δ: 158.09, 146.45, 145.44, 133.30, 132.55, 131.46, 130.37, 130.26, 128.87, 121.80 (q, J = 318.4 Hz), 113.86, 88.94, 88.40, 75.48, 75.44, 41.22, 27.13, 25.06; ^{19}F NMR (375 MHz, CD_3OD) δ: –80.10; *m/z* LRMS (ESI + APCI) found $[M – OTf]^+$ 312.2, $C_{19}H_{22}NO_3^+$ requires 312.2.

(*R*)-2-Phenyl-1-(1,1,1-trifluoropropan-2-yl)pyridin-1-ium trifluoromethanesulfonate (3u)

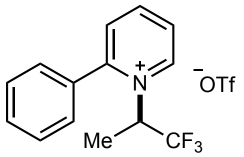

Prepared according to general procedure C using *N*-((1*Z*,2*E*,4*E*)-5-(dibenzylamino)-1-phenylpenta-2,4-dien-1-ylidene)-1,1,1-trifluoromethanesulfonamide (**1a**) (242 mg, 0.500 mmol), MeOH (2.50 mL, 0.2 M), potassium metabisulfite (111 mg, 0.500 mmol), (*R*)-1,1,1-trifluoropropan-2-amine hydrochloride (**2d**) (112 mg, 0.750 mmol), and heated to 70 ºC for 18 h. Isolated according to general isolation procedure C2 using H_2O (15 mL), Na_2CO_3 (265 mg, 2.50 mmol), EtOAc for first extractions (3 x 10 mL), H_2O for back extraction (2 x 10 mL), NaOTf (774 mg, 4.50 mmol), CH_2Cl_2 for extraction of pyridinium salt (3 x 20 mL), and 2:1 hexanes/Et_2O (50 mL) to afford the title compound as a clear yellow oil (111 mg, 0.277 mmol, 55% yield). IR ν_{max}/cm^{-1} (film): 3096, 1624, 1509, 1486, 1256, 1154, 1052, 1029, 636; ^{1}H NMR (400 MHz, CD_3OD) δ: 9.38 (d, J = 6.6 Hz, 1H), 8.81 (td, J = 7.8, 1.4 Hz, 1H), 8.32 (ddd, J = 8.0, 6.5, 1.6 Hz, 1H), 8.20 (dd, J = 8.0, 1.6 Hz, 1H), 7.81 – 7.67 (m, 3H), 7.66 – 7.56 (m, 2H), 5.74 (hept, J = 6.8 Hz, 1H), 2.02 (d, J = 7.0 Hz, 3H); ^{13}C NMR (100 MHz, CD_3OD) δ: 159.27, 149.32, 144.59, 133.15, 132.84, 132.28, 131.00, 130.09, 129.41, 124.56 (q, J = 282.2 Hz), 121.80 (q, J = 318.2 Hz), 63.17 (q, J = 32.6 Hz), 14.15 (q, J = 1.8 Hz); ^{19}F NMR (375 MHz, CD_3OD) δ: -75.24 (d, J = 6.5 Hz), –80.11; *m/z* LRMS (ESI + APCI) found $[M – OTf]^+$ 252.1, $C_{14}H_{13}F_3N^+$ requires 252.1.

6.2. Enantioenriched *N*-Alkylpyridinium Salts Prepared in One-Pot from Pyridines

(*S*)-1-(1-(2-Fluorophenyl)ethyl)-2-phenylpyridin-1-ium trifluoromethanesulfonate (3b)

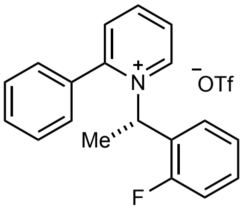

Prepared according to general procedure B using 2-phenylpyridine (71.0 μL, 0.500 mmol), EtOAc (1.25 mL, 0.4 M), Tf_2O (84.0 μL, 0.500 mmol), collidine (66.0 μL, 0.500 mmol), dibenzylamine (120 μL, 0.600 mmol), MeOH (1.25 mL, one reaction volume), potassium metabisulfite (111 mg, 0.500 mmol), (*S*)-1-(2-fluorophenyl)ethan-1-amine (**2ao**) (96.0 μL, 0.750 mmol), and heated to 70 ºC for 18 h. 1,3,5-Trimethylbenzene (70.0 μL, 0.500 mmol, 1.0 equiv.) was added as the internal standard for ^{1}H NMR analysis (82% yield). Isolated according to general isolation procedure B1 using NaOTf (516 mg, 3.00 mmol), CH_2Cl_2 (30 mL), 1.0 M AcOH wash (1 x 15 mL), H_2O washes (2 x 15 mL), sat. Na_2CO_3 wash (1 x 15 mL), and 2:1 hexanes/Et_2O (50 mL) to afford the title compound as a light-yellow oil (157 mg, 0.367 mmol, 73% yield). IR ν_{max}/cm^{-1} (film): 3069, 1618, 1508, 1484, 1223, 1028, 759, 701, 634; ^{1}H NMR (400 MHz, CD_3OD) δ: 8.94 (dd, J = 6.5, 1.4 Hz, 1H), 8.62 (td, J = 7.8, 1.4 Hz, 1H), 8.14 – 8.01 (m, 2H), 7.78 –

7.55 (m, 6H), 7.49 (tdd, *J* = 7.6, 5.5, 1.7 Hz, 1H), 7.33 (td, *J* = 7.7, 1.2 Hz, 1H), 7.10 (ddd, *J* = 11.0, 8.2, 1.2 Hz, 1H), 6.30 (q, *J* = 7.0 Hz, 1H), 2.08 (d, *J* = 7.0 Hz, 3H); ^{13}C NMR (100 MHz, CD_3OD) δ: 161.96 (d, *J* = 248.2 Hz), 157.81, 147.21, 144.08, 133.43, 133.35, 133.08, 132.38 (d, *J* = 59.1 Hz), 130.55, 129.93, 129.72 (d, *J* = 2.8 Hz), 128.94, 126.45 (d, *J* = 3.6 Hz), 125.90 (d, *J* = 13.4 Hz), 121.80 (q, *J* = 318.6 Hz), 117.05 (d, *J* = 21.2 Hz), 62.63 (d, *J* = 2.5 Hz), 20.26; ^{19}F NMR (375 MHz, CD_3OD) δ: –80.08, –116.77 (ddd, *J* = 13.0, 7.6, 5.5 Hz); *m/z* LRMS (ESI + APCI) found $[M – OTf]^+$ 278.0, $C_{19}H_{17}FN^+$ requires 278.1.

1-((1*S*,2*S*)-1-Hydroxy-1-phenylpropan-2-yl)-2-isopropylpyridin-1-ium trifluoromethanesulfonate (3c)

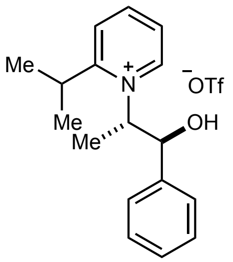

Prepared according to general procedure B using 2-isopropylpyridine (42.0 μL, 0.500 mmol), EtOAc (2.50 mL, 0.2 M), Tf_2O (84.0 μL, 0.500 mmol), dibenzylamine (120 μL, 0.600 mmol), collidine (66.0 μL, 0.500 mmol), AcOH (290 μL, 5.00 mmol), potassium metabisulfite (111 mg, 0.500 mmol), (1*S*,2*S*)-2-amino-1-phenylpropan-1-ol (113 mg, 0.750 mmol), and heated to 70 ºC for 18 h. 1,3,5-Trimethylbenzene (70.0 μL, 0.500 mmol, 1.0 equiv.) was added as the internal standard for ^{1}H NMR analysis (58% yield). Isolated according to general isolation procedure B1 using NaOTf (516 mg, 3.00 mmol), CH_2Cl_2 (30 mL), 1.0 M AcOH wash (1 x 15 mL), H_2O washes (2 x 15 mL), sat. Na_2CO_3 wash (1 x 15 mL), and 2:1 hexanes/Et_2O (50 mL) to afford the title compound as a clear yellow oil (106 mg, 0.261 mmol, 52% yield). The O–H proton was exchanged with deuterium from CD_3OD during characterization. IR ν_{max}/cm^{-1} (film): 3359, 2107, 2990, 1627, 1501, 1450, 1291, 1245, 1149, 1026, 635; ^{1}H NMR (400 MHz, CD_3OD) δ: 8.95 (dd, *J* = 6.6, 1.5 Hz, 1H), 8.46 (td, *J* = 7.9, 1.5 Hz, 1H), 8.02 (dd, *J* = 8.2, 1.6 Hz, 1H), 7.91 (ddd, *J* = 7.8, 6.5, 1.6 Hz, 1H), 7.39 – 7.21 (m, 5H), 5.43 (dt, *J* = 12.1, 6.6 Hz, 1H), 5.04 (d, *J* = 5.4 Hz, 1H), 3.67 (hept, *J* = 6.8 Hz, 1H), 1.76 (d, *J* = 6.7 Hz, 3H), 1.34 (d, *J* = 6.8 Hz, 3H), 1.31 (d, *J* = 6.7 Hz, 3H); ^{13}C NMR (100 MHz, CD_3OD) δ: 165.74, 146.87, 144.50, 141.15, 129.76, 129.65, 127.36, 127.16, 126.36, 121.81 (q, *J* = 318.2 Hz), 76.44, 66.56, 31.62, 23.27, 21.54, 16.54; ^{19}F NMR (375 MHz, CD_3OD) δ: –80.08; *m/z* LRMS (ESI + APCI) found $[M – OTf]^+$ 256.2, $C_{17}H_{22}NO^+$ requires 256.2.

1-((1*R*,2*S*)-1-(Methoxycarbonyl)-2-vinylcyclopropyl)-3-((*S*)-1-methylpyrrolidin-2-yl)pyridin-1-ium trifluoromethanesulfonate (3d)

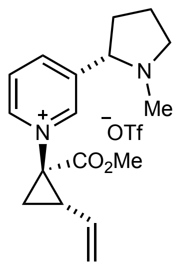

Prepared according to general procedure B using (*S*)-3-(1-methylpyrrolidin-2-yl)pyridine (80.0 μL, 0.500 mmol), EtOAc (1.25 mL, 0.4 M), Tf_2O (84.0 μL, 0.500 mmol), collidine (66.0 μL, 0.500 mmol),

dibenzylamine (96.0 μL, 0.600 mmol), MeOH (1.25 mL, one reaction volume), methyl (1*R*,2*S*)-1-amino-2-vinylcyclopropane-1-carboxylate 4-methylbenzenesulfonate (235 mg, 0.750 mmol), and heated to 50 ºC for 18 h. 1,3,5-Trimethylbenzene (70.0 μL, 0.500 mmol, 1.0 equiv.) was added as the internal standard for ^{1}H NMR analysis (70% yield). Isolated according to general isolation procedure B2 using AcOH (290 μL, 5.00 mmol), H_2O (15 mL), Na_2CO_3 (265 mg, 2.50 mmol), EtOAc for first extractions (3 x 10 mL), H_2O for back extraction (2 x 10 mL), NaOTf (774 mg, 4.5 mmol), CH_2Cl_2 for extraction of pyridinium salt (3 x 20 mL) and 2:1 hexanes/Et_2O (50 mL) to afford the title compound as a red oil (135 mg, 0.309 mmol, 62% yield). IR ν_{max}/cm^{-1} (film): 2958, 2791, 1737, 1630, 1491, 1256, 1223, 1150, 635; ^{1}H NMR (400 MHz, CD_3CN) δ: 8.78 (s, 1H), 8.72 (dd, *J* = 6.2, 1.4 Hz, 1H), 8.57 (dd, *J* = 8.1, 1.6 Hz, 1H), 8.00 (dd, *J* = 8.0, 6.1 Hz, 1H), 5.94 (ddd, *J* = 17.2, 10.3, 8.1 Hz, 1H), 5.56 (dd, *J* = 17.3, 0.8 Hz, 1H), 5.38 (dd, *J* = 10.4, 0.7 Hz, 1H), 3.68 (s, 3H), 3.48 (t, *J* = 8.1 Hz, 1H), 3.31 – 3.20 (m, 1H), 2.95 – 2.84 (m, 1H), 2.47 – 2.23 (m, 4H), 2.21 (s, 3H), 1.92 – 1.80 (m, 2H), 1.73 – 1.62 (m, 1H); ^{13}C NMR (100 MHz, CD_3OD) δ: 167.80, 148.01, 147.29, 147.25, 147.20, 132.04, 129.15, 121.80 (q, *J* = 318.6 Hz), 121.34, 68.46, 59.44, 57.86, 54.21, 40.57, 36.18, 35.06, 23.96, 22.96; ^{19}F NMR (377 MHz, CD_3OD) δ: –80.07; *m/z* LRMS (ESI + APCI) found $[M – OTf]^+$ 287.3, $C_{17}H_{23}N_2O_2^+$ requires 287.2.

3-(Methoxycarbonyl)-1-((2*S*,3*R*,4*R*,5*S*,6*R*)-2,4,5-triacetoxy-6-(acetoxymethyl)tetrahydro-2*H*-pyran-3-yl)pyridin-1-ium trifluoromethanesulfonate (3e)

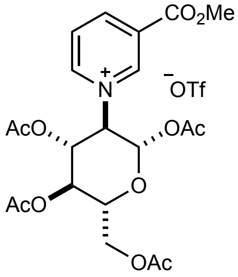

Prepared according to general procedure B using methyl nicotinate (68.6 mL, 0.500 mmol), EtOAc (1.25 mL, 0.4 M), Tf_2O (84.0 μL, 0.500 mmol), collidine (66.0 μL, 0.500 mmol), dibenzylamine (96.0 μL, 0.600 mmol), MeOH (1.25 mL, one reaction volume), (2*S*,3*R*,4*R*,5*S*,6*R*)-6-(acetoxymethyl)-3-aminotetrahydro-2*H*-pyran-2,4,5-triyl triacetate hydrochloride (**2ae**) (288 mg, 0.750 mmol), and heated to 70 ºC for 15 min. 1,3,5-Trimethylbenzene (70.0 μL, 0.500 mmol, 1.0 equiv.) was added as the internal standard for ^{1}H NMR analysis (73% yield). Isolated according to general isolation procedure B1 using NaOTf (516 mg, 3.00 mmol), CH_2Cl_2 (30 mL), 1.0 M AcOH wash (1 x 15 mL), H_2O washes (2 x 15 mL), sat. Na_2CO_3 wash (1 x 15 mL), and 2:1 hexanes/Et_2O (50 mL) to afford the title compound as a beige solid (106 mg, 0.172 mmol, 41% yield). The pyridinium salt product was unstable in the crude reaction mixture and partially decomposed during the isolation procedure. mp 114 – 117 ºC; IR ν_{max}/cm^{-1} (film): 3083, 1739, 1638, 1369, 1203, 1028, 916, 752, 637; ^{1}H NMR (400 MHz, CD_3OD) δ: 9.91 (s, 1H), 9.46 (d, *J* = 6.2 Hz, 1H), 9.19 (dt, *J* = 8.1, 1.4 Hz, 1H), 8.37 (dd, *J* = 8.1, 6.2 Hz, 1H), 6.49 (d, *J* = 8.6 Hz, 1H), 6.10 (dd, *J* = 10.7, 8.8 Hz, 1H), 5.43 (dd, *J* = 10.7, 8.5 Hz, 1H), 5.34 (dd, *J* = 10.0, 8.8 Hz, 1H), 4.44 – 4.28 (m, 2H), 4.21 (dd, *J* = 12.3, 2.1 Hz, 1H), 4.09 (s, 3H), 2.08 (s, 3H), 2.07 (s, 3H), 1.95 (s, 3H), 1.86 (s, 3H); ^{13}C NMR (100 MHz, CD_3OD) δ: 172.11, 171.49, 170.97, 169.69, 162.77, 149.18, 133.32, 130.43, 121.80 (q, *J* = 318.2 Hz), 95.41, 92.30, 76.54, 74.08, 73.69, 72.76, 69.17, 62.52, 20.54, 20.48, 20.43, 20.20, 19.92; ^{19}F NMR (375 MHz, CD_3OD) δ: –80.12; *m/z* LRMS (ESI + APCI) found $[M – OTf]^+$ 468.2, $C_{21}H_{26}NO_{11}^+$ requires 468.2.

1-((3*S*,4*R*)-1-(Tert-butoxycarbonyl)-3-fluoropiperidin-4-yl)-2-(isopropoxycarbonyl)-3-methylpyridin-1-ium trifluoromethanesulfonate (3f)

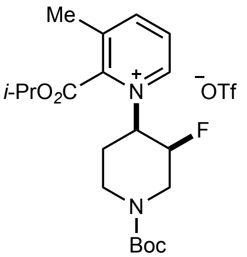

Prepared according to general procedure B using isopropyl 3-methylpicolinate (89.6 mg, 0.500 mmol), EtOAc (1.25 mL, 0.4 M), Tf_2O (84.0 μL, 0.500 mmol), collidine (66.0 μL, 0.500 mmol), dibenzylamine (120 μL, 0.600 mmol), MeOH (1.25 mL, one reaction volume), potassium metabisulfite (111 mg, 0.500 mmol), *tert*-butyl (3*S*,4*R*)-4-amino-3-fluoropiperidine-1-carboxylate (164 mg, 0.750 mmol), and heated to 50 ºC for 18 h. 1,3,5-Trimethylbenzene (70.0 μL, 0.500 mmol, 1.0 equiv.) was added as the internal standard for ^{1}H NMR analysis (71% yield). Isolated according to general isolation procedure B1 using NaOTf (516 mg, 3.00 mmol), CH_2Cl_2 (30 mL), 1.0 M AcOH wash (1 x 15 mL), H_2O washes (2 x 15 mL), sat. Na_2CO_3 wash (1 x 15 mL), and 2:1 hexanes/Et_2O (50 mL) to afford the title compound as an orange solid (174 mg, 0.328 mmol, 66% yield). mp 82 – 84 ºC; IR ν_{max}/cm^{-1} (film): 2984, 1736, 1691, 1424, 1256, 1153, 1029, 1003, 857, 636; ^{1}H NMR (400 MHz, CD_3CN) δ: 8.74 (d, *J* = 6.3 Hz, 1H), 8.53 (d, *J* = 8.0 Hz, 1H), 8.05 (dd, *J* = 8.1, 6.4 Hz, 1H), 5.52 (hept, *J* = 6.3 Hz, 1H), 5.06 (d, *J* = 49.3 Hz, 1H), 4.77 (dddd, *J* = 27.7, 12.5, 4.3, 1.8 Hz, 1H), 4.60 – 4.45 (m, 1H), 4.40 – 4.28 (m, 1H), 3.31 – 2.93 (m, 2H), 2.60 – 2.44 (m, 4H), 2.12 (dd, *J* = 12.3, 2.9 Hz, 1H), 1.53 – 1.41 (m, 15H); ^{13}C NMR (100 MHz, CD_3CN) δ: 160.63, 155.55, 150.66, 146.03, 142.84, 142.77, 139.18, 129.30, 122.12 (q, *J* = 321.0 Hz), 87.73 (d, *J* = 183.0 Hz), 81.02, 75.60, 69.22 (d, *J* = 18.9 Hz), 56.04, 34.80, 28.39, 26.73, 23.00, 21.64, 18.78, 14.28; ^{19}F NMR (375 MHz, CD_3CN) δ: –79.31, –203.20 (d, *J* = 35.3 Hz); *m/z* LRMS (ESI + APCI) found [M – OTf]$^+$ 381.3, $C_{20}H_{30}FN_2O_4^+$ requires 381.2.

3-Bromo-1-((1*R*,2*S*)-2-fluorocyclopropyl)-4-(methoxycarbonyl)pyridin-1-ium hexafluorophosphate(V) (3g)

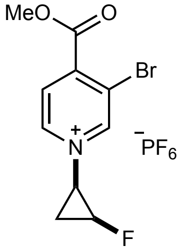

Prepared according to general procedure B using methyl 3-bromoisonicotinate (68.0 μL, 0.500 mmol), EtOAc (1.25 mL, 0.4 M), Tf_2O (84.0 μL, 0.500 mmol), collidine (66.0 μL, 0.500 mmol), dibenzylamine (96.0 μL, 0.600 mmol), MeOH (1.25 mL, one reaction volume), (1*R*,2*S*)-2-fluorocyclopropan-1-amine 4-methylbenzenesulfonate (186 mg, 0.750 mmol), and heated to 70 ºC for 15 min. 1,3,5-Trimethylbenzene (70.0 μL, 0.500 mmol, 1.0 equiv.) was added as the internal standard for ^{1}H NMR analysis (77% yield). Isolated according to general isolation procedure B2 using AcOH (290 μL, 5.00 mmol), H_2O (15 mL), Na_2CO_3 (265 mg, 2.50 mmol), EtOAc for first extractions (3 x 10 mL), H_2O for back extraction (2 x 10 mL), KPF_6 (828 mg, 4.5 mmol), 3:1 CH_2Cl_2:IPA for extraction of pyridinium salt (3 x 20 mL) and 2:1 hexanes/Et_2O (50 mL) to afford the title compound as a thick brown oil (147 mg, 0.250 mmol, 70% yield). IR ν_{max}/cm^{-1} (film): 3129, 1740, 1432, 1278, 1134, 822, 778, 553; ^{1}H NMR (400 MHz, CD_3OD) δ: 9.70 (s, 1H), 9.25 (d, *J* = 6.3 Hz, 1H), 8.42 (d, *J* = 6.3 Hz, 1H), 5.19 (dtd, *J* = 63.5, 5.6, 3.2 Hz, 1H), 4.50 (dt, *J* =

8.8, 5.5 Hz, 1H), 4.07 (s, 3H), 2.22 (dddd, *J* = 26.1, 9.5, 5.8, 3.2 Hz, 1H), 1.89 (dddd, *J* = 17.5, 9.9, 9.0, 5.9 Hz, 1H); ^{13}C NMR (100 MHz, CD_3OD) δ: 163.73, 152.50, 149.68, 147.76, 129.18, 122.34, 70.39 (d, *J* = 226.0 Hz), 54.60, 45.28 (d, *J* = 8.8 Hz), 14.20 (d, *J* = 11.0 Hz); ^{19}F NMR (375 MHz, CD_3OD) δ: –74.70 (d, *J* = 707.6 Hz), –219.74 (ddd, *J* = 64.5, 26.7, 20.2 Hz); ^{31}P NMR (162 MHz, CD_3OD) δ: –144.63 (hept, *J* = 707.7 Hz); *m/z* LRMS (ESI + APCI) found $[M – OTf]^+$ 273.9, $C_{10}H_{10}BrFNO_2^+$ requires 274.0.

6.3. Enantioenriched *N*-Alkylpyridinium Salts from Mechanism Studies

1-Isopropyl-2-phenylpyridin-1-ium trifluoromethanesulfonate (3h)

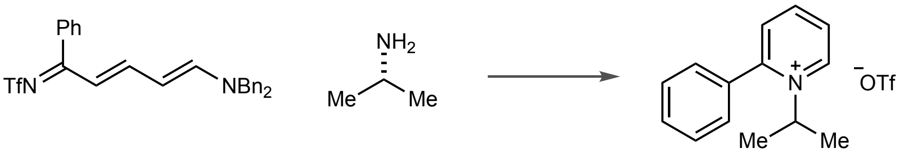

1a 2aw

Prepared according to general procedure B using 2-phenylpyridine (2.85 mL, 20.0 mmol), EtOAc (50.0 mL, 0.4 M), Tf_2O (3.37 mL, 20.0 mmol), collidine (2.64 mL, 20.0 mmol), dibenzylamine (4.61 mL, 24.0 mmol), MeOH (50.0 mL, one reaction volume), potassium metabisulfite (4.45 g, 20.0 mmol), propan-2-amine (**2aw**) (2.58 mL, 30.0 mmol), and heated to 70 ºC for 18 h. Isolated according to general isolation procedure B1 using NaOTf (17.2 g, 100 mmol), CH_2Cl_2 (100 mL), 1.0 M AcOH wash (1 x 50 mL), H_2O washes (2 x 50 mL), sat. Na_2CO_3 wash (1 x 50 mL), and 2:1 hexanes/Et_2O (1.00 L) to afford the title compound as an off-white solid (4.35 g, 12.5 mmol, 63% yield). mp 68 – 72 ºC; IR ν_{max}/cm^{-1} (film): 3094, 2978, 1622, 1508, 1485, 1257, 1143, 1057, 1026, 746, 633; ^{1}H NMR (400 MHz, CD_3OD) δ: 9.22 (dd, *J* = 6.1, 1.1 Hz, 1H), 8.59 (td, *J* = 7.8, 1.4 Hz, 1H), 8.18 (ddd, *J* = 7.8, 6.4, 1.6 Hz, 1H), 8.00 (dd, *J* = 7.9, 1.6 Hz, 1H), 7.73 – 7.65 (m, 3H), 7.64 – 7.60 (m, 2H), 5.03 (hept, *J* = 6.7 Hz, 1H), 1.63 (d, *J* = 6.7 Hz, 6H); ^{13}C NMR (100 MHz, CD_3OD) δ: 157.23, 146.38, 143.00, 133.33, 132.50, 131.84, 130.63, 129.76, 128.97, 121.80 (q, *J* = 318.6 Hz), 61.49, 23.02; ^{19}F NMR (375 MHz, CD_3OD) δ: -80.09; *m/z* LRMS (ESI + APCI) found $[M – OTf]^+$ 198.2, $C_{14}H_{16}N^+$ requires 198.1.

(*S*)-1-(1-Cyclohexylethyl)-3-phenylpyridin-1-ium trifluoromethanesulfonate (3i)

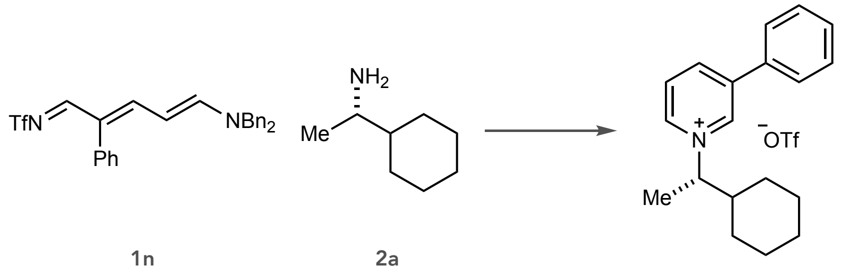

1n 2a

Prepared according to general procedure C using *N*-((1*E*,2*E*,4*E*)-5-(dibenzylamino)-2-phenylpenta-2,4-dien-1-ylidene)-1,1,1-trifluoromethanesulfonamide (**1m**) (242 mg, 0.500 mmol), MeOH (2.50 mL, 0.2 M), (*S*)-1-cyclohexylethan-1-amine (**2a**) (110 µL, 0.750 mmol), and heated to 70 ºC for 18 h. Isolated according to general isolation procedure C1 using NaOTf (516 mg, 9.00 mmol), CH_2Cl_2 (30 mL), H_2O washes (2 x 15 mL), sat. Na_2CO_3 (15 mL), and 2:1 hexanes/Et_2O (50 mL) to afford the title compound as a yellow oil (197 mg, 0.474 mmol, 95% yield). IR ν_{max}/cm^{-1} (film): 3067, 2930, 2856, 1580, 1488, 1251, 1027, 759, 697, 635; ^{1}H NMR (400 MHz, CD_3OD) δ: 9.28 (t, *J* = 1.7 Hz, 1H), 8.98 (dt, *J* = 6.1, 1.3 Hz, 1H), 8.86 (ddd, *J* = 8.1, 2.0, 1.1 Hz, 1H), 8.18 (dd, *J* = 8.1, 6.1 Hz, 1H), 7.89 – 7.78 (m, 2H), 7.68 – 7.54 (m, 3H), 4.64 (dq,

J = 9.3, 6.8 Hz, 1H), 2.08 – 1.94 (m, 2H), 1.92 – 1.82 (m, 1H), 1.78 (d, J = 6.8 Hz, 3H), 1.74 – 1.65 (m, 2H), 1.38 (qt, J = 12.8, 3.1 Hz, 1H), 1.28 – 0.96 (m, 5H); ^{13}C NMR (100 MHz, CD_3OD) δ: 144.82, 143.23, 142.83, 142.25, 134.72, 131.56, 130.81, 129.68, 128.76, 121.81 (q, J = 318.7 Hz), 75.99, 44.80, 30.37, 30.32, 26.82, 26.73, 26.49, 18.85; ^{19}F NMR (375 MHz, CD_3OD) δ: –80.06; *m/z* LRMS (ESI + APCI) found $[M – OTf]^+$ 266.2, $C_{19}H_{24}N^+$ requires 266.2.

(*S*)-1-(1-Cyclohexylethyl)-4-phenylpyridin-1-ium trifluoromethanesulfonate (3j)

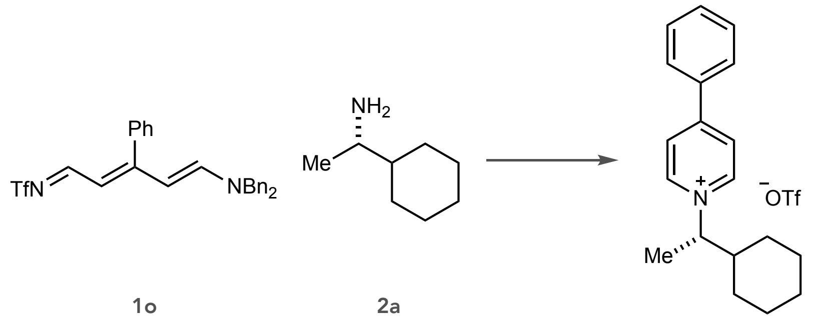

1o 2a

Prepared according to general procedure C using *N*-((1*E*,2*Z*,4*E*)-5-(dibenzylamino)-3-phenylpenta-2,4-dien-1-ylidene)-1,1,1-trifluoromethanesulfonamide (**1n**) (242 mg, 0.500 mmol), MeOH (2.50 mL, 0.2 M), (*S*)-1-cyclohexylethan-1-amine (**2a**) (110 µL, 0.750 mmol), and heated to 70 ºC for 18 h. Isolated according to general isolation procedure C1 using NaOTf (516 mg, 9.00 mmol), CH_2Cl_2 (30 mL), H_2O washes (2 x 15 mL), sat. Na_2CO_3 (15 mL), and 2:1 hexanes/Et_2O (50 mL) to afford the title compound as a clear oil (193 mg, 0.465 mmol, 93% yield). IR ν_{max}/cm^{-1} (film): 3059, 2930, 2856, 1635, 1559, 1488, 1254, 1192, 1151, 634; ^{1}H NMR (400 MHz, CD_3OD) δ: 8.96 (d, J = 7.0 Hz, 2H), 8.40 (d, J = 7.1 Hz, 2H), 8.02 (dd, J = 7.6, 2.1 Hz, 2H), 7.73 – 7.59 (m, 3H), 4.55 (dq, J = 9.2, 6.8 Hz, 1H), 2.06 – 1.82 (m, 3H), 1.78 – 1.59 (m, 5H), 1.37 (dtq, J = 12.9, 9.0, 3.1 Hz, 1H), 1.28 – 1.12 (m, 4H), 1.12 – 0.96 (m, 1H); ^{13}C NMR (100 MHz, CD_3OD) δ: 158.35, 144.48, 135.26, 133.47, 130.97, 129.21, 126.25, 121.82 (q, J = 318.5 Hz), 74.61, 44.88, 30.36, 30.34, 26.84, 26.76, 26.52, 18.76; ^{19}F NMR (375 MHz, CD_3OD) δ: –80.06; *m/z* LRMS (ESI + APCI) found $[M – OTf]^+$ 266.2, $C_{19}H_{24}N^+$ requires 266.2.

(*S*)-1-(1-Cyclohexylethyl)-3-methylpyridin-1-ium trifluoromethanesulfonate (3k)

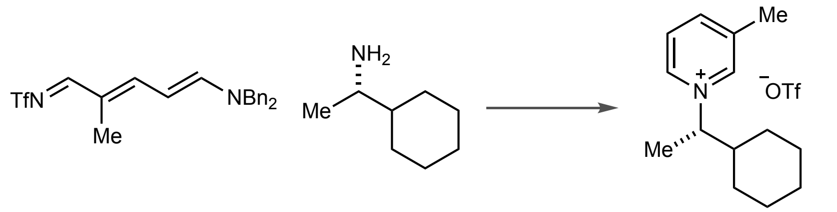

1p 2a

Prepared according to general procedure C using *N*-((1*E*,2*E*,4*E*)-5-(dibenzylamino)-2-methylpenta-2,4-dien-1-ylidene)-1,1,1-trifluoromethanesulfonamide (**1o**) (211 mg, 0.500 mmol), MeOH (2.50 mL, 0.2 M), potassium metabisulfite (111 mg, 0.500 mmol), (*S*)-1-cyclohexylethan-1-amine (**2a**) (110 µL, 0.750 mmol), and heated to 70 ºC for 18 h. Isolated according to general isolation procedure C1 using NaOTf (516 mg, 9.00 mmol), CH_2Cl_2 (30 mL), H_2O washes (2 x 15 mL), sat. Na_2CO_3 (15 mL), and 2:1 hexanes/Et_2O (50 mL) to afford the title compound as a light yellow solid (129 mg, 0.365 mmol, 73% yield). mp 132 – 135 ºC; IR ν_{max}/cm^{-1} (film): 3068, 2931, 2858, 1514, 1451, 1259, 1223, 1161, 1026, 633; ^{1}H NMR (400 MHz, CD_3OD) δ: 8.89 (s, 1H), 8.81 (d, J = 6.1 Hz, 1H), 8.43 (d, J = 7.9 Hz, 1H), 7.99 (dd,

J = 8.0, 6.1 Hz, 1H), 4.49 (dq, J = 9.4, 6.8 Hz, 1H), 2.60 (s, 3H), 2.02 – 1.80 (m, 3H), 1.72 – 1.65 (m, 5H), 1.35 (dtt, J = 16.7, 10.2, 3.5 Hz, 1H), 1.25 – 0.90 (m, 5H); ^{13}C NMR (100 MHz, CD_3OD) δ: 146.27, 142.70, 140.41, 140.22, 127.44, 120.42 (q, J = 318.5 Hz), 74.07, 43.37, 28.94, 28.89, 25.42, 25.32, 25.06, 17.49, 17.08; ^{19}F NMR (375 MHz, CD_3OD) δ: –80.05; *m/z* LRMS (ESI + APCI) found $[M – OTf]^+$ 204.1, $C_{14}H_{22}N^+$ requires 204.2.

(*S*)-1-(1-Cyclohexylethyl)-3-methyl-2-phenylpyridin-1-ium trifluoromethanesulfonate (3l)

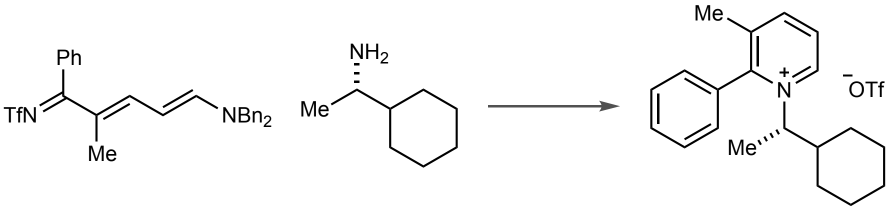

1q 2a

Prepared according to general procedure C using *N*-((1*Z*,2*E*,4*E*)-5-(dibenzylamino)-2-methyl-1-phenylpenta-2,4-dien-1-ylidene)-1,1,1-trifluoromethanesulfonamide (**1p**) (249 mg, 0.500 mmol), MeOH (2.50 mL, 0.2 M), potassium metabisulfite (111 mg, 0.500 mmol), (*S*)-1-cyclohexylethan-1-amine (**2a**) (110 µL, 0.750 mmol), and heated to 70 ºC for 18 h. Isolated according to general isolation procedure C1 using NaOTf (516 mg, 9.00 mmol), CH_2Cl_2 (30 mL), H_2O washes (2 x 15 mL), sat. Na_2CO_3 (15 mL), and 2:1 hexanes/Et_2O (50 mL) to afford the title compound as a light yellow solid (161 mg, 0.375 mmol, 75% yield). mp 164 – 166 ºC; IR ν_{max}/cm^{-1} (film): 3100, 2940, 2856, 1612, 1504, 1448, 1257, 1150, 1030, 755, 636; ^{1}H NMR (400 MHz, CD_3OD) δ: 9.01 (dd, J = 6.4, 1.4 Hz, 1H), 8.52 (dt, J = 7.8, 1.2 Hz, 1H), 8.09 (dd, J = 7.9, 6.4 Hz, 1H), 7.75 – 7.67 (m, 3H), 7.55 – 7.44 (m, 2H), 4.24 (dq, J = 10.0, 6.8 Hz, 1H), 2.23 (s, 3H), 1.98 (qt, J = 11.4, 3.4 Hz, 1H), 1.88 (dt, J = 12.6, 3.6 Hz, 1H), 1.80 – 1.72 (m, 1H), 1.69 – 1.61 (m, 2H), 1.59 (d, J = 6.8 Hz, 3H), 1.31 (qt, J = 13.0, 3.5 Hz, 1H), 1.22 – 1.00 (m, 3H), 0.75 (tdd, J = 12.6, 11.3, 3.5 Hz, 2H); ^{13}C NMR (100 MHz, CD_3OD) δ: 156.71, 147.78, 141.13, 140.83, 132.31, 132.12, 131.24, 131.02, 130.09, 128.97, 128.41, 121.81 (q, J = 318.6 Hz), 70.95, 44.92, 31.03, 30.53, 26.73, 26.65, 26.55, 20.48, 19.54; ^{19}F NMR (375 MHz, CD_3OD) δ: –80.03; *m/z* LRMS (ESI + APCI) found $[M – OTf]^+$ 280.2, $C_{20}H_{26}N^+$ requires 280.2.

(*S*)-1-(1-Cyclohexylethyl)-4-methyl-2-phenylpyridin-1-ium trifluoromethanesulfonate (3m)

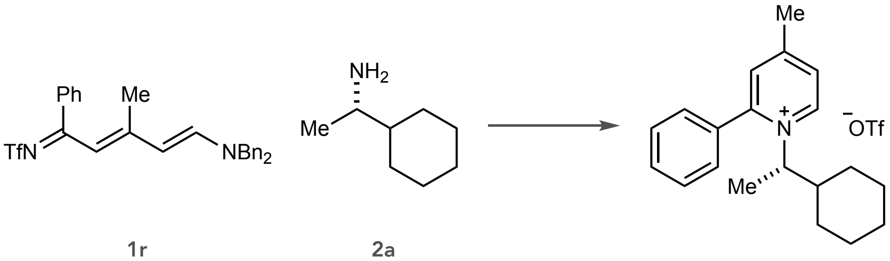

1r 2a

Prepared according to general procedure C using *N*-((1*Z*,2*E*,4*E*)-5-(dibenzylamino)-3-methyl-1-phenylpenta-2,4-dien-1-ylidene)-1,1,1-trifluoromethanesulfonamide (**1q**) (249 mg, 0.500 mmol), MeOH (2.50 mL, 0.2 M), (*S*)-1-cyclohexylethan-1-amine (**2a**) (110 µL, 0.750 mmol), and heated to 70 ºC for 18 h. Isolated according to general isolation procedure C1 using NaOTf (516 mg, 9.00 mmol), CH_2Cl_2 (30 mL), H_2O washes (2 x 15 mL), sat. Na_2CO_3 (15 mL), and 2:1 hexanes/Et_2O (50 mL) to afford the title compound as a brown solid (134 mg, 0.312 mmol, 62% yield). mp 53 – 56 ºC; IR ν_{max}/cm^{-1} (film): 3057, 2928, 2855, 1632, 1490, 1450, 1255, 1147, 1029, 634; ^{1}H NMR (400 MHz, CD_3OD) δ: 9.56 (d, J = 6.6 Hz,

1H), 8.61 (dd, J = 6.7, 2.1 Hz, 1H), 8.47 (d, J = 2.1 Hz, 1H), 8.35 – 8.23 (m, 3H), 8.16 (dd, J = 7.8, 1.8 Hz, 2H), 5.04 (dq, J = 9.8, 6.8 Hz, 1H), 3.31 (s, 3H), 2.58 – 2.42 (m, 2H), 2.40 – 2.27 (m, 4H), 2.24 – 2.15 (m, 2H), 1.89 (qt, J = 12.6, 3.4 Hz, 1H), 1.81 – 1.54 (m, 3H), 1.44 – 1.30 (m, 1H), 1.23 (qd, J = 12.3, 3.5 Hz, 1H); ^{13}C NMR (100 MHz, CD_3OD) δ: 160.91, 156.91, 142.17, 133.47, 132.42, 132.01, 130.59, 130.39, 129.66, 121.82 (q, J = 318.6 Hz), 69.48, 45.38, 30.77, 30.58, 26.68, 26.59, 26.46, 21.84, 19.19; ^{19}F NMR (375 MHz, CD_3OD) δ: –80.03; *m/z* LRMS (ESI + APCI) found $[M – OTf]^+$ 280.2, $C_{20}H_{26}N^+$ requires 280.2.

(*S*)-1-(1-Cyclohexylethyl)-5-methyl-2-phenylpyridin-1-ium trifluoromethanesulfonate (3n)

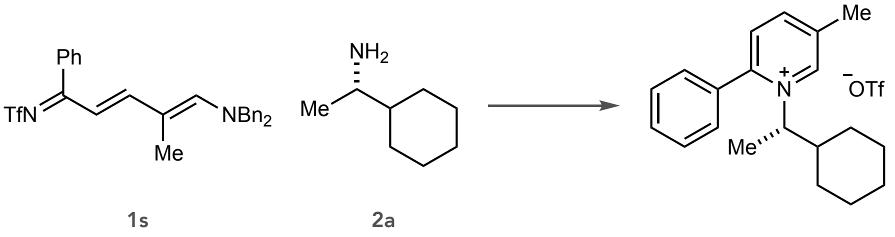

Prepared according to general procedure C using *N*-((1*Z*,2*E*,4*E*)-5-(dibenzylamino)-4-methyl-1-phenylpenta-2,4-dien-1-ylidene)-1,1,1-trifluoromethanesulfonamide (**1r**) (249 mg, 0.500 mmol), MeOH (2.50 mL, 0.2 M), potassium metabisulfite (111 mg, 0.500 mmol), (*S*)-1-cyclohexylethan-1-amine (**2a**) (110 µL, 0.750 mmol), and heated to 70 ºC for 18 h. Isolated according to general isolation procedure C1 using NaOTf (516 mg, 9.00 mmol), CH_2Cl_2 (30 mL), H_2O washes (2 x 15 mL), sat. Na_2CO_3 (15 mL), and 2:1 hexanes/Et_2O (50 mL) to afford the title compound as a yellow solid (174 mg, 0.405 mmol, 81% yield). mp 126 – 128 ºC; IR ν_{max}/cm^{-1} (film): 3065, 2928, 2855, 1630, 1522, 1488, 1254, 1029, 635; ^{1}H NMR (400 MHz, CD_3OD) δ: 9.02 (s, 1H), 8.42 (dd, J = 8.2, 1.1 Hz, 1H), 7.90 (d, J = 8.1 Hz, 1H), 7.72 – 7.64 (m, 3H), 7.60 – 7.52 (m, 2H), 4.49 (dq, J = 10.0, 6.8 Hz, 1H), 2.66 (s, 3H), 2.07 – 1.84 (m, 2H), 1.78 – 1.71 (m, 4H), 1.61 (tdt, J = 10.6, 3.7, 1.7 Hz, 2H), 1.30 (qt, J = 12.7, 3.5 Hz, 1H), 1.20 – 0.93 (m, 3H), 0.77 (tdd, J = 12.7, 11.0, 3.6 Hz, 1H), 0.61 (qd, J = 12.3, 3.5 Hz, 1H); ^{13}C NMR (100 MHz, CD_3OD) δ: 155.23, 147.15, 142.53, 141.06, 133.40, 132.40, 130.92, 130.66, 130.54, 121.81 (q, J = 318.4 Hz), 70.32, 45.31, 30.79, 30.62, 26.69, 26.58, 26.42, 19.26, 18.30; ^{19}F NMR (375 MHz, CD_3OD) δ: –80.04; *m/z* LRMS (ESI + APCI) found $[M – OTf]^+$ 280.2, $C_{20}H_{26}N^+$ requires 280.2.

6.4. Enantioenriched *N*-Alkylpyridinium Salts Prepared from Zincke Imines (HTE Validation)

(*R*)-1-(1-Cyclopropylethyl)-2-phenylpyridin-1-ium trifluoromethanesulfonate (3o)

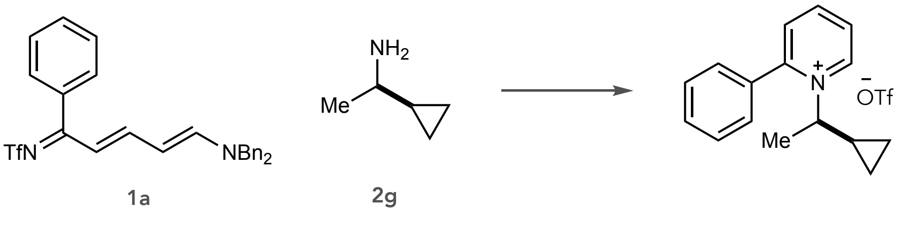

Prepared according to general procedure C using *N*-((1*Z*,2*E*,4*E*)-5-(dibenzylamino)-1-phenylpenta-2,4-dien-1-ylidene)-1,1,1-trifluoromethanesulfonamide (**1a**) (728 mg, 1.50 mmol), MeOH (7.50 mL, 0.2 M), potassium metabisulfite (334 mg, 1.50 mmol), (*R*)-1-cyclopropylethan-1-amine (**2g**) (210 µL, 2.25 mmol), and heated to 70 ºC for 18 h. 1,3,5-Trimethylbenzene (209 µL, 1.50 mmol, 1.0 equiv.) was added as the

internal standard for ^{1}H NMR analysis (67% yield). Isolated according to general isolation procedure C2 using H_2O (20 mL), Na_2CO_3 (795 mg, 7.50 mmol), EtOAc for first extractions (3 x 30 mL), H_2O for back extraction (10 mL), NaOTf (2.32 g, 13.5 mmol), CH_2Cl_2 for extraction of pyridinium salt (3 x 30 mL), and 2:1 hexanes/Et_2O (100 mL) to afford the title compound as a clear yellow oil (343 mg, 0.919 mmol, 61% yield). IR ν_{max}/cm^{-1} (film): 3091, 1622, 1508, 1484, 1256, 1223, 1147, 1028, 635; ^{1}H NMR (400 MHz, CD_3OD) δ: 9.38 (dd, *J* = 6.5, 1.4 Hz, 1H), 8.61 (td, *J* = 7.8, 1.4 Hz, 1H), 8.22 (ddd, *J* = 7.8, 6.4, 1.6 Hz, 1H), 8.01 (dd, *J* = 8.0, 1.6 Hz, 1H), 7.75 – 7.61 (m, 3H), 7.59 – 7.54 (m, 2H), 4.16 (dq, *J* = 9.2, 6.8 Hz, 1H), 1.71 (d, *J* = 6.8 Hz, 3H), 1.55 (qd, *J* = 8.8, 4.0 Hz, 1H), 0.81 (tt, *J* = 8.9, 5.5 Hz, 1H), 0.59 (tt, *J* = 7.7, 5.4 Hz, 1H), 0.39 (ddt, *J* = 9.5, 5.9, 4.8 Hz, 1H), 0.13 (ddd, *J* = 9.3, 6.0, 4.8 Hz, 1H); ^{13}C NMR (100 MHz, CD_3OD) δ: 157.03, 146.49, 143.64, 133.19, 132.57, 131.70, 130.70, 129.85, 129.06, 121.81 (q, *J* = 318.2 Hz), 70.85, 21.33, 18.05, 6.42, 4.85; ^{19}F NMR (375 MHz, CD_3OD) δ: –80.06; *m/z* LRMS (ESI + APCI) found [M – OTf]$^+$ 224.1, $C_{16}H_{18}N^+$ requires 224.1.

(*S*)-1-(1-Phenylethyl)-2-(thiophen-3-yl)pyridin-1-ium trifluoromethanesulfonate (3p)

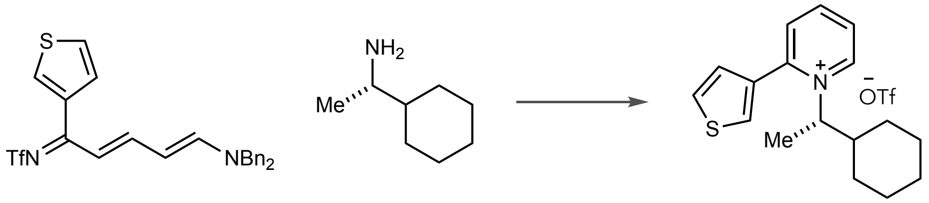

1d **2a**

Prepared according to general procedure C using *N*-((1*Z*,2*E*,4*E*)-5-(dibenzylamino)-1-(thiophen-3-yl)penta-2,4-dien-1-ylidene)-1,1,1-trifluoromethanesulfonamide (**1d**) (736 mg, 1.50 mmol), MeOH (7.50 mL, 0.2 M), potassium metabisulfite (334 mg, 1.50 mmol), (*S*)-1-cyclohexylethan-1-amine (**2a**) (330 μL, 2.25 mmol), and heated to 70 ºC for 18 h. 1,3,5-Trimethylbenzene (209 μL, 1.50 mmol, 1.0 equiv.) was added as the internal standard for ^{1}H NMR analysis (100% yield). Isolated according to general isolation procedure C1 using NaOTf (1.55 g, 9.00 mmol), 3:1 CH_2Cl_2:IPA (60 mL), H_2O washes (2 x 40 mL), sat. Na_2CO_3 (40 mL), and 2:1 hexanes/Et_2O (100 mL) to afford the title compound as a light yellow solid (592 mg, 1.40 mmol, 92% yield). mp 154 – 156 ºC; IR ν_{max}/cm^{-1} (film): 3103, 2926, 2856, 1219, 1572, 1447, 1257, 1152, 1053, 800, 637; ^{1}H NMR (400 MHz, CD_3OD) δ: 9.14 (dd, *J* = 6.5, 1.4 Hz, 1H), 8.57 (td, *J* = 7.8, 1.4 Hz, 1H), 8.15 (ddd, *J* = 7.8, 6.3, 1.6 Hz, 1H), 8.07 (dd, *J* = 8.0, 1.6 Hz, 1H), 8.01 (dd, *J* = 3.0, 1.4 Hz, 1H), 7.84 (dd, *J* = 5.0, 2.9 Hz, 1H), 7.39 (dd, *J* = 5.0, 1.4 Hz, 1H), 4.67 (dq, *J* = 9.7, 6.8 Hz, 1H), 2.02 – 1.86 (m, 2H), 1.80 – 1.71 (m, 4H), 1.66 – 1.55 (m, 2H), 1.30 (qt, *J* = 12.7, 3.5 Hz, 1H), 1.20 – 1.01 (m, 2H), 0.99 – 0.80 (m, 2H), 0.65 (qd, *J* = 12.3, 3.6 Hz, 1H); ^{13}C NMR (100 MHz, CD_3OD) δ: 153.82, 146.31, 143.51, 133.06, 131.95, 131.21, 130.22, 128.87, 128.60, 121.81 (q, *J* = 318.6 Hz), 70.35, 45.50, 30.60, 30.55, 26.68, 26.55, 26.39, 19.14; ^{19}F NMR (375 MHz, CD_3OD) δ: –80.06; *m/z* LRMS (ESI + APCI) found [M – OTf]$^+$ 272.3, $C_{17}H_{22}NS^+$ requires 272.2.

1-((3a*S*,4*R*,6*S*,6a*R*)-6-Hydroxy-2,2-dimethyltetrahydro-4*H*-cyclopenta[*d*][1,3]dioxol-4-yl)-2-isopropylpyridin-1-ium trifluoromethanesulfonate (3q)

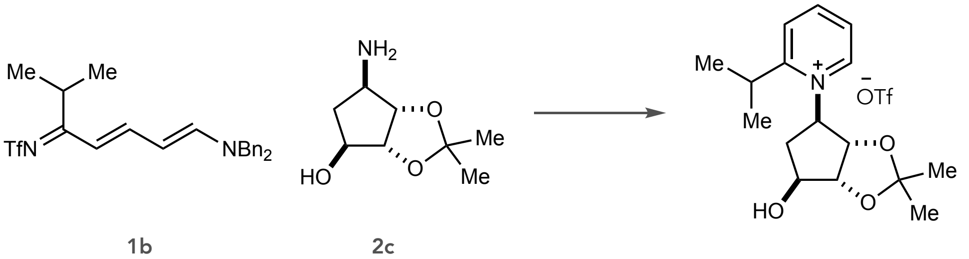

Prepared according to general procedure C using *N*-((3*E*,4*E*,6*E*)-7-(dibenzylamino)-2-methylhepta-4,6-dien-3-ylidene)-1,1,1-trifluoromethanesulfonamide (**1b**) (676 mg, 1.50 mmol), MeOH (7.50 mL, 0.2 M), potassium metabisulfite (334 mg, 1.50 mmol), (3a*R*,4*S*,6*R*,6a*S*)-6-amino-2,2-dimethyltetrahydro-4*H*-cyclopenta[*d*][1,3]dioxol-4-ol (**2c**) (390 mg, 2.25 mmol), and heated to 70 ºC for 18 h. 1,3,5-Trimethylbenzene (209 µL, 1.50 mmol, 1.0 equiv.) was added as the internal standard for ^{1}H NMR analysis (80% yield). Isolated according to general isolation procedure C2 using H_2O (20 mL), Na_2CO_3 (795 mg, 7.50 mmol), EtOAc for first extractions (3 x 30 mL), H_2O for back extraction (10 mL), NaOTf (2.32 g, 13.5 mmol), 3:1 CH_2Cl_2:IPA for extraction of pyridinium salt (3 x 30 mL), and 2:1 hexanes/Et_2O (100 mL) to afford the title compound as a yellow oil (449 mg, 1.05 mmol, 70% yield). The O–H proton was exchanged with deuterium from CD_3OD during characterization. IR ν_{max}/cm^{-1} (film): 3411, 3155, 2980, 2941, 1628, 1503, 1385, 1272, 1250, 1025, 634; ^{1}H NMR (400 MHz, CD_3OD) δ: 9.32 (dd, *J* = 6.5, 1.4 Hz, 1H), 8.52 – 8.45 (m, 1H), 8.12 (dd, *J* = 8.2, 1.6 Hz, 1H), 7.93 (ddd, *J* = 7.9, 6.5, 1.6 Hz, 1H), 5.43 (dt, *J* = 8.9, 2.9 Hz, 1H), 5.03 (dt, *J* = 5.9, 1.6 Hz, 1H), 4.73 (dt, *J* = 5.7, 1.5 Hz, 1H), 4.44 (ddd, *J* = 5.3, 2.4, 1.4 Hz, 1H), 3.81 (hept, *J* = 6.8 Hz, 1H), 3.03 (ddd, *J* = 15.5, 9.0, 5.3 Hz, 1H), 2.27 (dtd, *J* = 15.6, 2.3, 1.2 Hz, 1H), 1.55 (s, 3H), 1.53 – 1.44 (m, 6H), 1.35 (s, 3H); ^{13}C NMR (100 MHz, CD_3OD) δ: 165.99, 146.81, 145.21, 127.26, 126.71, 121.79 (q, *J* = 318.6 Hz), 113.85, 88.49, 88.47, 76.18, 73.66, 39.99, 31.79, 27.04, 24.73, 22.91, 22.12; ^{19}F NMR (377 MHz, CD_3OD) δ: –80.09; *m/z* LRMS (ESI + APCI) found [M – OTf]$^+$ 278.2, $C_{16}H_{24}NO_3^+$ requires 278.2.

6'-(Trifluoromethyl)-1-((1*R*,2*R*)-1,7,7-trimethylbicyclo[2.2.1]heptan-2-yl)-[3,3'-bipyridin]-1-ium trifluoromethanesulfonate (3r)

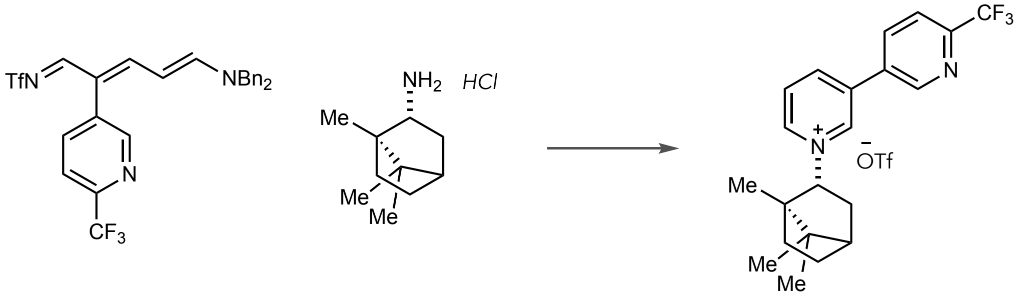

Prepared according to general procedure C using *N*-((1*E*,2*E*,4*E*)-5-(dibenzylamino)-2-(6-(trifluoromethyl)pyridin-3-yl)penta-2,4-dien-1-ylidene)-1,1,1-trifluoromethanesulfonamide (**1e**) (830 mg, 1.50 mmol), MeOH (7.50 mL, 0.2 M), potassium metabisulfite (334 mg, 1.50 mmol), (1*R*,2*R*)-1,7,7-trimethylbicyclo[2.2.1]heptan-2-amine hydrochloride (**2v**) (427 mg, 2.25 mmol), and heated to 70 ºC for 18 h. 1,3,5-Trimethylbenzene (209 µL, 1.50 mmol, 1.0 equiv.) was added as the internal standard for ^{1}H NMR analysis (91% yield). Isolated according to general isolation procedure C1 using NaOTf (1.55 g, 9.00 mmol), CH_2Cl_2 (60 mL), H_2O washes (2 x 40 mL), sat. Na_2CO_3 (40 mL), and 2:1 hexanes/Et_2O (100 mL) to afford the title compound as a light-yellow solid (648 mg, 1.27 mmol, 85% yield). mp 79 – 82 ºC; IR ν_{max}/cm^{-1} (film): 3074, 2962, 1631, 1518, 1373, 1253, 1137, 1028, 635; ^{1}H NMR (400 MHz, CD_3OD)

δ: 9.45 (d, *J* = 1.8 Hz, 1H), 9.29 (dt, *J* = 6.3, 1.4 Hz, 1H), 9.13 (d, *J* = 2.3 Hz, 1H), 8.96 (dt, *J* = 8.2, 1.3 Hz, 1H), 8.47 (dd, *J* = 8.3, 2.3 Hz, 1H), 8.26 (dd, *J* = 8.0, 6.3 Hz, 1H), 8.05 (d, *J* = 8.2 Hz, 1H), 5.02 (dd, *J* = 9.1, 6.8 Hz, 1H), 2.95 (ddq, *J* = 14.0, 7.0, 3.4 Hz, 1H), 2.23 (dd, *J* = 14.0, 9.2 Hz, 1H), 2.14 (t, *J* = 4.3 Hz, 1H), 2.04 – 1.93 (m, 1H), 1.87 (td, *J* = 12.3, 4.7 Hz, 1H), 1.65 (ddd, *J* = 12.9, 9.3, 3.5 Hz, 1H), 1.46 (ddd, *J* = 12.3, 9.3, 4.7 Hz, 1H), 1.00 (s, 3H), 0.97 (s, 3H), 0.94 (s, 3H); ^{13}C NMR (100 MHz, CD_3OD) δ: 150.12, 150.02 (q, *J* = 35.3 Hz), 145.84, 145.15, 144.93, 138.94, 138.31, 134.26, 129.18, 122.86 (q, *J* = 273.6 Hz), 122.23 (q, *J* = 2.8 Hz), 121.78 (q, *J* = 318.3 Hz), 82.04, 53.22, 49.29, 46.70, 38.39, 34.99, 27.41, 21.72, 20.61, 12.81; ^{19}F NMR (375 MHz, CD_3OD) δ: –69.51, –80.07; *m/z* LRMS (ESI + APCI) found [M – OTf]$^+$ 361.3, $C_{21}H_{24}F_3N_2^+$ requires 361.2.

1-((3*S*,3a*R*,6*R*,6a*R*)-6-((Tert-butoxycarbonyl)amino)hexahydrofuro[3,2-*b*]furan-3-yl)-5-methoxy-2-(4-methoxyphenyl)pyridin-1-ium trifluoromethanesulfonate (3s)

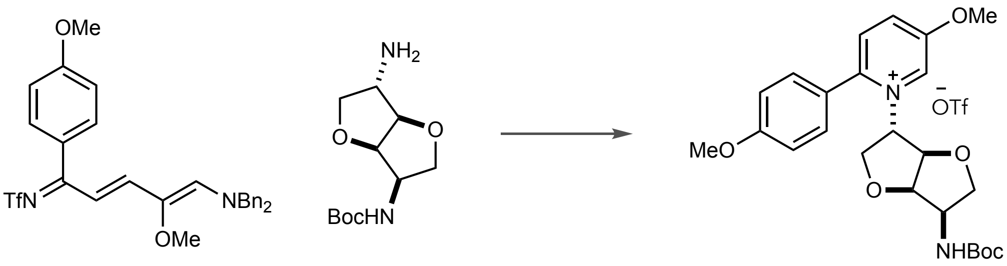

1k 2x

Prepared according to general procedure C using *N*-((1*Z*,2*E*,4*Z*)-5-(dibenzylamino)-4-methoxy-1-(4-methoxyphenyl)penta-2,4-dien-1-ylidene)-1,1,1-trifluoromethanesulfonamide (**1k**) (817 mg, 1.50 mmol), MeOH (7.50 mL, 0.2 M), potassium metabisulfite (334 mg, 1.50 mmol), *tert*-butyl ((3*R*,3a*R*,6*S*,6a*R*)-6-aminohexahydrofuro[3,2-*b*]furan-3-yl)carbamate (**2x**) (550 mg, 2.25 mmol), and heated to 70 ºC for 18 h. 1,3,5-Trimethylbenzene (209 µL, 1.50 mmol, 1.0 equiv.) was added as the internal standard for ^{1}H NMR analysis (94% yield). Isolated according to general isolation procedure C1 using NaOTf (1.55 g, 9.00 mmol), CH_2Cl_2 (60 mL), H_2O washes (2 x 40 mL), sat. Na_2CO_3 (40 mL), and 2:1 hexanes/Et_2O (100 mL) to afford the title compound as an orange/brown solid (818 mg, 1.38 mmol, 92% yield). The N–H proton was exchanged with deuterium from CD_3OD during characterization. mp 100 – 104 ºC; IR ν_{max}/cm^{-1} (film): 3339, 2977, 1702, 1608, 1501, 1251, 1156, 1029, 636; ^{1}H NMR (400 MHz, CD_3OD) δ: 8.45 (d, *J* = 2.6 Hz, 1H), 8.23 (dd, *J* = 9.0, 2.6 Hz, 1H), 7.94 (d, *J* = 9.0 Hz, 1H), 7.52 (d, *J* = 8.8 Hz, 2H), 7.18 (d, *J* = 8.8 Hz, 2H), 5.20 (dd, *J* = 6.0, 2.1 Hz, 1H), 5.08 (d, *J* = 4.8 Hz, 1H), 4.96 (t, *J* = 5.2 Hz, 1H), 4.47 (d, *J* = 12.1 Hz, 1H), 4.29 – 4.18 (m, 2H), 4.11 (s, 3H), 4.01 (dd, *J* = 8.8, 7.7 Hz, 1H), 3.91 (s, 3H), 3.42 (t, *J* = 9.1 Hz, 1H), 1.46 (s, 9H); ^{13}C NMR (100 MHz, CD_3OD) δ: 163.38, 160.05, 157.92, 150.25, 132.57, 132.43, 131.82, 130.12, 124.46, 121.77 (q, *J* = 318.6 Hz), 115.93, 89.99, 83.07, 80.73, 74.75, 74.68, 71.34, 57.97, 56.13, 55.43, 28.66; ^{19}F NMR (375 MHz, CD_3OD) δ: –80.03; *m/z* LRMS (ESI + APCI) found [M – OTf]$^+$ 443.1, $C_{24}H_{31}N_2O_6^+$ requires 443.2.

(*S*)-3-Bromo-1-(1,1-dioxidothiochroman-4-yl)-5-methylpyridin-1-ium trifluoromethanesulfonate (3t)

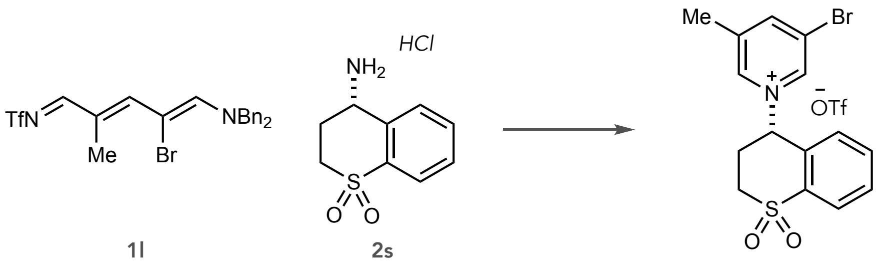

1l 2s

Prepared according to general procedure C using *N*-((1*E*,2*E*,4*Z*)-4-bromo-5-(dibenzylamino)-2-methylpenta-2,4-dien-1-ylidene)-1,1,1-trifluoromethanesulfonamide (**1l**) (545 mg, 1.50 mmol), MeOH (7.50 mL, 0.2 M), potassium metabisulfite (334 mg, 1.50 mmol), (*S*)-4-aminothiochromane 1,1-dioxide hydrochloride (**2s**) (526 mg, 2.25 mmol), and heated to 70 ºC for 18 h. 1,3,5-Trimethylbenzene (209 μL, 1.50 mmol, 1.0 equiv.) was added as the internal standard for ^{1}H NMR analysis (94% yield). Isolated according to general isolation procedure C1 using NaOTf (1.55 g, 9.00 mmol), 3:1 CH_2Cl_2:IPA (60 mL), H_2O washes (2 x 40 mL), sat. Na_2CO_3 (40 mL), and 2:1 hexanes/Et_2O (100 mL) to afford the title compound as a tan solid (603 mg, 1.20 mmol, 80% yield). mp 194 – 197 ºC; IR ν_{max}/cm^{-1} (film): 3066, 1580, 1485, 1290, 1251, 1157, 1029, 985, 760, 637; ^{1}H NMR (400 MHz, DMSO) δ: 9.37 (s, 1H), 8.91 (s, 2H), 8.01 (dd, *J* = 7.9, 1.4 Hz, 1H), 7.78 – 7.69 (m, 1H), 7.66 (td, *J* = 7.7, 1.5 Hz, 1H), 7.12 (d, *J* = 7.9 Hz, 1H), 6.41 (dd, *J* = 8.8, 5.9 Hz, 1H), 3.90 (ddd, *J* = 14.5, 6.9, 3.5 Hz, 1H), 3.80 (ddd, *J* = 14.3, 10.0, 4.0 Hz, 1H), 3.13 – 2.96 (m, 2H), 2.47 (s, 3H); ^{13}C NMR (100 MHz, DMSO) δ: 149.66, 143.80, 142.90, 140.97, 139.58, 133.40, 131.68, 130.84, 128.98, 123.86, 122.05, 120.67 (q, *J* = 322.2 Hz), 67.59, 47.26, 28.60, 17.81; ^{19}F NMR (375 MHz, DMSO) δ: –77.74; *m/z* LRMS (ESI + APCI) found [M – OTf]$^+$ 352.1, $C_{15}H_{14b}rNO_2S^+$ requires 352.0.

(*S*)-1-(1-Cyclohexylethyl)-2-ethylpyridin-1-ium trifluoromethanesulfonate (3v)

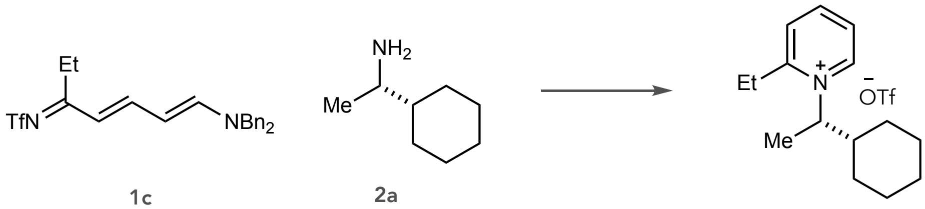

1c 2a

Prepared according to general procedure C (with **2a** as the limiting reagent) using (*S*)-1-cyclohexylethan-1-amine (**2a**) (79.0 μL, 0.500 mmol), *N*-((3*E*,4*E*,6*E*)-7-(dibenzylamino)hepta-4,6-dien-3-ylidene)-1,1,1-trifluoromethanesulfonamide (**1c**) (327 mg, 0.750 mmol), MeOH (2.50 mL, 0.2 M), potassium metabisulfite (111 mg, 0.500 mmol), and heated to 70 ºC for 18 h. 1,3,5-Trimethylbenzene (69.5 μL, 0.500 mmol, 1.0 equiv.) was added as the internal standard for ^{1}H NMR analysis (71% yield). Isolated according to general isolation procedure C2 using H_2O (20 mL), Na_2CO_3 (265 mg, 2.50 mmol), EtOAc for first extractions (3 x 20 mL), H_2O for back extraction (1 x 10 mL), NaOTf (774 mg, 4.50 mmol), CH_2Cl_2 for extraction of pyridinium salt (3 x 20 mL) to afford the title compound as a yellow solid (124 mg, 0.337 mmol, 67% yield). mp 77 – 80 ºC; IR ν_{max}/cm^{-1} (film): 3088, 2932, 2857, 1630, 1271, 1257, 1026, 631; ^{1}H NMR (400 MHz, $CDCl_3$) δ: 9.04 (dd, *J* = 6.5, 1.5 Hz, 1H), 8.40 (td, *J* = 7.8, 1.4 Hz, 1H), 8.11 (t, *J* = 7.0 Hz, 1H), 7.91 (dd, *J* = 8.2, 1.6 Hz, 1H), 4.64 (dq, *J* = 9.7, 6.8 Hz, 1H), 3.30 – 3.06 (m, 2H), 2.06 – 1.95 (m, 2H), 1.87 – 1.78 (m, 2H), 1.72 (d, *J* = 6.7 Hz, 3H), 1.68 – 1.60 (m, 1H), 1.46 (t, *J* = 7.5 Hz, 3H), 1.41 – 1.28 (m, 1H), 1.21 – 1.05 (m, 3H), 1.04 – 0.96 (m, 1H), 0.86 (qd, *J* = 12.1, 3.5 Hz, 1H); ^{13}C NMR (100

MHz, $CDCl_3$) δ: 158.91, 145.35, 143.12, 128.31, 127.41, 120.94 (q, J = 320.6 Hz), 67.29, 43.84, 29.82, 29.68, 26.94, 25.73, 25.54, 25.37, 19.58, 12.82; ^{19}F NMR (375 MHz, $CDCl_3$) δ: –78.24; *m/z* LRMS (ESI + APCI) found $[M – OTf]^+$ 218.1, $C_{15}H_{24}N^+$ requires 218.2.

2-(4-(((*R*)-But-3-yn-2-yl)oxy)phenyl)-1-((*S*)-1-cyclohexylethyl)pyridin-1-ium trifluoromethanesulfonate (3aa)

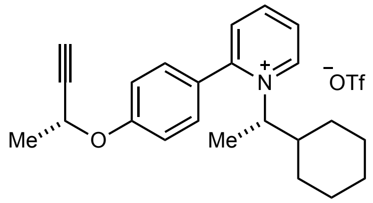

Prepared according to general procedure C using *N*-((1*Z*,2*E*,4*E*)-1-(4-(((*R*)-But-3-yn-2-yl)oxy)phenyl)-5-(dibenzylamino)penta-2,4-dien-1-ylidene)-1,1,1-trifluoromethanesulfonamide (**1s**) (221 mg, 0.400 mmol), MeOH (2.00 mL, 0.2 M), potassium metabisulfite (88.9 mg, 0.400 mmol), (*S*)-1-cyclohexylethan-1-amine (**2a**) (88.0 µL, 0.600 mmol), and heated to 70 ºC for 18 h. 1,3,5-Trimethylbenzene (56.0 µL, 0.400 mmol, 1.0 equiv.) was added as the internal standard for ^{1}H NMR analysis (94% yield, single diastereomer). Isolated according to general isolation procedure C1 using NaOTf (423 mg, 2.40 mmol), CH_2Cl_2 (30 mL), H_2O washes (3 x 15 mL), CH_2Cl_2 extractions (2 x 10 mL), sat. Na_2CO_3 wash (1 x 20 mL) and 2:1 hexanes/Et_2O (50 mL) to afford the title compound as a single diastereomer as a yellow solid (165 mg, 0.341 mmol, 85% yield). mp 64 – 67 ºC; IR ν_{max}/cm^{-1} (film): 3087, 2931, 2856, 1623, 1488, 1244, 1149, 1028, 635; ^{1}H NMR (400 MHz, CD_3OD) δ: 9.15 (dd, J = 6.4, 1.5 Hz, 1H), 8.58 (td, J = 7.8, 1.4 Hz, 1H), 8.16 (ddd, J = 7.8, 6.4, 1.6 Hz, 1H), 8.05 (dd, J = 8.0, 1.6 Hz, 1H), 7.54 (d, J = 9.0 Hz, 2H), 7.32 (d, J = 8.4 Hz, 2H), 5.16 (qd, J = 6.5, 2.0 Hz, 1H), 4.66 (dq, J = 9.9, 6.8 Hz, 1H), 3.05 (d, J = 2.0 Hz, 1H), 1.98 – 1.87 (m, 2H), 1.80 – 1.74 (m, 4H), 1.71 (d, J = 6.5 Hz, 3H), 1.67 – 1.57 (m, 2H), 1.37 – 1.22 (m, 1H), 1.10 (tdd, J = 15.9, 10.8, 3.1 Hz, 2H), 0.93 (dd, J = 9.1, 5.8 Hz, 1H), 0.84 (qd, J = 13.1, 3.7 Hz, 1H), 0.64 (qd, J = 12.2, 3.7 Hz, 1H); ^{13}C NMR (100 MHz, CD_3CN) δ: 160.36, 145.83, 142.79, 131.79, 128.47, 125.54, 122.13 (q, J = 320.8 Hz), 117.25, 83.13, 75.92, 69.68, 64.67, 45.17, 30.08, 29.85, 26.23, 26.21, 26.02, 22.40, 19.17; ^{19}F NMR (377 MHz, CD_3OD) δ: –80.10; *m/z* LRMS (ESI + APCI) found $[M – OTf]^+$ 334.3, $C_{23}H_{28}NO^+$ requires 334.2.

2-(4-(((*R*)-But-3-yn-2-yl)oxy)phenyl)-1-((*R*)-1,1,1-trifluoropropan-2-yl)pyridin-1-ium trifluoromethanesulfonate (3ab)

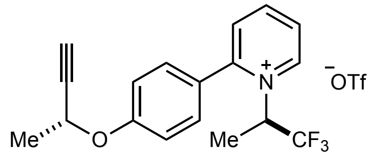

Prepared according to general procedure C using *N*-((1*Z*,2*E*,4*E*)-1-(4-(((*R*)-But-3-yn-2-yl)oxy)phenyl)-5-(dibenzylamino)penta-2,4-dien-1-ylidene)-1,1,1-trifluoromethanesulfonamide (**1s**) (221 mg, 0.400 mmol), MeOH (2.00 mL, 0.2 M), potassium metabisulfite (88.9 mg, 0.400 mmol), (*R*)-1,1,1-trifluoropropan-2-amine hydrochloride (**2d**) (89.7 mg, 0.600 mmol), and heated to 70 ºC for 18 h. 1,3,5-Trimethylbenzene (56.0 µL, 0.400 mmol, 1.0 equiv.) was added as the internal standard for ^{1}H NMR analysis (67% yield, single diastereomer). Isolated according to general isolation procedure C1 using NaOTf (423 mg, 2.40 mmol), CH_2Cl_2 (30 mL), H_2O washes (3 x 15 mL), CH_2Cl_2 extractions (2 x 10 mL), sat. Na_2CO_3 wash (1 x 20 mL) and 2:1 hexanes/Et_2O (50 mL) to afford the title compound as a single diastereomer as an orange

solid with an unknown impurity (10.8:1) (110 mg, 0.234 mmol, 59% yield). IR ν_{max}/cm^{-1} (film): 3084, 1623, 1604, 1491, 1254, 1158, 1029, 636; ^{1}H NMR (400 MHz, CD_3CN) δ: 9.01 (d, *J* = 5.8 Hz, 1H), 8.68 (td, *J* = 7.8, 1.3 Hz, 1H), 8.22 (td, *J* = 7.1, 1.6 Hz, 1H), 8.08 (dd, *J* = 8.0, 1.6 Hz, 1H), 7.52 (d, *J* = 8.8 Hz, 2H), 7.30 (d, *J* = 8.9 Hz, 2H), 5.72 (p, *J* = 6.9 Hz, 1H), 5.16 (qd, *J* = 6.6, 2.1 Hz, 1H), 2.91 (d, *J* = 2.0 Hz, 1H), 1.93 (d, *J* = 7.1 Hz, 3H), 1.70 (d, *J* = 6.5 Hz, 3H); ^{13}C NMR (100 MHz, CD_3CN) δ: 160.79, 158.61, 148.71, 143.77, 132.97, 131.75, 128.97, 124.30, 124.17 (q, *J* = 281.9 Hz), 122.12 (q, *J* = 321.0 Hz), 117.53, 83.03, 75.99, 64.69, 62.39 (q, *J* = 32.5 Hz), 22.36, 14.58 (q, *J* = 1.1 Hz); ^{19}F NMR (375 MHz, CD_3CN) δ: -74.22 (d, *J* = 6.8 Hz), –79.32; *m/z* LRMS (ESI + APCI) found [M – OTf]$^+$ 320.1, $C_{18}H_{17}F_3NO^+$ requires 320.1.

2-(4-(((*R*)-But-3-yn-2-yl)oxy)phenyl)-1-((*S*)-1-(2-fluorophenyl)ethyl)pyridin-1-ium trifluoromethanesulfonate (3ac)

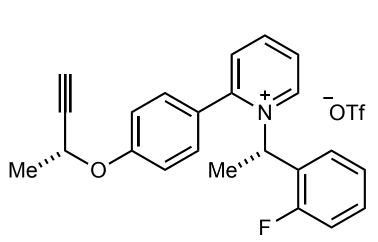

Prepared according to general procedure C using *N*-((1*Z*,2*E*,4*E*)-1-(4-(((*R*)-But-3-yn-2-yl)oxy)phenyl)-5-(dibenzylamino)penta-2,4-dien-1-ylidene)-1,1,1-trifluoromethanesulfonamide (**1s**) (221 mg, 0.400 mmol), MeOH (2.00 mL, 0.2 M), potassium metabisulfite (88.9 mg, 0.400 mmol), (*S*)-1-(2-fluorophenyl)ethan-1-amine (**2ao**) (79.0 μL, 0.600 mmol), and heated to 70 ºC for 18 h. 1,3,5-Trimethylbenzene (56.0 μL, 0.400 mmol, 1.0 equiv.) was added as the internal standard for ^{1}H NMR analysis (93% yield, single diastereomer). Isolated according to general isolation procedure C1 using NaOTf (413 mg, 2.40 mmol), CH_2Cl_2 (30 mL), H_2O washes (3 x 15 mL), CH_2Cl_2 extractions (2 x 10 mL), sat. Na_2CO_3 wash (1 x 20 mL) and 2:1 hexanes/Et_2O (50 mL) to afford the title compound as a single diastereomer as a light yellow solid (163 mg, 0.329 mmol, 82% yield). mp 59 – 62 ºC; IR ν_{max}/cm^{-1} (film): 3082, 1623, 1605, 1487, 1148, 1028, 937, 634; ^{1}H NMR (400 MHz, CD_3CN) δ: 8.62 (d, *J* = 6.4 Hz, 1H), 8.47 (td, *J* = 7.8, 1.4 Hz, 1H), 7.98 – 7.89 (m, 2H), 7.60 – 7.45 (m, 4H), 7.32 (td, *J* = 7.6, 1.2 Hz, 1H), 7.25 (d, *J* = 9.1 Hz, 2H), 7.09 (ddd, *J* = 11.1, 8.3, 1.2 Hz, 1H), 6.27 (q, *J* = 7.0 Hz, 1H), 5.13 (qd, *J* = 6.5, 2.0 Hz, 1H), 2.87 (d, *J* = 2.0 Hz, 1H), 1.99 (d, *J* = 7.0 Hz, 3H), 1.67 (d, *J* = 6.5 Hz, 3H); ^{13}C NMR (100 MHz, CD_3CN) δ: 161.53 (d, *J* = 247.9 Hz), 160.45, 157.06, 146.57, 143.57, 133.13 (d, *J* = 8.8 Hz), 132.12, 131.46, 129.64 (d, *J* = 2.9 Hz), 128.41, 126.11 (d, *J* = 3.4 Hz), 125.52 (d, *J* = 13.5 Hz), 125.27, 122.13 (q, *J* = 321.0 Hz), 117.11, 116.85 (d, *J* = 21.1 Hz), 83.14, 75.89, 64.63, 61.89 (d, *J* = 2.5 Hz), 22.38, 20.37; ^{19}F NMR (375 MHz, CD_3CN) δ: –79.31, –116.00 (dt, *J* = 12.8, 6.2 Hz); *m/z* LRMS (ESI + APCI) found [M – OTf]$^+$ 346.2, $C_{23}H_{21}FNO^+$ requires 346.2.

2-(4-(((*R*)-But-3-yn-2-yl)oxy)phenyl)-1-((*S*)-1-(pyridin-2-yl)ethyl)pyridin-1-ium trifluoromethanesulfonate (3ad) (major and minor)

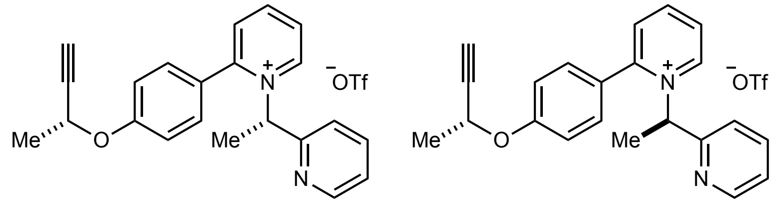

23 : 1

Prepared according to general procedure C using *N*-((1*Z*,2*E*,4*E*)-1-(4-(((*R*)-But-3-yn-2-yl)oxy)phenyl)-5-(dibenzylamino)penta-2,4-dien-1-ylidene)-1,1,1-trifluoromethanesulfonamide (**1s**) (221 mg, 0.400 mmol), MeOH (2.00 mL, 0.2 M), potassium metabisulfite (88.9 mg, 0.400 mmol), (*S*)-1-(pyridin-2-yl)ethan-1-amine (72.0 µL, 0.600 mmol), and heated to 70 ºC for 18 h. 1,3,5-Trimethylbenzene (56.0 µL, 0.400 mmol, 1.0 equiv.) was added as the internal standard for ^{1}H NMR analysis (86% yield, 23:1 d.r.). Isolated according to general isolation procedure C1 using NaOTf (413 mg, 2.40 mmol), CH_2Cl_2 (30 mL), H_2O washes (3 x 15 mL), CH_2Cl_2 extractions (2 x 10 mL), sat. Na_2CO_3 wash (1 x 20 mL) and 2:1 hexanes/Et_2O (50 mL) to afford the title compound as a mixture of diastereomers (25:1 d.r.) as a brown oil (145 mg, 0.303 mmol, 76% yield). IR ν_{max}/cm^{-1} (film): 3082, 2991, 1623, 1489, 1256, 1182, 1028, 781, 753, 635; ^{1}H NMR (major and minor, 400 MHz, CD_3CN) δ: 9.01 – 8.94 (m, 1H), 8.55 – 8.44 (m, 2H), 8.06 – 7.97 (m, 1H), 7.94 – 7.87 (m, 1H), 7.83 – 7.73 (m, 1H), 7.47 – 7.32 (m, 3H), 7.23 – 7.15 (m, 3H), 6.23 – 6.13 (m, 1H), 5.15 – 5.06 (m, 1H), 2.87 (major, d, *J* = 2.0 Hz, 1H), 2.69 (minor, d, *J* = 2.1 Hz, 1H), 1.96 – 1.94 (m, 3H), 1.66 – 1.64 (m, 3H); ^{13}C NMR (major, 100 MHz, CD_3OD) δ: 161.20, 161.16, 157.86, 157.45, 151.02, 146.72, 145.16, 139.09, 131.82, 131.57, 131.54, 127.91, 125.72, 125.23, 123.36, 121.80 (q, *J* = 318.6 Hz), 117.51, 67.71, 64.93, 22.50, 21.08; ^{19}F NMR (major, 375 MHz, CD_3CN) δ: –79.30; *m/z* LRMS (ESI + APCI) found $[M – OTf]^{+}$ 329.2, $C_{22}H_{21}N_2O^{+}$ requires 329.2.

2-(4-(((*R*)-But-3-yn-2-yl)oxy)phenyl)-1-((*S*)-1-methoxy-3-methyl-1-oxobutan-2-yl)pyridin-1-ium trifluoromethanesulfonate (3ae) (major and minor)

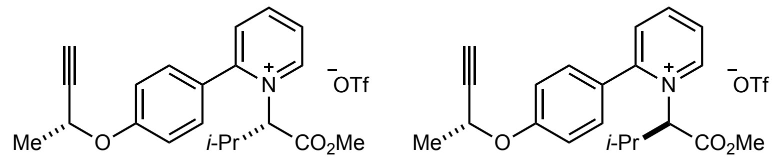

1.9 : 1

Prepared according to general procedure C using *N*-((1*Z*,2*E*,4*E*)-1-(4-(((*R*)-But-3-yn-2-yl)oxy)phenyl)-5-(dibenzylamino)penta-2,4-dien-1-ylidene)-1,1,1-trifluoromethanesulfonamide (**1s**) (221 mg, 0.400 mmol), MeOH (2.00 mL, 0.2 M), potassium metabisulfite (88.9 mg, 0.400 mmol), methyl *L*-valinate hydrochloride (**2aq**) (91.6 mg, 0.600 mmol), and heated to 70 ºC for 18 h. 1,3,5-Trimethylbenzene (56.0 µL, 0.400 mmol, 1.0 equiv.) was added as the internal standard for ^{1}H NMR analysis (84% yield, 1.9:1 d.r.). Isolated according to general isolation procedure C1 using NaOTf (413 mg, 2.40 mmol), CH_2Cl_2 (30 mL), H_2O washes (3 x 15 mL), CH_2Cl_2 extractions (2 x 10 mL), sat. Na_2CO_3 wash (1 x 20 mL) and 2:1 hexanes/Et_2O (50 mL) to afford the title compound as a mixture of diastereomers (2.1:1 d.r.) as an orange oil (145 mg, 0.303 mmol, 76% yield). IR ν_{max}/cm^{-1} (film): 3083, 2969, 1745, 1622, 1489, 1256, 1151, 1029, 936, 635; ^{1}H NMR (major and minor, 400 MHz, CD_3CN) δ: 9.11 – 9.04 (m, 1H), 8.62 – 8.53 (m, 1H), 8.17 – 8.08 (m, 1H), 8.03 – 7.96 (m, 1H), 7.50 – 7.45 (m, 2H), 7.28 – 7.24 (m, 2H), 5.19 – 5.06 (m, 1H), 5.06 – 5.04 (m, 1H), 3.84-3.83 (m, 3H), 2.90 (minor, d, *J* = 2.0 Hz, 1H), 2.88 (major, d, *J* = 2.0 Hz, 1H), 2.65 – 2.52 (m, 1H), 1.68 – 1.67 (m, 3H), 0.97 – 0.95 (m, 3H), 0.67 – 0.60 (m, 3H); ^{13}C NMR (major and minor, 100 MHz, CD_3CN) δ: 168.58, 168.56, 160.72 (major and minor), 157.91 (major and minor), 147.34 (major and minor), 144.54 (major and minor), 132.11 (major and minor), 128.28 (major and minor), 124.66, 124.63, 122.11 (major and minor, q, *J* = 320.9 Hz), 117.30 (major and minor), 83.08, 83.02, 75.97 (major and minor), 73.73 (major and minor), 64.71, 64.68, 54.46 (major and minor), 34.51, 34.47, 22.39, 22.37, 19.06, 18.58; ^{19}F NMR (major and minor, 375 MHz, CD_3CN) δ: –79.30 (major and minor); *m/z* LRMS (ESI + APCI) found $[M – OTf]^{+}$ 338.1, $C_{21}H_{24}NO_3^{+}$ requires 338.18.

7. Mechanism Studies

7.1. Examination of Reaction Additive Effects in Pyridinium Formation

- We first studied the effect of potassium metabisulfite independently from the byproducts that are generated during the pyridine ring-opening process (eq. S1). We observed that these byproducts negatively impact the pyridinium formation using isopropylamine **2aw** and amino alcohol **2b** with isolated Zincke imine **1a** in EtOAc (Table S4, entries 1–5). Interestingly, using potassium metabisulfite in EtOAc negatively impacts pyridinium **3h** formation, however, the yield improved with the combination of the ring-opening byproducts and metabisulfite (entries 6–8). Switching the solvent to just MeOH improves the reactivity of potassium metabisulfite in the reaction without needing the ring-opening byproducts (entry 9). This experiment was validated in Table S5 using amine **2a** and isolated Zincke imine **1a** to reproduce the yields observed in the one-pot pyridinium formation (Table S1) using potassium metabisulfite.

Byproducts generated during the ring-opening of pyridines (S1)

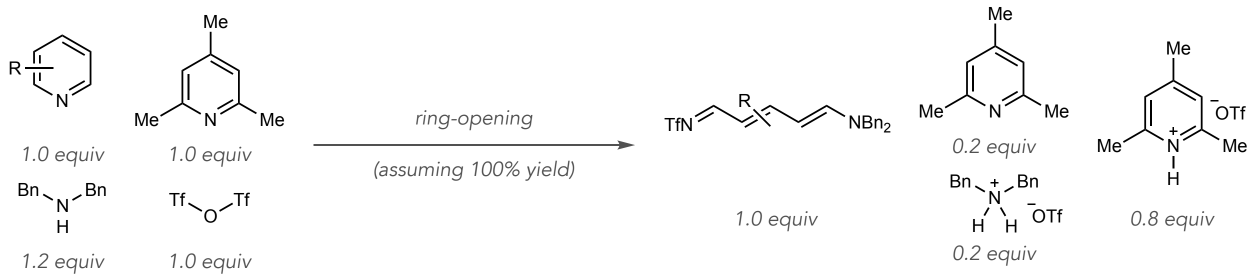

Table S4. Examining the effects of the ring-opening byproducts on pyridinium salt formation with and without potassium metabisulfite using isolated 1a and amines 2aw and 2b.[a]

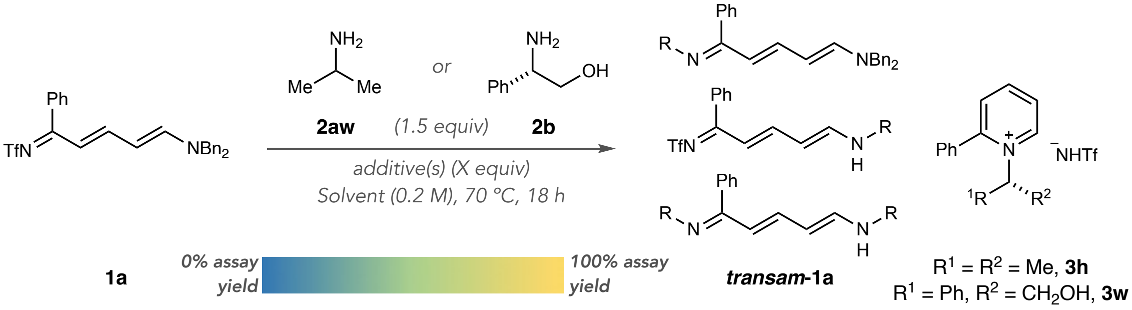

entry	additives (equiv)	solvent	using 2aw: % 1a + *trans-aminated*-1a	using 2aw: % 3h	using 2b: % 1a + *trans-aminated*-1a	using 2b: % 3w
1	none	EtOAc	0	**78**	0	**48**
2	$K_2S_2O_5$ (1)	EtOAc	0	**40**	0	**61**
3	Collidinium•OTf (1)	EtOAc	0	**74**	15	**44**
4	Bn_2NH_2•OTf (1)	EtOAc	0	**59**	26	**36**
5	Collidine (1), Bn_2NH (0.2) TfOH (1)	EtOAc	0	**67**	20	**42**
6	Collidinium•OTf (1), $K_2S_2O_5$ (1)	EtOAc	0	**92**	24	**85**
7	Bn_2NH_2•OTf (1), $K_2S_2O_5$ (1)	EtOAc	0	**94**	12	**79**
8	Collidine (1), Bn_2NH (0.2) TfOH (1), $K_2S_2O_5$ (1)	EtOAc	0	**99**	8	**76**
9	$K_2S_2O_5$	MeOH	0	**98**	0	**72**

[a]Reactions run using 0.1 mmol of **1a**. Yields determined by ^{1}H NMR using 1,3,5-trimethoxybenzene as an internal standard in CD_3OD.

Table S5. Validating reaction conditions in MeOH using isolated Zincke imine 1a and amine 2a.[a]

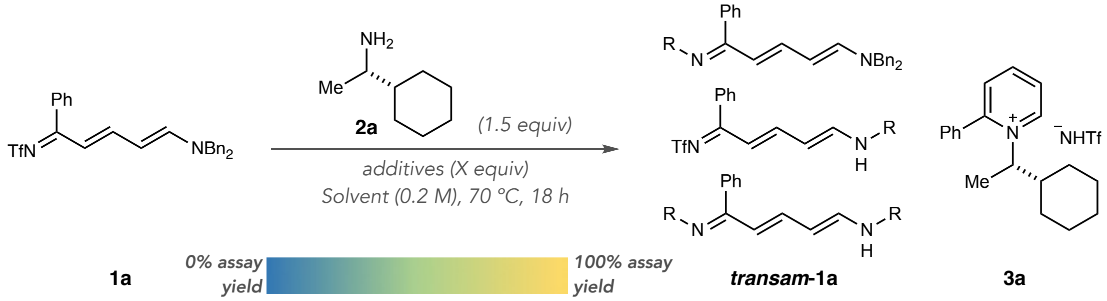

entry	additives (equiv)	solvent	% 1a + *transam*-1a	% 3a
1	none	EtOAc	0	**80**
2	$K_2S_2O_5$	EtOAc	0	**37**
3	$NaHSO_3$	EtOAc	0	**17**
3	Collidine (1), TfOH (1)	EtOAc	41	**5**
4	Collidine (1), Bn_2NH (0.2) TfOH (1), $K_2S_2O_5$ (1)	EtOAc	0	**91**
5	none	MeOH	73	**5**
6	$K_2S_2O_5$	MeOH	0	**98**
7	$NaHSO_3$	MeOH	0	**100**

[a]Reactions run using 0.1 mmol of **1a**. Yields determined by ^1H NMR using 1,3,5-trimethoxybenzene as an internal standard in CD_3OD.

- In Table S6, we next compared the reaction outcome for other amines between the conditions in EtOAc without an additive and the conditions in MeOH with $K_2S_2O_5$ (comparing conditions from Table S4 entries 1 & 9).

Table S6. Comparison of optimal reaction conditions using Zincke imine 1a and amines 2a–2d.[a]

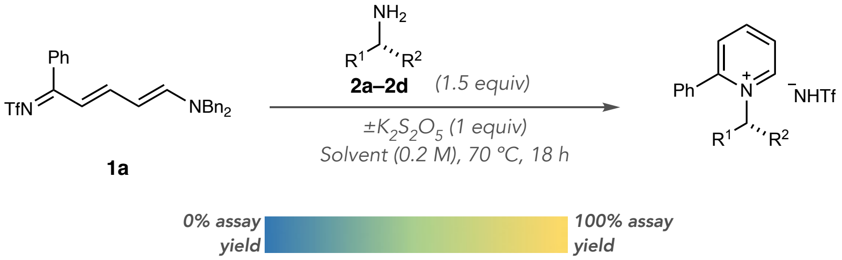

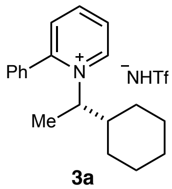

3a

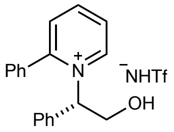

3w

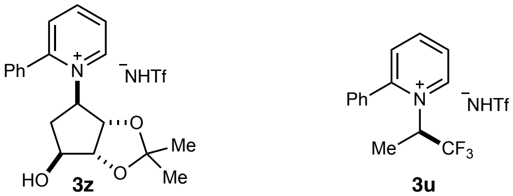

3z **3u**

	3a	3a	3w	3w	3z	3z	3u	3u
conditions	no additive EtOAc	$K_2S_2O_5$ MeOH	no additive EtOAc	$K_2S_2O_5$ MeOH	no additive EtOAc	$K_2S_2O_5$ MeOH	no additive EtOAc	$K_2S_2O_5$ MeOH
yield	**80**	**98**	**44**	**72**	**65**	**97**	**0**	**62**

[a]Reactions run using 0.1 mmol of **1a**. Yields determined by ^{1}H NMR using 1,3,5-trimethoxybenzene as an internal standard in CD_3OD.

- We then revisited the effect of other reaction additives on pyridinium **3a** formation independently from the ring-opening byproducts discussed above.

Table S7. Examining acidic, basic, and nucleophilic additives in pyridinium 3a formation from isolated Zincke imine 1a.[a]

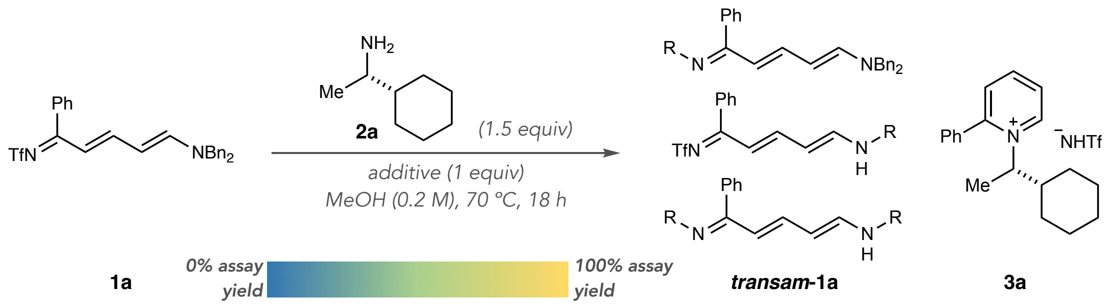

entry	additive (1 equiv)	% 1a + *transam*-1a	% 3a
1	none	73	5
2	$K_2S_2O_5$	0	**98**
3	$NaHSO_3$	0	**100**
4	AcOH	50	8
5	PPTs	43	4
6	$(OEt)_2PS_2H$	59	3
7	4-nitrophenol	49	7
8	2-Br-phenol	42	9
9	4-nitrothiophenol	34	14
10	4-Cl-thiophenol	18	**40**
11	2-hydroxypyridine	70	5
12	$PhSO_2Na$	85	5
13	pyridine *N*-oxide	80	7
14	DABCO	38	9
15	DMAP	87	10
16	$K_2H_2PO_4$	35	16
17	$NaHCO_3$	55	5
18	phenol	50	5
19	K_2SO_3	5	**84**

[a]Reactions run using 0.1 mmol of **1a**. Yields determined by ^{1}H NMR using 1,3,5-trimethoxybenzene as an internal standard in CD_3OD.

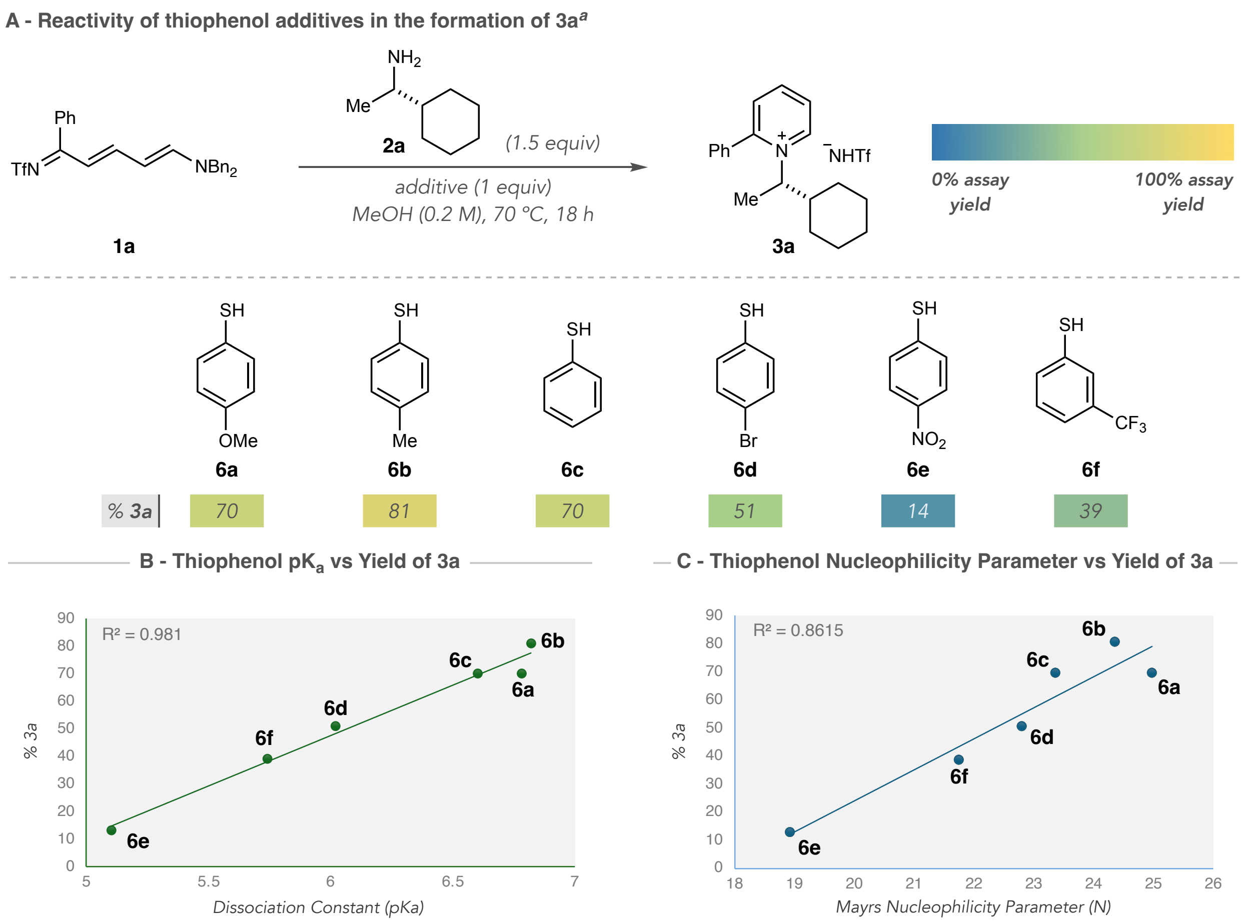

Figure S3. (A) Examination of thiophenol additives in the cyclization of Zincke imine **1a** with **2a**. (B) Correlation of thiophenol pK_a and yield of pyridinium salt **3a**. (C) Correlation of thiophenol nucleophilicity parameter and yield of pyridinium salt **3a**.[4,5] [a]Reactions run using 0.1 mmol of **1a**. Yields determined by ^1H NMR using 1,3,5-trimethoxybenzene as an internal standard in CD_3OD.

7.2. Sub-stoichiometric Metabisulfite Loading

- Investigation of sub-stoichiometric potassium metabisulfite loading on pyridinium formation.

Table S8. Sub-stoichiometric loading of potassium metabisulfite in the cyclization of Zincke imine 1a with amines 1a–1d.[a]

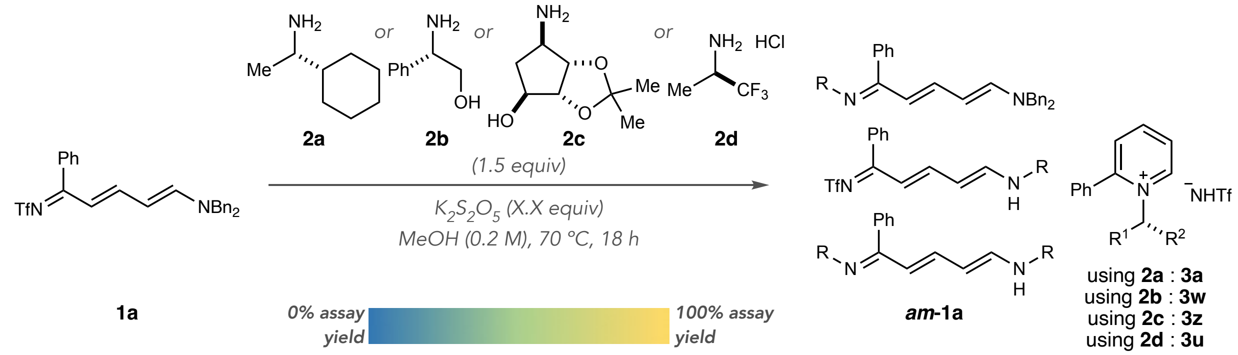

entry	$K_2S_2O_5$ equiv.	using 2a		using 2b		using 2c		using 2d	
		% **1a** + *am*-**1a**	% **3a**	% **1a** + *am*-**1a**	% **3w**	% **1a** + *am*-**1a**	% **3z**	% **1a** + *am*-**1a**	% **3u**
1	4.0	0	**101**	0	**65**	0	**99**	0	**51**
2	2.0	0	**102**	0	**64**	0	**99**	0	**54**
3	1.0	0	**104**	0	**72**	0	**102**	0	**68**
4	0.75	0	**105**	0	**74**	0	**96**	0	**67**
5	0.50	0	**102**	0	**75**	0	**83**	34	**58**
6	0.25	0	**100**	0	**68**	0	**87**	56	**15**
7	0.20	0	**95**	0	**60**	0	**87**	56	**7**
8	0.15	0	**86**	0	**57**	3	**76**	62	**4**
9	0.10	0	**86**	0	**55**	28	**58**	65	**3**
10	0.05	20	**30**	20	**52**	33	**53**	66	**2**
11	none	73	**5**	73	**42**	48	**25**	71	**2**

[a]Reactions run using 0.1 mmol of **1a**. Yields determined by ^{1}H NMR using 1,3,5-trimethoxybenzene as an internal standard in CD_3OD.

7.3. Hypotheses and Control Experiments

TEMPO Reaction Inhibition:

- Testing the hypothesis that potassium metabisulfite is involved in a radical mechanism for pyridinium formation.

Table S9. TEMPO inhibition screen for cyclization of Zincke imine 1a with 2a and 2aw using potassium metabisulfite or sodium bisulfite.[a]

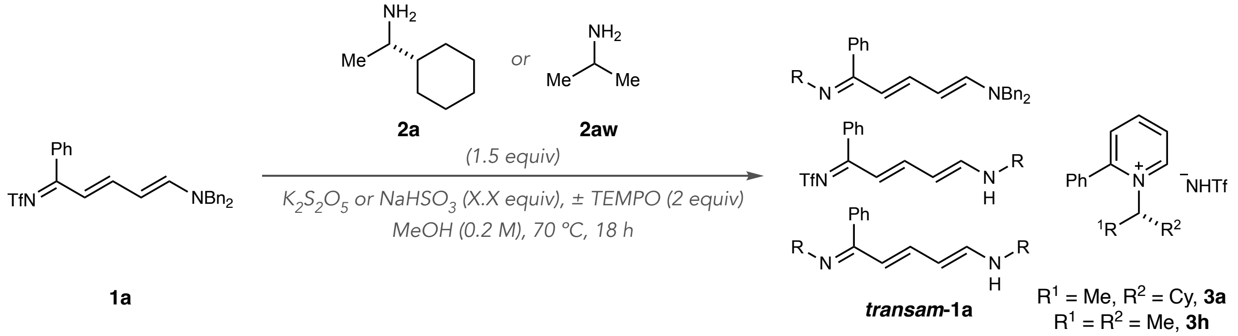

			using 2a		using 2aw	
entry	$K_2S_2O_5$ or $NaHSO_3$ (equiv.)	TEMPO (Y/N)	% **1a** +***transam*-1a**	% **3a**	% **1a** +***transam*-1a**	% **3h**
1	$K_2S_2O_5$ (1 equiv)	N	0	**98**	0	**98**
2	$K_2S_2O_5$ (1 equiv)	Y	0	**88**	0	**86**
3	$NaHSO_3$ (2 equiv)	N	0	**101**	0	**97**
4	$NaHSO_3$ (2 equiv)	Y	0	**90**	0	**92**

[a]Reactions run using 0.1 mmol of **1a**. Yields determined by ^{1}H NMR using 1,3,5-trimethoxybenzene as an internal standard in CD_3OD.

Pyridinium Salt Stability:

- Testing the hypothesis that potassium metabisulfite stabilizes the pyridinium salt product from decomposition under the reaction conditions.

Scheme S1. Stability experiment with pyridinium salt 3a with and without potassium metabisulfite under the reaction conditions for forming 3a.[a]

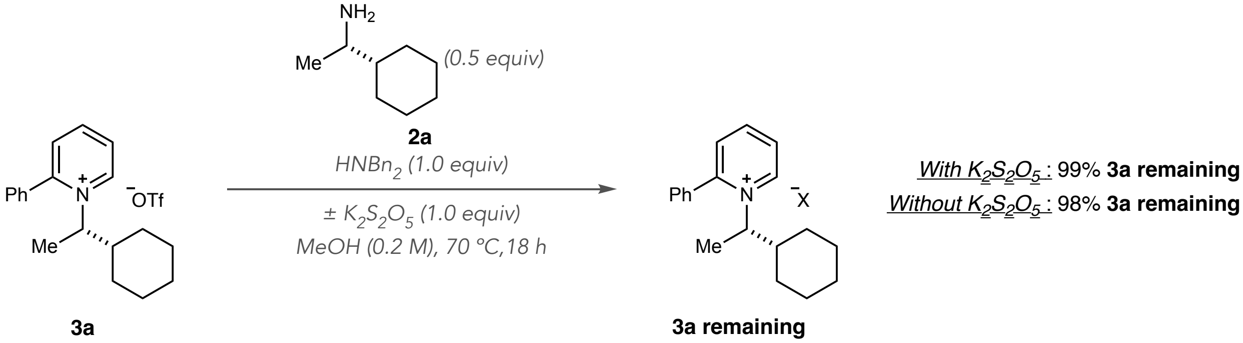

[a]Reactions run using 0.1 mmol of isolated **3a**. Yields determined by ^{1}H NMR using 1,3,5-trimethoxybenzene as an internal standard in CD_3OD.

Air Sensitivity:

- Testing the hypothesis that potassium metabisulfite prevents an oxidative decomposition pathway under the reaction conditions.

Scheme S2. Recyclization of Zincke imine 1a with 2b and potassium metabisulfite under an air atmosphere and nitrogen atmosphere.[a]

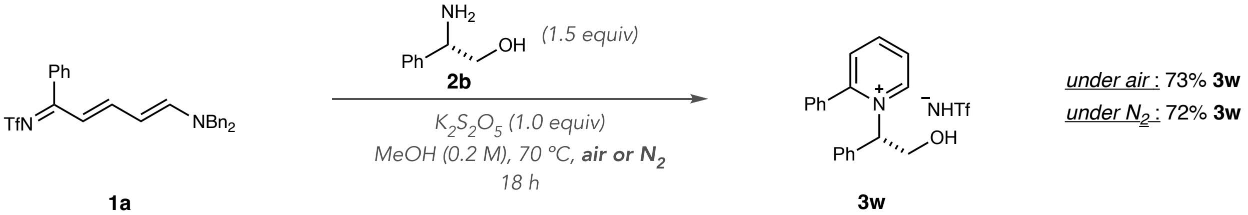

[a]Reactions run using 0.1 mmol of **1a**. Yields determined by ^{1}H NMR using 1,3,5-trimethoxybenzene as an internal standard in CD_3OD. Reaction under nitrogen atmosphere set up inside a glovebox with degassed MeOH.

Sulfur Dioxide Formation and Mechanistic Role:

- Testing the hypothesis that sulfur dioxide is formed from metabisulfite and involved in the reaction mechanism.

Scheme S3. Recyclization of Zincke imine 1a with 2a and potassium metabisulfite in an open system (reflux condenser) and closed system (sealed vial).[a]

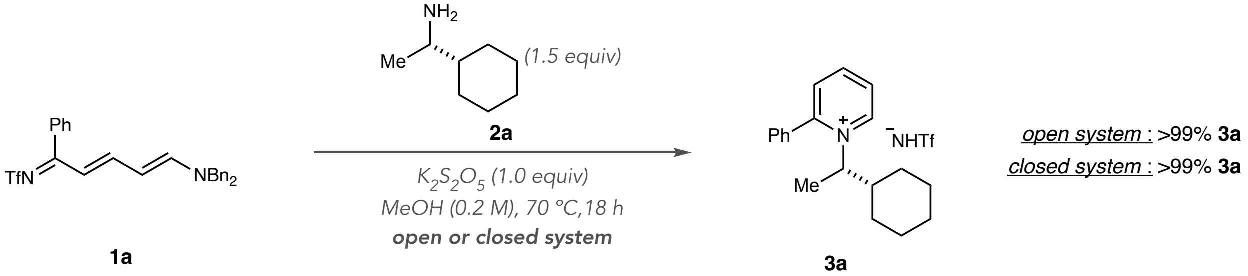

[a]Reactions run using 1.0 mmol of **1a**. Yields determined by ^{1}H NMR using 1,3,5-trimethoxybenzene as an internal standard in CD_3OD. Reaction in open system ran in a 25 mL round-bottom flask equipped with a reflux condenser, and reaction in closed system ran in a sealed 16 mL vial.

*Zincke Imine **1a** Reactivity with Bisulfite Control Experiment:*

- Examining whether bisulfite forms deconjugated Zincke imine intermediates that are observable by ^{1}H NMR. Only the starting Zincke imine was observed in the crude reaction after reacting at 70 ºC and room temperature for 48 h.

Scheme S4. Reactivity of Zincke imine 1a with potassium metabisulfite at room temperature.[a]

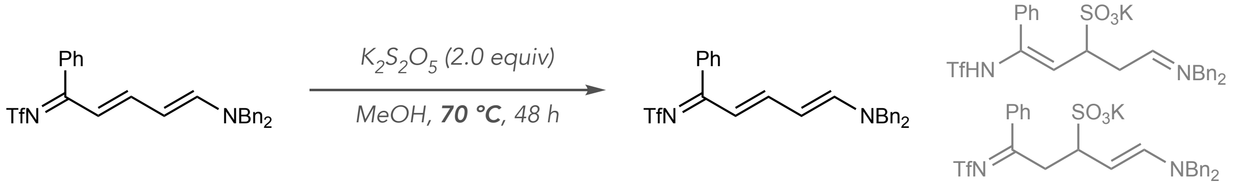

1a ***11% remaining*** ***Not Observed***

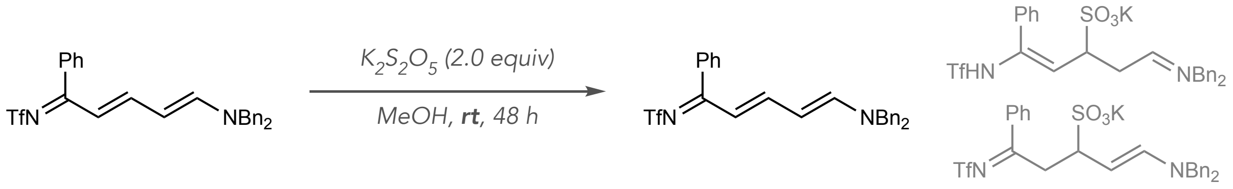

1a ***98% remaining*** ***Not Observed***

[a]Reaction run using 0.1 mmol of **1a**. Yields determined by ^{1}H NMR using 1,3,5-trimethoxybenzene as an internal standard in CD_3OD.

7.4. Deuterium Labeling Experiments

Procedure for Deuterium Labeling Experiments:

An oven dried 16 mL vial equipped with a stir bar was charged with *N*-((1*Z*,2*E*,4*E*)-5-(dibenzylamino)-1-phenylpenta-2,4-dien-1-ylidene)-1,1,1-trifluoromethanesulfonamide (**1a**) (1.0 equiv), CD_3OD (0.2 M), 1,3,5-trimethoxybenzene (1.0 equiv), and reaction additive (2.0 equiv). The reaction vial was capped and heated to 70 ºC for 2 h. The reactions were cooled to room temperature, and a direct aliquot was taken from each reaction and diluted with CD_3OD for ^{1}H NMR analysis of the crude reaction. The vials were again heated to 70 ºC for an additional 22 h before taking another direct aliquot for ^{1}H NMR analysis.

Results:

Table S10. Deuterium incorporation study with different reaction additives using Zincke imine 1a in CD_3OD.[a]

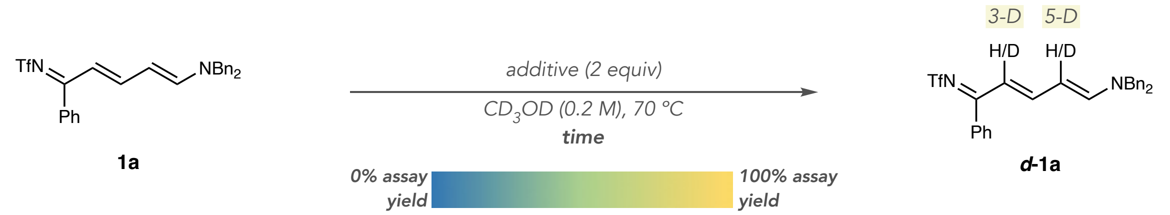

entry	additive (2 equiv)	after 2 h			after 24 h		
		% **1a+*d*-1a**	% **3-D**	% **5-D**	% **1a+*d*-1a**	% **3-D**	% **5-D**
1	none	106	0	0	96	3	22
2	$K_2S_2O_5$	81	22	100	10	100	100
3	$NaHSO_3$	80	15	100	47	81	100
4	AcOH	97	10	9	85	62	56
5	PPTs	39	97	95	0	N/A	N/A
6	4-Cl-thiophenol	85	35	98	84	45	100
7[b]	4-OMe-thiopenol (**6a**)	61	16	100	-	-	-
8[b]	4-Me-thiophenol (**6b**)	71	45	99	-	-	-
9[b]	thiophenol (**6c**)	74	45	97	-	-	-
10[b]	4-Br-thiophenol (**6d**)	86	52	97	-	-	-
11[b]	4-NO_2-thiophenol (**6e**)	30	97	97	-	-	-
12[b]	3-CF_3-thiophenol (**6f**)	101	52	98	-	-	-

[a]Reactions run using 0.1 mmol of **1a**. Yields determined by ^1H NMR using 1,3,5-trimethoxybenzene as an internal standard in CD_3OD. [b]Reactions stopped after 2 hours. Dashes indicate the reaction was not run

Deuterium Incorporation Assays (^{1}H NMR Spectra):

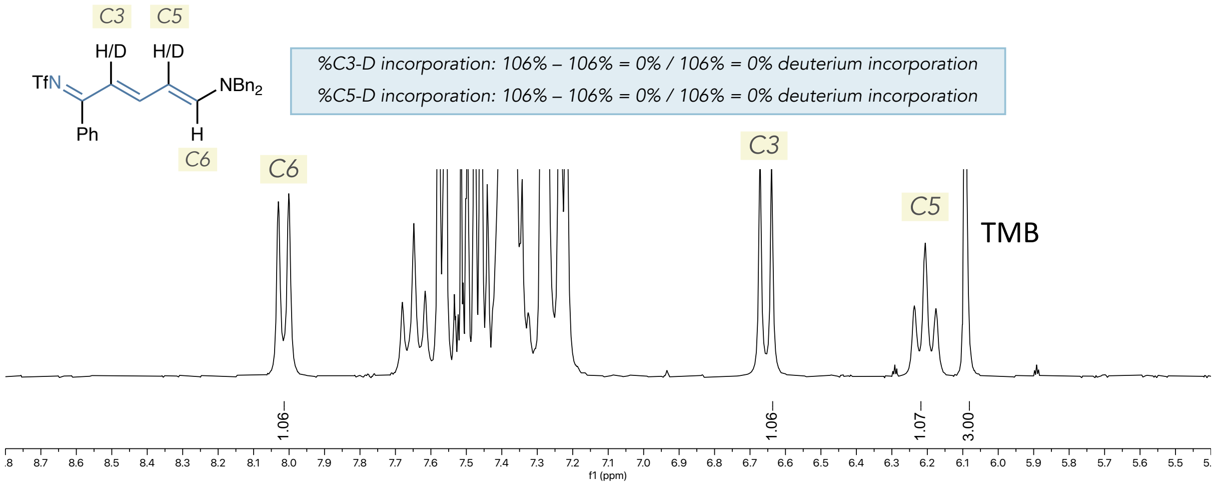

Figure S4. ^{1}H NMR spectrum (in CD_3OD) and reaction analysis for deuterium incorporation of Zincke imine **1a** without additives at 70 ºC after 2 h (entry 1 in Table S10).

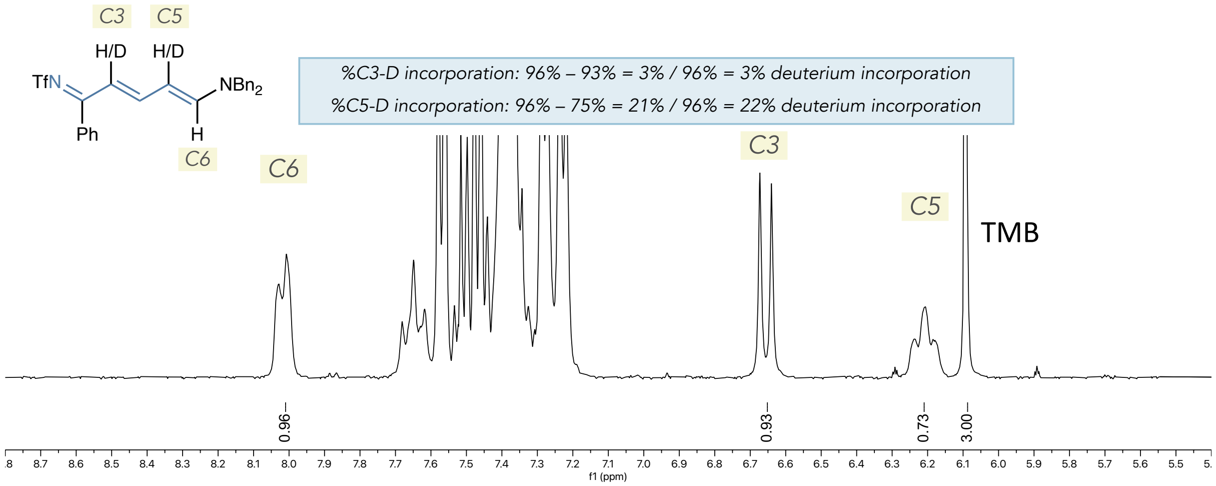

Figure S5. ^{1}H NMR spectrum (in CD_3OD) and reaction analysis for deuterium incorporation of Zincke imine **1a** without additives at 70 ºC after 24 h (entry 1 in Table S10).

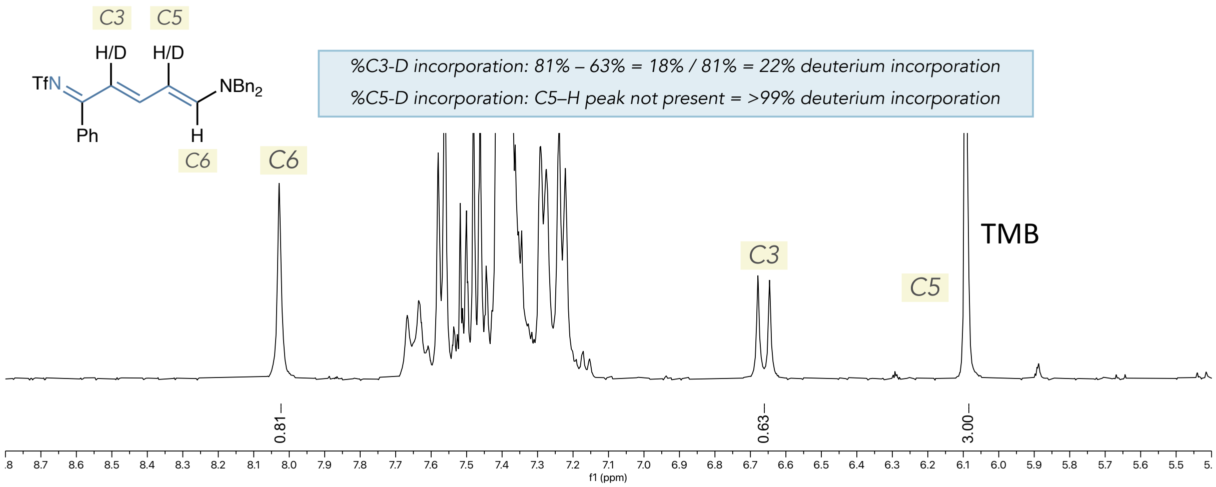

Figure S6. ^{1}H NMR spectrum (in CD_3OD) and reaction analysis for deuterium incorporation of Zincke imine **1a** using $K_2S_2O_5$ at 70 ºC after 2 h (entry 2 in Table S10).

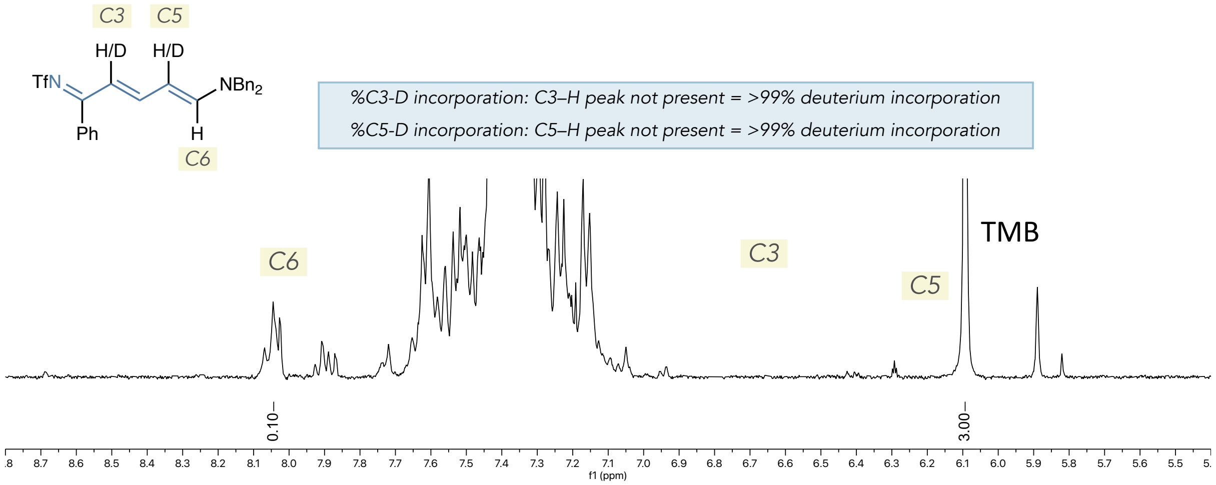

Figure S7. ^{1}H NMR spectrum (in CD_3OD) and reaction analysis for deuterium incorporation of Zincke imine **1a** using $K_2S_2O_5$ at 70 ºC after 24 h (entry 2 in Table S10).

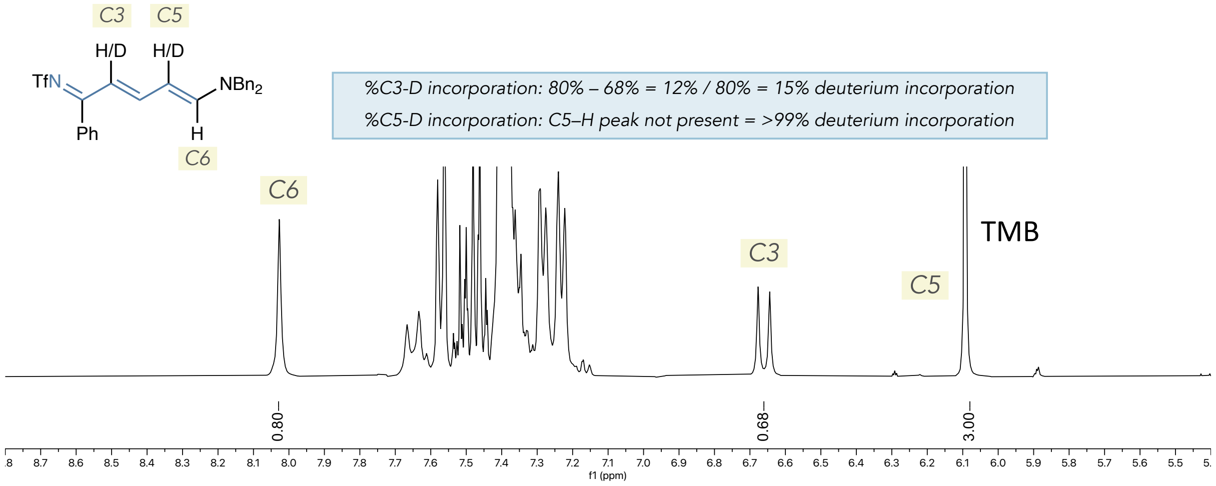

Figure S8. ^{1}H NMR spectrum (in CD_3OD) and reaction analysis for deuterium incorporation of Zincke imine **1a** using $NaHSO_3$ at 70 ºC after 2 h (entry 3 in Table S10).

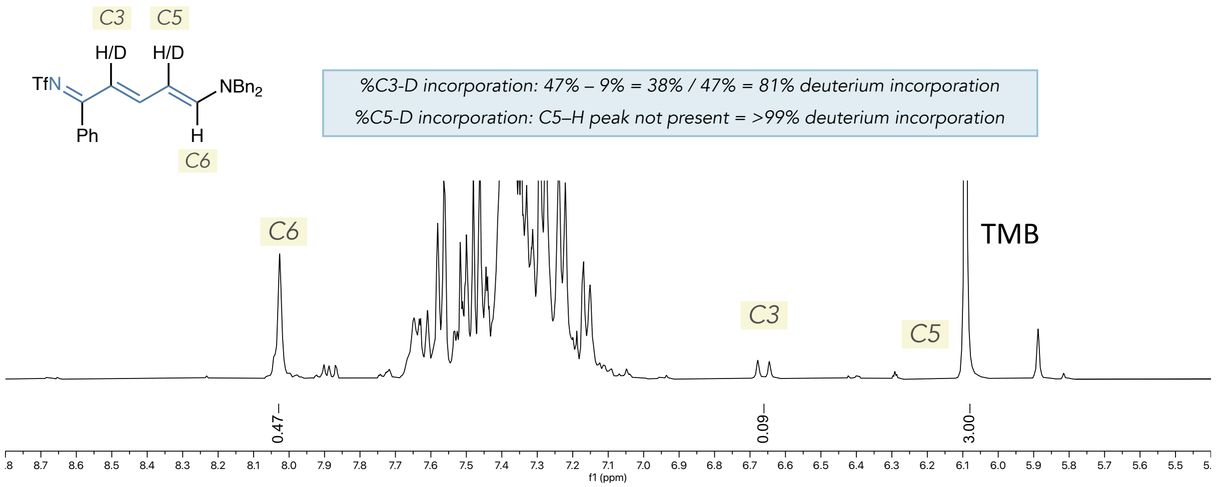

Figure S9. ^{1}H NMR spectrum (in CD_3OD) and reaction analysis for deuterium incorporation of Zincke imine **1a** using $NaHSO_3$ at 70 ºC after 24 h (entry 3 in Table S10).

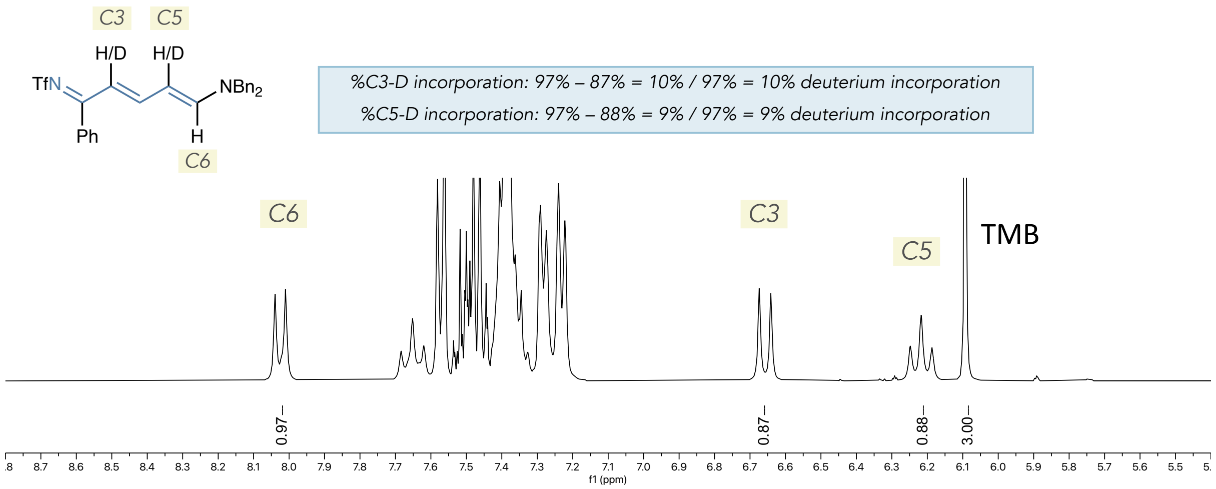

Figure S10. ^{1}H NMR spectrum (in CD_3OD) and reaction analysis for deuterium incorporation of Zincke imine **1a** using AcOH at 70 ºC after 2 h (entry 4 in Table S10).

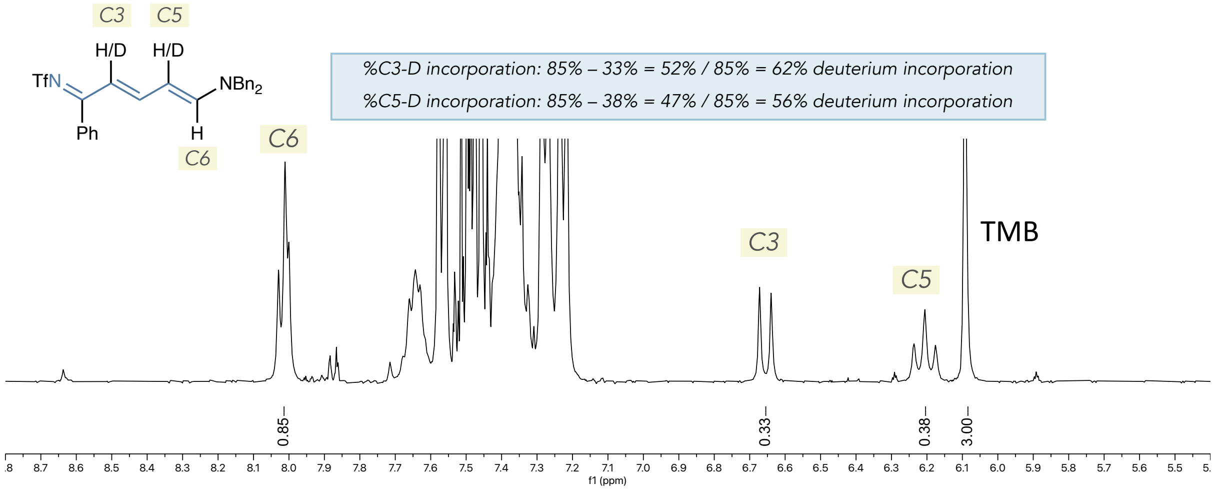

Figure S11. ^{1}H NMR spectrum (in CD_3OD) and reaction analysis for deuterium incorporation of Zincke imine **1a** using AcOH at 70 ºC after 24 h (entry 4 in Table S10).

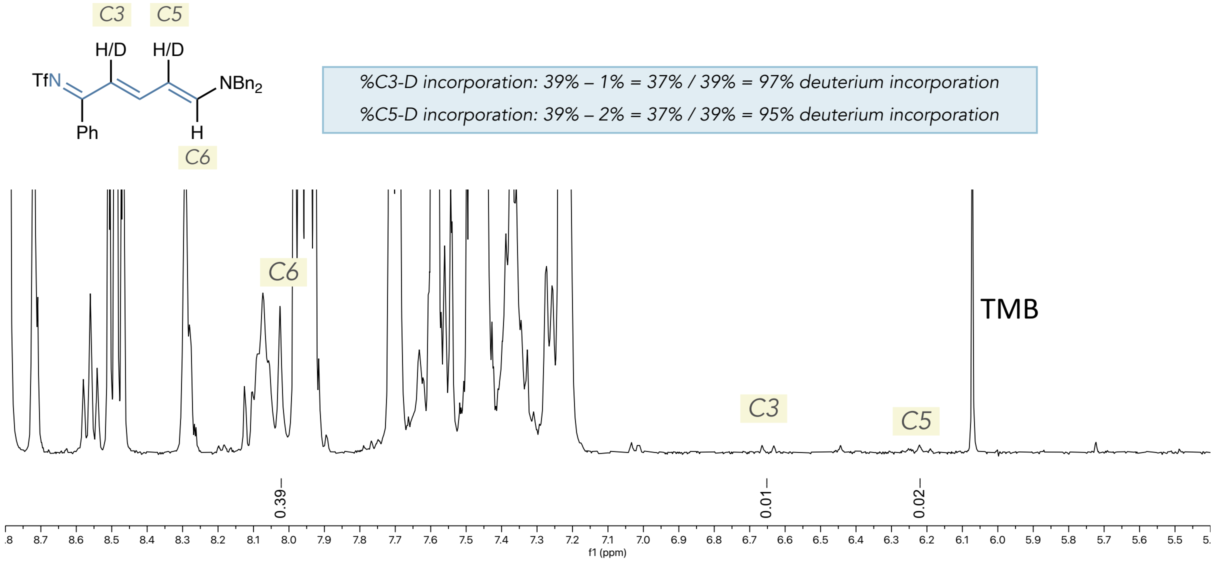

Figure S12. ^{1}H NMR spectrum (in CD_3OD) and reaction analysis for deuterium incorporation of Zincke imine **1a** using PPTS at 70 ºC after 2 h (entry 5 in Table S10). [a]Labeling of the aromatic C-H protons of TMB was observed under the reaction conditions, so the sp^3 methyl groups were used as the internal standard reference.

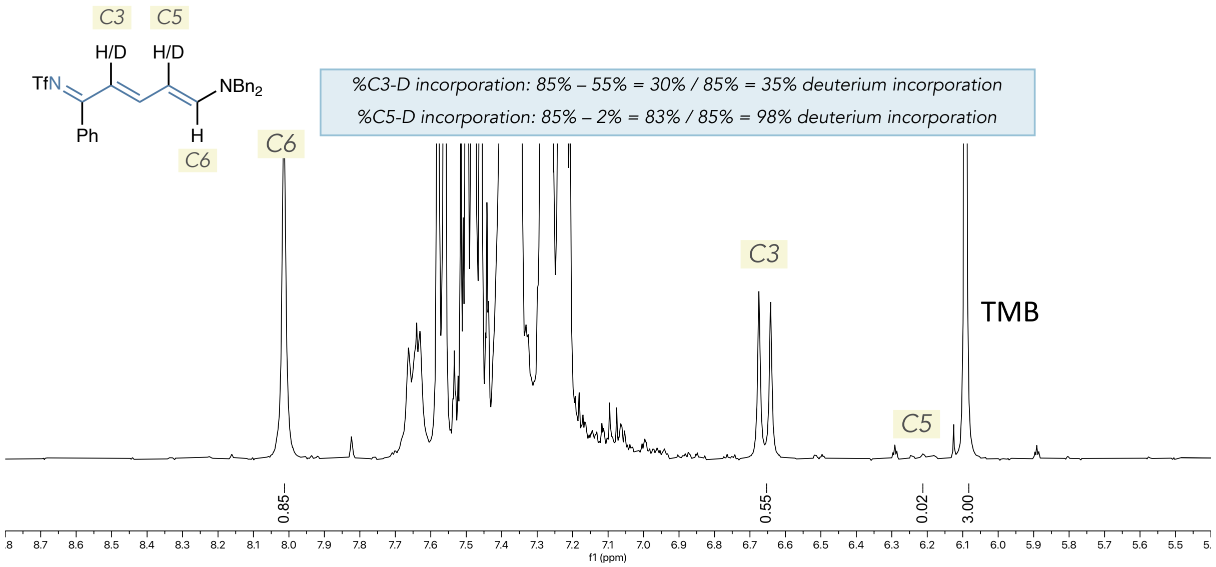

Figure S13. ^{1}H NMR spectrum (in CD_3OD) and reaction analysis for deuterium incorporation of Zincke imine **1a** using 4-Cl-thiophenol at 70 ºC after 2 h (entry 6 in Table S10).

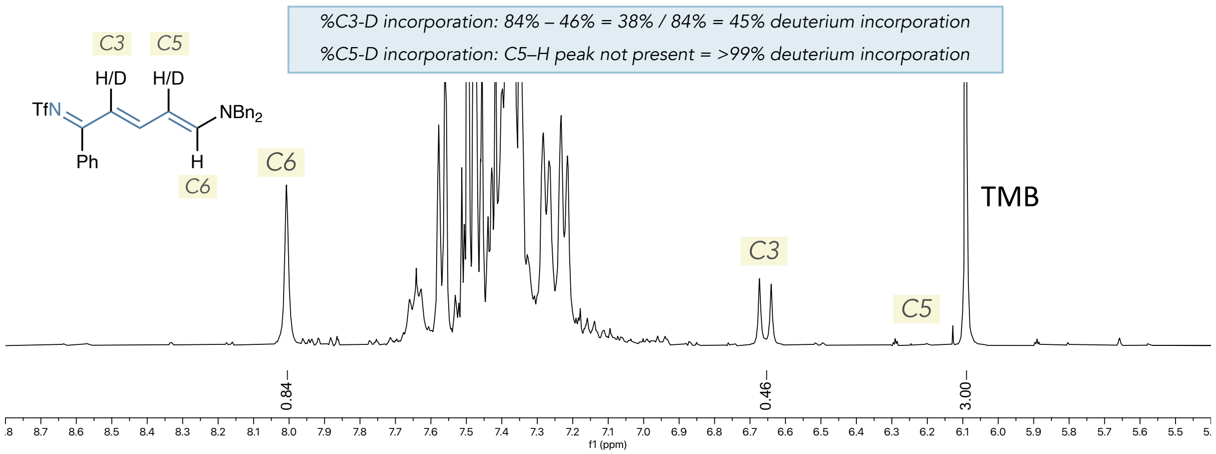

Figure S14. ^{1}H NMR spectrum (in CD_3OD) and reaction analysis for deuterium incorporation of Zincke imine **1a** using 4-Cl-thiophenol at 70 ºC after 24 h (entry 6 in Table S10).

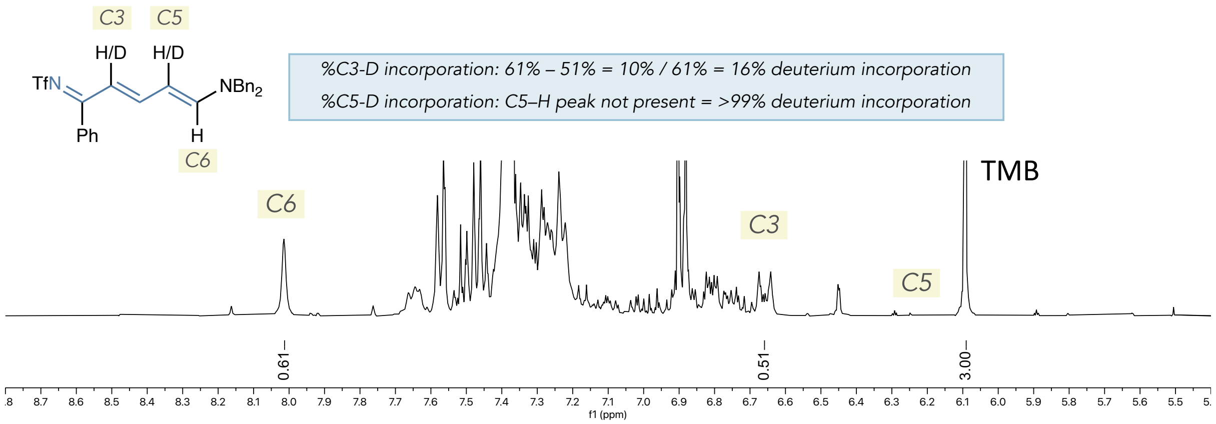

Figure S15. ^{1}H NMR spectrum (in CD_3OD) and reaction analysis for deuterium incorporation of Zincke imine **1a** using 4-OMe-thiophenol (**6a**) at 70 ºC after 2 h (entry 7 in Table S10).

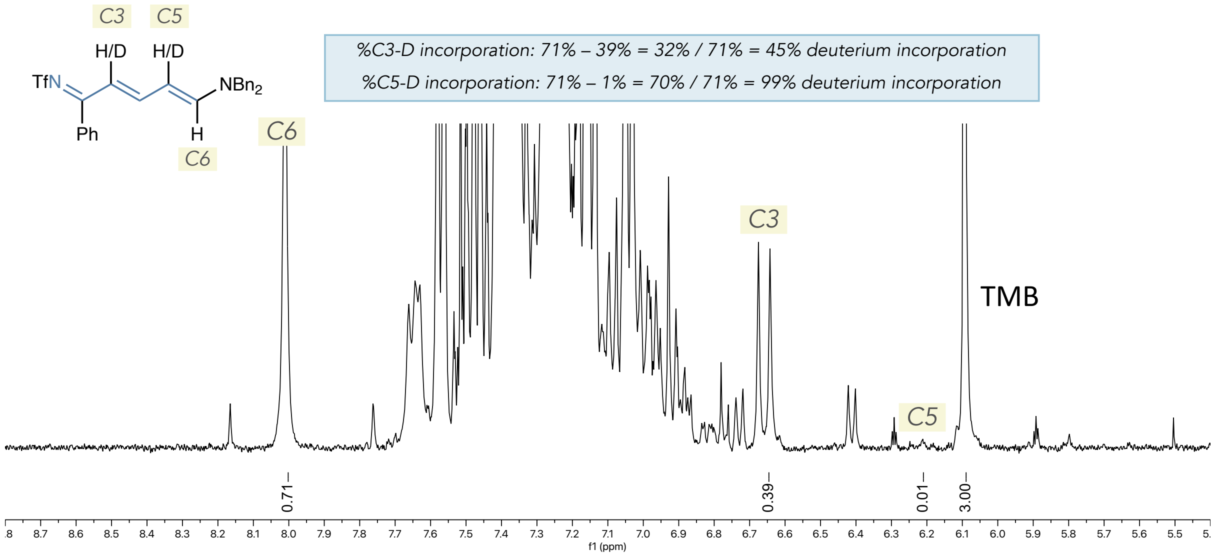

Figure S16. ^{1}H NMR spectrum (in CD_3OD) and reaction analysis for deuterium incorporation of Zincke imine **1a** using 4-Me-thiophenol (**6b**) at 70 ºC after 2 h (entry 8 in Table S10).

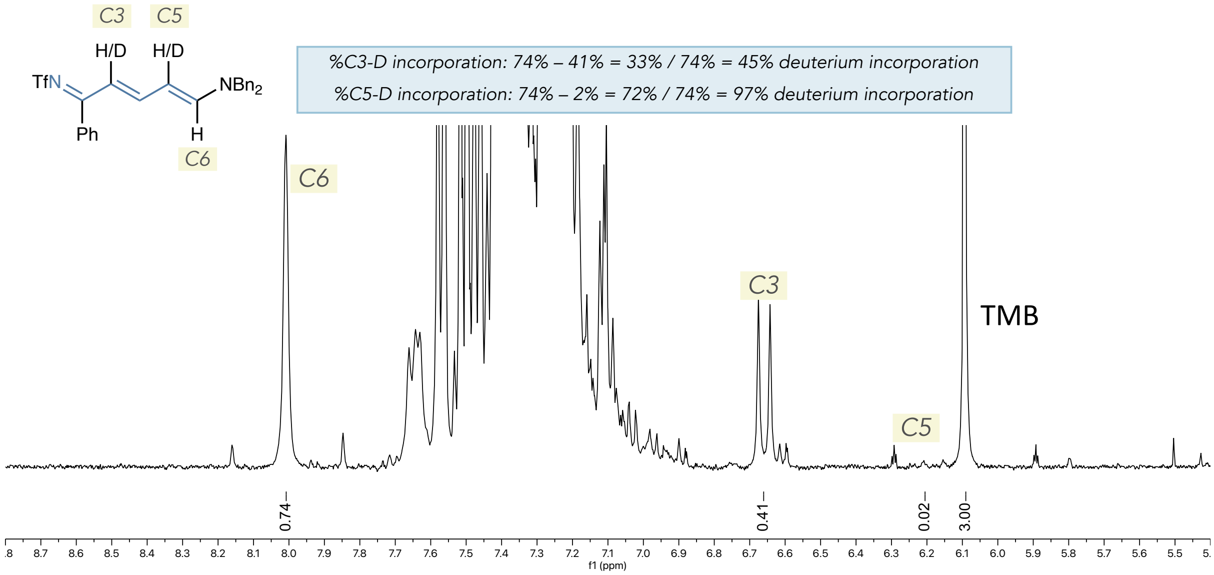

Figure S17. ^{1}H NMR spectrum (in CD_3OD) and reaction analysis for deuterium incorporation of Zincke imine **1a** using thiophenol (**6c**) at 70 ºC after 2 h (entry 9 in Table S10).

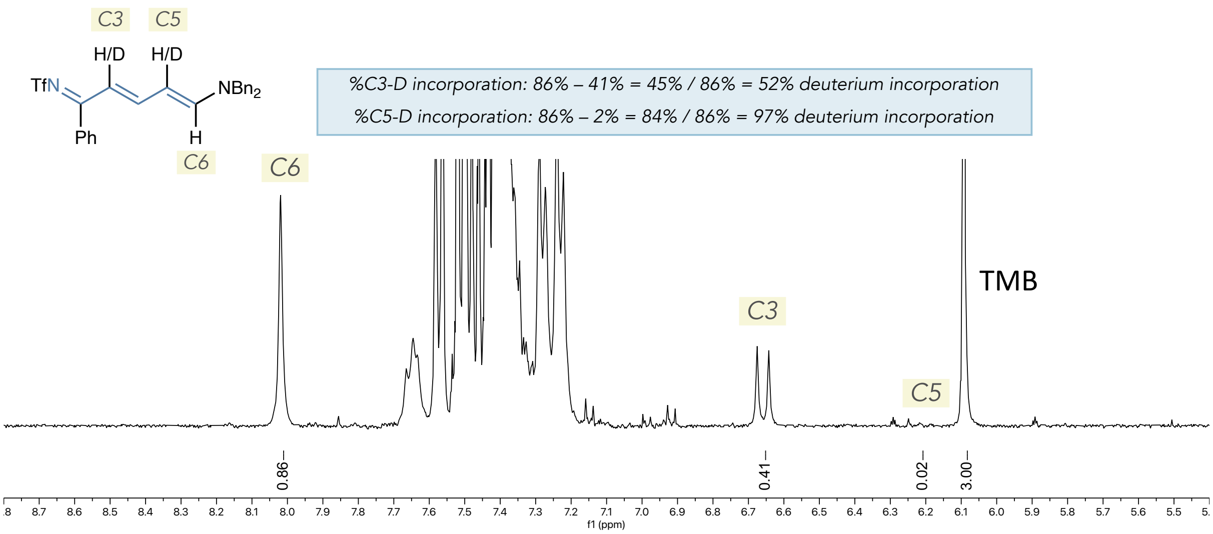

Figure S18. ^{1}H NMR spectrum (in CD_3OD) and reaction analysis for deuterium incorporation of Zincke imine **1a** using 4-Br-thiophenol (**6d**) at 70 ºC after 2 h (entry 10 in Table S10).

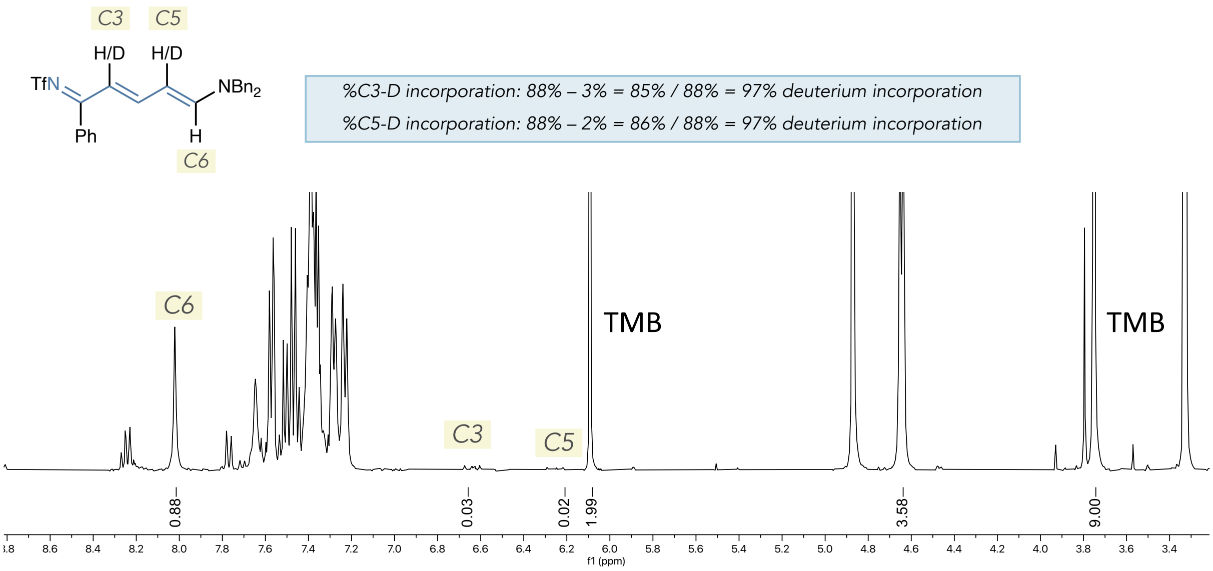

Figure S19. ^{1}H NMR spectrum (in CD_3OD) and reaction analysis for deuterium incorporation of Zincke imine **1a** using 4-NO_2-thiophenol (**6e**) at 70 ºC after 2 h (entry 11 in Table S10). [a]Labeling of the aromatic C-H protons of TMB was observed under the reaction conditions, so the sp^3 methyl groups were used as the internal standard reference.

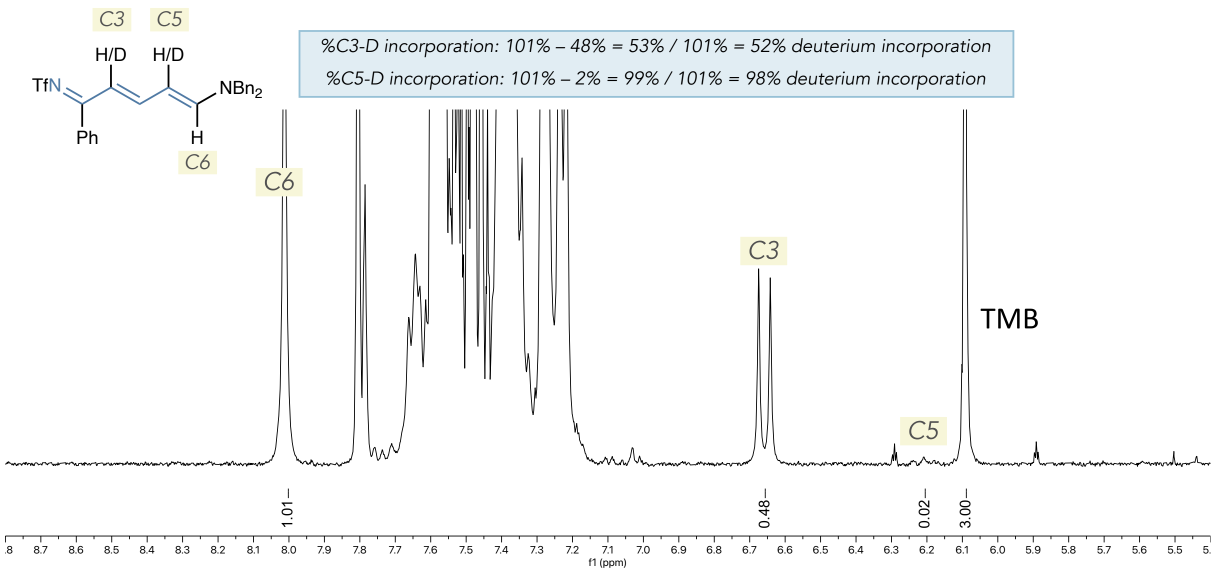

Figure S20. ^{1}H NMR spectrum (in CD_3OD) and reaction analysis for deuterium incorporation of Zincke imine **1a** using 3-CF_3-thiophenol (**6f**) at 70 ºC after 2 h (entry 12 in Table S10).

7.5. Transamination Time Study with Potassium Metabisulfite

- The transamination of Zincke imines leads to complex mixtures of mono- and bis-transaminated Zincke imines that are distinguishable from the starting material. The yields reported in Table S11 for transaminated-Zincke imines corresponds to the combined yield of the three possible species. The transamination equilibrium is affected by acid and base additives, but only potassium metabisulfite promotes the formation of **3h** at room temperature.

Table S11. Transamination time study using 1a and 2aw at room temperature with different reaction additives.[a]

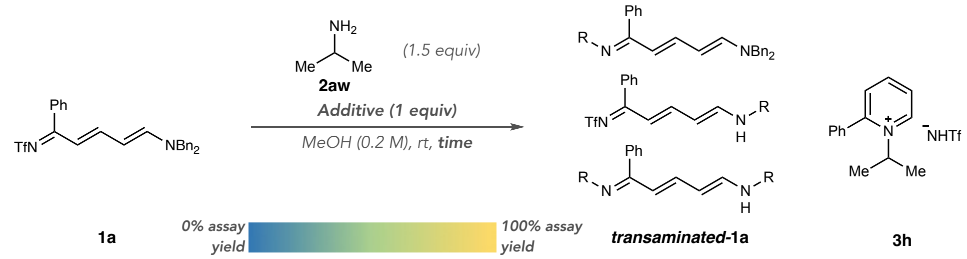

entry	Time (h)	no additive: % 1a	no additive: % *trans-aminated*-1a	no additive: % 3h	$K_2S_2O_5$: % 1a	$K_2S_2O_5$: % *trans-aminated*-1a	$K_2S_2O_5$: % 3h	AcOH[b]: % 1a	AcOH[b]: % *trans-aminated*-1a	AcOH[b]: % 3h	NEt_3: % 1a	NEt_3: % *trans-aminated*-1a	NEt_3: % 3h
1	1	28	**70**	nd	27	**68**	nd	38	**nd**	nd	14	**83**	nd
2	2	28	**67**	nd	24	**66**	2	36	**nd**	nd	11	**88**	nd
3	4	27	**73**	nd	15	**41**	6	33	**nd**	nd	10	**87**	nd
4	8	24	**76**	nd	4	**31**	21	44	**nd**	nd	8	**86**	nd
5	22	15	**81**	nd	nd	**7**	56	38	**nd**	nd	3	**90**	nd

[a]Reactions run using 0.1 mmol of **1a**. Yields determined by ^{1}H NMR using 1,3,5-trimethoxybenzene as an internal standard in CD_3OD, and the presence of transaminated-Zincke imines was validated by LRMS. [b]Zincke imine was insoluble in CD_3OD during ^{1}H NMR analysis. Transamination was not observed with AcOH.

7.6. Reaction Intermediate Characterizations and Control Experiments

*Observation of **4a** and **4b** in crude reaction mixture with ^{1}H NMR and HRMS:*

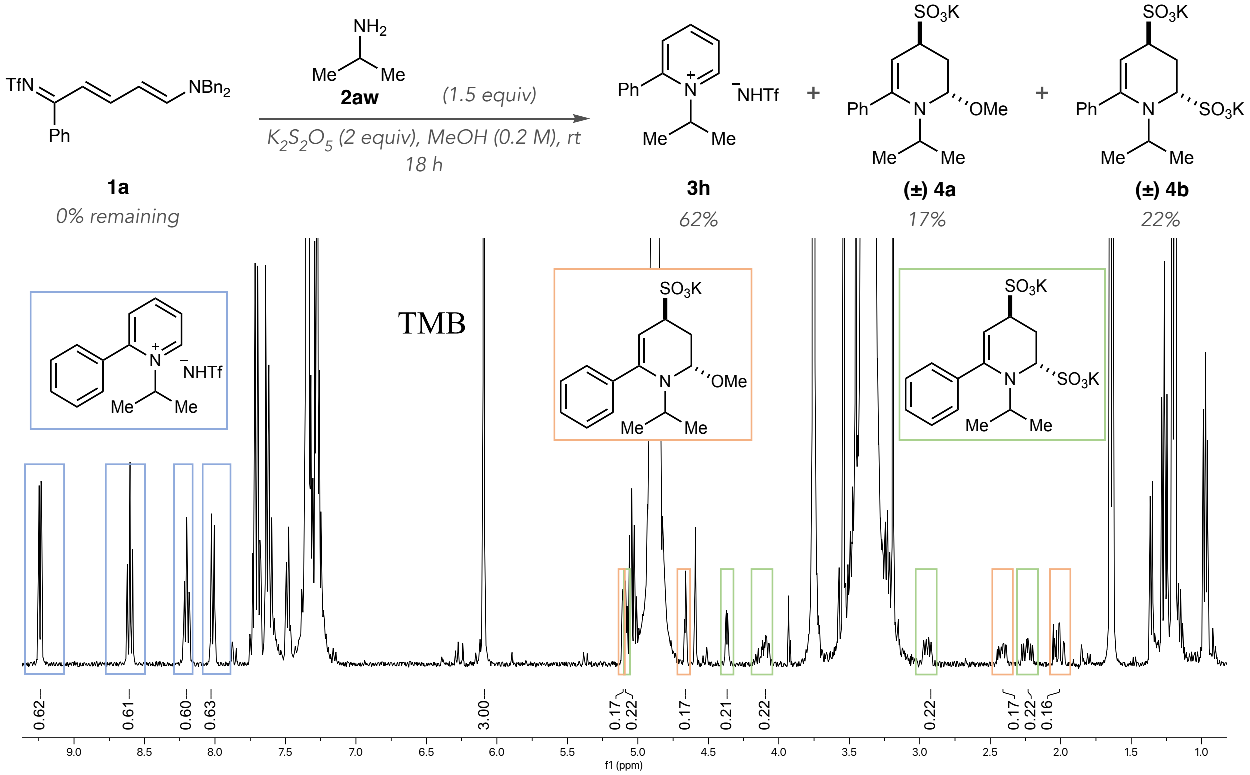

Figure S21. Crude ^{1}H NMR spectrum (CD_3OD) of the reaction between Zincke imine **1a** and *iso*-propylamine at room temperature after 18 h with 1,3,5-trimethoxybenzene as an internal standard.

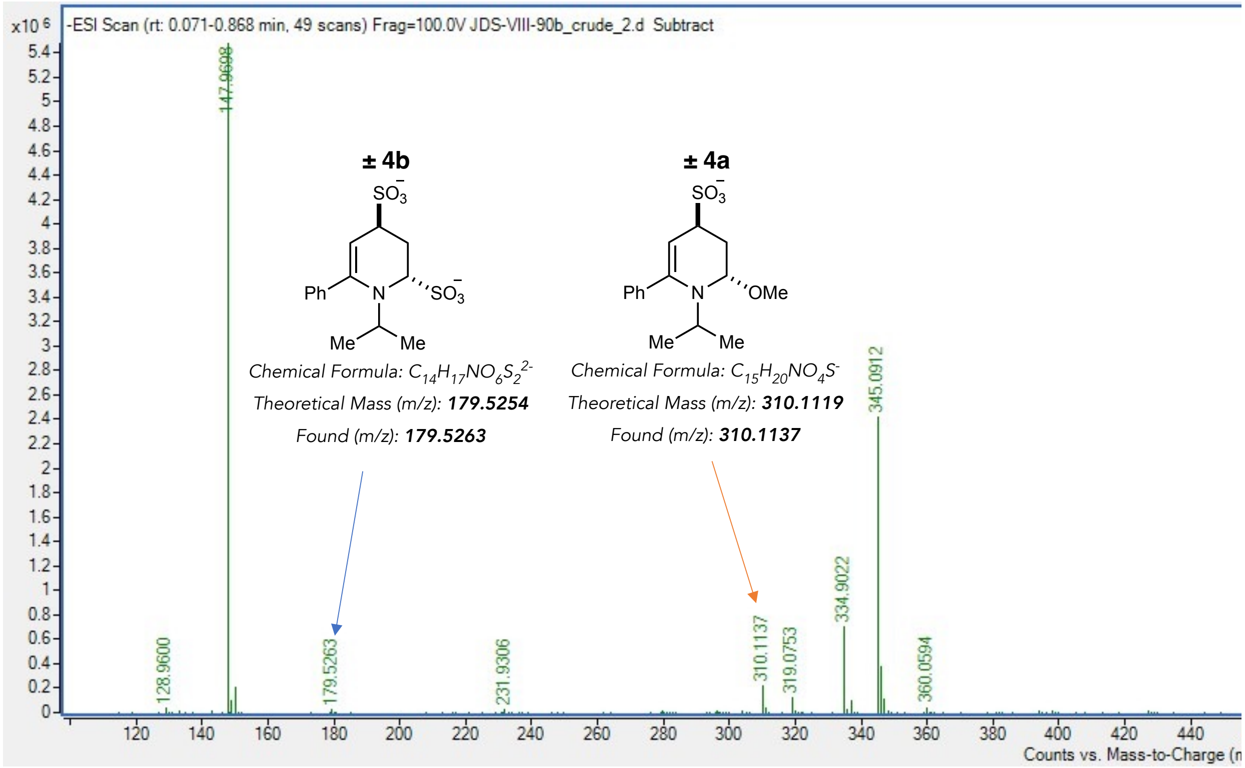

Figure S22. HRMS (ESI + APCI) spectrum of the crude reaction between Zincke imine **1a** and *iso*-propylamine at room temperature after 18 h. Methanol used as mobile phase, negative ionization $[M - K]^-$.

*Isolation and Characterization of **4b**:*

rac-Potassium (2*S*,4*S*)-1-isopropyl-6-phenyl-1,2,3,4-tetrahydropyridine-2,4-disulfonate (4b)

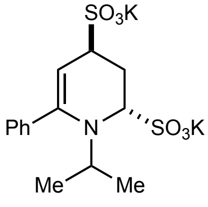

Prepared according to general procedure C using *N*-((2*E*,4*E*)-5-(dibenzylamino)-1-phenylpenta-2,4-dien-1-ylidene)-1,1,1-trifluoromethanesulfonamide (**1a**) (2.42 g, 5.00 mmol), MeOH (25.0 mL, 0.2 M), *iso*-propylamine (**2aw**) (640 µL, 7.50 mmol), potassium metabisulfite (2.22 g, 10.0 mmol), and stirred at room temperature for 96 h. Sodium carbonate (2.65 g, 25.0 mmol) was added to the reaction mixture and stirred for 5 minutes. The reaction mixture was extracted with hexanes (x3) to remove *iso*-propylamine, and the methanol layer was then decanted to remove the excess sodium carbonate. The reaction mixture was concentrated with a stream of N_2, and the resulting residue was rinsed with CH_2Cl_2, transferred to a filter frit, and washed with CH_2Cl_2 (x2), MeCN (x1) and CH_2Cl_2 (x1). The solid was collected and dried *in vacuo* to provide the title compound as a light-yellow solid which was 37% pure by weight (determined by ^1H NMR analysis with 1,3,5-trimethoxybenzene as the internal standard) (738 mg, 37% pure by weight, 0.623 mmol, 12% yield). The structure was determined using ^1H NMR, COSY, NOESY, HMBC, HSQC, ^{13}C NMR, ^{13}C DEPT-135, and HRMS experiments. ^1H NMR (400 MHz, CD_3OD) δ: 7.61 – 7.56 (m, 2H), 7.33 – 7.21 (m, 3H), 5.07 (dd, J = 2.7, 1.3 Hz, 1H), 4.35 (dd, J = 5.7, 2.0 Hz, 1H), 4.06 (ddd, J = 11.7, 7.0, 2.6 Hz, 1H), 3.48 (hept, J = 6.7 Hz, 1H), 2.91 (dddd, J = 13.8, 7.1, 1.7, 1.3 Hz, 1H), 2.21 (ddd, J = 13.8, 11.7, 5.6 Hz, 1H), 1.24 (d, J = 6.8 Hz, 3H), 0.95 (d, J = 6.7 Hz, 3H); ^{13}C NMR (100 MHz, CD_3OD) δ: 147.89, 141.82, 129.04, 128.79, 128.61, 103.67, 67.63, 54.43, 51.82, 26.38, 21.45, 18.94; HRMS (ESI + APCI) found $[M - K]^-$ 179.5267, $C_{14}H_{17}NO_6S_2^{2-}$ requires 179.5254.

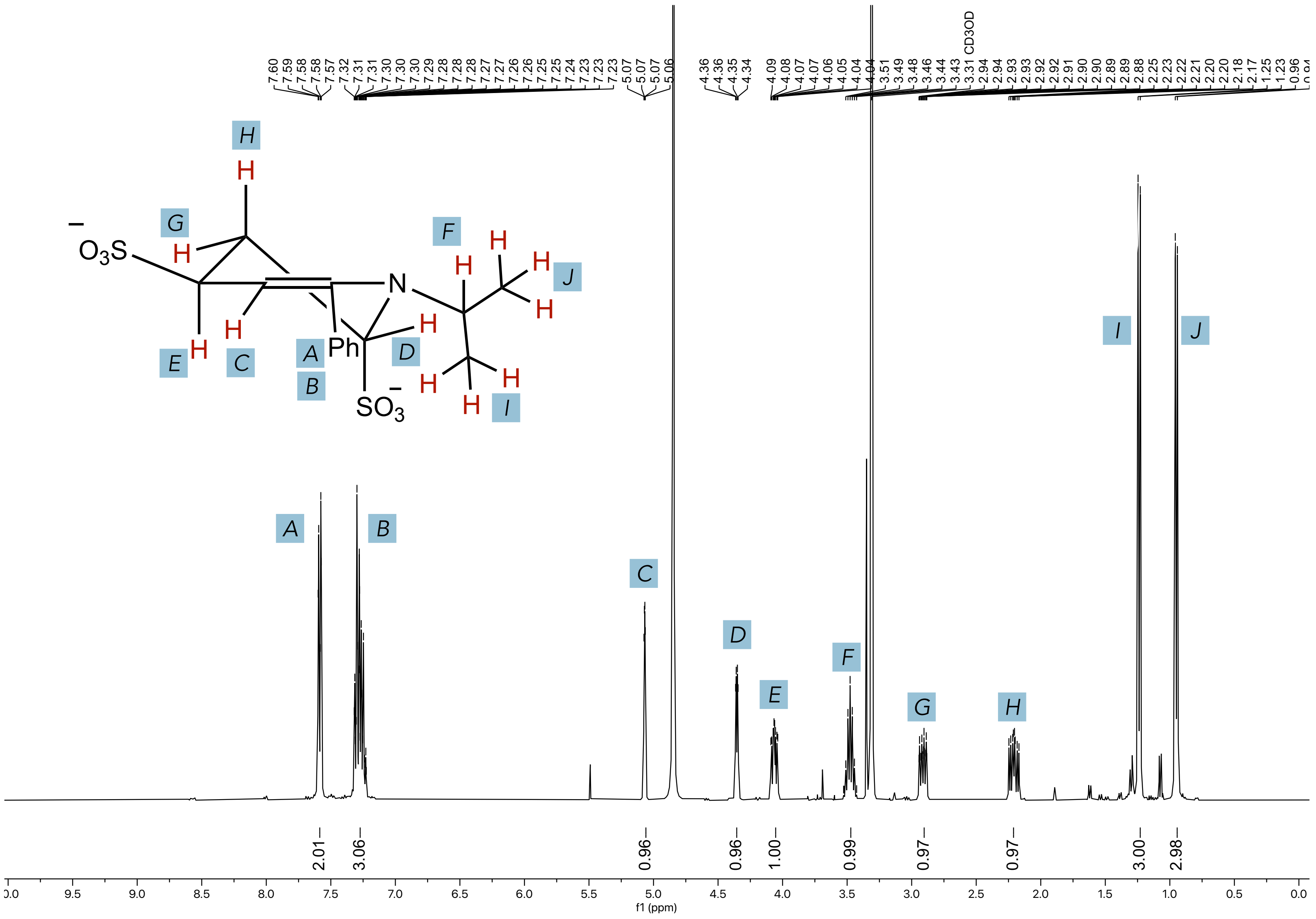

Figure S23. ^{1}H NMR (400 MHz) spectrum of **4b** in CD3OD and structural assignment.

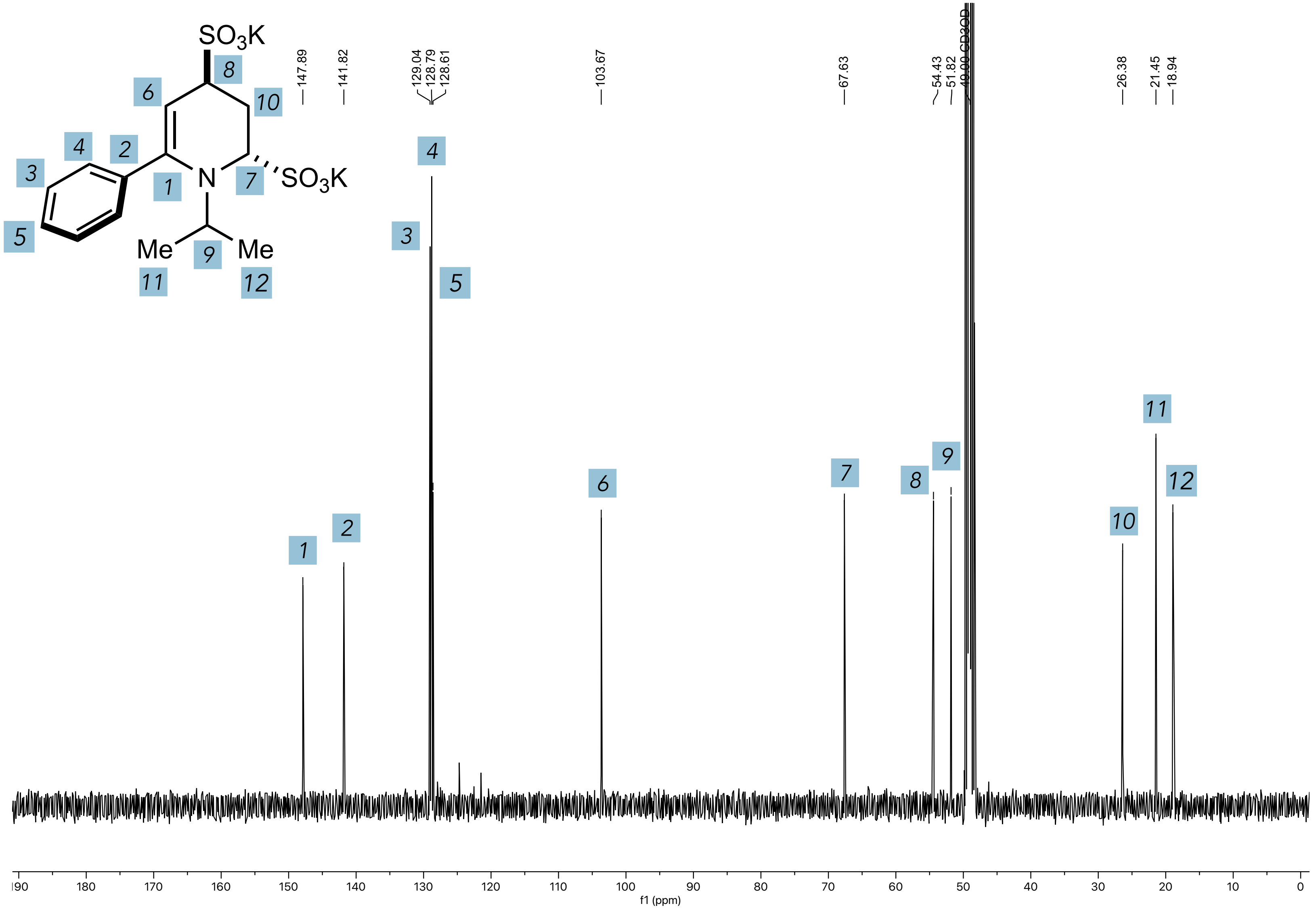

Figure S24. ^{13}C NMR (100 MHz) spectrum of **4b** in CD_3OD and structural assignment.

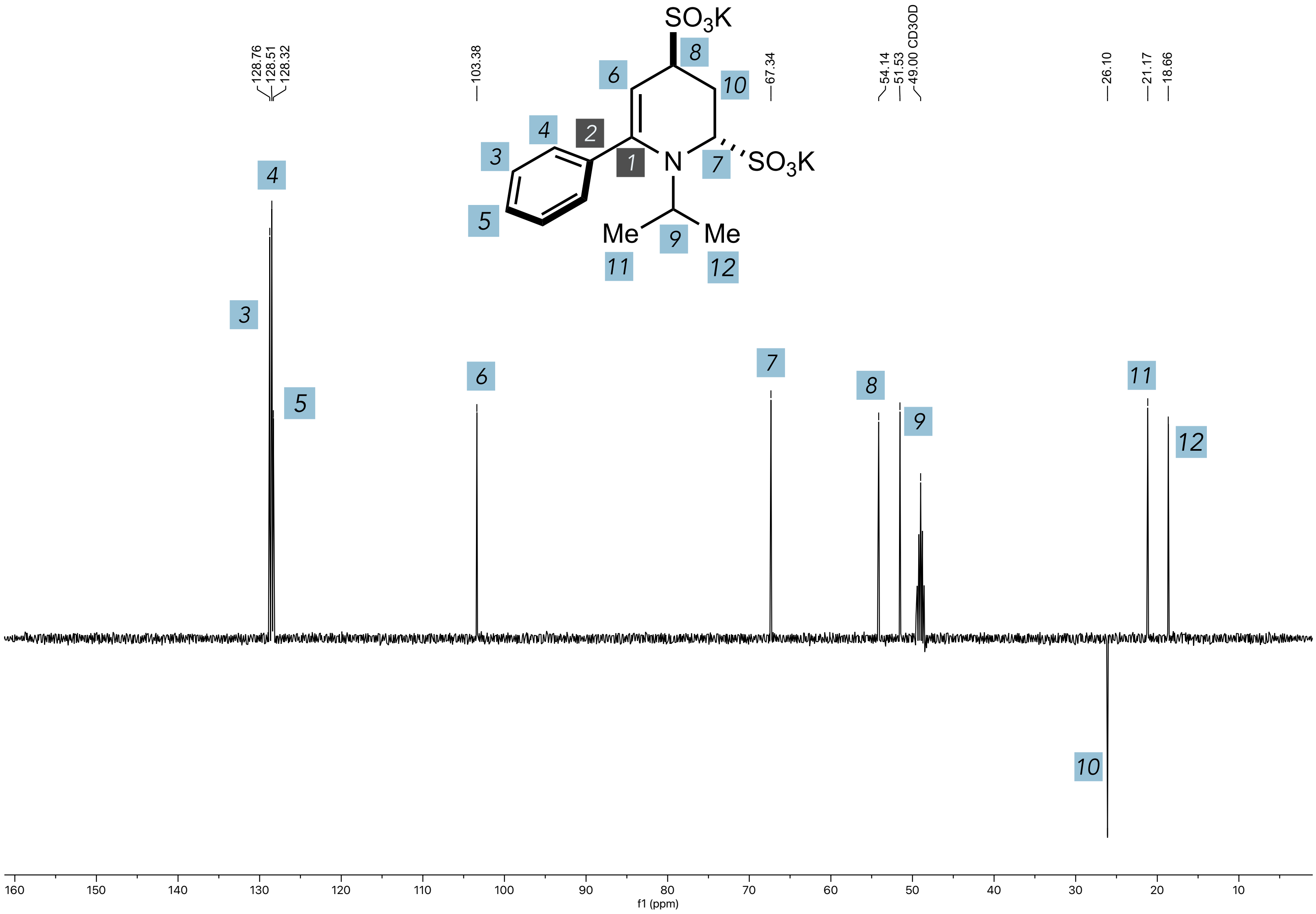

Figure S25. ^{13}C DEPT-135 (100 MHz) spectrum of **4b** in CD_3OD and structural assignment.

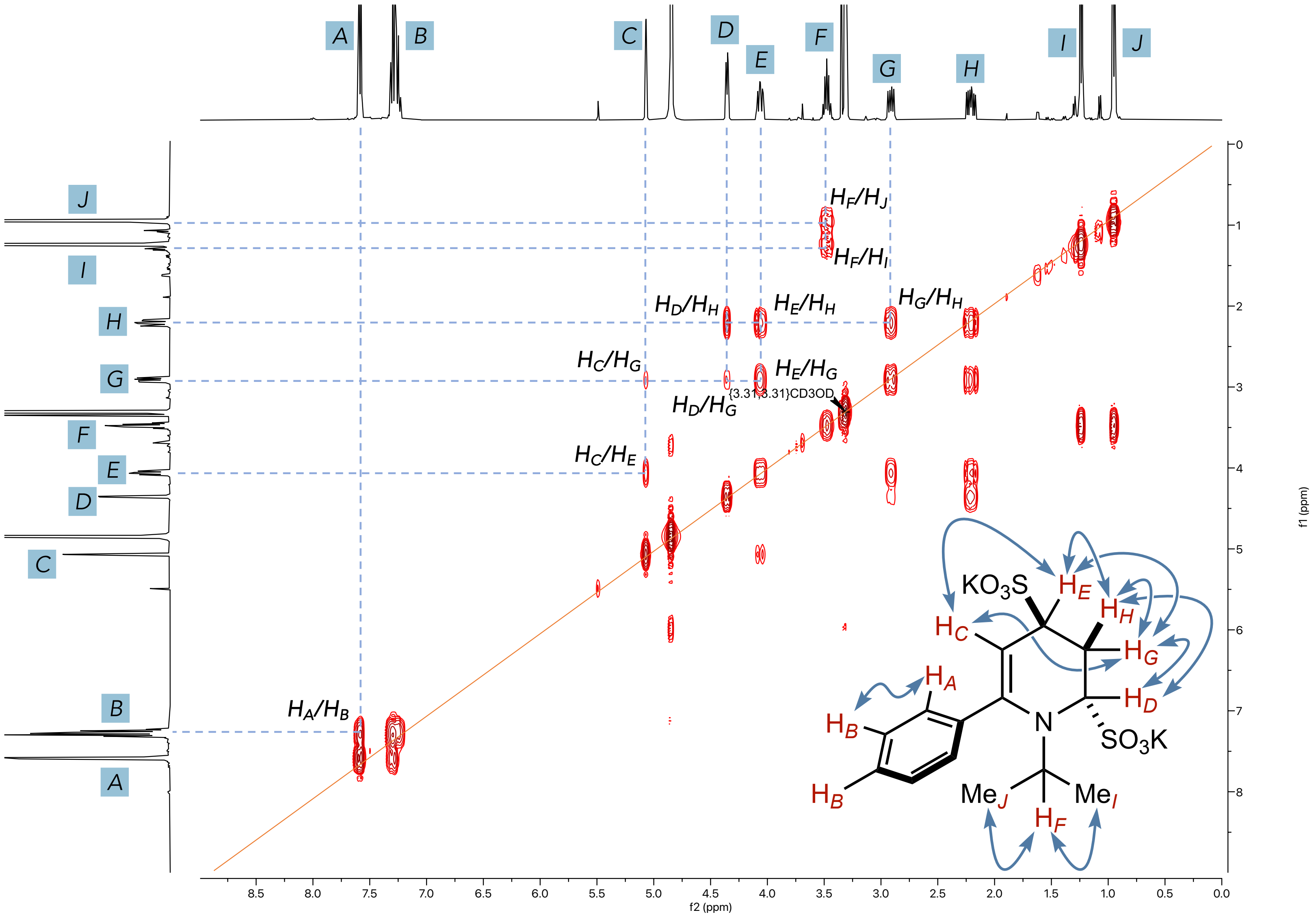

Figure S26. COSY (400 MHz) spectrum and ^{1}H-^{1}H correlations of **4b** in CD_3OD.

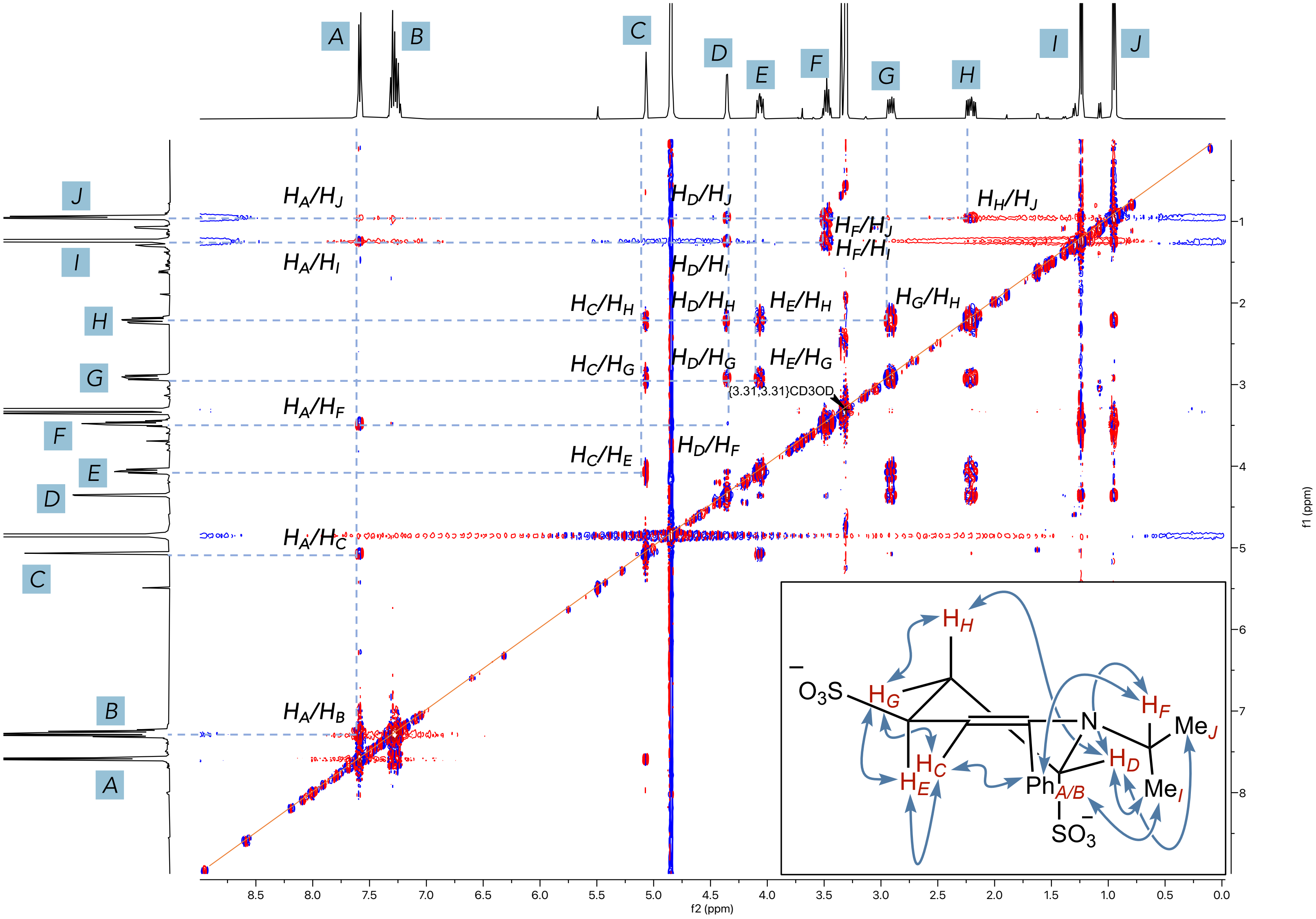

Figure S27. NOESY (400 MHz) spectrum and key characteristic ^1H-^1H NOE correlations of **4b** in CD_3OD.

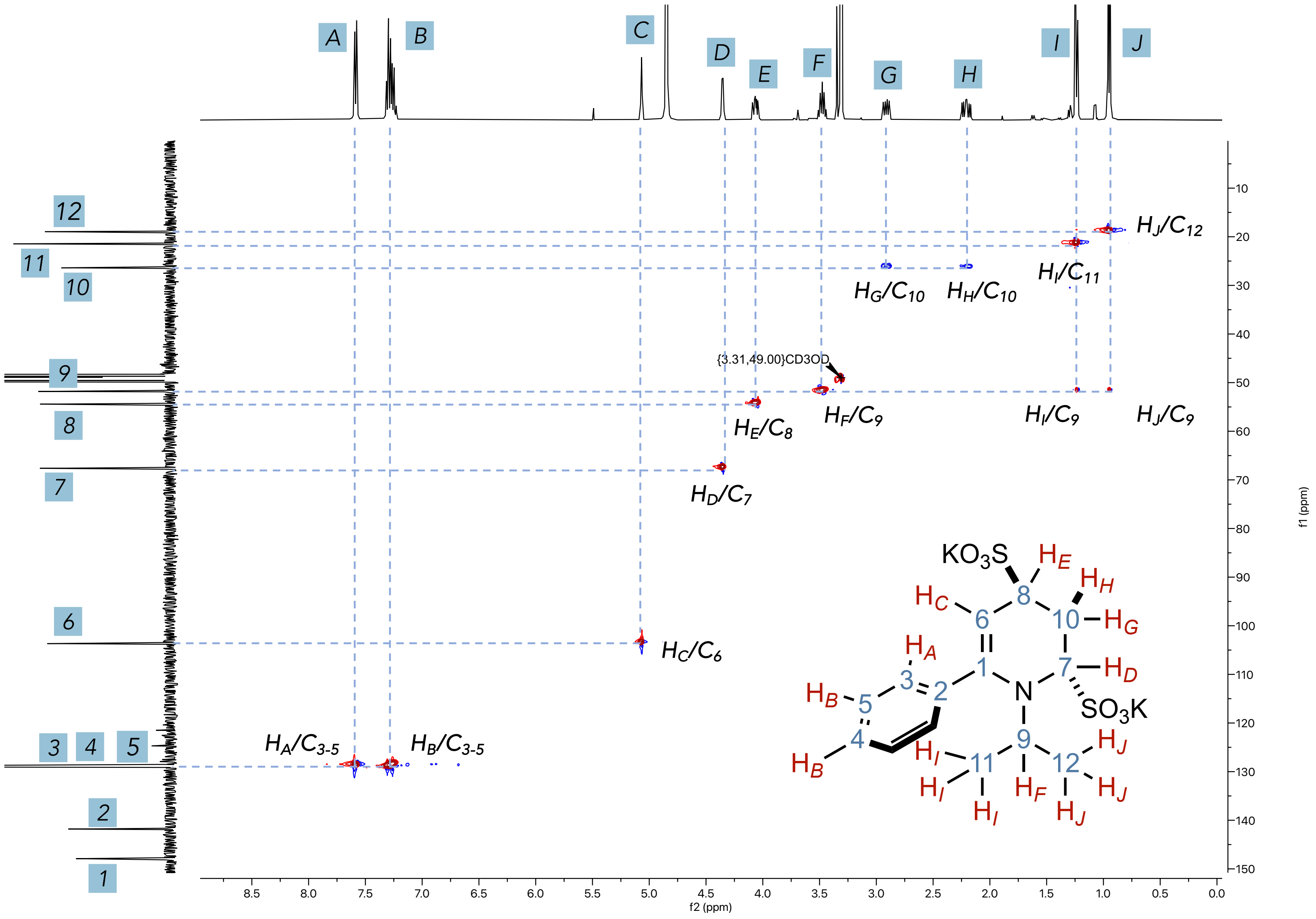

Figure S28. Multiplicity-edited HSQC (400 MHz) spectrum with ^{13}C-^{1}H correlations of **4b** in CD_3OD.

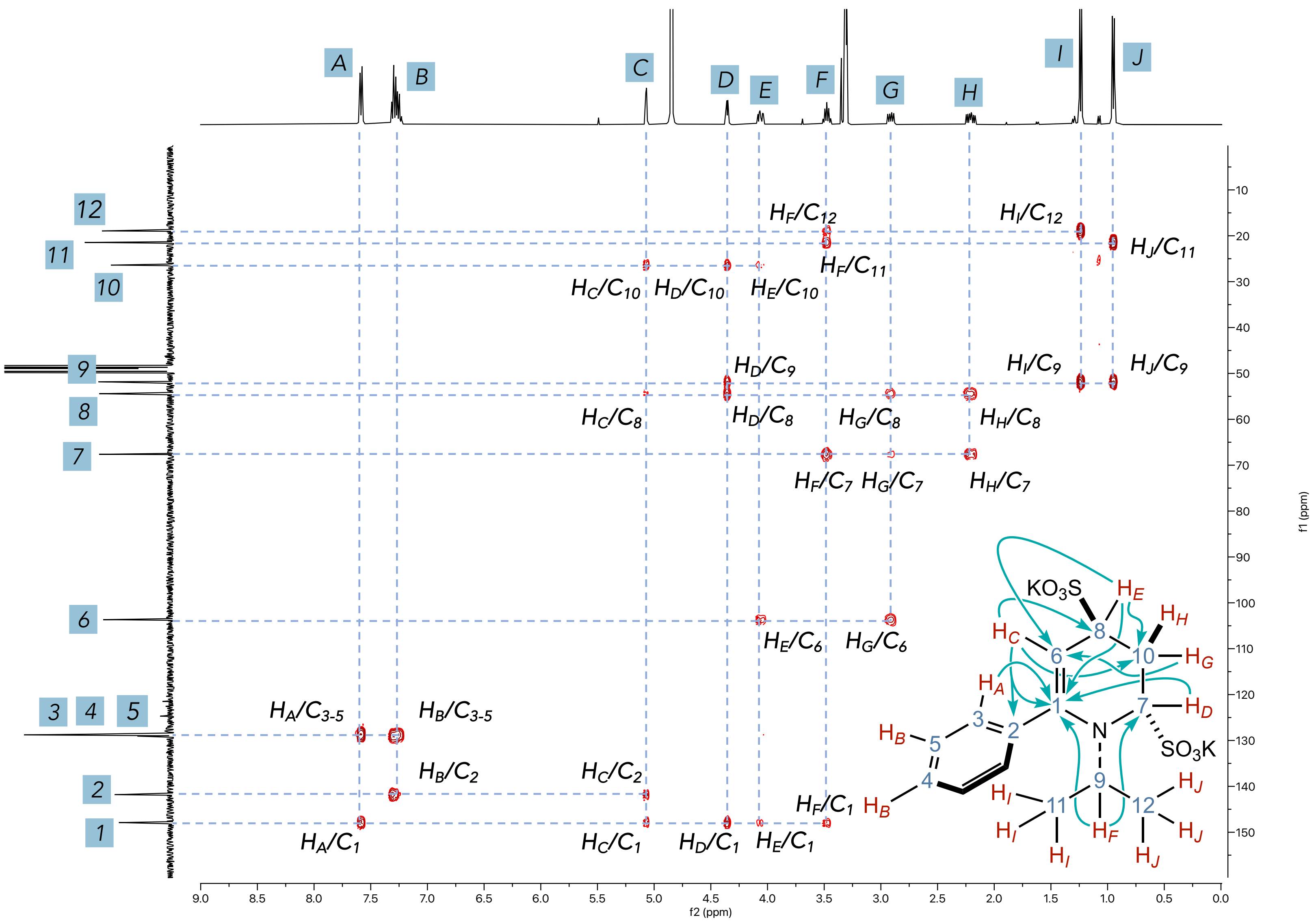

Figure S29. HMBC (400 MHz) spectrum with ^{13}C-^{1}H correlations of **4b** in CD_3OD.

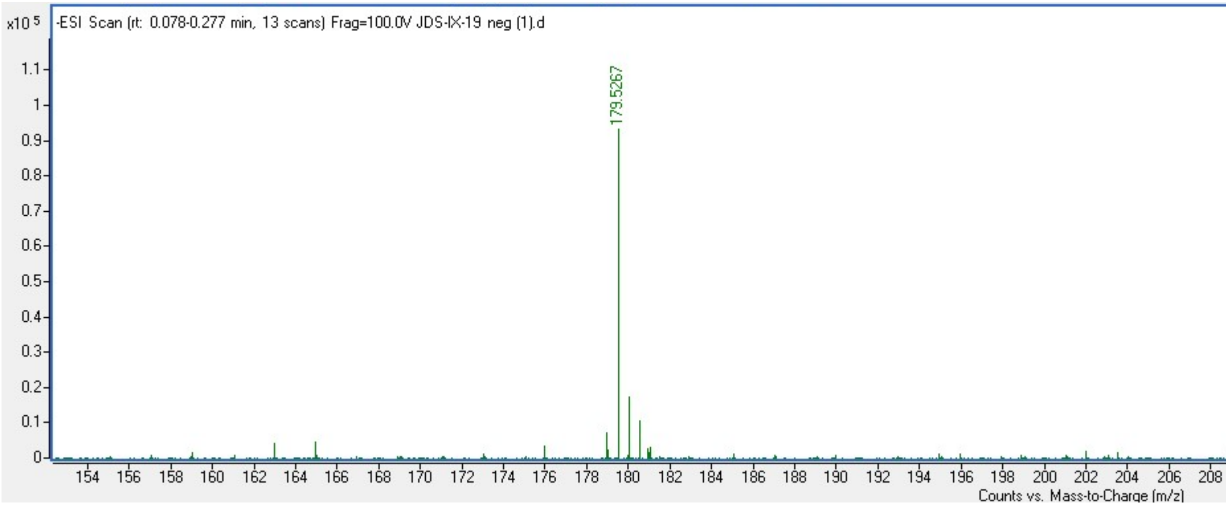

Figure S30. HRMS (ESI + APCI) of **4b**. Methanol used as mobile phase, negative ionization [M – K]$^{-}$.

*Characterization of **4a**:*

- The instability of **4a** towards formation of pyridinium **3h** and **4b** prevented its isolation from the crude reaction mixture. Characterization of **4a** was performed using the mixture of **4a** and **4b** with ^{1}H NMR, COSY, NOESY, and HRMS experiments.

***rac*-Potassium (2*S*,4*S*)-1-isopropyl-2-methoxy-6-phenyl-1,2,3,4-tetrahydropyridine-4-sulfonate (4a)**

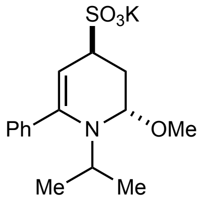

Prepared according to general procedure C using *N*-((2*E*,4*E*)-5-(dibenzylamino)-1-phenylpenta-2,4-dien-1-ylidene)-1,1,1-trifluoromethanesulfonamide (**1a**) (194 mg, 0.400 mmol), MeOH (2.00 mL, 0.2 M), *iso*-propylamine (**2aw**) (52.0 µL, 0.600 mmol), potassium metabisulfite (178 mg, 0.800 mmol), and stirred at room temperature for 18 h. The reaction mixture was extracted with hexanes (x3) to remove *iso*-propylamine, and the methanol layer was then concentrated with a stream of N_2, and the resulting residue was rinsed with CH_2Cl_2, transferred to a filter frit, and washed with CH_2Cl_2 (x2), MeCN (x1) and CH_2Cl_2 (x1). The solid was collected and dried *in vacuo* to provide the title compound as a light-yellow solid as a mixture with **4b** (1:4). ^{1}H NMR (400 MHz, CD_3OD) δ: 7.49 – 7.44 (m, 2H), 7.37 – 7.22 (m, 3H), 5.09 (dd, *J* = 2.6, 1.3 Hz, 1H), 4.65 (t, *J* = 2.7 Hz, 1H), 3.74 – 3.69 (m, 1H), 3.44 (s, 3H), 3.72 (ddd, *J* = 12.3, 6.9, 2.5 Hz, 1H), 2.39 (dddd, *J* = 13.0, 6.8, 2.7, 1.4 Hz, 1H), 1.99 (td, *J* = 12.7, 2.7 Hz, 1H), 1.25 (t, *J* = 6.7 Hz, 6H); HRMS (ESI + APCI) found $[M – K]^-$ 310.1130, $C_{15}H_{20}NO_4S^-$ requires 310.1119.

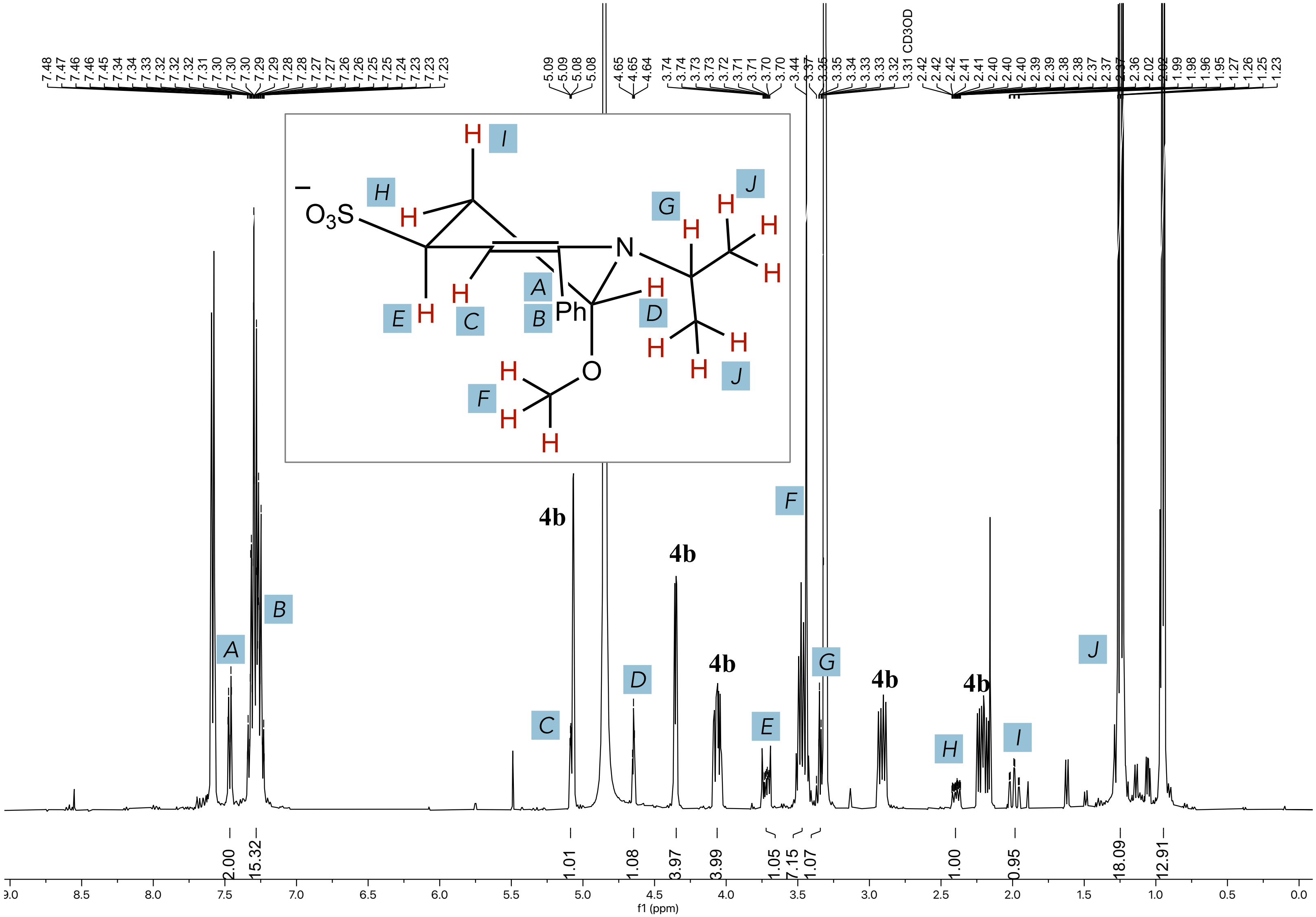

Figure S31. ^{1}H NMR (400 MHz) spectrum of **4a** in CD_3OD (with **4b**) and structural assignment.

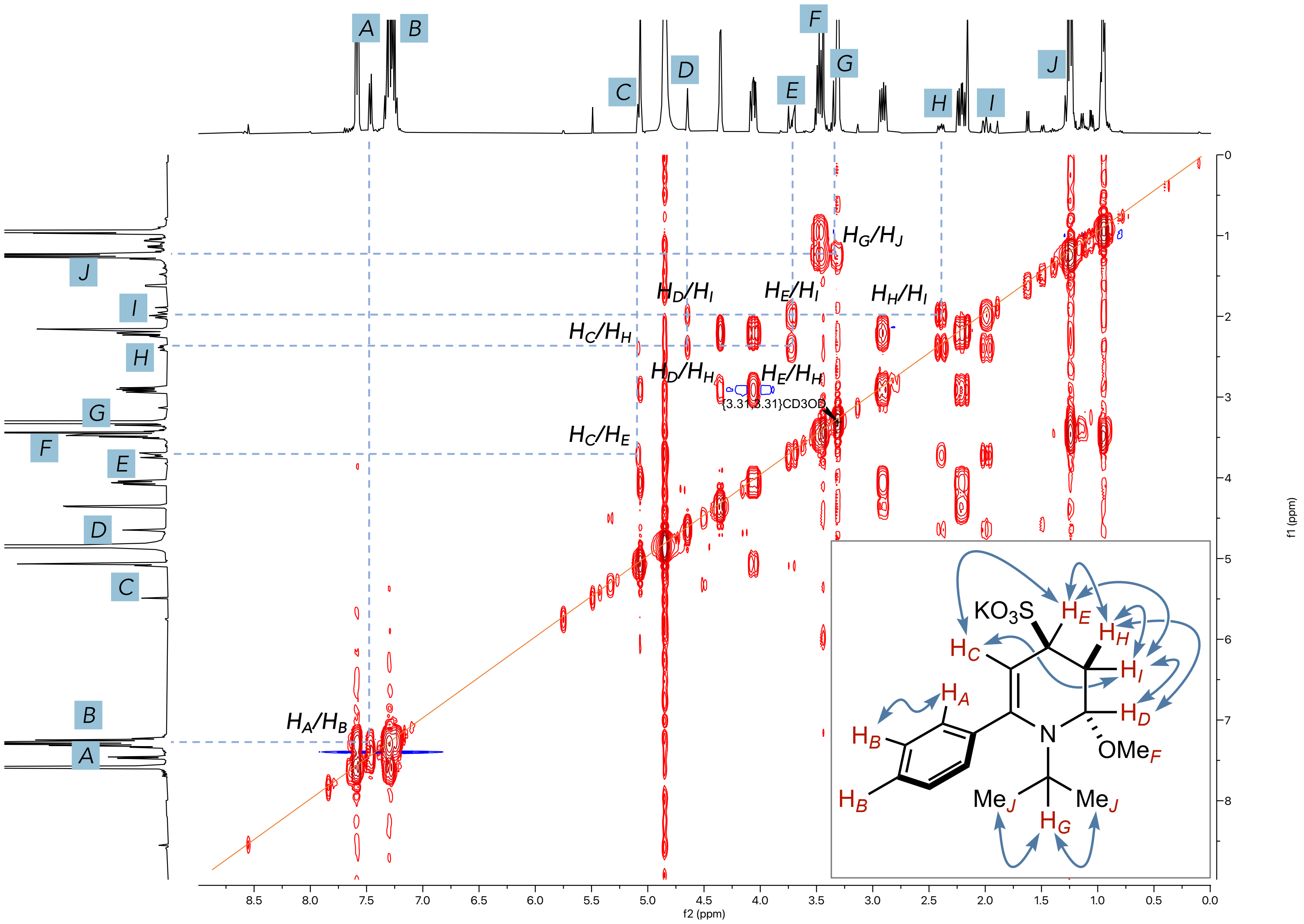

Figure S32. COSY (400 MHz) spectrum and ^1H-^1H correlations of **4a** (with **4b**) in CD_3OD.

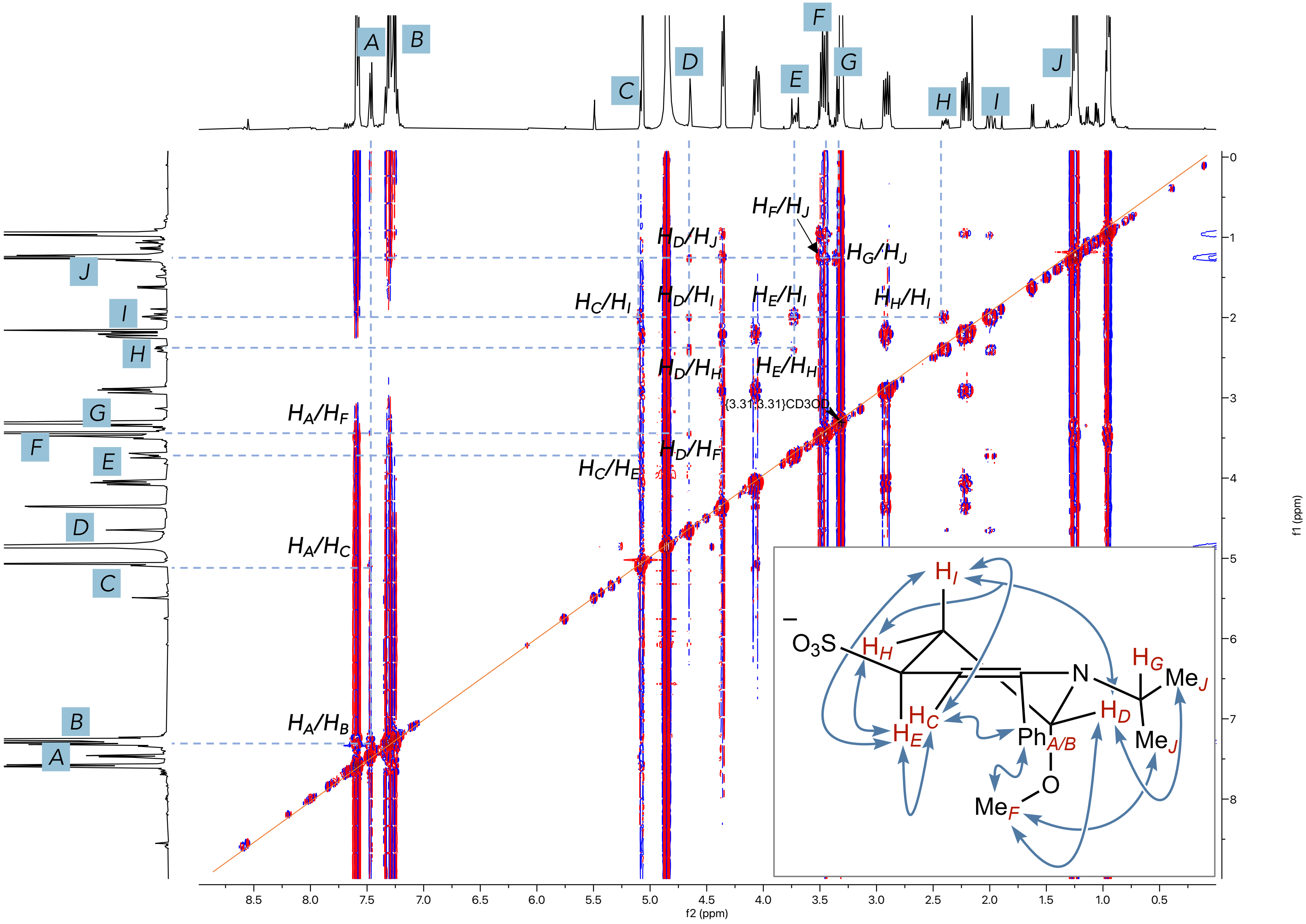

Figure S33. NOESY (400 MHz) spectrum and key characteristic ^{1}H-^{1}H NOE correlations of **4a** (with **4b**) in CD_3OD.

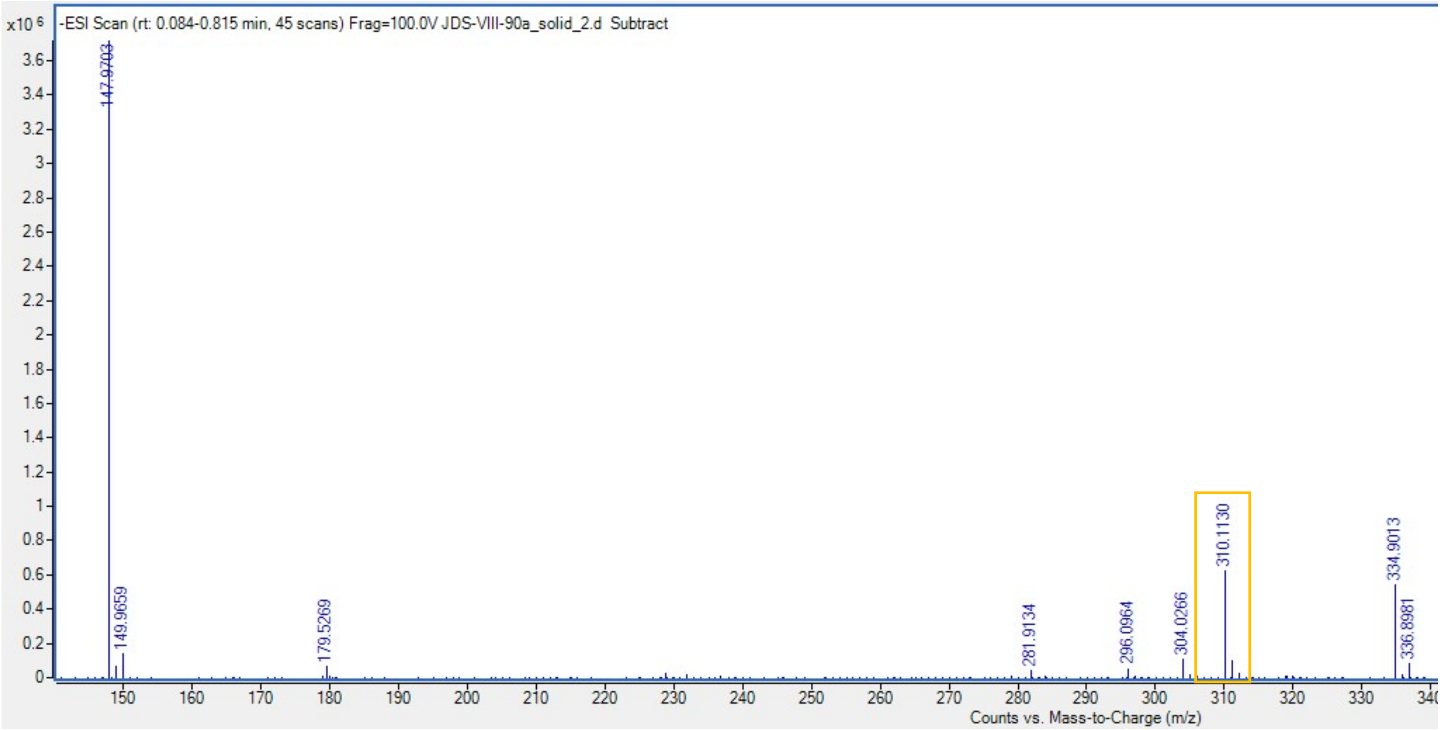

Figure S34. HRMS (ESI + APCI) of **4a**. Methanol used as mobile phase, negative ionization $[M – K]^{-}$.

*Time Study of **4a** and **4b** Formation at Room Temperature:*

Table S12. Time study for the formation of 4a and 4b at room temperature from Zincke imine 1a and amine 2aw.[a]

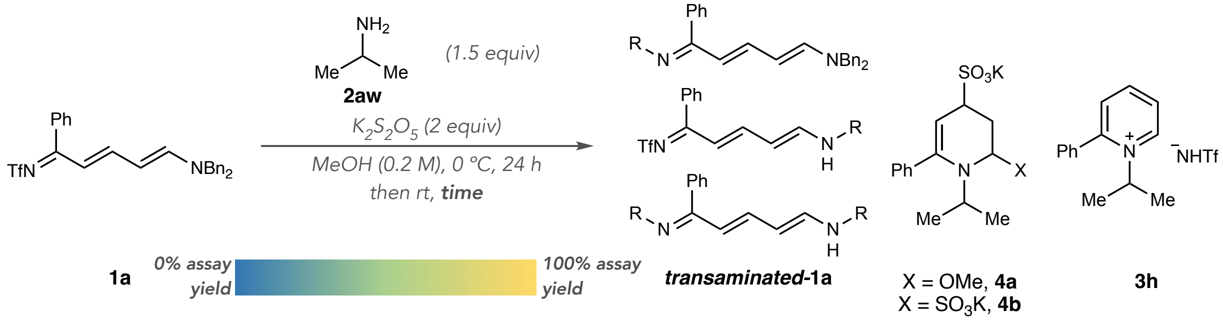

entry	Time at rt (h)	% **1a remaining**	% ***trans-aminated*-1a**	% **4a**	% **4b**	% **3h**
1	0	30	39	2	0	8
2	1	26	26	5	2	21
3	2	17	24	10	5	33
4	4	7	12	15	9	50
5	6	3	8	19	12	58
6	23	0	0	16	23	65

[a]Reaction run using 0.1 mmol of **1a**. Yields determined by ^{1}H NMR using 1,3,5-trimethoxybenzene as an internal standard in CD_3OD. Amine added to a solution of Zincke imine **1a** and $K_2S_2O_5$ at 0 ºC and stirred for 24 h before warming to room temperature. Direct aliquots taken from the reaction mixture for ^{1}H NMR analysis at various time points.

*Benchtop Stability of **4a** and **4b** After Isolation:*

- Storing the isolated mixture of **4a** and **4b** on the benchtop at room temperature results in the decomposition of **4a** to pyridinium **3h**, while **4b** is stable in the isolated mixture.

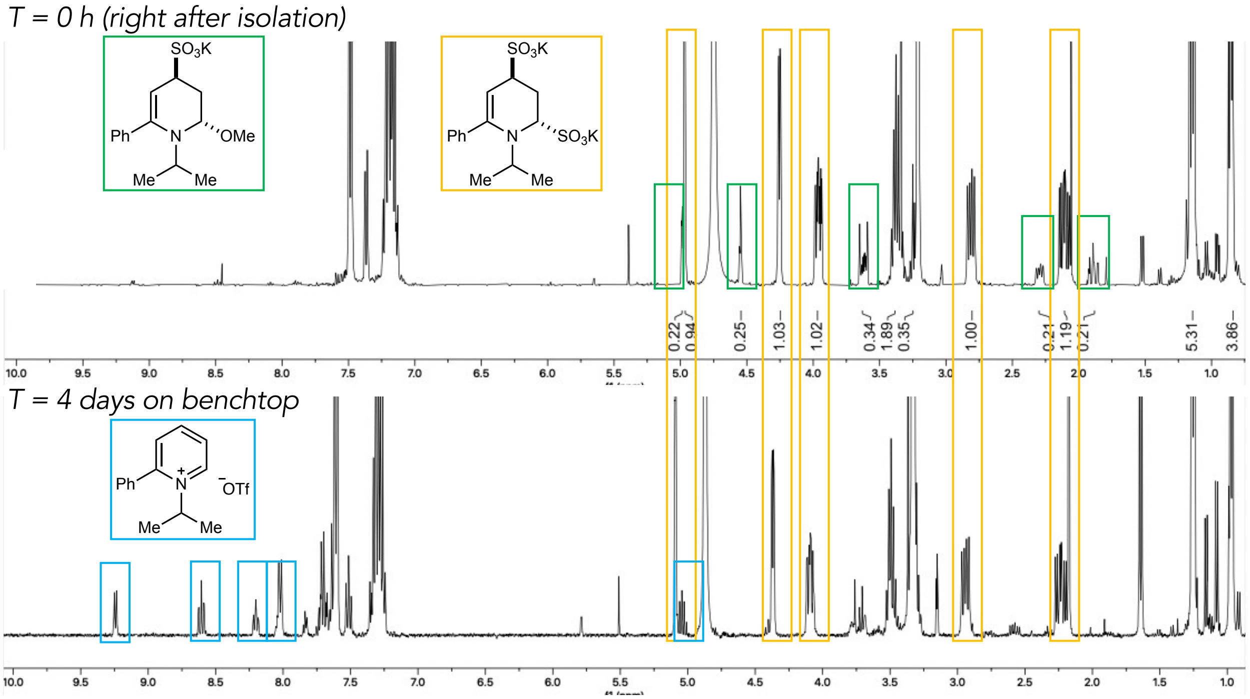

Figure S35. ^{1}H NMR (400 MHz) spectra of isolated mixture directly after isolation and drying *in vacuo* (top) and after storing on the benchtop in ambient conditions for 4 days (bottom). NMR samples prepared directly before running experiment for both time points.

*Conversion of **4a** and **4b** to Pyridinium Salt **3h** with Isolated Mixture:*

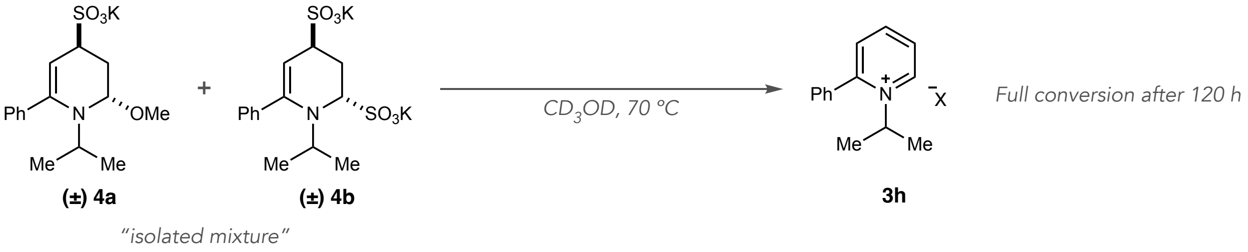

The isolated mixture of **4a** and **4b** was dissolved in CD_3OD and transferred to an NMR tube. The vessel was sealed and checked by ^{1}H NMR (400 MHz). The NMR tube was then heated to 70 ºC for 120 h without stirring. The vessel was cooled to room temperature and checked by ^{1}H NMR (400 MHz) for the conversion of **4a** and **4b** to pyridinium **3h**. The results are shown in Figure S36.

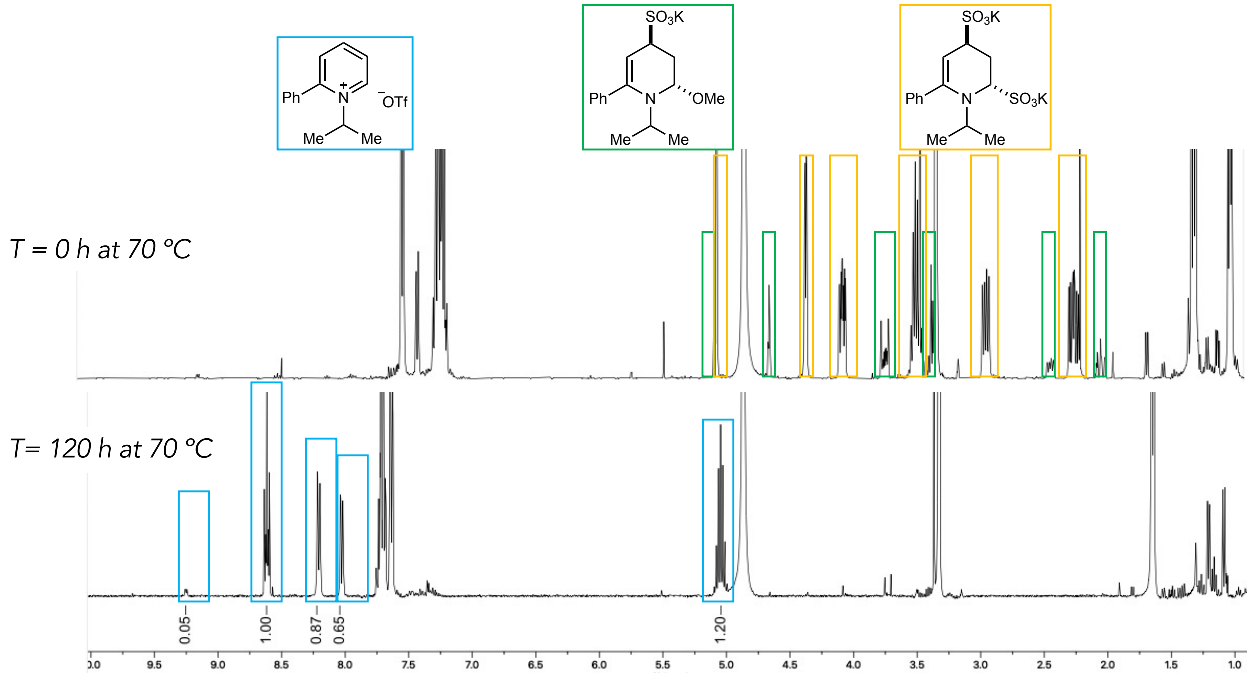

Figure S36. ^{1}H NMR spectra of isolated mixture before (top) and after heating in CD3OD (bottom). [a]C3-, C5-, and C6-positions of **3h** are exchanged with deuterium under the reaction conditions in CD3OD.

*Formation of **4a** and **4b** from Pyridinium Salt **3h**:*

Scheme S5. Investigation into the formation of 4a and 4b from isolated pyridinium salt 3h under the reaction conditions for forming 3h.[a]

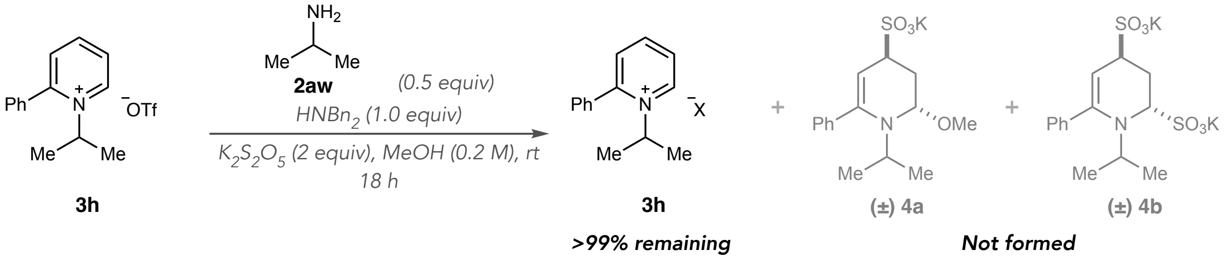

[a]Reactions run using 0.1 mmol of **3h**. Yields determined by ^{1}H NMR using 1,3,5-trimethoxybenzene as an internal standard in CD3OD.

*Effect of Potassium Metabisulfite Equivalents on **4a** and **4b** Formation:*

Table S13. Effect of potassium metabisulfite stoichiometry on the formation of 4a and 4b from Zincke imine 1a and amine 2aw.[a]

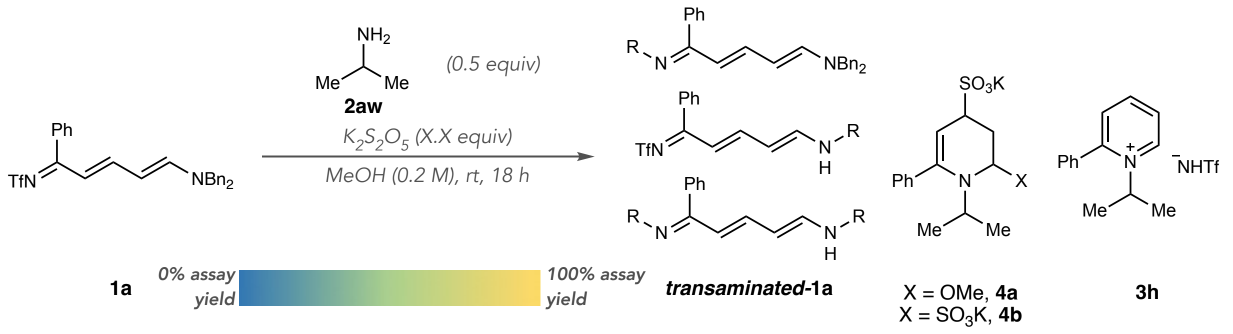

entry	$K_2S_2O_5$ equiv.	% 1a remaining	% *trans-aminated*-1a	% 4a	% 4b	% 3h
1	2.0	0	5	14	13	**52**
2	1.0	0	8	18	11	**59**
3	0.5	0	10	17	7	**55**
4	0.25	0	15	16	5	**55**

[a]Reaction run using 0.1 mmol of **1a**. Yields determined by ^{1}H NMR using 1,3,5-trimethoxybenzene as an internal standard in CD_3OD.

*Stability of **4a** and **4b** in Crude Reaction Mixture with Different Stimuli:*

Table S14. Reactivity and stability of 4a and 4b towards pyridinium salt 3h formation under various conditions in the crude reaction mixture.[a]

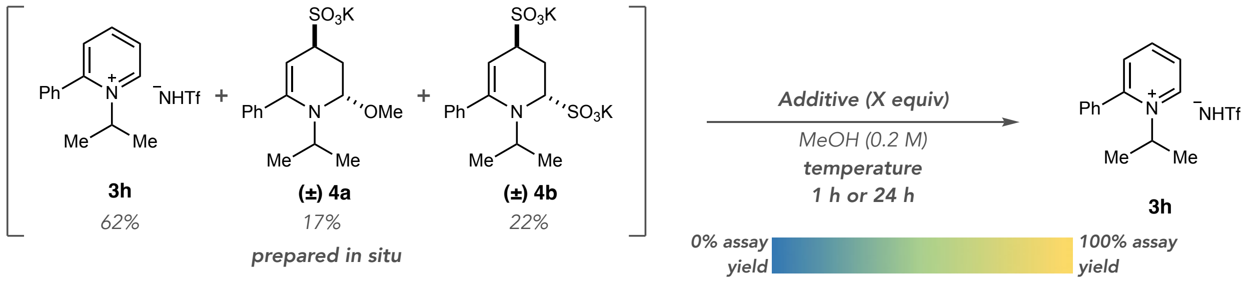

entry	additive (equiv)	temperature (°C)	time (hours)	% 4a remaining	% 4b remaining	% 3h
1	none	25	1	16	23	**62**
2	none	25	24	10	30	**62**
3	none	70	1	0	0	**98**
4	none	70	24	0	0	**100**
5	*i*-$PrNH_2$ (5 equiv)	25	1	18	19	**62**
6	*i*-$PrNH_2$ (5 equiv)	25	24	16	20	**65**
7	Bn_2NH (5 equiv)	25	1	17	16	**62**
8	Bn_2NH (5 equiv)	25	24	13	21	**70**
9	DBU (5 equiv)	25	1	18	19	**62**
10	DBU (5 equiv)	25	24	16	24	**64**
11	formic acid (5 equiv)	25	1	0	19	**78**
12	formic acid (5 equiv)	25	24	0	2	**86**
13	mesic acid (5 equiv)	25	1	0	0	**92**
14	mesic acid (5 equiv)	25	24	0	0	**98**
15	H_2SO_4 (5 equiv)	25	1	0	0	**98**
16	H_2SO_4 (5 equiv)	25	24	0	0	**100**

[a]Mixture of **3h**, **4a**, and **4b** prepared in situ using general procedure C at room temperature using 2.0 mmol of Zincke imine **1a**. The crude reaction mixture was divided into 0.1 mmol aliquots for screening of reaction conditions. Yields determined by ^{1}H NMR using 1,3,5-trimethylbenzene as an internal standard in CD_3OD.

7.7. Computation Details

7.7.1 General Information

All stationary point geometries were optimized using the hybrid meta Minnesota functional, M06-2X[1] with Grimme's empirical D3[2] dispersion correction and Pople's 6-31+G(d,p)[3–9] basis set implemented in Gaussian 16 using a Polarizable Continuum Model[10–12] (PCM) representation of methanol. Vibrational frequency calculations were used to confirm stationary points as minima or first-order saddle points on the

potential energy surface (PES) and to obtain quasi-harmonic rigid-rotor/harmonic oscillator[13] thermochemistry values with the GoodVibes[14] program. Where possible, Intrinsic Reaction Coordinate (IRC) calculations were used to verify that the transition structures are connected to their preceding and following intermediates. Energies were refined using single-point energy calculations (SPC) at the ωB97M-V[15]/def2-TZVPP[16] level of theory using a CPCM[17] representation of methanol. Conformer ensembles were generated for each **Int** and **TS** structure using the Global Optimizer Algorithm[18] (GOAT) implemented in ORCA 6.0.1 with GFN2-xTB[19]. These ensembles were reduced to a maximum of 10 representative structures for DFT optimization and single point correction using Principal Component Analysis (PCA) clustering implemented in CREST[20] and manually supplemented as needed. After conformational sampling, duplicate structures at the DFT level were excluded. Individual conformers are denoted by "_conf_x" at the end of the structure name where x is an integer. Grimme's quasi-harmonic corrections were introduced to the computed vibrational entropies using a frequency cut-off value of 100.0 cm-1 with GoodVibes[21] at 313.15 K. Also, a correction for the change in standard state from gas phase at 1 atm to a 1 M solution was introduced. Boltzmann weighted Gibbs energies are quoted throughout, which include considerations of molecular point group and entropies of mixing.

7.7.2 Evaluation of Methods

To probe the system's sensitivity to functional and solvation model used in our computations, we benchmarked the level of theory for single point energy corrections, focusing on the protonation thermodynamics in the first step of the first step, as well as the energies of **TS1** and **TS5** relative to the non-protonated, transaminated adduct **Int-VII** (Figure S37). We found these results to be qualitatively consistent and thus chose to use ωB97M-V with a CPCM description of methanol.

**Functional**	**Solvation Model**	**Int-VIII**	**TS1**	**TS5**
ωB97M-V	CPCM	-6.4	6.3	17.5
ωB97M-V	SMD[22]	-8.7	6.8	14.9
M06-2X	CPCM	-8.0	6.0	18.4
M06-2X	SMD	-10.2	6.7	15.9

Figure S37. Benchmarking studies with the def2-TZVPP basis set using M06-2X(D3)/6-31+g(d,p) geometries. All energies are relative to **Int-VII** and listed in kcal/mol.

7.7.3 Proposed Mechanism without Bisulfite

To understand the role of bisulfite, we constructed a Gibbs energy surface for the proposed reaction mechanism without bisulfite (Figure S38). This sequence starts with the same protonation step from **Int-VII** to **Int-VIII** as the main pathway shown in Figure 2. Without bisulfite, this ring closing is relatively challenging, with an energy span of 23.9 kcal/mol to yield the ring-closed **Int-S1**. After a proton transfer, **Int-S2** rearomatizes via the low-barrier **TS-S1** to give the product **3h**.

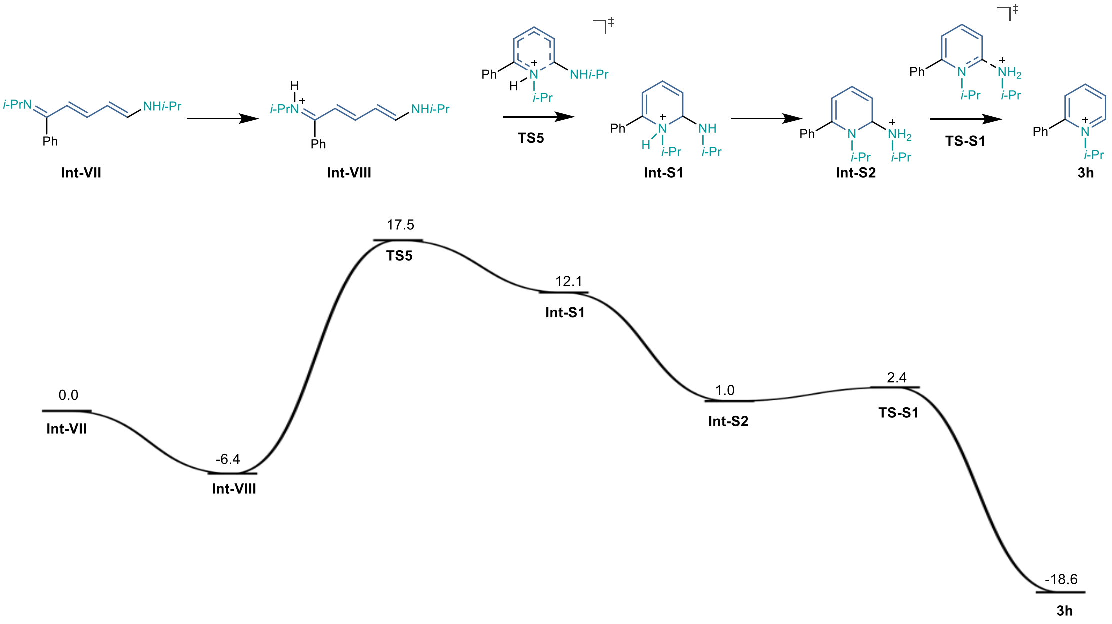

Figure S38. Proposed Gibbs energy surface for the reaction without bisulfite additive computed at the ωB97M-V/def2-TZVPP/CPCM(methanol)//M06-2X(D3)/6-31+G(d,p)/PCM(methanol) level of theory.

7.7.4 Protonation Equilibria

We computed the thermodynamics of protonating **Int-VII** to yield **Int-VIII** with several possible proton sources (Figure S39). With acid present in the system, we proposed that protonated dibenzyl amine is an appropriate acid and dibenzyl amine an appropriate conjugate base for modeling the system (i.e., a competent proton source for which the reaction with **Int-VII** is exergonic). In the case of no acid additives, we proposed that NH_2Tf, generated in the transamination step, is the most reasonable proton source. The PES's calculated using these two different proton sources are qualitatively similar. Additionally, the energy span of the reaction with bisulfite ($\Delta G^{\ddagger}$ = 19.9 kcal/mol) and the bisulfite addition step via **TS1** are the same with either proton source, and the overall reaction is exothermic in both cases.

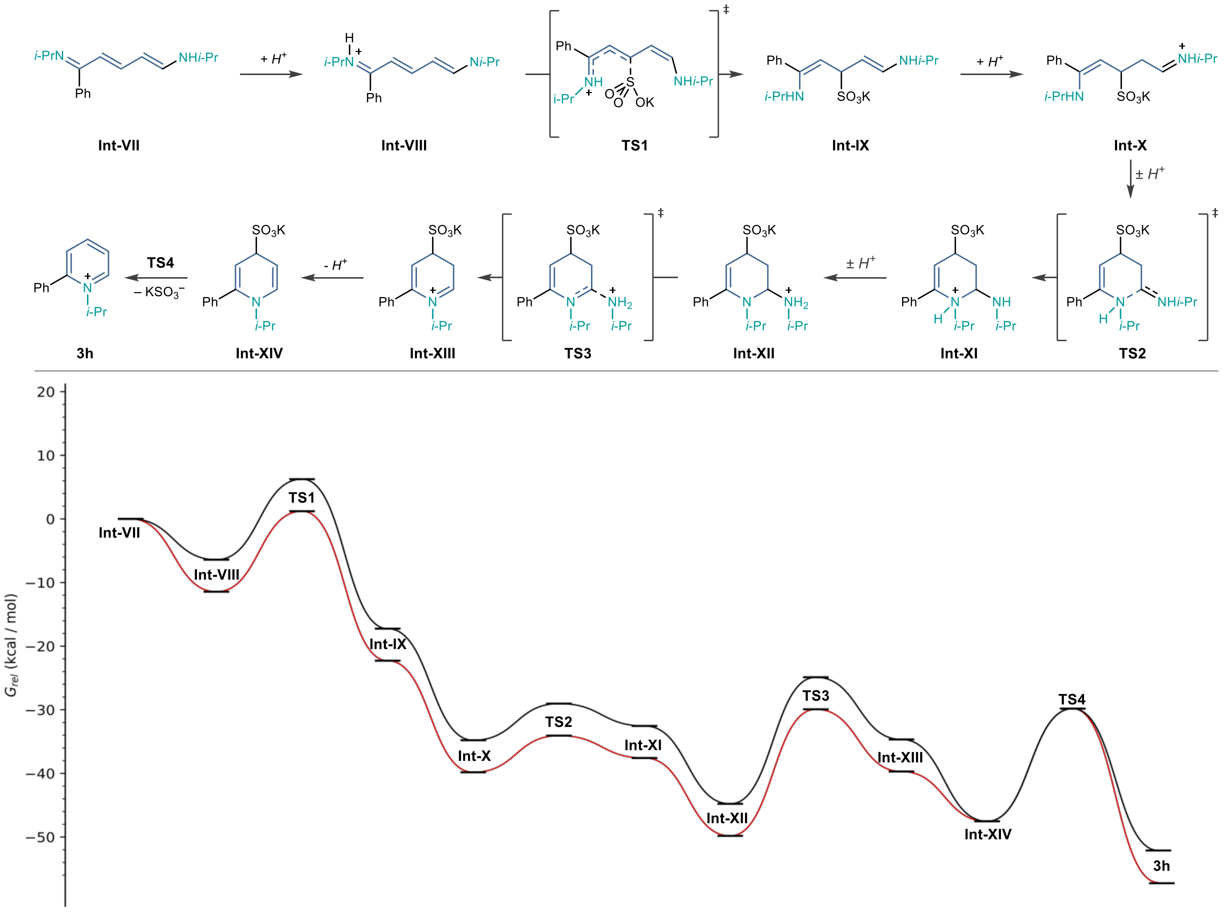

Figure S39. Potential energy surface (PES) for the reaction using $NH_2Bn_2^+$ (red) and NH_2Tf (black) as the proton source.

7.7.5 Formation of bisulfite adducts

We sought to identify possible routes to the formation of the experimentally observed bisulfite adducts **4a** and **4b** shown in Scheme 4B and 4C. In this endeavor, we were able to locate transition state structures (**TS_S2** and **TS_S3** respectively) connecting the iminium **Int-VIII** to these adducts (Figure S40) via kinetically accessible barrier heights.

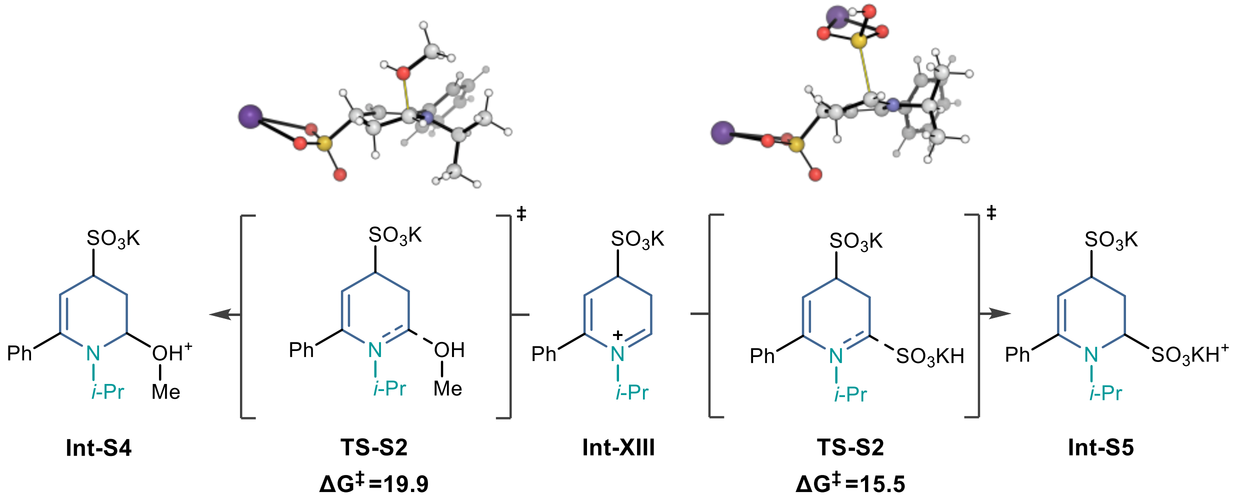

Figure S40. Transition structures leading to the observed bisulfite adducts from **Int-XIII**. Barriers in kcal/mol.

7.7.6 Thermochemical Data

Legend:
E_{SPC} = energy obtained in the single-point energy corrections
E = energy obtained in the geometry optimizations
ZPE = zero-point energy
H_{SPC} = enthalpy corrected with ESPC
T·S = temperature times entropy with no correction
T·qh-S = temperature times entropy with quasi-harmonic S correction
G(T) = Gibbs free energy corrected only with ESPC
qh-G(T) = Gibbs free energy with ESPC and quasi-harmonic S correction
ν_{imag} = imaginary frequencies

Structure	E_{SPC}	E	ZPE	H_{SPC}	T·S	T·qh-S	G(T)	qh-G(T)	ν_{imag}
**3h _conf_1**	-597.700556	-597.492286	0.260826	-597.423845	0.056022	0.054120	-597.479867	-597.477965	
**3h _conf_4**	-597.694629	-597.487002	0.261091	-597.417758	0.055682	0.053729	-597.473441	-597.471487	
**DBA**	-597.248946	-597.044441	0.249039	-596.984543	0.057999	0.054278	-597.042542	-597.038821	
**KHSO3**	-1224.504189	-1224.308754	0.021050	-1224.475019	0.039119	0.038398	-1224.514138	-1224.513417	
**KSO3minus**	-1224.026023	-1223.827213	0.010558	-1224.008408	0.036383	0.036141	-1224.044790	-1224.044549	
**MeOSidepdt**	-1263.790816	-1263.588093	0.049264	-1263.732082	0.042529	0.041572	-1263.774611	-1263.773654	
**NH2Tf**	-942.332890	-941.957713	0.049989	-942.273102	0.041963	0.041334	-942.315064	-942.314436	
**NHtfminus**	-941.870795	-941.501268	0.037460	-941.824071	0.040788	0.040326	-941.864860	-941.864397	
**TS1_conf_0**	-1996.188005	-1995.733021	0.389712	-1995.766961	0.095056	0.086978	-1995.862017	-1995.853939	-212.33
**TS1_conf_10_TS**	-1996.186475	-1995.734019	0.389920	-1995.765344	0.095064	0.087068	-1995.860408	-1995.852412	-195.20
**TS1_conf_4_TS**	-1996.189606	-1995.736540	0.389574	-1995.768627	0.096003	0.087546	-1995.864631	-1995.856174	-185.91
**TS1_conf_8_TS**	-1996.184629	-1995.732161	0.389596	-1995.763744	0.094963	0.087018	-1995.858707	-1995.850762	-222.05
**TS1_conf_9_TS**	-1996.187263	-1995.734107	0.389290	-1995.766453	0.097163	0.088163	-1995.863616	-1995.854617	-190.67
**TS2_conf_0**	-1996.679502	-1996.216823	0.407215	-1996.243044	0.089022	0.082369	-1996.332065	-1996.325412	-115.27
**TS2_conf_10_TS**	-1996.680743	-1996.216912	0.406099	-1996.244956	0.088352	0.082709	-1996.333309	-1996.327665	-55.73
**TS2_conf_1_TS**	-1996.683110	-1996.222183	0.408485	-1996.245926	0.085552	0.080502	-1996.331479	-1996.326428	-171.32
**TS2_conf_2_TS**	-1996.681746	-1996.221848	0.408659	-1996.244439	0.085123	0.080241	-1996.329561	-1996.324680	-163.48
**TS2_conf_3_TS**	-1996.681744	-1996.221848	0.408661	-1996.244434	0.085141	0.080250	-1996.329575	-1996.324684	-163.53
**TS2_conf_4_TS**	-1996.665089	-1996.205985	0.407909	-1996.228222	0.087183	0.081452	-1996.315406	-1996.309675	-204.94
**TS2_conf_5_TS**	-1996.665059	-1996.206309	0.408010	-1996.228184	0.085968	0.080812	-1996.314152	-1996.308996	-211.50

**TS2_conf_6_TS**	-1996.672019	-1996.213805	0.408182	-1996.234978	0.086128	0.080954	-1996.321106	-1996.315932	-124.11
**TS2_conf_7_TS**	-1996.673077	-1996.214257	0.408047	-1996.236204	0.085901	0.080861	-1996.322105	-1996.317065	-119.98
**TS2_conf_8_TS**	-1996.680352	-1996.220792	0.409303	-1996.242703	0.083582	0.079370	-1996.326285	-1996.322073	-168.57
**TS3_conf_10_ts**	-1996.668718	-1996.208286	0.406538	-1996.232647	0.088576	0.082600	-1996.321222	-1996.315247	-170.00
**TS3_conf_1_ts**	-1996.674513	-1996.213359	0.406363	-1996.238597	0.088684	0.082585	-1996.327281	-1996.321182	-100.38
**TS3_conf_2_ts**	-1996.674242	-1996.213860	0.406671	-1996.238196	0.086874	0.081747	-1996.325070	-1996.319943	-109.04
**TS3_conf_5_ts**	-1996.669469	-1996.208930	0.405872	-1996.233843	0.089624	0.083114	-1996.323467	-1996.316956	-70.16
**TS3_conf_6_ts**	-1996.668955	-1996.208264	0.406231	-1996.233079	0.088923	0.082760	-1996.322002	-1996.315839	-123.43
**TS3_conf_7_ts**	-1996.672004	-1996.210586	0.407197	-1996.235565	0.086835	0.081564	-1996.322399	-1996.317128	-169.45
**TS3_conf_8_ts**	-1996.669221	-1996.208753	0.406418	-1996.233328	0.087347	0.082030	-1996.320675	-1996.315358	-116.40
**TS4_conf_0**	-1821.729232	-1821.322583	0.271765	-1821.434307	0.075867	0.070753	-1821.510175	-1821.505061	-175.50
**TS4_conf_1**	-1821.728220	-1821.322617	0.271320	-1821.433493	0.077461	0.071501	-1821.510954	-1821.504994	-185.90
**TS4_conf_10**	-1821.722051	-1821.317322	0.272005	-1821.426996	0.074992	0.070245	-1821.501988	-1821.497241	-189.38
**TS4_conf_7**	-1821.727376	-1821.321336	0.271504	-1821.432583	0.076058	0.070879	-1821.508641	-1821.503462	-203.91
**TS5_conf_10_TS**	-772.124672	-771.868094	0.381476	-771.721330	0.068700	0.065747	-771.790030	-771.787077	-199.14
**TS5_conf_1_TS**	-772.127206	-771.870048	0.379869	-771.724874	0.070476	0.067193	-771.795350	-771.792067	-79.23
**TS5_conf_2_TS**	-772.127619	-771.869825	0.379677	-771.725223	0.071731	0.067917	-771.796955	-771.793140	-117.85
**TS5_conf_3_TS**	-772.126530	-771.869436	0.379773	-771.724135	0.070815	0.067436	-771.794951	-771.791571	-129.14
**TS5_conf_4_TS**	-772.127132	-771.868674	0.379782	-771.724738	0.071109	0.067603	-771.795847	-771.792341	-74.98
**TS5_conf_5_TS**	-772.125884	-771.869359	0.380933	-771.722853	0.069149	0.066275	-771.792002	-771.789128	-186.95
**TS5_conf_6_TS**	-772.126404	-771.869021	0.380236	-771.723875	0.070222	0.066791	-771.794097	-771.790667	-90.53
**TS5_conf_7_TS**	-772.124879	-771.868049	0.379853	-771.722528	0.071116	0.067403	-771.793644	-771.789932	-75.21
**TS5_conf_8_TS**	-772.126448	-771.868748	0.379606	-771.724159	0.071351	0.067705	-771.795510	-771.791865	-96.19
**TS5_conf_9_TS**	-772.126349	-771.869681	0.380448	-771.723548	0.070738	0.067164	-771.794286	-771.790712	-215.11
**TS_S1_conf_1_ts**	-772.153025	-771.897200	0.381284	-771.749553	0.069595	0.066541	-771.819148	-771.816093	-272.44
**TS_S1_conf_2_ts**	-772.153652	-771.897698	0.380860	-771.750406	0.070664	0.067233	-771.821070	-771.817638	-290.75
**TS_S1_conf_3_ts**	-772.152530	-771.896798	0.381401	-771.748978	0.069332	0.066459	-771.818310	-771.815437	-262.06
**TS_S1_conf_4_ts**	-772.150949	-771.895186	0.380336	-771.748022	0.071626	0.067699	-771.819648	-771.815721	-296.62
**TS_S1_conf_5_ts**	-772.149920	-771.894682	0.380713	-771.746849	0.070524	0.067088	-771.817373	-771.813937	-281.63
**TS_S1_conf_6_ts**	-772.146702	-771.890913	0.381851	-771.742817	0.068997	0.066194	-771.811814	-771.809012	-266.60
**TS_S1_conf_7_ts**	-772.146077	-771.890186	0.381113	-771.742694	0.070050	0.066786	-771.812744	-771.809480	-278.38
**TS_S1_conf_8_ts**	-772.147660	-771.892374	0.381165	-771.744354	0.069125	0.066419	-771.813479	-771.810773	-280.58

**TS_S1_conf_9_ts**	-772.146444	-771.891141	0.381567	-771.742840	0.068786	0.066096	-771.811625	-771.808935	-259.77
**TS_S2**	-1937.916230	-1937.452696	0.339498	-1937.550131	0.083531	0.077571	-1937.633662	-1937.627702	-187.03
**TS_S3_conf_10_ts**	-3046.702581	-3046.096212	0.309439	-3046.362364	0.093948	0.086745	-3046.456312	-3046.449109	-150.18
**TS_S3_conf_1_ts**	-3046.704841	-3046.096929	0.309152	-3046.364698	0.094213	0.086866	-3046.458911	-3046.451564	-121.71
**TS_S3_conf_2_ts**	-3046.704860	-3046.096612	0.309286	-3046.364639	0.094018	0.086740	-3046.458657	-3046.451378	-125.52
**TS_S3_conf_3_ts**	-3046.702478	-3046.096550	0.310364	-3046.361740	0.092253	0.085504	-3046.453993	-3046.447244	-86.44
**TS_S3_conf_4_ts**	-3046.702147	-3046.096075	0.309757	-3046.361666	0.092608	0.086045	-3046.454274	-3046.447711	-129.15
**TS_S3_conf_6_ts**	-3046.699014	-3046.092883	0.309976	-3046.358413	0.094698	0.086798	-3046.453110	-3046.445211	-103.63
**TS_S3_conf_9_ts**	-3046.694151	-3046.089278	0.309831	-3046.353989	0.092737	0.085685	-3046.446725	-3046.439673	-141.51
**int10_conf_1**	-1996.686035	-1996.225081	0.405676	-1996.249669	0.092538	0.085394	-1996.342208	-1996.335063	
**int10_conf_10**	-1996.680941	-1996.220372	0.406807	-1996.243686	0.091343	0.084687	-1996.335029	-1996.328374	
**int10_conf_2**	-1996.687426	-1996.220930	0.406982	-1996.249928	0.090526	0.084451	-1996.340454	-1996.334379	
**int10_conf_3**	-1996.686816	-1996.223183	0.405214	-1996.250695	0.093165	0.085945	-1996.343860	-1996.336640	
**int10_conf_4**	-1996.684253	-1996.223347	0.405830	-1996.247812	0.092335	0.085231	-1996.340147	-1996.333044	
**int10_conf_5**	-1996.682893	-1996.221484	0.407376	-1996.245463	0.088136	0.083184	-1996.333600	-1996.328647	
**int10_conf_6**	-1996.686247	-1996.223267	0.405397	-1996.250094	0.093516	0.085705	-1996.343611	-1996.335800	
**int10_conf_7**	-1996.683691	-1996.220822	0.405046	-1996.247660	0.093232	0.086126	-1996.340892	-1996.333786	
**int10_conf_8**	-1996.680450	-1996.218308	0.405162	-1996.244306	0.093211	0.086025	-1996.337517	-1996.330332	
**int10_conf_9**	-1996.682487	-1996.214030	0.406645	-1996.245368	0.090878	0.084500	-1996.336246	-1996.329868	
**int11_conf_0**	-1996.689113	-1996.225469	0.410126	-1996.249974	0.087124	0.081456	-1996.337098	-1996.331430	
**int11_conf_1**	-1996.688260	-1996.226098	0.410001	-1996.249180	0.086999	0.081465	-1996.336179	-1996.330645	
**int11_conf_10**	-1996.689588	-1996.226955	0.410023	-1996.250370	0.087612	0.081716	-1996.337982	-1996.332086	
**int11_conf_2**	-1996.690390	-1996.226934	0.410152	-1996.251049	0.087799	0.081765	-1996.338848	-1996.332814	
**int11_conf_3**	-1996.686195	-1996.224615	0.410935	-1996.246546	0.085926	0.080651	-1996.332472	-1996.327197	
**int11_conf_5**	-1996.687424	-1996.226006	0.410030	-1996.248405	0.085718	0.080852	-1996.334122	-1996.329256	
**int11_conf_6**	-1996.686379	-1996.223778	0.410129	-1996.247203	0.087036	0.081356	-1996.334239	-1996.328559	
**int11_conf_7**	-1996.690502	-1996.226918	0.410719	-1996.250793	0.087030	0.081261	-1996.337823	-1996.332054	
**int11_conf_8**	-1996.681941	-1996.220923	0.410014	-1996.242840	0.087363	0.081567	-1996.330203	-1996.324407	
**int11_conf_9**	-1996.684576	-1996.223366	0.410882	-1996.244936	0.085469	0.080484	-1996.330406	-1996.325420	
**int12_conf_0**	-1996.710478	-1996.248945	0.409863	-1996.271495	0.087439	0.081675	-1996.358934	-1996.353170	
**int12_conf_1**	-1996.711835	-1996.252029	0.410557	-1996.272410	0.086380	0.080967	-1996.358790	-1996.353378	
**int12_conf_10**	-1996.703659	-1996.243767	0.409824	-1996.264985	0.086729	0.081057	-1996.351715	-1996.346043	

**int12_conf_2**	-1996.710911	-1996.249469	0.409900	-1996.272029	0.086463	0.081106	-1996.358492	-1996.353135
**int12_conf_3**	-1996.710144	-1996.249993	0.409672	-1996.271464	0.087276	0.081301	-1996.358740	-1996.352765
**int12_conf_4**	-1996.708625	-1996.248452	0.410355	-1996.269618	0.085010	0.080360	-1996.354628	-1996.349978
**int12_conf_5**	-1996.706950	-1996.245584	0.410074	-1996.267933	0.086700	0.081066	-1996.354633	-1996.348998
**int12_conf_6**	-1996.694977	-1996.234301	0.410166	-1996.255814	0.086035	0.080980	-1996.341850	-1996.336794
**int12_conf_7**	-1996.706315	-1996.245102	0.409702	-1996.267568	0.087043	0.081450	-1996.354612	-1996.349019
**int12_conf_8**	-1996.704610	-1996.245058	0.409872	-1996.265957	0.085855	0.080634	-1996.351812	-1996.346591
**int12_conf_9**	-1996.712515	-1996.252591	0.410741	-1996.273015	0.085625	0.080544	-1996.358640	-1996.353558
**int13_conf_0**	-1822.202635	-1821.787526	0.286701	-1821.893102	0.073454	0.069379	-1821.966556	-1821.962480
**int13_conf_1**	-1822.202637	-1821.787526	0.286699	-1821.893104	0.073471	0.069387	-1821.966576	-1821.962492
**int13_conf_2**	-1822.202636	-1821.787526	0.286699	-1821.893103	0.073468	0.069386	-1821.966571	-1821.962489
**int14_conf_0**	-1821.759018	-1821.348048	0.273199	-1821.462728	0.075330	0.070238	-1821.538057	-1821.532965
**int14_conf_1**	-1821.758883	-1821.348040	0.273093	-1821.462636	0.075993	0.070552	-1821.538630	-1821.533188
**int14_conf_10**	-1821.753966	-1821.343906	0.273569	-1821.457615	0.073424	0.069126	-1821.531039	-1821.526741
**int14_conf_4**	-1821.759194	-1821.348455	0.273317	-1821.462883	0.074259	0.069687	-1821.537141	-1821.532569
**int14_conf_7**	-1821.753541	-1821.343249	0.273422	-1821.457250	0.074440	0.069682	-1821.531690	-1821.526931
**int7_conf_1**	-771.688585	-771.437034	0.364725	-771.299670	0.077524	0.071978	-771.377194	-771.371649
**int7_conf_10**	-771.678418	-771.427552	0.365220	-771.289247	0.075725	0.071082	-771.364972	-771.360330
**int7_conf_2**	-771.686765	-771.435290	0.364845	-771.297682	0.077928	0.072169	-771.375609	-771.369850
**int7_conf_3**	-771.683484	-771.431740	0.364589	-771.294588	0.078423	0.072454	-771.373012	-771.367042
**int7_conf_4**	-771.680269	-771.429289	0.365022	-771.291261	0.075937	0.071201	-771.367198	-771.362462
**int7_conf_6**	-771.685353	-771.434081	0.365315	-771.296086	0.076413	0.071309	-771.372499	-771.367395
**int7_conf_7**	-771.682734	-771.431296	0.364574	-771.293825	0.077980	0.072384	-771.371805	-771.366209
**int7_conf_8**	-771.684233	-771.433031	0.364924	-771.295325	0.076725	0.071353	-771.372050	-771.366678
**int7_conf_9**	-771.684829	-771.433762	0.365100	-771.295798	0.076014	0.071098	-771.371811	-771.366896
**int8_conf_0**	-772.162192	-771.905485	0.379014	-771.758974	0.077100	0.071779	-771.836074	-771.830753
**int8_conf_1**	-772.162193	-771.905485	0.379014	-771.758975	0.077101	0.071779	-771.836076	-771.830754
**int8_conf_10**	-772.159221	-771.903161	0.379747	-771.755394	0.076291	0.071574	-771.831685	-771.826968
**int8_conf_2**	-772.162648	-771.906438	0.379416	-771.759235	0.075831	0.071050	-771.835066	-771.830285
**int8_conf_3**	-772.160411	-771.903915	0.379395	-771.756944	0.077030	0.071589	-771.833974	-771.828533
**int8_conf_4**	-772.160428	-771.903876	0.379273	-771.757056	0.077101	0.071625	-771.834157	-771.828680
**int8_conf_5**	-772.162300	-771.906449	0.379817	-771.758605	0.076727	0.071493	-771.835332	-771.830098

**int8_conf_6**	-772.161888	-771.906018	0.379340	-771.758488	0.076016	0.071378	-771.834504	-771.829866
**int8_conf_7**	-772.157112	-771.900200	0.379355	-771.753630	0.076501	0.071498	-771.830131	-771.825128
**int8_conf_8**	-772.158306	-771.902287	0.379383	-771.754932	0.075783	0.071125	-771.830716	-771.826058
**int8_conf_9**	-772.156417	-771.900299	0.379389	-771.753013	0.075923	0.071174	-771.828936	-771.824187
**int9_conf_1**	-1996.225101	-1995.763903	0.391717	-1995.802223	0.093907	0.086286	-1995.896130	-1995.888510
**int9_conf_10**	-1996.231041	-1995.764513	0.391997	-1995.808211	0.092578	0.085464	-1995.900789	-1995.893675
**int9_conf_2**	-1996.228183	-1995.766091	0.392156	-1995.805179	0.093819	0.085963	-1995.898998	-1995.891143
**int9_conf_3**	-1996.226946	-1995.765915	0.392393	-1995.803848	0.092496	0.085359	-1995.896344	-1995.889207
**int9_conf_4**	-1996.228771	-1995.766487	0.392417	-1995.805583	0.091891	0.085181	-1995.897474	-1995.890764
**int9_conf_5**	-1996.227940	-1995.765380	0.391777	-1995.805047	0.094081	0.086302	-1995.899128	-1995.891350
**int9_conf_6**	-1996.226848	-1995.765097	0.392360	-1995.803780	0.092343	0.085300	-1995.896123	-1995.889080
**int9_conf_7**	-1996.226536	-1995.765779	0.392262	-1995.803353	0.093846	0.086066	-1995.897198	-1995.889419
**int9_conf_8**	-1996.224610	-1995.765044	0.392056	-1995.801625	0.093838	0.086032	-1995.895464	-1995.887657
**int9_conf_9**	-1996.224649	-1995.763298	0.392657	-1995.801263	0.092918	0.085411	-1995.894181	-1995.886674
**int_S1_conf_1**	-772.139542	-771.882599	0.382344	-771.734715	0.070946	0.067224	-771.805661	-771.801939
**int_S1_conf_10**	-772.129878	-771.874258	0.382774	-771.724797	0.069582	0.066533	-771.794379	-771.791330
**int_S1_conf_2**	-772.138666	-771.881585	0.382266	-771.733874	0.071594	0.067565	-771.805468	-771.801439
**int_S1_conf_4**	-772.137502	-771.881058	0.383091	-771.732238	0.069376	0.066329	-771.801614	-771.798567
**int_S1_conf_5**	-772.138500	-771.881658	0.382655	-771.733563	0.069716	0.066560	-771.803279	-771.800123
**int_S1_conf_6**	-772.136787	-771.880312	0.382218	-771.732007	0.070887	0.067258	-771.802894	-771.799266
**int_S1_conf_7**	-772.136027	-771.879871	0.382267	-771.731312	0.069861	0.066897	-771.801173	-771.798209
**int_S1_conf_8**	-772.135071	-771.879233	0.382287	-771.730366	0.069853	0.066711	-771.800219	-771.797077
**int_S1_conf_9**	-772.130569	-771.875426	0.382955	-771.725423	0.068992	0.066181	-771.794415	-771.791604
**int_S2_conf_1**	-772.138890	-771.880139	0.383772	-771.733083	0.068944	0.066128	-771.802027	-771.799211
**int_S2_conf_10**	-772.133973	-771.875415	0.383752	-771.728319	0.068701	0.065838	-771.797020	-771.794157
**int_S2_conf_2**	-772.137978	-771.879177	0.383638	-771.732399	0.068706	0.065897	-771.801105	-771.798296
**int_S2_conf_3**	-772.138429	-771.880081	0.383619	-771.732832	0.068552	0.065922	-771.801384	-771.798754
**int_S2_conf_4**	-772.141082	-771.882886	0.383317	-771.735719	0.068489	0.065991	-771.804208	-771.801710
**int_S2_conf_5**	-772.139076	-771.880084	0.382914	-771.733865	0.069762	0.066739	-771.803627	-771.800604
**int_S2_conf_6**	-772.139076	-771.880084	0.382914	-771.733865	0.069762	0.066739	-771.803627	-771.800603
**int_S2_conf_7**	-772.136918	-771.878671	0.383638	-771.731360	0.068585	0.065783	-771.799945	-771.797143
**int_S2_conf_8**	-772.137497	-771.878862	0.383421	-771.732007	0.068782	0.066132	-771.800789	-771.798139

**int_S2_conf_9**	-772.132351	-771.874445	0.383949	-771.726700	0.067675	0.065211	-771.794375	-771.791911
**ipr_amine**	-174.465191	-174.414507	0.117697	-174.340413	0.033049	0.033057	-174.373462	-174.373469
**methanol**	-115.725004	-115.677615	0.050153	-115.670296	0.025463	0.025465	-115.695760	-115.695761
**protDBA**	-597.706635	-597.493560	0.264394	-597.426844	0.057193	0.053989	-597.484037	-597.480833

7.7.7 Structural Data

```
31
3h_conf_1        Eopt   -597.492286
C    1.486483    2.469200   -0.892976
C    0.993095    1.596571    0.256965
C    1.593080    2.016127    1.596940
N    1.294658    0.177065   -0.001655
C    2.583759   -0.186756   -0.088776
C    2.966796   -1.496186   -0.243285
C    1.983086   -2.480573   -0.292125
C    0.661079   -2.093969   -0.213091
C    0.316631   -0.747106   -0.086144
C   -1.108866   -0.388843   -0.055188
C   -1.932799   -0.967813    0.910423
C   -3.284911   -0.680939    0.930391
C   -3.833261    0.159533   -0.025469
C   -3.024610    0.716772   -1.003358
C   -1.667893    0.452303   -1.019228
H    1.113454    3.479975   -0.755623
H    1.122466    2.101181   -1.849366
H    2.571439    2.517383   -0.930749
H   -0.093814    1.678936    0.327776
H    1.243548    3.015338    1.840228
H    2.678987    2.039547    1.573252
H    1.273309    1.344508    2.390506
H    3.316649    0.601659   -0.027123
H    4.012621   -1.745268   -0.316090
H    2.247837   -3.520687   -0.397561
H   -0.134125   -2.820598   -0.267899
H   -1.504324   -1.619474    1.657141
H   -3.911866   -1.120495    1.690001
H   -4.890084    0.373930   -0.013587
H   -3.450333    1.357757   -1.759203
H   -1.048385    0.866924   -1.800596
31
3h_conf_4        Eopt   -597.487002
C    0.067415    2.115737    1.396800
C    0.864083    1.878600    0.119666
C    0.117227    2.263447   -1.153416
N    1.432908    0.479735    0.033783
C    2.782409    0.368290    0.021358
C    3.417450   -0.856051   -0.051308
C    2.638277   -2.006933   -0.108548
C    1.253990   -1.873687   -0.099441
C    0.657211   -0.618872   -0.033531
C   -0.828515   -0.521637   -0.030234
C   -1.527388   -0.764477    1.156630
C   -2.920008   -0.753948    1.153272
C   -3.614769   -0.521680   -0.034772
C   -2.916406   -0.314106   -1.223743
C   -1.522418   -0.321401   -1.225933
H   -0.950644    1.726781    1.328827
H    0.007321    3.197074    1.543448
H    0.567145    1.682093    2.266660
H    1.760685    2.493437    0.178201
H   -0.910774    1.894771   -1.147335
H    0.631332    1.895819   -2.044804
H    0.085615    3.354490   -1.204534
H    3.339475    1.293843    0.073591
H    4.499292   -0.889669   -0.056956
H    3.096344   -2.988133   -0.161058
H    0.604491   -2.739401   -0.147819
H   -0.982294   -0.945463    2.078565
H   -3.460762   -0.929600    2.077154
H   -4.699819   -0.514404   -0.035366
H   -3.453289   -0.152727   -2.152462
H   -0.975821   -0.176741   -2.153394
30
DBA              Eopt   -597.044441
N    0.084679   -1.303668    0.814527
H    0.710149   -2.046334    1.053763
C   -1.274691   -1.840018    0.655334
H   -1.590213   -2.290311    1.573257
H   -1.279388   -2.574784   -0.122481
C    0.518154   -0.685043   -0.446545
H   -0.151098    0.109611   -0.702526
H    0.513455   -1.419809   -1.224361
C   -2.237914   -0.696312    0.286911
C   -2.856502   -0.680873   -0.963522
C   -2.492015    0.324077    1.203311
C   -3.728457    0.355117   -1.297648
H   -2.655369   -1.485066   -1.686059
C   -3.364973    1.359933    0.869609
H   -2.004695    0.311970    2.188959
C   -3.983082    1.375685   -0.380684
H   -4.215586    0.367686   -2.283469
H   -3.565448    2.164111    1.592506
H   -4.670670    2.192390   -0.644332
C    1.942257   -0.123154   -0.279772
```

C 3.042525 -0.977674 -0.355124
C 2.132175 1.240034 -0.053504
C 4.332347 -0.469139 -0.203557
H 2.892419 -2.052456 -0.532753
C 3.422380 1.749038 0.097148
H 1.265114 1.913699 0.005736
C 4.522405 0.894709 0.022299
H 5.199605 -1.142718 -0.262312
H 3.571839 2.823961 0.275131
H 5.539481 1.295450 0.141715
6
KHSO3 Eopt -1224.308754
O 0.094428 -1.190288 -0.467558
S 1.005070 0.009061 -0.488262
O 0.194417 1.271244 -0.350469
O 1.768388 -0.088312 1.035075
H 1.068867 -0.044658 1.710497
K -1.768834 -0.002182 0.229753
5
KSO3minus Eopt -1223.827213
O 2.498123 -0.548004 -0.285344
S 1.200462 0.000080 0.363431
O 0.990053 1.419145 -0.225396
O 0.041745 -0.871250 -0.187594
K -2.497198 -0.000022 -0.012012
9
MeOSidepdt Eopt -1263.588093
O -0.213193 -1.221042 -0.260157
S 0.636365 -0.001011 -0.539172
O -0.210477 1.221402 -0.261431
O 1.717152 0.000301 0.756132
K -2.581701 0.000498 0.252498
C 3.057726 0.000170 0.258376
H 3.696298 -0.503516 0.953665
H 3.088094 -0.505014 -0.684368
H 3.391874 1.008943 0.133384
10
NH2Tf Eopt -941.957713
N -1.448963 1.333823 -0.000114
H -1.333532 1.861223 0.860117
H -1.333547 1.861086 -0.860431
S -0.855084 -0.176460 0.000014
O -1.165598 -0.796328 1.271756
O -1.165602 -0.796567 -1.271617
C 1.001285 0.031704 -0.000003
F 1.587544 -1.160164 0.000120
F 1.380284 0.708929 1.083604
F 1.380290 0.708705 -1.083746
9
NHtfminus Eopt -941.501268
N -1.428271 0.921816 -1.058645
H -1.313563 1.911713 -0.860989
S -0.868894 -0.098879 0.067838
O -1.218349 0.418491 1.374954
O -1.173949 -1.453868 -0.343617
C 0.980506 0.021730 -0.015782
F 1.515396 -0.792068 0.884733
F 1.360556 1.269818 0.235832
F 1.398394 -0.325498 -1.228332
49
TS1_conf_0 Eopt -1995.733021
C 5.625446 2.806572 -1.450313
C 4.733375 2.115252 -0.425388
C 5.528684 1.183980 0.489030
N 3.673538 1.384730 -1.123879
C 2.441450 1.209117 -0.635028
C 1.419874 0.511347 -1.233134
C 0.158059 0.407114 -0.595495
C -0.997267 -0.043882 -1.310071
C -2.292639 0.218319 -0.948729
C -2.646755 0.963323 0.293835
C -2.287544 0.479290 1.555487
C -2.628995 1.204423 2.698712
C -3.321869 2.409339 2.590043
C -3.681171 2.892940 1.330059
C -3.350339 2.170192 0.186980
N -3.337492 -0.090140 -1.781311
C -4.655445 -0.508430 -1.278117
C -5.630414 -0.539311 -2.448387
C -4.570205 -1.860308 -0.568023
H 6.114525 2.065355 -2.092190
H 6.405865 3.377474 -0.942012
H 5.046286 3.487059 -2.079284
H 4.232473 2.874967 0.184529
H 4.871586 0.713057 1.225766
H 6.014337 0.399158 -0.100876
H 6.301801 1.744050 1.022727
H 3.919521 0.925515 -1.994036
H 2.261058 1.693051 0.324730
H 1.580689 0.008379 -2.183526
H -0.825145 -0.596871 -2.231796
H -1.746948 -0.462188 1.643107

H	-2.351681	0.822763	3.676535
H	-3.582888	2.970314	3.481965
H	-4.219334	3.831120	1.238731
H	-3.631961	2.537691	-0.796372
H	-4.985677	0.249961	-0.562697
H	-5.696121	0.440833	-2.927704
H	-6.625574	-0.827242	-2.101018
H	-5.309885	-1.273131	-3.196917
H	-4.235897	-2.635075	-1.266932
H	-3.859750	-1.817800	0.263962
H	-5.548575	-2.148972	-0.172778
H	-3.057439	-0.590710	-2.619907
H	-0.002449	1.023629	0.286564
S	0.687578	-1.597489	0.854358
O	-0.370161	-2.190355	1.776238
O	1.939529	-1.250776	1.674470
O	1.114851	-2.667558	-0.160184
K	3.738862	-2.482149	0.144898

49

TS1_conf_10_TS Eopt -1995.734019

C	4.726681	-2.432063	1.391634
C	4.479939	-2.911202	-0.039847
C	5.685043	-3.650911	-0.607406
N	4.160941	-1.784048	-0.916244
C	2.980566	-1.162204	-0.954907
C	1.849710	-1.502258	-0.243071
C	0.667097	-0.748149	-0.408842
C	-0.561601	-1.153611	0.119722
C	-1.784747	-0.498730	-0.118088
C	-3.033094	-1.234631	0.229476
C	-3.328439	-2.455115	-0.384518
C	-4.497163	-3.137981	-0.053320
C	-5.360679	-2.618852	0.912457
C	-5.049210	-1.419172	1.553588
C	-3.889499	-0.726648	1.211179
N	-1.843249	0.741706	-0.585481
C	-2.948110	1.461872	-1.243270
C	-3.901210	0.578088	-2.045599
C	-3.694336	2.388408	-0.280943
H	4.881229	-3.287821	2.054664
H	3.878625	-1.851714	1.765185
H	5.619604	-1.799730	1.424540
H	3.612504	-3.581719	-0.056631
H	5.928446	-4.506759	0.026111
H	5.485065	-4.011998	-1.619503
H	6.559503	-2.991234	-0.634842
H	4.915173	-1.394824	-1.466385
H	2.939415	-0.296937	-1.610335
H	1.854629	-2.317374	0.474059
H	-0.597081	-2.050509	0.730053
H	-2.652746	-2.851656	-1.136988
H	-4.732946	-4.075044	-0.547380
H	-6.269001	-3.153251	1.172001
H	-5.706802	-1.024056	2.321213
H	-3.633512	0.198904	1.717669
H	-2.420524	2.101879	-1.961048
H	-3.351676	-0.147938	-2.652062
H	-4.480097	1.218235	-2.716983
H	-4.604074	0.039297	-1.405947
H	-2.991627	2.973169	0.318684
H	-4.320129	3.079506	-0.853318
H	-4.343106	1.819147	0.389584
H	-0.959433	1.319202	-0.538411
H	0.663319	0.014224	-1.185723
S	1.343925	1.548377	0.574070
O	0.303675	2.311600	-0.329575
O	2.684671	1.743726	-0.157113
O	1.425794	2.390204	1.864030
K	1.981042	4.467162	0.108976

49

TS1_conf_4_TS Eopt -1995.736540

C	-5.560408	-1.287808	-0.252742
C	-4.971990	-2.588979	0.293668
C	-4.984914	-2.627023	1.821119
N	-3.612696	-2.798586	-0.228822
C	-2.590268	-1.992394	0.049849
C	-1.322160	-2.076381	-0.486923
C	-0.331038	-1.155514	-0.096856
C	1.009876	-1.290961	-0.475584
C	2.053461	-0.475366	-0.005173
C	3.452467	-0.943384	-0.217666
C	3.818427	-2.248610	0.125596
C	5.118180	-2.696783	-0.106873
C	6.054618	-1.849475	-0.698240
C	5.690604	-0.549463	-1.055043
C	4.397146	-0.096744	-0.811464
N	1.836739	0.701253	0.573140
C	2.749714	1.441858	1.447206
C	2.885292	2.878478	0.947862
C	2.200256	1.396771	2.874656
H	-4.940189	-0.435679	0.046361
H	-6.573598	-1.141503	0.131820

H	-5.600303	-1.311851	-1.345124
H	-5.555413	-3.434172	-0.080435
H	-4.541358	-3.554858	2.190901
H	-4.430831	-1.782125	2.242308
H	-6.014588	-2.562451	2.182054
H	-3.477772	-3.530020	-0.915397
H	-2.803247	-1.183851	0.745204
H	-1.092799	-2.825957	-1.240883
H	1.280588	-2.096669	-1.150387
H	3.085428	-2.904952	0.585372
H	5.397819	-3.707348	0.172934
H	7.064709	-2.200689	-0.883654
H	6.413114	0.109784	-1.525202
H	4.107575	0.910873	-1.096737
H	3.724262	0.948884	1.427001
H	3.297124	2.908860	-0.064543
H	3.542985	3.447896	1.610087
H	1.903019	3.362050	0.933305
H	2.860798	1.944705	3.552640
H	1.207469	1.858027	2.911476
H	2.117157	0.363997	3.223942
H	0.875829	1.133640	0.483690
H	-0.565214	-0.487287	0.729268
S	-1.290295	1.096864	-0.936728
O	-1.401217	2.087233	-2.115439
O	-0.531832	1.901140	0.183304
O	-2.717311	0.935706	-0.375726
K	-2.579939	3.730539	-0.207686

49

TS1_conf_8_TS Eopt -1995.732161

C	5.566178	-1.076769	0.373182
C	4.822669	-2.381096	0.666565
C	5.089847	-3.441894	-0.400345
N	3.383076	-2.143335	0.833926
C	2.572817	-1.743814	-0.158145
C	1.258183	-1.383614	-0.031892
C	0.522256	-1.011069	-1.191798
C	-0.855517	-0.834023	-1.308436
C	-1.748187	-0.559340	-0.241630
C	-3.181133	-0.927131	-0.405448
C	-4.194594	0.001992	-0.138247
C	-5.528904	-0.338604	-0.340808
C	-5.862034	-1.613143	-0.803495
C	-4.856974	-2.541057	-1.074913
C	-3.519643	-2.196539	-0.885512
N	-1.321643	0.060573	0.842608
C	-1.977541	0.155295	2.147292
C	-1.122153	-0.596999	3.169177
C	-2.138446	1.624036	2.534980
H	5.379654	-0.339448	1.158232
H	5.236624	-0.654385	-0.582243
H	6.642572	-1.259473	0.311520
H	5.159318	-2.770984	1.630909
H	6.157503	-3.674525	-0.427827
H	4.536527	-4.358880	-0.182908
H	4.803574	-3.088432	-1.396033
H	3.022085	-2.116282	1.778585
H	3.032265	-1.714270	-1.145983
H	0.796538	-1.361433	0.950281
H	-1.292285	-0.901323	-2.298946
H	-3.932557	0.997871	0.207406
H	-6.307791	0.390311	-0.142033
H	-6.903016	-1.879382	-0.956690
H	-5.112861	-3.531764	-1.436267
H	-2.731148	-2.912367	-1.098423
H	-2.957216	-0.324159	2.081240
H	-1.031793	-1.652283	2.897183
H	-1.574020	-0.528827	4.162571
H	-0.118329	-0.160271	3.216477
H	-1.157067	2.107413	2.582061
H	-2.750023	2.163929	1.807188
H	-2.611871	1.703370	3.517111
H	-0.394048	0.600503	0.730596
H	1.043460	-1.164765	-2.134606
S	1.226429	1.478517	-1.125302
O	0.770527	1.550324	0.382822
O	0.242667	2.410497	-1.860275
O	2.588923	2.201991	-1.125234
K	1.111218	4.275424	0.020949

49

TS1_conf_9_TS Eopt -1995.734107

C	5.883614	0.105348	-1.378072
C	5.506959	1.106721	-0.285821
C	6.732318	1.630695	0.454998
N	4.577804	0.507652	0.673074
C	3.245460	0.536593	0.535174
C	2.343540	-0.145512	1.310114
C	0.930544	-0.061939	1.131683
C	0.256941	0.909580	0.391302
C	-1.147993	1.042853	0.358848
C	-1.703641	2.311275	-0.193494
C	-1.244223	3.544365	0.279025

C	-1.736510	4.730283	-0.264387
C	-2.675213	4.690230	-1.294939
C	-3.125456	3.461034	-1.780335
C	-2.645884	2.276275	-1.228636
N	-1.970138	0.086140	0.761313
C	-3.390648	0.212340	1.096721
C	-4.204160	-0.778834	0.266754
C	-3.558268	-0.045964	2.595258
H	6.403611	-0.753512	-0.940866
H	6.546515	0.573421	-2.111385
H	4.990453	-0.256110	-1.895178
H	4.973956	1.949650	-0.739394
H	7.423783	2.099728	-0.249120
H	6.447845	2.366442	1.211153
H	7.261146	0.807660	0.948407
H	4.972517	-0.117836	1.367508
H	2.899930	1.180439	-0.272306
H	2.715047	-0.830702	2.066977
H	0.811404	1.647246	-0.177557
H	-0.510671	3.570099	1.079564
H	-1.384707	5.684008	0.115515
H	-3.053079	5.613671	-1.722051
H	-3.847226	3.425309	-2.589913
H	-2.985959	1.317873	-1.611055
H	-3.709432	1.232932	0.873317
H	-5.263433	-0.708811	0.528219
H	-3.863649	-1.800523	0.465695
H	-4.094418	-0.584276	-0.803516
H	-3.208353	-1.053046	2.846497
H	-2.983755	0.677892	3.179245
H	-4.612089	0.033090	2.876523
H	-1.576432	-0.889944	0.882770
H	0.336742	-0.633556	1.840505
S	0.303236	-2.249965	-0.100140
O	-0.976163	-2.397413	0.800883
O	0.900902	-3.667722	-0.153939
O	-0.246923	-1.953506	-1.507481
K	-1.685368	-4.300589	-1.088888

50

TS2_conf_0 Eopt -1996.216823

C	0.961941	1.137149	1.410932
C	-0.040945	1.985234	0.687626
N	0.075628	2.233447	-0.586410
C	-0.750692	3.165828	-1.377278
C	-1.468162	4.181931	-0.501228
C	0.158013	3.835225	-2.403574
H	-0.613589	2.687174	1.282635
H	-1.492976	2.554820	-1.907163
H	-2.184866	3.721129	0.184751
H	-0.750966	4.772570	0.078042
H	-2.028090	4.861270	-1.147040
H	-0.438552	4.458692	-3.072860
H	0.897068	4.465540	-1.900033
H	0.684519	3.091279	-3.008176
N	-1.665533	0.568008	0.827328
C	-2.518110	0.339213	2.036972
C	-1.203197	-0.652324	0.209503
C	0.091109	-0.993678	0.214441
H	0.365501	-1.924567	-0.275341
C	1.241545	-0.251628	0.821347
C	-3.132035	1.668419	2.455769
H	-3.326571	-0.346688	1.745707
C	-1.725153	-0.304664	3.165999
H	-3.809363	1.511776	3.298641
H	-2.359800	2.376635	2.774495
H	-3.710510	2.116313	1.641181
H	-2.415527	-0.608283	3.956818
H	-1.185959	-1.194939	2.829294
H	-1.011586	0.400287	3.602060
H	0.739311	1.653587	-1.119654
H	-2.259965	1.055103	0.153153
C	-4.153513	-3.235979	-1.549504
C	-3.131212	-3.767506	-0.764227
C	-2.180475	-2.926122	-0.189586
C	-2.233416	-1.542085	-0.398584
C	-3.272067	-1.016224	-1.178739
C	-4.221528	-1.857499	-1.753771
H	-4.896336	-3.889998	-1.994518
H	-3.078314	-4.837126	-0.588193
H	-1.400799	-3.341103	0.442675
H	-3.338182	0.052672	-1.365519
H	-5.012557	-1.435108	-2.364929
H	1.674390	-0.867789	1.619397
S	2.612504	-0.214045	-0.389205
O	3.752509	0.422986	0.316819
O	2.890573	-1.634558	-0.715243
O	2.125964	0.572023	-1.553646
K	5.425678	-1.774298	0.319734
H	0.609026	1.016230	2.434030
H	1.901082	1.701370	1.466411

50

TS2_conf_10_TS Eopt -1996.216912

C	-1.462754	-0.200741	2.071876
C	-1.820555	-1.055631	0.885297
N	-2.729540	-0.628428	0.041300
C	-3.423556	-1.432224	-0.983096
C	-4.171961	-2.597693	-0.343230
C	-4.363356	-0.500339	-1.737883
H	-1.748470	-2.130959	0.999063
H	-2.672135	-1.811896	-1.682228
H	-4.940597	-2.220325	0.337760
H	-4.654158	-3.192048	-1.123225
H	-3.509824	-3.262200	0.219027
H	-5.124222	-0.094895	-1.062981
H	-3.812834	0.329587	-2.189934
H	-4.868272	-1.051952	-2.533182
N	0.105839	-1.111070	-0.016188
C	0.526482	-2.518589	-0.288610
C	1.145930	-0.309991	0.600131
C	0.836482	0.669880	1.455759
H	1.636396	1.293016	1.843171
C	-0.562820	1.019871	1.828518
C	-0.459769	-3.227108	-1.205928
H	1.485576	-2.491350	-0.814878
C	0.737950	-3.257043	1.029689
H	-0.043008	-4.198083	-1.484073
H	-1.424073	-3.415424	-0.725710
H	-0.622951	-2.659482	-2.127432
H	1.456114	-2.734651	1.668500
H	-0.195316	-3.376512	1.588911
H	1.132609	-4.255279	0.825592
H	-2.949866	0.369064	0.056675
H	-0.204943	-0.631021	-0.866936
C	5.250202	-0.810894	-0.548726
C	4.267288	-0.615282	-1.521754
C	2.933651	-0.476767	-1.148246
C	2.567007	-0.529616	0.203708
C	3.555638	-0.725214	1.172174
C	4.892441	-0.863998	0.797225
H	6.289391	-0.922457	-0.840888
H	4.540733	-0.568244	-2.570971
H	2.171357	-0.317681	-1.908114
H	3.272969	-0.775914	2.220271
H	5.651067	-1.019209	1.557625
H	-0.573167	1.625389	2.738306
S	-1.219455	2.160475	0.555870
O	-0.491817	3.435764	0.755344
O	-0.894389	1.549294	-0.765403
O	-2.680080	2.230188	0.799680
K	1.093645	3.299055	-1.496560
H	-0.950578	-0.829014	2.804007
H	-2.403388	0.134716	2.518172

50

TS2_conf_1_TS Eopt -1996.222183

C	1.044426	1.710103	0.784700
C	-0.181356	2.101845	0.013783
N	-0.309149	1.742644	-1.235182
C	-1.355305	2.228690	-2.145924
C	-0.692856	2.813188	-3.389627
C	-2.319018	1.095497	-2.488373
H	-0.733241	2.984852	0.322521
H	-1.889062	3.017637	-1.605795
H	-0.128416	2.038769	-3.919758
H	-1.456282	3.202446	-4.067308
H	-0.010801	3.624222	-3.124176
H	-1.779036	0.262106	-2.950923
H	-2.831787	0.719055	-1.599068
H	-3.071896	1.453023	-3.195259
N	-1.464641	0.879801	1.191235
C	-1.318207	1.076019	2.665827
C	-1.118956	-0.408072	0.683389
C	0.181621	-0.737319	0.611322
H	0.448573	-1.744182	0.307285
C	1.338788	0.190457	0.841572
C	-2.207608	0.137760	3.478612
H	-0.277266	0.833862	2.900229
C	-1.603249	2.534628	3.003473
H	-2.063085	-0.906220	3.185915
H	-1.953128	0.232429	4.537586
H	-3.264632	0.399455	3.364466
H	-2.613621	2.810906	2.682140
H	-1.545058	2.677627	4.084804
H	-0.891837	3.221230	2.537934
H	0.345914	1.038866	-1.598080
H	-2.405557	1.152947	0.904352
C	-4.216788	-2.921116	-0.917036
C	-2.911097	-3.004619	-1.404662
C	-1.913905	-2.189793	-0.880177
C	-2.195220	-1.287791	0.158575
C	-3.511762	-1.203850	0.630524
C	-4.513498	-2.014542	0.098118
H	-4.997075	-3.549326	-1.334192
H	-2.673220	-3.690938	-2.211007
H	-0.915777	-2.226864	-1.305820

H	-3.770091	-0.511175	1.424021
H	-5.525610	-1.934643	0.481194
H	1.846387	-0.030577	1.788266
S	2.608637	-0.229391	-0.406783
O	3.786656	0.616994	-0.090148
O	2.904715	-1.672066	-0.222232
O	1.988502	0.087354	-1.719401
K	5.552554	-1.425172	0.466799
H	0.960173	2.107137	1.795748
H	1.900185	2.223996	0.331551

50

TS2_conf_2_TS Eopt -1996.221848

C	0.758295	-2.391988	0.050415
C	-0.111235	-1.900065	1.168317
N	0.252717	-0.857228	1.865946
C	-0.400034	-0.407855	3.104226
C	0.658243	-0.263923	4.193252
C	-1.151533	0.898015	2.860495
H	-0.779013	-2.601685	1.659154
H	-1.109311	-1.195646	3.378746
H	1.390680	0.500631	3.913042
H	0.186436	0.044074	5.129233
H	1.182133	-1.208453	4.356747
H	-0.463542	1.674522	2.509083
H	-1.941330	0.776826	2.114211
H	-1.608414	1.238906	3.792936
N	-1.678998	-1.313915	-0.145097
C	-2.094316	-2.372554	-1.115773
C	-1.108858	-0.129700	-0.700249
C	0.141558	-0.191188	-1.187997
H	0.548943	0.681848	-1.687199
C	1.086205	-1.348626	-1.045991
C	-3.176888	-1.895422	-2.081154
H	-1.203314	-2.608065	-1.705026
C	-2.549748	-3.604858	-0.343206
H	-3.339700	-2.662396	-2.842937
H	-4.126128	-1.735413	-1.559577
H	-2.884805	-0.970509	-2.586865
H	-3.385630	-3.354648	0.319367
H	-2.895510	-4.367559	-1.044540
H	-1.750307	-4.043969	0.258624
H	1.026700	-0.286103	1.503654
H	-2.463332	-1.094710	0.470468
C	-3.274142	3.569404	-0.378033
C	-1.878177	3.563132	-0.404197
C	-1.185609	2.362144	-0.511570
C	-1.872487	1.142225	-0.617000
C	-3.272744	1.160487	-0.575498
C	-3.967423	2.363836	-0.458848
H	-3.814591	4.505539	-0.282886
H	-1.327433	4.494312	-0.318314
H	-0.100531	2.365826	-0.476380
H	-3.838233	0.237591	-0.642535
H	-5.052151	2.354183	-0.432468
H	1.218032	-1.876729	-1.998242
S	2.750850	-0.650310	-0.746044
O	3.028922	0.255507	-1.888431
O	2.628662	0.128402	0.528775
O	3.662831	-1.802645	-0.644206
K	3.492102	2.567517	-0.512697
H	0.286221	-3.264157	-0.400157
H	1.694588	-2.752578	0.491613

50

TS2_conf_3_TS Eopt -1996.221848

C	0.758344	-2.391970	0.050191
C	-0.111157	-1.900133	1.168162
N	0.252828	-0.857353	1.865869
C	-0.399889	-0.408052	3.104186
C	0.658380	-0.264494	4.193272
C	-1.151161	0.897980	2.860626
H	-0.778903	-2.601811	1.658960
H	-1.109320	-1.195761	3.378550
H	1.181946	-1.209205	4.356758
H	1.391104	0.499802	3.913100
H	0.186656	0.043655	5.129244
H	-1.940966	0.777029	2.114309
H	-1.608002	1.238812	3.793107
H	-0.463042	1.674431	2.509337
N	-1.678955	-1.313899	-0.145068
C	-2.094349	-2.372503	-1.115746
C	-1.108881	-0.129656	-0.700243
C	0.141498	-0.191127	-1.188090
H	0.548849	0.681891	-1.687353
C	1.086178	-1.348536	-1.046168
C	-3.177012	-1.895354	-2.081017
H	-1.203389	-2.607961	-1.705084
C	-2.549688	-3.604863	-0.343210
H	-4.126226	-1.735430	-1.559367
H	-2.885015	-0.970393	-2.586688
H	-3.339835	-2.662288	-2.842837
H	-2.895539	-4.367505	-1.044564
H	-1.750173	-4.044032	0.258480

H	-3.385485	-3.354711	0.319490
H	1.026811	-0.286227	1.503579
H	-2.463220	-1.094720	0.470593
C	-3.274235	3.569393	-0.377891
C	-3.967492	2.363806	-0.458591
C	-3.272793	1.160469	-0.575280
C	-1.872542	1.142246	-0.616944
C	-1.185683	2.362188	-0.511613
C	-1.878271	3.563159	-0.404201
H	-3.814695	4.505520	-0.282721
H	-5.052217	2.354123	-0.432088
H	-3.838266	0.237556	-0.642213
H	-0.100604	2.365887	-0.476542
H	-1.327546	4.494358	-0.318394
H	1.218003	-1.876593	-1.998445
S	2.750828	-0.650222	-0.746236
O	3.028734	0.255883	-1.888442
O	2.628780	0.128171	0.528789
O	3.662860	-1.802540	-0.644783
K	3.491965	2.567639	-0.512374
H	0.286262	-3.264113	-0.400425
H	1.694659	-2.752572	0.491330

50

TS2_conf_4_TS Eopt -1996.205985

C	-0.802420	0.089382	-1.941089
C	-0.573748	1.403660	-1.238042
N	-1.554596	1.888730	-0.510412
C	-1.785444	3.293476	-0.147246
C	-2.978965	3.818049	-0.944165
C	-2.017157	3.401107	1.357113
H	0.083904	2.126928	-1.706710
H	-0.892096	3.848738	-0.440453
H	-3.880635	3.244149	-0.706866
H	-3.162400	4.864627	-0.688005
H	-2.790089	3.747131	-2.018093
H	-1.182296	2.985701	1.927757
H	-2.148447	4.448072	1.640574
H	-2.924259	2.855353	1.637810
N	1.023272	0.768454	0.015930
C	2.012888	1.756398	0.548086
C	1.559130	-0.524983	-0.322195
C	0.738378	-1.488710	-0.764993
H	1.151232	-2.466660	-0.985246
C	-0.682936	-1.218417	-1.128995
C	2.254061	1.494834	2.035229
H	2.940271	1.601144	-0.003116
C	1.589105	3.202309	0.328954
H	3.019940	2.174485	2.418299
H	1.331130	1.667382	2.601103
H	2.582921	0.468880	2.216166
H	2.385180	3.852629	0.700684
H	1.441581	3.439714	-0.727177
H	0.684459	3.444235	0.891383
H	-2.291461	1.235340	-0.229734
H	0.307149	0.600767	0.734100
C	5.743342	-1.472641	-0.112751
C	5.261555	-0.666278	-1.145408
C	3.908057	-0.342140	-1.204885
C	3.023836	-0.813727	-0.226998
C	3.510707	-1.629435	0.798608
C	4.865554	-1.957364	0.855456
H	6.797549	-1.726556	-0.067994
H	5.937741	-0.295568	-1.909060
H	3.531259	0.273596	-2.017791
H	2.824158	-2.008027	1.550959
H	5.232072	-2.591907	1.656023
H	-1.063306	-2.024738	-1.761195
S	-1.800778	-1.269435	0.312604
O	-3.072885	-0.639511	-0.156258
O	-1.989004	-2.700208	0.643179
O	-1.152571	-0.479719	1.387601
K	-4.712324	-2.827478	0.179238
H	-0.085835	0.006532	-2.759159
H	-1.803806	0.143187	-2.376301

50

TS2_conf_5_TS Eopt -1996.206309

C	1.200979	-0.292641	-2.189459
C	1.482915	-1.305243	-1.108181
N	2.554114	-1.132653	-0.367210
C	3.305450	-2.166530	0.358245
C	4.609396	-2.444824	-0.387766
C	3.557323	-1.703233	1.789620
H	1.171151	-2.330412	-1.273826
H	2.692072	-3.069785	0.348126
H	5.225897	-1.541487	-0.434050
H	5.175460	-3.219309	0.136061
H	4.408080	-2.785130	-1.406419
H	4.186817	-0.807116	1.788800
H	2.625052	-1.462574	2.307722
H	4.077759	-2.484317	2.348822
N	-0.263344	-0.957664	0.056487
C	-0.800118	-2.013478	0.970221

C -1.253422 -0.143141 -0.597646
C -0.864435 0.867387 -1.389974
H -1.621730 1.487114 -1.856892
C 0.558928 1.054725 -1.797086
C -1.157182 -1.391243 2.320667
H -1.701328 -2.408357 0.501219
C 0.162021 -3.176914 1.164747
H -1.861809 -0.563493 2.209855
H -1.608664 -2.141574 2.975176
H -0.251218 -1.013373 2.808957
H -0.322814 -3.920603 1.802396
H 0.416183 -3.670498 0.223926
H 1.074895 -2.859471 1.673876
H 2.972692 -0.197943 -0.365655
H 0.318514 -0.314040 0.607792
C -5.474002 -0.880605 -0.291465
C -4.895670 0.175055 0.411284
C -3.521226 0.399743 0.329575
C -2.714947 -0.428907 -0.455894
C -3.304091 -1.479239 -1.170233
C -4.675745 -1.706489 -1.084751
H -6.542941 -1.056578 -0.227079
H -5.511743 0.825724 1.023630
H -3.069156 1.222598 0.876792
H -2.685248 -2.114288 -1.799275
H -5.121635 -2.523306 -1.643072
H 0.609847 1.702373 -2.676053
S 1.528523 1.981430 -0.559651
O 1.072814 3.386673 -0.659562
O 1.185481 1.397405 0.774475
O 2.948739 1.760752 -0.918360
K -0.357418 3.498638 1.683170
H 0.533242 -0.747685 -2.922152
H 2.156493 -0.096446 -2.683270
50
TS2_conf_6_TS Eopt -1996.213805
C 1.049970 0.264192 1.975722
C 0.721588 1.523977 1.223869
N 1.593329 2.031081 0.385077
C 1.512577 3.367035 -0.221072
C 2.862296 4.060714 -0.064760
C 1.092747 3.261614 -1.685711
H 0.060573 2.244229 1.696763
H 0.753961 3.916688 0.346622
H 2.812609 5.065725 -0.489994
H 3.141221 4.137747 0.988523
H 3.641820 3.504718 -0.596315
H 1.821171 2.670283 -2.248892
H 0.110963 2.791264 -1.793734
H 1.041006 4.259768 -2.127699
N -0.966382 0.838303 0.125290
C -1.905206 1.990812 0.174248
C -1.423048 -0.494146 0.423839
C -0.551933 -1.387879 0.911551
H -0.883713 -2.412548 1.044771
C 0.870848 -1.069310 1.231037
C -2.658945 2.016332 1.500749
H -1.242326 2.864489 0.148246
C -2.846700 2.137197 -1.022899
H -3.151923 2.984436 1.616237
H -3.428325 1.241344 1.531422
H -1.991097 1.869185 2.355486
H -3.694448 1.452751 -0.964123
H -3.234329 3.160078 -1.034754
H -2.321283 1.964036 -1.967528
H 2.342386 1.417984 0.052171
H -0.431202 0.805672 -0.748334
C -5.327740 -1.928120 -0.631480
C -4.956196 -1.853105 0.710180
C -3.695846 -1.367740 1.059892
C -2.803931 -0.942434 0.071650
C -3.177555 -1.034262 -1.274505
C -4.432996 -1.523783 -1.624464
H -6.307799 -2.306179 -0.904400
H -5.644902 -2.172312 1.485850
H -3.401621 -1.307976 2.104361
H -2.478894 -0.723883 -2.047496
H -4.711668 -1.594051 -2.671066
H 1.298892 -1.854670 1.858864
S 1.887433 -1.147195 -0.290885
O 3.172435 -0.470458 0.065288
O 2.089709 -2.586762 -0.577462
O 1.145294 -0.415793 -1.342314
K 4.819668 -2.663909 -0.180884
H 0.411428 0.218285 2.860125
H 2.087174 0.344187 2.312107
50
TS2_conf_7_TS Eopt -1996.214257
C 1.500410 -0.280058 -2.202602
C 1.711414 -1.280230 -1.100142
N 2.693858 -1.112378 -0.247688
C 3.173230 -2.116115 0.712196

C	4.693640	-2.197862	0.618314
C	2.709139	-1.764172	2.124115
H	1.424516	-2.311802	-1.281007
H	2.738179	-3.072167	0.401400
H	5.008392	-2.460269	-0.394152
H	5.145154	-1.237939	0.890224
H	5.065919	-2.956464	1.310532
H	3.094134	-0.782518	2.416845
H	1.617987	-1.742480	2.199229
H	3.082821	-2.508248	2.831971
N	-0.134678	-1.052610	-0.067350
C	-0.493175	-2.446686	0.307827
C	-1.102121	-0.179337	-0.678055
C	-0.674346	0.805778	-1.480749
H	-1.403238	1.512657	-1.864185
C	0.758211	1.016978	-1.842714
C	-1.149552	-3.170298	-0.864188
H	0.478483	-2.921378	0.491650
C	-1.292997	-2.607870	1.602085
H	-0.598584	-3.021967	-1.798343
H	-1.180240	-4.241844	-0.653347
H	-2.175813	-2.827329	-1.014831
H	-1.206442	-3.646458	1.934448
H	-0.900253	-1.965958	2.397169
H	-2.351386	-2.383461	1.461397
H	3.108086	-0.178813	-0.181157
H	0.319003	-0.543329	0.697682
C	-5.285806	-0.236404	0.276449
C	-4.866072	-0.506807	-1.025331
C	-3.505731	-0.512693	-1.333741
C	-2.554913	-0.260162	-0.340603
C	-2.983800	0.022909	0.961596
C	-4.341833	0.034366	1.269045
H	-6.344045	-0.231347	0.517168
H	-5.595352	-0.713347	-1.802052
H	-3.173754	-0.724018	-2.346688
H	-2.249829	0.235201	1.735116
H	-4.663739	0.257568	2.281218
H	0.831789	1.686018	-2.703847
S	1.609032	1.952713	-0.518436
O	1.104884	3.342390	-0.620600
O	1.207148	1.325427	0.776215
O	3.056864	1.794174	-0.791024
K	-0.414077	3.360642	1.665160
H	0.927366	-0.761792	-2.997244
H	2.484177	-0.023152	-2.604748

50

TS2_conf_8_TS Eopt -1996.220792

C	0.767596	-2.367109	-0.102190
C	-0.173340	-1.960941	0.993356
N	0.175812	-1.015873	1.825204
C	-0.543099	-0.604036	3.046341
C	-1.195050	0.759389	2.823121
C	-1.546015	-1.651259	3.506255
H	-0.896949	-2.688247	1.342939
H	0.234403	-0.502679	3.810306
H	-1.605987	1.128048	3.766329
H	-0.469985	1.489627	2.452258
H	-2.011775	0.688665	2.096903
H	-1.970701	-1.325565	4.457969
H	-2.375633	-1.761420	2.799713
H	-1.075315	-2.625729	3.660709
N	-1.611927	-1.176718	-0.363766
C	-1.982584	-2.120014	-1.463595
C	-0.964460	0.034006	-0.754158
C	0.319163	-0.027245	-1.146691
H	0.791652	0.873076	-1.526280
C	1.209927	-1.232110	-1.058902
C	-2.969434	-1.512822	-2.457908
H	-1.055068	-2.332351	-2.003017
C	-2.537160	-3.401525	-0.852688
H	-3.954133	-1.373933	-2.000002
H	-2.613508	-0.552884	-2.842888
H	-3.088203	-2.195134	-3.303721
H	-1.802736	-3.929326	-0.239501
H	-3.413507	-3.181695	-0.232686
H	-2.853002	-4.078664	-1.649437
H	0.995875	-0.452632	1.572063
H	-2.436516	-0.985541	0.207009
C	-3.060143	3.760841	-0.295973
C	-1.666224	3.716680	-0.227275
C	-0.995698	2.507751	-0.376266
C	-1.700711	1.318160	-0.619373
C	-3.099617	1.373799	-0.671949
C	-3.773201	2.584336	-0.513001
H	-3.584051	4.702620	-0.169404
H	-1.101076	4.623196	-0.035974
H	0.083967	2.480445	-0.266747
H	-3.681689	0.475995	-0.847294
H	-4.857053	2.602551	-0.561398
H	1.389197	-1.665564	-2.050484
S	2.873761	-0.646547	-0.574551

O 3.259697 0.380094 -1.574500
O 2.692491 -0.026371 0.778206
O 3.735620 -1.840780 -0.559867
K 3.653644 2.478223 0.142398
H 0.307708 -3.172133 -0.673768
H 1.655336 -2.805040 0.368249
50
TS3_conf_10_ts Eopt -1996.208286
C -3.190297 2.282374 0.124530
C -1.820161 1.852638 -0.386725
C -1.831219 1.359243 -1.831952
N -1.102260 0.855087 0.476760
C -1.442334 -0.548879 0.480449
C -2.855806 -0.945205 0.231895
C -3.839847 -0.660446 1.184963
C -5.145708 -1.105129 0.996304
C -5.475008 -1.842793 -0.142410
C -4.493145 -2.144455 -1.085287
C -3.184499 -1.701632 -0.896160
C -0.528182 -1.462882 0.821738
C 0.869754 -1.070589 1.164727
C 0.875660 0.321159 1.794467
C -0.024798 1.272143 1.090220
N 1.530305 2.080849 -0.561757
C 2.064313 3.448728 -0.436394
C 2.781356 3.578460 0.903963
C 0.940471 4.469283 -0.578593
H -3.167515 2.483690 1.198821
H -3.456588 3.209881 -0.389802
H -3.963050 1.543241 -0.088200
H -1.162266 2.722192 -0.338408
H -2.017410 2.213802 -2.487536
H -0.869839 0.909128 -2.098053
H -2.622035 0.625319 -1.999432
H -3.579371 -0.090978 2.073103
H -5.903934 -0.880873 1.739459
H -6.493287 -2.188193 -0.289399
H -4.742794 -2.725433 -1.967073
H -2.413574 -1.933596 -1.625979
H -0.820401 -2.506469 0.851922
H 1.880875 0.743033 1.847364
H 0.501774 0.239839 2.825790
H 0.058869 2.332868 1.275001
H 2.292941 1.404739 -0.510994
H 1.102134 1.934337 -1.474236
H 2.795916 3.637444 -1.235978
H 2.081424 3.448392 1.737419
H 3.572720 2.827749 0.999447
H 3.237150 4.567437 1.000285
H 1.348483 5.483496 -0.588676
H 0.385515 4.318577 -1.510980
H 0.236899 4.404998 0.259676
H 1.322302 -1.784962 1.855497
S 1.919246 -1.213693 -0.331087
O 3.178750 -0.486921 0.006095
O 2.158968 -2.671556 -0.485394
O 1.174639 -0.604973 -1.450364
K 4.862561 -2.661928 -0.018094
50
TS3_conf_1_ts Eopt -1996.213359
C 1.930215 1.899303 1.364163
C 2.177467 1.825570 -0.141040
C 2.046695 3.179703 -0.820769
N 1.262707 0.796680 -0.732206
C 1.568711 -0.601380 -0.518715
C 2.953938 -0.966069 -0.115265
C 4.032922 -0.751223 -0.982431
C 5.313317 -1.158091 -0.618791
C 5.525585 -1.785668 0.610717
C 4.453490 -2.009031 1.472501
C 3.169525 -1.600633 1.110889
C 0.643535 -1.542796 -0.733364
C -0.743896 -1.210625 -1.168040
C -0.768400 0.129479 -1.896421
C 0.145660 1.146877 -1.309565
N -1.397681 1.958831 0.351734
C -1.594737 3.402451 0.551057
C -2.537965 3.700763 1.717527
C -2.106053 4.020972 -0.746095
H 2.690543 2.539507 1.817687
H 0.947999 2.330617 1.573465
H 1.988898 0.912544 1.830771
H 3.185648 1.454895 -0.319889
H 2.170577 3.103589 -1.904224
H 1.095157 3.670835 -0.597435
H 2.840826 3.820890 -0.432809
H 3.864963 -0.273723 -1.944653
H 6.143971 -0.991435 -1.296732
H 6.524579 -2.102219 0.892665
H 4.612959 -2.499089 2.427389
H 2.329375 -1.769936 1.778922
H 0.917007 -2.580223 -0.574969

H -1.776757 0.537563 -1.972123
H -0.406871 -0.030929 -2.923072
H 0.036462 2.179809 -1.600560
H -2.287903 1.513932 0.115743
H -1.096213 1.491144 1.205690
H -0.613562 3.834909 0.787340
H -3.532583 3.288582 1.512712
H -2.164099 3.253756 2.643632
H -2.637385 4.779685 1.871389
H -3.070304 3.577213 -1.020354
H -2.249134 5.098208 -0.629341
H -1.410841 3.860424 -1.575950
H -1.138872 -1.985139 -1.829310
S -1.874907 -1.305611 0.275625
O -3.100566 -0.564547 -0.139555
O -2.148571 -2.756995 0.441610
O -1.177669 -0.699741 1.427489
K -4.835089 -2.701134 -0.109325
50
TS3_conf_2_ts Eopt -1996.213860
C -0.552826 -1.919467 1.786785
C -0.802375 -2.450020 0.376758
C -0.078686 -3.760735 0.110818
N -0.438315 -1.383059 -0.610072
C -1.325956 -0.249744 -0.754374
C -2.733168 -0.396252 -0.292988
C -3.598268 -1.304781 -0.916380
C -4.930565 -1.384559 -0.521677
C -5.410395 -0.556139 0.495209
C -4.555124 0.354310 1.112867
C -3.219053 0.434533 0.719958
C -0.907062 0.873889 -1.346827
C 0.483266 1.027769 -1.864502
C 1.082522 -0.335336 -2.194606
C 0.714466 -1.400799 -1.222799
N 2.474252 -0.941418 0.356314
C 3.269795 -2.023939 0.955758
C 4.290786 -1.496783 1.965469
C 3.950147 -2.820787 -0.152817
H 0.517297 -1.786912 1.964398
H -1.058699 -0.964866 1.953117
H -0.936046 -2.641714 2.511333
H -1.869629 -2.620421 0.241890
H -0.219219 -4.100617 -0.918663
H 0.991030 -3.700276 0.331170
H -0.505018 -4.513238 0.777402
H -3.227392 -1.940917 -1.716258
H -5.595847 -2.087995 -1.011485
H -6.449502 -0.619562 0.801477
H -4.923928 1.001516 1.901844
H -2.545639 1.139287 1.200297
H -1.608511 1.694306 -1.452933
H 2.166421 -0.292009 -2.305120
H 0.672714 -0.666440 -3.160688
H 1.263976 -2.328629 -1.228287
H 3.089220 -0.293694 -0.141976
H 2.018846 -0.365049 1.063320
H 2.571709 -2.682726 1.488608
H 5.012438 -0.841610 1.464385
H 3.796338 -0.923284 2.755309
H 4.839715 -2.321326 2.430613
H 3.224421 -3.260765 -0.844000
H 4.617600 -2.170087 -0.729683
H 4.548417 -3.632865 0.268204
H 0.494482 1.650624 -2.761968
S 1.476576 2.025486 -0.687207
O 1.027796 3.423269 -0.919582
O 1.118875 1.579966 0.688468
O 2.886859 1.766959 -1.046382
K -0.162907 3.864438 1.503651
50
TS3_conf_5_ts Eopt -1996.208930
C -3.101472 2.474348 0.199591
C -1.748813 1.974019 -0.293587
C -1.754362 1.539268 -1.757532
N -1.128077 0.892939 0.545885
C -1.547781 -0.486268 0.460496
C -2.978320 -0.781475 0.175517
C -3.958654 -0.461472 1.121356
C -5.287544 -0.810680 0.896802
C -5.644264 -1.487576 -0.271095
C -4.667498 -1.824049 -1.207526
C -3.335892 -1.476643 -0.982782
C -0.689069 -1.468916 0.752592
C 0.719448 -1.175782 1.149517
C 0.780243 0.167608 1.874162
C -0.042875 1.208461 1.203629
N 1.588591 2.005770 -0.378771
C 1.974259 3.399893 -0.126776
C 2.755202 4.011982 -1.289856
C 2.774776 3.459005 1.171026
H -3.095021 2.632908 1.281192

H	-3.293609	3.435637	-0.284600
H	-3.913744	1.795506	-0.061370
H	-1.034404	2.794260	-0.190415
H	-0.817869	1.038258	-2.020939
H	-2.585863	0.864082	-1.969868
H	-1.871652	2.427983	-2.382594
H	-3.676640	0.060611	2.031873
H	-6.042796	-0.559522	1.634367
H	-6.680576	-1.757983	-0.446375
H	-4.939385	-2.357250	-2.112636
H	-2.569116	-1.734690	-1.708208
H	-1.038014	-2.494653	0.712564
H	1.804634	0.527914	1.981760
H	0.368631	0.037134	2.885725
H	0.103398	2.254746	1.437029
H	2.399055	1.385271	-0.338332
H	1.183760	1.876750	-1.303707
H	1.046610	3.976063	0.006135
H	2.176680	3.961032	-2.217231
H	2.991267	5.061697	-1.090005
H	3.694790	3.468332	-1.438753
H	3.055149	4.489027	1.404656
H	2.206099	3.063055	2.018177
H	3.693817	2.869112	1.072361
H	1.113983	-1.957805	1.801488
S	1.804508	-1.281905	-0.325156
O	3.087533	-0.639479	0.085003
O	1.978885	-2.740313	-0.551944
O	1.122341	-0.583048	-1.431274
K	4.664773	-2.884504	0.003312

50

TS3_conf_6_ts Eopt -1996.208264

C	-3.166854	2.343485	0.143065
C	-1.766770	1.922980	-0.288696
C	-1.675247	1.517668	-1.758045
N	-1.128755	0.855481	0.553503
C	-1.520093	-0.531679	0.461312
C	-2.943718	-0.850654	0.164750
C	-3.289274	-1.503586	-1.020974
C	-4.614900	-1.865516	-1.259672
C	-5.596332	-1.586906	-0.309567
C	-5.250464	-0.954704	0.886532
C	-3.928267	-0.590390	1.124564
C	-0.649204	-1.499864	0.763652
C	0.752096	-1.183643	1.166990
C	0.790367	0.164514	1.884965
C	-0.055737	1.191353	1.221354
N	1.596346	2.059285	-0.301262
C	2.215913	3.300004	0.188016
C	1.236587	4.460526	0.042299
C	3.537575	3.602258	-0.520256
H	-3.931198	1.636637	-0.181714
H	-3.227927	2.468804	1.227222
H	-3.377174	3.309572	-0.323927
H	-1.101356	2.775037	-0.136544
H	-0.693761	1.089694	-1.982159
H	-2.445757	0.788963	-2.019109
H	-1.828909	2.407417	-2.374023
H	-2.518559	-1.720169	-1.755671
H	-4.877893	-2.365957	-2.185868
H	-6.627698	-1.868754	-0.495660
H	-6.009254	-0.749703	1.634659
H	-3.654619	-0.101031	2.055479
H	-0.981359	-2.531114	0.722622
H	1.808179	0.545472	1.989701
H	0.388921	0.031656	2.900238
H	0.065640	2.235072	1.474466
H	2.285813	1.309003	-0.339785
H	1.251471	2.174111	-1.253807
H	2.424568	3.145661	1.256732
H	0.942832	4.578745	-1.007740
H	0.330206	4.313456	0.637465
H	1.698834	5.394564	0.371341
H	3.360913	3.779927	-1.587106
H	4.012159	4.492450	-0.096323
H	4.232001	2.762342	-0.421555
H	1.156391	-1.956800	1.823585
S	1.841825	-1.276550	-0.303666
O	3.116267	-0.620816	0.114550
O	2.031673	-2.731034	-0.538244
O	1.159330	-0.572178	-1.405747
K	4.719105	-2.849814	0.023907

50

TS3_conf_7_ts Eopt -1996.210586

C	-0.810523	-1.651832	2.034801
C	-0.787285	-2.353757	0.678600
C	0.061069	-3.613753	0.696869
N	-0.341953	-1.371080	-0.362767
C	-1.264357	-0.314827	-0.728750
C	-2.712018	-0.511605	-0.441933
C	-3.430670	-1.537382	-1.069337
C	-4.799733	-1.665421	-0.853379

C	-5.463143	-0.768336	-0.013559
C	-4.754030	0.259231	0.605376
C	-3.381730	0.387934	0.391599
C	-0.836663	0.766145	-1.388445
C	0.599109	0.947948	-1.744232
C	1.283032	-0.407315	-1.891496
C	0.860652	-1.408615	-0.872404
N	2.563895	-0.900550	0.752983
C	3.899606	-1.534029	0.721546
C	4.535807	-1.309658	-0.646244
C	3.792007	-3.018155	1.049383
H	0.199101	-1.376407	2.347860
H	-1.425332	-0.749210	2.013146
H	-1.228754	-2.333634	2.778868
H	-1.804179	-2.631364	0.403861
H	0.110848	-4.090143	-0.286020
H	1.072105	-3.421724	1.064084
H	-0.411121	-4.316759	1.386260
H	-2.917532	-2.227511	-1.734569
H	-5.349906	-2.460400	-1.345912
H	-6.530557	-0.869598	0.153572
H	-5.265126	0.960657	1.256657
H	-2.823134	1.184727	0.875001
H	-1.560902	1.521378	-1.672887
H	2.368356	-0.311448	-1.890209
H	0.995482	-0.837025	-2.862707
H	1.425756	-2.319315	-0.757224
H	2.670506	0.105592	0.632628
H	2.156244	-1.016048	1.679154
H	4.544196	-1.064387	1.479222
H	3.991010	-1.859768	-1.422195
H	4.539014	-0.246151	-0.906702
H	5.568485	-1.668805	-0.646136
H	3.224562	-3.550685	0.277541
H	4.788238	-3.466167	1.097715
H	3.302583	-3.178838	2.015955
H	0.702924	1.508058	-2.676502
S	1.398518	2.048575	-0.519158
O	0.869402	3.402031	-0.826920
O	0.960632	1.593897	0.829441
O	2.851912	1.887702	-0.739510
K	-0.446058	3.851090	1.528427

50

TS3_conf_8_ts Eopt -1996.208753

C	1.737088	-3.155900	-0.632446
C	0.584858	-2.164449	-0.745402
C	0.640503	-1.301698	-2.004147
N	0.383466	-1.273188	0.446453
C	1.259003	-0.160267	0.729031
C	2.702224	-0.291010	0.387459
C	3.277195	0.526259	-0.588546
C	4.644016	0.444491	-0.854238
C	5.441837	-0.445820	-0.137191
C	4.874363	-1.248253	0.854791
C	3.509920	-1.169618	1.118482
C	0.804351	0.900953	1.403621
C	-0.616800	0.983351	1.851590
C	-1.152096	-0.419259	2.134059
C	-0.740618	-1.406717	1.101640
N	-2.592182	-1.109893	-0.409930
C	-3.626787	-2.129291	-0.182359
C	-3.189246	-3.455914	-0.795116
C	-4.991505	-1.696005	-0.719682
H	1.754407	-3.634911	0.349894
H	1.576906	-3.931090	-1.386826
H	2.705063	-2.691678	-0.824286
H	-0.344722	-2.736201	-0.774252
H	0.457144	-1.940326	-2.872070
H	-0.119537	-0.515488	-1.967861
H	1.622894	-0.840140	-2.126387
H	2.649800	1.219165	-1.142804
H	5.082923	1.076836	-1.619027
H	6.505242	-0.509553	-0.344142
H	5.494919	-1.931807	1.424947
H	3.065612	-1.791437	1.891118
H	1.494450	1.703046	1.640597
H	-2.239456	-0.430924	2.234201
H	-0.735936	-0.768915	3.090262
H	-1.238579	-2.364594	1.044172
H	-2.942427	-0.184803	-0.163196
H	-2.335673	-1.063447	-1.395399
H	-3.710191	-2.253925	0.907523
H	-2.265121	-3.833660	-0.346942
H	-3.960796	-4.216342	-0.651169
H	-3.024616	-3.338935	-1.873077
H	-4.947869	-1.579958	-1.808462
H	-5.757694	-2.440212	-0.482126
H	-5.294246	-0.739421	-0.282936
H	-0.710103	1.596367	2.750621
S	-1.594978	1.917817	0.616629
O	-1.175290	3.330736	0.801932
O	-1.206892	1.413694	-0.728566

O -3.010739 1.648354 0.953344
K 0.020621 3.691129 -1.638793
36
TS4_conf_0 Eopt -1821.322583
C -3.647196 -2.582780 0.148283
C -2.608661 -1.653017 0.767703
C -2.020066 -2.196534 2.067106
N -1.514049 -1.359289 -0.205048
C -0.725856 -2.396825 -0.604448
C 0.383472 -2.220895 -1.379143
C 0.792039 -0.906928 -1.716154
C -0.125138 0.131551 -1.434044
C -1.234774 -0.093770 -0.651494
C -2.143485 1.035787 -0.319965
C -1.645204 2.112147 0.419076
C -2.466524 3.203123 0.703153
C -3.782064 3.225769 0.242990
C -4.278749 2.156133 -0.504910
C -3.463483 1.063340 -0.785929
H -3.236262 -3.578018 -0.044351
H -4.031916 -2.177212 -0.790878
H -4.481199 -2.696078 0.844981
H -3.076516 -0.693976 0.981691
H -1.555578 -3.176422 1.923920
H -2.823928 -2.309014 2.798700
H -1.273235 -1.508576 2.470745
H -1.036217 -3.379931 -0.272965
H 0.965698 -3.085810 -1.671294
H 0.052671 1.138863 -1.790069
H -0.619091 2.086119 0.775187
H -2.076779 4.033069 1.283416
H -4.419875 4.075854 0.462749
H -5.299434 2.174845 -0.872616
H -3.845973 0.234589 -1.375674
H 1.566782 -0.752084 -2.455443
S 2.497818 -0.378716 0.096064
O 3.925956 -0.957381 0.133988
O 1.762734 -0.817335 1.356000
O 2.653183 1.153547 0.129198
K 5.309645 1.310264 0.155968
36
TS4_conf_1 Eopt -1821.322617
C -2.912245 -2.666001 1.233469
C -1.812665 -1.611185 1.278442
C -0.802288 -1.842372 2.401140
N -1.095666 -1.534505 -0.031208
C -0.311744 -2.589619 -0.373017
C 0.567333 -2.538037 -1.419179
C 0.713383 -1.326884 -2.125073
C -0.252034 -0.332520 -1.898356
C -1.099024 -0.412760 -0.811874
C -1.961275 0.751290 -0.487216
C -3.355833 0.659104 -0.412741
C -4.112655 1.798386 -0.152796
C -3.481321 3.029875 0.035628
C -2.092407 3.122978 -0.046206
C -1.327840 1.987393 -0.314112
H -2.498850 -3.669959 1.099249
H -3.620925 -2.471551 0.424710
H -3.454781 -2.652215 2.181929
H -2.261923 -0.630408 1.421608
H -1.296125 -1.667809 3.360252
H 0.045919 -1.161509 2.291272
H -0.426654 -2.870087 2.400677
H -0.408051 -3.471795 0.247175
H 1.185099 -3.400501 -1.635342
H -0.277243 0.565443 -2.501632
H -3.847327 -0.296606 -0.572715
H -5.194169 1.726491 -0.102419
H -4.074224 3.915669 0.240223
H -1.602284 4.080929 0.095833
H -0.241153 2.032381 -0.389946
H 1.346701 -1.278193 -3.001062
S 2.435197 -0.110078 -0.688017
O 1.912184 -0.582288 0.683289
O 1.995509 1.364970 -0.817041
O 3.976647 -0.090812 -0.582567
K 3.457972 1.581034 1.576254
36
TS4_conf_10 Eopt -1821.317322
C -1.954160 -0.343190 2.313535
C -1.906832 -1.645754 1.517930
C -3.287712 -2.235161 1.249265
N -1.081291 -1.571729 0.263902
C -0.224997 -2.615726 0.060640
C 0.666679 -2.650128 -0.972944
C 0.758576 -1.532805 -1.834348
C -0.266049 -0.574502 -1.735136
C -1.135141 -0.568572 -0.666393
C -2.071060 0.580635 -0.530379
C -3.427806 0.476703 -0.842564
C -4.252170 1.595662 -0.726052

C -3.719483 2.816041 -0.310282
C -2.354675 2.926586 -0.037125
C -1.525229 1.813891 -0.156965
H -2.225491 -0.599828 3.340991
H -2.699786 0.355683 1.931872
H -0.977715 0.148232 2.328876
H -1.350589 -2.361009 2.126191
H -3.216800 -3.151518 0.657434
H -3.933643 -1.523357 0.733159
H -3.753002 -2.479292 2.208020
H -0.290566 -3.421594 0.781269
H 1.331045 -3.499316 -1.068967
H -0.330770 0.243451 -2.442526
H -3.835026 -0.467440 -1.191173
H -5.307056 1.514466 -0.967307
H -4.364203 3.684031 -0.215516
H -1.936579 3.881240 0.266149
H -0.456346 1.865472 0.043552
H 1.380932 -1.574917 -2.718520
S 2.544244 -0.163442 -0.631506
O 1.659795 0.858886 0.105882
O 3.658987 0.645173 -1.324182
O 3.229646 -1.009859 0.456740
K 4.138929 1.515115 1.292667
36
TS4_conf_7 Eopt -1821.321336
C 2.800985 2.800643 1.191538
C 1.806691 1.647285 1.262240
C 0.779652 1.802184 2.382207
N 1.095194 1.473604 -0.040480
C 0.256362 2.467820 -0.434788
C -0.598894 2.328969 -1.491230
C -0.688312 1.077185 -2.137796
C 0.309083 0.130280 -1.836111
C 1.156357 0.316617 -0.764421
C 2.147828 -0.734688 -0.417620
C 1.690674 -2.008644 -0.069601
C 2.604368 -3.023584 0.213616
C 3.973460 -2.771509 0.141281
C 4.432041 -1.501767 -0.215620
C 3.523306 -0.484597 -0.493587
H 3.356477 2.845542 2.131456
H 2.296530 3.761882 1.057111
H 3.512781 2.662831 0.373799
H 2.351286 0.718700 1.422991
H 1.291860 1.732180 3.345003
H 0.021573 1.018008 2.310390
H 0.279557 2.774432 2.336404
H 0.301239 3.381418 0.144717
H -1.246536 3.153872 -1.760302
H 0.390213 -0.791064 -2.400156
H 0.621865 -2.195283 -0.013389
H 2.244874 -4.009519 0.489462
H 4.683642 -3.562780 0.358504
H 5.497010 -1.305078 -0.282457
H 3.878858 0.500644 -0.782751
H -1.284379 0.966870 -3.033960
S -2.522414 -0.008788 -0.735158
O -1.826457 0.229403 0.617776
O -2.554091 -1.537269 -0.939221
O -3.988466 0.435952 -0.551769
K -3.879688 -1.427849 1.544954
44
TS5_conf_10_TS Eopt -771.868094
C -1.813711 -0.142142 -1.099999
C -1.830000 -1.493316 -1.693135
C -0.668745 -2.107025 -1.967848
C 0.616799 -1.512290 -1.602287
C 0.769727 -0.685593 -0.544081
H -0.667861 -3.065938 -2.477017
H 1.482005 -1.777425 -2.198779
N -0.365583 -0.502508 0.329649
H -2.792197 -1.932954 -1.933681
C 2.028243 0.031129 -0.237421
C 2.982942 0.239449 -1.247870
C 2.290478 0.543223 1.041614
C 4.172960 0.903610 -0.976160
H 2.784533 -0.096787 -2.259980
C 3.483047 1.211689 1.311996
H 1.576295 0.416618 1.848109
C 4.431060 1.389738 0.307305
H 4.894682 1.055396 -1.772079
H 3.666731 1.592515 2.311208
H 5.358088 1.913163 0.517090
H -1.103177 0.568028 -1.517183
N -2.928258 0.361777 -0.617669
H -3.680122 -0.285202 -0.398813
C -3.177936 1.772727 -0.256620
H -4.153544 2.005595 -0.695185
C -2.136216 2.702815 -0.859864
H -2.076414 2.598816 -1.946242
H -2.424387 3.731270 -0.633774

H	-1.142875	2.539677	-0.427990
C	-3.271977	1.924009	1.260169
H	-2.304034	1.729590	1.732347
H	-3.571672	2.946344	1.502903
H	-4.013084	1.242042	1.685878
C	-0.674422	-1.648274	1.241002
H	-1.081040	-2.433448	0.595311
C	0.549908	-2.206754	1.964496
H	0.912112	-1.512097	2.728065
H	0.265861	-3.135769	2.466082
H	1.366653	-2.434358	1.274252
C	-1.741663	-1.209679	2.234808
H	-1.997328	-2.049169	2.885583
H	-1.368285	-0.396475	2.868511
H	-2.654685	-0.875307	1.739853
H	-0.282339	0.361102	0.865020

44

TS5_conf_1_TS Eopt -771.870048

C	1.975712	0.930999	-0.595559
C	1.717897	2.318079	-0.170507
C	0.495545	2.865023	-0.237878
C	-0.729734	2.129916	-0.539293
C	-0.890927	0.807318	-0.342637
H	0.381395	3.917996	0.003306
H	-1.597490	2.708836	-0.839682
N	0.247066	0.014954	0.070355
H	2.576743	2.899657	0.150706
C	-2.208659	0.136350	-0.452670
C	-3.363323	0.768826	0.029148
C	-2.324483	-1.134099	-1.035127
C	-4.606192	0.149274	-0.078066
H	-3.280795	1.737605	0.513166
C	-3.567459	-1.750544	-1.144948
H	-1.448335	-1.645521	-1.424654
C	-4.711539	-1.111145	-0.665686
H	-5.489726	0.646773	0.308394
H	-3.642279	-2.729686	-1.606445
H	-5.679165	-1.595734	-0.745762
H	1.667795	0.648206	-1.601358
N	3.025091	0.290976	-0.116901
H	3.462393	0.647956	0.728768
C	3.590986	-0.941797	-0.687030
H	2.980320	-1.171405	-1.566297
C	3.498111	-2.087791	0.316510
H	4.048645	-1.842008	1.230558
H	2.461448	-2.308926	0.583502

H	3.939178	-2.989904	-0.113922
C	5.029377	-0.679197	-1.124395
H	5.447397	-1.579995	-1.580147
H	5.072172	0.135345	-1.850934
H	5.649127	-0.414460	-0.261538
C	0.305537	-0.408950	1.503037
H	1.380125	-0.547742	1.690445
C	-0.210769	0.679286	2.436404
H	0.290697	1.633625	2.262950
H	-1.290322	0.819222	2.321076
H	-0.019199	0.372531	3.467310
C	-0.400373	-1.742411	1.743466
H	-0.179402	-2.090254	2.756196
H	-1.484554	-1.637445	1.643214
H	-0.060016	-2.511256	1.041426
H	0.310515	-0.819755	-0.513079

44

TS5_conf_2_TS Eopt -771.869825

C	-2.200517	-0.529198	0.310283
C	-2.131701	-1.431145	1.487144
C	-1.013351	-1.567985	2.213347
C	0.245740	-0.891289	1.892920
C	0.590476	-0.522907	0.644295
H	-1.043660	-2.172338	3.114851
H	0.912180	-0.663671	2.717760
N	-0.303046	-0.902579	-0.418303
H	-3.063144	-1.910673	1.772845
C	1.826209	0.227071	0.316336
C	1.881116	1.098912	-0.779990
C	2.974316	0.076224	1.110080
C	3.042345	1.816475	-1.061955
H	1.018099	1.251205	-1.422037
C	4.133264	0.790563	0.825830
H	2.969437	-0.626156	1.937604
C	4.171746	1.666768	-0.260376
H	3.059159	2.493004	-1.910096
H	5.012817	0.652329	1.446233
H	5.077428	2.220901	-0.483774
H	-2.630283	-0.890966	-0.618973
N	-2.329625	0.760558	0.539851
H	-2.111415	1.099857	1.473349
C	-2.666953	1.764191	-0.484503
H	-2.454098	1.295838	-1.451768
C	-1.777339	2.988333	-0.299739
H	-1.981007	3.464406	0.664961
H	-0.717056	2.725967	-0.341117

H	-1.986671	3.716312	-1.086472
C	-4.150546	2.112757	-0.401636
H	-4.404226	2.837835	-1.179055
H	-4.769282	1.222435	-0.538441
H	-4.381805	2.554845	0.572457
C	-0.098480	-2.308475	-0.902083
H	-0.210875	-2.941932	-0.017071
C	-1.175634	-2.657082	-1.923283
H	-1.208335	-1.913409	-2.727648
H	-0.943634	-3.624611	-2.374083
H	-2.167409	-2.736869	-1.472801
C	1.301575	-2.491178	-1.485346
H	2.079675	-2.280181	-0.747746
H	1.421832	-3.524363	-1.820880
H	1.449396	-1.831197	-2.347704
H	-0.212011	-0.276636	-1.219168

44

TS5_conf_3_TS Eopt -771.869436

C	2.251438	0.803886	-0.257772
C	2.400154	1.062146	1.196654
C	1.458830	0.807388	2.114759
C	0.166212	0.201095	1.805167
C	-0.418727	0.271902	0.595283
H	1.686936	0.996522	3.159235
H	-0.319796	-0.364445	2.593486
N	0.209889	1.085853	-0.409753
H	3.371739	1.456899	1.482438
C	-1.690967	-0.411315	0.256942
C	-2.683249	-0.575857	1.234995
C	-1.929018	-0.907625	-1.032513
C	-3.873716	-1.230250	0.934443
H	-2.533550	-0.163155	2.227976
C	-3.120389	-1.566111	-1.330923
H	-1.181909	-0.813340	-1.816542
C	-4.096580	-1.729458	-0.349727
H	-4.633917	-1.338526	1.701243
H	-3.281658	-1.952055	-2.332075
H	-5.026942	-2.235832	-0.584925
H	2.407915	1.650451	-0.918852
N	2.585784	-0.365515	-0.769550
H	2.709645	-0.406023	-1.777274
C	2.657560	-1.659538	-0.069216
H	2.478309	-1.449315	0.987240
C	4.057177	-2.240311	-0.245735
H	4.812893	-1.561519	0.156773
H	4.128906	-3.196473	0.278141

H	4.270952	-2.415532	-1.305441
C	1.571505	-2.593305	-0.596479
H	1.633095	-3.554722	-0.080246
H	0.574939	-2.177064	-0.426505
H	1.702682	-2.773096	-1.668665
C	-0.113299	2.551077	-0.341510
H	0.508731	2.955401	0.465297
C	0.268999	3.194458	-1.669992
H	1.316508	3.040207	-1.937728
H	-0.356518	2.796026	-2.476555
H	0.098802	4.271672	-1.612419
C	-1.583473	2.815176	-0.022846
H	-2.230843	2.383319	-0.793722
H	-1.871841	2.405308	0.947849
H	-1.751493	3.894905	0.002324
H	-0.018043	0.747877	-1.345096

44

TS5_conf_4_TS Eopt -771.868674

C	2.158532	-0.716859	-0.284794
C	1.947599	-1.758870	-1.315085
C	0.803911	-1.899519	-1.995213
C	-0.415332	-1.131081	-1.750878
C	-0.709209	-0.511842	-0.593664
H	0.762207	-2.638223	-2.790087
H	-1.139412	-1.093875	-2.557921
N	0.247520	-0.572136	0.488556
H	2.813568	-2.380141	-1.526450
C	-1.991870	0.193847	-0.346013
C	-2.070887	1.272503	0.546609
C	-3.161967	-0.214834	-1.004907
C	-3.275422	1.941920	0.751575
H	-1.194501	1.618366	1.087471
C	-4.364149	0.455290	-0.801220
H	-3.137440	-1.078378	-1.661813
C	-4.425565	1.539307	0.075418
H	-3.310750	2.779444	1.440494
H	-5.258021	0.120285	-1.317345
H	-5.364368	2.058623	0.237471
H	2.548651	-1.013837	0.685762
N	2.489442	0.491565	-0.696243
H	2.304389	0.722182	-1.669330
C	2.988847	1.575366	0.166066
H	2.908434	1.209227	1.195363
C	2.112599	2.812267	-0.007729
H	1.066152	2.604674	0.232765
H	2.464802	3.608833	0.651389

H	2.163303	3.175791	-1.039122
C	4.452749	1.857049	-0.159426
H	5.060840	0.960386	-0.019137
H	4.554182	2.192641	-1.196426
H	4.832233	2.645522	0.495062
C	-0.167982	-1.482315	1.606771
H	-1.229452	-1.282926	1.807402
C	-0.008537	-2.941017	1.199483
H	1.045583	-3.197145	1.056950
H	-0.411273	-3.576197	1.992175
H	-0.551007	-3.163213	0.276858
C	0.635529	-1.139279	2.854731
H	0.283794	-1.750111	3.689130
H	1.701592	-1.346238	2.718432
H	0.514999	-0.087468	3.133101
H	0.359677	0.364171	0.881276

44

TS5_conf_5_TS Eopt -771.869359

C	1.709661	0.292189	-1.012678
C	1.661769	1.680242	-1.511098
C	0.475721	2.243498	-1.787867
C	-0.784588	1.553481	-1.513827
C	-0.925367	0.645678	-0.522910
H	0.435098	3.234092	-2.230397
H	-1.643657	1.814351	-2.121046
N	0.188611	0.458295	0.376450
H	2.603190	2.190684	-1.684589
C	-2.152814	-0.154945	-0.312815
C	-2.430667	-0.764220	0.919226
C	-3.058321	-0.345345	-1.370787
C	-3.593288	-1.511042	1.099890
H	-1.751768	-0.654161	1.757990
C	-4.218567	-1.088165	-1.188551
H	-2.842831	0.067272	-2.350685
C	-4.494089	-1.671659	0.049878
H	-3.790477	-1.967028	2.064451
H	-4.902436	-1.224978	-2.019825
H	-5.397346	-2.256536	0.189157
H	1.069107	-0.438906	-1.505770
N	2.829225	-0.181034	-0.511082
H	3.541800	0.479112	-0.210609
C	3.107975	-1.607913	-0.280815
H	2.279540	-2.150404	-0.747596
C	3.144632	-1.919400	1.212735
H	3.921510	-1.331087	1.712470
H	2.185297	-1.708359	1.690745

H	3.373249	-2.977988	1.357524
C	4.416100	-1.972543	-0.975224
H	5.249298	-1.412234	-0.538803
H	4.617938	-3.037908	-0.843105
H	4.365534	-1.753336	-2.044017
C	0.407263	1.542735	1.383653
H	0.810831	2.388796	0.817570
C	-0.869710	2.001732	2.085923
H	-1.664456	2.245033	1.375713
H	-1.236012	1.243502	2.784114
H	-0.646252	2.904363	2.660991
C	1.445632	1.071661	2.393587
H	2.391162	0.801883	1.919889
H	1.642274	1.872540	3.110195
H	1.073926	0.205707	2.954097
H	0.142569	-0.451153	0.834036

44

TS5_conf_6_TS Eopt -771.869021

C	2.104844	0.872807	-0.663296
C	1.850196	2.309881	-0.456629
C	0.629148	2.840267	-0.614405
C	-0.591852	2.063397	-0.811696
C	-0.753388	0.787150	-0.412964
H	0.512223	3.917334	-0.536400
H	-1.456089	2.583616	-1.212917
N	0.377313	0.076489	0.144387
H	2.708513	2.931870	-0.221015
C	-2.066045	0.098145	-0.443491
C	-2.165001	-1.243245	-0.840479
C	-3.230786	0.783267	-0.070901
C	-3.401950	-1.880211	-0.874653
H	-1.280542	-1.795914	-1.146577
C	-4.467610	0.142973	-0.100912
H	-3.161249	1.812897	0.267777
C	-4.556314	-1.189502	-0.502766
H	-3.464439	-2.915498	-1.193351
H	-5.359545	0.682483	0.200610
H	-5.519261	-1.689363	-0.522660
H	1.784970	0.446775	-1.611458
N	3.161754	0.323533	-0.095924
H	3.574537	0.813564	0.691928
C	3.738577	-1.002840	-0.394381
H	4.821532	-0.845883	-0.384331
C	3.333398	-1.497684	-1.775217
H	3.577024	-0.771028	-2.554621
H	3.881449	-2.418433	-1.984756

H	2.266171	-1.737802	-1.829758
C	3.376919	-2.002259	0.703048
H	3.912922	-2.939741	0.535995
H	3.648727	-1.622701	1.691716
H	2.303605	-2.220725	0.693909
C	0.411134	-0.099000	1.630086
H	1.478660	-0.237475	1.851909
C	-0.077668	1.146103	2.359817
H	0.457238	2.042056	2.039188
H	-1.150675	1.298350	2.205917
H	0.092075	1.008447	3.430293
C	-0.342818	-1.347606	2.085637
H	-0.024497	-2.237882	1.533093
H	-0.143079	-1.521243	3.146531
H	-1.421784	-1.225949	1.952862
H	0.443329	-0.847773	-0.283917

44

TS5_conf_7_TS Eopt -771.868049

C	2.128040	0.737770	-0.505544
C	1.888039	2.191685	-0.442863
C	0.693716	2.739385	-0.708166
C	-0.543272	1.990119	-0.906728
C	-0.763819	0.746210	-0.442130
H	0.611121	3.822312	-0.724490
H	-1.372796	2.511559	-1.374131
N	0.318264	0.026541	0.194283
H	2.749380	2.812538	-0.214676
C	-2.097256	0.099246	-0.491530
C	-3.251025	0.838939	-0.198300
C	-2.226803	-1.255086	-0.831790
C	-4.507245	0.238829	-0.250254
H	-3.159195	1.880511	0.095636
C	-3.482655	-1.852024	-0.887874
H	-1.350270	-1.850299	-1.074557
C	-4.626156	-1.107167	-0.595029
H	-5.391178	0.820811	-0.010561
H	-3.568327	-2.898392	-1.161934
H	-5.604333	-1.575502	-0.632072
H	1.902946	0.234404	-1.445936
N	3.107886	0.222406	0.213762
H	3.475097	0.777249	0.979131
C	3.739366	-1.092248	-0.030679
H	4.366873	-1.266951	0.845893
C	4.621283	-1.024882	-1.276003
H	5.367970	-0.233396	-1.180288
H	5.137080	-1.978155	-1.415541

H	4.017671	-0.830984	-2.168736
C	2.706044	-2.211741	-0.119346
H	2.072113	-2.105129	-1.006674
H	3.222875	-3.169607	-0.210817
H	2.082433	-2.252021	0.778399
C	0.275020	-0.106312	1.681937
H	1.322343	-0.303869	1.953265
C	-0.162178	1.193136	2.346331
H	-0.046786	1.088453	3.427645
H	0.442327	2.040287	2.015517
H	-1.215636	1.406774	2.138954
C	-0.571788	-1.291570	2.144361
H	-0.418106	-1.444339	3.216041
H	-1.636083	-1.109124	1.971133
H	-0.289729	-2.215332	1.628035
H	0.383309	-0.910165	-0.203907

44

TS5_conf_8_TS Eopt -771.868748

C	2.265772	0.762104	-0.109194
C	2.319517	0.945059	1.360461
C	1.334488	0.656189	2.218312
C	0.026793	0.119774	1.851344
C	-0.515798	0.201093	0.623925
H	1.518507	0.806546	3.277865
H	-0.539787	-0.368214	2.637617
N	0.225690	0.870333	-0.420482
H	3.268693	1.338824	1.717030
C	-1.860990	-0.327567	0.285437
C	-2.870745	-0.365427	1.259390
C	-2.153613	-0.800132	-1.002212
C	-4.126314	-0.885141	0.960682
H	-2.679981	0.037481	2.249017
C	-3.411612	-1.319850	-1.299931
H	-1.403913	-0.786527	-1.788932
C	-4.401074	-1.367661	-0.319624
H	-4.895671	-0.900325	1.725809
H	-3.614426	-1.688702	-2.299966
H	-5.381743	-1.769063	-0.553104
H	2.431516	1.650666	-0.712629
N	2.688356	-0.360157	-0.665901
H	2.883989	-0.332010	-1.662510
C	2.710778	-1.698131	-0.051902
H	2.592167	-1.546804	1.023620
C	4.062812	-2.344505	-0.333992
H	4.103525	-3.332046	0.131183
H	4.210754	-2.470760	-1.411995

H 4.877389 -1.734791 0.063649
C 1.550202 -2.536579 -0.584892
H 1.578000 -3.532325 -0.134984
H 0.585059 -2.083923 -0.339144
H 1.623583 -2.649107 -1.671456
C -0.340132 2.213005 -0.781335
H -1.430919 2.097859 -0.838790
C -0.008180 3.238535 0.294503
H -0.347506 2.910707 1.280519
H 1.068394 3.429573 0.340189
H -0.511251 4.178811 0.056578
C 0.176934 2.621005 -2.155082
H 1.258542 2.786080 -2.151271
H -0.059713 1.865763 -2.911030
H -0.298551 3.557786 -2.454181
H 0.212000 0.292642 -1.263728
44
TS5_conf_9_TS Eopt -771.869681
C 1.802754 0.130553 -0.946473
C 1.737585 1.393018 -1.711279
C 0.554642 1.880155 -2.113928
C -0.714011 1.252125 -1.748899
C -0.884255 0.539312 -0.615038
H 0.523312 2.771744 -2.732807
H -1.561764 1.414757 -2.404521
N 0.208886 0.478099 0.322241
H 2.675605 1.878758 -1.959496
C -2.136367 -0.172949 -0.273840
C -3.039905 -0.538095 -1.286184
C -2.442074 -0.520545 1.050112
C -4.224435 -1.196368 -0.977690
H -2.804394 -0.329012 -2.324428
C -3.629257 -1.182380 1.357839
H -1.765216 -0.268654 1.860472
C -4.526954 -1.518241 0.347167
H -4.906974 -1.471949 -1.774960
H -3.848441 -1.434114 2.390214
H -5.449559 -2.036931 0.586220
H 1.236381 -0.716619 -1.326890
N 2.900372 -0.139953 -0.273077
H 3.523695 0.635669 -0.064632
C 3.238769 -1.419873 0.379122
H 2.991892 -1.314759 1.443609
C 4.741754 -1.637035 0.237740
H 5.304974 -0.790547 0.640752
H 5.035632 -2.532001 0.790080

H 5.008581 -1.769148 -0.814894
C 2.438312 -2.575865 -0.202478
H 1.363638 -2.476842 -0.026565
H 2.618587 -2.675057 -1.277653
H 2.763233 -3.497465 0.284370
C 0.486886 1.677011 1.173971
H 1.156148 2.316198 0.584982
C -0.753570 2.493177 1.528556
H -0.434729 3.398621 2.051559
H -1.310218 2.797788 0.639186
H -1.422810 1.938868 2.192684
C 1.217580 1.206523 2.425946
H 2.121097 0.642376 2.182382
H 1.511252 2.070001 3.026707
H 0.565157 0.573980 3.039102
H 0.135382 -0.355818 0.902507
44
TS_S1_conf_1_ts Eopt -771.897200
C 1.574562 1.157205 0.459523
C 1.975522 0.846545 1.811326
C 1.142551 0.120497 2.602708
C -0.120421 -0.308640 2.105713
C -0.554403 0.133152 0.886717
H 1.433296 -0.131260 3.617481
H -0.746480 -0.983826 2.675329
N 0.249244 0.974387 0.132252
H 2.940651 1.200788 2.154262
C -1.857537 -0.317083 0.337277
C -1.923132 -0.914468 -0.929031
C -3.016732 -0.215691 1.112194
C -3.134243 -1.400183 -1.412192
H -1.021004 -1.009632 -1.528545
C -4.229579 -0.700985 0.624283
H -2.967725 0.254389 2.089912
C -4.290451 -1.292600 -0.636430
H -3.174790 -1.867008 -2.390844
H -5.126330 -0.612111 1.228682
H -5.234882 -1.669875 -1.014995
H 1.999976 2.053369 0.019118
N 2.669328 0.042283 -0.647810
H 2.237243 0.006184 -1.573403
C 2.980016 -1.327056 -0.170095
H 3.180098 -1.233513 0.903495
C 4.226625 -1.864312 -0.868547
H 4.475579 -2.851970 -0.471871
H 4.053635 -1.960203 -1.945410

H	5.085821	-1.206798	-0.708359
C	1.775769	-2.232883	-0.386279
H	2.002077	-3.237165	-0.020874
H	0.894210	-1.872791	0.148906
H	1.538185	-2.306579	-1.454031
C	-0.336672	1.880056	-0.901989
H	-1.362746	1.543379	-1.038246
C	-0.375511	3.311303	-0.368218
H	0.628287	3.727326	-0.239441
H	-0.909123	3.945170	-1.080652
H	-0.898093	3.348696	0.591050
C	0.386845	1.773014	-2.239326
H	1.420263	2.130773	-2.179064
H	0.377562	0.747651	-2.621263
H	-0.128666	2.403400	-2.967554
H	3.533709	0.577697	-0.750579

44

TS_S1_conf_2_ts Eopt -771.897698

C	-1.408589	0.387032	-0.844459
C	-1.750959	-0.607123	-1.834276
C	-0.862311	-1.607089	-2.079193
C	0.396476	-1.616395	-1.414456
C	0.791622	-0.520903	-0.693634
H	-1.098768	-2.392890	-2.789240
H	1.073273	-2.456430	-1.505965
N	-0.073875	0.548941	-0.546843
H	-2.709942	-0.541819	-2.334338
C	2.129550	-0.472601	-0.051324
C	3.279232	-0.706697	-0.811010
C	2.244008	-0.258322	1.328772
C	4.532280	-0.716808	-0.198605
H	3.190323	-0.867964	-1.881225
C	3.495048	-0.271834	1.937635
H	1.349871	-0.096031	1.925490
C	4.641983	-0.498564	1.173655
H	5.421149	-0.891862	-0.795786
H	3.574868	-0.111069	3.007736
H	5.617494	-0.506378	1.648959
H	-1.962665	1.319439	-0.864129
N	-2.190208	-0.259390	0.780109
H	-1.551618	-0.999712	1.078255
C	-3.571729	-0.779998	0.652915
H	-3.535232	-1.518919	-0.155227
C	-4.501334	0.362589	0.263374
H	-5.525887	-0.007296	0.184238
H	-4.482940	1.150632	1.024529

H	-4.230219	0.803236	-0.700002
C	-4.014671	-1.470071	1.939764
H	-3.328865	-2.276833	2.212755
H	-4.060375	-0.753153	2.765995
H	-5.010627	-1.899620	1.804162
C	0.399901	1.915340	-0.179012
H	1.457790	1.804943	0.053828
C	0.272111	2.844919	-1.384756
H	0.719221	3.811712	-1.140747
H	0.793993	2.427090	-2.249212
H	-0.774037	3.019437	-1.654805
C	-0.317248	2.456569	1.053131
H	-1.382969	2.626135	0.864569
H	-0.202591	1.789259	1.912030
H	0.118736	3.422531	1.318094
H	-2.143617	0.478445	1.486048

44

TS_S1_conf_3_ts Eopt -771.896798

C	1.714924	-1.255000	-0.015759
C	1.942596	-1.819192	-1.329995
C	0.982750	-1.703689	-2.281582
C	-0.241303	-1.037826	-1.980811
C	-0.507464	-0.650113	-0.698298
H	1.137770	-2.119773	-3.271561
H	-0.971865	-0.835987	-2.754023
N	0.431855	-0.875705	0.302851
H	2.879508	-2.334416	-1.506946
C	-1.790544	0.019537	-0.363778
C	-1.812082	1.248256	0.310114
C	-2.995668	-0.550205	-0.787179
C	-3.019556	1.895092	0.553694
H	-0.882328	1.704913	0.638333
C	-4.205048	0.098171	-0.539514
H	-2.983183	-1.507761	-1.299223
C	-4.219450	1.320478	0.129962
H	-3.023180	2.849233	1.070266
H	-5.134710	-0.355982	-0.866370
H	-5.160803	1.824698	0.322565
H	2.238327	-1.736357	0.803808
N	2.852653	0.282603	0.080799
H	3.331395	0.239216	0.982213
C	2.213382	1.608474	-0.097383
H	1.258072	1.550277	0.435752
C	1.945113	1.864180	-1.573487
H	2.888046	1.934639	-2.126620
H	1.334691	1.074457	-2.017085

H	1.411475	2.810627	-1.687797
C	3.065718	2.706643	0.532107
H	4.037417	2.772664	0.032507
H	2.559756	3.670060	0.430215
H	3.231058	2.519769	1.597422
C	0.007209	-1.022171	1.730818
H	-0.975617	-0.559501	1.796673
C	-0.137481	-2.506927	2.059433
H	-0.537704	-2.617604	3.070296
H	-0.823449	-2.989916	1.358141
H	0.827112	-3.022986	2.020117
C	0.941103	-0.297548	2.692621
H	0.528356	-0.379562	3.700626
H	1.940014	-0.744179	2.719955
H	1.025887	0.765941	2.453932
H	3.567195	0.130290	-0.634534

44
TS_S1_conf_4_ts Eopt -771.895186

C	1.394619	0.164324	0.983265
C	1.760889	-1.092714	1.592730
C	0.902842	-2.141251	1.477302
C	-0.346301	-1.965008	0.818338
C	-0.769878	-0.703677	0.494113
H	1.158565	-3.111229	1.891558
H	-0.994307	-2.806586	0.608500
N	0.058602	0.379058	0.734180
H	2.713672	-1.169903	2.102125
C	-2.095083	-0.481343	-0.136597
C	-3.251035	-0.998193	0.455007
C	-2.185610	0.188940	-1.363863
C	-4.487447	-0.838364	-0.170250
H	-3.180717	-1.513076	1.408573
C	-3.419908	0.344759	-1.986670
H	-1.283769	0.572381	-1.834737
C	-4.573739	-0.166947	-1.388771
H	-5.382009	-1.235861	0.297843
H	-3.481321	0.859291	-2.939990
H	-5.536395	-0.043254	-1.874128
H	1.927021	1.047752	1.323241
N	2.223007	0.163306	-0.743511
H	1.703916	-0.510108	-1.309872
C	3.677509	-0.151157	-0.712270
H	4.010421	0.038766	0.316751
C	4.444027	0.770158	-1.654537
H	4.285088	1.821494	-1.396793
H	5.514805	0.562545	-1.586180

H	4.126298	0.608214	-2.689534
C	3.894531	-1.621535	-1.046773
H	3.316490	-2.273099	-0.386527
H	3.599558	-1.820639	-2.082946
H	4.952100	-1.873236	-0.938575
C	-0.466268	1.771298	0.855497
H	-1.515987	1.712779	0.572004
C	-0.391031	2.218484	2.314498
H	-0.870524	3.195180	2.416216
H	-0.909054	1.506032	2.961335
H	0.644046	2.316023	2.656031
C	0.239065	2.739443	-0.088226
H	-0.235061	3.719795	-0.001630
H	1.295157	2.871016	0.171345
H	0.156775	2.420507	-1.131302
H	2.050881	1.085374	-1.144756

44
TS_S1_conf_5_ts Eopt -771.894682

C	1.613384	-1.241732	-0.043185
C	1.654571	-2.275742	-1.058447
C	0.600340	-2.382862	-1.905500
C	-0.527468	-1.521963	-1.737495
C	-0.646700	-0.769527	-0.603247
H	0.601280	-3.115819	-2.705397
H	-1.319033	-1.488867	-2.475847
N	0.379217	-0.766710	0.337585
H	2.538352	-2.898034	-1.130336
C	-1.857807	0.055057	-0.356950
C	-1.744203	1.426679	-0.094371
C	-3.127395	-0.522241	-0.457069
C	-2.884798	2.206591	0.069220
H	-0.760417	1.884713	-0.032543
C	-4.269501	0.260156	-0.289511
H	-3.217100	-1.586704	-0.654637
C	-4.150176	1.623537	-0.025595
H	-2.786687	3.269231	0.265377
H	-5.250402	-0.198014	-0.362004
H	-5.039169	2.232019	0.104865
H	2.325863	-1.320987	0.768673
N	2.453191	0.216783	-0.957131
H	3.363317	-0.115102	-1.281418
C	2.565935	1.490850	-0.215356
H	1.666783	1.545458	0.413023
C	2.567827	2.685059	-1.165212
H	3.438586	2.646336	-1.827050
H	1.661153	2.705136	-1.777029

H	2.611469	3.614108	-0.590980
C	3.812383	1.450132	0.659392
H	3.851578	0.548514	1.277772
H	4.711174	1.476102	0.034097
H	3.832366	2.318416	1.321932
C	0.030284	-0.580615	1.786597
H	-0.635980	0.281554	1.827454
C	-0.719628	-1.824306	2.261806
H	-1.610402	-2.007069	1.654382
H	-0.068323	-2.702419	2.202089
H	-1.033884	-1.692178	3.300232
C	1.230475	-0.286347	2.675085
H	0.847242	-0.043604	3.668714
H	1.892983	-1.149421	2.787951
H	1.808867	0.572353	2.328706
H	1.858388	0.317412	-1.782157

44

TS_S1_conf_6_ts Eopt -771.890913

C	1.613333	1.364976	0.241664
C	2.054418	1.253496	1.609894
C	1.286543	0.587363	2.511053
C	0.035571	0.041875	2.109669
C	-0.437478	0.271739	0.847913
H	1.609443	0.490825	3.542531
H	-0.570445	-0.537723	2.794401
N	0.311611	1.009920	-0.052649
H	2.997172	1.715409	1.878917
C	-1.762918	-0.295462	0.461060
C	-1.824812	-1.557366	-0.139708
C	-2.945063	0.374877	0.783545
C	-3.057625	-2.121537	-0.456438
H	-0.906315	-2.090364	-0.369186
C	-4.179528	-0.189113	0.460916
H	-2.899007	1.335162	1.289058
C	-4.236695	-1.432691	-0.166517
H	-3.097734	-3.097889	-0.928241
H	-5.093646	0.341352	0.706704
H	-5.197266	-1.870899	-0.417464
H	1.950604	2.238861	-0.306178
N	2.785186	0.247621	-0.781173
H	2.300937	0.007665	-1.650078
C	3.312017	-0.976047	-0.131543
H	3.630313	-0.669627	0.871646
C	4.516674	-1.514061	-0.899195
H	4.926113	-2.385098	-0.380969
H	4.224335	-1.821174	-1.908569

H	5.306595	-0.761553	-0.976588
C	2.202129	-2.010121	-0.013826
H	2.591022	-2.916333	0.456152
H	1.374240	-1.643382	0.597232
H	1.821165	-2.279078	-1.006307
C	-0.220979	1.573484	-1.336979
H	0.676190	1.950068	-1.834282
C	-0.851516	0.561016	-2.292961
H	-0.288026	-0.375979	-2.322935
H	-1.889003	0.338791	-2.039625
H	-0.834814	1.001283	-3.293512
C	-1.134068	2.764023	-1.058574
H	-2.069270	2.441369	-0.595990
H	-0.643456	3.488716	-0.403507
H	-1.375700	3.256902	-2.003967
H	3.558953	0.861201	-1.042331

44

TS_S1_conf_7_ts Eopt -771.890186

C	-1.502289	0.808365	0.644783
C	-1.716527	2.187218	0.268426
C	-0.730455	2.846861	-0.390896
C	0.499537	2.183281	-0.661430
C	0.758168	0.961343	-0.104256
H	-0.869411	3.875166	-0.707619
H	1.260042	2.643277	-1.279796
N	-0.202843	0.350459	0.691205
H	-2.671114	2.643791	0.502046
C	2.069077	0.305888	-0.359784
C	3.245427	0.921997	0.080424
C	2.147107	-0.875618	-1.103708
C	4.482552	0.336723	-0.177931
H	3.183552	1.846529	0.647251
C	3.386026	-1.458867	-1.363646
H	1.244057	-1.328485	-1.501421
C	4.554058	-0.860320	-0.891422
H	5.389068	0.813356	0.180539
H	3.437768	-2.375442	-1.942167
H	5.517644	-1.318515	-1.089376
H	-2.118864	0.413010	1.447490
N	-2.404849	-0.201438	-0.709373
H	-2.579203	0.445310	-1.482557
C	-3.669242	-0.845613	-0.273060
H	-3.422283	-1.418595	0.629339
C	-4.190956	-1.798830	-1.343454
H	-4.441854	-1.249092	-2.256338
H	-3.449061	-2.564449	-1.587867

H -5.093947 -2.299740 -0.985159
C -4.686188 0.233617 0.077504
H -4.896754 0.860480 -0.796054
H -5.621282 -0.233606 0.393540
H -4.342243 0.875729 0.892650
C 0.059545 -0.761303 1.665637
H -0.705108 -0.595647 2.429154
C -0.170882 -2.151276 1.077138
H -0.096171 -2.883952 1.885236
H -1.167334 -2.253804 0.638268
H 0.580203 -2.400058 0.325650
C 1.407542 -0.654254 2.375204
H 1.639983 0.381764 2.635901
H 1.335968 -1.230715 3.301065
H 2.226845 -1.066760 1.784298
H -1.741652 -0.897973 -1.054069
44
TS_S1_conf_8_ts Eopt -771.892374
C 1.572546 -1.263681 0.205421
C 1.636263 -2.404931 -0.685600
C 0.623230 -2.597563 -1.567223
C -0.499276 -1.717631 -1.551599
C -0.653557 -0.831696 -0.522676
H 0.653080 -3.418580 -2.275854
H -1.262735 -1.771082 -2.317648
N 0.331543 -0.727730 0.454705
H 2.509882 -3.044623 -0.644397
C -1.873459 0.015892 -0.438617
C -1.778103 1.412375 -0.399068
C -3.136802 -0.583586 -0.480320
C -2.928962 2.195484 -0.388712
H -0.801111 1.886425 -0.396999
C -4.288668 0.201580 -0.467248
H -3.213882 -1.666848 -0.510359
C -4.186825 1.591121 -0.418860
H -2.842968 3.276843 -0.364056
H -5.263673 -0.273935 -0.492134
H -5.083370 2.202398 -0.408100
H 2.233012 -1.274543 1.063475
N 2.608564 0.009355 -0.781423
H 3.381619 -0.569417 -1.113913
C 3.135800 1.243492 -0.144618
H 3.498160 0.941358 0.845117
C 2.017476 2.266579 -0.002020
H 1.177151 1.876348 0.576993
H 2.392351 3.156865 0.507858

H 1.650903 2.568455 -0.989780
C 4.308597 1.800385 -0.947277
H 3.983136 2.085353 -1.952813
H 4.709066 2.688060 -0.450592
H 5.113531 1.065126 -1.032911
C -0.048052 -0.254253 1.828210
H -0.591257 0.683148 1.692882
C -0.973365 -1.293285 2.461657
H -1.867022 -1.462669 1.856975
H -0.441145 -2.242802 2.576769
H -1.289236 -0.947573 3.449181
C 1.146303 0.013457 2.735106
H 1.877018 0.693424 2.292869
H 0.764561 0.487525 3.641812
H 1.649236 -0.909369 3.037654
H 2.044069 0.236304 -1.604331
44
TS_S1_conf_9_ts Eopt -771.891141
C 1.778278 -1.254959 0.654246
C 2.076039 -2.282304 -0.322739
C 1.171892 -2.562572 -1.293117
C -0.048446 -1.828773 -1.350513
C -0.378521 -0.963658 -0.346275
H 1.371245 -3.336088 -2.027209
H -0.726241 -1.936216 -2.188354
N 0.480053 -0.801816 0.739404
H 3.015292 -2.814694 -0.227348
C -1.643372 -0.188624 -0.436251
C -1.631422 1.209484 -0.465558
C -2.859670 -0.867336 -0.573302
C -2.823461 1.920263 -0.590848
H -0.689869 1.746782 -0.415441
C -4.050585 -0.156296 -0.696351
H -2.869000 -1.953347 -0.555403
C -4.035920 1.239144 -0.696116
H -2.801899 3.004871 -0.614613
H -4.990043 -0.691764 -0.786820
H -4.964421 1.793239 -0.787806
H 2.246514 -1.377310 1.625503
N 2.938751 0.204069 0.215664
H 3.365346 0.506094 1.093319
C 2.341413 1.360969 -0.494919
H 1.368085 1.526671 -0.020930
C 2.136511 1.020625 -1.964078
H 1.628901 1.850184 -2.462128
H 3.101624 0.865575 -2.458716

H 1.527128 0.123270 -2.092417
C 3.198805 2.610124 -0.311862
H 2.725595 3.457389 -0.814772
H 3.315449 2.865178 0.745622
H 4.191483 2.460510 -0.748114
C 0.036914 -0.363536 2.109937
H 0.686889 -0.946393 2.768236
C 0.299584 1.111333 2.399720
H 1.350262 1.377784 2.256146
H -0.325815 1.762910 1.785514
H 0.055743 1.302032 3.448397
C -1.395328 -0.767708 2.452018
H -1.625750 -1.773228 2.089493
H -1.480806 -0.770886 3.541532
H -2.134530 -0.068000 2.058828
H 3.690923 -0.207383 -0.341948
43
TS_S2 Eopt -1937.452696
C -2.285452 -1.507554 2.531575
C -2.697816 -1.317764 1.073694
C -3.539938 -2.467070 0.539209
N -1.473772 -1.064662 0.241780
C -1.073090 0.276472 -0.067022
C -2.140181 1.302416 -0.197874
C -3.268922 1.074163 -0.996367
C -4.230767 2.066934 -1.154462
C -4.076967 3.298782 -0.514520
C -2.958124 3.530866 0.283154
C -1.993725 2.535906 0.443436
C 0.204933 0.579564 -0.336266
C 1.324122 -0.408382 -0.269793
C 0.893402 -1.717472 0.385098
C -0.554337 -2.038091 0.147334
H -3.176617 -1.557195 3.161913
H -1.722644 -2.436992 2.670418
H -1.668080 -0.670077 2.869811
H -3.286002 -0.404733 1.000495
H -3.889198 -2.268259 -0.476643
H -4.416079 -2.580249 1.182013
H -3.004747 -3.421883 0.553818
H -3.393818 0.118838 -1.499670
H -5.098151 1.880736 -1.779286
H -4.829052 4.071536 -0.636222
H -2.836516 4.483360 0.788621
H -1.127666 2.707744 1.075829
H 0.440205 1.584663 -0.669227

H 0.955906 -1.608579 1.477903
H 1.528212 -2.556388 0.099535
H 1.758363 -0.564401 -1.266182
S 2.724849 0.291499 0.663456
O 3.082961 1.540251 -0.058644
O 2.239652 0.504346 2.040330
O 3.803454 -0.726972 0.554148
K 5.581167 0.772674 -0.894501
O -0.440296 -2.610773 -1.569949
C -1.677805 -2.712654 -2.304779
H -2.240049 -1.781638 -2.215415
H -1.437070 -2.915121 -3.347273
H -2.228696 -3.547018 -1.876214
H -0.877341 -2.999751 0.536756
H 0.187510 -2.058266 -2.064779
43
TS_S3_conf_10_ts Eopt -3046.096212
C 2.275864 2.841715 1.943251
C 2.483846 1.813269 0.839587
C 2.582742 2.484016 -0.530939
N 1.429138 0.755811 0.845192
C 1.646503 -0.456650 0.111204
C 3.041339 -0.805098 -0.280161
C 4.008184 -1.069367 0.698326
C 5.290508 -1.465205 0.328339
C 5.618142 -1.604508 -1.022009
C 4.657297 -1.351755 -1.999176
C 3.372524 -0.952864 -1.629711
C 0.637134 -1.289504 -0.179447
C -0.763825 -1.053701 0.293489
C -0.749302 -0.139102 1.512987
C 0.198016 1.012043 1.316598
H 3.161616 3.479780 1.973618
H 1.414942 3.488692 1.746724
H 2.164998 2.370331 2.923374
H 3.418669 1.290905 1.041309
H 2.667595 1.747602 -1.335965
H 3.480732 3.106433 -0.560504
H 1.725225 3.143161 -0.703125
H 3.748326 -0.977252 1.750034
H 6.031609 -1.672134 1.093520
H 6.618150 -1.913227 -1.309088
H 4.904867 -1.461666 -3.050016
H 2.620911 -0.751940 -2.388340
H 0.856371 -2.194366 -0.736770
H -0.368978 -0.698105 2.379643

H	-1.745199	0.232615	1.754993
H	-1.402213	-0.656846	-0.505488
S	-1.554407	-2.627639	0.726780
O	-1.510201	-3.439880	-0.519071
O	-0.777016	-3.198425	1.844702
O	-2.953142	-2.268647	1.087626
K	-4.205114	-3.504484	-1.008235
H	0.131671	1.817998	2.039785
S	-0.979595	2.155647	-0.241818
O	-0.697780	3.611586	-0.297804
O	-0.458640	1.523594	-1.676266
O	-2.421896	1.824221	-0.269547
K	-3.402985	4.443349	-0.447282
H	0.420207	1.871455	-1.905787

43

TS_S3_conf_1_ts Eopt -3046.096929

C	-2.370615	-0.661947	-3.451237
C	-2.624826	-0.219701	-2.011810
C	-3.477061	1.042481	-1.924480
N	-1.323733	-0.054725	-1.303832
C	-0.916875	-0.886057	-0.225085
C	-1.954275	-1.710753	0.449896
C	-1.808419	-3.097359	0.528630
C	-2.757008	-3.862945	1.208581
C	-3.852062	-3.245453	1.810043
C	-3.997927	-1.857750	1.736016
C	-3.053473	-1.090969	1.059774
C	0.338112	-0.840643	0.251899
C	1.425930	0.004655	-0.330219
C	0.992432	0.706926	-1.616682
C	-0.493575	0.929091	-1.684580
H	-3.324539	-0.877269	-3.939257
H	-1.868284	0.121126	-4.028844
H	-1.754266	-1.564419	-3.475087
H	-3.139889	-1.026200	-1.491660
H	-3.011471	1.884475	-2.448624
H	-3.647557	1.333360	-0.885797
H	-4.440559	0.852743	-2.403767
H	-0.955733	-3.572169	0.051894
H	-2.640069	-4.940456	1.263739
H	-4.590550	-3.841352	2.336831
H	-4.845324	-1.373600	2.210965
H	-3.145574	-0.008558	1.008089
H	0.572777	-1.427747	1.133373
H	1.205729	0.061122	-2.480946
H	1.528627	1.645432	-1.763286
H	1.780484	0.729523	0.411497
S	2.894686	-1.011545	-0.668574
O	3.280975	-1.592488	0.646219
O	2.494660	-2.015658	-1.674698
O	3.925914	-0.054030	-1.156634
K	5.749684	-0.426708	0.852347
H	-0.837477	1.518495	-2.531823
S	-0.773116	2.500806	-0.080160
O	0.460108	2.902736	0.641054
O	-1.373014	3.866929	-0.771355
O	-1.882426	2.080918	0.803038
K	-0.426511	2.001463	3.181755
H	-0.657481	4.355351	-1.214132

43

TS_S3_conf_2_ts Eopt -3046.096612

C	1.992295	-0.859125	3.535517
C	2.429245	-0.338025	2.168428
C	3.354493	0.869997	2.265764
N	1.222293	-0.051804	1.342923
C	0.838824	-0.863367	0.240887
C	1.882632	-1.710020	-0.394755
C	1.675307	-3.082315	-0.550637
C	2.633784	-3.865899	-1.195049
C	3.800629	-3.281179	-1.683574
C	4.009235	-1.907834	-1.531226
C	3.055262	-1.122981	-0.890050
C	-0.390295	-0.771840	-0.293349
C	-1.479205	0.093742	0.253888
C	-1.069109	0.819717	1.536700
C	0.421374	0.975371	1.672530
H	1.449688	-0.090093	4.095698
H	1.345203	-1.733694	3.426875
H	2.871532	-1.144566	4.118824
H	2.956535	-1.135383	1.646536
H	2.915478	1.672798	2.868024
H	3.597722	1.260234	1.274512
H	4.279052	0.565983	2.762490
H	0.767519	-3.532620	-0.159495
H	2.468902	-4.932443	-1.309349
H	4.546986	-3.891496	-2.181959
H	4.914007	-1.448928	-1.917003
H	3.198627	-0.050594	-0.776025
H	-0.602361	-1.338248	-1.193916
H	-1.350559	0.215290	2.411492
H	-1.567174	1.785820	1.629358
H	-1.809788	0.805993	-0.509421

S -2.961457 -0.906735 0.581738
O -3.332940 -1.501112 -0.731367
O -2.584196 -1.901530 1.606118
O -3.992398 0.064186 1.043241
K -5.790330 -0.318127 -0.986250
H 0.748807 1.556506 2.532563
S 0.832805 2.505186 0.055945
O -0.307475 2.780019 -0.845383
O 1.116576 3.980984 0.724199
O 2.083755 2.116294 -0.639478
K 1.051339 1.800221 -3.196689
H 2.019614 4.014894 1.084170
43
TS_S3_conf_3_ts Eopt -3046.096550
C 0.279615 1.573821 2.724676
C -0.768657 0.589634 2.208009
C -0.999657 -0.534910 3.208246
N -0.353571 0.084892 0.863256
C -0.109721 1.047186 -0.167926
C -0.995205 2.235038 -0.245261
C -0.449749 3.502922 -0.474923
C -1.283212 4.610368 -0.618995
C -2.667078 4.460672 -0.529320
C -3.214871 3.198468 -0.294121
C -2.385667 2.088288 -0.151493
C 0.884810 0.830206 -1.040944
C 1.668122 -0.445643 -1.005735
C 0.742563 -1.586474 -0.583942
C -0.069524 -1.198600 0.639139
H 0.396177 2.431646 2.057193
H -0.033906 1.947799 3.702821
H 1.244512 1.069060 2.816304
H -1.719764 1.107218 2.057765
H -1.724419 -1.272240 2.852855
H -1.405396 -0.086096 4.117451
H -0.065331 -1.037313 3.478370
H 0.629370 3.620281 -0.524157
H -0.850419 5.590471 -0.791863
H -3.315736 5.324142 -0.636705
H -4.291240 3.077425 -0.223719
H -2.805380 1.100544 0.025618
H 1.067993 1.548203 -1.831789
H 1.296102 -2.502326 -0.362544
H 0.080381 -1.800516 -1.428619
H 2.093838 -0.660417 -1.988365
S 3.123125 -0.359553 0.088631
O 3.832847 0.898726 -0.265678
O 2.604143 -0.393032 1.472372
O 3.939097 -1.554403 -0.265836
K 6.176761 -0.221478 -1.127161
H 0.052850 -1.814033 1.522107
S -2.059725 -2.151816 0.135554
O -2.222801 -2.798457 -1.194644
O -2.423879 -3.311391 1.257388
O -3.096908 -1.129891 0.421988
K -4.499135 -1.349852 -1.975765
H -1.914533 -4.119195 1.073928
43
TS_S3_conf_4_ts Eopt -3046.096075
C 1.207290 0.070712 3.013360
C 0.009431 0.621999 2.244732
C -1.289605 0.496632 3.033328
N -0.082204 -0.033420 0.905063
C 0.272540 0.670574 -0.283470
C 0.026541 2.133763 -0.333520
C 1.003629 2.983207 -0.862858
C 0.767002 4.353869 -0.960923
C -0.445062 4.886413 -0.524386
C -1.422912 4.042410 0.006610
C -1.191575 2.673070 0.102724
C 0.767979 -0.003428 -1.332987
C 1.031529 -1.471568 -1.263345
C 0.048260 -2.189156 -0.334780
C -0.415555 -1.327185 0.827608
H 1.065828 -0.986602 3.261507
H 2.120997 0.159737 2.424053
H 1.317829 0.625808 3.948722
H 0.189045 1.676331 2.034440
H -1.186014 1.059390 3.964293
H -1.503966 -0.543108 3.302208
H -2.140851 0.892260 2.475451
H 1.952313 2.567969 -1.189989
H 1.533295 5.004346 -1.370109
H -0.627517 5.953916 -0.595201
H -2.370731 4.451668 0.341714
H -1.956679 2.006504 0.490975
H 0.983559 0.529862 -2.251178
H 0.506176 -3.089971 0.085439
H -0.809410 -2.527530 -0.921915
H 0.988020 -1.918770 -2.259271
S 2.751689 -1.794286 -0.744530
O 2.870705 -1.289565 0.651779

O 2.923701 -3.257336 -0.859831
O 3.606374 -1.008244 -1.675857
K 4.677679 0.706353 0.167708
H -0.490783 -1.828566 1.789310
S -2.649218 -1.339500 0.462074
O -3.117574 -2.242957 -0.622750
O -3.440490 -1.828409 1.825197
O -3.123601 0.055832 0.311820
K -4.529441 -0.282255 -2.076456
H -3.421582 -2.799023 1.884164
43
TS_S3_conf_6_ts Eopt -3046.092883
C -1.865702 0.917597 2.743605
C -0.425595 0.680876 2.295070
C 0.445213 1.920256 2.481362
N -0.297868 0.143062 0.901364
C -0.087307 1.027757 -0.203817
C -1.011591 2.173400 -0.378152
C -2.393861 1.976023 -0.274282
C -3.268698 3.033975 -0.513141
C -2.772922 4.293342 -0.853437
C -1.395600 4.492675 -0.956656
C -0.517476 3.437099 -0.720562
C 0.897053 0.752244 -1.071673
C 1.683189 -0.518269 -0.950041
C 0.745457 -1.629289 -0.472240
C -0.036574 -1.160326 0.742574
H -1.868981 1.043038 3.830299
H -2.278715 1.825029 2.297182
H -2.510798 0.075609 2.483262
H -0.000895 -0.115708 2.913075
H 0.020354 2.789350 1.972159
H 1.458614 1.736088 2.118048
H 0.490006 2.147870 3.549897
H -2.772638 0.991263 -0.009632
H -4.339534 2.874075 -0.434474
H -3.456529 5.116773 -1.034077
H -1.004057 5.472018 -1.212386
H 0.556229 3.593336 -0.780314
H 1.055805 1.399849 -1.926443
H 1.286462 -2.542310 -0.211117
H 0.074257 -1.872214 -1.301061
H 2.118668 -0.792177 -1.913434
S 3.127227 -0.371140 0.152784
O 3.834019 0.871834 -0.256934
O 2.597567 -0.337938 1.532931
O 3.952078 -1.577640 -0.134092
K 6.179319 -0.285916 -1.073559
H 0.132955 -1.700877 1.669915
S -2.001044 -2.208275 0.335824
O -2.068220 -2.971042 -0.933933
O -2.246648 -3.393589 1.457968
O -3.121996 -1.247225 0.502419
K -4.389404 -1.709634 -1.927404
H -2.451346 -2.995739 2.321597
43
TS_S3_conf_9_ts Eopt -3046.089278
C 2.839439 1.469401 0.794424
C 2.500955 1.171737 -0.663406
C 2.955379 2.271706 -1.619359
N 1.065467 0.779983 -0.824662
C -0.007971 1.413076 -0.112298
C 0.058571 2.882527 0.106920
C -0.077175 3.408958 1.395071
C -0.099912 4.789146 1.589928
C 0.006888 5.650954 0.498552
C 0.127981 5.129536 -0.790625
C 0.151472 3.750811 -0.986262
C -1.105387 0.715112 0.216317
C -1.121743 -0.734068 -0.154962
C -0.583473 -0.833751 -1.587276
C 0.833946 -0.353538 -1.521502
H 2.448849 2.439188 1.109616
H 2.446292 0.687768 1.450386
H 3.927616 1.496419 0.896493
H 3.042319 0.260239 -0.940904
H 2.615350 3.256034 -1.293786
H 2.593612 2.085436 -2.634453
H 4.048616 2.279429 -1.641448
H -0.149348 2.732178 2.241873
H -0.197134 5.189676 2.593800
H -0.008905 6.725420 0.650462
H 0.197119 5.795851 -1.644445
H 0.232117 3.342334 -1.990101
H -1.953430 1.203997 0.682986
H -1.159611 -0.166476 -2.240160
H -0.628996 -1.839311 -2.007067
H -0.476646 -1.260805 0.557605
S -2.642531 -1.666181 0.004352
O -3.564822 -1.242117 -1.077709
O -2.136008 -3.059164 -0.150404
O -3.196159 -1.371931 1.347644

K -5.635163 -0.381635 0.509391
H 1.501600 -0.536715 -2.362245
S 1.713664 -2.099326 -0.380676
O 1.753364 -1.800594 1.074771
O 0.738697 -3.404881 -0.578392
O 3.004822 -2.583429 -0.919889
K 4.447986 -2.372598 1.495081
H -0.192412 -3.264698 -0.281083
50
int10_conf_1 Eopt -1996.225081
C 3.213803 3.039747 -1.385565
C 2.029882 3.407388 -0.494212
C 0.784748 3.705913 -1.323380
N 1.739593 2.260500 0.338472
C 1.884072 2.184531 1.596445
C 1.632308 0.984088 2.417231
C 0.756174 -0.084687 1.784352
C -0.550338 0.422059 1.318743
C -1.509797 -0.328324 0.727033
C -2.757094 0.351073 0.304784
C -2.703090 1.368264 -0.640906
C -3.859121 2.019133 -1.033952
C -5.077375 1.664452 -0.479390
C -5.136747 0.656762 0.469170
C -3.983484 0.000126 0.859544
N -1.428961 -1.674301 0.569700
C -2.174264 -2.406769 -0.440614
C -2.126162 -3.885914 -0.078990
C -1.583755 -2.158500 -1.830382
H 2.972317 2.158214 -1.975271
H 4.096567 2.829385 -0.787024
H 3.436181 3.863449 -2.057374
H 2.283418 4.264572 0.140475
H 0.973294 4.548876 -1.981325
H -0.059037 3.951438 -0.683148
H 0.529454 2.840536 -1.930076
H 2.242221 3.065818 2.128731
H 2.621492 0.551364 2.619764
H 1.213072 1.292608 3.379821
H -0.736152 1.478078 1.430512
H -1.750260 1.630781 -1.077836
H -3.809864 2.802925 -1.775059
H -5.978865 2.172552 -0.785375
H -6.084230 0.381587 0.906584
H -4.025426 -0.778995 1.606183
H -0.509995 -2.073678 0.713687
H -3.211520 -2.057431 -0.426108
H -1.095220 -4.236707 -0.090985
H -2.538119 -4.047727 0.913580
H -2.702293 -4.467608 -0.792411
H -1.661427 -1.105353 -2.090266
H -2.115045 -2.742578 -2.576576
H -0.530214 -2.439107 -1.836051
H 0.680050 -0.947725 2.446913
S 1.707816 -0.793156 0.341081
O 3.141069 -0.638242 0.606832
O 1.329357 -0.009301 -0.848847
O 1.403658 -2.213584 0.179052
H 1.465786 1.388083 -0.223627
K 3.408585 -2.551752 -0.901473
50
int10_conf_10 Eopt -1996.220372
C 1.166412 4.234565 -0.178195
C 0.238980 3.271312 0.545145
C -0.736149 3.985893 1.479292
N -0.538198 2.436439 -0.341826
C -0.467734 2.308952 -1.603998
C -1.266896 1.293696 -2.319858
C -1.000846 -0.155827 -1.818825
C 0.416890 -0.497632 -1.540782
C 1.107547 -0.494732 -0.382669
C 2.547208 -0.845858 -0.450544
C 2.955098 -2.062159 -0.985521
C 4.300492 -2.377447 -1.057912
C 5.251017 -1.478250 -0.604732
C 4.852440 -0.260541 -0.078896
C 3.508079 0.054339 0.000124
N 0.630477 -0.095580 0.841085
C 1.130413 -0.692200 2.078699
C 0.783865 -2.179062 2.152205
C 0.510452 0.075614 3.240503
H 1.899048 3.703002 -0.781014
H 1.705601 4.826621 0.555487
H 0.603704 4.917612 -0.810531
H 0.831269 2.559700 1.138721
H -0.189870 4.502244 2.262438
H -1.410812 3.269444 1.940979
H -1.322627 4.717536 0.929513
H 0.231551 2.915297 -2.170455
H -2.336471 1.463099 -2.163454
H -1.056839 1.346772 -3.388098
H 0.971271 -0.776979 -2.425685

H	2.210329	-2.763532	-1.331593
H	4.607482	-3.327178	-1.468529
H	6.299865	-1.724969	-0.661839
H	5.590352	0.445744	0.270416
H	3.197695	1.007864	0.404058
H	-0.378469	-0.029697	0.878238
H	2.218832	-0.575512	2.102086
H	1.327715	-2.730048	1.388800
H	1.048892	-2.582691	3.125198
H	-0.282801	-2.318640	1.981264
H	-0.575475	0.003242	3.197416
H	0.791988	1.125654	3.206511
H	0.850179	-0.337826	4.185526
H	-1.385949	-0.825428	-2.588562
S	-2.214511	-0.480170	-0.479070
O	-3.518214	-0.515157	-1.132441
O	-2.163103	0.615098	0.512337
O	-1.952904	-1.767488	0.149461
H	-1.234221	1.792464	0.145749
K	-4.217677	-2.160563	0.377294

50

int10_conf_2 Eopt -1996.220930

C	-5.716740	1.576197	0.129575
C	-4.237821	1.661737	0.506201
C	-3.971623	1.015036	1.861157
N	-3.480480	0.941281	-0.490626
C	-2.669095	1.427000	-1.340261
C	-1.971304	0.567191	-2.322360
C	-0.775650	-0.165082	-1.699442
C	0.271641	0.727778	-1.168064
C	1.496550	0.312345	-0.782526
C	2.428695	1.314239	-0.206443
C	2.188047	1.791466	1.077128
C	3.036320	2.727350	1.643027
C	4.125922	3.197456	0.928089
C	4.362264	2.735134	-0.355764
C	3.517873	1.796421	-0.923295
N	1.936908	-0.970036	-0.913990
C	3.211367	-1.471360	-0.452820
C	3.561965	-2.708856	-1.270909
C	3.189693	-1.804343	1.043397
H	-5.903471	2.058340	-0.826755
H	-6.313956	2.072703	0.888393
H	-6.030239	0.537596	0.066426
H	-3.913182	2.708780	0.510864
H	-4.431278	1.605053	2.648236
H	-2.901656	0.944998	2.041425
H	-4.388915	0.011709	1.883412
H	-2.505603	2.501847	-1.358701
H	-2.663574	-0.198385	-2.681763
H	-1.614148	1.166316	-3.161593
H	0.008257	1.756766	-0.986784
H	1.323923	1.424685	1.620084
H	2.845403	3.094104	2.640620
H	4.786579	3.927775	1.369187
H	5.204776	3.107703	-0.917994
H	3.693381	1.444447	-1.929394
H	1.232538	-1.679342	-1.063771
H	3.966925	-0.694900	-0.620883
H	4.530006	-3.100828	-0.971703
H	2.808430	-3.481629	-1.120895
H	3.602181	-2.465097	-2.329173
H	4.176440	-2.134753	1.360341
H	2.469872	-2.608838	1.224546
H	2.917568	-0.919575	1.618004
H	-0.383788	-0.895085	-2.408949
S	-1.392286	-1.244155	-0.311295
O	-0.533795	-2.425567	-0.289001
O	-2.809257	-1.539349	-0.543996
O	-1.206741	-0.496411	0.939030
H	-3.501763	-0.124947	-0.442741
K	-0.095969	-2.234701	1.942851

50

int10_conf_3 Eopt -1996.223183

C	-5.365399	-0.185832	1.910948
C	-5.334721	-1.010132	0.626136
C	-6.201676	-0.373421	-0.457001
N	-3.974245	-1.020354	0.143835
C	-3.234290	-2.030820	-0.061199
C	-1.849297	-1.927104	-0.569095
C	-0.969016	-0.864716	0.099606
C	0.465561	-1.141135	-0.162802
C	1.519398	-0.605898	0.487109
C	2.873574	-1.013989	0.039363
C	3.288784	-0.711681	-1.253239
C	4.545148	-1.091710	-1.692582
C	5.394909	-1.786233	-0.846568
C	4.981544	-2.106494	0.435889
C	3.727677	-1.723458	0.878539
N	1.418118	0.213057	1.579574
C	2.507634	1.042096	2.061886
C	2.117429	1.567706	3.439412

C	2.809421	2.203723	1.109476
H	-4.991013	0.815976	1.716873
H	-4.755928	-0.647994	2.683528
H	-6.385463	-0.113124	2.275548
H	-5.661184	-2.038185	0.823335
H	-5.832239	0.621792	-0.690354
H	-7.226465	-0.293396	-0.107054
H	-6.191938	-0.973595	-1.363324
H	-3.632686	-3.024063	0.141391
H	-1.362599	-2.901272	-0.485434
H	-1.881681	-1.659484	-1.633161
H	0.673855	-1.790236	-1.000360
H	2.607506	-0.184040	-1.919585
H	4.859381	-0.853295	-2.698300
H	6.373583	-2.083597	-1.189589
H	5.636141	-2.658957	1.092504
H	3.397531	-1.988875	1.872148
H	0.527077	0.686336	1.650266
H	3.402192	0.417248	2.152602
H	1.249366	2.219296	3.360003
H	1.875055	0.742976	4.104409
H	2.936069	2.132627	3.875450
H	3.182617	1.822097	0.157793
H	3.565807	2.851575	1.544810
H	1.898046	2.779542	0.946865
H	-1.193740	-0.749290	1.160951
S	-1.307751	0.785261	-0.670463
O	-0.444130	1.747687	0.006485
O	-2.733468	1.098357	-0.499074
O	-0.904233	0.659689	-2.066165
H	-3.554107	-0.045939	-0.101979
K	0.752436	2.237448	-1.869001

50

int10_conf_4 Eopt -1996.223347

C	3.092557	4.202528	-0.165926
C	2.393394	3.018139	-0.814716
C	3.284283	2.315384	-1.839464
N	1.949523	2.002745	0.123642
C	2.123020	1.974202	1.381148
C	1.712789	0.874954	2.278164
C	0.687401	-0.100791	1.725765
C	-0.545354	0.549378	1.237698
C	-1.606184	-0.099037	0.701309
C	-2.754669	0.715328	0.239615
C	-2.575224	1.649631	-0.773926
C	-3.637773	2.423069	-1.206778
C	-4.885672	2.275604	-0.624930
C	-5.068769	1.352077	0.391085
C	-4.009994	0.572771	0.821472
N	-1.712259	-1.450951	0.633085
C	-2.562966	-2.137701	-0.325000
C	-2.710306	-3.583849	0.130824
C	-1.962484	-2.059857	-1.730256
H	2.445432	4.706882	0.547850
H	3.354711	4.920136	-0.938110
H	4.011369	3.894519	0.327838
H	1.484259	3.362686	-1.323355
H	4.203242	1.975124	-1.368097
H	3.536052	3.002624	-2.641450
H	2.768444	1.455968	-2.261172
H	2.627169	2.809204	1.861338
H	2.634008	0.322714	2.507833
H	1.352977	1.303073	3.218945
H	-0.583772	1.626036	1.280714
H	-1.600679	1.749861	-1.231216
H	-3.493040	3.140824	-2.000378
H	-5.713843	2.879436	-0.962398
H	-6.039216	1.238475	0.849672
H	-4.147225	-0.141434	1.619781
H	-0.854136	-1.961939	0.798149
H	-3.543168	-1.650649	-0.328062
H	-3.368734	-4.125814	-0.541549
H	-1.736550	-4.071378	0.137040
H	-3.127536	-3.625200	1.133481
H	-2.578078	-2.610863	-2.435789
H	-0.957090	-2.481412	-1.723014
H	-1.898850	-1.024360	-2.056414
H	0.505325	-0.900361	2.444907
S	1.507536	-1.022127	0.323386
O	2.953912	-1.048621	0.565431
O	1.216704	-0.267585	-0.908225
O	1.012840	-2.395943	0.257347
H	1.539458	1.143037	-0.365284
K	2.951900	-3.073658	-0.783971

50

int10_conf_5 Eopt -1996.221484

C	4.615280	-2.554020	-0.352002
C	3.336693	-1.987989	0.267714
C	3.644240	-1.186007	1.527176
N	2.720361	-1.105288	-0.697499
C	1.666112	-1.328230	-1.393515
C	1.225684	-0.366075	-2.416527

C	0.500003	0.901589	-1.877164
C	-0.912484	0.696493	-1.505686
C	-1.396973	-0.177355	-0.598295
C	-2.871606	-0.306535	-0.494164
C	-3.527747	-1.470152	-0.882524
C	-4.905969	-1.556123	-0.792979
C	-5.640541	-0.483689	-0.315900
C	-4.994585	0.681803	0.063199
C	-3.616932	0.771978	-0.029215
N	-0.591753	-0.980573	0.177860
C	-1.128031	-1.717900	1.314353
C	-1.413200	-0.785068	2.493226
C	-0.122807	-2.792335	1.715983
H	4.389614	-3.143542	-1.236830
H	5.117576	-3.191442	0.369324
H	5.289787	-1.748609	-0.629983
H	2.642844	-2.806551	0.487943
H	3.985820	-1.848021	2.317373
H	2.758152	-0.651401	1.863191
H	4.422412	-0.455277	1.322505
H	1.138790	-2.266926	-1.265725
H	2.110505	0.003224	-2.939933
H	0.554629	-0.850693	-3.126446
H	-1.636363	1.243753	-2.091642
H	-2.957160	-2.300947	-1.271580
H	-5.407303	-2.461121	-1.100120
H	-6.714752	-0.552827	-0.245566
H	-5.565328	1.522869	0.426879
H	-3.104368	1.686556	0.257635
H	0.270054	-0.502919	0.428873
H	-2.061626	-2.197614	1.005175
H	-0.500793	-0.251003	2.766778
H	-2.186334	-0.066328	2.227374
H	-1.756367	-1.358793	3.349542
H	-0.524411	-3.391418	2.528039
H	0.803609	-2.332544	2.052620
H	0.085894	-3.454588	0.879375
H	0.561751	1.666292	-2.648822
S	1.521031	1.677327	-0.550061
O	1.046990	3.044473	-0.433186
O	2.911631	1.503273	-0.940561
O	1.245488	0.980511	0.728629
H	3.125316	-0.138795	-0.776880
K	0.397137	2.912847	1.700432

50

int10_conf_6 Eopt -1996.223267

C	3.616908	4.040532	0.090491
C	2.212893	3.825060	0.644240
C	1.124900	4.369945	-0.276274
N	1.991899	2.374464	0.804184
C	1.707594	1.785029	1.899906
C	1.470929	0.316418	2.036706
C	0.841244	-0.431176	0.842976
C	-0.367692	0.223802	0.248028
C	-1.645729	-0.161916	0.457263
C	-2.748098	0.525164	-0.273994
C	-2.619957	0.827789	-1.633997
C	-3.638219	1.503154	-2.306194
C	-4.797248	1.876505	-1.626587
C	-4.936351	1.568065	-0.271643
C	-3.920751	0.892187	0.399389
N	-2.050804	-1.118020	1.386872
C	-2.938660	-2.210515	0.944685
C	-3.322651	-3.040570	2.162471
C	-2.287203	-3.061391	-0.145480
H	3.795669	5.111934	-0.022458
H	3.718948	3.570396	-0.892513
H	4.372696	3.627357	0.761910
H	2.123366	4.264966	1.641267
H	1.266595	5.445607	-0.401492
H	0.131104	4.192105	0.141602
H	1.185053	3.898877	-1.262735
H	1.651517	2.401891	2.796125
H	2.429205	-0.142629	2.317311
H	0.809012	0.188160	2.897650
H	-0.201220	1.084215	-0.393277
H	-1.725410	0.517536	-2.167065
H	-3.529278	1.728096	-3.362439
H	-5.591151	2.399739	-2.150108
H	-5.836738	1.855741	0.262093
H	-4.022649	0.649923	1.453658
H	-1.290508	-1.476769	1.955815
H	-3.841296	-1.744209	0.536732
H	-2.434372	-3.505595	2.606184
H	-3.804653	-2.418356	2.921110
H	-4.010688	-3.839532	1.875214
H	-2.012769	-2.446054	-1.008860
H	-2.970826	-3.845172	-0.484861
H	-1.377691	-3.538682	0.238490
H	0.607102	-1.440078	1.199374
S	2.061593	-0.774748	-0.468804
O	3.263079	-1.310854	0.218753

O	2.312015	0.538477	-1.139444
O	1.446385	-1.771808	-1.373796
H	2.100551	1.780417	-0.057526
K	3.326420	-3.760739	-1.054408

50

int10_conf_7 Eopt -1996.220822

C	5.297879	1.548350	-0.078990
C	5.077245	0.351183	-0.999842
C	5.774506	-0.894954	-0.460015
N	3.659136	0.076438	-1.031208
C	2.907460	0.043786	-2.054785
C	1.466339	-0.268848	-2.008478
C	0.690810	0.255950	-0.797324
C	-0.764983	0.151350	-1.020001
C	-1.781797	0.713680	-0.327023
C	-3.150952	0.234984	-0.647603
C	-3.433004	-1.127580	-0.630080
C	-4.709452	-1.584404	-0.908183
C	-5.717924	-0.684078	-1.213560
C	-5.445976	0.673549	-1.236476
C	-4.172330	1.131708	-0.949873
N	-1.770095	1.675837	0.627975
C	-0.666018	2.430427	1.177274
C	-1.065310	2.921600	2.564615
C	-0.307132	3.607751	0.263513
H	4.900998	1.332175	0.909567
H	4.806814	2.435341	-0.471647
H	6.360383	1.753783	0.008455
H	5.428669	0.578040	-2.013432
H	5.629220	-1.739763	-1.128320
H	5.373793	-1.147968	0.518081
H	6.839908	-0.707751	-0.365318
H	3.347084	0.247951	-3.030445
H	0.989828	0.082647	-2.926952
H	1.359326	-1.362414	-1.977148
H	-1.046676	-0.617007	-1.726600
H	-2.634709	-1.832479	-0.401243
H	-4.917489	-2.645103	-0.894978
H	-6.711768	-1.040504	-1.435471
H	-6.227169	1.377348	-1.479917
H	-3.962023	2.191006	-0.986788
H	-2.678103	1.954247	0.966758
H	0.188239	1.756413	1.290621
H	-1.937113	3.570790	2.507400
H	-1.292430	2.073001	3.205002
H	-0.250194	3.482977	3.011583
H	0.580319	4.116043	0.630469
H	-1.126051	4.323127	0.238547
H	-0.124385	3.267328	-0.753458
H	1.035197	1.240893	-0.490487
S	1.042084	-0.873475	0.636603
O	0.055629	-0.635439	1.681592
O	2.417247	-0.604501	1.088963
O	0.868728	-2.232421	0.114176
H	3.225933	-0.187328	-0.069298
K	-0.725433	-2.738997	1.704481

50

int10_conf_8 Eopt -1996.218308

C	5.160357	1.604584	0.321115
C	5.018294	0.667932	-0.876156
C	5.827403	-0.610413	-0.673880
N	3.627091	0.293305	-0.978145
C	2.848497	0.481022	-1.967602
C	1.435618	0.062079	-1.977693
C	0.664909	0.304977	-0.669880
C	-0.791395	0.213098	-0.879795
C	-1.821494	0.862811	-0.290534
C	-3.179730	0.314932	-0.537510
C	-4.225543	1.146634	-0.930266
C	-5.488389	0.626789	-1.150577
C	-5.724999	-0.725729	-0.969822
C	-4.692734	-1.559202	-0.569144
C	-3.427062	-1.042067	-0.356407
N	-1.844066	2.004363	0.447623
C	-0.746992	2.822263	0.916351
C	-0.124114	2.250725	2.194424
C	-1.279939	4.233448	1.158075
H	4.599067	2.522376	0.164367
H	6.205499	1.861048	0.465192
H	4.793209	1.113972	1.218980
H	5.328429	1.175186	-1.797498
H	5.475079	-1.134394	0.210896
H	6.876653	-0.364612	-0.541453
H	5.732169	-1.268751	-1.533603
H	3.241319	0.983233	-2.850279
H	0.912689	0.546978	-2.805039
H	1.387547	-1.022711	-2.142558
H	-1.062253	-0.637308	-1.491719
H	-4.040714	2.199462	-1.085235
H	-6.289219	1.278477	-1.464932
H	-6.710943	-1.129739	-1.139800
H	-4.875247	-2.615346	-0.423708

H -2.614372 -1.691348 -0.033352
H -2.710490 2.168496 0.938587
H -0.002812 2.874842 0.115188
H -0.821901 2.356829 3.021627
H 0.102783 1.192802 2.078093
H 0.787273 2.786510 2.445872
H -2.041379 4.224650 1.934769
H -1.714712 4.639160 0.247944
H -0.474664 4.888012 1.478346
H 1.003463 1.192874 -0.148481
S 1.052448 -1.101481 0.473617
O 0.101517 -1.076371 1.578383
O 2.442541 -0.941222 0.926583
O 0.856369 -2.329976 -0.300454
H 3.232641 -0.231293 -0.118260
K -0.503105 -3.237479 1.332188
50
int10_conf_9 Eopt -1996.214030
C 4.857876 1.116949 0.236928
C 5.024248 0.028289 -0.825054
C 5.961513 -1.075398 -0.348888
N 3.722671 -0.555348 -1.040087
C 2.926949 -0.365119 -2.011814
C 1.563781 -0.940874 -2.007531
C 0.691497 -0.224849 -0.962938
C -0.746276 -0.487325 -1.122705
C -1.769685 0.190208 -0.568862
C -3.102174 -0.451258 -0.527972
C -4.246819 0.242115 -0.917193
C -5.485463 -0.371594 -0.871256
C -5.597819 -1.676587 -0.420916
C -4.466414 -2.367803 -0.017540
C -3.224106 -1.761997 -0.071873
N -1.709327 1.427251 0.061673
C -1.030011 2.584190 -0.521201
C -0.091514 3.214102 0.504349
C -2.061257 3.605168 -1.001961
H 4.354459 0.713401 1.112225
H 4.266583 1.944701 -0.147106
H 5.832786 1.493058 0.531728
H 5.388353 0.467281 -1.761699
H 6.945647 -0.663964 -0.146507
H 6.061958 -1.849539 -1.105327
H 5.575188 -1.524077 0.562449
H 3.239492 0.260952 -2.845234
H 1.105217 -0.844873 -2.992660
H 1.601936 -1.996330 -1.725983
H -0.984829 -1.453734 -1.542683
H -4.169019 1.253583 -1.293321
H -6.363829 0.168625 -1.190708
H -6.565153 -2.153109 -0.385140
H -4.552984 -3.383307 0.337601
H -2.337455 -2.285847 0.253116
H -2.631453 1.698350 0.389663
H -0.457214 2.246192 -1.391202
H 0.622247 2.479475 0.870510
H 0.452740 4.041972 0.056505
H -0.669867 3.600710 1.343625
H -1.564276 4.469417 -1.434381
H -2.676212 3.948531 -0.170562
H -2.705932 3.172879 -1.764421
H 0.949419 0.827888 -0.890438
S 1.127421 -0.831892 0.773563
O 1.456637 0.407216 1.482681
O 2.267079 -1.746802 0.668676
O -0.062643 -1.427754 1.349634
H 3.310699 -1.133140 -0.223420
K -0.561852 0.331436 2.671364
50
int11_conf_0 Eopt -1996.225469
C 0.903245 1.067972 1.418207
C -0.332047 1.667501 0.755351
N -0.085344 2.156557 -0.535710
C -1.107691 2.909423 -1.265417
C -1.457770 4.207815 -0.542826
C -0.567721 3.183875 -2.664470
H -0.754053 2.448107 1.389918
H -2.037861 2.321701 -1.385808
H -1.905223 4.036997 0.441035
H -0.555658 4.813815 -0.413624
H -2.182523 4.774374 -1.133322
H -1.310836 3.720570 -3.258391
H 0.338517 3.794507 -2.600164
H -0.323505 2.250459 -3.180951
N -1.498521 0.572007 0.732709
C -2.389685 0.448559 1.976859
C -1.055065 -0.744901 0.241527
C 0.220138 -1.121202 0.327814
H 0.479516 -2.101408 -0.062834
C 1.336240 -0.295223 0.871911
C -3.180588 1.733730 2.176494
H -3.084144 -0.351585 1.704696

C -1.608351 0.031553 3.211034
H -3.934057 1.550844 2.945711
H -2.553516 2.559383 2.522700
H -3.706739 2.035214 1.265072
H -2.328403 -0.286852 3.967999
H -0.940788 -0.811527 3.011729
H -1.037713 0.864162 3.629679
H 0.478023 1.535998 -1.116259
H -2.134320 0.940526 0.015798
C -4.153202 -3.234281 -1.346309
C -3.277529 -3.731957 -0.381890
C -2.274982 -2.917011 0.139500
C -2.136096 -1.598368 -0.307958
C -3.017604 -1.103832 -1.277856
C -4.021504 -1.919384 -1.793697
H -4.936896 -3.868106 -1.748012
H -3.379707 -4.751933 -0.026376
H -1.605198 -3.293499 0.907206
H -2.908986 -0.093270 -1.666713
H -4.693034 -1.530036 -2.551487
H 1.844231 -0.855586 1.664981
S 2.643735 -0.188888 -0.403119
O 3.728083 0.618777 0.210405
O 3.066390 -1.595642 -0.626937
O 2.031234 0.439173 -1.595902
K 5.665402 -1.354853 0.201206
H 0.721678 0.961818 2.486915
H 1.711449 1.795700 1.308319
50
int11_conf_1 Eopt -1996.226098
C 2.824011 2.155804 1.095398
C 1.436974 1.530967 1.094270
C 1.283776 0.462721 2.163456
N 1.060248 1.003665 -0.281507
C 1.437849 -0.394561 -0.528351
C 2.851636 -0.761149 -0.319799
C 3.871115 -0.055316 -0.956963
C 5.188700 -0.453540 -0.816839
C 5.504000 -1.557596 -0.042495
C 4.494702 -2.273728 0.580838
C 3.175324 -1.884612 0.438178
C 0.575842 -1.283186 -1.024477
C -0.791241 -0.958800 -1.447504
S -1.959081 -1.383121 -0.063077
O -3.196632 -0.643246 -0.329317
O -2.231602 -2.810959 -0.144013
O -1.332954 -0.975711 1.177551
C -0.918430 0.521941 -1.731325
C -0.430196 1.291828 -0.510896
N -0.568722 2.706630 -0.579457
C -1.623571 3.325480 0.229379
C -1.412314 4.835323 0.192981
C -3.028996 2.950521 -0.240363
H 3.609369 1.413354 0.991117
H 2.924388 2.900584 0.308009
H 2.970105 2.662037 2.045997
H 0.717164 2.333653 1.270981
H 0.375871 -0.118388 2.016876
H 1.223574 0.944457 3.135314
H 2.136293 -0.209794 2.174243
H 3.639790 0.792346 -1.587322
H 5.969191 0.097671 -1.318163
H 6.532309 -1.863271 0.067550
H 4.735403 -3.139432 1.177714
H 2.384768 -2.437889 0.921997
H 0.914165 -2.298874 -1.158047
H -1.133040 -1.585187 -2.266511
H -0.343250 0.794665 -2.622351
H -1.967004 0.770122 -1.895194
H -0.938151 0.891902 0.371552
H -0.626094 3.046444 -1.533554
H -1.487847 2.965815 1.255631
H -2.106397 5.325357 0.868385
H -1.581721 5.219135 -0.810118
H -0.398724 5.086235 0.494162
H -3.767856 3.453656 0.376442
H -3.195507 1.876790 -0.164585
H -3.179004 3.261925 -1.272636
H 1.575310 1.595537 -0.945037
K -4.401267 -2.468118 0.549833
50
int11_conf_10 Eopt -1996.226955
C -0.520233 -0.869435 3.274767
C -0.146533 0.075212 2.139953
C -0.304336 1.520732 2.595018
N -1.024062 -0.156771 0.938058
C -0.601858 0.640543 -0.217611
C -1.192021 1.977303 -0.348510
C -0.403499 3.044387 -0.780043
C -0.951738 4.305053 -0.923790
C -2.292528 4.518457 -0.644465
C -3.085892 3.463076 -0.226075

C	-2.541955	2.200440	-0.076387
C	0.227674	0.106145	-1.115211
C	0.809649	-1.233776	-1.011139
S	2.578731	-0.970065	-0.498302
O	3.234905	-0.251155	-1.584955
O	2.543363	-0.084693	0.679196
O	3.130570	-2.263163	-0.203070
C	0.166829	-2.134426	0.033587
C	-1.195098	-1.613112	0.489605
N	-2.228407	-1.594742	-0.485173
C	-3.373166	-2.488426	-0.322898
C	-4.465782	-2.027545	-1.280961
C	-3.017977	-3.956041	-0.563496
H	-0.242603	-1.899180	3.071138
H	0.018814	-0.566542	4.167913
H	-1.585045	-0.817864	3.498287
H	0.886805	-0.088136	1.817349
H	0.312434	1.683084	3.474019
H	-1.337324	1.741556	2.861409
H	0.022011	2.219684	1.831114
H	0.646356	2.876630	-0.979722
H	-0.332006	5.124581	-1.253279
H	-2.719042	5.502431	-0.757486
H	-4.133190	3.622063	-0.020935
H	-3.190013	1.381644	0.209392
H	0.518549	0.688773	-1.974804
H	0.926081	-1.701529	-1.985260
H	0.037340	-3.141332	-0.364278
H	0.846830	-2.213174	0.882871
H	-1.539540	-2.166722	1.367245
H	-1.887582	-1.611918	-1.437228
H	-3.727340	-2.367341	0.708306
H	-4.149265	-2.167510	-2.311302
H	-4.688646	-0.975076	-1.128762
H	-5.372064	-2.602685	-1.118677
H	-2.262438	-4.299137	0.140327
H	-2.640991	-4.091491	-1.574883
H	-3.900574	-4.576950	-0.442971
H	-1.966100	0.173446	1.185024
K	4.685591	0.719730	-0.122400

50

int11_conf_2 Eopt -1996.226934

C	0.710307	0.865452	3.330798
C	0.509439	-0.161306	2.223724
C	1.154717	-1.483585	2.617945
N	1.127247	0.319877	0.935314
C	0.861758	-0.591081	-0.182017
C	1.833396	-1.668845	-0.398792
C	3.203275	-1.443916	-0.260590
C	4.106094	-2.465871	-0.490320
C	3.656471	-3.723675	-0.856591
C	2.297904	-3.954169	-1.004341
C	1.390753	-2.935874	-0.780261
C	-0.179828	-0.363530	-0.983786
C	-1.140922	0.729794	-0.809787
S	-2.668250	-0.074458	-0.113461
O	-3.702153	0.956814	-0.078433
O	-3.072978	-1.121327	-1.046432
O	-2.302962	-0.566876	1.195900
C	-0.717771	1.799732	0.185654
C	0.778315	1.744369	0.498019
N	1.664283	2.044192	-0.572924
C	2.460176	3.267831	-0.513898
C	3.547728	3.170302	-1.577844
C	1.621231	4.532276	-0.703721
H	0.105622	1.756263	3.189361
H	0.403143	0.420565	4.272796
H	1.758261	1.147867	3.424176
H	-0.556669	-0.323299	2.031041
H	0.970415	-2.257493	1.879917
H	0.723429	-1.819155	3.556344
H	2.229507	-1.375068	2.758573
H	3.580516	-0.458095	-0.018684
H	5.163796	-2.278186	-0.387777
H	4.363166	-4.519649	-1.030501
H	1.944230	-4.931904	-1.291946
H	0.330768	-3.116718	-0.874249
H	-0.358906	-1.030690	-1.811619
H	-1.482822	1.122933	-1.763594
H	-0.956111	2.792059	-0.197747
H	-1.294203	1.657780	1.100960
H	1.010756	2.393714	1.346464
H	1.248836	1.924972	-1.487205
H	2.930651	3.293221	0.476696
H	4.115932	2.251843	-1.459387
H	4.226691	4.013416	-1.497050
H	3.107098	3.177379	-2.571563
H	0.867593	4.625486	0.075444
H	1.124134	4.512991	-1.671323
H	2.258755	5.410456	-0.665962
H	2.146576	0.311134	1.073300
K	-5.302283	-0.637646	-0.605856

50
int11_conf_3 Eopt -1996.224615

C	2.885884	2.240176	0.957796
C	1.483274	1.651627	0.974097
C	1.290195	0.650285	2.100112
N	1.102683	1.059458	-0.372406
C	1.461653	-0.356093	-0.537798
C	2.868881	-0.729707	-0.299145
C	3.173256	-1.809859	0.526438
C	4.486177	-2.209160	0.697810
C	5.508086	-1.547421	0.036195
C	5.211869	-0.487835	-0.805087
C	3.900926	-0.079424	-0.974088
C	0.591454	-1.260665	-0.988392
C	-0.763905	-0.938504	-1.448439
S	-1.961317	-1.271819	-0.064798
O	-1.373509	-0.766397	1.159851
O	-3.198874	-0.567767	-0.413497
O	-2.217275	-2.704436	-0.044654
C	-0.857318	0.526386	-1.817537
C	-0.379413	1.351100	-0.626505
N	-0.513931	2.766898	-0.744853
C	-1.693733	3.409447	-0.141226
C	-3.015983	2.920744	-0.735308
C	-1.705577	3.262183	1.377067
H	3.654599	1.474859	0.911505
H	3.016491	2.933053	0.128561
H	3.031136	2.800147	1.877971
H	0.780811	2.479309	1.092501
H	2.126558	-0.039464	2.163629
H	0.371639	0.081695	1.973417
H	1.226382	1.188695	3.041522
H	2.373317	-2.322093	1.039081
H	4.711914	-3.040656	1.346904
H	6.531383	-1.861090	0.168602
H	6.002032	0.020227	-1.335935
H	3.684571	0.732308	-1.655110
H	0.915867	-2.287108	-1.059056
H	-1.104417	-1.604192	-2.236548
H	-0.257293	0.737890	-2.709173
H	-1.896186	0.781534	-2.022009
H	-0.901636	0.993351	0.263325
H	-0.427989	3.069836	-1.709375
H	-1.571753	4.472397	-0.379389
H	-3.202541	1.880406	-0.472966
H	-3.012759	3.022282	-1.819285
H	-3.832926	3.521928	-0.346943
H	-2.553499	3.813368	1.773417
H	-0.804022	3.679246	1.818595
H	-1.812228	2.223310	1.680755
H	1.632191	1.604077	-1.064211
K	-4.409417	-2.317977	0.578857

50
int11_conf_5 Eopt -1996.226006

C	2.365747	2.346460	1.340802
C	1.060590	1.578288	1.196195
C	0.990837	0.378599	2.125897
N	0.797754	1.182778	-0.246149
C	1.334758	-0.126756	-0.641133
C	2.765441	-0.381638	-0.385302
C	3.158545	-1.555030	0.254877
C	4.500406	-1.835489	0.436965
C	5.465321	-0.957470	-0.030409
C	5.083614	0.199939	-0.688193
C	3.742222	0.488726	-0.866496
C	0.597938	-1.021589	-1.300959
C	-0.773892	-0.781472	-1.761062
S	-1.965089	-1.490931	-0.518225
O	-2.158956	-2.894608	-0.853910
O	-1.320173	-1.416942	0.805368
O	-3.161839	-0.687796	-0.572711
C	-1.046942	0.702796	-1.860518
C	-0.709089	1.337733	-0.518979
N	-1.002650	2.723749	-0.400473
C	-2.147224	3.114708	0.430579
C	-2.078383	4.624824	0.632144
C	-3.486270	2.693741	-0.172207
H	2.418893	2.745210	2.350383
H	3.234580	1.713318	1.187747
H	2.407601	3.188962	0.652971
H	0.247383	2.270405	1.427875
H	0.814182	0.727518	3.139348
H	1.922976	-0.178747	2.119049
H	0.178148	-0.288396	1.846691
H	2.400765	-2.235232	0.617168
H	4.794465	-2.742515	0.941672
H	6.511600	-1.178376	0.109431
H	5.830960	0.878500	-1.069007
H	3.461445	1.382300	-1.406639
H	1.050685	-1.969949	-1.546175
H	-1.013469	-1.328502	-2.668259
H	-0.461146	1.153202	-2.668191

H	-2.107190	0.863662	-2.055581
H	-1.205720	0.762325	0.267340
H	-1.066182	3.184818	-1.301804
H	-2.021995	2.614479	1.397852
H	-1.109723	4.915260	1.030513
H	-2.849409	4.942966	1.326657
H	-2.234636	5.141202	-0.311835
H	-3.551676	1.611254	-0.266429
H	-3.618332	3.145660	-1.153809
H	-4.298420	3.028586	0.466216
H	1.264844	1.903186	-0.810564
K	-2.628364	-3.399229	1.316428

50

int11_conf_6 Eopt -1996.223778

C	0.787265	1.068934	2.186968
C	1.675930	1.437823	1.011345
C	1.984612	2.925721	0.955124
N	1.108324	0.970682	-0.321180
C	1.484717	-0.432750	-0.580226
C	2.888455	-0.795245	-0.305147
C	3.942801	-0.049627	-0.834211
C	5.250988	-0.438095	-0.611604
C	5.522966	-1.568261	0.142187
C	4.481383	-2.314000	0.669633
C	3.170502	-1.933774	0.446575
C	0.638756	-1.319518	-1.101868
C	-0.737135	-0.999585	-1.493357
S	-1.828816	-1.489040	-0.068004
O	-3.065875	-0.710895	-0.202220
O	-2.141549	-2.902324	-0.228190
O	-1.111069	-1.177660	1.148266
C	-0.888318	0.485716	-1.727255
C	-0.386338	1.252215	-0.504549
N	-0.562714	2.664154	-0.594944
C	-1.654116	3.269831	0.172019
C	-1.545266	4.784760	0.026295
C	-3.039338	2.784138	-0.257377
H	-0.022227	1.781279	2.314316
H	0.367359	0.072391	2.071903
H	1.387582	1.081499	3.092678
H	2.620538	0.897022	1.120583
H	2.698425	3.151890	0.164991
H	2.430584	3.226588	1.898960
H	1.083243	3.506536	0.795762
H	3.746070	0.823114	-1.442182
H	6.058808	0.140784	-1.031539

H	6.544720	-1.867318	0.315134
H	4.690131	-3.194214	1.257219
H	2.353102	-2.500785	0.864561
H	0.982757	-2.331594	-1.245512
H	-1.095367	-1.607215	-2.319101
H	-0.337252	0.787967	-2.624591
H	-1.942752	0.724783	-1.863645
H	-0.879493	0.848150	0.380935
H	-0.606536	2.982281	-1.557613
H	-1.494743	2.996303	1.220614
H	-1.728719	5.081359	-1.003828
H	-0.558316	5.135692	0.316005
H	-2.282164	5.271692	0.657463
H	-3.800078	3.283401	0.335701
H	-3.146229	1.710295	-0.112483
H	-3.216162	3.022308	-1.304884
H	1.584795	1.556157	-1.018482
K	-4.258712	-2.569592	0.595148

50

int11_conf_7 Eopt -1996.226918

C	-0.761226	-1.120503	3.204458
C	-0.496472	-0.035435	2.168635
C	-1.106880	1.281438	2.631273
N	-1.092173	-0.413126	0.836705
C	-0.762938	0.555033	-0.213457
C	-1.675163	1.692604	-0.376596
C	-3.056741	1.539389	-0.261087
C	-3.899076	2.621268	-0.439792
C	-3.376989	3.870192	-0.732318
C	-2.005888	4.031780	-0.855054
C	-1.159388	2.953314	-0.681109
C	0.286733	0.335219	-1.006777
C	1.206253	-0.799152	-0.878999
S	2.739486	-0.087041	-0.099201
O	3.740421	-1.150754	-0.105093
O	3.197315	1.003396	-0.954189
O	2.356022	0.333854	1.229997
C	0.724540	-1.908678	0.043304
C	-0.775383	-1.819999	0.318799
N	-1.618720	-2.027519	-0.803996
C	-2.607306	-3.103717	-0.745166
C	-3.766975	-2.685503	0.158674
C	-3.104196	-3.381786	-2.157091
H	-0.190154	-2.025212	3.019124
H	-0.456499	-0.747764	4.178080
H	-1.821453	-1.364380	3.261735

H	0.579380	0.100843	2.014332
H	-0.692962	1.538882	3.601572
H	-2.188735	1.203998	2.734129
H	-0.873116	2.094465	1.951758
H	-3.492026	0.566299	-0.073311
H	-4.966605	2.487825	-0.354963
H	-4.036391	4.712840	-0.867263
H	-1.594953	5.002550	-1.083532
H	-0.090103	3.080949	-0.753774
H	0.510695	1.044686	-1.787002
H	1.558812	-1.147141	-1.846210
H	0.940776	-2.886939	-0.387511
H	1.280134	-1.836948	0.979268
H	-1.051067	-2.512579	1.120068
H	-1.137524	-1.995224	-1.690280
H	-2.153304	-4.022250	-0.337033
H	-4.240806	-1.789722	-0.235463
H	-3.434387	-2.500018	1.178410
H	-4.509861	-3.476578	0.192341
H	-3.532574	-2.479952	-2.586937
H	-3.866218	-4.154919	-2.140013
H	-2.290112	-3.721179	-2.793267
H	-2.114554	-0.381400	0.945318
K	5.404242	0.423226	-0.490170

50

int11_conf_8 Eopt -1996.220923

C	1.044808	0.895256	2.727191
C	0.306971	0.025967	1.720602
C	0.412113	-1.441031	2.102583
N	0.887893	0.313626	0.347432
C	0.842982	-0.832970	-0.550716
C	1.968315	-1.773522	-0.450162
C	3.272680	-1.316679	-0.256541
C	4.327228	-2.209795	-0.198933
C	4.098079	-3.569224	-0.329469
C	2.807096	-4.032661	-0.527044
C	1.750392	-3.144441	-0.589773
C	-0.158723	-0.959276	-1.421463
C	-1.147187	0.114093	-1.598444
S	-2.486237	-0.003307	-0.310709
O	-3.751580	0.003563	-1.033709
O	-2.332145	-1.302187	0.356263
O	-2.328169	1.104831	0.608162
C	-0.445528	1.457196	-1.510996
C	0.362227	1.646200	-0.222590
N	1.519504	2.473557	-0.352760
C	1.466133	3.844140	0.159765
C	2.885235	4.401595	0.145671
C	0.513983	4.740053	-0.631095
H	2.081984	0.575984	2.823033
H	1.024708	1.945548	2.452731
H	0.573779	0.799964	3.701051
H	-0.751448	0.289406	1.667613
H	0.033421	-1.551137	3.115109
H	1.439219	-1.797868	2.084269
H	-0.207262	-2.051625	1.452228
H	3.489133	-0.257252	-0.188448
H	5.331319	-1.841416	-0.055738
H	4.922165	-4.263086	-0.280172
H	2.623109	-5.090642	-0.629349
H	0.742990	-3.508227	-0.719907
H	-0.204445	-1.826074	-2.060471
H	-1.702707	0.017845	-2.526676
H	0.221693	1.546954	-2.375151
H	-1.185132	2.255666	-1.563607
H	-0.307180	2.046838	0.541748
H	1.892049	2.478615	-1.296615
H	1.116402	3.783472	1.196344
H	2.905361	5.381529	0.611899
H	3.245140	4.500701	-0.875599
H	3.558606	3.745049	0.690078
H	0.543840	5.751073	-0.235997
H	-0.509414	4.378566	-0.561225
H	0.805351	4.773928	-1.679058
H	1.886823	0.510657	0.516497
K	-4.694062	-1.451100	0.439306

50

int11_conf_9 Eopt -1996.223366

C	2.494559	2.410123	1.118253
C	1.176065	1.659015	1.011550
C	1.033259	0.589093	2.081275
N	0.958643	1.086227	-0.377481
C	1.507348	-0.265830	-0.566357
C	2.934125	-0.467686	-0.249772
C	3.326963	-1.536183	0.553419
C	4.668216	-1.773459	0.792843
C	5.633013	-0.958986	0.222163
C	5.252134	0.090763	-0.597026
C	3.911543	0.337113	-0.833815
C	0.787065	-1.253296	-1.099702
C	-0.562444	-1.082178	-1.647391
S	-1.809082	-1.669591	-0.401348

O	-1.472066	-1.077507	0.906376
O	-3.103118	-1.118550	-0.807116
O	-1.766174	-3.104669	-0.380442
C	-0.821819	0.373240	-1.967859
C	-0.530767	1.192288	-0.715485
N	-0.850522	2.583320	-0.777794
C	-2.139797	3.034656	-0.230608
C	-3.347982	2.413941	-0.934223
C	-2.227086	2.813662	1.276667
H	3.351990	1.744508	1.099002
H	2.595469	3.149639	0.326127
H	2.508931	2.941583	2.066208
H	0.371947	2.391491	1.110725
H	1.943694	0.005070	2.178079
H	0.205764	-0.082512	1.863003
H	0.839539	1.069171	3.036700
H	2.571520	-2.169871	0.993005
H	4.961154	-2.598291	1.423295
H	6.678677	-1.147033	0.407158
H	5.999360	0.717226	-1.058920
H	3.632792	1.144028	-1.497672
H	1.240437	-2.228895	-1.181215
H	-0.755398	-1.747520	-2.484582
H	-0.194090	0.697244	-2.804962
H	-1.868684	0.500473	-2.238644
H	-1.050524	0.717952	0.119027
H	-0.743887	2.946644	-1.719121
H	-2.146745	4.116007	-0.412693
H	-3.404468	1.341484	-0.743903
H	-3.295145	2.581865	-2.008095
H	-4.260300	2.879447	-0.570346
H	-3.164985	3.229872	1.635539
H	-1.419000	3.325126	1.793381
H	-2.205653	1.752931	1.529450
H	1.454769	1.722182	-1.013776
K	-3.868118	-1.632897	1.348820

50

int12_conf_0 Eopt -1996.248945

C	0.915036	1.031478	1.410156
C	-0.329984	1.638856	0.770749
N	-0.089548	2.149829	-0.518161
C	-1.119382	2.915018	-1.223991
C	-1.442652	4.213022	-0.488219
C	-0.605699	3.191458	-2.632520
H	-0.732879	2.418239	1.419551
H	-2.058432	2.338054	-1.329176
H	-1.866136	4.040971	0.506015
H	-0.533286	4.812038	-0.379066
H	-2.178082	4.787067	-1.057999
H	-1.353814	3.742131	-3.207075
H	0.309764	3.789613	-2.583409
H	-0.384974	2.258355	-3.160011
N	-1.488007	0.566346	0.744260
C	-2.369546	0.433097	1.997343
C	-1.062085	-0.751045	0.234017
C	0.210448	-1.139199	0.302690
H	0.457076	-2.117701	-0.100294
C	1.337712	-0.325348	0.841593
C	-3.150477	1.720216	2.221951
H	-3.071173	-0.358588	1.719762
C	-1.579246	-0.005883	3.217845
H	-3.895923	1.530546	2.997358
H	-2.515348	2.537868	2.572191
H	-3.686070	2.036547	1.321145
H	-2.294686	-0.328174	3.977539
H	-0.920644	-0.851724	3.001398
H	-0.998068	0.816719	3.641632
H	0.148928	1.373086	-1.101084
C	-4.203860	-3.193455	-1.340026
C	-3.324862	-3.707591	-0.387367
C	-2.307817	-2.907777	0.129414
C	-2.157830	-1.588045	-0.311202
C	-3.042247	-1.077101	-1.269896
C	-4.060799	-1.877470	-1.780761
H	-4.998940	-3.815375	-1.737953
H	-3.435547	-4.728569	-0.037340
H	-1.635148	-3.296884	0.888238
H	-2.924828	-0.065666	-1.653952
H	-4.734905	-1.475568	-2.529639
H	1.854404	-0.897455	1.620439
S	2.627570	-0.208864	-0.451114
O	3.715463	0.602889	0.150781
O	3.053946	-1.613510	-0.681501
O	1.996081	0.418251	-1.634127
K	5.626131	-1.384644	0.247028
H	0.749782	0.913159	2.480262
H	1.721660	1.759999	1.295859
H	0.718349	2.735722	-0.454662

50

int12_conf_1 Eopt -1996.252029

C	-0.740651	0.759330	-1.773741
C	0.082239	1.520306	-0.730824

N -0.652738 1.628032 0.587549
C -1.883521 2.463368 0.670788
C -2.407679 2.347542 2.096212
C -1.601782 3.912670 0.301068
H 0.239275 2.542656 -1.087300
H -2.619147 2.024762 -0.008286
H -3.361880 2.858510 2.183113
H -2.553381 1.301581 2.356852
H -1.715576 2.799851 2.803992
H -1.343992 4.026608 -0.748117
H -2.492347 4.507170 0.482009
H -0.799896 4.320766 0.913481
N 1.349751 0.927427 -0.459041
C 2.544443 1.766438 -0.552141
C 1.461144 -0.459207 -0.494871
C 0.492904 -1.252165 -0.981395
H 0.595142 -2.320878 -0.910616
C -0.779173 -0.732423 -1.506605
C 2.494516 2.919670 0.449019
H 3.384930 1.121380 -0.287159
C 2.770555 2.269249 -1.979211
H 1.721468 3.643813 0.200429
H 2.333732 2.547283 1.458655
H 3.446868 3.441784 0.437865
H 2.751779 1.435108 -2.676085
H 2.019531 2.995819 -2.281544
H 3.743314 2.747195 -2.046565
H 0.023835 1.964466 1.278686
C 4.925055 -2.272890 1.188691
C 4.186282 -1.365606 1.930419
C 3.071700 -0.762242 1.378394
C 2.680859 -1.064290 0.075966
C 3.421579 -1.984154 -0.658733
C 4.539780 -2.581493 -0.105153
H 5.796441 -2.740431 1.620088
H 4.477543 -1.129850 2.942696
H 2.486622 -0.067951 1.963942
H 3.120999 -2.219659 -1.668448
H 5.111052 -3.290040 -0.684815
H -1.136565 -1.287044 -2.371252
S -2.077365 -1.097624 -0.239394
O -3.255380 -0.264338 -0.526257
O -2.452382 -2.498075 -0.303318
O -1.516683 -0.724806 1.076491
K -4.612951 -2.065476 0.258734
H -0.266687 0.929135 -2.743949
H -1.758616 1.144859 -1.820178
H -0.953580 0.614069 0.884701
50
int12_conf_10 Eopt -1996.243767
C 0.897360 0.886691 1.602291
C -0.090249 1.579749 0.653426
N 0.404491 1.622924 -0.771081
C 1.128362 2.836037 -1.256346
C 0.201304 4.044049 -1.298581
C 2.378383 3.093475 -0.430150
H -0.226633 2.616154 0.977918
H 1.433258 2.590111 -2.279178
H -0.696137 3.830109 -1.874963
H -0.089674 4.369209 -0.303184
H 0.713156 4.873617 -1.777102
H 2.988524 3.841301 -0.928680
H 2.131123 3.472618 0.557858
H 2.969247 2.184698 -0.333019
N -1.366879 0.933021 0.618176
C -2.501134 1.596483 1.280222
C -1.326355 -0.458598 0.464923
C -0.236611 -1.181741 0.768001
H -0.211002 -2.234303 0.543159
C 0.995816 -0.602897 1.332849
C -3.206964 0.687534 2.281469
H -2.073807 2.435941 1.842867
C -3.490729 2.169521 0.266113
H -3.793151 -0.073185 1.775259
H -2.485750 0.199138 2.932511
H -3.877770 1.282761 2.894666
H -2.981289 2.792936 -0.465265
H -4.018368 1.373702 -0.250339
H -4.221884 2.783390 0.784244
H -0.426687 1.486254 -1.355084
C -4.624710 -2.410643 -1.389781
C -4.069533 -1.281189 -1.967220
C -3.009699 -0.637259 -1.354190
C -2.492777 -1.116530 -0.154147
C -3.043505 -2.264382 0.408809
C -4.106618 -2.902715 -0.202803
H -5.454323 -2.908924 -1.866595
H -4.460371 -0.902145 -2.899371
H -2.567136 0.230437 -1.822615
H -2.642492 -2.642029 1.337276
H -4.532046 -3.786441 0.247148
H 1.346005 -1.148711 2.207128

S 2.357328 -0.897851 0.119173
O 3.523698 -0.106328 0.527318
O 2.712744 -2.304792 0.097121
O 1.863504 -0.422780 -1.190919
K 4.922337 -1.834934 -0.287636
H 0.521060 1.045186 2.616319
H 1.892182 1.323816 1.537305
H 1.045442 0.747782 -0.967079
50
int12_conf_2 Eopt -1996.249469
C -0.698006 1.135921 -2.007038
C 0.009963 1.679148 -0.758244
N -0.926968 1.561156 0.412181
C -0.790567 2.531565 1.526762
C -1.401597 3.863376 1.117439
C -1.497775 1.948284 2.740805
H 0.228715 2.741385 -0.893017
H 0.275382 2.650038 1.741225
H -2.452159 3.733407 0.867773
H -1.331222 4.570587 1.938503
H -0.893556 4.299552 0.261604
H -1.062586 0.990873 3.019313
H -1.409487 2.623640 3.586725
H -2.554419 1.800709 2.526158
N 1.221047 0.984131 -0.445400
C 2.499595 1.682932 -0.622167
C 1.207960 -0.401511 -0.632659
C 0.222352 -1.044571 -1.280436
H 0.218260 -2.122255 -1.305583
C -0.942067 -0.353389 -1.863596
C 2.651039 2.849091 0.349624
H 3.270840 0.946012 -0.384861
C 2.706693 2.131251 -2.070311
H 3.652511 3.258741 0.254672
H 1.946686 3.651652 0.143742
H 2.525751 2.512405 1.375596
H 2.019782 2.926946 -2.350431
H 3.719236 2.503905 -2.193980
H 2.570317 1.290885 -2.747095
H -0.868924 0.579670 0.769512
C 4.308839 -2.646680 1.227456
C 3.956356 -2.911123 -0.085638
C 2.957340 -2.176899 -0.699183
C 2.302689 -1.164133 -0.004997
C 2.663075 -0.904077 1.315842
C 3.658279 -1.643357 1.927161
H 5.088873 -3.219475 1.704231
H 4.463260 -3.689442 -0.635032
H 2.691022 -2.374002 -1.726684
H 2.151039 -0.126185 1.863645
H 3.927852 -1.436316 2.951723
H -1.281764 -0.805191 -2.792173
S -2.381610 -0.641041 -0.719200
O -3.155467 -1.780961 -1.173409
O -1.806821 -0.961675 0.615770
O -3.134144 0.612388 -0.622240
K -3.439300 -2.621146 0.900409
H -0.051305 1.310840 -2.869852
H -1.640372 1.659025 -2.171529
H -1.923365 1.551030 0.051899
50
int12_conf_3 Eopt -1996.249993
C 1.157358 -0.651857 -2.088002
C 0.780449 -1.501143 -0.872269
N 1.746501 -1.185265 0.267366
C 2.628704 -2.269644 0.767357
C 3.692007 -2.566613 -0.279592
C 3.257479 -1.790741 2.067871
H 0.947764 -2.557831 -1.109046
H 2.025881 -3.165972 0.952527
H 3.257644 -2.946772 -1.200303
H 4.255221 -1.663445 -0.501901
H 4.382423 -3.315860 0.096549
H 3.929727 -2.548136 2.460205
H 3.829351 -0.882615 1.891263
H 2.500017 -1.588155 2.822354
N -0.574959 -1.326019 -0.484547
C -1.218663 -2.312719 0.383798
C -1.246474 -0.145255 -0.787443
C -0.698820 0.844773 -1.503039
H -1.269479 1.734963 -1.712885
C 0.713092 0.789020 -1.917173
C -0.842339 -2.123979 1.858103
H -2.289893 -2.120801 0.295448
C -0.979554 -3.749147 -0.076494
H -1.569051 -2.637069 2.482333
H 0.135339 -2.539432 2.087686
H -0.863139 -1.067282 2.119531
H -1.673799 -4.402056 0.444658
H -1.163447 -3.846006 -1.143606
H 0.025546 -4.099508 0.146549
H 1.203534 -0.749146 1.023174

C	-5.249021	0.490361	0.523109
C	-5.007880	-0.359494	-0.543706
C	-3.711503	-0.590623	-0.968762
C	-2.645109	0.028842	-0.324341
C	-2.894198	0.891984	0.736164
C	-4.191598	1.118142	1.161295
H	-6.260767	0.668478	0.852893
H	-5.831185	-0.840591	-1.048874
H	-3.521872	-1.245550	-1.806529
H	-2.056466	1.380652	1.224386
H	-4.379213	1.784738	1.989786
H	0.935701	1.392193	-2.792526
S	1.671448	1.622276	-0.567996
O	1.696673	3.048206	-0.835482
O	0.951444	1.387635	0.705030
O	2.984827	0.967280	-0.502606
K	0.889368	3.641591	1.186330
H	0.652151	-1.063134	-2.965962
H	2.233169	-0.703925	-2.255508
H	2.395751	-0.381812	-0.041046

50

int12_conf_4 Eopt -1996.248452

C	0.744767	0.453605	2.049234
C	0.029259	1.361346	1.036851
N	0.943559	1.547955	-0.141558
C	0.729149	2.701852	-1.058017
C	2.091215	3.195597	-1.523231
C	-0.127790	2.252661	-2.229784
H	-0.120970	2.351233	1.479725
H	0.221499	3.495542	-0.500388
H	1.971877	4.029034	-2.209710
H	2.693910	3.534131	-0.683310
H	2.621095	2.398086	-2.041537
H	-1.083769	1.875770	-1.880686
H	-0.310320	3.083531	-2.905480
H	0.376597	1.461408	-2.780144
N	-1.236027	0.848252	0.604799
C	-2.451417	1.537005	1.063749
C	-1.324670	-0.539357	0.441366
C	-0.355326	-1.387933	0.824790
H	-0.439058	-2.431992	0.577506
C	0.880697	-0.950819	1.495144
C	-2.513627	2.991429	0.608155
H	-3.280800	1.003019	0.593652
C	-2.617650	1.428981	2.581207
H	-1.787056	3.617739	1.121025
H	-2.365368	3.074664	-0.464089
H	-3.499547	3.384279	0.840322
H	-1.860136	2.004493	3.109474
H	-3.592248	1.811263	2.870174
H	-2.554952	0.388965	2.893100
H	0.963460	0.640842	-0.713522
C	-4.792250	-2.154563	-1.435657
C	-4.327415	-2.689046	-0.245661
C	-3.207194	-2.155620	0.365101
C	-2.538993	-1.075291	-0.204910
C	-3.014103	-0.544482	-1.401505
C	-4.130852	-1.083310	-2.012859
H	-5.665377	-2.571895	-1.912570
H	-4.838886	-3.523473	0.208885
H	-2.850612	-2.567207	1.297328
H	-2.495457	0.280791	-1.864741
H	-4.484333	-0.666953	-2.943819
H	1.223625	-1.657313	2.246956
S	2.245053	-1.002399	0.233056
O	3.174331	0.117985	0.528379
O	2.967748	-2.256884	0.308572
O	1.634303	-0.759395	-1.081492
K	4.886523	-1.338711	-0.471157
H	0.141520	0.415985	2.959300
H	1.725505	0.857766	2.302402
H	1.918435	1.544734	0.213033

50

int12_conf_5 Eopt -1996.245584

C	-0.850220	1.190254	-2.084005
C	-0.163526	1.782385	-0.847439
N	-1.078063	1.646220	0.339930
C	-0.841808	2.570302	1.477610
C	-1.441472	3.933696	1.167757
C	-1.469031	1.958069	2.721007
H	-0.011236	2.855010	-1.008762
H	0.242138	2.651705	1.610434
H	-1.283784	4.606097	2.005729
H	-0.988150	4.387830	0.290798
H	-2.512385	3.845161	0.999740
H	-1.047551	0.975667	2.922589
H	-1.288691	2.592836	3.583685
H	-2.544247	1.855114	2.589000
N	1.085561	1.165807	-0.524098
C	2.298258	1.995246	-0.603374
C	1.151347	-0.218365	-0.709470
C	0.214782	-0.918099	-1.369527

H 0.288437 -1.993487 -1.398848
C -1.000153 -0.309197 -1.937569
C 3.108281 1.692298 -1.862512
H 1.940680 3.029315 -0.671005
C 3.175407 1.906862 0.644248
H 3.910820 2.417219 -1.963828
H 3.547527 0.701254 -1.806264
H 2.480264 1.748755 -2.748856
H 2.578345 1.977616 1.549698
H 3.740147 0.981646 0.670527
H 3.880528 2.733988 0.635698
H -1.038630 0.639743 0.661514
C 4.161467 -2.519173 1.233301
C 3.246267 -1.791424 1.976672
C 2.287608 -1.024725 1.339478
C 2.237637 -0.976904 -0.050569
C 3.138797 -1.733904 -0.790379
C 4.103028 -2.492806 -0.149901
H 4.913259 -3.111393 1.731405
H 3.280230 -1.821510 3.055204
H 1.567162 -0.463841 1.917480
H 3.083602 -1.724127 -1.869126
H 4.806774 -3.067301 -0.732236
H -1.331003 -0.784577 -2.857050
S -2.389247 -0.670875 -0.753172
O -3.067623 -1.891985 -1.143856
O -1.753611 -0.894665 0.581074
O -3.238317 0.514139 -0.674372
K -3.241033 -2.674175 0.963876
H -0.224565 1.405037 -2.953940
H -1.824304 1.655041 -2.237838
H -2.073865 1.679322 0.015783
50
int12_conf_6 Eopt -1996.234301
C 1.222089 -0.646880 -2.102099
C 0.909355 -1.503583 -0.877201
N 1.935639 -1.207220 0.212474
C 2.841235 -2.296510 0.658364
C 3.875875 -2.556085 -0.426299
C 3.498532 -1.846675 1.954697
H 1.056551 -2.563157 -1.132353
H 2.251913 -3.203487 0.837693
H 4.431039 -1.644885 -0.635569
H 4.577731 -3.315399 -0.093763
H 3.415682 -2.908288 -1.345616
H 2.756755 -1.676834 2.732372
H 4.188862 -2.607029 2.307816
H 4.055212 -0.926907 1.791154
N -0.421435 -1.332969 -0.392698
C -0.805649 -2.232934 0.701724
C -1.159121 -0.205236 -0.738276
C -0.658349 0.788836 -1.484774
H -1.282484 1.625145 -1.756527
C 0.755656 0.782999 -1.898824
C -1.934334 -3.192680 0.321978
H 0.076865 -2.854881 0.884824
C -1.119602 -1.480309 1.996623
H -1.980399 -3.995001 1.053659
H -2.896106 -2.692524 0.305945
H -1.747366 -3.630385 -0.655349
H -0.991501 -2.145593 2.846618
H -0.461752 -0.619489 2.117175
H -2.144908 -1.123812 2.000283
H 1.431283 -0.777021 0.999464
C -5.270943 0.313415 0.247743
C -4.906789 -0.615355 -0.712226
C -3.570879 -0.813029 -1.018668
C -2.586833 -0.087313 -0.356244
C -2.961642 0.868735 0.581930
C -4.296821 1.061199 0.890146
H -6.312138 0.464752 0.486225
H -5.663844 -1.183647 -1.230312
H -3.290260 -1.518269 -1.786357
H -2.189868 1.453547 1.076209
H -4.579297 1.796843 1.628686
H 0.959895 1.408754 -2.762594
S 1.700925 1.602669 -0.537378
O 1.636237 3.036225 -0.747383
O 1.021038 1.279856 0.740041
O 3.044794 1.012225 -0.532890
K 0.651398 3.486541 1.234967
H 0.689816 -1.060884 -2.962614
H 2.291409 -0.680149 -2.311017
H 2.558884 -0.385075 -0.110520
50
int12_conf_7 Eopt -1996.245102
C -0.881748 0.637793 -2.076017
C -0.091446 1.473258 -1.060717
N -0.901264 1.621236 0.199536
C -0.399221 2.602722 1.195957
C -0.730448 4.022969 0.761997
C -1.024699 2.270865 2.542620

H	0.045554	2.480636	-1.470717
H	0.685154	2.465972	1.247749
H	-0.253828	4.283779	-0.179073
H	-1.805932	4.149515	0.657047
H	-0.380929	4.725757	1.512473
H	-0.838547	1.232786	2.807543
H	-0.604366	2.905553	3.317354
H	-2.100463	2.432829	2.514452
N	1.183196	0.925973	-0.714393
C	2.375600	1.687735	-1.122359
C	1.243661	-0.466707	-0.596586
C	0.268972	-1.284569	-1.025524
H	0.344622	-2.339463	-0.824233
C	-0.985924	-0.804156	-1.625649
C	2.984543	1.124192	-2.405283
H	2.017558	2.702880	-1.329534
C	3.433977	1.804609	-0.027628
H	2.239107	1.063795	-3.195441
H	3.790905	1.768872	-2.742896
H	3.389184	0.132154	-2.229651
H	2.985993	2.058832	0.929130
H	3.996702	0.884795	0.084792
H	4.128434	2.595846	-0.298432
H	-0.988140	0.646650	0.665205
C	4.420914	-2.290317	1.570053
C	4.245267	-2.587279	0.229205
C	3.224662	-1.986953	-0.487280
C	2.383911	-1.067306	0.128474
C	2.552080	-0.788824	1.481822
C	3.566722	-1.397600	2.196881
H	5.215279	-2.760897	2.128389
H	4.900401	-3.292104	-0.259404
H	3.077882	-2.229014	-1.529821
H	1.874356	-0.106187	1.972469
H	3.689402	-1.178882	3.246645
H	-1.368387	-1.456469	-2.406110
S	-2.274682	-0.930941	-0.290796
O	-3.235139	0.180242	-0.470799
O	-2.977449	-2.196630	-0.371078
O	-1.577792	-0.735276	0.992891
K	-4.879416	-1.333734	0.507775
H	-0.348111	0.675294	-3.028902
H	-1.877489	1.057754	-2.221842
H	-1.881949	1.815573	-0.046072

50

int12_conf_8 Eopt -1996.245058

C	-0.829647	0.845801	-1.730525
C	0.123014	1.610157	-0.799962
N	-0.407585	1.714431	0.606046
C	-1.144932	2.946617	0.997839
C	-2.437506	3.073324	0.206521
C	-1.419238	2.846233	2.491790
H	0.240841	2.636026	-1.169783
H	-0.498548	3.809875	0.793805
H	-2.248389	3.332473	-0.831443
H	-3.001481	2.143146	0.245789
H	-3.048215	3.862471	0.635350
H	-2.018409	1.962249	2.698963
H	-0.494296	2.785433	3.060969
H	-1.963146	3.721704	2.832987
N	1.413085	0.997814	-0.704877
C	2.545545	1.647434	-1.383707
C	1.399661	-0.384265	-0.474826
C	0.332698	-1.147282	-0.759413
H	0.324523	-2.186189	-0.476859
C	-0.898050	-0.629826	-1.384068
C	3.275812	0.702915	-2.333105
H	2.111907	2.453226	-1.988932
C	3.514283	2.281834	-0.386271
H	3.861107	-0.028905	-1.785029
H	2.569383	0.179007	-2.972868
H	3.950103	1.277463	-2.962042
H	4.052255	1.519790	0.168805
H	4.238062	2.887671	-0.924014
H	2.985820	2.925605	0.313626
H	0.402384	1.606910	1.225012
C	4.699205	-2.153864	1.552827
C	4.107679	-1.006985	2.054683
C	3.047430	-0.422419	1.385473
C	2.566284	-0.979546	0.204285
C	3.153635	-2.144085	-0.282359
C	4.217150	-2.722911	0.385244
H	5.529277	-2.605676	2.072959
H	4.470485	-0.567570	2.971493
H	2.577428	0.460688	1.794612
H	2.780673	-2.581854	-1.195923
H	4.671199	-3.620291	-0.005897
H	-1.211774	-1.229632	-2.236459
S	-2.285583	-0.900835	-0.194703
O	-3.466348	-0.181010	-0.684302
O	-2.589605	-2.316486	-0.094485
O	-1.854621	-0.329245	1.099447

K	-4.823945	-1.892293	0.216916
H	-0.439094	0.961776	-2.744741
H	-1.836103	1.260112	-1.704668
H	-1.053750	0.850833	0.838120

50

int12_conf_9 Eopt -1996.252591

C	-1.352572	-0.337979	2.055961
C	-1.123212	-1.257079	0.854096
N	-2.125413	-0.864024	-0.235023
C	-3.444471	-1.562727	-0.221095
C	-4.436622	-0.710484	-1.000807
C	-3.364043	-2.968078	-0.797017
H	-1.400831	-2.281922	1.130599
H	-3.759537	-1.612172	0.825659
H	-5.431257	-1.140612	-0.931499
H	-4.469315	0.299147	-0.600053
H	-4.159964	-0.662307	-2.051472
H	-3.105710	-2.940633	-1.853300
H	-2.647375	-3.590810	-0.268797
H	-4.336219	-3.445083	-0.709282
N	0.219995	-1.254937	0.384403
C	0.718785	-2.399794	-0.380390
C	1.128049	-0.282450	0.791567
C	0.795704	0.746920	1.578917
H	1.534724	1.482302	1.854899
C	-0.620260	0.982717	1.901137
C	0.193247	-2.410403	-1.818338
H	1.799972	-2.255774	-0.433243
C	0.480613	-3.738864	0.319135
H	-0.818348	-2.801879	-1.878913
H	0.235498	-1.407494	-2.240512
H	0.824975	-3.054002	-2.424909
H	0.753857	-3.674652	1.369548
H	-0.548942	-4.078717	0.239049
H	1.107505	-4.494117	-0.146007
H	-1.669387	-0.940100	-1.147749
C	5.173785	-0.361337	-0.537380
C	4.186219	0.205926	-1.327954
C	2.870611	0.205858	-0.899017
C	2.534049	-0.367886	0.323309
C	3.529861	-0.925346	1.117790
C	4.844787	-0.923644	0.684507
H	6.199129	-0.361392	-0.872883
H	4.442380	0.643164	-2.281806
H	2.085394	0.638006	-1.509922
H	3.270741	-1.358906	2.072238
H	5.613321	-1.359715	1.303809
H	-0.780955	1.643549	2.747274
S	-1.269552	1.940459	0.456053
O	-0.926788	3.334956	0.664609
O	-0.581409	1.455268	-0.758494
O	-2.698602	1.631143	0.345891
K	0.296971	3.506431	-1.234420
H	-0.974408	-0.833558	2.954142
H	-2.420975	-0.164161	2.186345
H	-2.368745	0.171987	-0.104316

37

int13_conf_0 Eopt -1821.787526

C	2.098890	1.700767	1.941704
C	2.294249	1.976898	0.452478
C	2.458561	3.458314	0.150477
N	1.160188	1.359282	-0.307359
C	1.147779	-0.077358	-0.479707
C	2.422953	-0.820915	-0.290814
C	3.505607	-0.618267	-1.156340
C	4.668404	-1.368734	-1.008393
C	4.757774	-2.329322	0.001498
C	3.679807	-2.540023	0.859137
C	2.513915	-1.787895	0.713811
C	0.030516	-0.710041	-0.853501
C	-1.254687	0.011738	-1.080556
C	-0.990896	1.470200	-1.441547
C	0.139805	2.078367	-0.688788
H	2.992358	2.021858	2.482313
H	1.243147	2.259596	2.329007
H	1.942911	0.636618	2.136942
H	3.189501	1.461570	0.107853
H	2.541352	3.646910	-0.923110
H	1.646409	4.063139	0.564119
H	3.383901	3.790496	0.625536
H	3.430798	0.121053	-1.950004
H	5.501967	-1.208809	-1.684382
H	5.665186	-2.913539	0.115450
H	3.743373	-3.287412	1.643208
H	1.670312	-1.945678	1.380308
H	0.066542	-1.785911	-0.985144
H	-1.882782	2.088102	-1.332988
H	-0.694516	1.513839	-2.500183
H	0.260333	3.150237	-0.699145
H	-1.826759	-0.458360	-1.883608
S	-2.349708	-0.218983	0.375493
O	-3.384838	0.848054	0.258099

O -2.939926 -1.570255 0.189368
O -1.511652 -0.101188 1.585367
K -5.554987 -0.802117 -0.119051
37
int13_conf_1 Eopt -1821.787526
C -1.796837 3.447097 -0.663037
C -1.805866 1.911215 -0.558840
C -0.973180 1.314068 -1.709766
N -1.224121 1.504902 0.728497
C 0.152378 1.960541 0.853078
C 0.937564 1.308643 1.979132
C 1.219938 -0.100131 1.529444
C -0.155538 -0.748548 1.229286
C -1.237001 0.008424 0.796695
C -2.510459 -0.730721 0.347551
C -3.619233 -0.004482 -0.107239
C -4.778343 -0.677653 -0.516323
C -4.829755 -2.076351 -0.468891
C -3.719246 -2.804282 -0.013646
C -2.559629 -2.129826 0.393999
H -0.789570 3.801412 -0.598068
H -2.373891 3.863737 0.136346
H -2.220146 3.743527 -1.599963
H -2.813304 1.554932 -0.624096
H 0.034130 1.669542 -1.642463
H -1.394615 1.611749 -2.647297
H -0.979914 0.246431 -1.638876
H 0.580810 2.690268 0.197721
H 1.833077 1.855599 2.189341
H 0.337768 1.268789 2.864017
H -0.271775 -1.805761 1.349016
H -3.580751 1.064115 -0.142160
H -5.624982 -0.123829 -0.865044
H -5.717556 -2.587016 -0.779957
H -3.756104 -3.873551 0.023112
H -1.711448 -2.682702 0.741045
H 1.731028 -0.641927 2.297502
S 2.226409 -0.088789 0.061183
O 2.463202 -1.470367 -0.380490
O 3.512206 0.559654 0.353870
O 1.440784 0.755123 -1.147158
K 4.967645 -1.241697 -0.763002
37
int13_conf_2 Eopt -1821.787526
C -1.775288 3.449660 -0.670499
C -1.793801 1.913615 -0.561760
C -0.964056 1.307748 -1.708951
N -1.217385 1.506699 0.727839
C 0.160866 1.953928 0.855065
C 0.938801 1.300562 1.985561
C 1.215427 -0.110398 1.539121
C -0.162722 -0.751664 1.236161
C -1.238704 0.010251 0.798667
C -2.514912 -0.723325 0.346163
C -2.571473 -2.122899 0.389918
C -3.732822 -2.790453 -0.021861
C -4.837610 -2.058433 -0.477395
C -4.781049 -0.658858 -0.521151
C -3.619700 0.008695 -0.109371
H -2.351800 3.870620 0.126575
H -2.194856 3.745850 -1.609187
H -0.766346 3.799761 -0.604439
H -2.802744 1.563514 -0.627820
H -0.976918 0.240496 -1.633398
H 0.044887 1.657849 -1.642890
H -1.383624 1.603938 -2.647638
H 0.595104 2.678694 0.198520
H 1.836172 1.843303 2.197825
H 0.335873 1.265546 2.868824
H -0.285409 -1.807647 1.357599
H -1.727942 -2.681813 0.737728
H -3.776008 -3.859059 0.011549
H -5.724326 -2.568125 -0.791796
H -5.624578 -0.099945 -0.868962
H -3.576514 1.077301 -0.142779
H 1.722079 -0.653332 2.309463
S 2.225280 -0.107415 0.073315
O 3.514157 0.534342 0.367711
O 2.455821 -1.490882 -0.365541
O 1.446116 0.738236 -1.137750
K 4.963561 -1.217333 -0.784234
36
int14_conf_0 Eopt -1821.348048
C 2.955717 3.037210 -0.047294
C 2.176647 1.915488 0.629064
C 1.447979 2.364150 1.892259
N 1.189744 1.297099 -0.312434
C 0.247449 2.051527 -0.839922
C -0.836595 1.543085 -1.597357
C -1.234347 0.125929 -1.245378
C -0.001020 -0.680590 -0.971604
C 1.126254 -0.122484 -0.502484

C 2.343014 -0.930830 -0.238566
C 2.235964 -2.099023 0.523122
C 3.351580 -2.910025 0.728329
C 4.583273 -2.557374 0.179974
C 4.698345 -1.388642 -0.575506
C 3.585723 -0.579498 -0.783605
H 2.322394 3.901434 -0.267127
H 3.419919 2.697308 -0.977013
H 3.744519 3.373026 0.629612
H 2.867191 1.116260 0.891570
H 0.761197 3.188881 1.676024
H 2.179088 2.709946 2.627092
H 0.872208 1.539012 2.317704
H 0.364714 3.123163 -0.703516
H -1.661282 2.250714 -1.687813
H -0.017170 -1.751216 -1.143037
H 1.278646 -2.362291 0.963385
H 3.257406 -3.812317 1.323689
H 5.452249 -3.186592 0.342855
H 5.654074 -1.108639 -1.006277
H 3.682658 0.325092 -1.377935
H -1.837328 -0.329141 -2.037722
S -2.329415 0.099606 0.220180
O -3.556751 0.839944 -0.188410
O -1.572257 0.758306 1.306183
O -2.629541 -1.337518 0.477056
K -5.337345 -1.213023 0.075150
36
int14_conf_1 Eopt -1821.348040
C 2.667309 3.012602 0.674155
C 1.727598 1.835801 0.943928
C 0.692511 2.177578 2.018563
N 1.055651 1.394857 -0.268739
C 0.101877 2.208068 -0.837953
C -0.882107 1.751642 -1.617806
C -1.101900 0.304553 -1.734222
C 0.127779 -0.444531 -1.460900
C 1.086104 0.069719 -0.669320
C 2.254044 -0.754502 -0.304678
C 2.047315 -1.991374 0.299820
C 3.122825 -2.798878 0.622447
C 4.414417 -2.384474 0.340950
C 4.627329 -1.159008 -0.268430
C 3.554725 -0.347450 -0.589391
H 2.114955 3.896871 0.366797
H 3.382758 2.764461 -0.105894
H 3.215490 3.253229 1.581342
H 2.324915 0.988691 1.294365
H 1.165810 2.223256 2.996237
H -0.083665 1.414576 2.026360
H 0.223473 3.137183 1.814816
H 0.234331 3.263981 -0.659754
H -1.578373 2.426596 -2.085038
H 0.209005 -1.457685 -1.817815
H 1.036027 -2.297748 0.521957
H 2.954420 -3.755132 1.095875
H 5.253650 -3.015938 0.593117
H 5.632690 -0.836214 -0.497119
H 3.719963 0.600552 -1.080181
H -1.661731 -0.017874 -2.606999
S -2.275515 -0.175435 -0.319242
O -2.578922 -1.614074 -0.433944
O -3.538344 0.572433 -0.453914
O -1.627389 0.115944 0.955495
K -4.410882 -1.106250 0.974048
36
int14_conf_10 Eopt -1821.343906
C 1.049861 0.637796 2.305106
C 1.354068 1.813501 1.377872
C 2.837508 2.190661 1.405167
N 0.843868 1.568669 0.033651
C -0.103566 2.442141 -0.458200
C -1.018097 2.107807 -1.372017
C -1.174295 0.697739 -1.750134
C 0.079301 -0.035595 -1.552232
C 0.973859 0.356523 -0.628946
C 2.182171 -0.473521 -0.433162
C 3.440201 -0.017539 -0.812625
C 4.548121 -0.837111 -0.683679
C 4.410102 -2.119569 -0.180052
C 3.156337 -2.588073 0.178113
C 2.046314 -1.773544 0.044318
H 1.156220 0.950495 3.341175
H 1.729014 -0.190700 2.122896
H 0.027659 0.306011 2.132100
H 0.787307 2.676702 1.744525
H 3.050699 2.950680 0.656757
H 3.468539 1.326618 1.220572
H 3.085736 2.592818 2.384452
H -0.021897 3.453819 -0.086243
H -1.704557 2.838282 -1.764020
H 0.240365 -0.948836 -2.101043

H	3.544034	0.974383	-1.227075
H	5.521339	-0.475290	-0.982394
H	5.276325	-2.756318	-0.076710
H	3.044126	-3.592920	0.558291
H	1.061588	-2.128771	0.312570
H	-1.677142	0.516274	-2.695249
S	-2.399263	-0.059706	-0.520318
O	-1.878140	0.101001	0.847961
O	-2.524744	-1.498628	-0.804822
O	-3.699683	0.598201	-0.645032
K	-3.893632	-1.396343	1.196153

36

int14_conf_4 Eopt -1821.348455

C	2.540631	3.018318	0.835543
C	1.615506	1.813891	1.020263
C	0.546063	2.083949	2.081641
N	0.984286	1.422531	-0.230272
C	0.036890	2.250199	-0.789194
C	-0.913122	1.819915	-1.624261
C	-1.102439	0.378121	-1.824154
C	0.129185	-0.365553	-1.547732
C	1.051064	0.120873	-0.697542
C	2.217173	-0.705864	-0.332315
C	3.521468	-0.271035	-0.551977
C	4.592705	-1.085068	-0.232601
C	4.374596	-2.340542	0.310441
C	3.079583	-2.782213	0.527138
C	2.005631	-1.972175	0.205670
H	1.980557	3.908759	0.561813
H	3.277959	2.823125	0.060901
H	3.063391	3.219200	1.766908
H	2.218532	0.960576	1.345347
H	0.058672	3.040102	1.907183
H	0.993766	2.097800	3.072261
H	-0.210414	1.302723	2.035548
H	0.145422	3.297277	-0.552711
H	-1.605187	2.507400	-2.079094
H	0.239135	-1.357576	-1.952756
H	3.691392	0.701000	-0.991355
H	5.601118	-0.740859	-0.410966
H	5.212693	-2.973920	0.561142
H	2.907290	-3.761874	0.948201
H	0.990925	-2.300484	0.376943
H	-1.628335	0.093165	-2.730479
S	-2.321373	-0.198707	-0.485803
O	-3.570938	0.563249	-0.579789
O	-1.713946	-0.002472	0.839369
O	-2.595132	-1.632073	-0.678098
K	-3.990233	-1.200072	1.290973

36

int14_conf_7 Eopt -1821.343249

C	1.182506	0.866341	2.252796
C	1.483515	1.927636	1.196165
C	2.972896	2.276993	1.145218
N	0.934196	1.545925	-0.101041
C	-0.022619	2.369477	-0.655422
C	-0.973477	1.945167	-1.491886
C	-1.158947	0.502881	-1.695214
C	0.094579	-0.220267	-1.461064
C	1.027879	0.263889	-0.623158
C	2.238510	-0.550671	-0.383163
C	2.115299	-1.790723	0.236251
C	3.226209	-2.595170	0.415342
C	4.467507	-2.177929	-0.037020
C	4.591951	-0.957708	-0.679536
C	3.483375	-0.147635	-0.854702
H	1.839486	0.007662	2.143840
H	0.149450	0.540650	2.144064
H	1.323720	1.285511	3.246083
H	0.940815	2.836904	1.478488
H	3.584782	1.386337	1.039578
H	3.253337	2.777368	2.069062
H	3.178342	2.948430	0.314491
H	0.082917	3.416203	-0.406870
H	-1.666625	2.635262	-1.940889
H	0.226627	-1.191991	-1.907436
H	1.140611	-2.106428	0.578536
H	3.124222	-3.552315	0.905519
H	5.334563	-2.806825	0.102119
H	5.554937	-0.637173	-1.050132
H	3.575964	0.794091	-1.375639
H	-1.701318	0.218628	-2.591835
S	-2.332733	-0.081575	-0.326436
O	-3.648178	0.556627	-0.509704
O	-1.761948	0.258480	0.972804
O	-2.508145	-1.540068	-0.452831
K	-4.430863	-1.186453	0.890078

43

int7_conf_1 Eopt -771.437034

C	-5.740430	0.400990	1.423837
C	-5.558163	0.193427	-0.080842
C	-6.836644	-0.310528	-0.739993

N	-4.468567	-0.739776	-0.368923
C	-3.167089	-0.366876	-0.254831
C	-2.091488	-1.193674	-0.155319
C	-0.752125	-0.672331	-0.116760
C	0.374247	-1.421512	-0.030302
C	1.729715	-0.878459	0.016430
C	1.882325	0.616656	-0.021595
C	2.044519	1.277845	-1.242209
C	2.190635	2.664801	-1.275828
C	2.174176	3.398889	-0.089401
C	2.009596	2.742353	1.130924
C	1.862309	1.355772	1.164512
N	2.707630	-1.684753	0.087051
C	4.074312	-1.180624	0.134262
C	4.776496	-1.798947	1.342291
C	4.778208	-1.561996	-1.168167
H	-6.024773	-0.542208	1.903090
H	-6.525142	1.137794	1.619432
H	-4.811764	0.754278	1.881909
H	-5.272778	1.147490	-0.540352
H	-6.703046	-0.420537	-1.819013
H	-7.122116	-1.283068	-0.322903
H	-7.657591	0.386884	-0.556291
H	-4.677423	-1.720945	-0.217499
H	-3.009675	0.711878	-0.286705
H	-2.236201	-2.271733	-0.111592
H	-0.655195	0.413742	-0.158994
H	0.306617	-2.507412	0.009241
H	2.055968	0.703967	-2.165315
H	2.317209	3.170927	-2.227794
H	2.288021	4.477988	-0.115615
H	1.995476	3.309129	2.056644
H	1.733046	0.842014	2.113725
H	4.104537	-0.086583	0.237177
H	4.268146	-1.520289	2.270266
H	5.814003	-1.455546	1.398160
H	4.773739	-2.891123	1.262134
H	4.754272	-2.648312	-1.304680
H	4.285043	-1.095604	-2.026468
H	5.822582	-1.235760	-1.147509

43

int7_conf_10 Eopt -771.427552

C	-5.672794	1.159615	-0.656244
C	-5.634885	-0.224942	-0.010271
C	-5.710678	-0.121590	1.517137
N	-4.464332	-0.967848	-0.438754
C	-3.189969	-0.515845	-0.281609
C	-2.088953	-1.292545	-0.345612
C	-0.766822	-0.771052	-0.252749
C	0.348784	-1.526624	-0.303730
C	1.699435	-1.018040	-0.205937
C	1.826216	0.462355	-0.055856
C	1.963069	1.031978	1.204484
C	2.066639	2.404991	1.343652
C	2.025648	3.221881	0.226507
C	1.868344	2.661899	-1.030796
C	1.762550	1.290167	-1.171629
N	2.635498	-1.878684	-0.255746
C	4.047760	-1.633561	-0.159398
C	4.594668	-0.383532	-0.858780
C	4.468850	-1.644485	1.315275
H	-4.929459	1.819819	-0.217914
H	-6.651857	1.606556	-0.506766
H	-5.484133	1.079443	-1.723671
H	-6.501036	-0.793747	-0.369312
H	-6.613499	0.404657	1.815175
H	-5.722056	-1.112511	1.966147
H	-4.847629	0.418901	1.899255
H	-4.560981	-1.972606	-0.428408
H	-3.089141	0.551692	-0.152178
H	-2.195765	-2.361368	-0.476772
H	-0.681297	0.302058	-0.128992
H	0.275948	-2.598398	-0.422964
H	1.980507	0.394227	2.077508
H	2.177504	2.838710	2.327391
H	2.109867	4.293527	0.335722
H	1.825140	3.297335	-1.903771
H	1.635087	0.850504	-2.150839
H	4.499400	-2.513578	-0.642406
H	4.341963	0.522454	-0.314153
H	4.194206	-0.309352	-1.867596
H	5.678731	-0.455558	-0.921982
H	4.093239	-0.756785	1.818927
H	4.066455	-2.525300	1.809897
H	5.553477	-1.658964	1.392521

43

int7_conf_2 Eopt -771.435290

C	5.779964	-0.171125	1.454269
C	5.661683	-0.395730	-0.057432
C	5.749078	0.927480	-0.817107
N	4.446969	-1.112874	-0.397698
C	3.199678	-0.587219	-0.256452

C 2.061656 -1.311142 -0.235599
C 0.768588 -0.719162 -0.161843
C -0.384039 -1.418474 -0.128707
C -1.696948 -0.826091 -0.048478
C -1.768348 0.665861 -0.004973
C -1.792442 1.332587 1.214244
C -1.862751 2.713871 1.253336
C -1.910553 3.440672 0.075502
C -1.887589 2.781979 -1.142455
C -1.816995 1.400800 -1.183748
N -2.705252 -1.603179 -0.020674
C -4.052055 -1.110720 0.054010
C -4.809529 -1.624650 -1.172335
C -4.683157 -1.653417 1.338142
H 5.755135 -1.121057 1.983970
H 4.953467 0.441078 1.808070
H 6.713899 0.332527 1.688479
H 6.490760 -1.034188 -0.385420
H 6.751820 1.336030 -0.726701
H 5.529412 0.769298 -1.869790
H 5.049439 1.657377 -0.419143
H 4.496183 -2.117064 -0.306403
H 3.152621 0.491033 -0.214888
H 2.114243 -2.391164 -0.278922
H 0.735425 0.363483 -0.126670
H -0.370390 -2.498597 -0.162096
H -1.755013 0.761291 2.131442
H -1.881417 3.224762 2.205400
H -1.966593 4.519277 0.106845
H -1.925575 3.346312 -2.063392
H -1.799036 0.882607 -2.132352
H -4.102809 -0.006574 0.069169
H -5.854840 -1.329706 -1.120693
H -4.743439 -2.708626 -1.213099
H -4.372688 -1.216627 -2.081136
H -4.157799 -1.265182 2.207957
H -5.728318 -1.359128 1.397978
H -4.613941 -2.737900 1.347751
43
int7_conf_3 Eopt -771.431740
C -5.802341 0.475870 1.455939
C -5.458546 0.452608 -0.037291
C -6.687334 0.114556 -0.877607
N -4.412475 -0.503452 -0.319793
C -3.091720 -0.213319 -0.207429
C -2.106414 -1.135023 -0.154527
C -0.706240 -0.854061 -0.106811
C -0.118728 0.360621 -0.121441
C 1.307347 0.569928 -0.063624
C 2.177834 -0.642097 0.009134
C 2.621412 -1.257889 -1.155230
C 3.434442 -2.375276 -1.087517
C 3.811795 -2.886562 0.142968
C 3.373734 -2.276888 1.306639
C 2.560767 -1.159290 1.241126
N 1.735015 1.769850 -0.078986
C 3.134994 2.086208 -0.028944
C 3.486381 2.871912 -1.294265
C 3.382085 2.940736 1.216381
H -6.189161 -0.491733 1.769854
H -6.555448 1.232270 1.659483
H -4.914346 0.700419 2.042488
H -5.080817 1.438515 -0.330811
H -7.085609 -0.857060 -0.592419
H -7.462287 0.861274 -0.729515
H -6.426121 0.086552 -1.932406
H -4.668977 -1.473801 -0.204319
H -2.862924 0.842422 -0.212296
H -2.386006 -2.181225 -0.147856
H -0.066658 -1.727182 -0.049067
H -0.699584 1.268466 -0.179141
H 2.324241 -0.855501 -2.113633
H 3.775040 -2.848516 -1.997382
H 4.447205 -3.758816 0.195004
H 3.666979 -2.673010 2.268339
H 2.216518 -0.680143 2.147089
H 3.774731 1.186392 0.022180
H 4.525612 3.190373 -1.264046
H 2.844788 3.745837 -1.370413
H 3.333364 2.252157 -2.175029
H 2.739077 3.816544 1.190833
H 3.154623 2.370099 2.114208
H 4.421179 3.258666 1.255300
43
int7_conf_4 Eopt -771.429289
C -5.824071 0.144899 1.550928
C -5.524988 0.301225 0.055861
C -6.755108 -0.028282 -0.785967
N -4.429316 -0.547325 -0.353535
C -3.125546 -0.187642 -0.223393
C -2.079185 -1.037640 -0.273980
C -0.724510 -0.603075 -0.209206

C 0.337824 -1.432660 -0.247701
C 1.720083 -1.012348 -0.177800
C 1.946452 0.460059 -0.074251
C 2.133071 1.056904 1.166973
C 2.329058 2.423593 1.263141
C 2.331743 3.207377 0.121749
C 2.125516 2.621482 -1.116420
C 1.927341 1.256319 -1.214380
N 2.596645 -1.934424 -0.209977
C 4.023095 -1.780424 -0.133017
C 4.644072 -0.592591 -0.877390
C 4.457303 -1.771624 1.337933
H -4.934554 0.363810 2.137426
H -6.139481 -0.873731 1.768302
H -6.616667 0.824524 1.851967
H -5.220539 1.335847 -0.139936
H -6.525372 0.071598 -1.843574
H -7.081144 -1.048895 -0.596200
H -7.571371 0.645297 -0.540551
H -4.618956 -1.539309 -0.328771
H -2.958267 0.877162 -0.125114
H -2.258645 -2.100151 -0.370646
H -0.566906 0.465047 -0.118808
H 0.192527 -2.500205 -0.333599
H 2.116350 0.445996 2.058964
H 2.477817 2.878302 2.232215
H 2.488029 4.273777 0.197439
H 2.116232 3.231905 -2.008207
H 1.761343 0.796831 -2.178572
H 4.410910 -2.703575 -0.590483
H 4.238441 -0.523967 -1.884483
H 5.720275 -0.738715 -0.947631
H 4.458002 0.345109 -0.360306
H 4.000607 -2.605901 1.864896
H 5.539044 -1.857836 1.407468
H 4.148133 -0.844113 1.814299
43
int7_conf_6 Eopt -771.434081
C 4.912208 -1.019307 1.635937
C 4.804986 -0.629329 0.157622
C 5.412399 0.749222 -0.090248
N 3.430646 -0.626534 -0.285342
C 2.795088 -1.736488 -0.737830
C 1.460457 -1.896051 -0.886707
C 0.463501 -0.946344 -0.514666
C -0.859246 -1.131527 -0.697846
C -1.882536 -0.186149 -0.287469
C -1.498423 1.237171 -0.149085
C -2.000683 1.984617 0.912079
C -1.660824 3.316581 1.055883
C -0.822135 3.923335 0.134924
C -0.333747 3.192665 -0.935307
C -0.668335 1.858016 -1.078180
N -3.104090 -0.449219 -0.022244
C -3.652190 -1.777080 -0.060911
C -3.225249 -2.560905 1.186013
C -5.174028 -1.644175 -0.114523
H 4.460407 -1.994750 1.802062
H 4.396766 -0.289420 2.257080
H 5.953973 -1.060813 1.942144
H 5.338293 -1.372919 -0.446258
H 5.318086 1.019496 -1.138865
H 4.902038 1.500522 0.509023
H 6.465174 0.751319 0.177712
H 2.850631 0.095064 0.116672
H 3.463687 -2.528533 -1.050775
H 1.113275 -2.818102 -1.329797
H 0.792943 -0.033208 -0.033759
H -1.197941 -2.062760 -1.134383
H -2.659299 1.500251 1.616085
H -2.051014 3.886354 1.887093
H -0.555454 4.964469 0.246442
H 0.308832 3.666013 -1.664338
H -0.297302 1.296554 -1.923987
H -3.316452 -2.332778 -0.953057
H -3.670814 -3.553027 1.178032
H -3.553234 -2.033557 2.078985
H -2.141820 -2.658912 1.220254
H -5.524482 -1.100383 0.758468
H -5.467180 -1.090657 -1.002769
H -5.642458 -2.625476 -0.137085
43
int7_conf_7 Eopt -771.431296
C 6.102993 -0.211393 1.485043
C 5.951153 -0.525643 -0.007541
C 6.212401 0.716576 -0.858519
N 4.649899 -1.095354 -0.303428
C 3.486005 -0.397003 -0.209989
C 2.258728 -0.955193 -0.150264
C 1.062991 -0.185679 -0.126333
C -0.181813 -0.700677 -0.060547
C -1.378097 0.112787 -0.029805

C	-2.653029	-0.662103	0.025812
C	-3.053106	-1.415607	-1.071929
C	-4.228147	-2.144242	-1.029087
C	-5.011551	-2.133165	0.112969
C	-4.615989	-1.390423	1.212500
C	-3.443728	-0.656788	1.169311
N	-1.285337	1.382262	-0.056970
C	-2.431755	2.245275	-0.071503
C	-2.375089	3.137509	1.170783
C	-2.356037	3.097515	-1.341212
H	5.365219	0.527409	1.789134
H	7.095771	0.180648	1.688636
H	5.955372	-1.109779	2.080876
H	6.686149	-1.291199	-0.283953
H	5.619992	1.560543	-0.516068
H	7.261768	0.991241	-0.792842
H	5.967871	0.515759	-1.898387
H	4.564043	-2.088357	-0.142545
H	3.585025	0.677564	-0.241797
H	2.163517	-2.033467	-0.120684
H	1.155885	0.891028	-0.159535
H	-0.329507	-1.772508	-0.024847
H	-2.440322	-1.419962	-1.962802
H	-4.533447	-2.723810	-1.888727
H	-5.928568	-2.703525	0.146250
H	-5.223426	-1.381035	2.106263
H	-3.130321	-0.080212	2.028611
H	-3.388069	1.691560	-0.073276
H	-2.449551	2.534659	2.073142
H	-3.193144	3.853789	1.158144
H	-1.429700	3.673112	1.191768
H	-3.174783	3.812656	-1.365088
H	-1.410579	3.632851	-1.365598
H	-2.416709	2.462189	-2.222015

43

int7_conf_8 Eopt -771.433031

C	5.411920	-1.182111	1.442382
C	4.603305	-1.047407	0.154878
C	5.039057	0.187524	-0.641559
N	3.196192	-0.994041	0.482794
C	2.227017	-1.358587	-0.400803
C	0.935631	-0.984353	-0.321168
C	-0.059642	-1.458149	-1.229879
C	-1.352411	-1.085242	-1.264727
C	-1.983093	-0.066535	-0.425849
C	-1.260326	1.203636	-0.204613
C	-1.440780	1.903368	0.985587
C	-0.778099	3.096017	1.205231
C	0.064740	3.611991	0.234060
C	0.234744	2.932183	-0.961030
C	-0.422188	1.735923	-1.181122
N	-3.137973	-0.148457	0.108221
C	-3.957042	-1.326425	0.012628
C	-3.398816	-2.440636	0.905095
C	-5.369890	-0.942147	0.450390
H	5.282461	-0.298532	2.063860
H	6.468546	-1.291805	1.214585
H	5.082961	-2.052325	2.004252
H	4.755727	-1.941172	-0.461455
H	4.440914	0.282470	-1.545304
H	4.905923	1.087340	-0.044215
H	6.085903	0.108553	-0.922206
H	2.937110	-0.263976	1.131975
H	2.557387	-2.037841	-1.176547
H	0.629759	-0.303314	0.459779
H	0.272805	-2.217317	-1.930159
H	-2.020972	-1.600813	-1.944527
H	-2.105598	1.489851	1.727901
H	-0.918080	3.627789	2.135679
H	0.583955	4.544228	0.405285
H	0.881418	3.338652	-1.725734
H	-0.290466	1.210940	-2.116200
H	-3.997522	-1.702685	-1.023803
H	-2.396230	-2.716006	0.583308
H	-4.039917	-3.317840	0.857489
H	-3.349804	-2.094418	1.935168
H	-5.350123	-0.579684	1.474658
H	-5.753091	-0.148884	-0.186029
H	-6.035372	-1.800025	0.388142

43

int7_conf_9 Eopt -771.433762

C	4.845381	-2.046805	1.044899
C	4.741112	-0.895234	0.038619
C	5.727697	0.218981	0.378050
N	3.404279	-0.347891	-0.002366
C	2.404950	-0.897925	-0.743496
C	1.091218	-0.668187	-0.555299
C	0.087695	-1.205995	-1.418667
C	-1.239909	-1.018039	-1.312981
C	-1.907324	-0.157730	-0.328230
C	-1.380011	1.208208	-0.128680
C	-0.741533	1.894638	-1.158308

C	-0.268287	3.177424	-0.953757
C	-0.425334	3.791301	0.277899
C	-1.070233	3.119768	1.304204
C	-1.548598	1.839429	1.101556
N	-2.935563	-0.465205	0.356393
C	-3.553260	-1.760962	0.289765
C	-3.964739	-2.147039	1.711294
C	-4.785704	-1.671253	-0.614896
H	4.640197	-1.687692	2.051478
H	5.842969	-2.477290	1.026875
H	4.124613	-2.824707	0.802628
H	4.961877	-1.278805	-0.964467
H	5.633218	1.034805	-0.333959
H	5.532354	0.605360	1.376145
H	6.746589	-0.156749	0.346713
H	3.111413	0.138860	0.833259
H	2.743026	-1.514263	-1.567169
H	0.773001	-0.055670	0.276089
H	0.452100	-1.858318	-2.205559
H	-1.896492	-1.555886	-1.985208
H	-0.620798	1.421894	-2.122072
H	0.224923	3.702384	-1.759447
H	-0.049663	4.792095	0.435931
H	-1.199512	3.598423	2.264567
H	-2.057687	1.302513	1.886833
H	-2.870490	-2.533461	-0.105164
H	-4.484422	-3.102176	1.709143
H	-4.620003	-1.382723	2.120285
H	-3.085895	-2.225688	2.347074
H	-4.495576	-1.406358	-1.629682
H	-5.308032	-2.624817	-0.635293
H	-5.457438	-0.903536	-0.239026

44

int8_conf_0 Eopt -771.905485

C	-5.764040	0.385178	1.412212
C	-5.579929	0.175000	-0.091902
C	-6.854623	-0.339904	-0.749926
N	-4.483420	-0.750977	-0.377180
C	-3.184412	-0.367046	-0.265125
C	-2.102588	-1.184593	-0.159882
C	-0.766823	-0.652668	-0.123542
C	0.364497	-1.392240	-0.028547
C	1.718373	-0.839499	0.015504
C	1.855417	0.658376	-0.021293
C	2.102213	1.313712	-1.230884
C	2.232814	2.702158	-1.264330

C	2.118050	3.444007	-0.088186
C	1.869566	2.793639	1.121065
C	1.735622	1.405565	1.153884
N	2.713968	-1.650239	0.080652
C	4.084425	-1.152744	0.137597
C	4.766632	-1.757557	1.363914
C	4.804978	-1.557630	-1.148707
H	-6.042060	-0.558737	1.893795
H	-6.553848	1.117052	1.605809
H	-4.837917	0.745945	1.869614
H	-5.301269	1.129865	-0.553855
H	-6.720091	-0.451610	-1.828650
H	-7.132997	-1.313474	-0.330440
H	-7.680703	0.351914	-0.568009
H	-4.684209	-1.732708	-0.218651
H	-3.035421	0.712681	-0.303871
H	-2.239060	-2.263412	-0.108646
H	-0.678063	0.433681	-0.175047
H	0.304496	-2.478279	0.018508
H	2.191049	0.734069	-2.146175
H	2.424530	3.203295	-2.207997
H	2.220591	4.524250	-0.114006
H	1.778673	3.366307	2.038804
H	1.538842	0.897241	2.094350
H	4.119421	-0.058164	0.225517
H	4.249410	-1.460147	2.281088
H	5.806226	-1.421819	1.427299
H	4.755667	-2.850791	1.300889
H	4.777926	-2.645926	-1.268143
H	4.326739	-1.103300	-2.021815
H	5.850479	-1.235857	-1.118260
H	2.546711	-2.636081	0.092471

44

int8_conf_1 Eopt -771.905485

C	-6.453397	-0.007552	1.193138
C	-5.640113	0.174722	-0.086534
C	-6.450209	-0.218050	-1.320195
N	-4.444894	-0.642537	-0.016690
C	-3.200655	-0.208936	-0.049637
C	-2.086227	-1.016425	0.019082
C	-0.795210	-0.502593	-0.020396
C	0.347212	-1.275620	0.037255
C	1.646334	-0.762721	0.007680
C	1.882667	0.693280	0.003692
C	2.489557	1.307787	-1.089585
C	2.693485	2.675590	-1.091041

C	2.307438	3.438441	-0.000707
C	1.705577	2.831882	1.089778
C	1.485329	1.466230	1.092229
N	2.682749	-1.586954	-0.023543
C	4.094133	-1.262526	0.072606
C	4.659711	-1.897831	1.341323
C	4.816585	-1.774725	-1.170940
H	-6.783414	-1.038945	1.293158
H	-7.331396	0.630344	1.165604
H	-5.862113	0.257461	2.066234
H	-5.320908	1.217909	-0.173650
H	-5.856281	-0.102101	-2.223593
H	-6.780395	-1.251627	-1.248076
H	-7.327849	0.415683	-1.401632
H	-4.605208	-1.639580	0.063917
H	-3.073522	0.863414	-0.137409
H	-2.219387	-2.085829	0.106853
H	-0.684285	0.570609	-0.106074
H	0.235324	-2.351208	0.086231
H	2.779168	0.715022	-1.944349
H	3.157386	3.145345	-1.944265
H	2.475000	4.504102	-0.001368
H	1.405783	3.422857	1.941337
H	1.023497	0.988894	1.943849
H	4.186970	-0.175181	0.135730
H	4.129990	-1.538165	2.220146
H	5.709383	-1.640284	1.444290
H	4.576334	-2.981704	1.299504
H	4.738008	-2.857337	-1.240855
H	4.396154	-1.336545	-2.072525
H	5.868421	-1.511026	-1.119868
H	2.485048	-2.581376	-0.038048

44

int8_conf_10		Eopt	-771.903161
C	6.124897	0.325066	-1.111129
C	5.306848	0.311618	0.178233
C	6.096557	-0.304347	1.331045
N	4.091033	-0.449517	-0.032135
C	2.858286	0.004984	0.074983
C	1.723167	-0.747843	-0.132319
C	0.445928	-0.211076	-0.010923
C	-0.714398	-0.941134	-0.182864
C	-1.996887	-0.399023	-0.085746
C	-2.192802	1.060685	-0.028014
C	-1.592197	1.896989	-0.968556
C	-1.808902	3.262036	-0.924221

C	-2.616188	3.808597	0.060546
C	-3.216410	2.984212	0.998911
C	-3.016604	1.617104	0.950518
N	-3.121053	-1.108621	-0.051435
C	-3.338800	-2.552930	-0.005048
C	-2.951475	-3.230601	-1.319825
C	-2.665179	-3.191694	1.208708
H	5.547136	0.747606	-1.929572
H	6.429932	-0.683185	-1.381473
H	7.018265	0.926393	-0.974139
H	5.013872	1.333442	0.438274
H	6.400771	-1.319611	1.087477
H	6.989574	0.283077	1.520883
H	5.498660	-0.325910	2.238912
H	4.225771	-1.421846	-0.282568
H	2.759533	1.049344	0.345610
H	1.829344	-1.789785	-0.401937
H	0.368443	0.834455	0.256423
H	-0.609195	-1.996865	-0.371923
H	-0.983137	1.467957	-1.750194
H	-1.349361	3.900341	-1.662793
H	-2.780237	4.874165	0.094947
H	-3.843695	3.406409	1.768405
H	-3.470463	0.980735	1.697000
H	-4.420865	-2.667814	0.117549
H	-3.414255	-2.717671	-2.159317
H	-3.306157	-4.257160	-1.308260
H	-1.877538	-3.245480	-1.474348
H	-2.946222	-2.665056	2.117628
H	-2.995148	-4.222735	1.295882
H	-1.583191	-3.185141	1.128366
H	-3.966729	-0.554561	-0.031625

44

int8_conf_2		Eopt	-771.906438
C	5.535298	-1.151927	1.233439
C	5.305359	-0.513052	-0.137173
C	6.623808	-0.178335	-0.823058
N	4.536562	0.711642	0.009752
C	3.219539	0.818745	0.063965
C	2.300044	-0.202060	-0.041869
C	0.931625	0.046330	0.007076
C	-0.035481	-0.933536	-0.086497
C	-1.410687	-0.687387	-0.046182
C	-1.928020	0.692687	0.010660
C	-1.689659	1.569827	-1.044949
C	-2.173503	2.864710	-0.992591

C -2.882822 3.299118 0.115376
C -3.111974 2.434029 1.173030
C -2.643981 1.133738 1.121602
N -2.264440 -1.699357 -0.053278
C -3.712252 -1.655026 -0.146964
C -4.142329 -2.339020 -1.443235
C -4.319026 -2.347566 1.070764
H 6.080993 -2.083638 1.119752
H 4.588312 -1.360309 1.725143
H 6.117126 -0.488082 1.868192
H 4.732466 -1.197502 -0.769963
H 7.198364 -1.085526 -0.983949
H 6.448700 0.290520 -1.788410
H 7.217579 0.493329 -0.206610
H 5.091548 1.544108 0.152834
H 2.843540 1.826696 0.195915
H 2.630478 -1.222123 -0.162978
H 0.610702 1.072313 0.133460
H 0.285117 -1.963771 -0.176028
H -1.142970 1.225505 -1.910312
H -1.995158 3.535314 -1.818925
H -3.255996 4.310491 0.154961
H -3.658979 2.770607 2.039902
H -2.811821 0.463220 1.951142
H -4.017175 -0.605585 -0.168810
H -3.694894 -1.847546 -2.303858
H -5.222223 -2.289780 -1.543312
H -3.846720 -3.385970 -1.442866
H -3.991752 -1.871384 1.991535
H -5.402236 -2.293095 1.022951
H -4.029890 -3.395767 1.099118
H -1.875603 -2.635666 -0.074577
44
int8_conf_3 Eopt -771.903915
C -5.590863 1.258072 -0.256472
C -5.737188 -0.255590 -0.215801
C -6.458724 -0.709774 1.054573
N -4.459681 -0.948571 -0.300019
C -3.245765 -0.446344 -0.187763
C -2.092350 -1.201774 -0.217907
C -0.829968 -0.630746 -0.113625
C 0.348600 -1.350506 -0.131549
C 1.620082 -0.780788 -0.038336
C 1.789618 0.684141 -0.000618
C 2.318996 1.304983 1.128740
C 2.460496 2.680249 1.161644

C 2.088600 3.445060 0.067783
C 1.563751 2.832373 -1.058456
C 1.406315 1.458375 -1.093090
N 2.691327 -1.558035 0.023479
C 4.088855 -1.168086 -0.007782
C 4.782287 -1.668934 1.256774
C 4.735830 -1.753760 -1.261612
H -6.580298 1.704518 -0.294150
H -5.045343 1.576320 -1.141630
H -5.090783 1.630252 0.634562
H -6.321923 -0.578485 -1.087229
H -6.581511 -1.790112 1.067773
H -5.896730 -0.411040 1.936369
H -7.443389 -0.255308 1.101606
H -4.536851 -1.955625 -0.380687
H -3.166447 0.625704 -0.077566
H -2.171145 -2.274791 -0.327659
H -0.771545 0.444838 -0.007857
H 0.288051 -2.429173 -0.201132
H 2.597721 0.710107 1.985781
H 2.864763 3.154555 2.042334
H 2.207587 4.516888 0.093324
H 1.275285 3.424851 -1.912866
H 1.004919 0.976585 -1.972439
H 4.133995 -0.076683 -0.049677
H 5.821816 -1.356003 1.254672
H 4.751858 -2.755183 1.305611
H 4.304665 -1.267104 2.146780
H 5.775847 -1.447118 -1.315579
H 4.700435 -2.840834 -1.241258
H 4.226925 -1.402244 -2.156029
H 2.539140 -2.560383 0.013711
44
int8_conf_4 Eopt -771.903876
C 6.809457 -0.927259 0.237131
C 5.695906 -0.091609 -0.385828
C 5.606184 1.281947 0.271521
N 4.451609 -0.837659 -0.255532
C 3.223179 -0.357997 -0.245442
C 2.089801 -1.135476 -0.138509
C 0.813057 -0.586639 -0.129115
C -0.347542 -1.328988 -0.034325
C -1.632294 -0.781872 -0.015391
C -1.829929 0.680012 -0.006575
C -2.458623 1.314175 -1.075907
C -2.626633 2.686832 -1.066051

C -2.182310 3.435208 0.011989
C -1.558206 2.809065 1.078650
C -1.374170 1.438023 1.069456
N -2.691415 -1.577659 -0.012749
C -4.088801 -1.215997 0.138573
C -4.871852 -1.702452 -1.078426
C -4.622911 -1.841434 1.425656
H 6.873889 -1.904903 -0.235617
H 6.634555 -1.059902 1.302397
H 7.765364 -0.429456 0.107196
H 5.904754 0.037833 -1.458006
H 5.260966 1.189134 1.298797
H 6.591542 1.737757 0.280858
H 4.940792 1.948025 -0.270677
H 4.560805 -1.842993 -0.196701
H 3.117718 0.715084 -0.324857
H 2.196558 -2.208799 -0.059484
H 0.727746 0.489503 -0.206162
H -0.262433 -2.407546 0.005599
H -2.793826 0.732420 -1.921552
H -3.108230 3.171809 -1.900863
H -2.321867 4.504800 0.020329
H -1.212937 3.388858 1.920680
H -0.895085 0.945481 1.902824
H -4.150157 -0.126798 0.208647
H -5.914475 -1.415616 -0.983185
H -4.820534 -2.785925 -1.159033
H -4.478104 -1.266468 -1.993083
H -5.658999 -1.551662 1.571595
H -4.575366 -2.926928 1.375734
H -4.048346 -1.502746 2.284577
H -2.521575 -2.576968 -0.036486
44
int8_conf_5 Eopt -771.906449
C 5.471420 0.930167 0.864726
C 5.510127 -0.499255 0.345687
C 6.261818 -0.587144 -0.983998
N 4.183377 -1.072129 0.170207
C 3.015155 -0.460943 0.200525
C 1.810901 -1.086044 -0.044756
C 0.599323 -0.406549 0.007317
C -0.628077 -1.004330 -0.205205
C -1.847034 -0.324785 -0.173342
C -1.909105 1.144679 -0.102995
C -1.184073 1.932087 -0.996732
C -1.274787 3.310753 -0.939504

C -2.078942 3.918318 0.011459
C -2.802677 3.142258 0.902595
C -2.728358 1.763158 0.841142
N -3.022996 -0.940479 -0.217999
C -3.292827 -2.368114 -0.207536
C -3.215323 -2.903482 1.223399
C -4.669199 -2.601480 -0.816495
H 6.487159 1.256564 1.067987
H 4.906248 0.996594 1.791361
H 5.045046 1.605960 0.127102
H 6.024180 -1.129992 1.082986
H 5.768894 0.021202 -1.738571
H 7.277619 -0.225499 -0.857532
H 6.307967 -1.614181 -1.338367
H 4.184098 -2.054121 -0.079004
H 3.018506 0.591757 0.444276
H 1.807750 -2.139630 -0.289322
H 0.626873 0.647038 0.252508
H -0.639405 -2.072073 -0.366814
H -0.578078 1.457559 -1.754138
H -0.719161 3.912505 -1.642029
H -2.144699 4.994073 0.055912
H -3.428654 3.611932 1.645293
H -3.277893 1.162760 1.552199
H -2.538962 -2.866611 -0.825490
H -3.381415 -3.976473 1.225967
H -3.976376 -2.436537 1.843895
H -2.239461 -2.701279 1.658584
H -5.441737 -2.131267 -0.211656
H -4.717467 -2.199792 -1.825773
H -4.877745 -3.666030 -0.863129
H -3.846016 -0.351970 -0.179429
44
int8_conf_6 Eopt -771.906018
C 5.694475 -0.320729 1.483357
C 5.500909 -0.585321 -0.009797
C 5.688963 0.687064 -0.833651
N 4.191081 -1.178039 -0.253765
C 3.034141 -0.559316 -0.102454
C 1.804903 -1.144419 -0.307533
C 0.613293 -0.443491 -0.152204
C -0.636650 -1.004814 -0.322371
C -1.836581 -0.301900 -0.190136
C -1.859355 1.163927 -0.054200
C -1.163413 1.974718 -0.950068
C -1.218944 3.351101 -0.828869

C -1.958295 3.932732 0.188585
C -2.652680 3.133612 1.082785
C -2.613956 1.757438 0.958019
N -3.026058 -0.890728 -0.194174
C -3.326597 -2.312057 -0.226774
C -3.170933 -2.910564 1.172436
C -4.743654 -2.491147 -0.755211
H 6.700265 0.047154 1.661508
H 5.556023 -1.234449 2.056116
H 4.987720 0.424098 1.840326
H 6.235372 -1.326859 -0.336643
H 5.051220 1.490561 -0.475552
H 6.720626 1.016724 -0.757145
H 5.464970 0.500876 -1.881168
H 4.167499 -2.153492 -0.517820
H 3.080859 0.479223 0.196703
H 1.763783 -2.183327 -0.605401
H 0.679412 0.595657 0.142209
H -0.682459 -2.062882 -0.534321
H -0.608700 1.521390 -1.758139
H -0.686833 3.971577 -1.533235
H -1.996516 5.006694 0.282521
H -3.227675 3.583141 1.877320
H -3.139538 1.137820 1.670668
H -2.624564 -2.797720 -0.912374
H -2.165804 -2.745365 1.553414
H -3.358738 -3.979395 1.139848
H -3.881710 -2.457478 1.859235
H -5.465456 -2.030197 -0.084133
H -4.846298 -2.047591 -1.742768
H -4.979991 -3.548251 -0.830563
H -3.832366 -0.287543 -0.086515
44
int8_conf_7 Eopt -771.900200
C 6.346216 0.443406 -1.250725
C 5.503785 0.491446 0.022338
C 6.341094 0.156085 1.254699
N 4.412077 -0.455216 -0.088669
C 3.124795 -0.171956 -0.065282
C 2.127377 -1.117560 -0.175433
C 0.756824 -0.866533 -0.154605
C 0.120497 0.354902 -0.042109
C -1.269736 0.496520 -0.019716
C -2.152660 -0.685369 -0.015905
C -2.134568 -1.565561 1.063682
C -2.962828 -2.673391 1.068350

C -3.801525 -2.920396 -0.006250
C -3.814721 -2.054043 -1.087450
C -3.000133 -0.936499 -1.092732
N -1.813240 1.704728 -0.005547
C -3.213349 2.058827 0.142400
C -3.384262 2.863897 1.429120
C -3.663956 2.861286 -1.075674
H 6.793845 -0.539721 -1.375651
H 7.144432 1.176865 -1.192463
H 5.737637 0.664427 -2.124314
H 5.066260 1.488390 0.135707
H 6.787081 -0.830720 1.156410
H 7.140380 0.882273 1.365816
H 5.729368 0.175695 2.153506
H 4.687822 -1.424377 -0.195613
H 2.880202 0.875482 0.050693
H 2.433287 -2.148717 -0.286521
H 0.120009 -1.737213 -0.242618
H 0.705367 1.263324 0.006042
H -1.483548 -1.366864 1.902225
H -2.950934 -3.345451 1.912294
H -4.443079 -3.787586 -0.002066
H -4.462068 -2.246721 -1.928757
H -3.001139 -0.268041 -1.940698
H -3.789532 1.132608 0.211357
H -2.819583 3.792533 1.380698
H -3.045881 2.290542 2.288869
H -4.432171 3.109335 1.571607
H -4.717918 3.105214 -0.985348
H -3.102984 3.790107 -1.151981
H -3.521015 2.291680 -1.990427
H -1.184796 2.499993 -0.027498
44
int8_conf_8 Eopt -771.902287
C -6.699049 -0.552555 -0.785039
C -5.351815 -0.579425 -0.074193
C -5.491404 -1.067764 1.368595
N -4.793498 0.762301 -0.092225
C -3.511583 1.088204 -0.061320
C -2.440168 0.223029 -0.036367
C -1.124374 0.680497 -0.027895
C -0.029152 -0.157835 -0.012339
C 1.321454 0.220582 0.008254
C 2.321628 -0.858855 -0.008696
C 3.290868 -0.954649 0.989561
C 4.199237 -1.996687 0.977928

C	4.161609	-2.942960	-0.033423
C	3.200084	-2.854706	-1.027437
C	2.276110	-1.826699	-1.011143
N	1.713587	1.483620	0.062581
C	3.052707	2.021287	-0.099908
C	3.083212	2.882064	-1.361786
C	3.424247	2.840104	1.133743
H	-6.590907	-0.188634	-1.803929
H	-7.400875	0.087038	-0.253975
H	-7.116923	-1.553857	-0.823554
H	-4.667987	-1.236833	-0.619630
H	-5.875671	-2.083119	1.378682
H	-4.529130	-1.055309	1.874678
H	-6.181660	-0.433111	1.918823
H	-5.477882	1.505132	-0.056474
H	-3.309078	2.153158	-0.066590
H	-2.602818	-0.843181	-0.018506
H	-0.985393	1.755432	-0.037959
H	-0.215995	-1.221613	-0.005623
H	3.307318	-0.233334	1.792693
H	4.938111	-2.069438	1.760748
H	4.876822	-3.750345	-0.043910
H	3.166801	-3.590721	-1.815581
H	1.531518	-1.752811	-1.789783
H	3.744256	1.183297	-0.215186
H	2.799298	2.296646	-2.233098
H	4.086437	3.265921	-1.519253
H	2.405090	3.728006	-1.269587
H	2.752530	3.688123	1.247828
H	3.372832	2.233721	2.034560
H	4.436925	3.217937	1.032687
H	0.994098	2.194651	0.108247

44

int8_conf_9 Eopt -771.900299

C	6.389290	0.093750	-1.260563
C	5.561907	0.335139	0.000023
C	6.388079	0.094073	1.261596
N	4.416303	-0.553337	-0.000513
C	3.149400	-0.192068	-0.000336
C	2.083895	-1.066807	-0.000144
C	0.765707	-0.628359	-0.000463
C	-0.330980	-1.468672	-0.000404
C	-1.653886	-1.022335	-0.000745
C	-1.920264	0.433889	-0.000417
C	-2.009567	1.126574	1.202910
C	-2.220910	2.494419	1.199761
C	-2.332425	3.179415	0.000930
C	-2.220463	2.495698	-1.198460
C	-2.009167	1.127844	-1.203115
N	-2.639561	-1.908379	-0.001053
C	-4.089233	-1.716657	-0.000912
C	-4.570605	-1.006196	1.262845
C	-4.571050	-1.001889	-1.261808
H	7.228142	0.782196	-1.287933
H	5.786820	0.249035	-2.152356
H	6.779696	-0.921130	-1.273815
H	5.182328	1.361500	0.000057
H	6.776718	-0.921561	1.276513
H	7.228158	0.781068	1.288471
H	5.785041	0.251675	2.152503
H	4.635088	-1.542690	0.000288
H	2.958963	0.874501	-0.000632
H	2.280030	-2.130054	0.000356
H	0.590066	0.439844	-0.000981
H	-0.157741	-2.537488	-0.000125
H	-1.912181	0.592729	2.136630
H	-2.296989	3.024277	2.136545
H	-2.499941	4.244966	0.001430
H	-2.296186	3.026532	-2.134709
H	-1.911629	0.594912	-2.137318
H	-4.503555	-2.729963	-0.002467
H	-4.301154	0.045467	1.250492
H	-4.147988	-1.471797	2.149902
H	-5.652552	-1.081062	1.320420
H	-4.148650	-1.464225	-2.150681
H	-5.652970	-1.076924	-1.319391
H	-4.301967	0.049826	-1.245940
H	-2.343112	-2.876524	-0.000478

49

int9_conf_1 Eopt -1995.763903

C	6.764831	0.812071	0.730774
C	5.406276	0.174173	0.454531
C	5.252431	-0.207897	-1.020871
N	4.357818	1.089502	0.873110
C	3.080046	0.591115	0.968163
C	1.953632	1.263479	0.717800
C	0.641348	0.611174	0.781980
C	-0.523916	1.498205	0.700978
C	-1.804732	1.100069	0.794163
C	-2.169463	-0.292933	1.121062
C	-1.747320	-0.919056	2.288791
C	-2.097453	-2.230217	2.547132

C -2.880565 -2.935091 1.648342
C -3.324975 -2.314194 0.493435
C -2.980908 -0.998628 0.236888
N -2.890292 1.943069 0.603681
C -2.851842 3.034078 -0.354471
C -2.709478 2.516721 -1.789431
C -4.118372 3.868032 -0.184273
H 6.890741 1.709765 0.128808
H 7.563024 0.117277 0.484035
H 6.847493 1.084392 1.779894
H 5.300376 -0.726982 1.069619
H 4.231260 -0.534447 -1.214927
H 5.462145 0.651882 -1.655140
H 5.940358 -1.007691 -1.284002
H 4.400349 1.974891 0.381817
H 3.026190 -0.429883 1.321963
H 1.993594 2.266925 0.318252
H -0.310105 2.507676 0.383985
H -1.155480 -0.365830 3.003033
H -1.763192 -2.705293 3.458156
H -3.155263 -3.959949 1.855030
H -3.976056 -2.846556 -0.193338
H -3.335415 -0.512194 -0.663348
H -1.988679 3.659740 -0.109497
H -1.879627 1.814208 -1.849852
H -2.528263 3.339180 -2.476257
H -3.621659 2.005546 -2.096316
H -4.205632 4.214500 0.842211
H -4.091532 4.730803 -0.844321
H -4.999583 3.276395 -0.426746
H -3.767836 1.440198 0.570298
H 0.585507 -0.152055 1.551639
S 0.555721 -0.567671 -0.719244
O 1.823936 -0.604193 -1.425809
O -0.551026 -0.170127 -1.615464
O 0.209290 -1.892937 -0.160828
K -0.937988 -2.409806 -2.018830
49
int9_conf_10 Eopt -1995.764513
C -2.481767 3.611551 -0.719452
C -3.445929 2.459932 -0.421607
C -4.581284 2.917516 0.488563
N -2.759324 1.339467 0.203181
C -1.924832 0.553226 -0.587457
C -0.804527 -0.013860 -0.150353
C -0.124843 -1.118230 -0.844172
C 1.287095 -1.382250 -0.497816
C 2.180381 -0.506144 -0.012357
C 1.921681 0.945052 0.061587
C 1.476960 1.644305 -1.056609
C 1.229841 3.002532 -0.981026
C 1.416185 3.679848 0.212776
C 1.865575 2.993136 1.328818
C 2.124360 1.636897 1.252854
N 3.397828 -0.927044 0.518532
C 4.624548 -0.234722 0.159432
C 5.727041 -0.669639 1.120428
C 5.024752 -0.503039 -1.295108
H -2.992054 4.416289 -1.242384
H -1.659521 3.255564 -1.336026
H -2.066750 4.006785 0.206551
H -3.869657 2.090983 -1.363230
H -5.250821 2.085146 0.697845
H -4.182764 3.291468 1.429661
H -5.148723 3.713456 0.014212
H -2.277331 1.637708 1.048028
H -2.296314 0.365532 -1.587143
H -0.481883 0.157302 0.870258
H 1.551035 -2.429695 -0.559531
H 1.334937 1.116223 -1.987422
H 0.897478 3.539358 -1.858388
H 1.218641 4.740530 0.270894
H 2.017115 3.518650 2.261089
H 2.473393 1.095571 2.119848
H 4.441936 0.838522 0.282108
H 6.640268 -0.113870 0.924940
H 5.934524 -1.730966 1.001696
H 5.420225 -0.491141 2.147423
H 4.222450 -0.197750 -1.963553
H 5.923614 0.051321 -1.552704
H 5.214559 -1.564299 -1.445325
H 3.501565 -1.933545 0.455762
H -0.320250 -1.135215 -1.917923
S -1.112178 -2.607976 -0.278119
O -0.411316 -3.817084 -0.623820
O -2.414555 -2.441405 -0.954351
O -1.334345 -2.415194 1.167236
K -3.512107 -1.904763 0.916721
49
int9_conf_2 Eopt -1995.766091
C -6.055822 -2.256312 -1.272921
C -4.872035 -1.760325 -0.448452

C	-5.169650	-0.396144	0.191020
N	-3.694215	-1.709682	-1.291228
C	-2.484464	-1.432507	-0.685861
C	-1.470085	-0.775970	-1.247963
C	-0.262515	-0.415626	-0.490821
C	0.912021	0.007306	-1.256068
C	2.193742	-0.205161	-0.915119
C	2.595277	-1.023713	0.248050
C	2.185784	-0.699311	1.537650
C	2.572647	-1.490323	2.606027
C	3.368975	-2.604681	2.401301
C	3.786529	-2.926473	1.119956
C	3.405257	-2.139436	0.049331
N	3.241604	0.218311	-1.727803
C	4.397007	0.838017	-1.094251
C	5.521795	0.939720	-2.119865
C	4.041993	2.210892	-0.514862
H	-5.817985	-3.211899	-1.732513
H	-6.295374	-1.543743	-2.060174
H	-6.931484	-2.380330	-0.641324
H	-4.660921	-2.481414	0.350734
H	-4.288680	-0.056544	0.734497
H	-5.410065	0.332373	-0.584742
H	-6.011211	-0.473399	0.876483
H	-3.839680	-1.174712	-2.139705
H	-2.383974	-1.834541	0.312945
H	-1.572786	-0.342463	-2.232468
H	0.694216	0.604568	-2.132075
H	1.578799	0.180061	1.692713
H	2.251635	-1.232188	3.604728
H	3.667565	-3.220106	3.237868
H	4.410680	-3.793345	0.955958
H	3.725898	-2.384645	-0.952639
H	4.711680	0.178268	-0.279008
H	6.418389	1.341876	-1.656028
H	5.231282	1.595816	-2.938088
H	5.747893	-0.042089	-2.527528
H	3.182829	2.125430	0.148264
H	4.880487	2.617696	0.045009
H	3.789129	2.904668	-1.315299
H	2.907628	0.778129	-2.505144
H	-0.040410	-1.100547	0.323237
S	-0.823800	1.134238	0.422761
O	0.300322	1.796760	1.063867
O	-1.847107	0.727580	1.408530
O	-1.487723	2.020568	-0.551797
K	-2.806270	2.806625	1.146085

49

int9_conf_3 Eopt -1995.765915

C	-6.468320	-1.902611	-1.005749
C	-5.233154	-1.431194	-0.244654
C	-5.427634	-0.023874	0.329459
N	-4.081881	-1.486740	-1.129354
C	-2.841477	-1.295489	-0.568736
C	-1.786894	-0.738875	-1.170174
C	-0.537908	-0.482798	-0.440963
C	0.646384	-0.132520	-1.228296
C	1.918809	-0.447460	-0.943820
C	2.316833	-1.351815	0.154195
C	1.952772	-1.105999	1.474412
C	2.337349	-1.983196	2.474127
C	3.087873	-3.106736	2.171090
C	3.462865	-3.350093	0.859743
C	3.083125	-2.477157	-0.142377
N	2.967523	-0.042120	-1.774252
C	4.135435	0.546015	-1.135889
C	5.240207	0.699027	-2.176430
C	3.796635	1.890759	-0.479965
H	-6.299192	-2.892711	-1.421367
H	-6.695392	-1.218240	-1.820793
H	-7.326959	-1.943301	-0.341039
H	-5.030822	-2.125743	0.579226
H	-4.506163	0.314560	0.801582
H	-5.682535	0.674514	-0.466005
H	-6.227762	-0.018858	1.065506
H	-4.218197	-0.959621	-1.983822
H	-2.744919	-1.682460	0.436299
H	-1.875821	-0.308434	-2.156799
H	0.445181	0.514568	-2.073095
H	1.385336	-0.218468	1.709709
H	2.050196	-1.785573	3.496716
H	3.384135	-3.789631	2.954436
H	4.052061	-4.223397	0.618688
H	3.370174	-2.661316	-1.167171
H	4.465115	-0.152145	-0.358970
H	5.453618	-0.260728	-2.639009
H	6.148046	1.073029	-1.710707
H	4.936227	1.397639	-2.953828
H	4.655118	2.274396	0.066282
H	3.512074	2.618190	-1.241014
H	2.962870	1.764334	0.210106
H	2.631704	0.548383	-2.528374

H -0.349096 -1.189400 0.362736
S -0.940765 1.109502 0.485904
O -2.068336 0.884440 1.373333
O -1.234819 2.150050 -0.516890
O 0.254660 1.559385 1.233942
K -0.492195 3.716499 0.991188
49
int9_conf_4 Eopt -1995.766487
C 5.897370 1.734672 -1.984694
C 4.742743 1.528099 -1.009260
C 5.040926 0.388486 -0.024444
N 3.526498 1.282329 -1.758800
C 2.341437 1.223098 -1.051788
C 1.291803 0.462698 -1.361364
C 0.115921 0.369001 -0.484162
C -1.095512 -0.233495 -1.043771
C -2.354945 0.105890 -0.720123
C -2.672924 1.267833 0.141209
C -2.219006 1.322431 1.455766
C -2.542250 2.405628 2.254642
C -3.320164 3.437429 1.755369
C -3.784572 3.380916 0.451887
C -3.467410 2.299431 -0.350272
N -3.479529 -0.520360 -1.237415
C -3.451533 -1.917150 -1.632435
C -3.153358 -2.835950 -0.443242
C -4.793024 -2.258976 -2.274947
H 6.085650 0.822797 -2.548445
H 6.803564 2.001705 -1.447621
H 5.658316 2.530661 -2.684817
H 4.583031 2.450646 -0.437858
H 5.230433 -0.535544 -0.572447
H 5.914296 0.627106 0.579222
H 4.179250 0.242346 0.625983
H 3.622203 0.524582 -2.425190
H 2.294450 1.895247 -0.205914
H 1.340393 -0.235926 -2.184234
H -0.910862 -1.080601 -1.685907
H -1.628258 0.505101 1.842274
H -2.186486 2.442333 3.274031
H -3.568200 4.282370 2.381592
H -4.395013 4.181714 0.059360
H -3.823286 2.253690 -1.369581
H -2.665271 -2.031678 -2.385175
H -2.233161 -2.528452 0.050505
H -3.047272 -3.865432 -0.775686
H -3.964621 -2.785415 0.281037
H -5.597312 -2.166848 -1.547162
H -4.996286 -1.585611 -3.104006
H -4.782241 -3.279538 -2.647879
H -4.320213 -0.283903 -0.726146
H -0.055623 1.264658 0.106469
S 0.695902 -0.873721 0.809285
O -0.412483 -1.299030 1.650746
O 1.767128 -0.239979 1.605924
O 1.298619 -2.020266 0.102640
K 2.648430 -2.347700 1.927781
49
int9_conf_5 Eopt -1995.765380
C 6.790570 -0.256213 -1.162780
C 5.480161 0.225120 -0.546515
C 5.412309 -0.089545 0.951092
N 4.369196 -0.377438 -1.265911
C 3.110451 0.113894 -0.997550
C 1.975825 -0.589454 -0.999971
C 0.700680 0.008599 -0.591589
C -0.519026 -0.757628 -0.874321
C -1.774328 -0.286066 -0.795362
C -2.089621 1.152428 -0.760188
C -1.513381 2.035016 -1.672296
C -1.817565 3.382425 -1.638998
C -2.711895 3.872676 -0.701978
C -3.314528 3.000491 0.188102
C -3.015700 1.651269 0.151865
N -2.911007 -1.095788 -0.656443
C -2.876098 -2.527829 -0.885926
C -4.139059 -3.140746 -0.286101
C -2.768619 -2.865491 -2.376836
H 6.809427 -0.037473 -2.227483
H 6.900446 -1.330371 -1.027451
H 7.633300 0.238560 -0.687636
H 5.393908 1.308986 -0.686961
H 5.577286 -1.152782 1.119275
H 6.171553 0.468400 1.493688
H 4.429641 0.170358 1.343177
H 4.405997 -1.390300 -1.249687
H 3.082362 1.179131 -0.813804
H 1.995716 -1.661305 -1.140740
H -0.354431 -1.826073 -0.890657
H -0.833767 1.654279 -2.421125
H -1.358307 4.053555 -2.350456
H -2.943424 4.927123 -0.671654

H	-4.026003	3.373257	0.911687
H	-3.504069	0.973824	0.843692
H	-2.008946	-2.927129	-0.350729
H	-4.120141	-4.221705	-0.394650
H	-5.024349	-2.759698	-0.792976
H	-4.207355	-2.895595	0.773485
H	-2.691155	-3.939645	-2.522439
H	-3.648979	-2.506564	-2.908786
H	-1.889167	-2.391094	-2.805537
H	-3.724406	-0.676342	-1.097889
H	0.638925	1.074990	-0.781578
S	0.726075	0.026574	1.307397
O	2.045547	0.376837	1.773239
O	0.265234	-1.307849	1.740297
O	-0.324823	0.997428	1.668821
K	-1.684068	-0.522315	2.548032

49

int9_conf_6 Eopt -1995.765097

C	5.067612	-0.620539	0.307279
C	4.643757	-2.023431	-0.150160
C	5.805526	-2.770583	-0.797389
N	3.535469	-1.969175	-1.082965
C	2.338732	-1.455724	-0.626638
C	1.459072	-0.781694	-1.367611
C	0.232425	-0.168066	-0.822978
C	-0.576655	-0.919347	0.144763
C	-1.918424	-0.925032	0.206635
C	-2.783378	-0.245661	-0.781218
C	-3.707703	-0.995093	-1.505132
C	-4.524981	-0.385297	-2.438591
C	-4.432866	0.980048	-2.654958
C	-3.522433	1.731542	-1.930979
C	-2.699816	1.126641	-0.995876
N	-2.595608	-1.704770	1.140612
C	-3.742110	-1.127481	1.827362
C	-4.485574	-2.241345	2.558174
C	-3.312566	-0.016816	2.790845
H	5.436498	-0.047221	-0.543658
H	5.854182	-0.687161	1.056414
H	4.204402	-0.107009	0.729990
H	4.295401	-2.585491	0.725134
H	6.617714	-2.896478	-0.086358
H	5.480090	-3.751437	-1.133550
H	6.180994	-2.217276	-1.656199
H	3.800453	-1.606883	-1.991517
H	2.137002	-1.672248	0.411477
H	1.698856	-0.536302	-2.392921
H	-0.020574	-1.458768	0.897809
H	-3.772579	-2.059337	-1.331655
H	-5.235780	-0.975336	-2.999612
H	-5.072245	1.456186	-3.384514
H	-3.452375	2.797377	-2.092646
H	-1.996809	1.710058	-0.419992
H	-4.397343	-0.700085	1.061323
H	-5.384893	-1.849726	3.025844
H	-3.854450	-2.675598	3.331162
H	-4.766115	-3.025559	1.859914
H	-2.724503	0.729888	2.260461
H	-4.182323	0.466235	3.229207
H	-2.701875	-0.427952	3.593167
H	-1.956886	-2.141105	1.796591
H	-0.367295	0.304054	-1.596225
S	0.880648	1.297555	0.154459
O	-0.222973	2.061589	0.715526
O	1.702702	2.120885	-0.753832
O	1.774630	0.801883	1.219438
K	2.864866	2.845595	1.091855

49

int9_conf_7 Eopt -1995.765779

C	-5.043030	-0.358000	-0.835472
C	-4.569113	1.010525	-1.340930
C	-5.607488	1.653991	-2.255741
N	-3.307039	0.908120	-2.048319
C	-2.189018	0.593131	-1.305171
C	-1.161039	-0.153112	-1.704097
C	-0.006618	-0.431285	-0.818363
C	0.805534	0.726283	-0.414873
C	2.142650	0.757471	-0.286818
C	3.030504	-0.351465	-0.704169
C	4.041815	-0.100522	-1.628951
C	4.895468	-1.112583	-2.027464
C	4.756116	-2.385337	-1.498937
C	3.761390	-2.637625	-0.569209
C	2.901172	-1.629073	-0.168154
N	2.841869	1.882798	0.126789
C	2.227510	2.919262	0.934066
C	1.752176	2.390703	2.291946
C	3.242662	4.046324	1.107507
H	-5.901311	-0.246768	-0.174560
H	-4.229204	-0.848346	-0.300708
H	-5.330919	-0.988620	-1.675271
H	-4.392205	1.663453	-0.476566

H	-5.250214	2.617189	-2.610009
H	-5.798572	1.018136	-3.117923
H	-6.542397	1.801259	-1.721482
H	-3.379501	0.372664	-2.905856
H	-2.177224	1.026941	-0.314736
H	-1.167960	-0.634543	-2.672005
H	0.219247	1.586227	-0.134781
H	4.143498	0.892081	-2.043954
H	5.671200	-0.908702	-2.751968
H	5.423671	-3.176277	-1.809442
H	3.653840	-3.626625	-0.148312
H	2.134304	-1.823000	0.567005
H	1.368012	3.305528	0.376037
H	1.018531	1.598534	2.156842
H	1.298697	3.192659	2.869184
H	2.595052	1.990607	2.852919
H	4.099294	3.700605	1.683419
H	3.591900	4.390445	0.137136
H	2.790761	4.883526	1.632400
H	3.785087	1.667343	0.421542
H	0.584202	-1.274220	-1.165559
S	-0.736531	-1.046989	0.793868
O	0.271605	-1.794361	1.531480
O	-1.918721	-1.885171	0.513222
O	-1.203313	0.110286	1.586704
K	-2.644622	-1.370676	2.651222

49

int9_conf_8 Eopt -1995.765044

C	-6.793791	-1.271001	0.643592
C	-5.461788	-0.543794	0.481209
C	-5.422852	0.279124	-0.810288
N	-4.382524	-1.516175	0.518544
C	-3.102605	-1.039011	0.676225
C	-1.995662	-1.569293	0.150418
C	-0.684866	-0.935809	0.330192
C	0.477762	-1.705299	-0.120526
C	1.756710	-1.367323	0.116376
C	2.079351	-0.188127	0.953093
C	2.614523	0.968687	0.399048
C	2.868027	2.073377	1.193348
C	2.592788	2.036165	2.549130
C	2.067763	0.884489	3.110025
C	1.815973	-0.220541	2.318672
N	2.800022	-2.174736	-0.303121
C	4.118809	-1.675636	-0.630962
C	5.137789	-2.789231	-0.390553
C	4.182044	-1.184008	-2.082015
H	-6.794660	-1.856224	1.559695
H	-6.963502	-1.941836	-0.196139
H	-7.610494	-0.555193	0.683054
H	-5.310819	0.125783	1.335799
H	-6.129303	1.104196	-0.760713
H	-4.419886	0.673959	-0.967870
H	-5.681263	-0.347108	-1.662632
H	-4.457842	-2.204402	-0.221584
H	-3.023999	-0.177387	1.325727
H	-2.060228	-2.398144	-0.540189
H	0.273895	-2.541991	-0.775886
H	2.826401	1.004256	-0.658643
H	3.308755	2.962855	0.753699
H	2.792196	2.899002	3.168989
H	1.855405	0.847174	4.168869
H	1.412593	-1.122532	2.756421
H	4.335558	-0.845702	0.048565
H	4.932232	-3.637967	-1.039638
H	5.088699	-3.124761	0.641751
H	6.142963	-2.431398	-0.596189
H	3.352819	-0.508708	-2.285716
H	5.119296	-0.666669	-2.272409
H	4.108015	-2.027695	-2.766235
H	2.494853	-2.903552	-0.936210
H	-0.558231	-0.480651	1.309333
S	-0.745034	0.688619	-0.671577
O	-2.030351	0.848550	-1.333341
O	0.365836	0.740139	-1.644238
O	-0.515479	1.769455	0.312646
K	0.416038	3.031126	-1.307212

49

int9_conf_9 Eopt -1995.763298

C	-4.789219	2.235528	-0.299048
C	-4.749244	1.324184	-1.523362
C	-5.200943	-0.096271	-1.163524
N	-3.425023	1.321141	-2.130581
C	-2.355071	0.904211	-1.360810
C	-1.327733	0.169259	-1.779641
C	-0.224366	-0.224069	-0.870203
C	0.628863	0.860696	-0.356154
C	1.966718	0.839837	-0.246811
C	2.810085	-0.275431	-0.725307
C	2.633253	-1.574633	-0.259415
C	3.441789	-2.594663	-0.732106
C	4.430572	-2.332172	-1.665216

C	4.614402	-1.038918	-2.127360
C	3.811722	-0.015933	-1.658583
N	2.668828	1.957153	0.201472
C	3.768923	1.763760	1.134312
C	4.550014	3.069659	1.244584
C	3.265270	1.298339	2.504122
H	-4.317052	3.186509	-0.530876
H	-4.269142	1.779186	0.542192
H	-5.822519	2.419205	-0.012338
H	-5.429484	1.738196	-2.280647
H	-6.147402	-0.072471	-0.625324
H	-5.329860	-0.694210	-2.063852
H	-4.434941	-0.570838	-0.547686
H	-3.427215	0.885618	-3.045883
H	-2.369626	1.240807	-0.337247
H	-1.290535	-0.211312	-2.791563
H	0.087048	1.729130	-0.006841
H	1.866891	-1.777333	0.474236
H	3.298353	-3.600751	-0.365593
H	5.058819	-3.131685	-2.030992
H	5.385385	-0.828612	-2.855067
H	3.947668	0.994507	-2.015683
H	4.419543	0.989698	0.714148
H	4.883992	3.390879	0.261636
H	5.418546	2.936162	1.883919
H	3.923739	3.851471	1.670046
H	2.667281	0.395832	2.393269
H	4.100771	1.087575	3.167027
H	2.644098	2.067869	2.959875
H	2.040678	2.686689	0.520696
H	0.350353	-1.057338	-1.265740
S	-1.023952	-0.942155	0.653886
O	-0.032466	-1.688376	1.410670
O	-2.149963	-1.803770	0.242345
O	-1.594916	0.148747	1.471825
K	-3.105802	-1.391656	2.291489

44

int_S1_conf_1 Eopt -771.882599

C	1.650360	-0.547037	-0.063264
C	1.781113	-1.524730	-1.187039
C	0.763432	-1.771201	-2.014444
C	-0.515539	-1.143760	-1.796460
C	-0.792977	-0.477468	-0.667430
H	0.878146	-2.419965	-2.866993
H	-1.249395	-1.183007	-2.584323
N	0.197946	-0.437319	0.412870

H	2.762475	-1.943076	-1.338377
C	-2.048610	0.218144	-0.385098
C	-2.064722	1.435815	0.300054
C	-3.260297	-0.325852	-0.816726
C	-3.259187	2.086922	0.543663
H	-1.142288	1.914376	0.606404
C	-4.451523	0.327467	-0.565676
H	-3.263650	-1.275053	-1.329868
C	-4.455520	1.533691	0.116567
H	-3.255295	3.032187	1.063734
H	-5.379862	-0.107665	-0.900640
H	-5.385672	2.043344	0.311167
H	2.225078	-0.873529	0.808281
N	2.033637	0.784640	-0.410853
H	2.020610	0.955454	-1.409511
C	3.229449	1.362622	0.201325
H	3.141840	1.194454	1.282055
C	3.217831	2.861948	-0.073261
H	4.041050	3.341235	0.447048
H	3.326433	3.053686	-1.137763
H	2.286396	3.307578	0.265799
C	4.521109	0.723155	-0.307328
H	5.378917	1.190419	0.166551
H	4.549174	-0.341863	-0.084856
H	4.609650	0.856312	-1.383394
C	-0.210317	-1.363091	1.531969
H	-1.288756	-1.206066	1.651562
C	0.036803	-2.817531	1.168039
H	-0.377530	-3.450253	1.948010
H	-0.450753	-3.076364	0.232232
H	1.097325	-3.037830	1.084003
C	0.484702	-0.963497	2.825571
H	1.548420	-1.185760	2.808210
H	0.346985	0.094456	3.040355
H	0.050021	-1.526317	3.646822
H	0.152947	0.518422	0.796614

44

int_S1_conf_10 Eopt -771.874258

C	1.902723	0.767155	-0.269718
C	2.382785	0.659307	1.145265
C	1.574912	0.320110	2.151946
C	0.220983	-0.092793	1.881624
C	-0.351126	0.134588	0.690030
H	1.939494	0.261174	3.164346
H	-0.293451	-0.678867	2.624919
N	0.361569	0.977685	-0.269598

H 3.421933 0.900195 1.298412
C -1.641610 -0.398530 0.256641
C -1.867631 -0.758427 -1.074910
C -2.670012 -0.588672 1.182975
C -3.083616 -1.288868 -1.463148
H -1.084499 -0.676419 -1.819820
C -3.884910 -1.113750 0.788316
H -2.521573 -0.299555 2.211631
C -4.097634 -1.463651 -0.535748
H -3.237489 -1.570996 -2.493123
H -4.670065 -1.247518 1.515469
H -5.046260 -1.873574 -0.843374
H 2.290381 1.712163 -0.666149
N 2.190875 -0.263793 -1.205020
H 2.708856 0.075565 -2.001745
C 2.627040 -1.630255 -0.886262
H 2.720226 -2.098271 -1.872975
C 1.577694 -2.409388 -0.102066
H 0.594808 -2.307209 -0.554564
H 1.845095 -3.462050 -0.112217
H 1.529516 -2.086116 0.932641
C 3.990540 -1.695847 -0.198013
H 4.713317 -1.064054 -0.710291
H 3.920432 -1.384100 0.840665
H 4.358686 -2.717287 -0.220346
C 0.007393 2.441184 -0.135638
H 0.690873 2.851424 0.614201
C 0.205508 3.121438 -1.483602
H 1.201709 2.973139 -1.892460
H -0.524789 2.756974 -2.203522
H 0.056722 4.191707 -1.372702
C -1.424245 2.648399 0.338971
H -1.621524 3.716199 0.379086
H -2.138963 2.199142 -0.346282
H -1.585604 2.244268 1.333684
H 0.052285 0.674829 -1.203142
44
int_S1_conf_2 Eopt -771.881585
C 1.722184 -0.626828 -0.096849
C 1.893261 -1.457159 -1.333401
C 0.884982 -1.604816 -2.196291
C -0.378356 -0.960798 -1.931466
C -0.673690 -0.479502 -0.715298
H 1.007459 -2.144747 -3.120565
H -1.058445 -0.805470 -2.752382
N 0.265560 -0.744773 0.380767

H 2.879837 -1.846216 -1.521377
C -1.857499 0.307644 -0.377703
C -3.082410 0.037877 -0.992472
C -1.783424 1.360683 0.537848
C -4.200253 0.790659 -0.689965
H -3.159270 -0.780662 -1.691285
C -2.904357 2.113869 0.832062
H -0.839332 1.640771 0.989826
C -4.116625 1.827914 0.225551
H -5.140876 0.565716 -1.167471
H -2.829700 2.930545 1.533341
H -4.990016 2.414539 0.461689
H 2.334039 -1.019771 0.720549
N 1.985366 0.758609 -0.269022
H 1.936206 1.064114 -1.233074
C 3.121493 1.365154 0.422072
H 3.061192 1.045120 1.469850
C 2.959240 2.878976 0.351910
H 1.991316 3.180312 0.743324
H 3.735720 3.363454 0.935532
H 3.038519 3.221630 -0.676743
C 4.464423 0.929491 -0.163509
H 4.532168 1.221921 -1.209143
H 5.277867 1.403055 0.377445
H 4.592932 -0.149024 -0.095212
C -0.002037 -2.047364 1.094558
H 0.507679 -2.823159 0.515228
C 0.565070 -1.967262 2.505145
H 0.038329 -1.212616 3.086451
H 0.428344 -2.923904 3.001330
H 1.628546 -1.744474 2.522676
C -1.490758 -2.360509 1.150856
H -2.041545 -1.572628 1.659371
H -1.912736 -2.503745 0.160747
H -1.630838 -3.283030 1.707273
H 0.128656 0.013232 1.062112
44
int_S1_conf_4 Eopt -771.881058
C 1.754377 -0.467355 -0.247486
C 1.897849 -1.598862 -1.219624
C 0.837791 -2.041667 -1.900264
C -0.448266 -1.419917 -1.694963
C -0.682756 -0.638650 -0.630642
H 0.927389 -2.812230 -2.647930
H -1.200826 -1.533520 -2.457188
N 0.360466 -0.548466 0.398601

H 2.895798 -1.965714 -1.391895
C -1.892421 0.148833 -0.404788
C -1.826112 1.414588 0.183153
C -3.138026 -0.340880 -0.804451
C -2.973721 2.161436 0.371099
H -0.872768 1.855306 0.447872
C -4.282011 0.407956 -0.608800
H -3.207728 -1.321866 -1.248440
C -4.205077 1.658765 -0.016826
H -2.905687 3.141768 0.816684
H -5.237720 0.013357 -0.916238
H -5.098850 2.242171 0.136243
H 2.465034 -0.571537 0.574649
N 1.862942 0.821781 -0.836107
H 1.770440 0.809326 -1.842331
C 2.911255 1.742506 -0.391298
H 2.766145 2.642201 -0.997101
C 4.322135 1.208256 -0.640209
H 4.532562 0.337821 -0.021782
H 4.444991 0.930196 -1.684647
H 5.054196 1.974336 -0.404037
C 2.704693 2.118408 1.074119
H 1.691595 2.478537 1.238034
H 2.902195 1.283391 1.742475
H 3.389945 2.918186 1.337896
C 0.252024 -1.641456 1.431037
H 0.692397 -2.534998 0.978475
C 1.019582 -1.241558 2.684182
H 0.905634 -2.020229 3.432958
H 2.085193 -1.121738 2.511001
H 0.622223 -0.318419 3.102211
C -1.203288 -1.918903 1.784932
H -1.235833 -2.679316 2.560189
H -1.692795 -1.026006 2.167015
H -1.764409 -2.292124 0.933801
H 0.218864 0.348907 0.879604
44
int_S1_conf_5 Eopt -771.881658
C 1.815347 -0.727273 -0.244820
C 1.883690 -1.716653 -1.369504
C 0.814393 -1.941825 -2.136238
C -0.413114 -1.230516 -1.872708
C -0.616890 -0.597761 -0.708072
H 0.858392 -2.601151 -2.987198
H -1.142536 -1.156854 -2.661870
N 0.389838 -0.756240 0.347765

H 2.845805 -2.159317 -1.565670
C -1.765075 0.249418 -0.393857
C -3.027578 -0.056995 -0.907507
C -1.623818 1.392043 0.397829
C -4.114230 0.748610 -0.628124
H -3.158248 -0.943995 -1.507636
C -2.713687 2.197312 0.669694
H -0.652642 1.696749 0.767663
C -3.962665 1.875891 0.163807
H -5.083714 0.494506 -1.026512
H -2.586215 3.081760 1.274542
H -4.811650 2.503673 0.382091
H 2.476009 -1.027437 0.574994
N 2.087390 0.603280 -0.639356
H 2.095274 0.748474 -1.638453
C 3.121089 1.368575 0.058291
H 4.015560 0.744719 0.215665
C 2.592642 1.824335 1.418551
H 3.351549 2.411715 1.926324
H 1.712711 2.449645 1.286793
H 2.344974 0.979871 2.059044
C 3.498512 2.567140 -0.800972
H 4.251090 3.165137 -0.296151
H 3.906250 2.244216 -1.756037
H 2.626013 3.189540 -0.983548
C 0.147041 -1.965805 1.215136
H 0.572638 -2.817850 0.676574
C 0.843580 -1.776691 2.556030
H 0.420559 -0.928781 3.092000
H 0.689832 -2.662986 3.165241
H 1.917024 -1.636834 2.461560
C -1.341036 -2.202613 1.436404
H -1.812572 -1.340935 1.903368
H -1.858383 -2.428518 0.509160
H -1.463325 -3.054320 2.099685
H 0.315812 0.076539 0.945858
44
int_S1_conf_6 Eopt -771.880312
C 1.844564 0.744581 -0.308080
C 2.267509 0.990757 1.108756
C 1.453709 0.766287 2.141545
C 0.114854 0.281897 1.918542
C -0.424507 0.242880 0.692059
H 1.783854 0.910105 3.157221
H -0.439640 -0.105371 2.757247
N 0.321489 0.789847 -0.445309

H 3.284285 1.324339 1.238684
C -1.748986 -0.288439 0.369807
C -1.978613 -0.998707 -0.811428
C -2.809391 -0.106088 1.260234
C -3.231618 -1.511184 -1.089183
H -1.172107 -1.200740 -1.506097
C -4.061211 -0.616172 0.974277
H -2.652000 0.458098 2.166292
C -4.277110 -1.318468 -0.200579
H -3.390649 -2.067247 -1.999960
H -4.871586 -0.462286 1.669246
H -5.254097 -1.717153 -0.422366
H 2.224744 1.551059 -0.942062
N 2.234542 -0.497029 -0.906318
H 3.054671 -0.423344 -1.492937
C 2.289894 -1.729319 -0.114176
H 1.498280 -1.671533 0.639429
C 3.637057 -1.925858 0.577749
H 4.436294 -1.972759 -0.159279
H 3.845040 -1.113635 1.270044
H 3.634941 -2.856792 1.136596
C 1.997098 -2.895003 -1.056372
H 2.762037 -2.959925 -1.825896
H 1.987472 -3.827930 -0.501728
H 1.031328 -2.772489 -1.539709
C -0.265502 2.120494 -0.849737
H -1.351409 1.977731 -0.801832
C 0.126867 3.219149 0.124024
H -0.412535 4.126392 -0.133555
H -0.135269 2.951876 1.144016
H 1.190198 3.437791 0.081045
C 0.116353 2.451365 -2.284864
H 1.170997 2.692143 -2.387808
H -0.125768 1.632452 -2.959135
H -0.447651 3.322364 -2.606588
H 0.150565 0.148096 -1.234931
44
int_S1_conf_7 Eopt -771.879871
C 1.935194 0.748806 -0.209590
C 2.251455 0.999413 1.230255
C 1.382260 0.750031 2.209731
C 0.066016 0.238348 1.920097
C -0.435603 0.216243 0.676556
H 1.650762 0.900412 3.242663
H -0.508988 -0.171611 2.733654
N 0.345253 0.786279 -0.420374

H 3.254589 1.342533 1.422558
C -1.758903 -0.296859 0.315669
C -2.007646 -0.898054 -0.921649
C -2.814070 -0.190073 1.226135
C -3.264222 -1.384516 -1.229363
H -1.218442 -1.034073 -1.651522
C -4.068882 -0.674175 0.912044
H -2.651651 0.298651 2.173936
C -4.299274 -1.273878 -0.315854
H -3.433111 -1.855330 -2.185344
H -4.871047 -0.578838 1.626624
H -5.278582 -1.653030 -0.560278
H 2.277949 1.590102 -0.818252
N 2.485181 -0.421566 -0.699142
H 2.616606 -0.497814 -1.695565
C 2.531353 -1.699475 0.000530
H 2.450276 -1.485486 1.069646
C 3.882414 -2.350285 -0.280102
H 3.984908 -3.265090 0.295130
H 3.975479 -2.595665 -1.335367
H 4.690373 -1.677382 -0.006883
C 1.386370 -2.622742 -0.420635
H 1.511555 -3.595017 0.045717
H 0.419943 -2.230140 -0.111907
H 1.389968 -2.766127 -1.499795
C -0.221477 2.126388 -0.816447
H -1.309961 1.990638 -0.816187
C 0.132283 3.205016 0.194186
H -0.169531 2.916296 1.197119
H 1.195897 3.425396 0.197962
H -0.399053 4.116283 -0.065804
C 0.215881 2.490487 -2.228611
H 1.276298 2.721450 -2.287935
H -0.013370 1.694113 -2.934438
H -0.326515 3.376483 -2.546406
H 0.193714 0.172208 -1.230418
44
int_S1_conf_8 Eopt -771.879233
C 1.964882 0.843226 -0.495097
C 2.332206 1.299857 0.880478
C 1.507726 1.168751 1.920793
C 0.211199 0.559953 1.759406
C -0.339612 0.356821 0.552793
H 1.809048 1.469592 2.910836
H -0.295152 0.205487 2.641871
N 0.350085 0.859010 -0.630766

H	3.332307	1.688771	0.980420
C	-1.603524	-0.349284	0.333178
C	-2.642401	-0.215696	1.258006
C	-1.797976	-1.182341	-0.771951
C	-3.834784	-0.889987	1.080128
H	-2.519977	0.441835	2.104779
C	-2.990239	-1.860303	-0.941233
H	-1.009046	-1.347427	-1.496002
C	-4.013660	-1.714419	-0.019226
H	-4.628395	-0.769213	1.800587
H	-3.118286	-2.507994	-1.794509
H	-4.943798	-2.241960	-0.156659
H	2.256694	1.598205	-1.234042
N	2.484631	-0.384038	-0.838908
H	2.611340	-0.595242	-1.815532
C	2.699286	-1.518139	0.051392
H	2.632479	-1.143136	1.075875
C	4.101002	-2.070483	-0.191512
H	4.183575	-2.469196	-1.199855
H	4.845041	-1.288769	-0.064204
H	4.315999	-2.869183	0.511271
C	1.641933	-2.601468	-0.161355
H	0.651090	-2.250136	0.117241
H	1.630088	-2.923498	-1.200847
H	1.874324	-3.464709	0.454476
C	-0.179521	2.185695	-1.114560
H	0.477405	2.471624	-1.944809
C	-1.596286	2.036630	-1.653400
H	-2.306270	1.828456	-0.857702
H	-1.890753	2.967836	-2.129478
H	-1.664169	1.247697	-2.399607
C	-0.113499	3.256393	-0.037915
H	0.903012	3.419710	0.307141
H	-0.483818	4.189720	-0.452549
H	-0.737054	2.996367	0.812922
H	0.150895	0.189138	-1.383466

44

int_S1_conf_9 Eopt -771.875426

C	1.912388	0.727537	-0.537131
C	2.401430	0.909781	0.865652
C	1.608606	0.771544	1.930039
C	0.249368	0.323580	1.768710
C	-0.337022	0.267196	0.563923
H	1.986061	0.917828	2.928868
H	-0.268536	-0.058598	2.632832
N	0.363391	0.823290	-0.594507

H	3.439918	1.184360	0.954043
C	-1.640770	-0.344423	0.307827
C	-2.669170	-0.215459	1.244466
C	-1.875315	-1.098649	-0.844681
C	-3.895138	-0.814477	1.028874
H	-2.510495	0.378490	2.131377
C	-3.101334	-1.702183	-1.051080
H	-1.091563	-1.262002	-1.575289
C	-4.116286	-1.558356	-0.119245
H	-4.681192	-0.698786	1.758393
H	-3.262975	-2.290746	-1.940794
H	-5.072811	-2.027169	-0.286184
H	2.249513	1.598318	-1.113693
N	2.271787	-0.447155	-1.260549
H	2.824017	-0.239929	-2.079619
C	2.733182	-1.712545	-0.672020
H	2.848222	-2.365235	-1.545273
C	1.690127	-2.341393	0.244183
H	1.608192	-1.805143	1.184113
H	0.714782	-2.375100	-0.233847
H	1.989732	-3.361523	0.465526
C	4.088848	-1.611291	0.027569
H	3.997948	-1.113632	0.989348
H	4.482517	-2.608725	0.199643
H	4.802792	-1.067767	-0.587786
C	-0.122282	2.210640	-0.948104
H	0.516769	2.534330	-1.778017
C	-1.561497	2.174660	-1.441656
H	-1.694790	1.450855	-2.242660
H	-2.256630	1.943341	-0.639637
H	-1.819568	3.154456	-1.834567
C	0.038883	3.175899	0.214781
H	1.069364	3.233024	0.553417
H	-0.265623	4.167000	-0.109242
H	-0.590610	2.888404	1.052383
H	0.114598	0.218638	-1.390576

44

int_S2_conf_1 Eopt -771.880139

C	1.348299	0.492675	-0.388290
C	1.558119	1.953276	-0.107639
C	0.488200	2.725083	-0.334406
C	-0.767539	2.100538	-0.642571
C	-0.982377	0.809244	-0.293019
H	0.521832	3.795923	-0.221497
H	-1.568423	2.695384	-1.044460
N	0.090349	0.002246	0.156415

H 2.505203 2.315648 0.253195
C -2.294090 0.164486 -0.343663
C -2.393823 -1.193648 -0.651411
C -3.462780 0.888920 -0.108100
C -3.628902 -1.806303 -0.731479
H -1.494847 -1.758315 -0.849837
C -4.696213 0.270986 -0.187755
H -3.399731 1.932814 0.158310
C -4.783727 -1.076126 -0.499974
H -3.692886 -2.855022 -0.978792
H -5.593091 0.841331 -0.001337
H -5.748074 -1.555747 -0.561658
H 1.250290 0.347124 -1.480711
N 2.493728 -0.339055 0.067154
H 2.732315 -0.039882 1.021854
C 3.721142 -0.273955 -0.797387
H 3.801049 0.752685 -1.165592
C 3.562038 -1.237784 -1.960709
H 4.416317 -1.150996 -2.625241
H 3.516819 -2.266543 -1.608816
H 2.670234 -1.022800 -2.543678
C 4.931072 -0.613701 0.058913
H 5.832101 -0.559673 -0.544746
H 5.046945 0.087433 0.882968
H 4.859488 -1.623449 0.457521
C -0.044007 -0.454850 1.545637
H -1.126321 -0.524571 1.690139
C 0.529578 -1.861828 1.764465
H 1.574713 -1.855828 2.072483
H -0.023561 -2.344247 2.565880
H 0.400780 -2.475478 0.874880
C 0.486756 0.540821 2.579882
H 0.064910 1.527341 2.404777
H 0.190406 0.216630 3.573851
H 1.573784 0.625111 2.570335
H 2.170734 -1.314223 0.120216
44
int_S2_conf_10 Eopt -771.875415
C 1.367297 0.368741 -0.345190
C 1.678261 1.833587 -0.443878
C 0.625803 2.570802 -0.822155
C -0.649615 1.918744 -0.996231
C -0.909109 0.745667 -0.375146
H 0.693684 3.635188 -0.973401
H -1.407669 2.408087 -1.582666
N 0.144706 0.135349 0.405181

H 2.655282 2.227130 -0.224367
C -2.186334 0.048022 -0.422912
C -2.214866 -1.347441 -0.355718
C -3.389432 0.739507 -0.577028
C -3.411199 -2.030116 -0.447979
H -1.286762 -1.887327 -0.242165
C -4.584065 0.051043 -0.664049
H -3.388559 1.818304 -0.600561
C -4.599450 -1.333505 -0.602529
H -3.419455 -3.108450 -0.400901
H -5.508391 0.596292 -0.776130
H -5.534305 -1.867472 -0.671541
H 1.124132 -0.006845 -1.355965
N 2.472150 -0.502109 0.165801
H 3.072596 0.041896 0.797349
C 3.343366 -1.157319 -0.865307
H 2.677397 -1.656839 -1.575293
C 4.215178 -2.186614 -0.162633
H 4.880826 -1.713528 0.556294
H 3.615836 -2.936717 0.348582
H 4.831743 -2.701486 -0.893279
C 4.171840 -0.104155 -1.580564
H 4.838988 0.403160 -0.886885
H 4.784553 -0.580061 -2.340367
H 3.543919 0.632876 -2.073325
C 0.034120 0.615764 1.809083
H -0.289964 1.666987 1.798942
C -1.029030 -0.220004 2.520455
H -1.998858 -0.108352 2.047660
H -0.758760 -1.272836 2.505753
H -1.116045 0.102870 3.553684
C 1.329679 0.557855 2.616495
H 1.110036 0.938174 3.611298
H 1.693411 -0.460639 2.742017
H 2.102114 1.203434 2.202075
H 2.011597 -1.229995 0.728028
44
int_S2_conf_2 Eopt -771.879177
C 1.361893 0.543352 -0.263080
C 1.562896 1.933604 0.269188
C 0.498646 2.737261 0.152630
C -0.749632 2.179316 -0.284759
C -0.971685 0.848380 -0.160023
H 0.529921 3.772887 0.447728
H -1.542038 2.836045 -0.596741
N 0.090675 -0.026679 0.167100

H	2.499976	2.226318	0.710744
C	-2.284052	0.228049	-0.340292
C	-2.382235	-1.055784	-0.879876
C	-3.454350	0.906684	-0.000017
C	-3.617590	-1.639197	-1.082325
H	-1.481981	-1.581709	-1.161029
C	-4.687916	0.317915	-0.202882
H	-3.392629	1.888711	0.443384
C	-4.773940	-0.954564	-0.744355
H	-3.680269	-2.628902	-1.508625
H	-5.586103	0.851906	0.066452
H	-5.738477	-1.411588	-0.901909
H	1.272906	0.592249	-1.365070
N	2.496594	-0.371328	0.050416
H	2.994347	-0.003405	0.870497
C	3.499420	-0.596804	-1.042868
H	2.934260	-0.873448	-1.937799
C	4.401520	-1.748434	-0.626000
H	4.960482	-1.504464	0.274876
H	3.834076	-2.661033	-0.456094
H	5.120138	-1.951266	-1.414309
C	4.284794	0.678571	-1.294446
H	3.632219	1.503421	-1.566638
H	4.863488	0.958189	-0.416709
H	4.980116	0.520361	-2.113348
C	-0.075235	-0.722336	1.449887
H	-1.160572	-0.777368	1.575231
C	0.449934	-2.167423	1.412739
H	0.363698	-2.589819	0.412861
H	1.471046	-2.263216	1.786650
H	-0.164143	-2.781478	2.066722
C	0.481809	0.048784	2.648483
H	0.166866	-0.440252	3.566271
H	1.570076	0.092036	2.650959
H	0.095198	1.064753	2.655871
H	2.080858	-1.277115	0.315393

44

int_S2_conf_3 Eopt -771.880081

C	1.390762	0.744909	-0.362966
C	1.468484	2.228874	-0.134662
C	0.337908	2.891042	-0.407497
C	-0.850607	2.142470	-0.709527
C	-0.955359	0.850938	-0.313960
H	0.273277	3.964311	-0.341658
H	-1.691905	2.646969	-1.150414
N	0.177406	0.166239	0.195151

H	2.377494	2.691468	0.211412
C	-2.198661	0.083255	-0.361170
C	-2.159170	-1.295361	-0.578900
C	-3.438414	0.705011	-0.209298
C	-3.327290	-2.028231	-0.653928
H	-1.204265	-1.782566	-0.709431
C	-4.604346	-0.032814	-0.284146
H	-3.484653	1.764729	-0.010900
C	-4.553349	-1.399408	-0.507407
H	-3.283176	-3.092084	-0.830887
H	-5.557123	0.459148	-0.162999
H	-5.465490	-1.972616	-0.565524
H	1.294013	0.573187	-1.451089
N	2.608166	0.021368	0.102389
H	3.353670	0.709894	0.260180
C	3.140372	-1.038953	-0.818849
H	2.276422	-1.602162	-1.182454
C	4.062375	-1.952770	-0.026288
H	4.901680	-1.398764	0.388742
H	3.535369	-2.457377	0.779890
H	4.465865	-2.718083	-0.682475
C	3.869824	-0.378415	-1.976473
H	4.191913	-1.135597	-2.684858
H	3.234633	0.323553	-2.510133
H	4.755554	0.147088	-1.625026
C	0.053572	-0.191757	1.615261
H	-1.021825	-0.340905	1.749309
C	0.740734	-1.525113	1.952242
H	1.745365	-1.400663	2.364177
H	0.166841	-2.032707	2.723355
H	0.766341	-2.181447	1.085520
C	0.483056	0.920575	2.575088
H	-0.053328	1.839170	2.351304
H	0.241848	0.628073	3.593597
H	1.551514	1.127742	2.528056
H	2.371607	-0.419121	1.004417

44

int_S2_conf_4 Eopt -771.882886

C	1.562941	0.856122	-0.533150
C	1.592731	2.357854	-0.478167
C	0.422724	2.928368	-0.793149
C	-0.729380	2.087578	-0.984507
C	-0.765445	0.851400	-0.432532
H	0.301112	3.997519	-0.846153
H	-1.597739	2.487969	-1.477741
N	0.408697	0.311421	0.163206

H	2.486258	2.894071	-0.208606
C	-1.964049	0.015835	-0.408836
C	-1.858012	-1.362238	-0.600938
C	-3.228933	0.578082	-0.235781
C	-2.988553	-2.155984	-0.620758
H	-0.883830	-1.801107	-0.757292
C	-4.356364	-0.220914	-0.250236
H	-3.322192	1.640566	-0.068015
C	-4.240075	-1.588317	-0.442590
H	-2.894908	-3.219722	-0.777972
H	-5.329268	0.223660	-0.108555
H	-5.121937	-2.209582	-0.454507
H	1.417857	0.548281	-1.585110
N	2.829521	0.227359	-0.066435
H	3.615559	0.722611	-0.502753
C	2.949872	-1.244429	-0.357966
H	1.939395	-1.652897	-0.269728
C	3.862870	-1.873643	0.681520
H	4.862498	-1.446406	0.637144
H	3.466262	-1.750833	1.687208
H	3.951310	-2.939417	0.492457
C	3.478287	-1.433010	-1.769042
H	4.491121	-1.045382	-1.860206
H	3.505931	-2.491750	-2.009044
H	2.844644	-0.946364	-2.506011
C	0.398557	0.260931	1.641392
H	1.433140	0.397026	1.988853
C	-0.435379	1.368626	2.284196
H	-0.311465	1.334399	3.363297
H	-0.121109	2.348649	1.931791
H	-1.489198	1.236348	2.057509
C	-0.072708	-1.112079	2.119030
H	0.041298	-1.180753	3.197482
H	-1.119912	-1.259576	1.875345
H	0.503599	-1.911407	1.659571
H	2.889557	0.367546	0.952723

44

int_S2_conf_5 Eopt -771.880084

C	1.490534	0.521510	-0.320379
C	1.690559	2.005422	-0.206146
C	0.621325	2.727883	-0.564593
C	-0.610283	2.047429	-0.861696
C	-0.832442	0.807588	-0.362404
H	0.636213	3.805012	-0.576054
H	-1.389269	2.570127	-1.388226
N	0.226908	0.103485	0.262247

H	2.621140	2.418724	0.142983
C	-2.119837	0.120545	-0.440041
C	-2.159805	-1.259316	-0.642661
C	-3.320920	0.824971	-0.349350
C	-3.370408	-1.917108	-0.748540
H	-1.234373	-1.806304	-0.745360
C	-4.528670	0.162089	-0.453055
H	-3.302588	1.889505	-0.172145
C	-4.557556	-1.209582	-0.650790
H	-3.389020	-2.983997	-0.911917
H	-5.451608	0.715692	-0.375596
H	-5.502073	-1.724728	-0.730209
H	1.404106	0.250665	-1.388568
N	2.626943	-0.264622	0.244123
H	2.948714	0.198753	1.102516
C	3.804937	-0.438167	-0.675154
H	3.917415	0.500759	-1.224956
C	3.526430	-1.582508	-1.633999
H	3.450962	-2.526842	-1.098559
H	2.612764	-1.423281	-2.200594
H	4.342572	-1.667871	-2.345244
C	5.041946	-0.695440	0.170568
H	4.938865	-1.610231	0.750368
H	5.907583	-0.808949	-0.475305
H	5.244384	0.133443	0.845630
C	0.145779	-0.097535	1.725152
H	1.105871	0.213378	2.159949
C	-0.929320	0.752193	2.397753
H	-0.805512	1.802587	2.144275
H	-1.923463	0.433608	2.099401
H	-0.845190	0.646256	3.476016
C	-0.077092	-1.579792	2.034226
H	-1.075020	-1.876670	1.725625
H	0.640130	-2.218693	1.519231
H	0.015742	-1.752373	3.102911
H	2.263637	-1.191694	0.505907

44

int_S2_conf_6 Eopt -771.880084

C	-1.461714	-0.143501	-0.459700
C	-1.724688	-1.486699	-1.082425
C	-0.670816	-2.027590	-1.704521
C	0.601185	-1.363182	-1.607399
C	0.845821	-0.497338	-0.596592
H	-0.733214	-2.966727	-2.228338
H	1.383741	-1.620940	-2.299554
N	-0.206852	-0.103343	0.280256

H	-2.695133	-1.947896	-1.012722
C	2.154377	0.121888	-0.392789
C	2.236480	1.472805	-0.052799
C	3.331926	-0.597069	-0.595081
C	3.466335	2.086626	0.082323
H	1.327044	2.040444	0.081097
C	4.560059	0.019985	-0.451506
H	3.280738	-1.647140	-0.841493
C	4.631067	1.361762	-0.112928
H	3.518621	3.134237	0.337500
H	5.465382	-0.547467	-0.602488
H	5.591028	1.841545	-0.003026
H	-1.326001	0.603480	-1.263502
N	-2.603718	0.322070	0.379905
H	-2.895429	-0.457685	0.982026
C	-3.799295	0.819516	-0.387803
H	-3.887932	0.194138	-1.280549
C	-3.570666	2.268209	-0.782527
H	-3.511355	2.905618	0.097432
H	-2.664191	2.389242	-1.369741
H	-4.402128	2.615284	-1.388669
C	-5.033310	0.656730	0.485338
H	-4.954381	1.251428	1.392859
H	-5.910709	0.996264	-0.057195
H	-5.197969	-0.384449	0.754833
C	-0.167835	-0.679244	1.637707
H	-1.022439	-0.257517	2.184818
C	-0.274290	-2.206486	1.658712
H	-1.212097	-2.558483	1.233106
H	0.542416	-2.645069	1.091223
H	-0.206395	-2.561868	2.683104
C	1.086670	-0.238090	2.387822
H	0.989060	-0.518897	3.433323
H	1.973480	-0.718112	1.986992
H	1.218132	0.838136	2.328843
H	-2.264136	1.081405	0.984003

44

int_S2_conf_7 Eopt -771.878671

C	1.575189	0.695982	-0.638040
C	1.653553	2.197593	-0.729333
C	0.498206	2.799257	-1.033685
C	-0.695981	2.000473	-1.097490
C	-0.753721	0.807233	-0.462764
H	0.424952	3.864287	-1.177263
H	-1.570094	2.397054	-1.583974
N	0.417482	0.250094	0.129376

H	2.585247	2.714320	-0.567642
C	-1.987047	0.023604	-0.386817
C	-1.955142	-1.355064	-0.596410
C	-3.216426	0.644843	-0.168004
C	-3.122745	-2.092916	-0.582928
H	-1.011448	-1.840432	-0.797688
C	-4.381274	-0.098571	-0.147989
H	-3.251619	1.709422	0.009249
C	-4.337976	-1.467953	-0.354247
H	-3.086412	-3.157899	-0.754882
H	-5.326034	0.391521	0.030116
H	-5.248683	-2.046022	-0.339766
H	1.399337	0.297066	-1.654905
N	2.858459	0.105073	-0.157713
H	3.611846	0.747748	-0.429108
C	3.206254	-1.269735	-0.656118
H	3.073604	-1.251022	-1.742053
C	2.273202	-2.293553	-0.035583
H	2.455570	-2.392191	1.032064
H	1.234569	-2.017538	-0.192766
H	2.442618	-3.263354	-0.493778
C	4.663777	-1.542628	-0.319502
H	4.827672	-1.537718	0.756118
H	4.943860	-2.523827	-0.691054
H	5.325952	-0.814604	-0.783316
C	0.462124	0.301003	1.602171
H	1.438194	-0.103246	1.911356
C	0.321291	1.711837	2.177043
H	-0.628751	2.146050	1.876770
H	0.346565	1.667339	3.262361
H	1.119910	2.369225	1.839435
C	-0.589940	-0.622910	2.212358
H	-0.400508	-0.721205	3.278091
H	-1.588718	-0.220190	2.080528
H	-0.551766	-1.610141	1.761353
H	2.822491	0.078215	0.873144

44

int_S2_conf_8 Eopt -771.878862

C	1.612941	0.413330	-0.132346
C	1.869914	1.892964	-0.121788
C	0.866350	2.621165	-0.628023
C	-0.367710	1.964805	-0.968376
C	-0.685111	0.785957	-0.380672
H	0.930217	3.691981	-0.727328
H	-1.077723	2.467576	-1.600975
N	0.291053	0.103153	0.387732

H	2.785344	2.301792	0.269904
C	-1.991514	0.142470	-0.498226
C	-2.075761	-1.248975	-0.561771
C	-3.163769	0.894739	-0.580991
C	-3.301699	-1.870811	-0.701741
H	-1.169842	-1.835842	-0.529287
C	-4.387221	0.267724	-0.718119
H	-3.113082	1.970710	-0.511811
C	-4.460471	-1.115089	-0.776929
H	-3.354175	-2.947671	-0.756639
H	-5.288256	0.858748	-0.775064
H	-5.417447	-1.601903	-0.882829
H	1.579117	0.064494	-1.178009
N	2.655172	-0.387412	0.583466
H	3.124791	0.230686	1.254316
C	3.710169	-1.060777	-0.260401
H	4.394041	-1.532961	0.453895
C	4.472567	-0.026423	-1.070127
H	5.270193	-0.519308	-1.618186
H	3.831137	0.472876	-1.790476
H	4.927964	0.722357	-0.426540
C	3.078420	-2.142039	-1.120994
H	2.456325	-1.726894	-1.908580
H	3.864606	-2.721490	-1.596338
H	2.479990	-2.824893	-0.521940
C	0.100481	0.061026	1.853972
H	1.033548	0.404176	2.323167
C	-1.001830	0.991904	2.352631
H	-1.978225	0.659604	2.013416
H	-1.000065	0.996523	3.439345
H	-0.835291	2.008369	2.003827
C	-0.181346	-1.376828	2.299508
H	-0.177664	-1.435672	3.384437
H	-1.158824	-1.688671	1.943739
H	0.556285	-2.083205	1.915746
H	2.146996	-1.112433	1.110190

44

int_S2_conf_9 Eopt -771.874445

C	1.461272	0.741979	-0.665822
C	1.602658	2.238630	-0.728944
C	0.447508	2.876498	-0.953708
C	-0.767136	2.101194	-1.010728
C	-0.830276	0.883106	-0.427808
H	0.391571	3.946436	-1.064534
H	-1.637812	2.529915	-1.475932
N	0.359187	0.347187	0.193117

H	2.556985	2.724101	-0.613772
C	-2.048518	0.085060	-0.348056
C	-3.298251	0.692268	-0.213345
C	-1.983729	-1.306228	-0.447923
C	-4.447577	-0.073659	-0.179252
H	-3.363102	1.764452	-0.108408
C	-3.135894	-2.066794	-0.419147
H	-1.023403	-1.783980	-0.569016
C	-4.371155	-1.453553	-0.283236
H	-5.407186	0.406919	-0.067673
H	-3.072013	-3.140853	-0.504900
H	-5.270505	-2.048720	-0.257246
H	1.157605	0.387887	-1.668703
N	2.740980	0.038878	-0.344622
H	3.503856	0.599366	-0.740624
C	2.881218	-1.372728	-0.840909
H	2.479286	-1.396141	-1.858033
C	2.083228	-2.301759	0.056345
H	2.548240	-2.388949	1.036009
H	1.066327	-1.937449	0.174116
H	2.045521	-3.293811	-0.383211
C	4.360001	-1.726395	-0.863626
H	4.483653	-2.745305	-1.218538
H	4.917527	-1.075175	-1.533507
H	4.794385	-1.668385	0.132207
C	0.343973	0.726061	1.633805
H	-0.166720	1.695246	1.730174
C	-0.455981	-0.325873	2.401147
H	-1.465783	-0.408818	2.014273
H	0.021782	-1.299671	2.329719
H	-0.515714	-0.045096	3.448735
C	1.711738	0.886722	2.299311
H	2.321976	1.650435	1.819551
H	1.534738	1.230197	3.316765
H	2.239372	-0.064476	2.392660
H	2.839764	0.032251	0.681801

10

int_S3 Eopt -1264.020000

O	0.305756	-1.213428	-0.983276
S	-0.526121	-1.251380	0.214284
O	-0.050537	-0.379665	1.282313
O	-2.209699	0.375013	-0.507841
K	2.327158	0.712398	-0.097990
C	-2.007120	1.644393	0.116245
H	-2.814052	2.331271	-0.149350
H	-2.018595	1.471103	1.192170

H -1.043826 2.077148 -0.168537
H -2.243031 0.485270 -1.468068
13
ipr_amine Eopt -174.414507
N -0.001880 1.394560 -0.210361
H -0.819941 1.885023 0.090007
C 0.000022 0.043678 0.369306
H 0.000006 0.113716 1.437011
C -1.256375 -0.716057 -0.095314
H -1.239430 -1.709656 0.301389
H -2.130418 -0.207332 0.254182
H -1.272227 -0.759051 -1.164332
C 1.258433 -0.712599 -0.095503
H 1.087297 -1.766518 -0.025733
H 1.476022 -0.453013 -1.110476
H 2.086329 -0.443731 0.526750
H 0.813045 1.888518 0.092794
6
methanol Eopt -115.677615
C 0.666669 -0.020012 0.000000
O -0.747901 0.122966 0.000000
H 1.019993 -0.548681 0.892813
H 1.019993 -0.548682 -0.892812
H 1.087843 0.986612 -0.000001
H -1.144638 -0.752903 0.000000
31
protDBA Eopt -597.493560
N 0.084062 -1.306302 0.786309
H 0.709622 -2.051417 1.017564
C -1.275295 -1.841033 0.621653
H -1.590642 -2.301050 1.534801
H -1.280062 -2.567515 -0.163905
C 0.517297 -0.674317 -0.468204
H -0.152051 0.122957 -0.715646
H 0.512528 -1.400799 -1.253762
C -2.238656 -0.693555 0.265521
C -2.857440 -0.664910 -0.984582
C -2.492685 0.317047 1.192722
C -3.729520 0.374500 -1.307575
H -2.656364 -1.461387 -1.715633
C -3.365767 1.356318 0.870151
H -2.005209 0.294531 2.178110
C -3.984072 1.385275 -0.379808
H -4.216804 0.397481 -2.293132
H -3.566185 2.152777 1.601561
H -4.671759 2.204680 -0.634680
C 1.941386 -0.114125 -0.295708
C 3.041702 -0.967719 -0.380283
C 2.131245 1.246602 -0.055037
C 4.331513 -0.460727 -0.223536
H 2.891644 -2.040570 -0.569268
C 3.421437 1.754074 0.100801
H 1.264146 1.919540 0.011474
C 4.521511 0.900665 0.016731
H 5.198809 -1.133584 -0.289561
H 3.570849 2.827061 0.290142
H 5.538578 1.300190 0.140229
H 0.088518 -0.627346 1.520474

7.8. Effects of Potassium Metabisulfite with Zincke Imine Substitution Patterns

- The effects of potassium metabisulfite on the reaction outcome for forming pyridinium salts was examined using Zincke imines **1a** and **1m–1r** with amine **2a** and *iso*-propylamine (**2aw**).

Table S15. Effects of potassium metabisulfite with different Zincke imine substitution patterns using amines 2a and 2aw.[a]

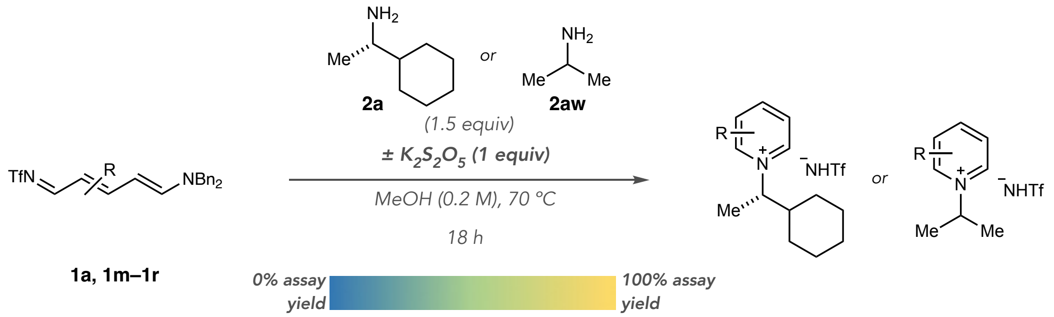

		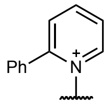	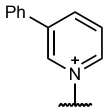	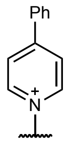	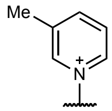	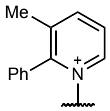	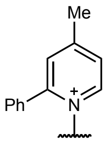	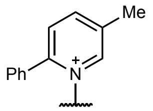
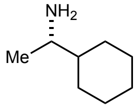	*with K2S2O5*	98%	100%	99%	86%	84%	73%	93%
	without K2S2O5	5%	98%	100%	50%	2%	71%	7%
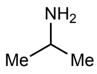	*with K2S2O5*	100%	101%	101%	96%	99%	82%	105%
	without K2S2O5	33%	100%	103%	80%	72%	82%	93%

[a]Reactions run using 0.1 mmol of **1a** and **1m–1r**. Yields determined by ^{1}H NMR using 1,3,5-trimethoxybenzene as an internal standard in CD_3OD.

7.9. Enantioenriched *N*-Alkylpyridinium and *N*-(Heteroaryl)pyridinium Salt Yield Improvements with Acid

- The alternative cyclization mechanism with bisulfite can improve pyridinium salt yields with lower-yielding and unreactive (heteroaryl)anilines from our previous report.[2] Notably, these examples initially are lower-yielding than previously reported with AcOH as the reaction additive. However, adding either H_2SO_4 or MsOH and heating the reaction generates the pyridinium products in higher yields, and, in other examples, enables the formation of previously inaccessible pyridinium salts. We suspect that (heteroaryl)aniline nucleophiles generate more stable derivatives of **4a** and/or **4b** with bisulfite, which require acid and heat to rearomatize to the pyridinium product.

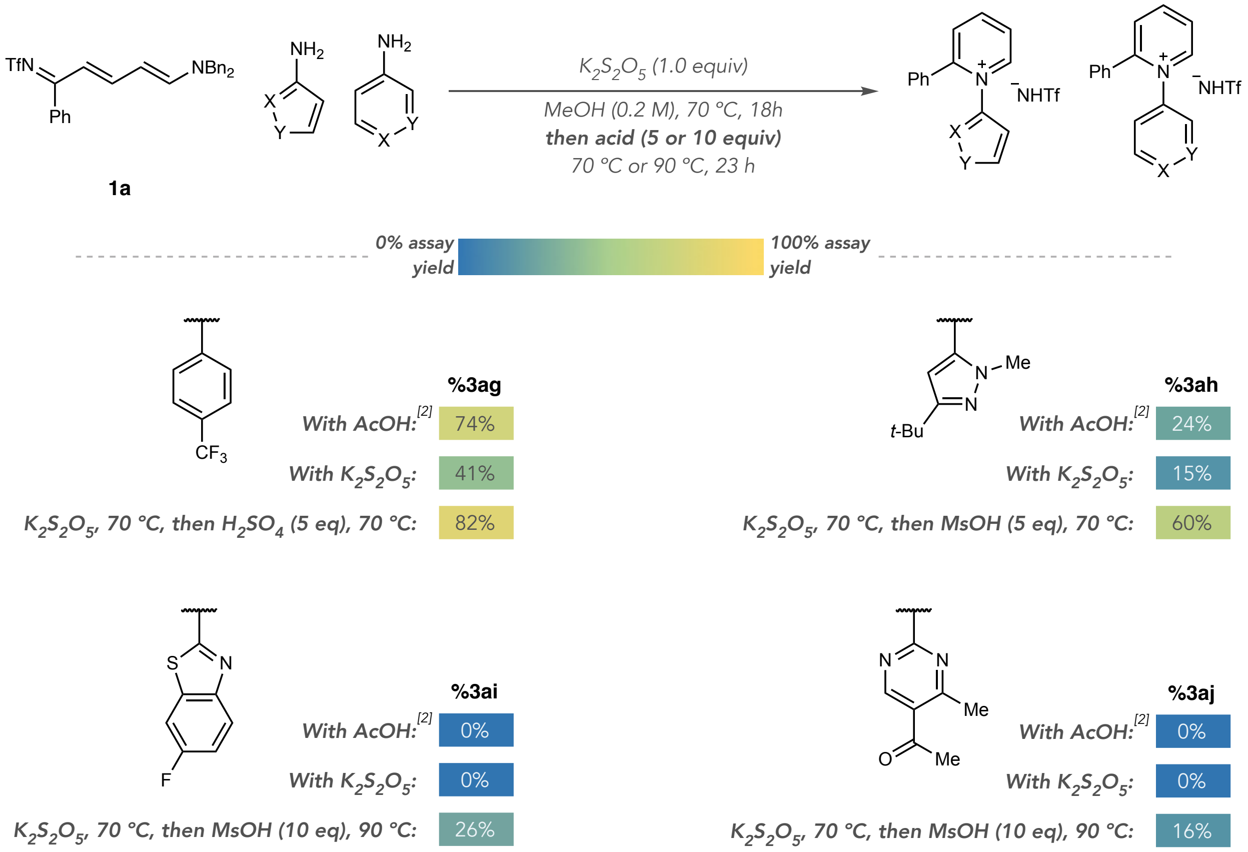

Figure S41. Reaction improvements for *N*-(hetero)arylpyridinium salts using potassium metabisulfite and subsequent acid mediated rearomatization. Yields determined by ^{1}H NMR using 1,3,5-trimethylbenzene as an internal standard in CD3OD. Yields with AcOH reported based on our previous report for *N*-(hetero)arylpyridinium salt formation.[2]

8. HTE for Enantioenriched *N*-Alkylpyridinium Salt Formation

8.1. Procedure and Assay for HTE Screens

General Procedure for HTE Screening of Zincke Imines and Enantioenriched (α-Chiral)amines and Assay

Stock solutions of Zincke imines (cores) **1a–1l** and amines (monomers) **2a–2av** were prepared in DCE and MeOH, respectively, as detailed in the Table S16. The solutions of cores (1 equiv) were manually pre-dosed into glass microvials in 96-position aluminum reaction plates. The solvent was then removed on a Genevac EZ-2 centrifugal evaporator. The reaction vials were manually dosed with solid, pulverized potassium metabisulfite (2 equiv) using a solid dosing pipet. The solutions of monomers (1.5 equiv) were prepared using a Tecan Fluent® liquid handler and solubilized by mixing in a Resodin™ LabRAM® II acoustic mixer for 2 hours at 15 G of force. The solutions or suspensions of monomers were then dosed into the reaction vials using a Tecan Fluent® liquid handler, giving a final reaction concentration of 0.2 M.

Table S16. Solution Concentrations and Volumes for Zincke Imine and Enantioenriched Amine Screening.

Reagent	Reaction (µmol)	Equivalents	Stock solution		
			Solvent	Concentration (M)	Amount Dispensed
Zincke Imine	7.5	1	DCE	0.2	37.5 µL
Enantioenriched Amine	11.25	1.5	MeOH	0.2	37.5 µL
Potassium Metabisulfite	15	2	neat	neat	3.3 mg

The reaction plate lid was secured with screws, and the plate was heated with a top and bottom heat plate, secured in a Resodyn™ LabRAM® II acoustic mixer, and mixed with 15 G of force at 70 ºC for 18 h. The reaction plates were cooled to room temperature, centrifuged for 5 minutes, and diluted to a final volume of 100 µL with DMSO for a concentration of 75 mM. The reaction plates were sealed again, shaken in the LabRAM® for 5 minutes at 15 G of force, and centrifuged again for 5 minutes. An analytical solution was then prepared by transferring 5 µL from each reaction vial into a 96-well analytical plate and further diluting to 750 µL with DMSO for a final concentration of 0.5 mM.

Each of the crude reactions was then analyzed by UPLC-MS-Charged Aerosol Detection (CAD) with Noscapine as an external calibrant for quantification. Eight Noscapine standard solutions in DMSO were used at the following concentrations (mg/mL): 1.0, 0.50, 0.25, 0.13, 0.063, 0.031, 0.016, 0.008, 0.004, 0.002, and 0.001. A calibration curve of CAD peak area of the Noscapine standards versus amount (µg) was plotted using a second order polynomial fit. The mass of target compound in the analytical samples was calculated using the calibration curve and then back extrapolated to quantify the amount in each well.[2,28]

Table S17. UPLC-CAD Assay Conditions for HTE Screening.

Column	Mobile Phase	Gradient	Column Temp.	Flow Rate Injection	UV Detection	CAD Settings
Cortecs UPLC C18 2.1x50 mm, 1.6 µg	A: 20 mM NH_4OAc in water<br>B: CH_3CN	5 min method<br><br>Initial: 95% A, 5% B<br>4.10 min: 100% B<br>4.60 min: 100% B<br>4.64 min: 95% A, 5% B	55 ºC	0.5 mL/min<br><br>Inj. 1 µL	210 nm, 215 nm, 254 nm	35 ºC evaporation temperature, 20 pA range, Power Function – 1.4

For the calculations of yield, it was assumed the pyridinium counterion is the acetate salt present from the UPLC mobile phase. CAD yields can vary from the ^{1}H NMR validation yields, and this is considered a contributing factor. Additional factors impacting yields include solvent evaporation for individual wells during the reaction as well as partial or no swapping of pyridinium counterions for individual products.

8.2. Results and Validations for HTE for Enantioenriched *N*-Alkylpyridinium Salt Formation Without Metabisulfite

- The general procedure for HTE screening was followed as described in Supporting Information Section 8.1, except potassium metabisulfite was not dosed into the reaction wells. The yields with and without metabisulfite are compared in the tables below.

Table S18. Results and ^{1}H NMR Validations for Zincke Imine 1a x 48 Enantioenriched (α-Chiral)amines (2a–2av) Screen.[a]

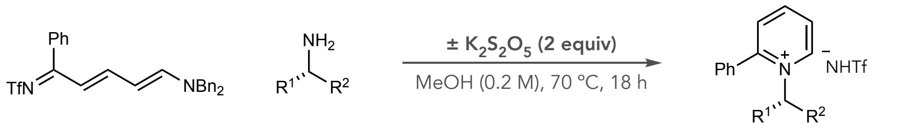

(7.5 µmol) (1.5 eq) Enantioenriched

1a **2a–2av** ***N*-Alkylpyridinium Salt**

	2a	2b	2c	2d	2e	2f	2g	2h	2i	2j	2k	2l	2m	2n	2o	2p	2q	2r	2s	2t	2u	2v	2w	2x
With metabisulfite	113	61	111	27	0	68	32	66	80	0	60	45	67	97	0	63	77	32	41	39	74	0	8	90
NMR (with metabisulfite)	100	74	87	58			70											30						
Without metabisulfite	0	0	0	0	0	0	0	0	7	0	0	2	0	0	0	0	2	5	0	0	1	0	0	6
NMR (without metabisulfite)	5	42	25	2			2											41						

	2y	2z	2aa	2ab	2ac	2ad	2ae	2af	2ag	2ah	2ai	2aj	2ak	2al	2am	2an	2ao	2ap	2aq	2ar	2as	2at	2au	2av
With metabisulfite	33	84	25	31	87	58	0	52	14	0	27	63	45	106	9	39	73	62	77	39	0	0	0	98
NMR (with metabisulfite)																	84		87					
Without metabisulfite	0	18	0	0	0	0	0	0	0	0	0	0	0	0	0	0	8	0	0	0	0	0	0	5
NMR (without metabisulfite)																	3		1					

[a]Quantitative ^{1}H NMR validations ran on 0.1 mmol scale using general procedure C with 1,3,5-trimethoxybenzene as an internal standard in CD_3OD. HTE reaction wells assayed with UPLC-MS-CAD as described in Section 8.1.

Table S19. Results and ^{1}H NMR Validations for Zincke Imine 1c x 48 Enantioenriched (α-Chiral)amines (2a–2av) Screen.[a]

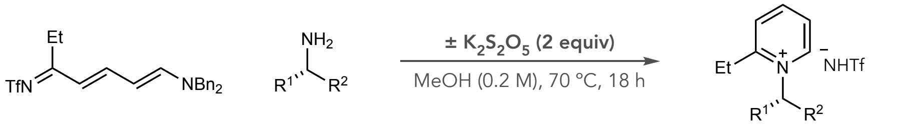

(7.5 µmol) (1.5 eq) Enantioenriched

1c **2a–2av** ***N*-Alkylpyridinium Salt**

	2a	2b	2c	2d	2e	2f	2g	2h	2i	2j	2k	2l	2m	2n	2o	2p	2q	2r	2s	2t	2u	2v	2w	2x
With metabisulfite	29	37	24	56	0	22	47	17	0	0	28	17	20	21	0	37	34	15	50	13	28	0	39	40
NMR (with metabisulfite)	33	36																						
Without metabisulfite	0	0	0	0	0	0	0	0	0	0	0	0	0	0	0	0	0	22	0	0	0	0	0	0
NMR (without metabisulfite)		42																						

	2y	2z	2aa	2ab	2ac	2ad	2ae	2af	2ag	2ah	2ai	2aj	2ak	2al	2am	2an	2ao	2ap	2aq	2ar	2as	2at	2au	2av
With metabisulfite	28	50	11	55	30	16	0	22	3	0	11	17	4	37	0	0	24	15	39	12	0	0	0	47
NMR (with metabisulfite)				36	55		0																	
Without metabisulfite	0	0	0	21	0	0	49	0	0	0	0	0	0	0	0	0	0	0	0	0	0	0	0	45
NMR (without metabisulfite)				22	2		29																	

[a]Quantitative ^{1}H NMR validations ran on 0.1 mmol scale using general procedure C with 1,3,5-trimethoxybenzene as an internal standard in CD_3OD. HTE reaction wells assayed with UPLC-MS-CAD as described in Section 8.1.

Table S20. Results and ^{1}H NMR Validations for Zincke Imine 1l x 48 Enantioenriched (α-Chiral)amines (2a–2av) Screen.[a]

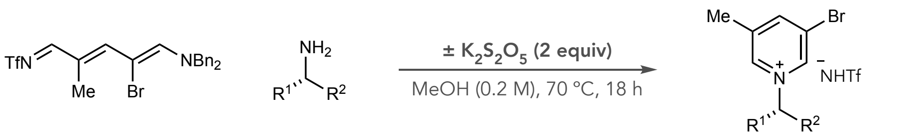

(7.5 µmol) **1m** | (1.5 eq) **2a–2av** | **Enantioenriched *N*-Alkylpyridinium Salt**

	2a	2b	2c	2d	2e	2f	2g	2h	2i	2j	2k	2l	2m	2n	2o	2p	2q	2r	2s	2t	2u	2v	2w	2x
With metabisulfite	28	61	0	38	0	12	30	58	21	0	51	55	71	56	0	0	74	22	54	29	27	0	31	67
NMR (with metabisulfite)	72	79	62																94					36
Without metabisulfite	51	95	63	28	0	19	19	81	28	0	72	37	82	75	0	0	49	70	95	17	0	0	54	96
NMR (without metabisulfite)	78	77	50																					

	2y	2z	2aa	2ab	2ac	2ad	2ae	2af	2ag	2ah	2ai	2aj	2ak	2al	2am	2an	2ao	2ap	2aq	2ar	2as	2at	2au	2av
With metabisulfite	5	75	7	58	58	53	3	42	16	0	16	11	55	53	0	12	0	3	58	0	0	0	0	46
NMR (with metabisulfite)				87	93		1																	
Without metabisulfite	20	88	0	94	117	89	26	86	14	0	31	76	105	95	5	28	0	10	94	51	0	0	30	30
NMR (without metabisulfite)				85	94		14																	

[a]Quantitative ^{1}H NMR validations ran on 0.1 mmol scale using general procedure C with 1,3,5-trimethoxybenzene as an internal standard in CD_3OD. HTE reaction wells assayed with UPLC-MS-CAD as described in Section 8.1.

Table S21. Results for 12 Zincke Imines (1a–1l) x Enantioenriched (α-Chiral)amine 2ao Screen.[a]

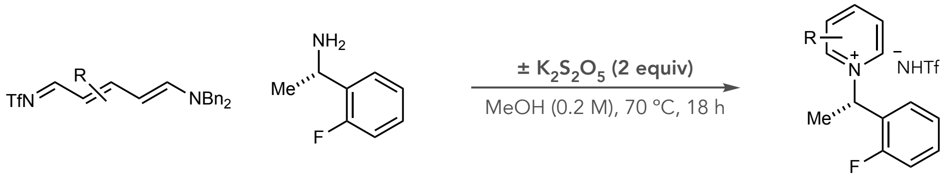

(7.5 µmol) **1a–1l** | (1.5 eq) **2ao** | **Enantioenriched *N*-Alkylpyridinium Salt**

	1a	1b	1c	1d	1e	1f	1g	1h	1i	1j	1k	1l
With metabisulfite	73	52	24	70	56	42	17	59	33	21	19	0
Without metabisulfite	8	8	0	0	89	10	0	0	0	29	51	0

[a]HTE reaction wells assayed with UPLC-MS-CAD as described in Section 8.1.

8.3. Results and Validations for 12 Zincke imines x 48 Enantioenriched (a-Chiral)amines Screen

Zincke Imine and Amine Scopes:

Table S22. Zincke imines and enantioenriched amines used in HTE Screening.

*N*Tf-Zincke imines 1a–1l

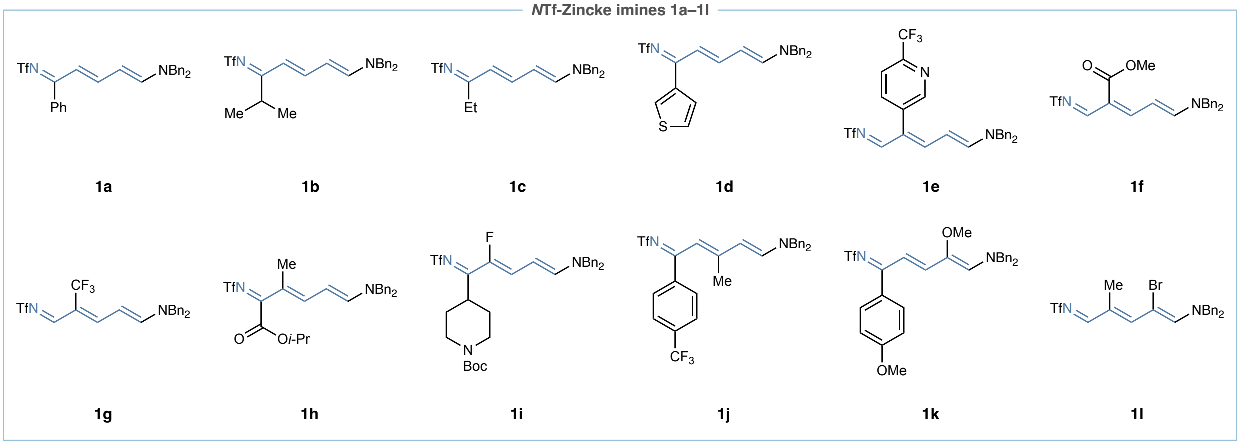

enantioenriched (α-chiral)amines 2a–2av

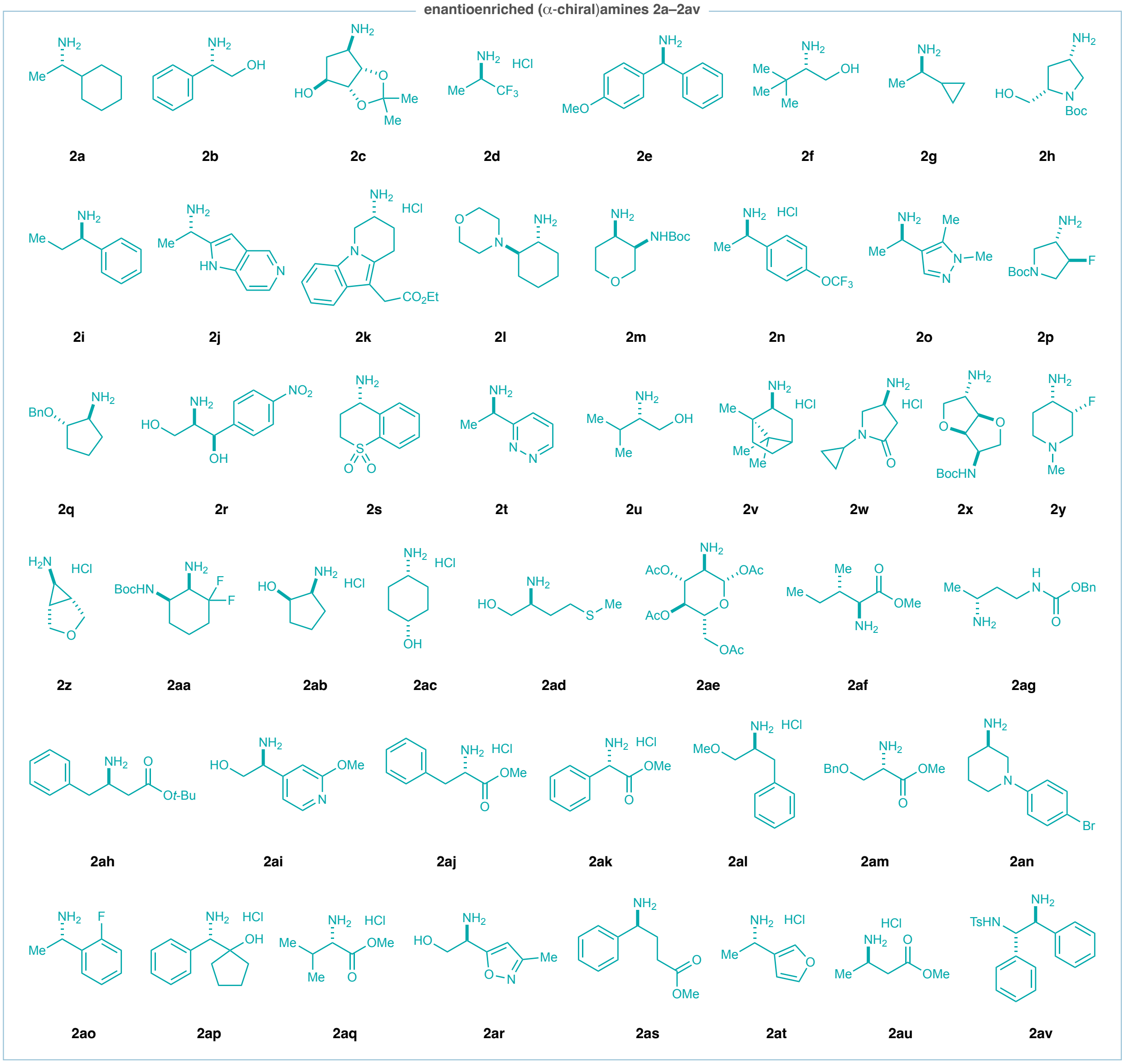

Results:

Table S23. Results from 12 Zincke Imines (1a–1l) x 48 Enantioenriched (α-Chiral)amines (2a–2av) Screen.

	1a	1b	1c	1d	1e	1f	1g	1h	1i	1j	1k	1l
2a	113	147	29	72	78	104	28	59	27	22	40	28
2b	61	87	37	78	88	62	34	0	48	67	44	61
2c	111	58	24	64	91	16	52	79	40	23	65	0
2d	27	14	56	39	78	86	53	32	10	23	28	38
2e	0	0	0	0	0	0	0	0	0	0	0	0
2f	68	28	22	75	91	71	40	23	28	76	63	12
2g	32	81	47	40	25	45	39	0	0	30	7	30
2h	66	67	17	49	102	23	22	45	30	10	81	58
2i	80	95	0	66	33	39	13	19	8	12	24	21
2j	0	0	0	0	0	0	0	0	0	0	29	0
2k	60	86	28	48	58	47	29	29	23	42	40	51
2l	45	48	17	77	74	128	78	42	8	28	72	55
2m	67	85	20	90	99	53	40	54	28	17	97	71
2n	97	61	21	57	77	34	37	52	12	30	41	56
2o	0	0	0	0	0	0	0	0	0	0	0	0
2p	63	44	37	49	42	35	7	0	9	46	0	0
2q	77	102	34	47	89	51	34	105	47	39	86	74
2r	32	10	15	25	31	45	19	0	6	24	25	22
2s	41	27	50	20	61	50	13	8	11	0	19	54
2t	39	67	13	50	75	51	46	16	0	21	30	29
2u	74	94	28	58	86	68	29	5	49	53	76	27
2v	0	0	0	0	106	50	8	0	0	0	0	0
2w	8	26	39	31	20	16	12	5	0	19	11	31
2x	90	114	40	75	73	75	19	40	27	15	61	67
2y	33	39	28	37	27	19	34	28	13	38	0	5
2z	84	25	50	64	86	142	56	88	40	69	119	75
2aa	25	32	11	21	6	80	22	26	21	6	10	7
2ab	31	46	55	58	75	77	51	16	11	63	102	58
2ac	87	132	30	104	99	71	87	80	11	64	46	58
2ad	58	101	16	62	91	69	27	0	37	46	48	53
2ae	0	0	0	0	0	3	0	0	0	0	0	3
2af	52	60	22	73	102	75	45	81	19	24	51	42
2ag	14	18	3	22	10	15	10	9	1	15	10	16
2ah	0	0	0	0	0	0	0	0	0	0	0	0
2ai	27	24	11	22	0	0	10	0	8	14	7	16
2aj	63	54	17	44	71	44	28	64	17	24	40	11
2ak	45	70	4	18	70	58	36	31	20	27	35	55
2al	106	85	37	112	88	57	40	83	16	43	28	53
2am	9	0	0	0	0	0	0	6	9	0	0	0
2an	39	21	0	21	26	13	27	32	16	21	20	12
2ao	73	52	24	70	56	42	17	59	33	21	19	0
2ap	62	19	15	24	67	44	26	68	18	41	75	3
2aq	77	82	39	45	98	142	36	57	15	30	26	58
2ar	39	21	12	51	0	7	0	14	10	16	17	0
2as	0	0	0	0	0	0	0	0	0	0	0	0
2at	0	0	0	0	0	0	0	0	0	0	0	0
2au	0	11	0	0	0	0	0	0	0	0	0	0
2av	98	97	47	6	54	43	10	64	51	17	50	46

0% assay yield — *100% assay yield*

Quantitative ^{1}H NMR Validations:

Table S24. Results from 12 Zincke Imines (1a–1l) x 48 Enantioenriched (α-Chiral)amines (2a–2av) Screen.[a]

	1a	NMR Valid.	1b	NMR Valid.	1c	NMR Valid.	1d	NMR Valid.	1e	NMR Valid.	1f	NMR Valid.	1g	NMR Valid.	1h	NMR Valid.	1i	NMR Valid.	1j	NMR Valid.	1k	NMR Valid.	1l	NMR Valid.
2a	113	100	147		29	33	72	100	78		104	67	28		59	69	27	30	22	52	40		28	72
2b	61	74	87	41	37	36	78		88	102	62		34		0		48		67		44		61	79
2c	111	87	58	58	24		64		91		16		52		79		40		23		65		0	62
2d	27	58	14	20	56		39		78	102	86		53		32		10		23		28		38	
2e	0	0	0		0		0		0		0		0		0		0		0		0		0	
2f	68		28		22		75		91		71		40		23		28		76		63		12	
2g	32	70	81		47		40		25		45		39		0		0		30		7		30	
2h	66		67		17		49		102		23		22		45		30		10		81		58	
2i	80	96	95		0	25	66		33		39	46	13	34	19		8		12		24	71	21	
2j	0		0		0		0		0		0		0		0		0		0		29		0	
2k	60		86		28		48		58		47		29		29		23		42		40		51	
2l	45		48		17		77		74		128		78		42		8		28		72		55	
2m	67		85		20		90		99		53		40		54		28		17		97		71	
2n	97		61		21		57		77		34		37		52		12		30		41		56	
2o	0	3	0		0		0		0		0		0		0		0		0		0		0	
2p	63		44		37		49		42		35		7		0		9		46		0		0	
2q	77		102		34		47		89		51		34		105		47		39		86		74	
2r	32	30	10		15		25		31		45		19		0		6		24		25		22	
2s	41		27		50		20		61		50		13	61	8		11		0		19	0	54	94
2t	39		67		13		50		75		51		46		16		0		21		30		29	
2u	74		94		28		58		86		68		29		5		49		53		76		27	
2v	0		0		0		0		106	91	50		8	15	0		0		0		0	0	0	
2w	8		26		39		31		20		16		12		5		0		19		11		31	
2x	90		114		40		75		73		75		19		40		27		15		61	92	67	36
2y	33		39		28		37		27		19		34		28		13		38		0		5	
2z	84		25		50		64		86		142		56		88		40		69		119		75	
2aa	25	3	32		11		21		6		80		22		26		21		6		10		7	
2ab	31		46		55	36	58		75	98	77		51		16		11		63		102		58	87
2ac	87		132		30	55	104		99		71		87		80		11		64		46		58	93
2ad	58		101	62	16		62		91		69		27		0		37		46		48		53	
2ae	0		0	0	0	0	0		0		3		0		0		0		0		0		3	1
2af	52	18	60		22		73		102		75		45		81		19		24		51		42	
2ag	14	13	18		3		22		10		15		10		9		1		15		10		16	
2ah	0		0		0		0		0		0		0		0		0		0		0		0	
2ai	27		24		11		22		0		0		10		0		8		14		7		16	
2aj	63		54		17		44		71		44		28		64		17		24		40		11	
2ak	45		70		4		18		70		58		36		31		20		27		35		55	
2al	106		85		37		112		88		57		40		83		16		43		28		53	
2am	9	59	0		0		0		0		0		0		6		9		0		0		0	
2an	39		21		0		21		26		13		27		32		16		21		20		12	
2ao	73	84	52		24		70		56		42		17		59		33		21		19		0	
2ap	62		19		15		24		67		44		26		68		18		41		75		3	
2aq	77	87	82		39		45		98		142		36		57		15		30		26		58	
2ar	39		21		12		51		0		7		0		14		10		16		17		0	
2as	0		0		0		0		0		0		0		0		0		0		0		0	
2at	0		0		0		0		0		0		0		0		0		0		0		0	
2au	0		11		0		0		0		0		0		0		0		0		0		0	
2av	98		97		47		6		54		43		10		64		51		17		50		46	

[a]Quantitative ^{1}H NMR validations ran on 0.1 mmol scale using general procedure C with $K_2S_2O_5$ (2 equiv) with 1,3,5-trimethoxybenzene as an internal standard in CD_3OD.

8.4. Validation of Amine Stereoretention

- We validated the amine stereoretention in the pyridinium product using Zincke imine **1s** that contains a distal reporter stereocenter. After cyclization, the crude reaction mixtures were analyzed with ^{1}H NMR to determine the diastereomeric ratio of the product (Scheme S6). A representative ^{1}H NMR spectrum for this analysis is provided below in Figure S42 (see Supporting Information Section 11 for full list of spectra).

Scheme S6. Validation of amine stereoretention under the reaction conditions for pyridinium formation.[a]

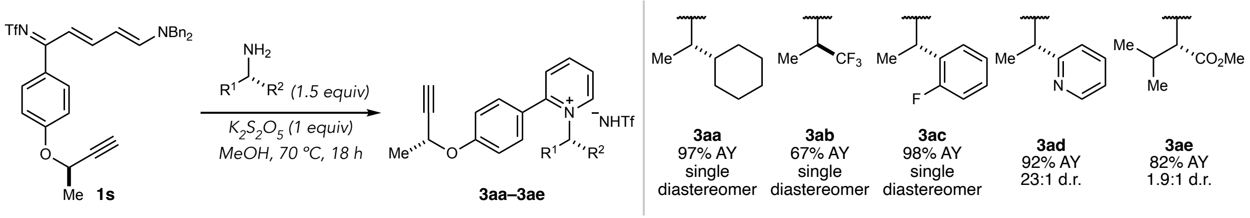

[a]Yields determined by ^{1}H NMR using 1,3,5-trimethoxybenzene as an internal standard in CD_3OD. Diastereomeric ratio determined by ^{1}H NMR analysis of the crude reaction mixture.

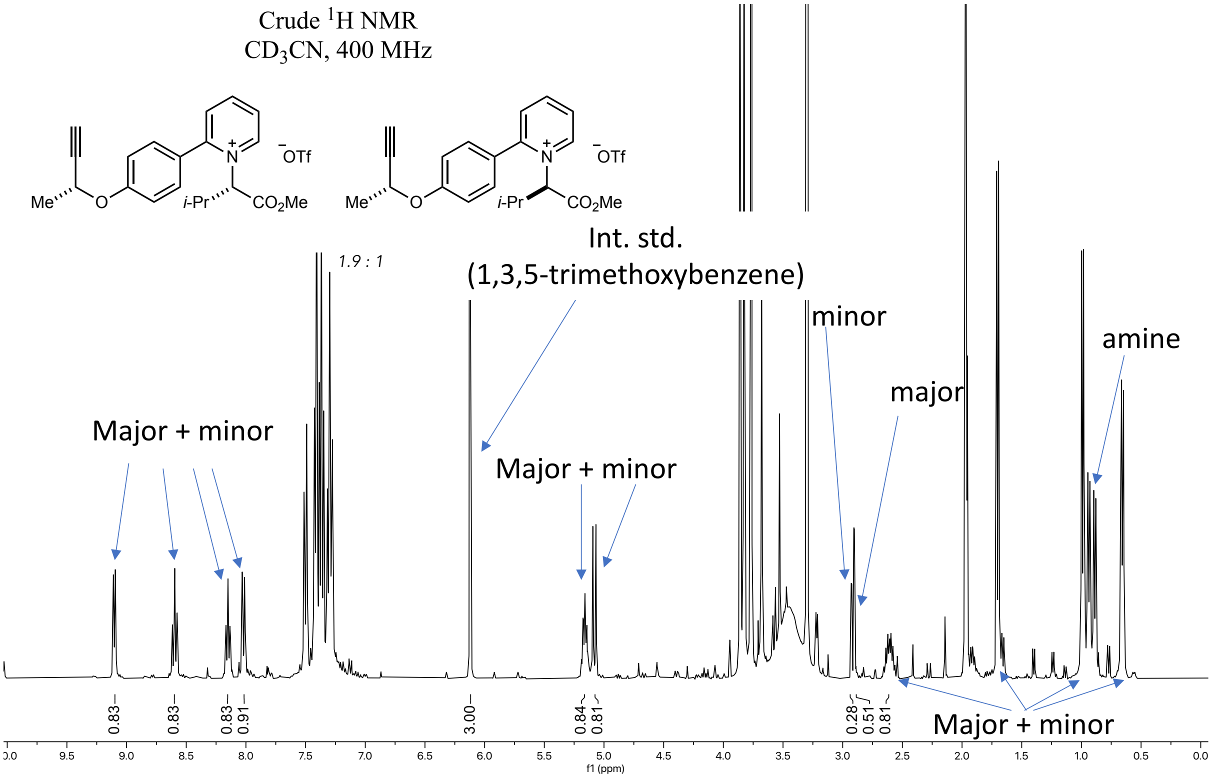

Figure S42. Representative crude ^{1}H NMR (in CD_3CN) of the reaction between Zincke imine **1s** and amine **2aq** with metabisulfite (1 equiv) at 70 ºC where diastereomers were observed.

8.5. Limitations and Yield Improvements for Enantioenriched Pyridinium Salt Formation

<u>Problematic Substrates and Limitations:</u>

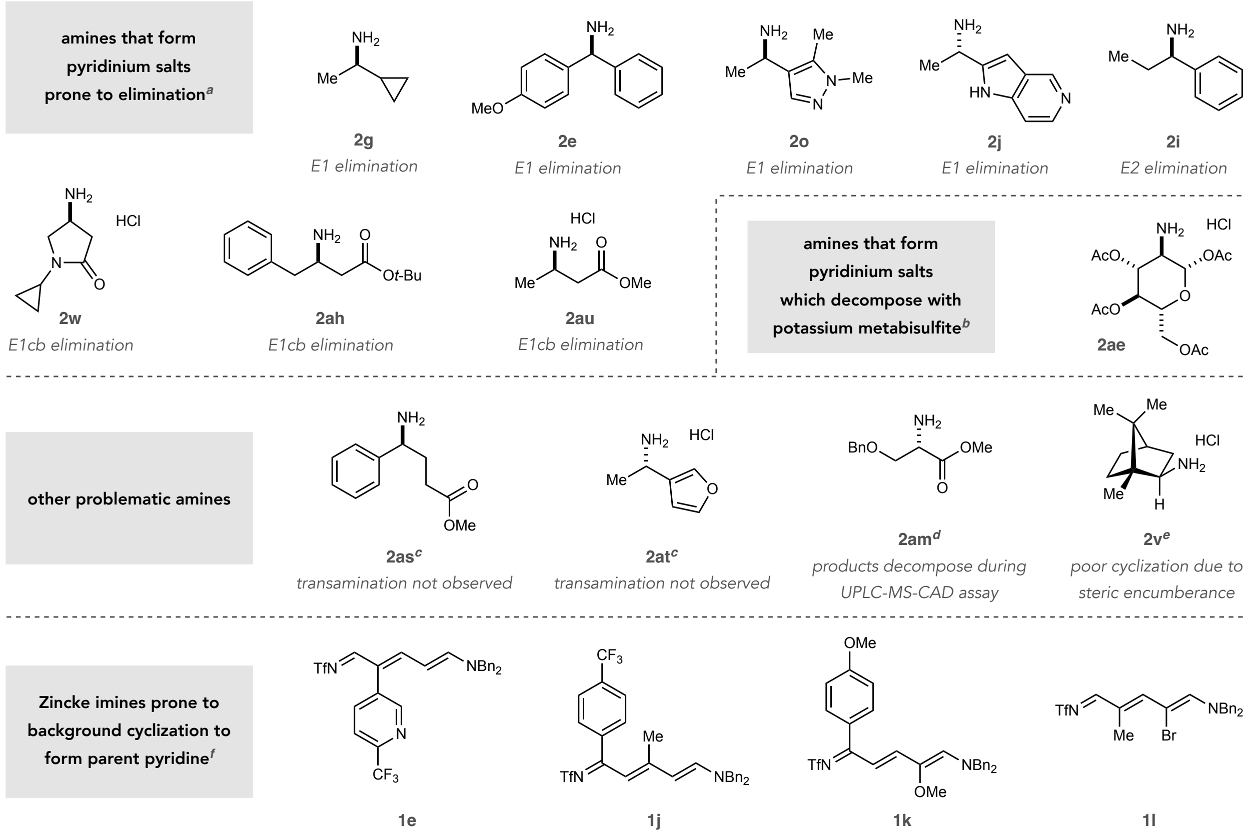

Figure S43. Problematic amines and Zincke imines for enantioenriched *N*-alkylpyridinium salt formation and associated limitations. [a]Parent pyridine and elimination byproducts observed by UPLC-MS-CAD, and/or LRMS and ^{1}H NMR. [b]Many unknown byproducts observed by UPLC-MS-CAD, and/or LRMS and ^{1}H NMR. [c]Unreacted Zincke imines observed in reaction wells by UPLC-MS-CAD. [d]Decomposition byproducts observed and desired pyridinium salt not observed by UPLC-MS-CAD and LRMS, but desired products are observed by ^{1}H NMR. [e]Transaminated-Zincke imines observed in reaction wells by UPLC-MS-CAD, and/or LRMS and ^{1}H NMR; **2v** only cyclizes with mono-3-substituted Zincke imines. [f]Parent pyridine observed with desired product by UPLC-MS-CAD, LRMS, and ^{1}H NMR; elimination byproducts were not observed unless amines prone to elimination pathways were used.

<u>Additional Discussion of HTE Screen Outcomes:</u>

- Although the bulk of the amines were successful in pyridinium formation, **2e**, **2j**, **2o**, **2v**, **2ae**, **2ag**, **2ah**, **2am**, **2as**, **2at**, and **2au** formed < 20% product with most of the Zincke imines tested. Our analysis of the reaction wells for amines **2e**, **2j**, **2o**, **2ah**, and **2au** revealed that the parent pyridine and elimination byproducts were present, indicating the pyridinium products were susceptible to decomposition under the reaction conditions. We also observed product elimination with **2g**, **2i**, and **2w**; however, these amines still formed ≥ 20% pyridinium product in most cases. The reaction wells using **2v**, **2as**, and **2at** contained unreacted Zincke imine. We suspect the steric hindrance around the nitrogen atom in **2v** inhibits the transamination-cyclization process, and **2as** was largely insoluble MeOH. Additionally, no products formed with **2ae**, potentially due to product instability under the reaction conditions.

- Although the UPLC-CAD assay provided reasonable estimates of reaction outcomes, *O*-Benzyl serine ester **2am** is an exception that was unsuccessful in Table S22 yet forms pyridinium products detected by ^{1}H NMR. We suspect the products from **2am** are unstable during the UPLC-CAD analysis.
- Disubstituted Zincke imines **1h–1l** performed well in pyridinium salt formation, although we detected the parent pyridine for Zincke imines **1j** and **1l** in the reaction wells. While pyridinium formation is usually favored, we suspect that the steric repulsion between the C2- and C4-substituents in **1j**, or C3- and C5-substituents in **1l**, promotes an unwanted background recyclization pathway.

Pyridinium Salt Yield Improvements at Lower Temperatures:

- Amines that form products prone to elimination can generally be employed at lower temperatures to minimize pyridinium salt decomposition and improve yields of the desired product. However, pyridinium salts derived from amines **2e** and **2o** are unstable at room temperature and readily decompose to the corresponding pyridine and elimination byproduct. Additionally, Zincke imines that are prone to pyridine formation can be run at lower temperatures to minimize background recyclization.

Scheme S7. Improvement of the reaction between 1a and 2g at lower temperatures to minimize pyridinium salt E1 decomposition.[a]

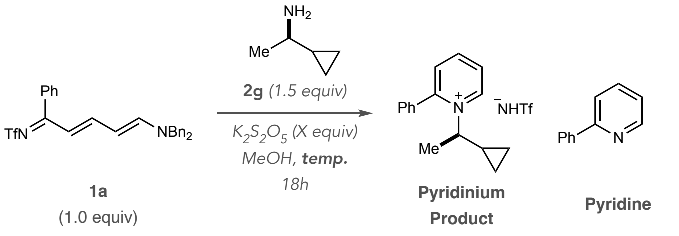

Scale	$K_2S_2O_5$ equiv	Temp.	Pyridine	Zincke Imine	Pyridinium Product
7.5 µmol	2.0	70 °C	detected	nd	32%
0.1 mmol	2.0	70 °C	30%	nd	67%
0.1 mmol	1.0	50 °C	7%	nd	82%

[a]Reactions on 7.5 µmol scale assayed with UPLC-MS-CAD. Reactions on 0.1 mmol scale assayed with quantitative ^{1}H NMR using 1,3,5-trimethoxybenzene as an internal standard in CD_3OD and validated with LRMS.

Scheme S8. Improvement of the reaction between 1a and 2au at lower temperatures to minimize pyridinium salt E1$_{cb}$ decomposition.[a]

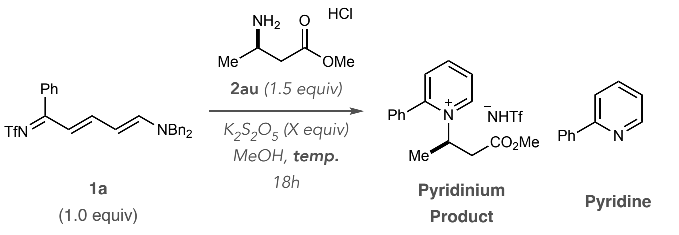

Scale	$K_2S_2O_5$ equiv	Temp.	Pyridine	Zincke Imine	Pyridinium Product
7.5 µmol	2.0	70 °C	detected	nd	nd
0.1 mmol	2.0	70 °C	85%	nd	nd
0.1 mmol	1.0	50 °C	44%	nd	41%
0.1 mmol	1.0	rt	6%	32%	45%

[a]Reactions on 7.5 µmol scale assayed with UPLC-MS-CAD. Reactions on 0.1 mmol scale assayed with quantitative ^{1}H NMR using 1,3,5-trimethoxybenzene as an internal standard in CD_3OD and validated with LRMS.

Scheme S9. Improvement of the reaction between 1g and 2i at lower temperatures to minimize pyridinium salt E2 decomposition.[a]

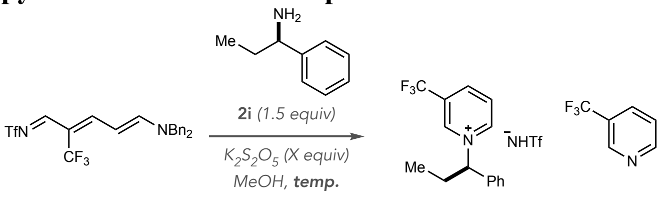

1g
(1.0 equiv)

Scale	$K_2S_2O_5$ equiv	Temp.	Pyridine	Zincke Imine	Pyridinium Product
7.5 µmol	2.0	70 °C	detected	nd	13%
0.1 mmol	2.0	70 °C	27%	nd	34%
0.1 mmol	1.0	50 °C	nd	nd	73%

[a]Reactions on 7.5 µmol scale assayed with UPLC-MS-CAD. Reactions on 0.1 mmol scale assayed with quantitative ^{1}H NMR using 1,3,5-trimethoxybenzene as an internal standard in CD_3OD and validated with LRMS.

Scheme S10. Improvement of the reaction between 1j and 2a at lower temperatures to minimize background recyclization of the Zincke imine.[a]

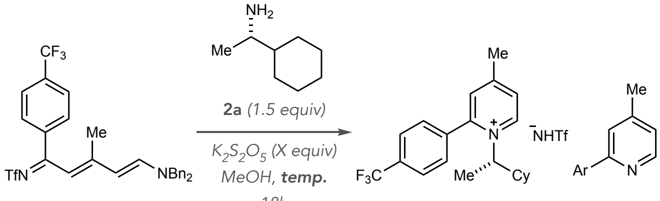

1j
(1.0 equiv)

Scale	$K_2S_2O_5$ equiv	Temp.	Pyridine	Zincke Imine	Pyridinium Product
7.5 µmol	2.0	70 °C	detected	nd	22%
0.1 mmol	2.0	70 °C	10%	nd	41%
0.1 mmol	none	50 °C	nd	nd	52%

[a]Reactions on 7.5 µmol scale assayed with UPLC-MS-CAD. Reactions on 0.1 mmol scale assayed with quantitative ^{1}H NMR using 1,3,5-trimethoxybenzene as an internal standard in CD_3OD and validated with LRMS.

Scheme S11. Limitations for pyridinium formation from 1a with amines 2e and 2o.[a]

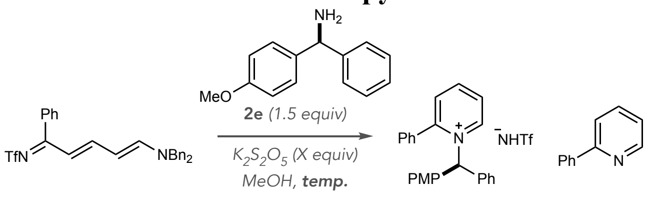

1a
(1.0 equiv)

Scale	$K_2S_2O_5$ equiv	Temp.	Pyridine	Zincke Imine	Pyridinium Product
7.5 µmol	2.0	70 °C	detected	nd	nd
0.1 mmol	2.0	70 °C	94%	nd	nd
0.1 mmol	1.0	50 °C	88%	nd	trace
0.1 mmol	1.0	rt	22%	73%	nd

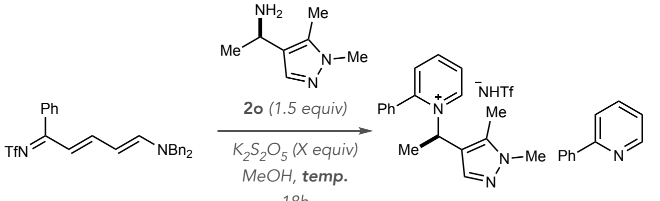

1a
(1.0 equiv)

Scale	$K_2S_2O_5$ equiv	Temp.	Pyridine	Zincke Imine	Pyridinium Product
7.5 µmol	2.0	70 °C	detected	nd	nd
0.1 mmol	2.0	70 °C	62%	nd	trace
0.1 mmol	1.0	50 °C	28%	45%	nd
0.1 mmol	1.0	rt	nd	85%	nd

[a]Reactions on 7.5 µmol scale assayed with UPLC-MS-CAD. Reactions on 0.1 mmol scale assayed with quantitative ^{1}H NMR using 1,3,5-trimethoxybenzene as an internal standard in CD_3OD and validated with LRMS.

Improvements for Pyridinium Salts that Decompose with Metabisulfite:

- Some products decompose under the reaction conditions with potassium metabisulfite. In these cases, metabisulfite is excluded from the reaction conditions to minimize product decomposition.

Scheme S12. Improvements for pyridinium formation with amine 2ae and Zincke imines 1c and 1l without potassium metabisulfite.[a]

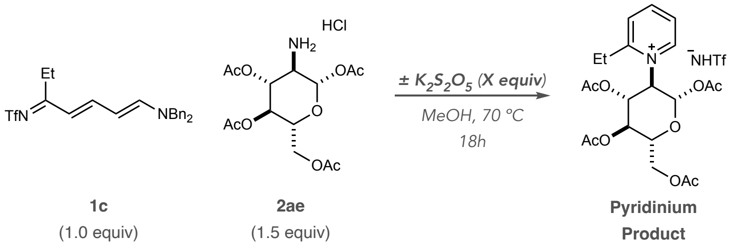

Scale	$K_2S_2O_5$ equiv	Pyridine	Zincke Imine	Pyridinium Product
7.5 µmol	2.0	nd	nd	nd
0.1 mmol	2.0	nd	nd	nd
7.5 µmol	none	nd	nd	49%
0.1 mmol	none	nd	nd	29%

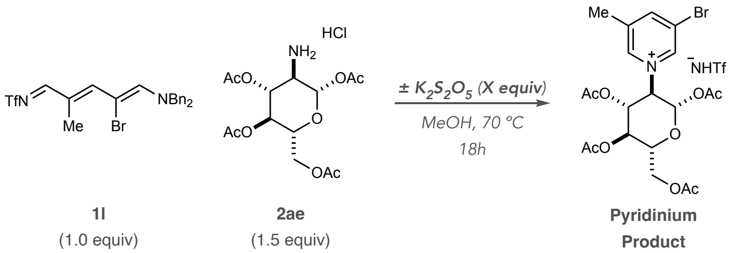

Scale	$K_2S_2O_5$ equiv	Pyridine	Zincke Imine	Pyridinium Product
7.5 µmol	2.0	detected	nd	3%
0.1 mmol	2.0	23%	nd	1%
7.5 µmol	none	detected	nd	26%
0.1 mmol	none	51%	nd	14%

[a]Reactions on 7.5 µmol scale assayed with UPLC-MS-CAD. Reactions on 0.1 mmol scale assayed with quantitative ^{1}H NMR using 1,3,5-trimethoxybenzene as an internal standard in CD_3OD and validated with LRMS.

Improvements for Pyridinium Salts with Acid After Cyclization:

- Electron-deficient amines, amino alcohols and diamines are susceptible to forming bisulfite addition adducts and/or cyclized products. These byproducts are converted to pyridinium salts with additional acid after cyclization (see Supporting Information Section 7.6. Table S14). Reactions were set up according to general procedure C; after cooling to room temperature, MsOH or H_2SO_4 (5 equiv) was added to the reaction and stirred at room temperature or 70 ºC for 18 hours.

Scheme S13. Improvement of pyridinium formation using electron-deficient amine 2d and Zincke imine 1a with acid after cyclization.[a]

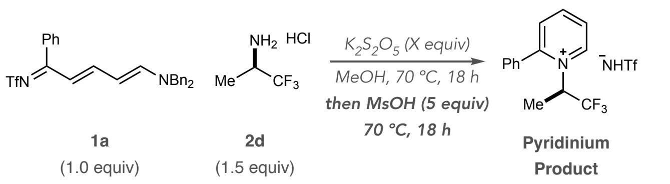

Scale	$K_2S_2O_5$ equiv	MsOH equiv	Pyridine	Zincke Imine	Pyridinium Product
7.5 µmol	2.0	none	nd	nd	27%
0.1 mmol	1.0	none	nd	nd	68%
0.1 mmol	1.0	5.0	nd	nd	92%

[a]Reactions on 7.5 µmol scale assayed with UPLC-MS-CAD. Reactions on 0.1 mmol scale assayed with quantitative ^{1}H NMR using 1,3,5-trimethoxybenzene as an internal standard in CD_3OD and validated with LRMS.

Scheme S14. Improvement of pyridinium formation using amino alcohols 2ab & 2r and Zincke imine 1a with acid after cyclization.[a]

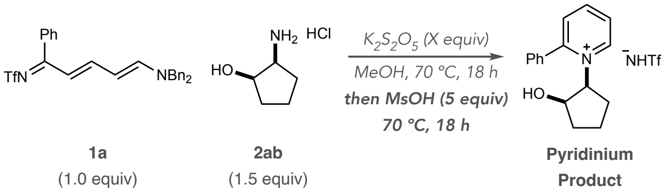

Scale	$K_2S_2O_5$ equiv	MsOH equiv	Pyridine	Zincke Imine	Pyridinium Product
7.5 µmol	2.0	none	nd	nd	31%
0.1 mmol	1.0	none	nd	nd	56%
0.1 mmol	1.0	5.0	nd	nd	68%

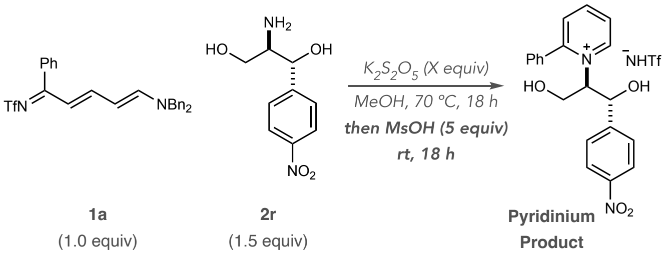

Scale	$K_2S_2O_5$ equiv	MsOH equiv	Pyridine	Zincke Imine	Pyridinium Product
7.5 µmol	2.0	none	nd	nd	32%
0.1 mmol	1.0	none	nd	nd	44%
0.1 mmol	1.0	5.0	nd	nd	98%

[a]Reactions on 7.5 µmol scale assayed with UPLC-MS-CAD. Reactions on 0.1 mmol scale assayed with quantitative ^{1}H NMR using 1,3,5-trimethoxybenzene as an internal standard in CD_3OD and validated with LRMS.

Scheme S15. Improvement of pyridinium formation using diamine 2aa and Zincke imine 1a with acid after cyclization.[a]

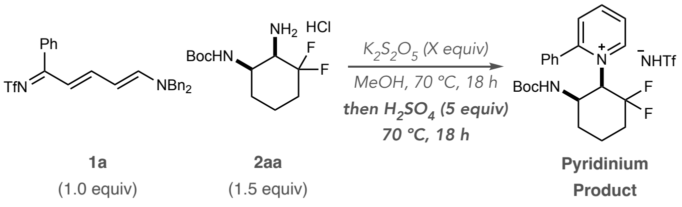

Scale	$K_2S_2O_5$ equiv	H_2SO_4 equiv	Pyridine	Zincke Imine	Pyridinium Product
7.5 µmol	2.0	none	nd	nd	25%
0.1 mmol	1.0	none	nd	nd	3%
0.1 mmol	1.0	5.0	nd	nd	62%[b]

[a]Reactions on 7.5 µmol scale assayed with UPLC-MS-CAD. Reactions on 0.1 mmol scale assayed with quantitative ^{1}H NMR using 1,3,5-trimethoxybenzene as an internal standard in CD_3OD and validated with LRMS. [b]Deprotection of Boc group observed.

Improved Pyridinium Salt Yields with the Amine as the Limiting Reagent:

- The amine can be used as the limiting reagent for pyridinium formation with C2-alkyl substituted Zincke imines for increased yields.

Scheme S16. Improvement of pyridinium formation using 2a as the limiting reagent with Zincke imine 1c.[a]

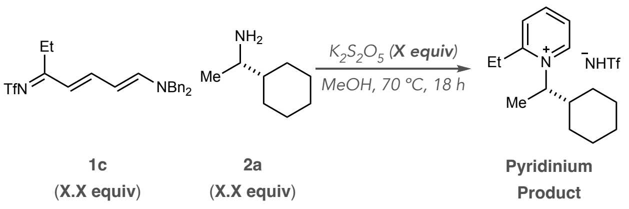

Scale	1c equiv	2a equiv	$K_2S_2O_5$ equiv	Pyridine	Zincke Imine	Pyridinium Product
7.5 µmol	1.0	1.5	2.0	nd	nd	29%
0.1 mmol	1.0	1.5	1.0	nd	nd	33%
0.1 mmol	1.5	1.0	1.0	nd	nd	71%

[a]Reactions on 7.5 µmol scale assayed with UPLC-MS-CAD. Reactions on 0.1 mmol scale assayed with quantitative ^{1}H NMR using 1,3,5-trimethoxybenzene as an internal standard in CD_3OD and validated with LRMS.

9. Synthetic Applications

9.1. Gram-Scale Enantioenriched *N*-Alkylpyridinium Salt Synthesis

(*S*)-1-(1-Cyclohexylethyl)-2-phenylpyridin-1-ium trifluoromethanesulfonate (3a)

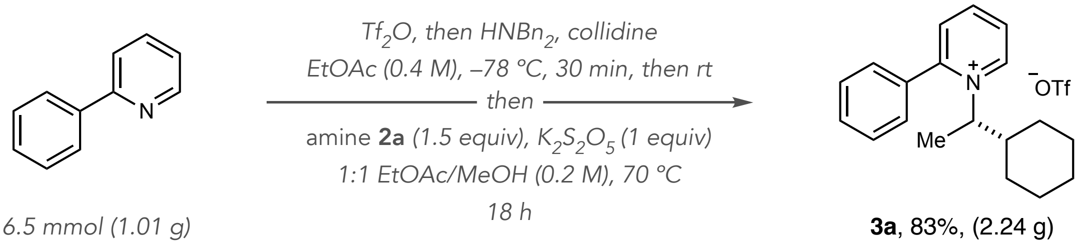

Prepared according to general procedure B using 2-phenylpyridine (950 µL, 6.50 mmol), EtOAc (16.3 mL, 0.4 M), Tf_2O (1.09 mL, 6.50 mmol), collidine (860 µL, 6.50 mmol), dibenzylamine (1.50 mL, 7.80 mmol), MeOH (16.3 mL, one reaction volume), potassium metabisulfite (1.44 g, 6.50 mmol), (*S*)-1-cyclohexylethan-1-amine (**2a**) (1.43 mL, 9.75 mmol), and heated to 70 ºC for 18 h. Isolated according to general isolation procedure B1 using NaOTf (5.59 g, 32.5 mmol), CH_2Cl_2 (80 mL), 1.0 M AcOH wash (1 x 50 mL), H_2O washes (2 x 50 mL), sat. Na_2CO_3 wash (1 x 50 mL), and 2:1 hexanes/Et_2O (400 mL) to afford the title compound as a light-yellow solid (2.24 g, 5.39 mmol, 83% yield). Isolated **3a** was weighed into three 8 mL vials (41.5 mg, 0.1 mmol) and mesitylene was added (13.9 µL, 0.1 mmol) for ^{1}H NMR weight percent measurement in CD_3OD, shown in Figure S44 ((100 + 98 + 99)/3 = 99% pure by weight). See section 6.1 for **3a** characterization data.

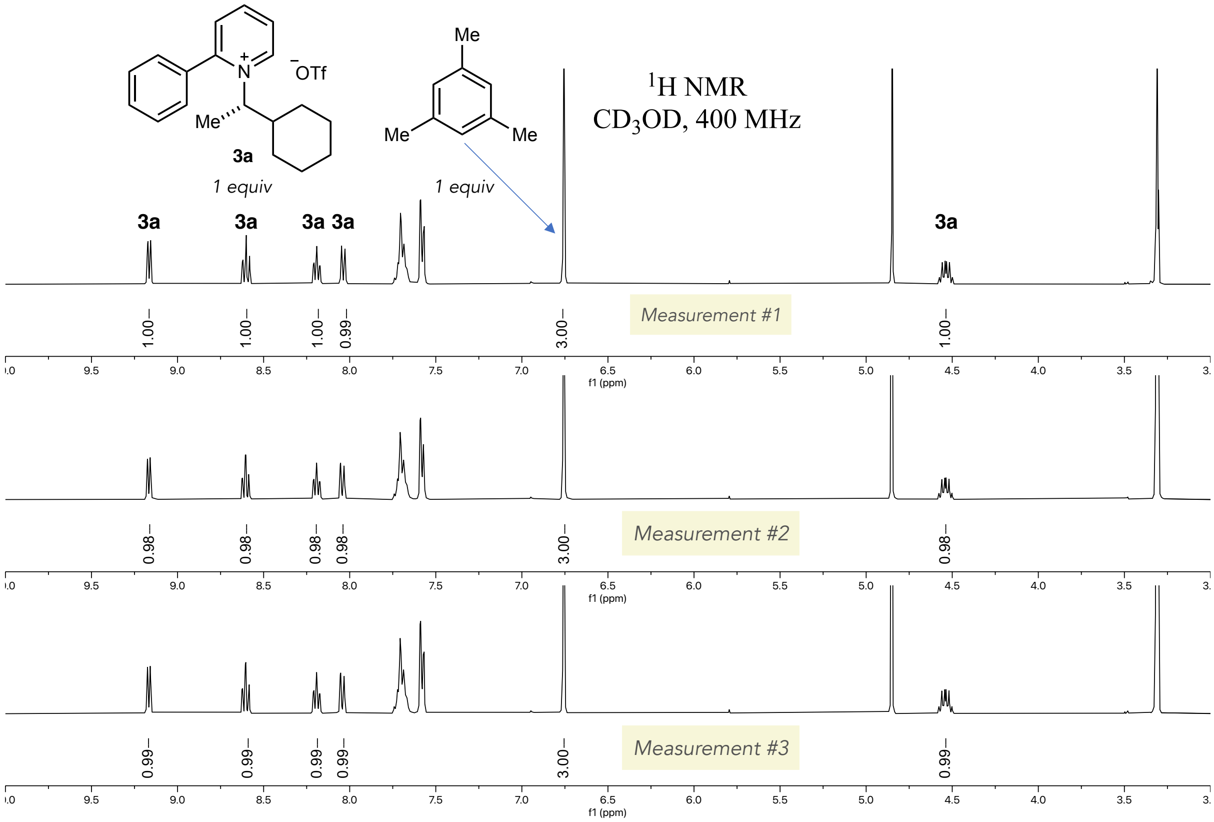

Figure S44. ^{1}H NMR (CD_3OD) weight percent measurements of **3a** using mesitylene (1 equiv) from gram-scale synthesis.

9.2. Convergent Coupling of Etoricoxib and Linagliptin

(*R*)-1'-(1-(7-(But-2-yn-1-yl)-3-methyl-1-((4-methylquinazolin-2-yl)methyl)-2,6-dioxo-2,3,6,7-tetrahydro-1*H*-purin-8-yl)piperidin-3-yl)-5-chloro-6'-methyl-3-(4-(methylsulfonyl)phenyl)-[2,3'-bipyridin]-1'-ium trifluoromethanesulfonate (3af)

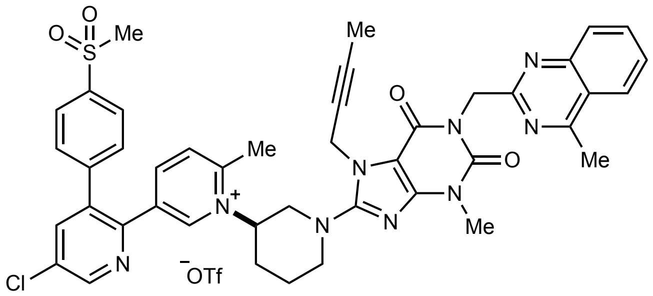

Prepared according to general procedure B using 5-chloro-6'-methyl-3-(4-(methylsulfonyl)phenyl)-2,3'-bipyridine (etoricoxib) (538 mg, 1.50 mmol), EtOAc (7.5 mL, 0.2 M), Tf_2O (250 μL, 1.50 mmol), dibenzylamine (350 μL, 1.80 mmol), collidine (200 μL, 1.500 mmol), AcOH (860 μL, 15.0 mmol), potassium metabisulfite (334 mg, 1.500 mmol), (*R*)-8-(3-aminopiperidin-1-yl)-7-(but-2-yn-1-yl)-3-methyl-1-((4-methylquinazolin-2-yl)methyl)-3,7-dihydro-1*H*-purine-2,6-dione (linagliptin) (1.06 g, 2.25 mmol), and heated to 70 ºC for 18 h. Isolated according to general isolation procedure B1 using NaOTf (1.29 g, 7.50 mmol), CH_2Cl_2 (60 mL), 1.0 M AcOH wash (1 x 30 mL), H_2O washes (2 x 30 mL), sat. Na_2CO_3 wash (1 x 30 mL), and 1:1 EtOAc/Et_2O (100 mL) to afford the title compound as an off-white solid (502 mg, 0.521 mmol, 35% yield). ^{1}H NMR (400 MHz, DMSO) δ: 8.98 (d, *J* = 2.2 Hz, 1H), 8.81 (d, *J* = 1.9 Hz, 1H), 8.55 (dd, *J* = 8.3, 1.7 Hz, 1H), 8.34 (d, *J* = 2.3 Hz, 1H), 8.25 (d, *J* = 7.8 Hz, 1H), 8.14 (d, *J* = 8.4 Hz, 1H), 7.96 (d, *J* = 8.4 Hz, 2H), 7.92 (ddd, *J* = 8.5, 6.9, 1.4 Hz, 1H), 7.79 (d, *J* = 8.4 Hz, 1H), 7.68 (ddd, *J* = 8.3, 6.8, 1.2 Hz, 1H), 7.63 (d, *J* = 8.4 Hz, 2H), 5.33 (t, *J* = 2.7 Hz, 2H), 5.17 – 5.07 (m, 1H), 4.91 (d, *J* = 3.1 Hz, 2H), 3.93 (d, *J* = 12.4 Hz, 1H), 3.77 (d, *J* = 13.1 Hz, 1H), 3.40 (s, 3H), 3.27 – 3.19 (m, 4H), 3.15 – 3.00 (m, 3H), 2.88 (s, 3H), 1.89 – 1.82 (m, 1H), 1.81 – 1.65 (m, 6H), 1.58 (dd, *J* = 10.9, 5.1 Hz, 1H); ^{13}C NMR (100 MHz, DMSO) δ: 168.91, 160.86, 155.62, 154.56, 153.37, 150.83, 149.02, 148.67, 148.17, 147.15, 145.56, 142.84, 141.43, 140.67, 138.34, 136.54, 135.99, 134.15, 131.91, 131.21, 130.06, 127.81, 127.38, 127.20, 125.81, 122.51, 121.96 (q, *J* = 313.7 Hz), 103.66, 81.45, 73.52, 60.02, 52.19, 49.41, 45.64, 43.21, 35.35, 29.46, 28.51, 23.26, 21.59, 20.15, 3.04; ^{19}F NMR (375 MHz, DMSO) δ: –77.75; m/z LRMS (ESI + APCI) found $[M – OTf]^+$ 814.3, $C_{43}H_{41}ClN_9O_4S^+$ requires 814.3.

9.3. Heterogeneous Hydrogenation of Enantioenriched *N*-Alkylpyridinium Salts

1-((*S*)-1-Cyclohexylethyl)-2-phenylpiperidine (5a) (major and minor)

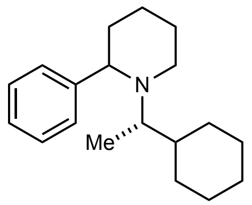

5.9:1 d.r.

major and minor

An oven dried 16 mL vial equipped with a stir bar was charged with (*S*)-1-(1-cyclohexylethyl)-2-phenylpyridin-1-ium trifluoromethanesulfonate (**3a**) (166 mg, 0.400 mmol), PtO_2 (10 mol%, 0.0400 mmol), and MeOH (4.00 mL, 0.1 M). The reaction vial was capped, and a double-skinned balloon of hydrogen gas was bubbled through the solution while stirring (550 rpm). After the balloon was depleted, it was replaced with a fresh, double-skinned balloon filled with hydrogen gas hovering in the headspace of the vial. The reaction was monitored by LCMS until full conversion of the starting material and intermediates was observed (5 h). The balloon was removed, and the reaction mixture was filtered through Celite and concentrated *in vacuo*. The resulting residue was dissolved in CH_2Cl_2 and washed with H_2O (x1) and sat. Na_2CO_3. The combined aqueous layers were extracted with CH_2Cl_2 (x2), and the combined organic layers were dried over Na_2SO_4, filtered, and concentrated *in vacuo*. Automated flash chromatography (silica gel: 5% MeOH in CH_2Cl_2) afforded the title compound as a mixture of diastereomers (5.9:1 d.r.) as a yellow oil (92.0 mg, 0.339 mmol, 84% yield). IR ν_{max}/cm^{-1} (film): 3025, 2921, 2850, 2793, 1447, 1329, 1029, 700; ^{1}H NMR (major, 400 MHz, $CDCl_3$) δ: 7.31 – 7.26 (m, 4H), 7.26 – 7.15 (m, 1H), 3.33 (dd, *J* = 10.8, 2.8 Hz, 1H), 2.78 (d, *J* = 11.5 Hz, 1H), 2.28 – 2.07 (m, 3H), 1.78 – 1.48 (m, 9H), 1.36 – 0.97 (m, 5H), 0.76 – 0.61 (m, 4H), 0.53 (qd, *J* = 12.1, 3.0 Hz, 1H); ^{13}C NMR (major, 100 MHz, $CDCl_3$) δ: 145.76, 128.25, 128.08, 126.65, 65.65, 57.48, 44.91, 41.15, 37.59, 31.19, 30.63, 26.89, 26.79, 26.79, 26.63, 25.79, 8.40; *m/z* LRMS (ESI + APCI) found $[M + H]^+$ 272.2, $C_{19}H_{30}N^+$ requires 272.2.

(3a*R*,4*S*,6*R*,6a*S*)-6-(2-Isopropylpiperidin-1-yl)-2,2-dimethyltetrahydro-4*H*-cyclopenta[*d*][1,3]dioxol-4-ol (5b) (major and minor)

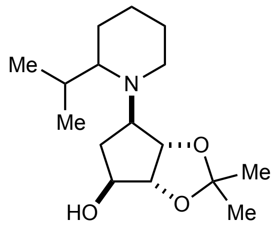

4.0:1 d.r.
major and minor

An oven dried 16 mL vial equipped with a stir bar was charged with 1-((3a*S*,4*R*,6*S*,6a*R*)-6-Hydroxy-2,2-dimethyltetrahydro-4*H*-cyclopenta[*d*][1,3]dioxol-4-yl)-2-isopropylpyridin-1-ium trifluoromethanesulfonate (**3q**) (171 mg, 0.400 mmol), PtO_2 (10 mol%, 0.0400 mmol), and MeOH (4.00 mL, 0.1 M). The reaction vial was capped, and a double-skinned balloon of hydrogen gas was bubbled through the solution while stirring (550 rpm). After the balloon was depleted, it was replaced with a fresh, double-skinned balloon filled with hydrogen gas hovering in the headspace of the vial. The reaction was monitored by LCMS until full conversion of the starting material and intermediates was observed (2.5 h). The balloon was removed, and the reaction mixture was filtered through Celite and concentrated *in vacuo*. The resulting residue was dissolved in CH_2Cl_2 and washed with H_2O (x1) and sat. Na_2CO_3. The combined aqueous layers were extracted with CH_2Cl_2 (x2), and the combined organic layers were dried over Na_2SO_4, filtered, and concentrated *in vacuo*. Automated flash chromatography (silica gel: 2% to 10% MeOH in CH_2Cl_2) afforded the title compound as a mixture of diastereomers (4.0:1 d.r.) as a light-yellow oil (89.0 mg, 0.314 mmol, 79% yield). The O–H proton was exchanged with deuterium CD_3OD during characterization. IR ν_{max}/cm^{-1} (film): 3387, 2931, 2862, 1454, 1370, 1206, 1160, 1054, 865; ^{1}H NMR (major and minor, 400 MHz, $CDCl_3$) δ: 4.97 – 4.61 (m, 1H, minor), 4.57 – 4.48 (m, 1H, major), 4.12 – 4.02 (m, 1H, major and minor), 3.67 – 3.50 (m, 1H, major and minor), 3.05 – 2.79 (m, 2H, major and minor), 2.62 – 2.50 (m, 1H, major and minor), 2.31 – 2.04 (m, 2H, major and minor), 2.04 – 1.92 (m, 1H, major and minor), 1.91 – 1.74 (m, 1H, major and minor), 1.65 – 1.47 (m, 4H, major and minor), 1.43 (s, 3H, major and minor), 1.33 – 1.21 (m, 5H, major and minor), 1.02 – 0.81 (m, 7H, major and minor); ^{13}C NMR (major

and minor, 100 MHz, $CDCl_3$) δ: 110.46 (major and minor), 86.19, 85.86, 82.97, 80.91, 77.36, 76.96 (major and minor), 51.01 (major and minor), 33.70, 31.18 (major and minor), 29.85, 26.57, 26.51, 24.29, 24.19, 20.97, 20.45 (major and minor), 20.37 (major and minor), 20.15, 19.96, 19.78 (major and minor); *m/z* LRMS (ESI + APCI) found $[M + H]^+$ 284.2, $C_{16}H_{30}NO_3^+$ requires 284.2.

2-(Trifluoromethyl)-5-(1-((1*R*,2*R*)-1,7,7-trimethylbicyclo[2.2.1]heptan-2-yl)piperidin-3-yl)pyridine (5c) (major and minor)

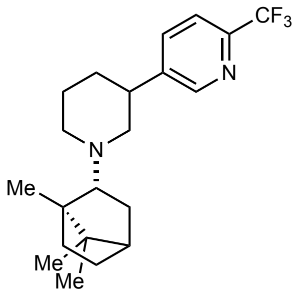

1.4:1 d.r.
major and minor

An oven dried 16 mL vial equipped with a stir bar was charged with 6'-(Trifluoromethyl)-1-((1*R*,2*R*)-1,7,7-trimethylbicyclo[2.2.1]heptan-2-yl)-[3,3'-bipyridin]-1-ium trifluoromethanesulfonate (**3r**) (204 mg, 0.400 mmol), PtO_2 (10 mol%, 0.0400 mmol), and MeOH (4.00 mL, 0.1 M). The reaction vial was capped, and a double-skinned balloon of hydrogen gas was bubbled through the solution while stirring (550 rpm). After the balloon was depleted, it was replaced with a fresh, double-skinned balloon filled with hydrogen gas hovering in the headspace of the vial. The reaction was monitored by LCMS until full conversion of the starting material and intermediates was observed (5 h). The balloon was removed, and the reaction mixture was filtered through Celite and concentrated *in vacuo*. The resulting residue was dissolved in CH_2Cl_2 and washed with H_2O (x1) and sat. Na_2CO_3. The combined aqueous layers were extracted with CH_2Cl_2 (x2), and the combined organic layers were dried over Na_2SO_4, filtered, and concentrated *in vacuo*. Automated flash chromatography (silica gel: 2% to 5% MeOH in CH_2Cl_2) afforded the title compound as a mixture of diastereomers (1.4:1 d.r.) as a light-yellow oil (119 mg, 0.325 mmol, 81% yield). IR ν_{max}/cm^{-1} (film): 2931, 2873, 2765, 1453, 1336, 1132, 1085, 1026, 845; ^1H NMR (major and minor, 400 MHz, $CDCl_3$) δ: 8.69 – 8.53 (m, 1H), 7.78 – 7.66 (m, 1H), 7.65 – 7.54 (m, 1H), 3.21 – 3.01 (m, 2H), 2.90 (major, tt, *J* = 11.0, 3.6 Hz, 1H), 2.81 (minor, tt, *J* = 11.1, 3.8 Hz, 1H), 2.21 – 2.14 (m, 1H), 2.03 – 1.76 (m, 5H), 1.72 – 1.56 (m, 4H), 1.52 – 1.23 (m, 3H), 1.09 – 0.99 (m, 2H), 0.99 – 0.94 (m, 3H), 0.93 – 0.88 (m, 3H), 0.79 (s, 3H); ^{13}C NMR (major and minor, 100 MHz, $CDCl_3$) δ: 149.77, 149.70, 144.22, 144.03, 136.01, 135.80, 128.78 (major and minor, q, *J* = 81.4 Hz), 121.85 (major and minor, q, *J* = 273.3 Hz), 120.55 – 119.98 (major and minor, m), 72.89, 72.64, 62.97 (major and minor), 56.54, 56.39, 49.72 (major and minor), 47.16, 47.13, 45.10 (major and minor), 41.42, 40.55, 37.33, 37.17, 33.45, 33.44, 32.11, 31.80, 27.45 (major and minor), 26.06, 25.57, 20.86 (major and minor), 19.79, 19.75, 14.34, 14.16; ^{19}F NMR (major and minor, 375 MHz, $CDCl_3$) δ: –67.72 (minor), –67.73 (major); *m/z* LRMS (ESI + APCI) found $[M + H]^+$ 367.1, $C_{21}H_{30}F_3N_2^+$ requires 367.2.

10. References

(1) Boyle, B. T.; Levy, J. N.; de Lescure, L.; Paton, R. S.; McNally, A. Halogenation of the 3-Position of Pyridines through Zincke Imine Intermediates. *Science* **2022**, *378* (6621), 773–779. https://doi.org/10.1126/science.add8980.

(2) Selingo, J. D.; Greenwood, J. W.; Andrews, M. K.; Patel, C.; Neel, A. J.; Pio, B.; Shevlin, M.; Phillips, E. M.; Maddess, M. L.; McNally, A. A General Strategy for N-(Hetero)Arylpiperidine Synthesis Using Zincke Imine Intermediates. *J. Am. Chem. Soc.* **2024**, *146* (1), 936–945. https://doi.org/10.1021/jacs.3c11504.
(3) Nguyen, H. M. H.; Thomas, D. C.; Hart, M. A.; Steenback, K. R.; Levy, J. N.; McNally, A. Synthesis of 15N-Pyridines and Higher Mass Isotopologs via Zincke Imine Intermediates. *J. Am. Chem. Soc.* **2024**, *146* (5), 2944–2949. https://doi.org/10.1021/jacs.3c12445.
(4) Jüstel, P. M.; Pignot, C. D.; Ofial, A. R. Nucleophilic Reactivities of Thiophenolates. *J. Org. Chem.* **2021**, *86* (8), 5965–5972. https://doi.org/10.1021/acs.joc.1c00025.
(5) Bordwell, F. G.; Hughes, D. L. Thiol Acidities and Thiolate Ion Reactivities toward Butyl Chloride in Dimethyl Sulfoxide Solution. The Question of Curvature in Broensted Plots. *J. Org. Chem.* **1982**, *47* (17), 3224–3232. https://doi.org/10.1021/jo00138a005.
(6) Zhao, Y.; Truhlar, D. G. The M06 Suite of Density Functionals for Main Group Thermochemistry, Thermochemical Kinetics, Noncovalent Interactions, Excited States, and Transition Elements: Two New Functionals and Systematic Testing of Four M06-Class Functionals and 12 Other Functionals. *Theor. Chem. Acc.* **2008**, *120* (1–3), 215–241. https://doi.org/10.1007/s00214-007-0310-x.
(7) Grimme, S.; Antony, J.; Ehrlich, S.; Krieg, H. A Consistent and Accurate *Ab Initio* Parametrization of Density Functional Dispersion Correction (DFT-D) for the 94 Elements H-Pu. *J. Chem. Phys.* **2010**, *132* (15), 154104. https://doi.org/10.1063/1.3382344.
(8) Spitznagel, G. W.; Clark, T.; Von Ragué Schleyer, P.; Hehre, W. J. An Evaluation of the Performance of Diffuse Function-augmented Basis Sets for Second Row Elements, Na-Cl. *J. Comput. Chem.* **1987**, *8* (8), 1109–1116. https://doi.org/10.1002/jcc.540080807.
(9) Clark, T.; Chandrasekhar, J.; Spitznagel, G. W.; Schleyer, P. V. R. Efficient Diffuse Function-augmented Basis Sets for Anion Calculations. III. The 3-21+G Basis Set for First-row Elements, Li–F. *J. Comput. Chem.* **1983**, *4* (3), 294–301. https://doi.org/10.1002/jcc.540040303.
(10) Francl, M. M.; Pietro, W. J.; Hehre, W. J.; Binkley, J. S.; Gordon, M. S.; DeFrees, D. J.; Pople, J. A. Self-Consistent Molecular Orbital Methods. XXIII. A Polarization-Type Basis Set for Second-Row Elements. *J. Chem. Phys.* **1982**, *77* (7), 3654–3665. https://doi.org/10.1063/1.444267.
(11) Gordon, M. S.; Binkley, J. S.; Pople, J. A.; Pietro, W. J.; Hehre, W. J. Self-Consistent Molecular-Orbital Methods. 22. Small Split-Valence Basis Sets for Second-Row Elements. *J. Am. Chem. Soc.* **1982**, *104* (10), 2797–2803. https://doi.org/10.1021/ja00374a017.
(12) Ditchfield, R.; Hehre, W. J.; Pople, J. A. Self-Consistent Molecular-Orbital Methods. IX. An Extended Gaussian-Type Basis for Molecular-Orbital Studies of Organic Molecules. *J. Chem. Phys.* **1971**, *54* (2), 724–728. https://doi.org/10.1063/1.1674902.
(13) Hehre, W. J.; Ditchfield, R.; Pople, J. A. Self—Consistent Molecular Orbital Methods. XII. Further Extensions of Gaussian—Type Basis Sets for Use in Molecular Orbital Studies of Organic Molecules. *J. Chem. Phys.* **1972**, *56* (5), 2257–2261. https://doi.org/10.1063/1.1677527.
(14) Hariharan, P. C.; Pople, J. A. The Influence of Polarization Functions on Molecular Orbital Hydrogenation Energies. *Theor. Chim. Acta* **1973**, *28* (3), 213–222. https://doi.org/10.1007/BF00533485.
(15) Miertus, S. APPROXIMiTE EVALUATIONS OF THJZELECTROSTATIC FREE ENERGY ANb INTERNAL ENERGY CHANGES IN SOLUTION PROCESSES.
(16) Miertuš, S.; Scrocco, E.; Tomasi, J. Electrostatic Interaction of a Solute with a Continuum. A Direct Utilizaion of AB Initio Molecular Potentials for the Prevision of Solvent Effects. *Chem. Phys.* **1981**, *55* (1), 117–129. https://doi.org/10.1016/0301-0104(81)85090-2.
(17) Tomasi, J.; Mennucci, B.; Cammi, R. Quantum Mechanical Continuum Solvation Models. *Chem. Rev.* **2005**, *105* (8), 2999–3094. https://doi.org/10.1021/cr9904009.
(18) Grimme, S. Supramolecular Binding Thermodynamics by Dispersion-Corrected Density Functional Theory. *Chem. – Eur. J.* **2012**, *18* (32), 9955–9964. https://doi.org/10.1002/chem.201200497.

(19) Luchini, G.; Alegre-Requena, J. V.; Funes-Ardoiz, I.; Paton, R. S. GoodVibes: Automated Thermochemistry for Heterogeneous Computational Chemistry Data. *F1000Research* **2020**, *9*, 291. https://doi.org/10.12688/f1000research.22758.1.
(20) Mardirossian, N.; Head-Gordon, M. *ω* B97M-V: A Combinatorially Optimized, Range-Separated Hybrid, Meta-GGA Density Functional with VV10 Nonlocal Correlation. *J. Chem. Phys.* **2016**, *144* (21), 214110. https://doi.org/10.1063/1.4952647.
(21) Weigend, F.; Ahlrichs, R. Balanced Basis Sets of Split Valence, Triple Zeta Valence and Quadruple Zeta Valence Quality for H to Rn: Design and Assessment of Accuracy. *Phys. Chem. Chem. Phys.* **2005**, *7* (18), 3297. https://doi.org/10.1039/b508541a.
(22) Cossi, M.; Rega, N.; Scalmani, G.; Barone, V. Energies, Structures, and Electronic Properties of Molecules in Solution with the C-PCM Solvation Model. *J. Comput. Chem.* **2003**, *24* (6), 669–681. https://doi.org/10.1002/jcc.10189.
(23) De Souza, B. GOAT: A Global Optimization Algorithm for Molecules and Atomic Clusters. *Angew. Chem. Int. Ed.* **2025**, *64* (18), e202500393. https://doi.org/10.1002/anie.202500393.
(24) Bannwarth, C.; Ehlert, S.; Grimme, S. GFN2-xTB—An Accurate and Broadly Parametrized Self-Consistent Tight-Binding Quantum Chemical Method with Multipole Electrostatics and Density-Dependent Dispersion Contributions. *J. Chem. Theory Comput.* **2019**, *15* (3), 1652–1671. https://doi.org/10.1021/acs.jctc.8b01176.
(25) Pracht, P.; Bohle, F.; Grimme, S. Automated Exploration of the Low-Energy Chemical Space with Fast Quantum Chemical Methods. *Phys. Chem. Chem. Phys.* **2020**, *22* (14), 7169–7192. https://doi.org/10.1039/C9CP06869D.
(26) Luchini, G.; Alegre-Requena, J. V.; Funes-Ardoiz, I.; Paton, R. S. GoodVibes: Automated Thermochemistry for Heterogeneous Computational Chemistry Data. *F1000Research* **2020**, *9*, 291. https://doi.org/10.12688/f1000research.22758.1.
(27) Marenich, A. V.; Cramer, C. J.; Truhlar, D. G. Universal Solvation Model Based on Solute Electron Density and on a Continuum Model of the Solvent Defined by the Bulk Dielectric Constant and Atomic Surface Tensions. *J. Phys. Chem. B* **2009**, *113* (18), 6378–6396. https://doi.org/10.1021/jp810292n.
(28) Dykstra, K. D.; Streckfuss, E.; Liu, M.; Liu, J.; Yu, Y.; Wang, M.; Kozlowski, J. A.; Myers, R. W.; Buevich, A. V.; Maletic, M. M.; Vachal, P.; Krska, S. W. Synthesis of HDAC Inhibitor Libraries via Microscale Workflow. *ACS Med. Chem. Lett.* **2021**, *12* (3), 337–342. https://doi.org/10.1021/acsmedchemlett.0c00596.

11. NMR Data (^{1}H, ^{13}C, ^{19}F spectra)

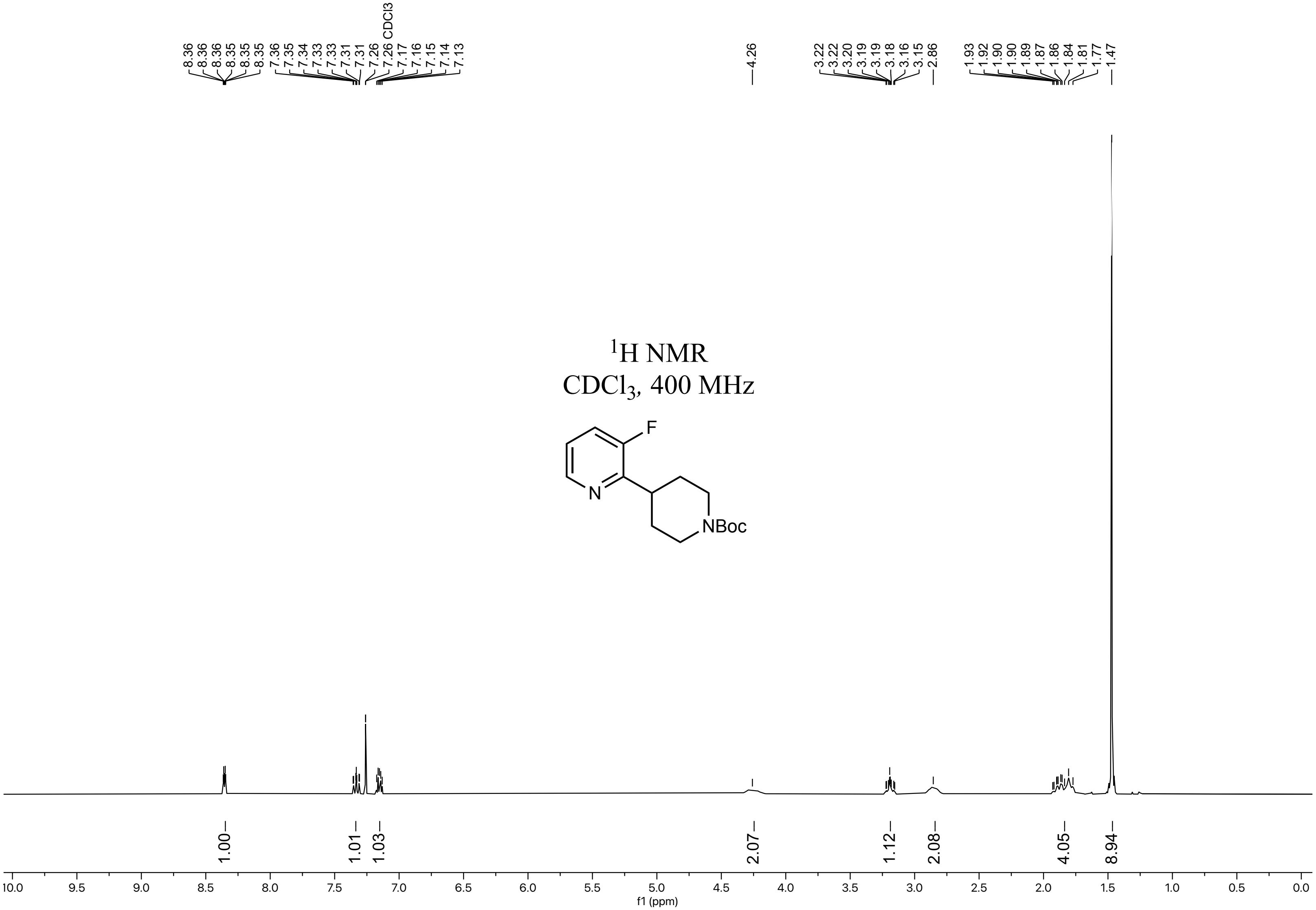

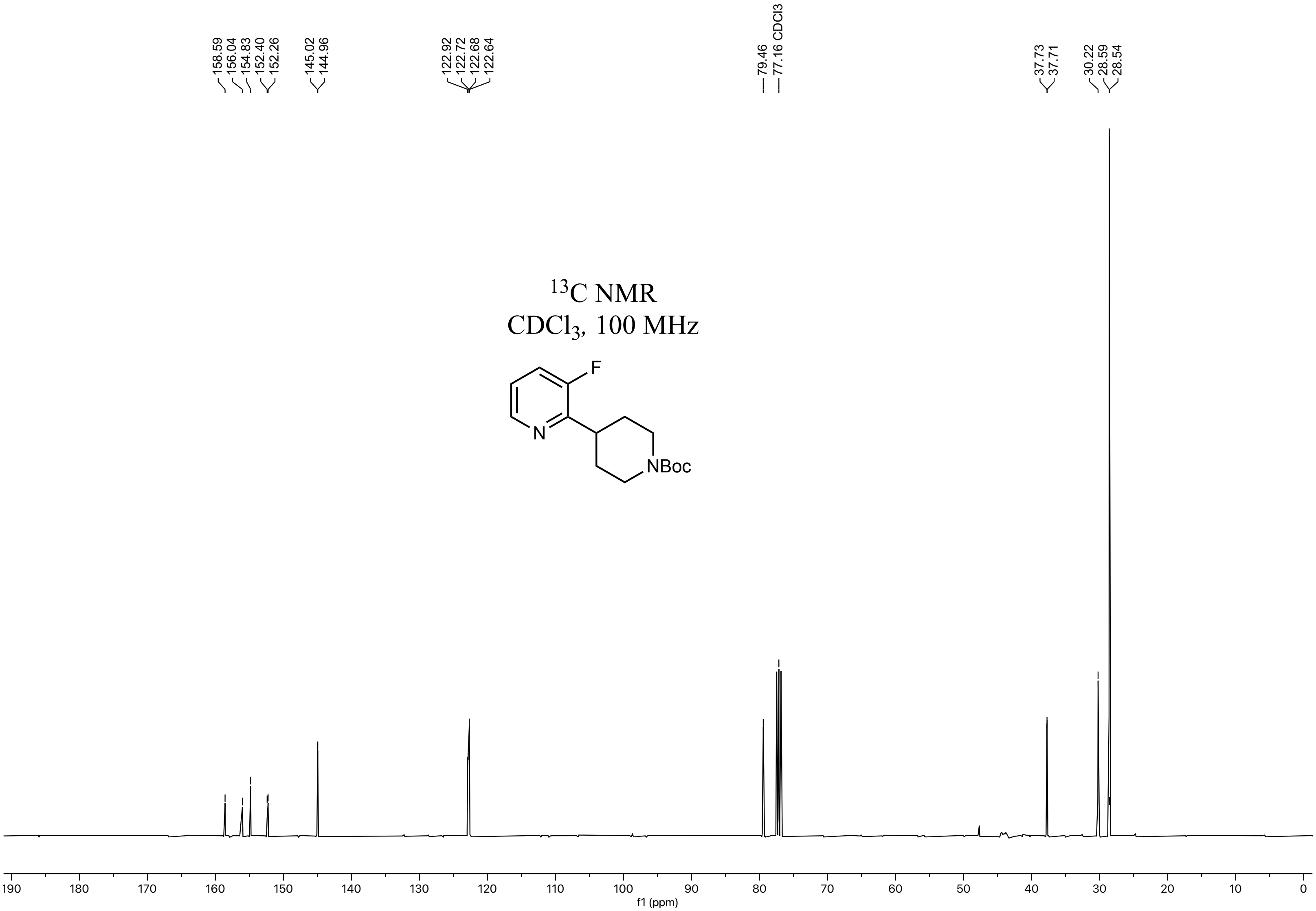

158.59
156.04
154.83
152.40
152.26
145.02
144.96
122.92
122.72
122.68
122.64
79.46
77.16 CDCl3
37.73
37.71
30.22
28.59
28.54
^{13}C NMR
$CDCl_3$, 100 MHz
F
N
NBoc
190
180
170
160
150
140
130
120
110
100
90
80
70
60
50
40
30
20
10
0
f1 (ppm)

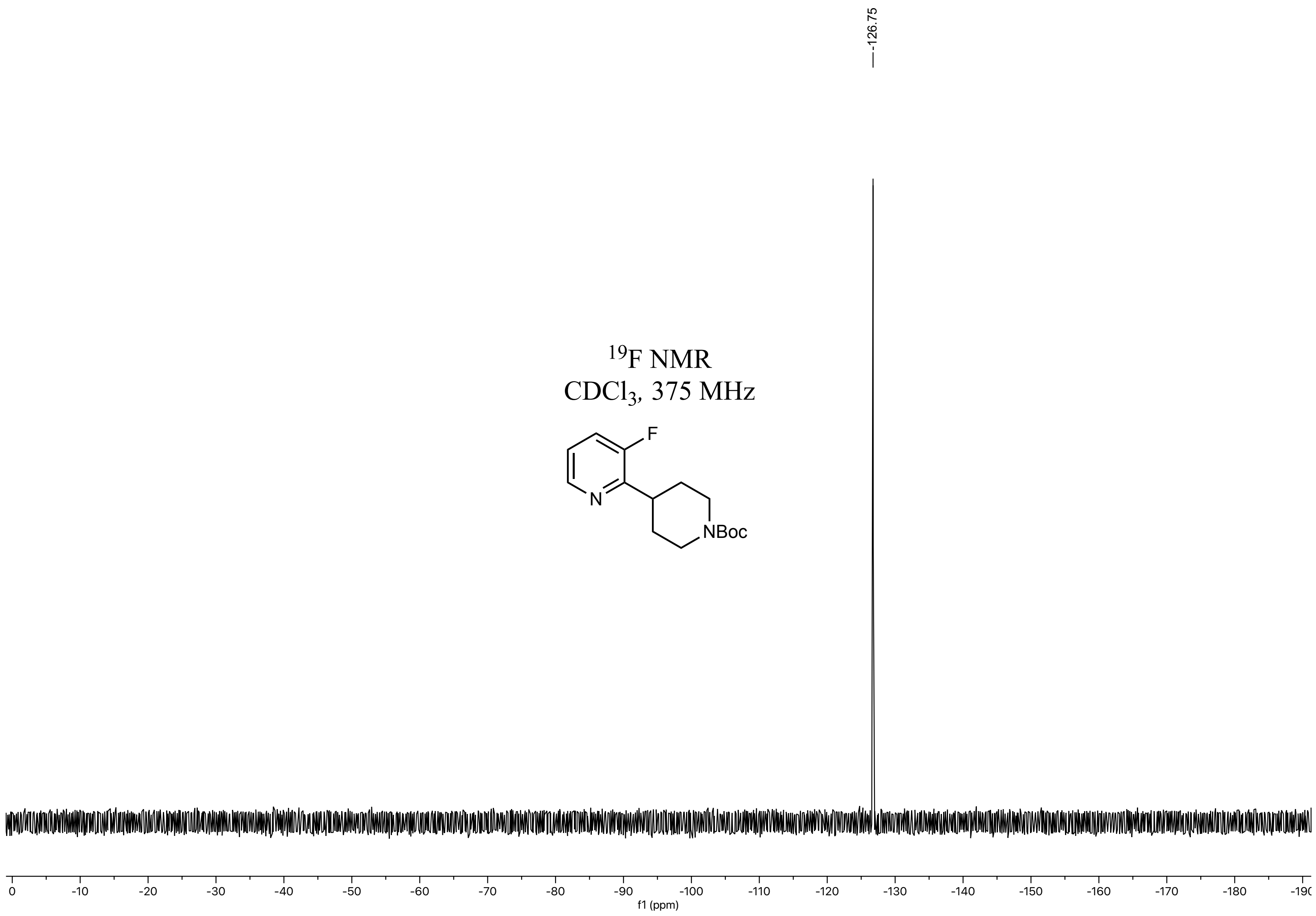
-126.75
19F NMR
CDCl3, 375 MHz
F
N
NBoc
0 -10 -20 -30 -40 -50 -60 -70 -80 -90 -100 -110 -120 -130 -140 -150 -160 -170 -180 -190
f1 (ppm)

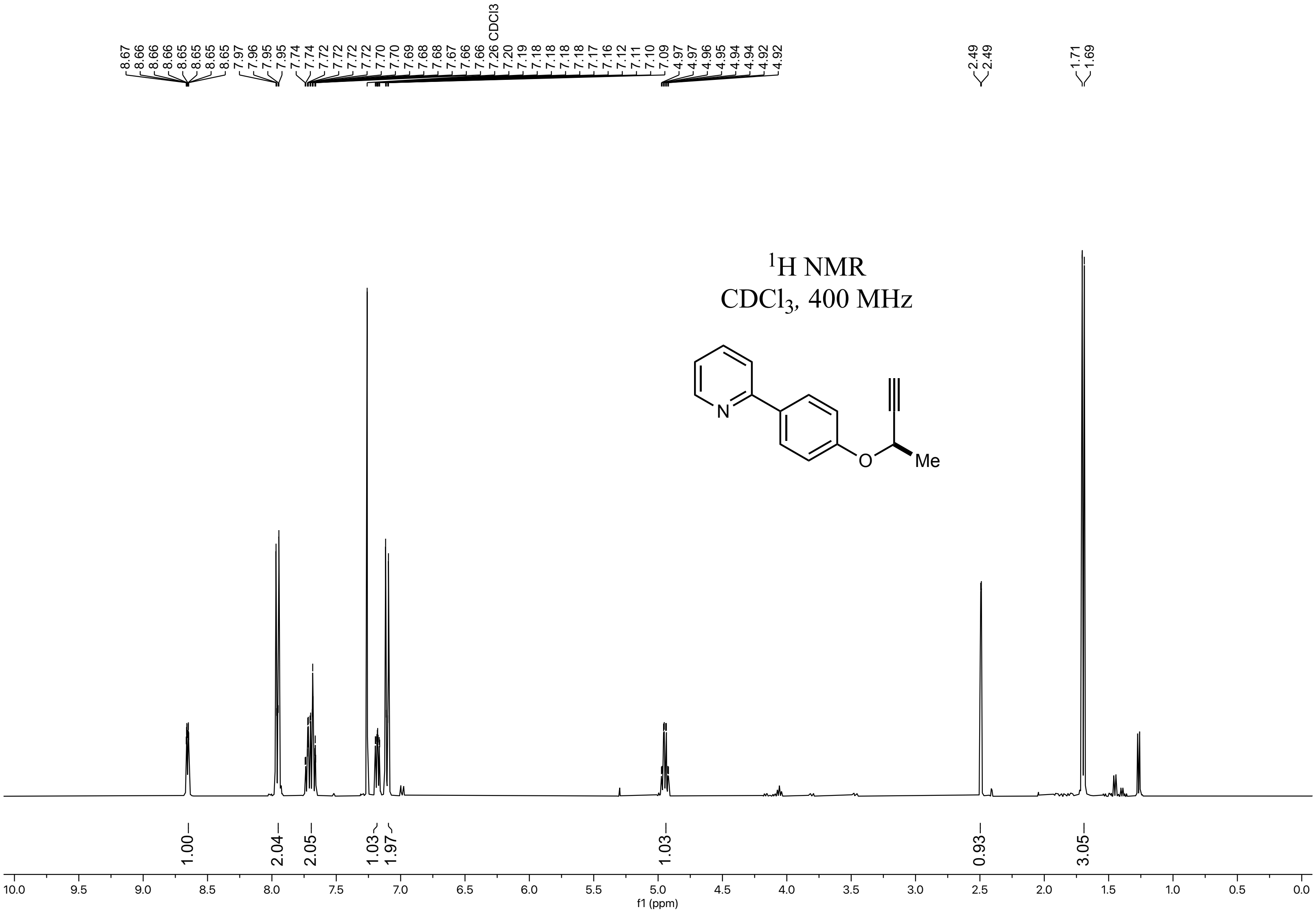

1H NMR
CDCl3, 400 MHz
N
O
Me
8.67
8.66
8.66
8.66
8.65
8.65
8.65
8.65
7.97
7.96
7.95
7.95
7.74
7.74
7.72
7.72
7.72
7.72
7.70
7.70
7.69
7.68
7.68
7.67
7.66
7.66
7.26 CDCl3
7.20
7.19
7.18
7.18
7.18
7.18
7.17
7.16
7.12
7.11
7.10
7.09
4.97
4.97
4.96
4.95
4.94
4.94
4.92
4.92
2.49
2.49
1.71
1.69
1.00
2.04
2.05
1.03
1.97
1.03
0.93
3.05
f1 (ppm)

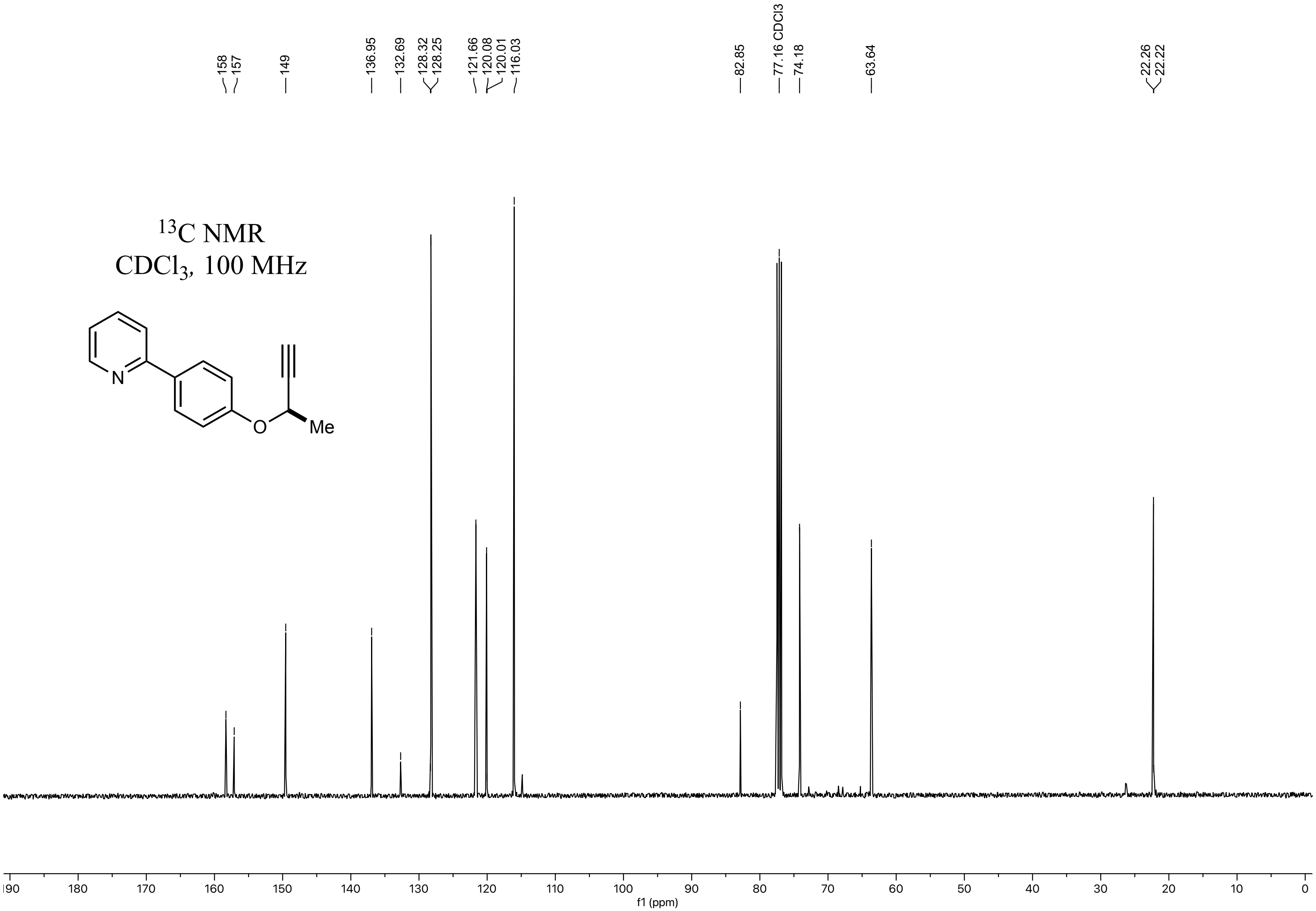
158
157
149
136.95
132.69
128.32
128.25
121.66
120.08
120.01
116.03
82.85
77.16 CDCl3
74.18
63.64
22.26
22.22
13C NMR
CDCl3, 100 MHz
N
O
Me
f1 (ppm)

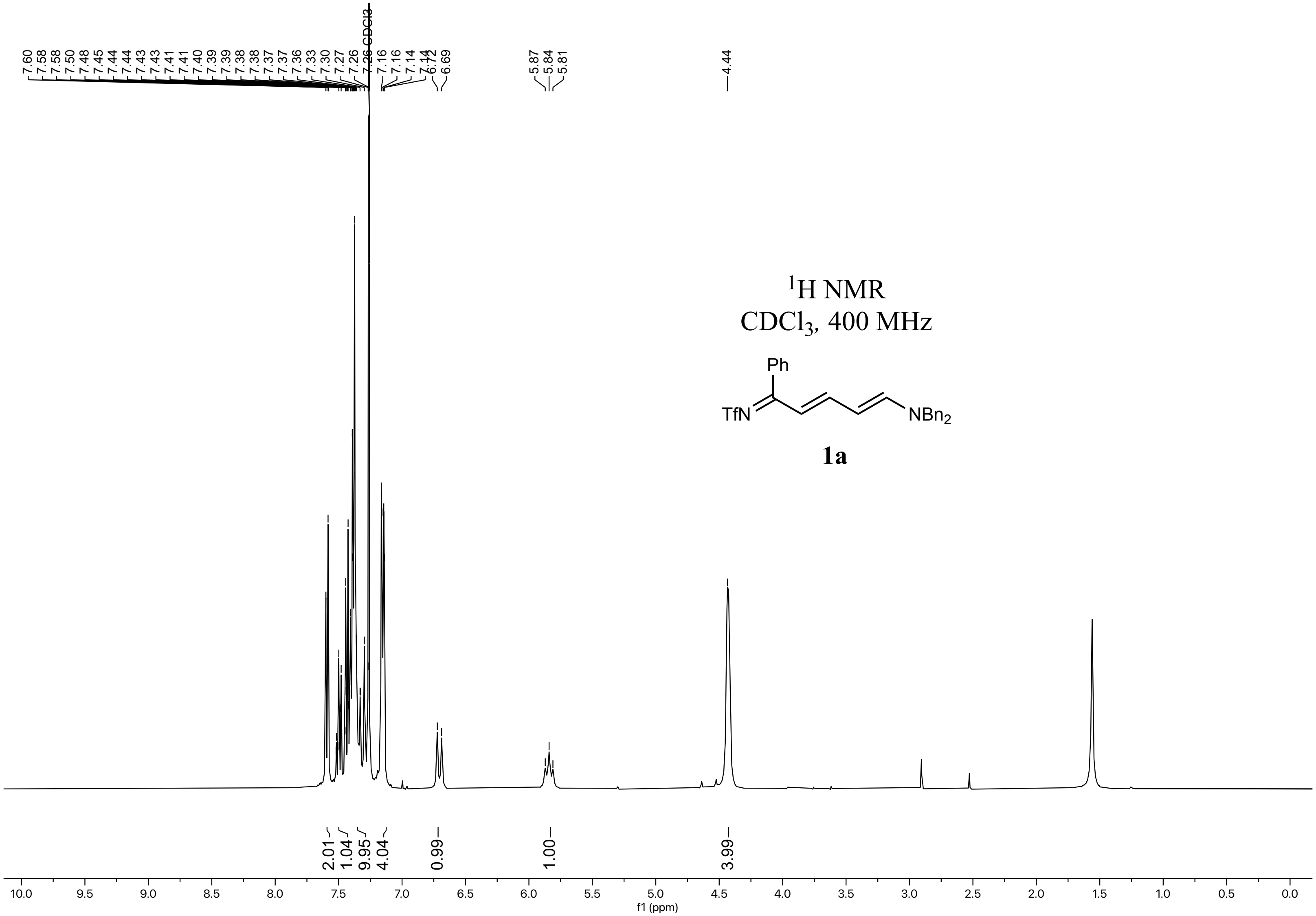

¹H NMR
CDCl3, 400 MHz
Ph
TfN
NBn2
1a
f1 (ppm)

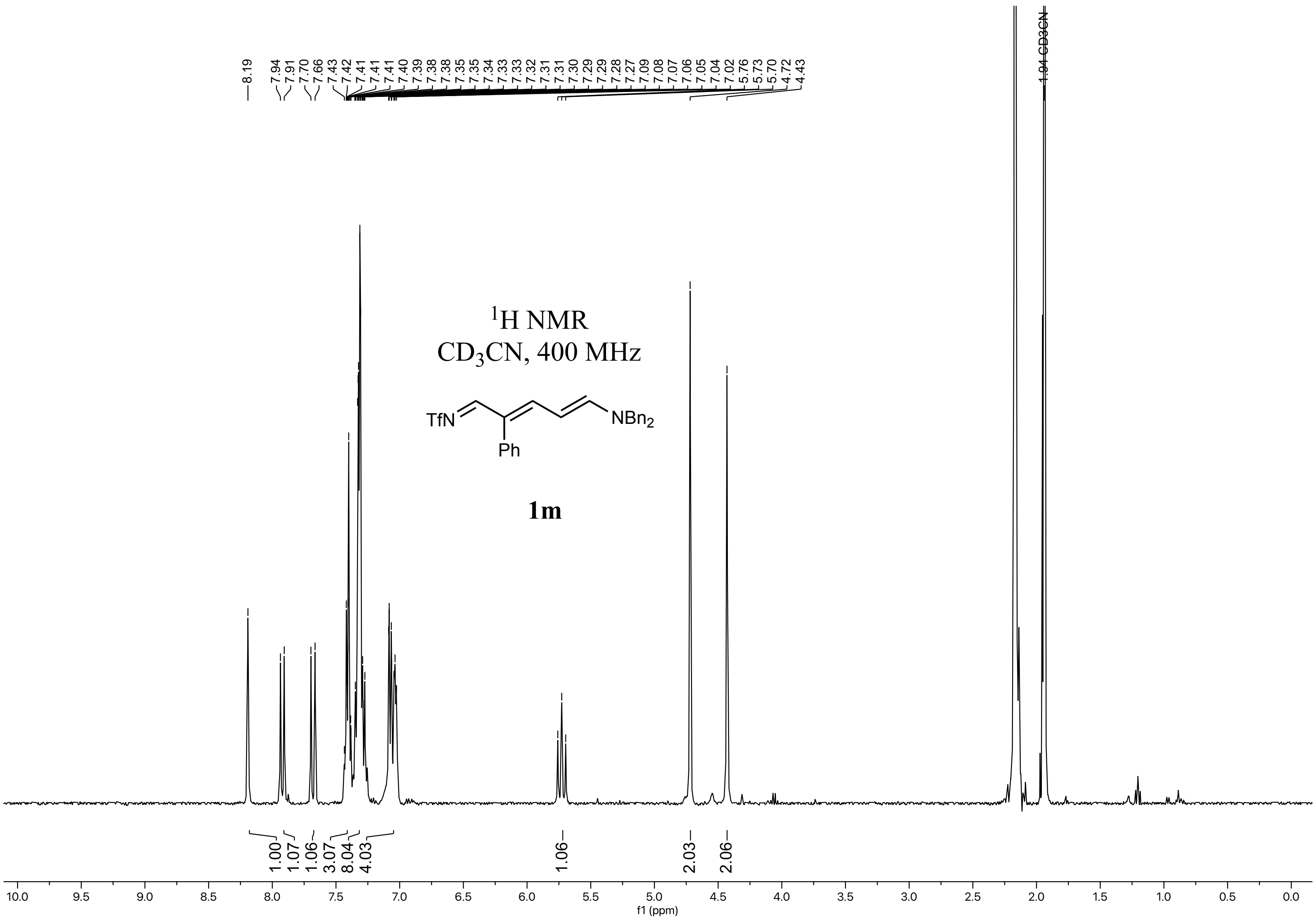

8.19
7.94
7.91
7.70
7.66
7.43
7.42
7.41
7.41
7.41
7.40
7.39
7.38
7.38
7.35
7.35
7.34
7.33
7.33
7.32
7.31
7.31
7.30
7.29
7.29
7.28
7.27
7.09
7.08
7.07
7.06
7.05
7.04
7.02
5.76
5.73
5.70
4.72
4.43
1.94 CD3CN
^{1}H NMR
CD_3CN, 400 MHz
TfN
NBn$_2$
Ph
1m
1.00
1.07
1.06
3.07
8.04
4.03
1.06
2.03
2.06
10.0
9.5
9.0
8.5
8.0
7.5
7.0
6.5
6.0
5.5
5.0
4.5
4.0
3.5
3.0
2.5
2.0
1.5
1.0
0.5
0.0
f1 (ppm)

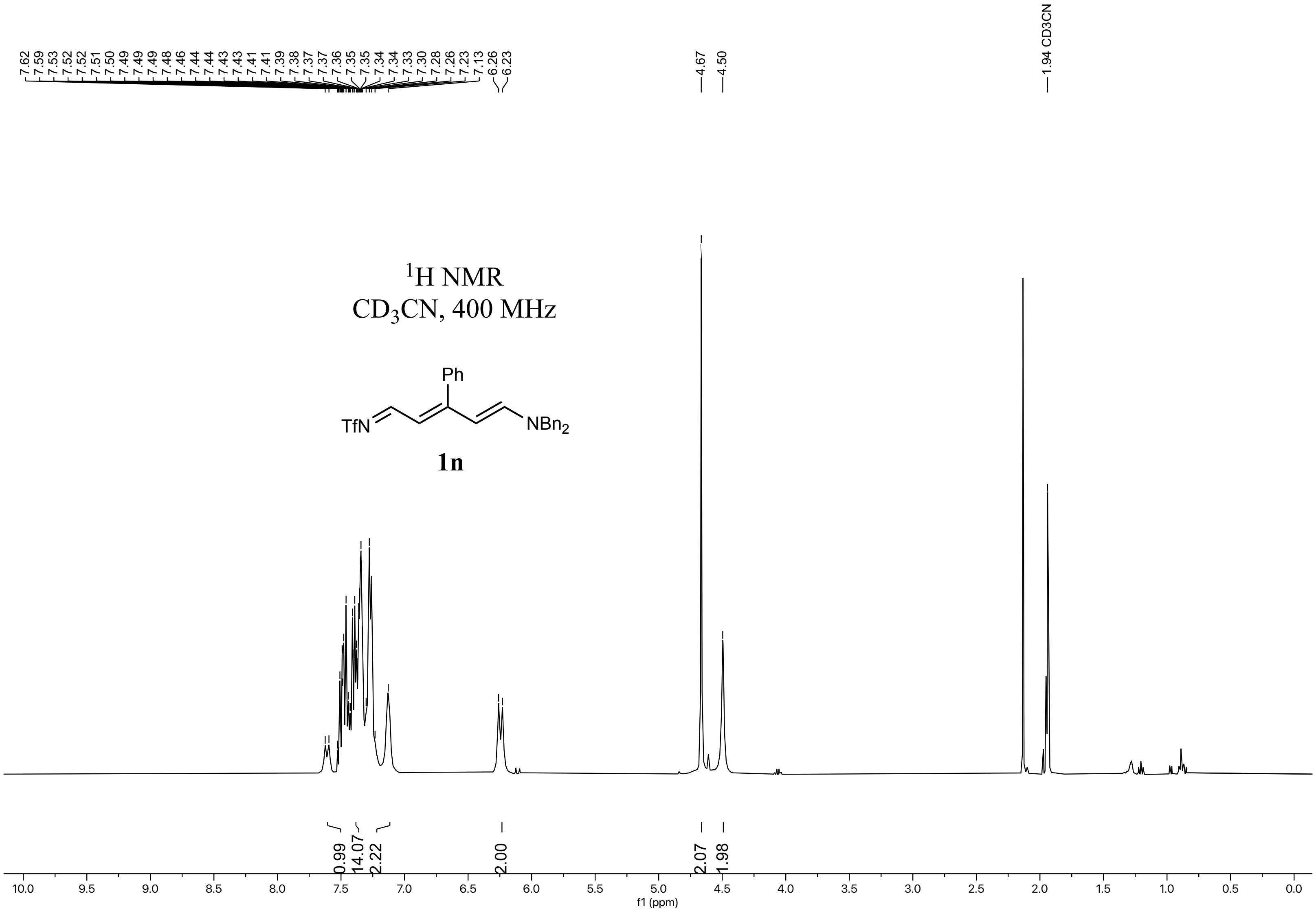
7.62 7.59 7.53 7.52 7.52 7.51 7.50 7.49 7.49 7.49 7.48 7.46 7.44 7.44 7.43 7.43 7.41 7.41 7.39 7.38 7.37 7.37 7.36 7.35 7.35 7.34 7.34 7.33 7.30 7.28 7.26 7.23 7.13 6.26 6.23
4.67
4.50
1.94 CD3CN
^{1}H NMR
CD_3CN, 400 MHz
Ph
TfN
NBn_2
1n
0.99
14.07
2.22
2.00
2.07
1.98
10.0 9.5 9.0 8.5 8.0 7.5 7.0 6.5 6.0 5.5 5.0 4.5 4.0 3.5 3.0 2.5 2.0 1.5 1.0 0.5 0.0
f1 (ppm)

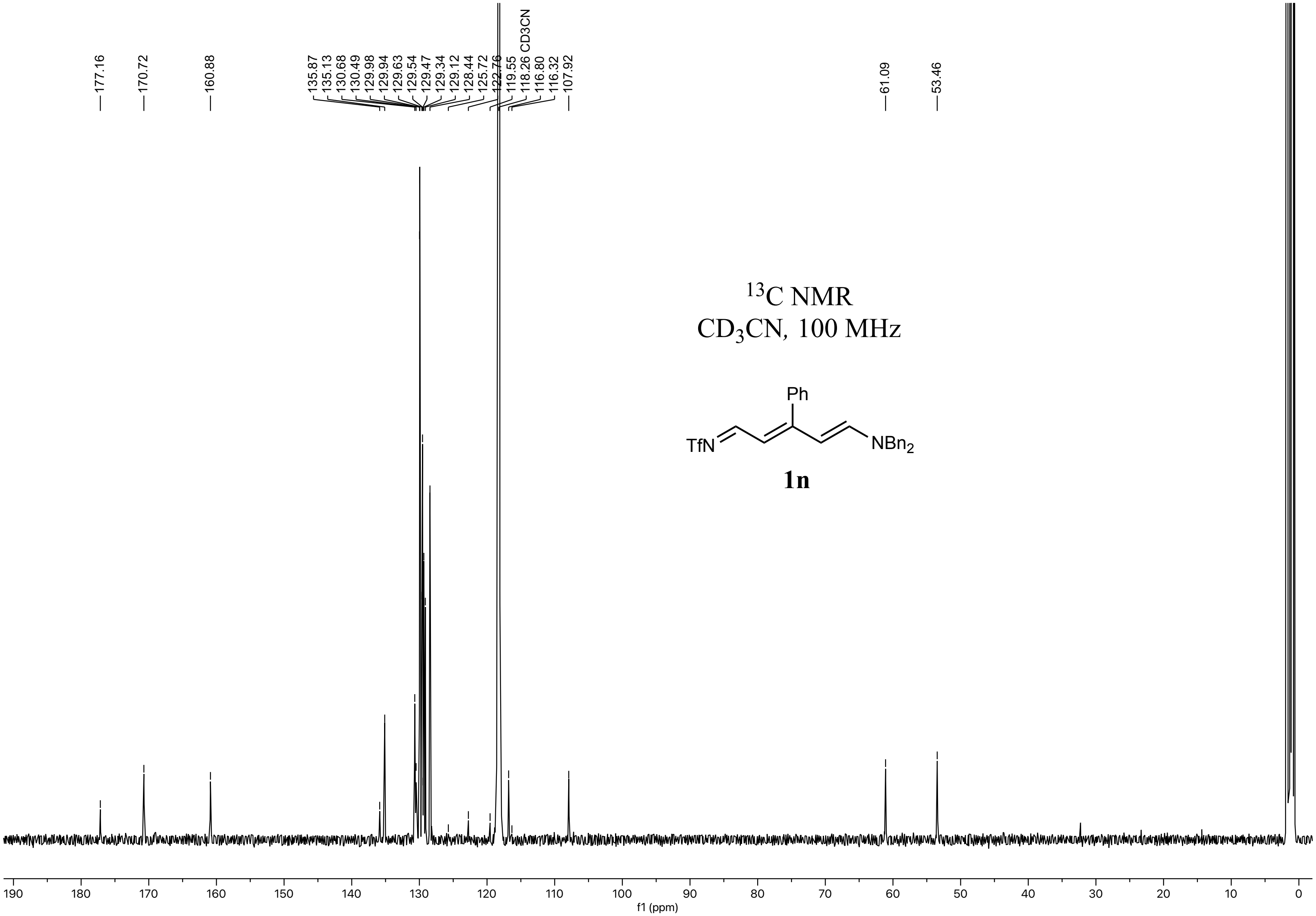
177.16
170.72
160.88
135.87
135.13
130.68
130.49
129.98
129.94
129.63
129.54
129.47
129.34
129.12
128.44
125.72
122.76
119.55
118.26 CD3CN
116.80
116.32
107.92
61.09
53.46
^{13}C NMR
CD_3CN, 100 MHz
Ph
TfN
NBn_2
1n
f1 (ppm)

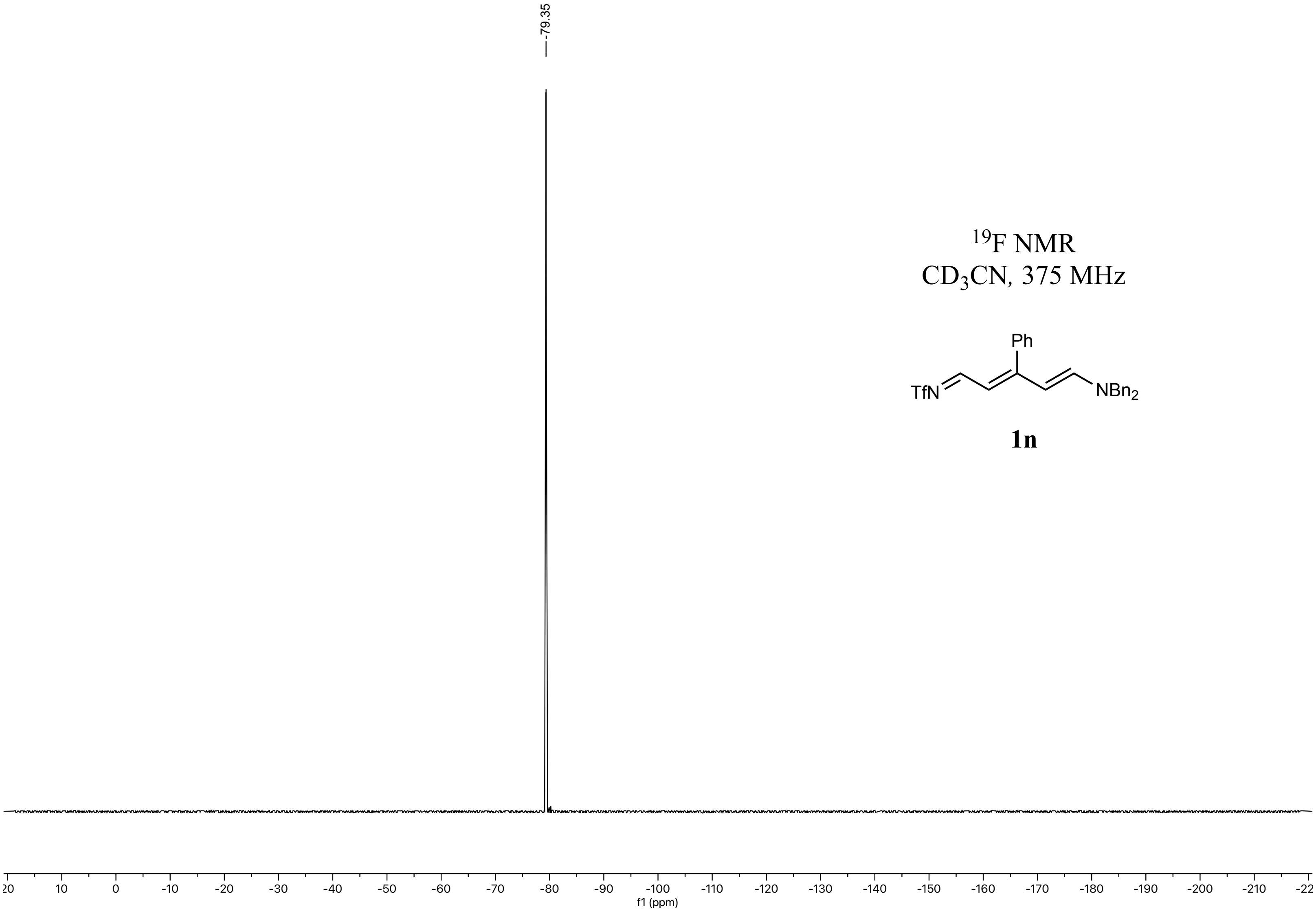

^{19}F NMR
CD_3CN, 375 MHz

1n

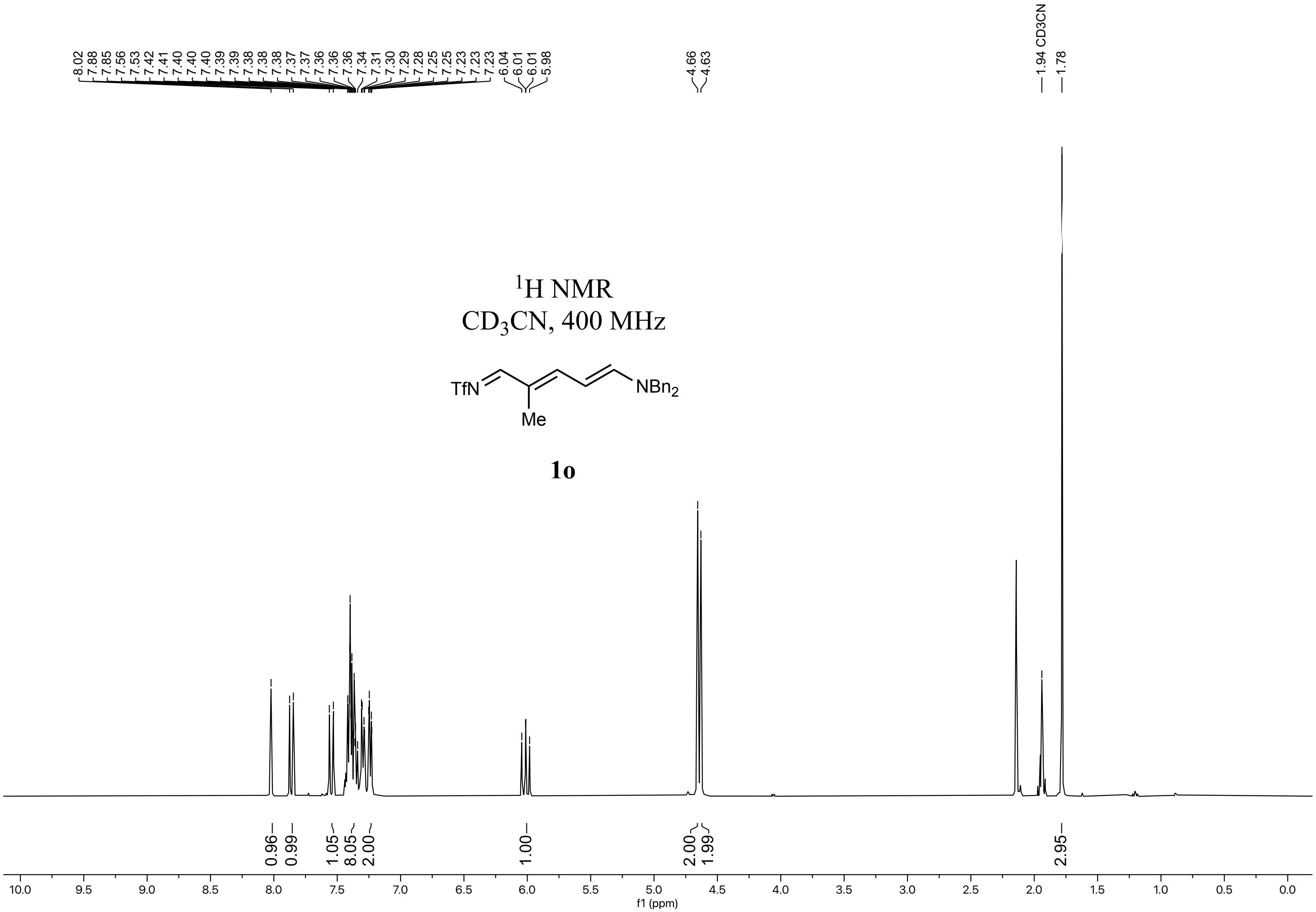

8.02
7.88
7.85
7.56
7.53
7.42
7.41
7.40
7.40
7.39
7.39
7.38
7.38
7.38
7.37
7.37
7.36
7.36
7.36
7.34
7.31
7.30
7.29
7.28
7.25
7.25
7.23
7.23
7.23
6.04
6.01
6.01
5.98
4.66
4.63
1.94 CD3CN
1.78
^{1}H NMR
CD_3CN, 400 MHz
TfN
NBn2
Me
1o
0.96
0.99
1.05
8.05
2.00
1.00
2.00
1.99
2.95
f1 (ppm)

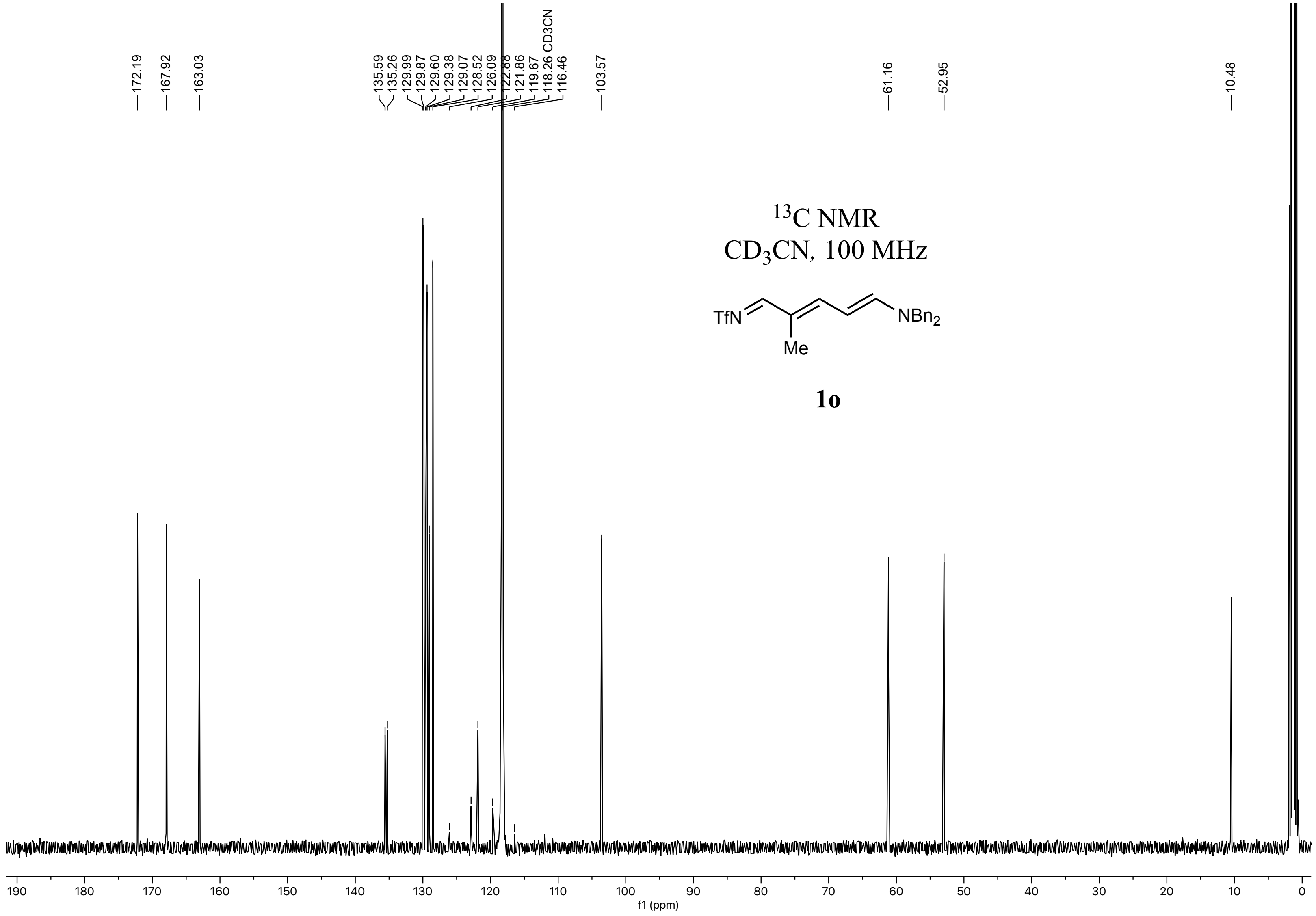

172.19
167.92
163.03
135.59
135.26
129.99
129.87
129.60
129.38
129.07
128.52
126.09
122.88
121.86
119.67
118.26 CD3CN
116.46
103.57
61.16
52.95
10.48
^{13}C NMR
CD_3CN, 100 MHz
TfN
NBn2
Me
1o
f1 (ppm)

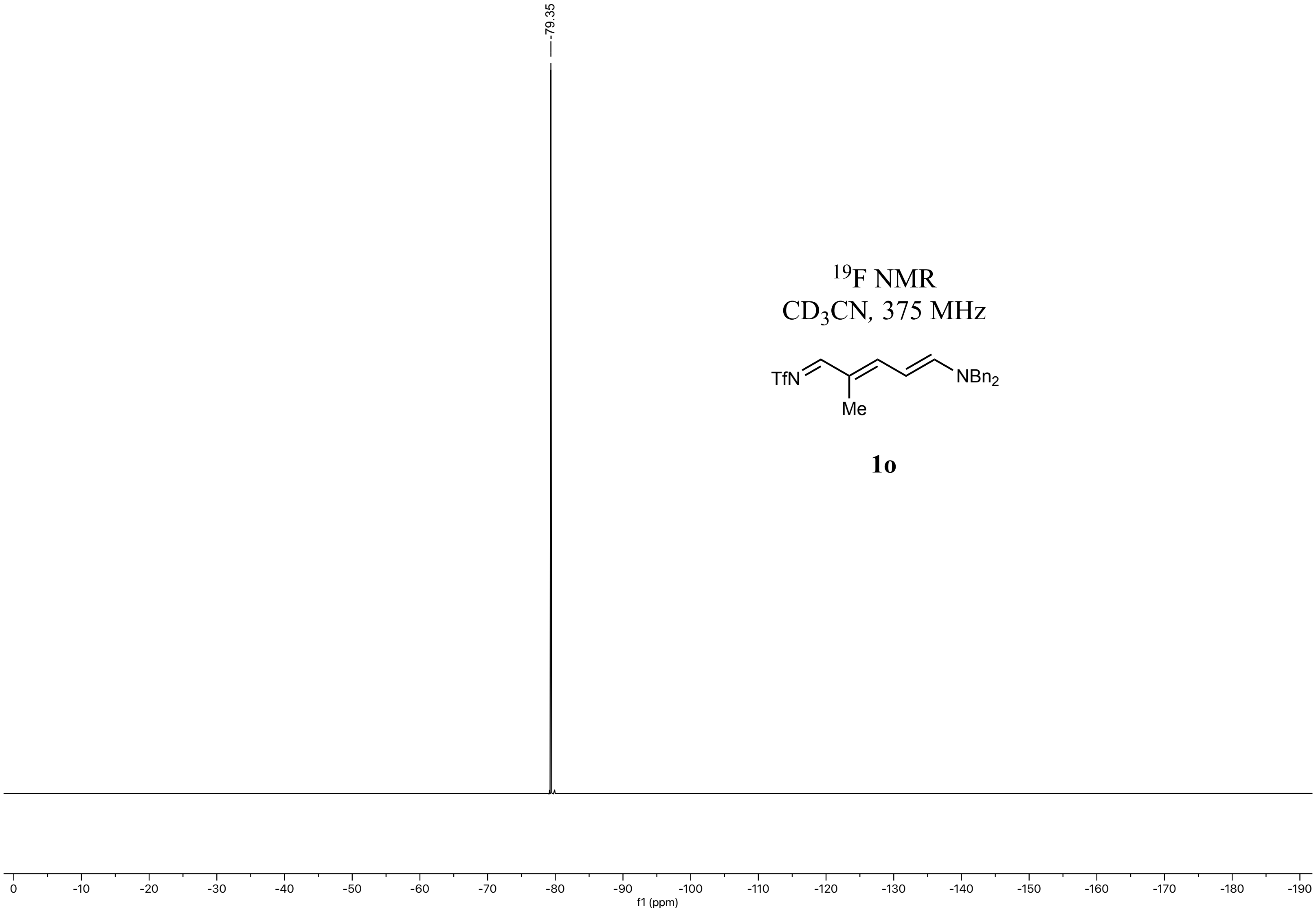
-79.35
^{19}F NMR
CD3CN, 375 MHz
TfN
NBn2
Me
1o
0
-10
-20
-30
-40
-50
-60
-70
-80
-90
-100
-110
-120
-130
-140
-150
-160
-170
-180
-190
f1 (ppm)

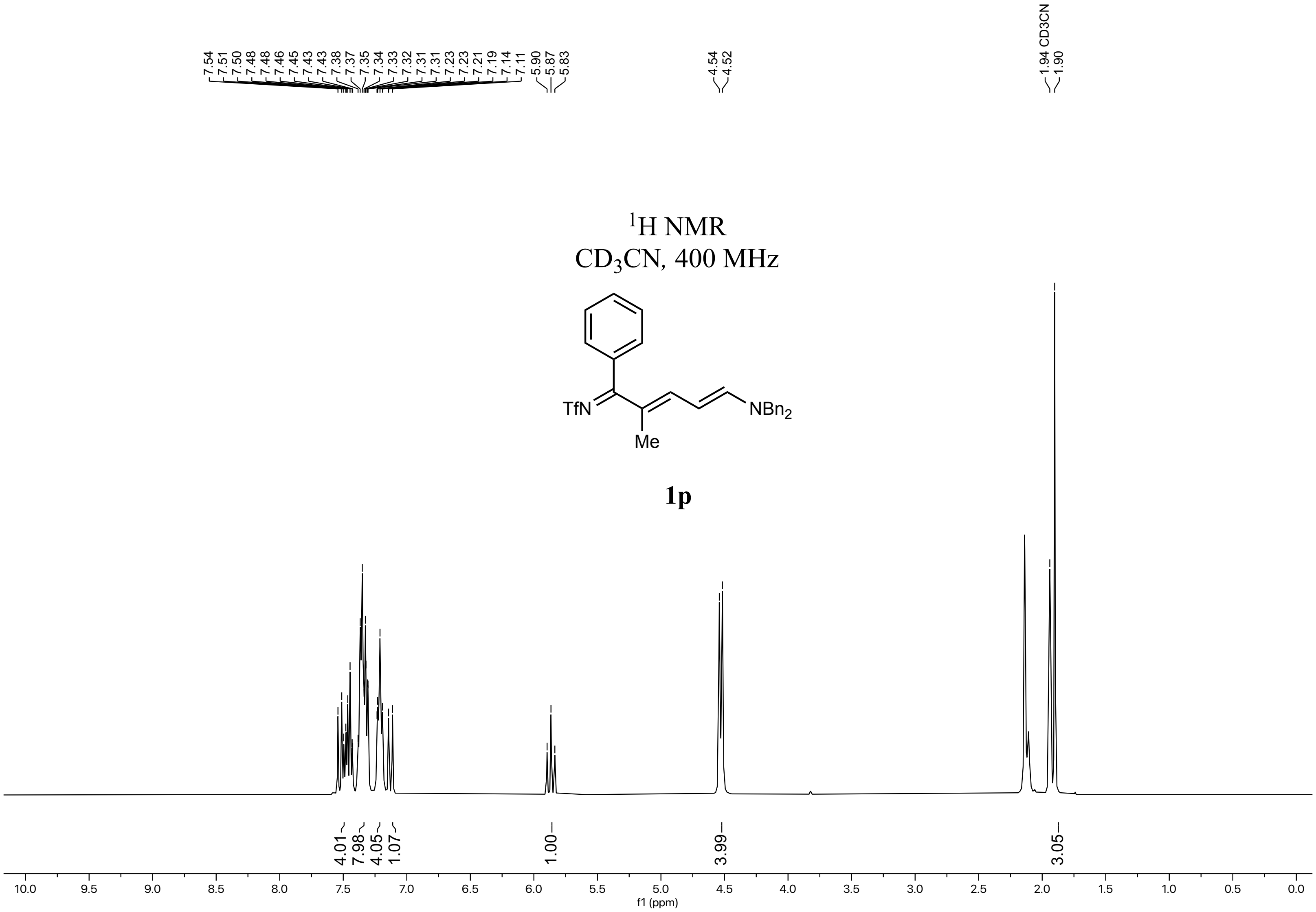

7.54
7.51
7.50
7.48
7.48
7.46
7.45
7.43
7.43
7.38
7.37
7.35
7.34
7.33
7.32
7.31
7.31
7.23
7.23
7.21
7.19
7.14
7.11
5.90
5.87
5.83
4.54
4.52
1.94 CD3CN
1.90
1H NMR
CD3CN, 400 MHz
TfN
NBn2
Me
1p
4.01
7.98
4.05
1.07
1.00
3.99
3.05
10.0
9.5
9.0
8.5
8.0
7.5
7.0
6.5
6.0
5.5
5.0
4.5
4.0
3.5
3.0
2.5
2.0
1.5
1.0
0.5
0.0
f1 (ppm)

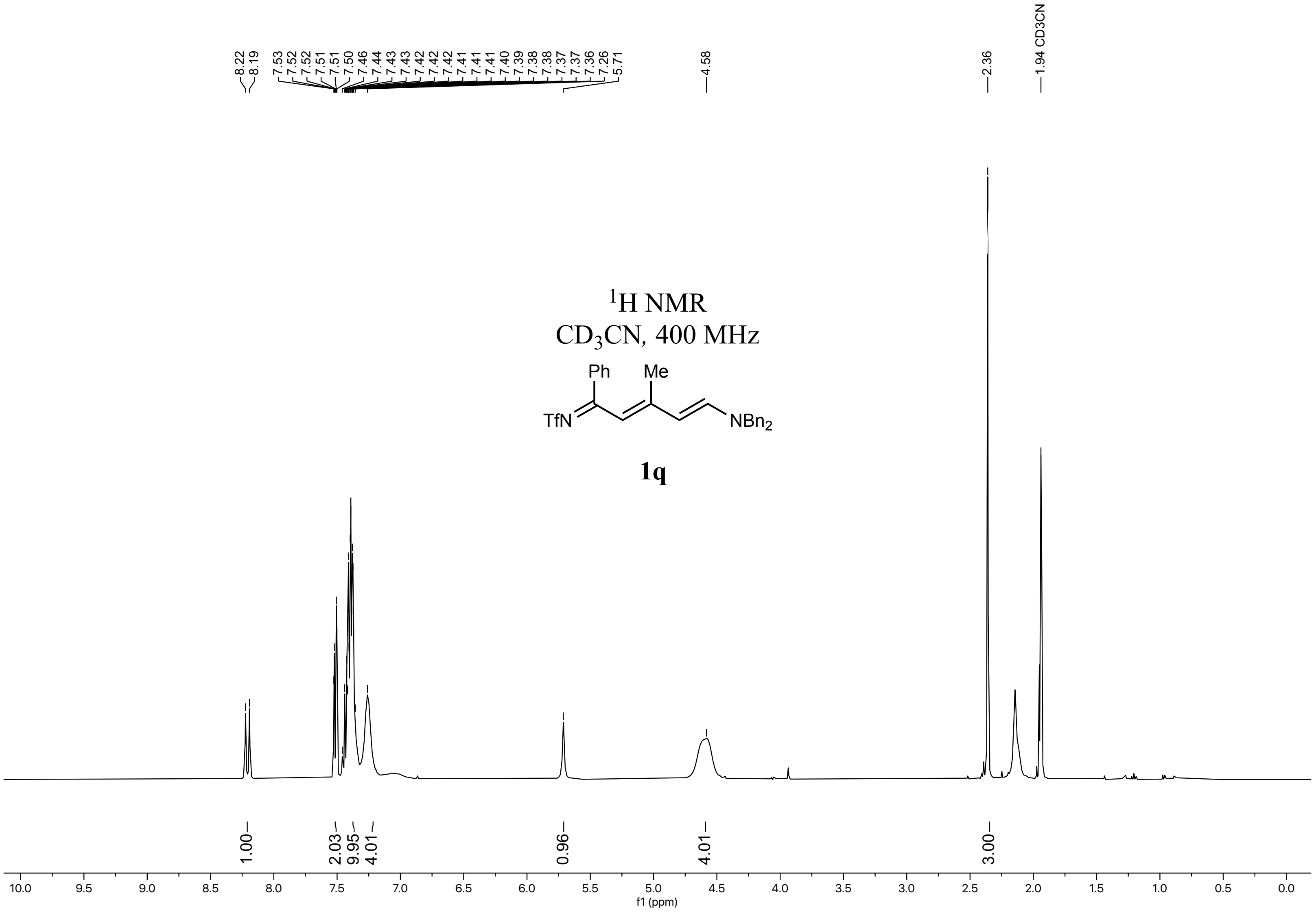
8.22
8.19
7.53
7.52
7.52
7.51
7.51
7.50
7.46
7.44
7.43
7.43
7.42
7.42
7.42
7.41
7.41
7.40
7.39
7.38
7.38
7.37
7.37
7.36
7.26
5.71
4.58
2.36
1.94 CD3CN
^{1}H NMR
CD_3CN, 400 MHz
Ph
Me
TfN
NBn_2
1q
1.00
2.03
9.95
4.01
0.96
4.01
3.00
10.0
9.5
9.0
8.5
8.0
7.5
7.0
6.5
6.0
5.5
5.0
4.5
4.0
3.5
3.0
2.5
2.0
1.5
1.0
0.5
0.0
f1 (ppm)

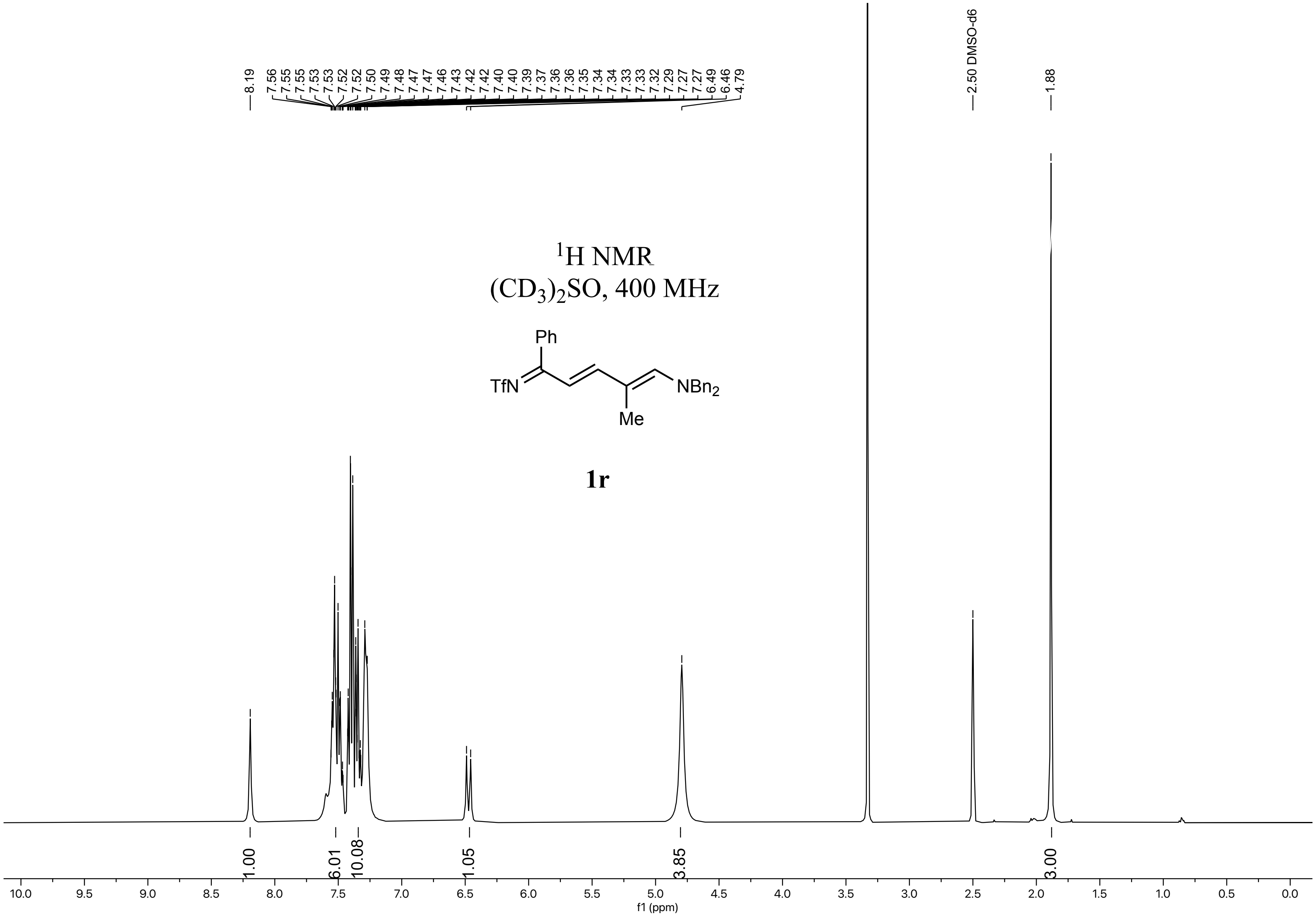

8.19
7.56
7.55
7.55
7.53
7.53
7.52
7.52
7.50
7.49
7.48
7.47
7.47
7.46
7.43
7.42
7.42
7.40
7.40
7.39
7.37
7.36
7.36
7.35
7.34
7.34
7.33
7.33
7.32
7.29
7.27
7.27
6.49
6.46
4.79
2.50 DMSO-d6
1.88
^{1}H NMR
$(CD_3)_2SO$, 400 MHz
Ph
TfN
NBn$_2$
Me
1r
1.00
6.01
10.08
1.05
3.85
3.00
10.0
9.5
9.0
8.5
8.0
7.5
7.0
6.5
6.0
5.5
5.0
4.5
4.0
3.5
3.0
2.5
2.0
1.5
1.0
0.5
0.0
f1 (ppm)

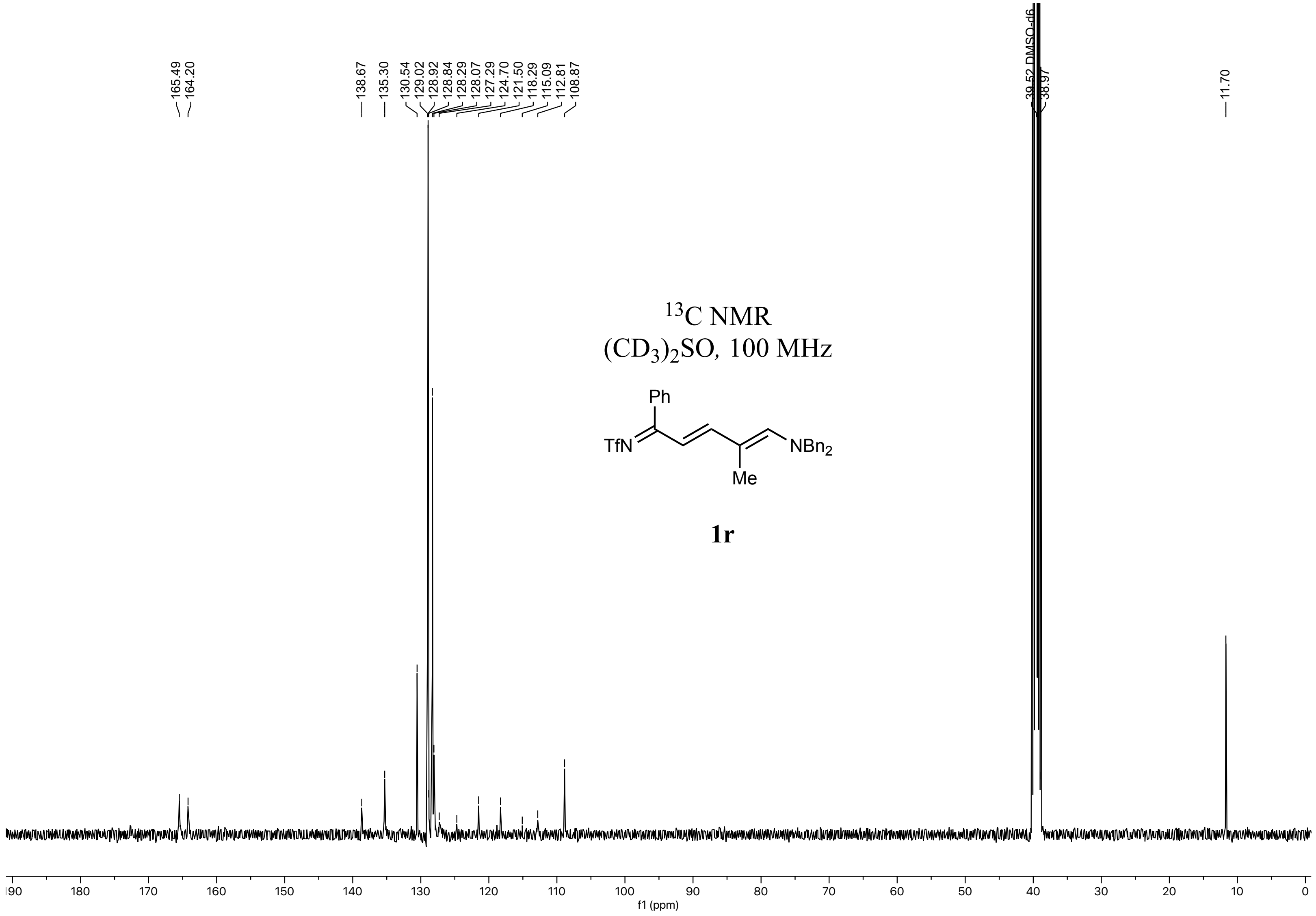

165.49
164.20
138.67
135.30
130.54
129.02
128.92
128.84
128.29
128.07
127.29
124.70
121.50
118.29
115.09
112.81
108.87
39.52 DMSO-d6
38.97
11.70
^{13}C NMR
$(CD_3)_2SO$, 100 MHz
Ph
TfN
NBn2
Me
1r
f1 (ppm)

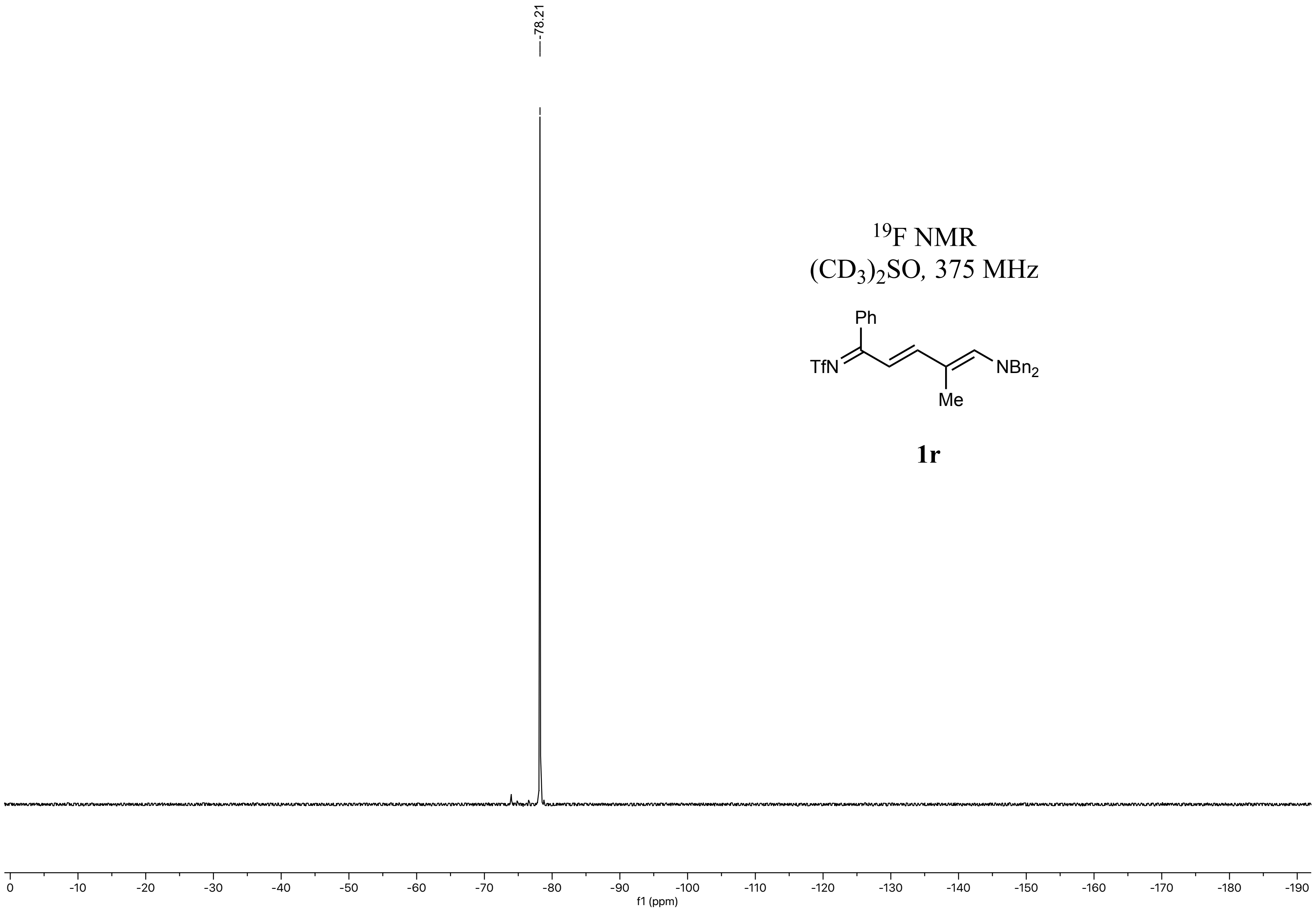

^{19}F NMR
$(CD_3)_2SO$, 375 MHz

1r

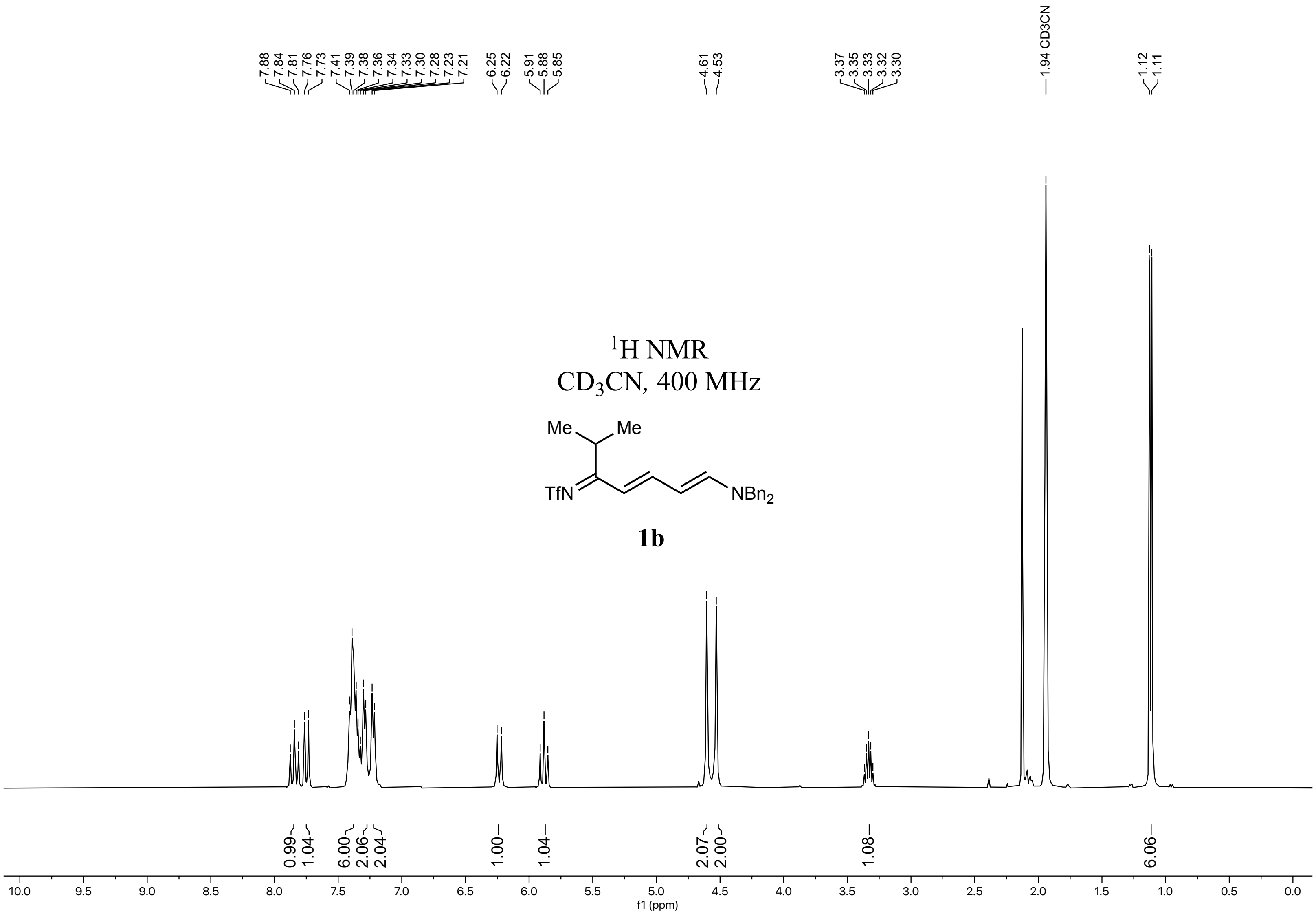
1H NMR
CD3CN, 400 MHz
Me
Me
TfN
NBn2
1b
1.94 CD3CN
f1 (ppm)

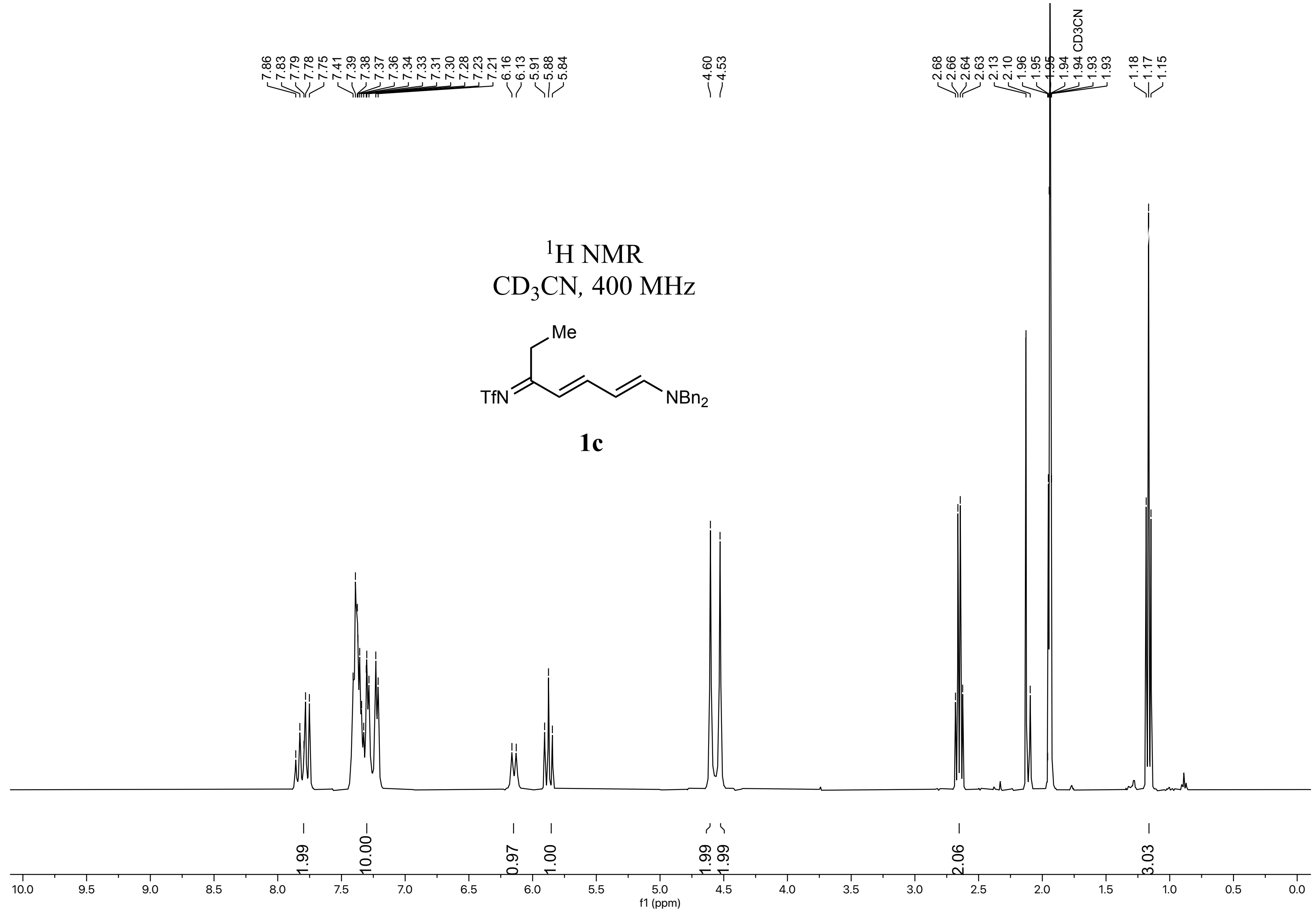

7.86
7.83
7.79
7.78
7.75
7.41
7.39
7.38
7.37
7.36
7.34
7.33
7.31
7.30
7.28
7.23
7.21
6.16
6.13
5.91
5.88
5.84
4.60
4.53
2.68
2.66
2.64
2.63
2.13
2.10
1.96
1.95
1.95
1.94
1.94 CD3CN
1.93
1.93
1.18
1.17
1.15
1H NMR
CD3CN, 400 MHz
Me
TfN
NBn2
1c
1.99
10.00
0.97
1.00
1.99
1.99
2.06
3.03
10.0
9.5
9.0
8.5
8.0
7.5
7.0
6.5
6.0
5.5
5.0
4.5
4.0
3.5
3.0
2.5
2.0
1.5
1.0
0.5
0.0
f1 (ppm)

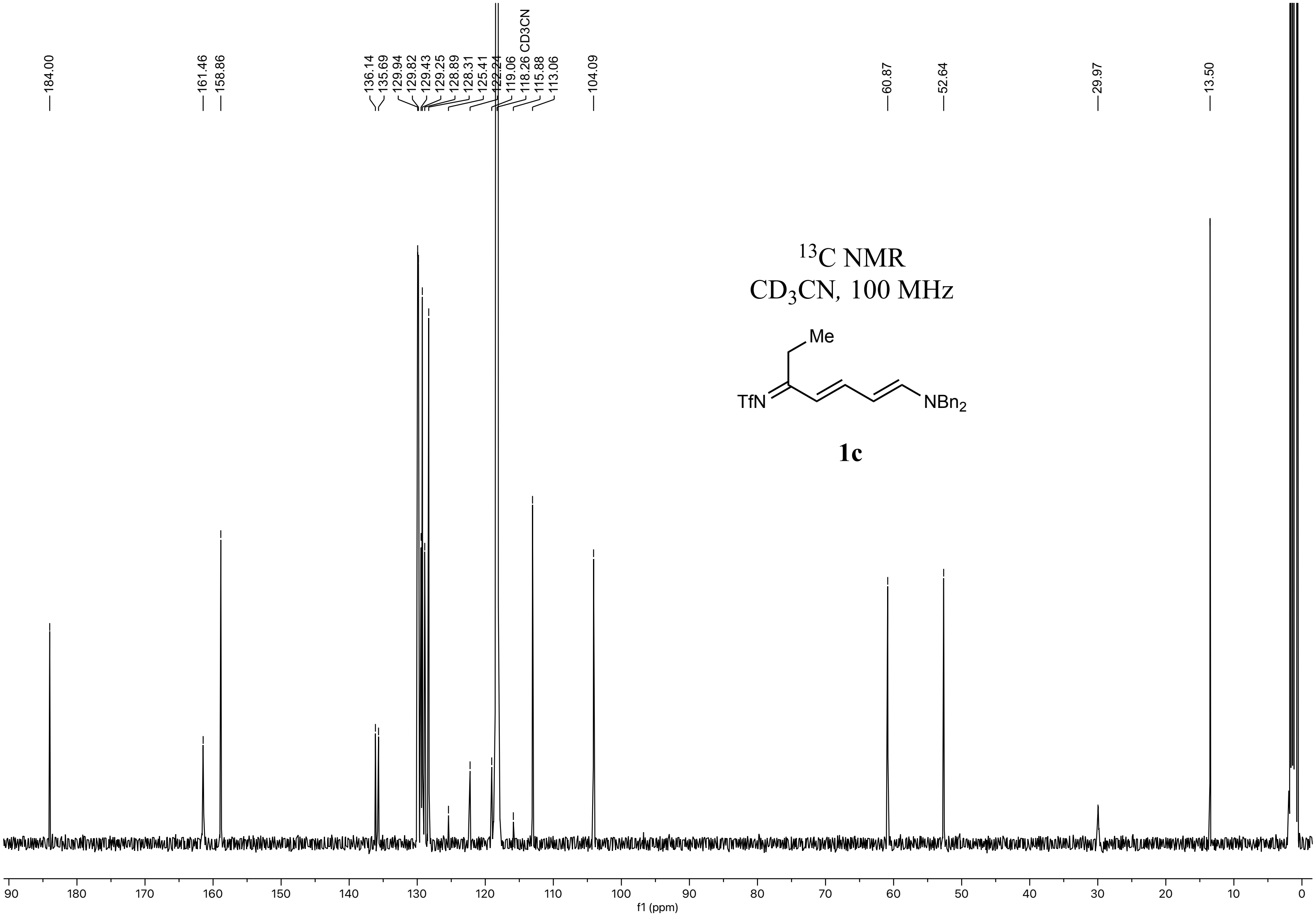
184.00
161.46
158.86
136.14
135.69
129.94
129.82
129.43
129.25
128.89
128.31
125.41
122.24
119.06
118.26 CD3CN
115.88
113.06
104.09
60.87
52.64
29.97
13.50
^{13}C NMR
CD_3CN, 100 MHz
Me
TfN
NBn_2
1c
f1 (ppm)

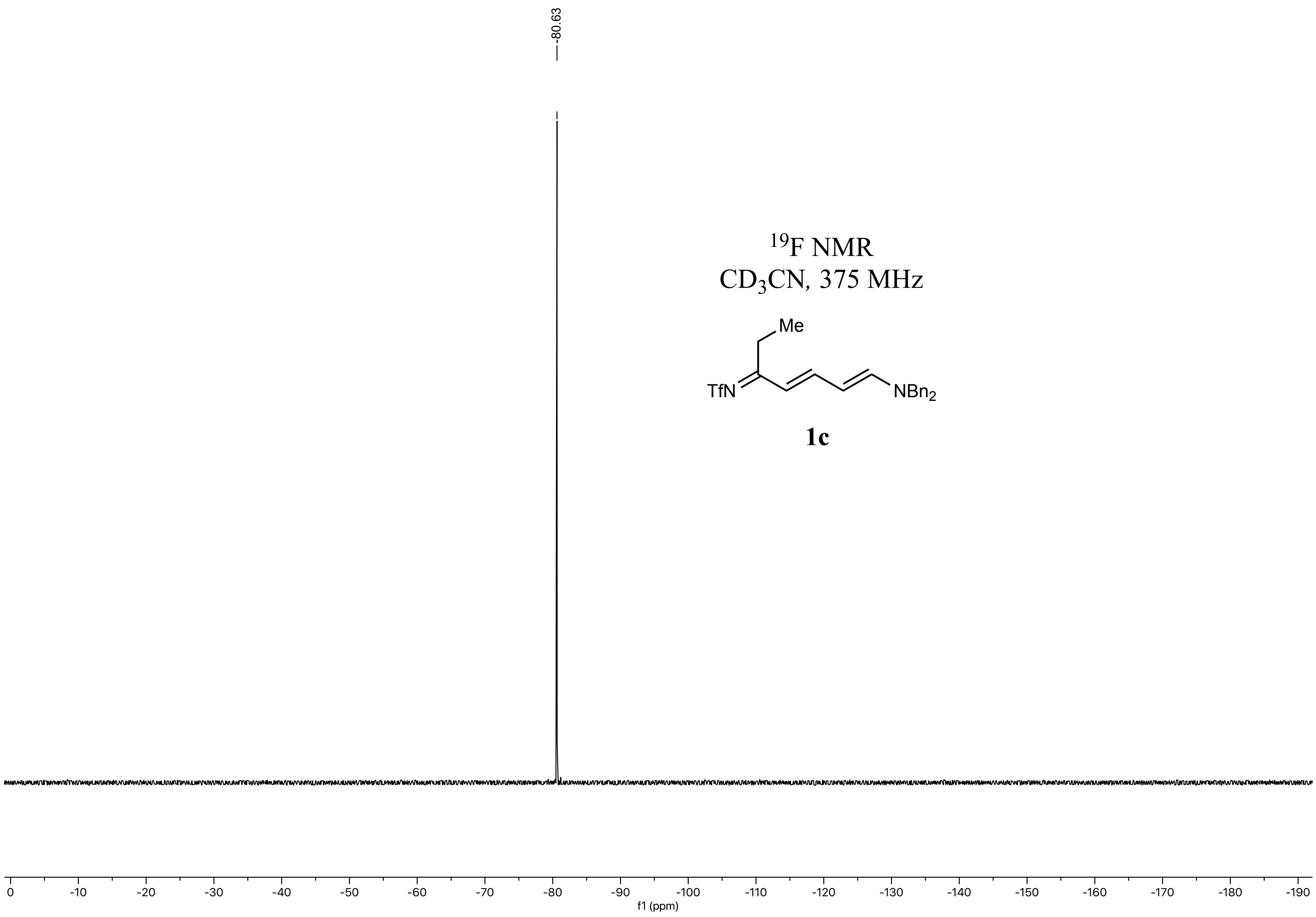

^{19}F NMR
CD_3CN, 375 MHz

1c

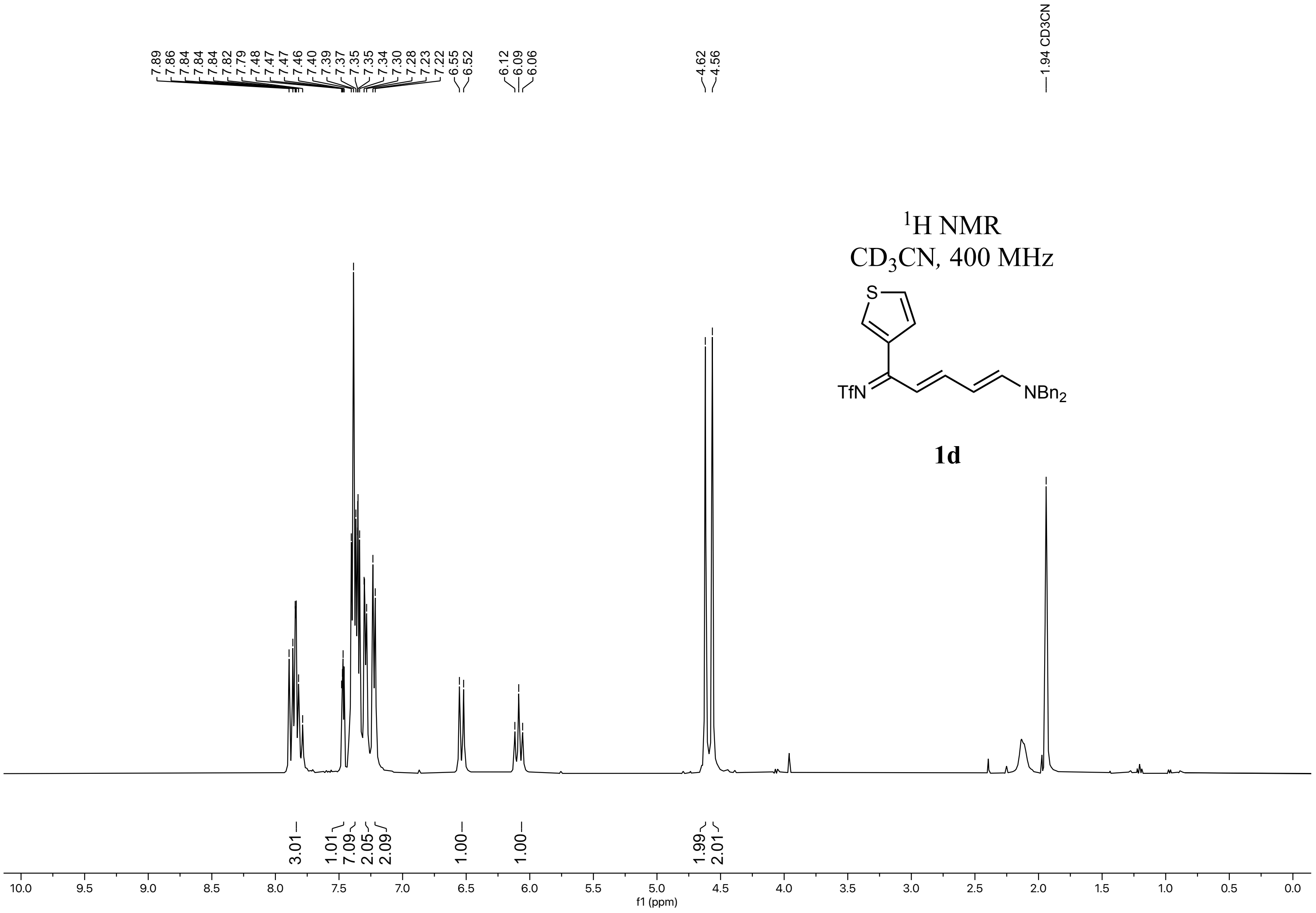
1H NMR
CD3CN, 400 MHz
TfN
NBn2
1d
1.94 CD3CN
f1 (ppm)

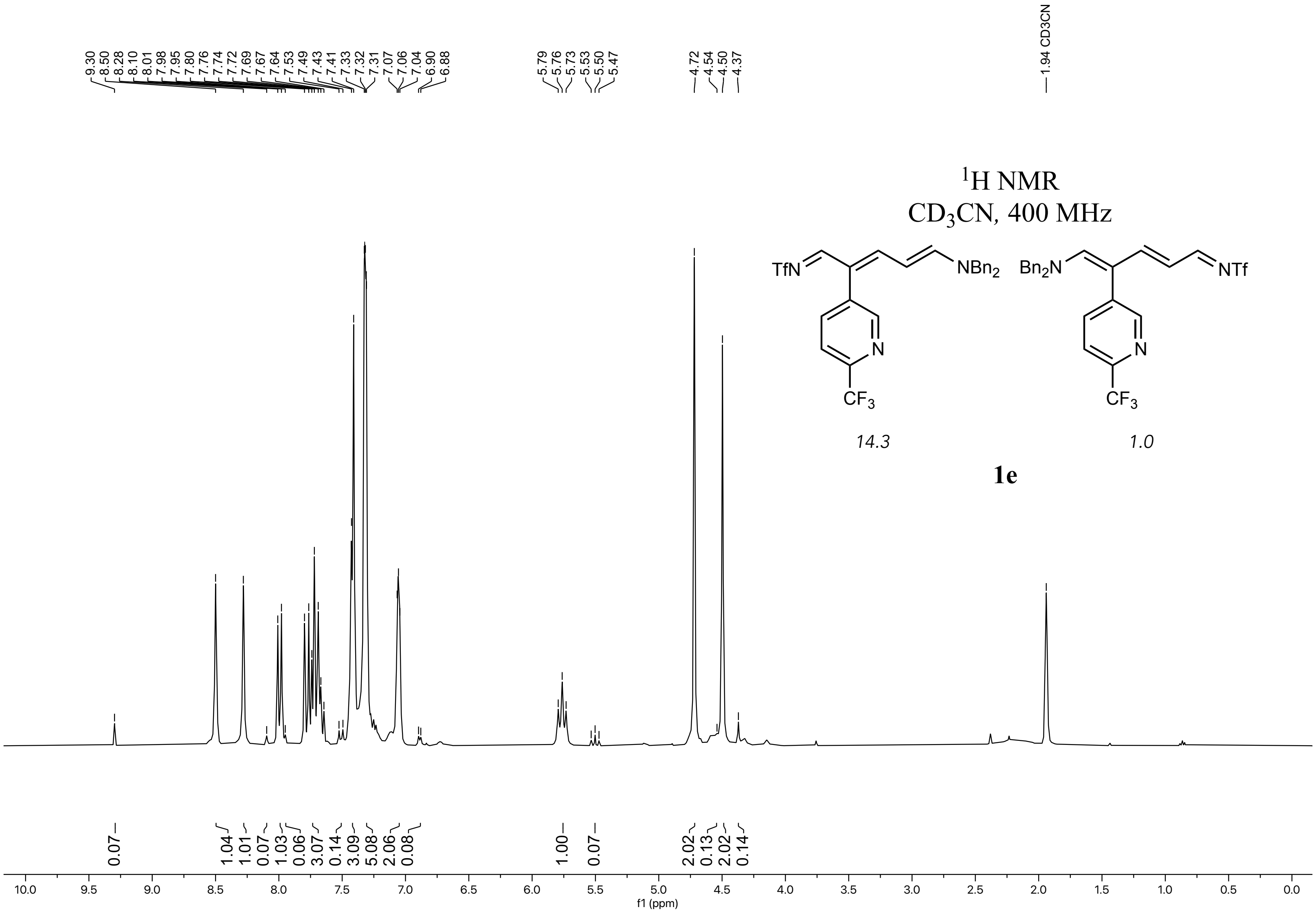

^{1}H NMR

CD_3CN, 400 MHz

14.3 *1.0*

1e

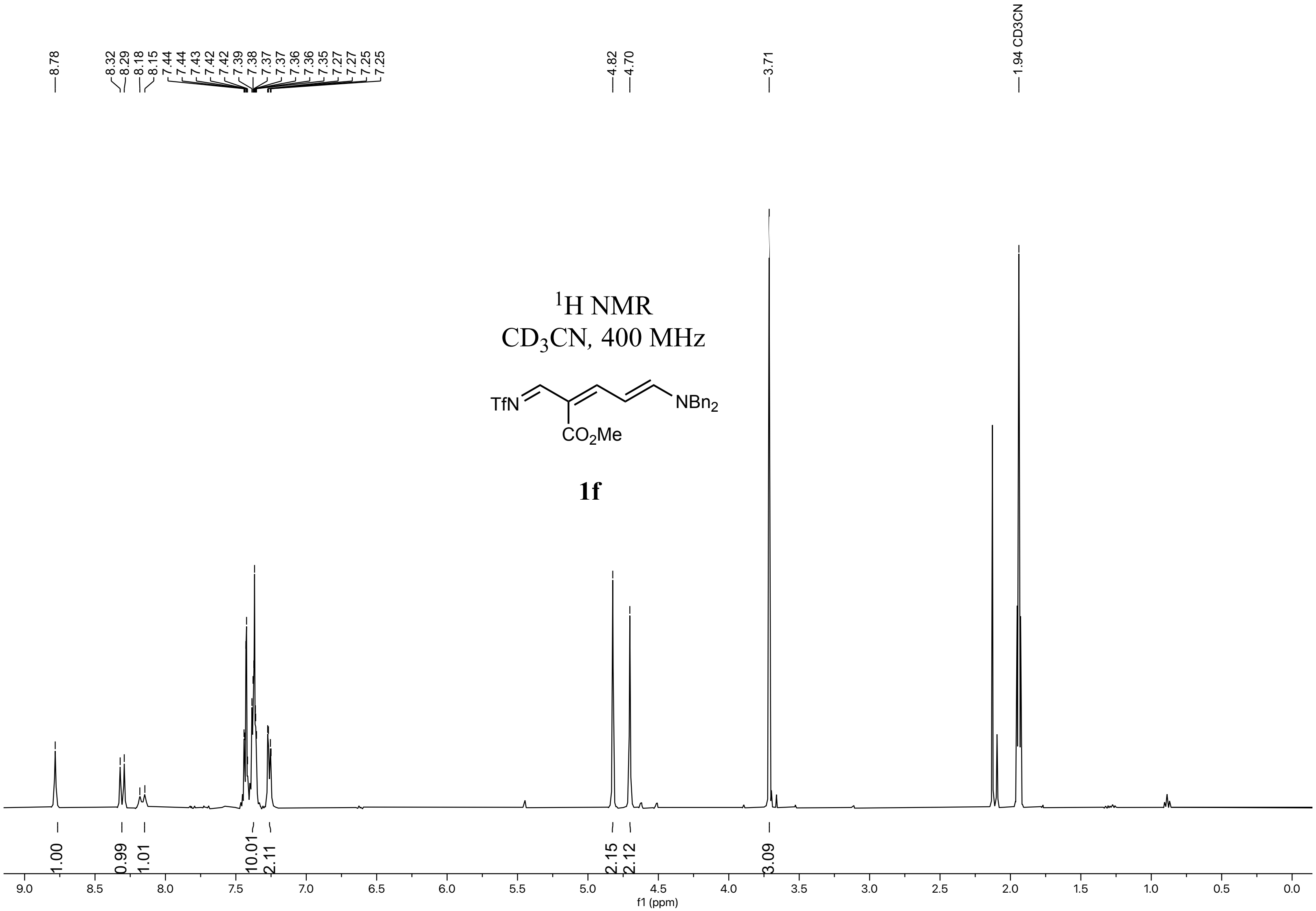
8.78
8.32
8.29
8.18
8.15
7.44
7.44
7.43
7.42
7.42
7.39
7.38
7.37
7.37
7.36
7.36
7.35
7.27
7.27
7.25
7.25
4.82
4.70
3.71
1.94 CD3CN
1H NMR
CD3CN, 400 MHz
TfN
NBn2
CO2Me
1f
1.00
0.99
1.01
10.01
2.11
2.15
2.12
3.09
9.0
8.5
8.0
7.5
7.0
6.5
6.0
5.5
5.0
4.5
4.0
3.5
3.0
2.5
2.0
1.5
1.0
0.5
0.0
f1 (ppm)

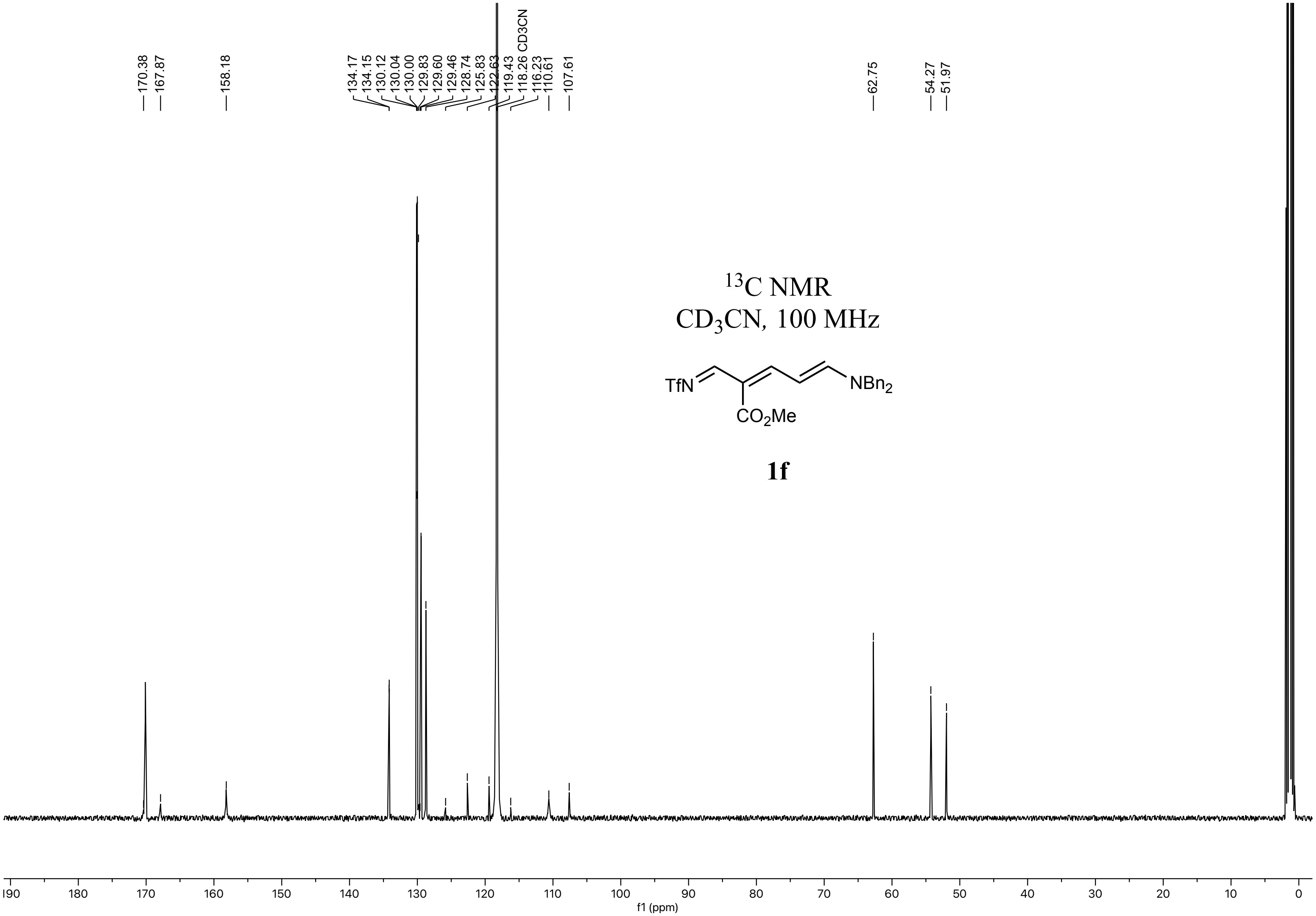
170.38
167.87
158.18
134.17
134.15
130.12
130.04
130.00
129.83
129.60
129.46
128.74
125.83
122.63
119.43
118.26 CD3CN
116.23
110.61
107.61
62.75
54.27
51.97
^{13}C NMR
CD_3CN, 100 MHz
TfN
NBn_2
CO_2Me
1f
190
180
170
160
150
140
130
120
110
100
90
80
70
60
50
40
30
20
10
0
f1 (ppm)

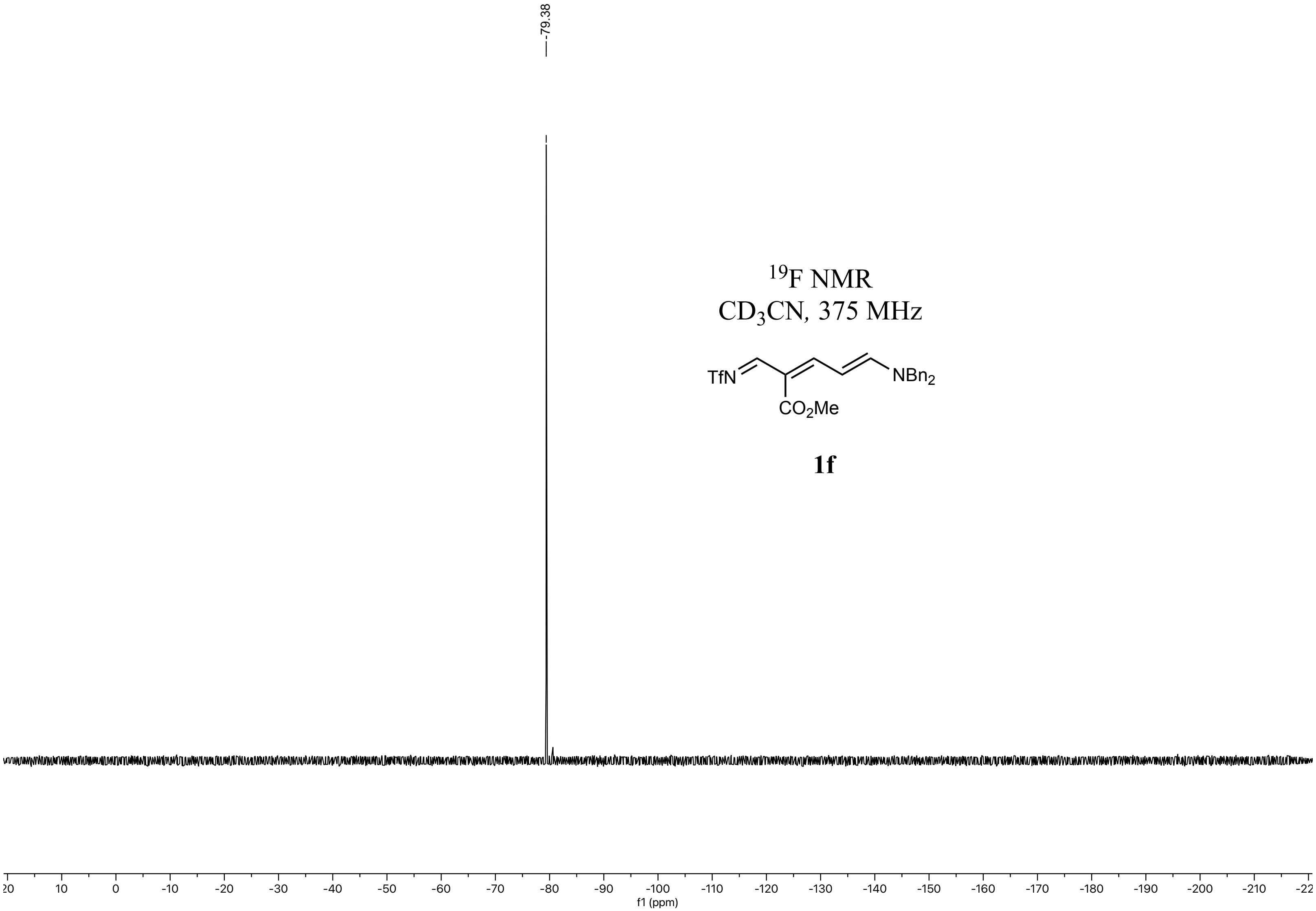

^{19}F NMR
CD_3CN, 375 MHz

1f

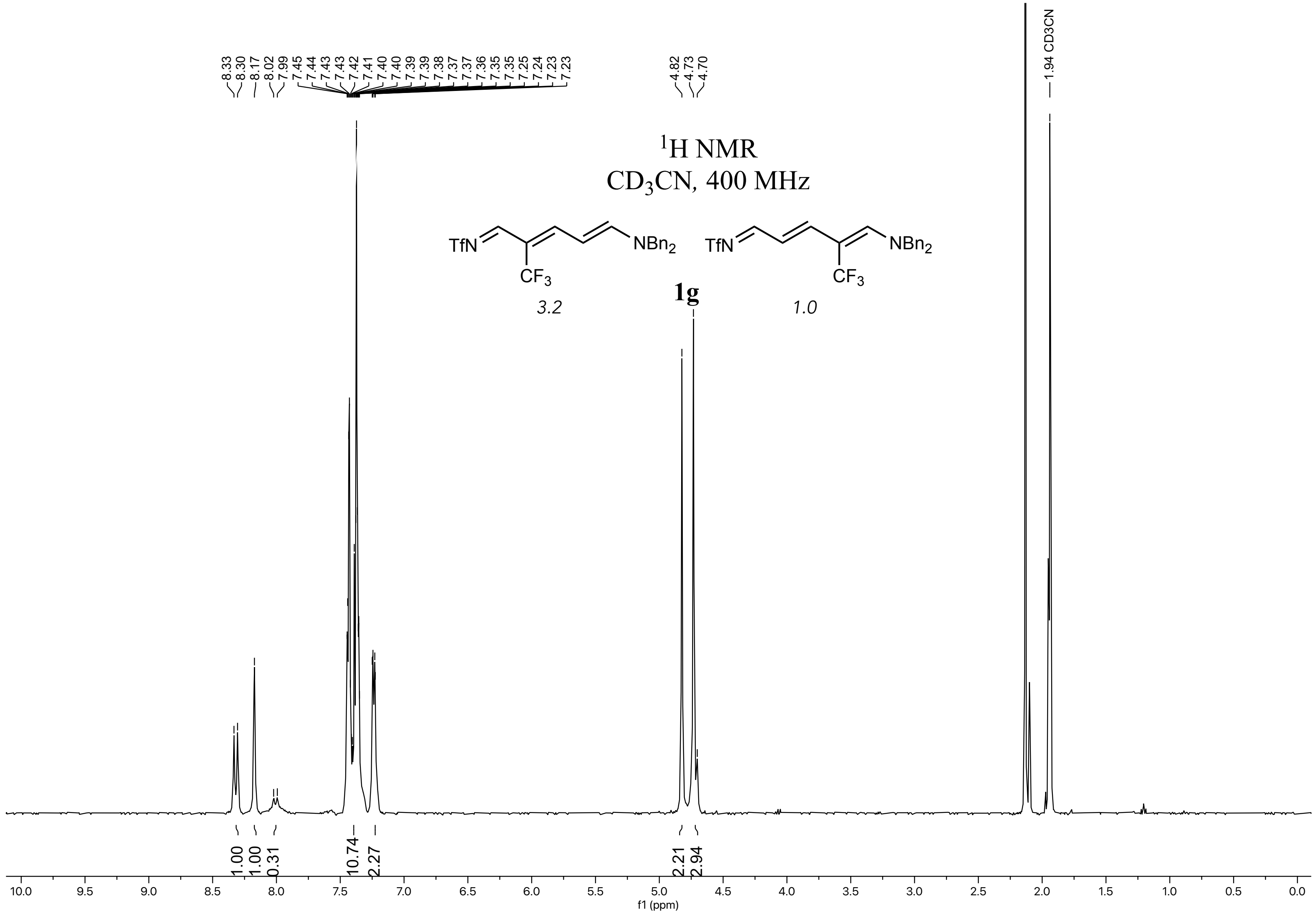
8.33
8.30
8.17
8.02
7.99
7.45
7.44
7.43
7.43
7.42
7.41
7.40
7.40
7.39
7.39
7.38
7.37
7.37
7.36
7.35
7.35
7.25
7.24
7.23
7.23
4.82
4.73
4.70
1.94 CD3CN
1H NMR
CD3CN, 400 MHz
TfN
NBn2
CF3
3.2
1g
TfN
NBn2
CF3
1.0
1.00
1.00
0.31
10.74
2.27
2.21
2.94
10.0
9.5
9.0
8.5
8.0
7.5
7.0
6.5
6.0
5.5
5.0
4.5
4.0
3.5
3.0
2.5
2.0
1.5
1.0
0.5
0.0
f1 (ppm)

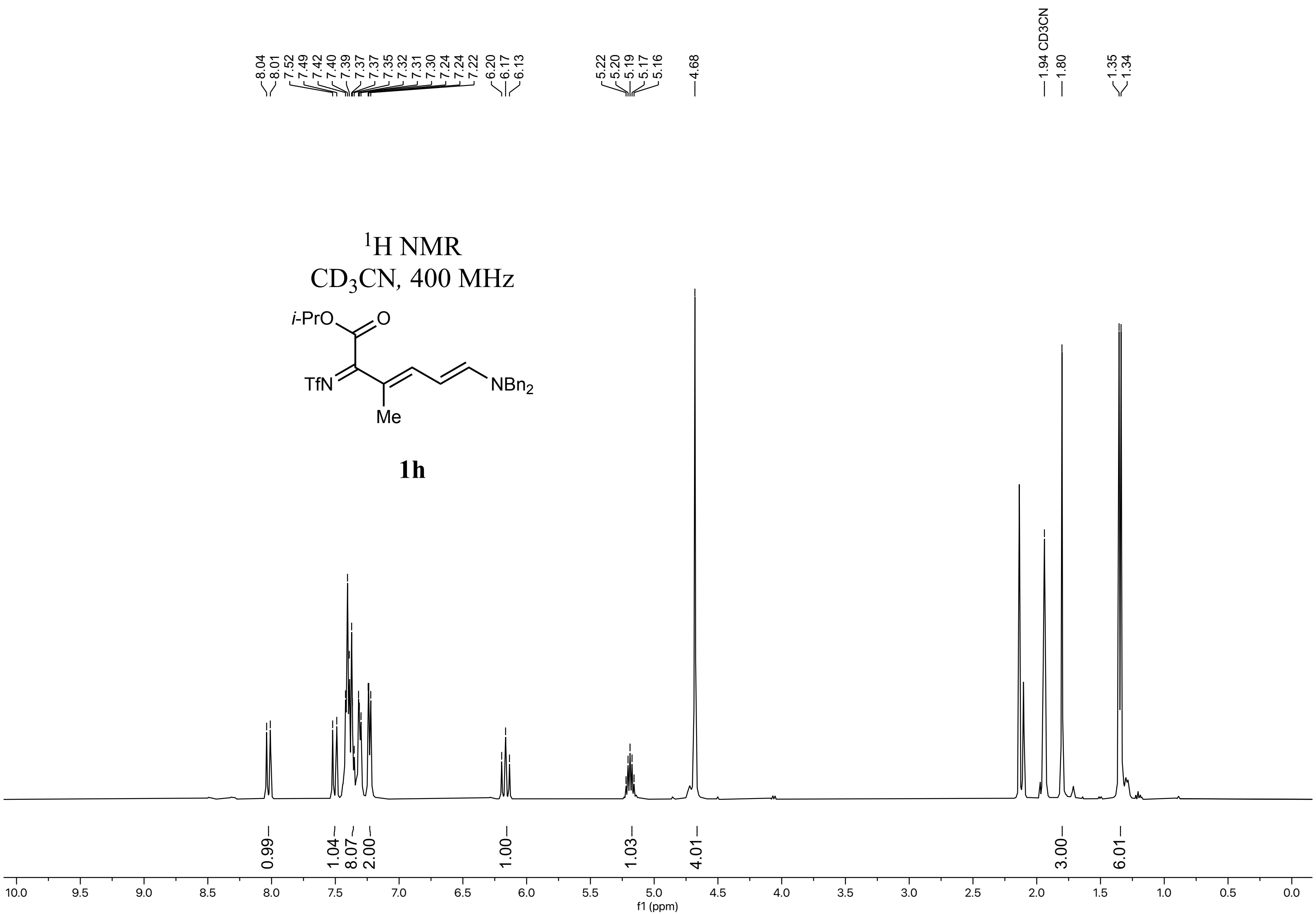
8.04
8.01
7.52
7.49
7.42
7.40
7.39
7.37
7.37
7.35
7.32
7.31
7.30
7.24
7.24
7.22
6.20
6.17
6.13
5.22
5.20
5.19
5.17
5.16
4.68
1.94 CD3CN
1.80
1.35
1.34
^{1}H NMR
CD_3CN, 400 MHz
i-PrO
O
TfN
NBn2
Me
1h
0.99
1.04
8.07
2.00
1.00
1.03
4.01
3.00
6.01
10.0
9.5
9.0
8.5
8.0
7.5
7.0
6.5
6.0
5.5
5.0
4.5
4.0
3.5
3.0
2.5
2.0
1.5
1.0
0.5
0.0
f1 (ppm)

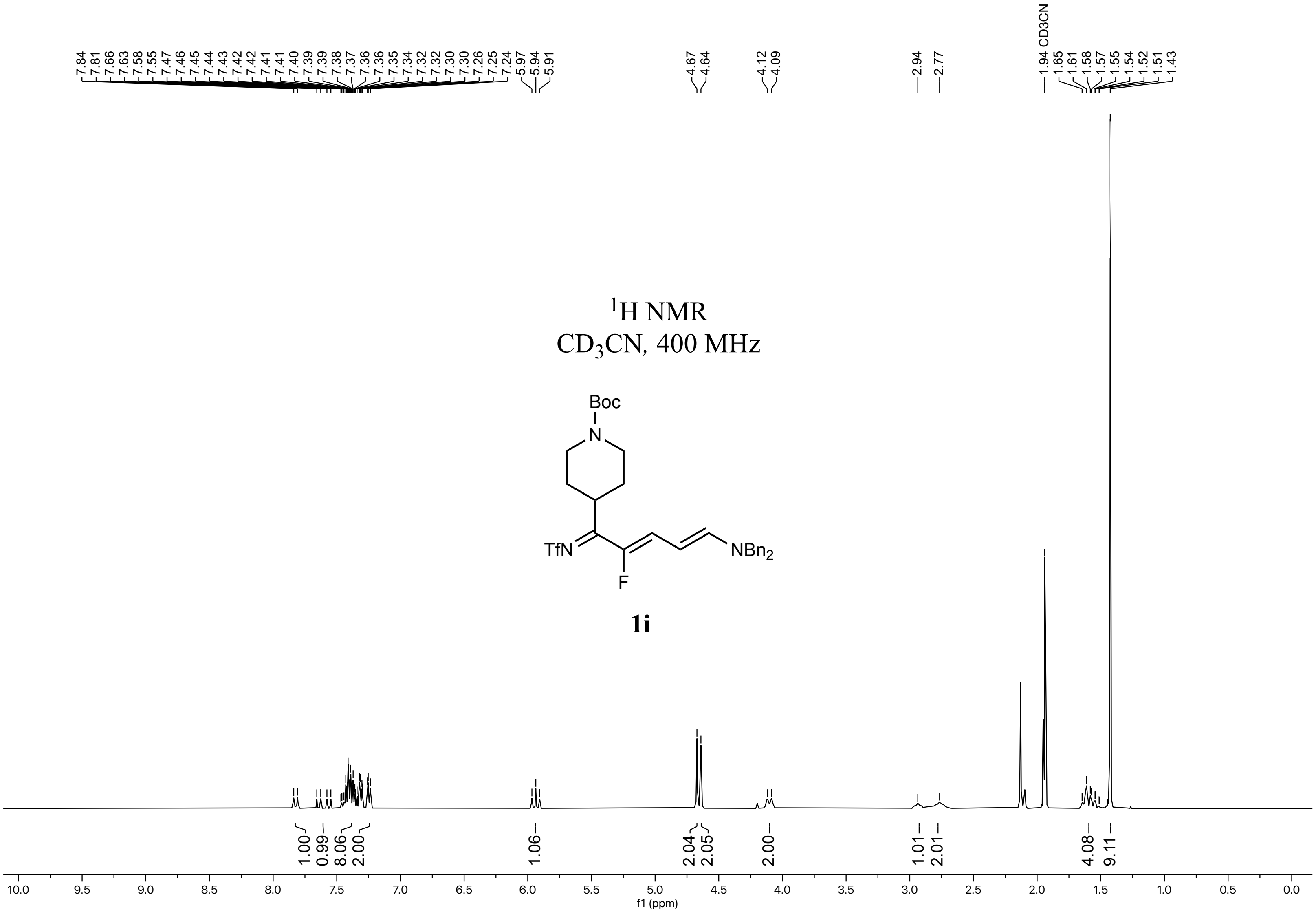

1H NMR
CD3CN, 400 MHz
Boc
N
TfN
F
NBn2
1i
1.94 CD3CN
f1 (ppm)

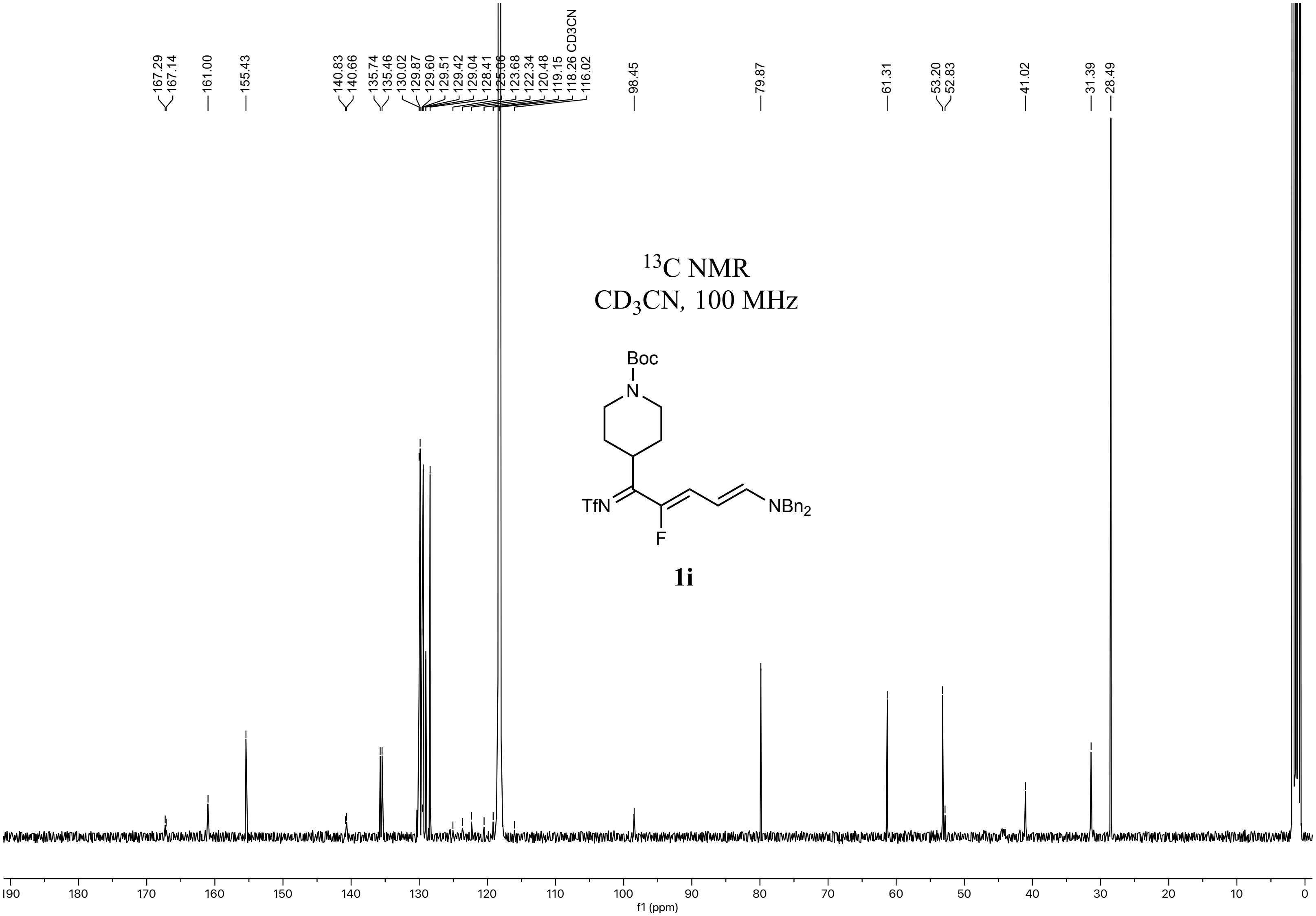

^{13}C NMR
CD_3CN, 100 MHz

1i

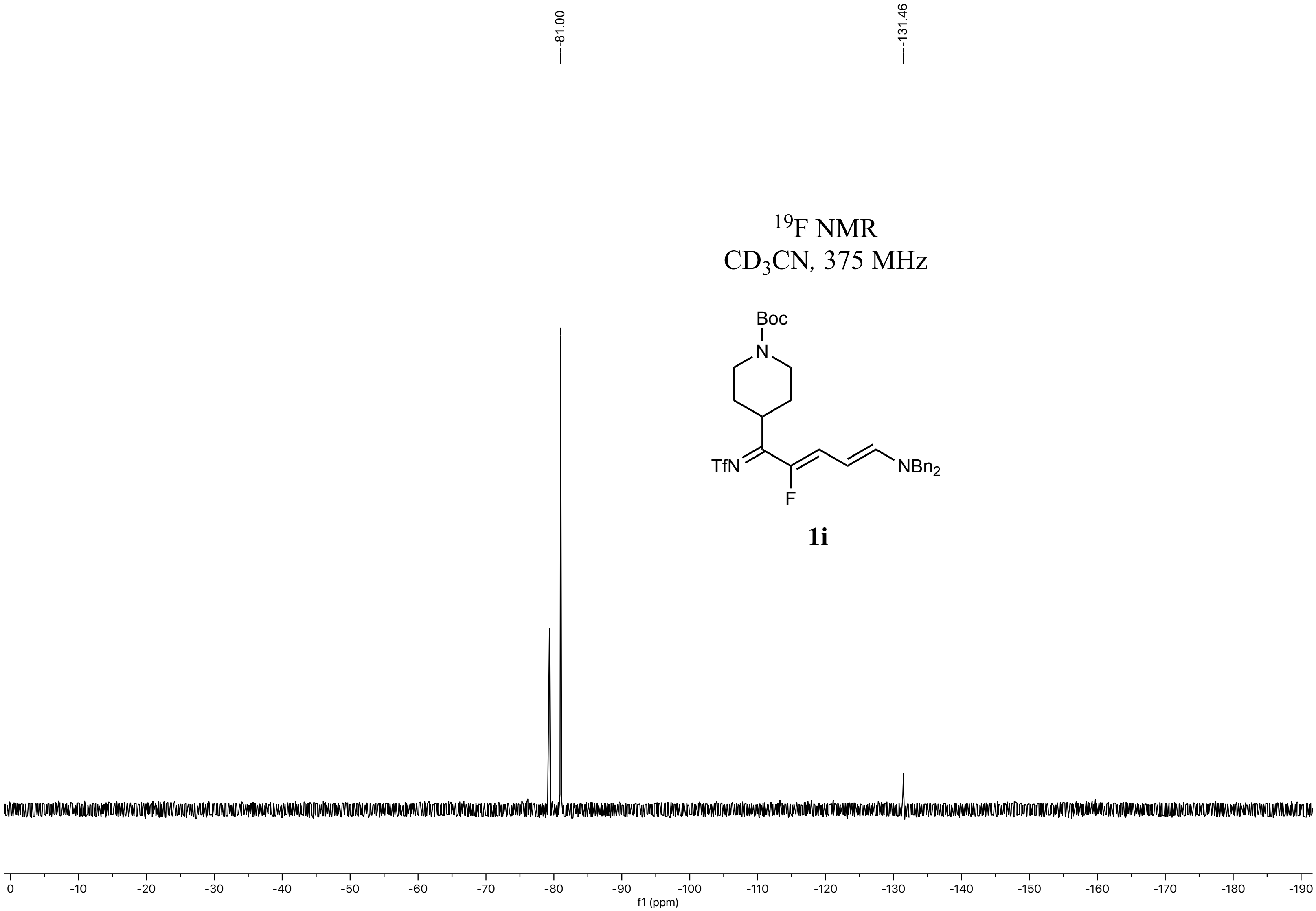
-81.00
-131.46
^{19}F NMR
CD_3CN, 375 MHz
Boc
N
TfN
F
NBn_2
1i
0 -10 -20 -30 -40 -50 -60 -70 -80 -90 -100 -110 -120 -130 -140 -150 -160 -170 -180 -190
f1 (ppm)

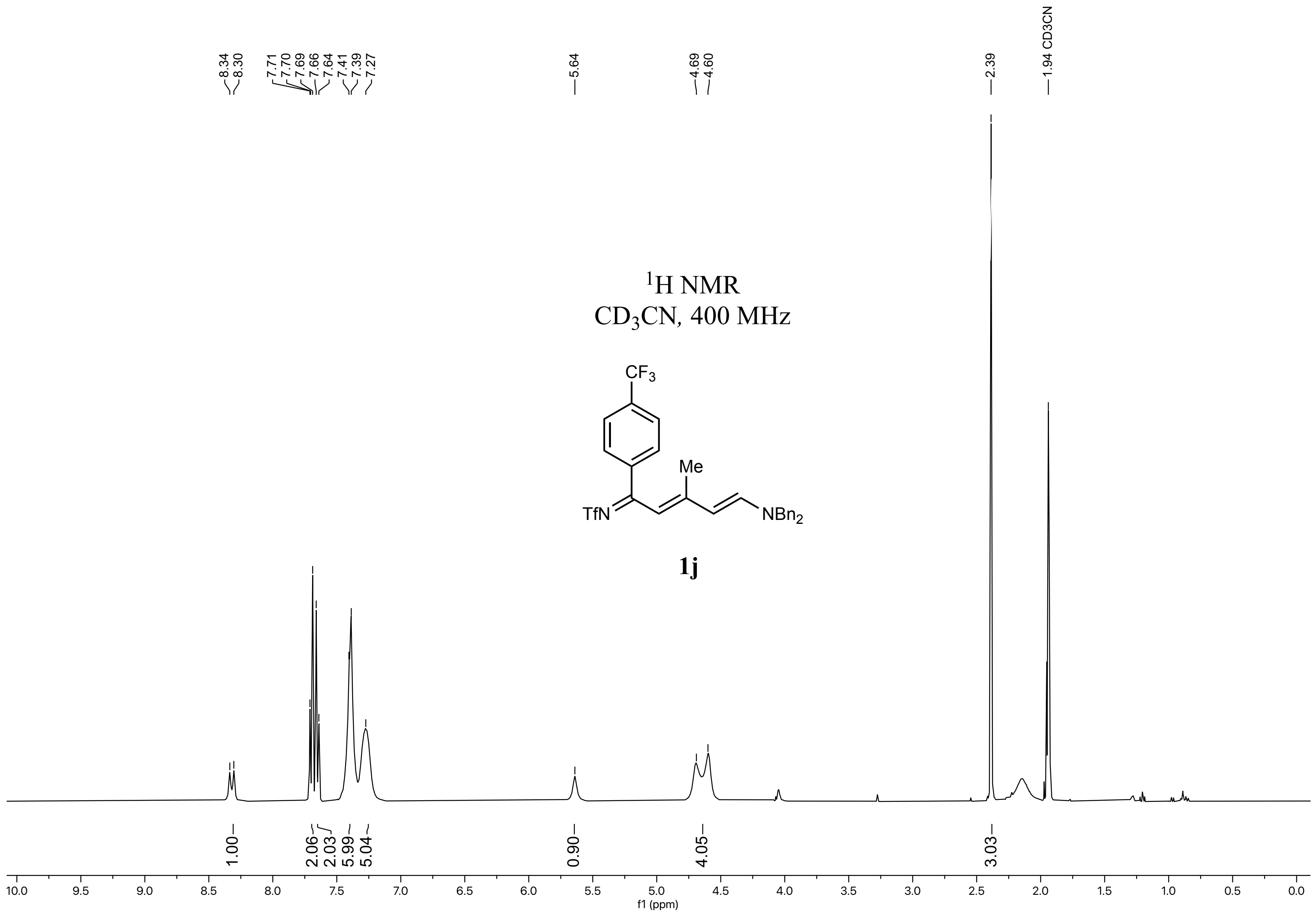
8.34
8.30
7.71
7.70
7.69
7.66
7.64
7.41
7.39
7.27
5.64
4.69
4.60
2.39
1.94 CD3CN
^{1}H NMR
CD_3CN, 400 MHz
CF_3
Me
TfN
NBn_2
1j
1.00
2.06
2.03
5.99
5.04
0.90
4.05
3.03
f1 (ppm)

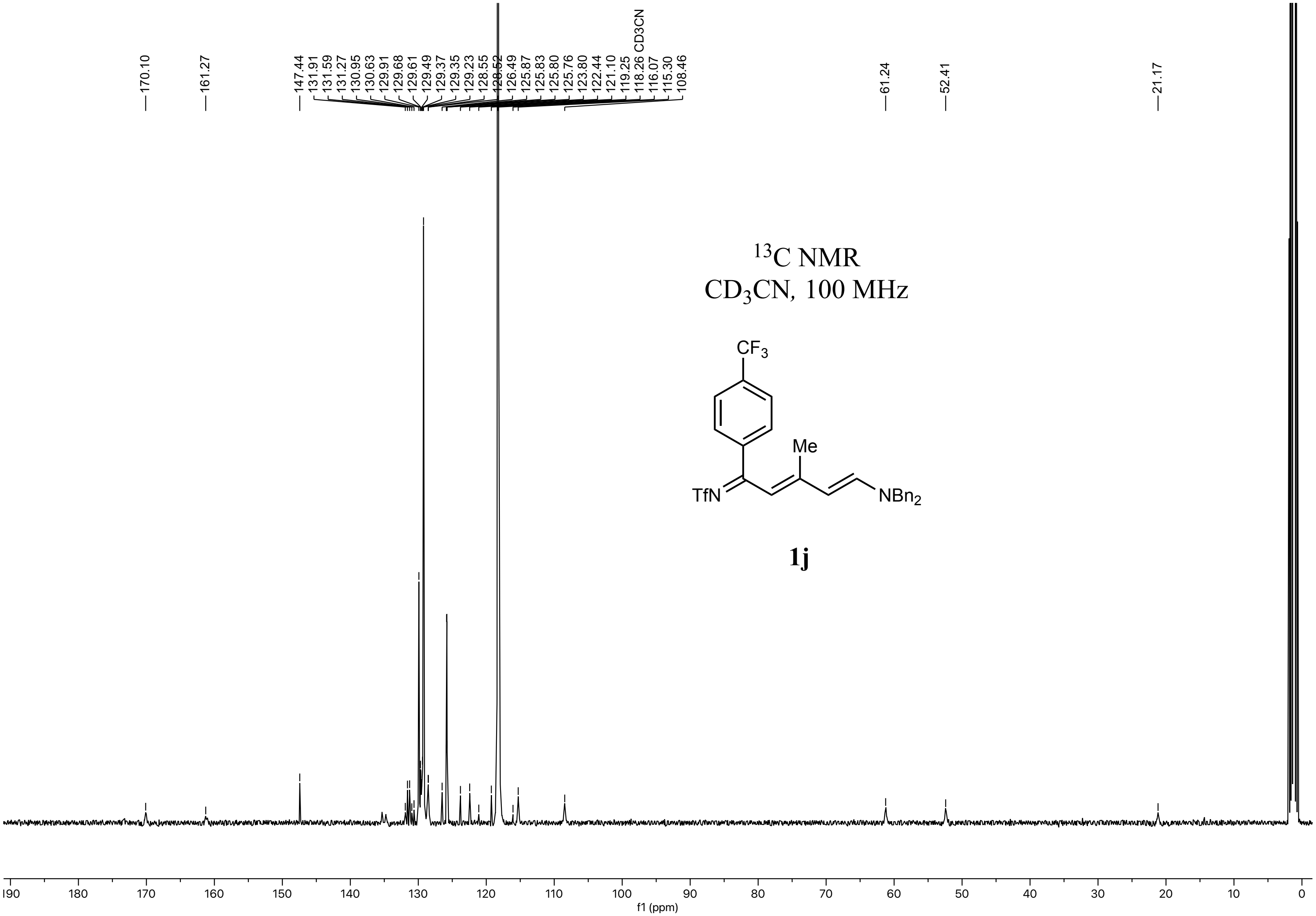
170.10
161.27
147.44
131.91
131.59
131.27
130.95
130.63
129.91
129.68
129.61
129.49
129.37
129.35
129.23
128.55
128.52
126.49
125.87
125.83
125.80
125.76
123.80
122.44
121.10
119.25
118.26 CD3CN
116.07
115.30
108.46
61.24
52.41
21.17
^{13}C NMR
CD_3CN, 100 MHz
CF_3
Me
TfN
NBn_2
1j
f1 (ppm)

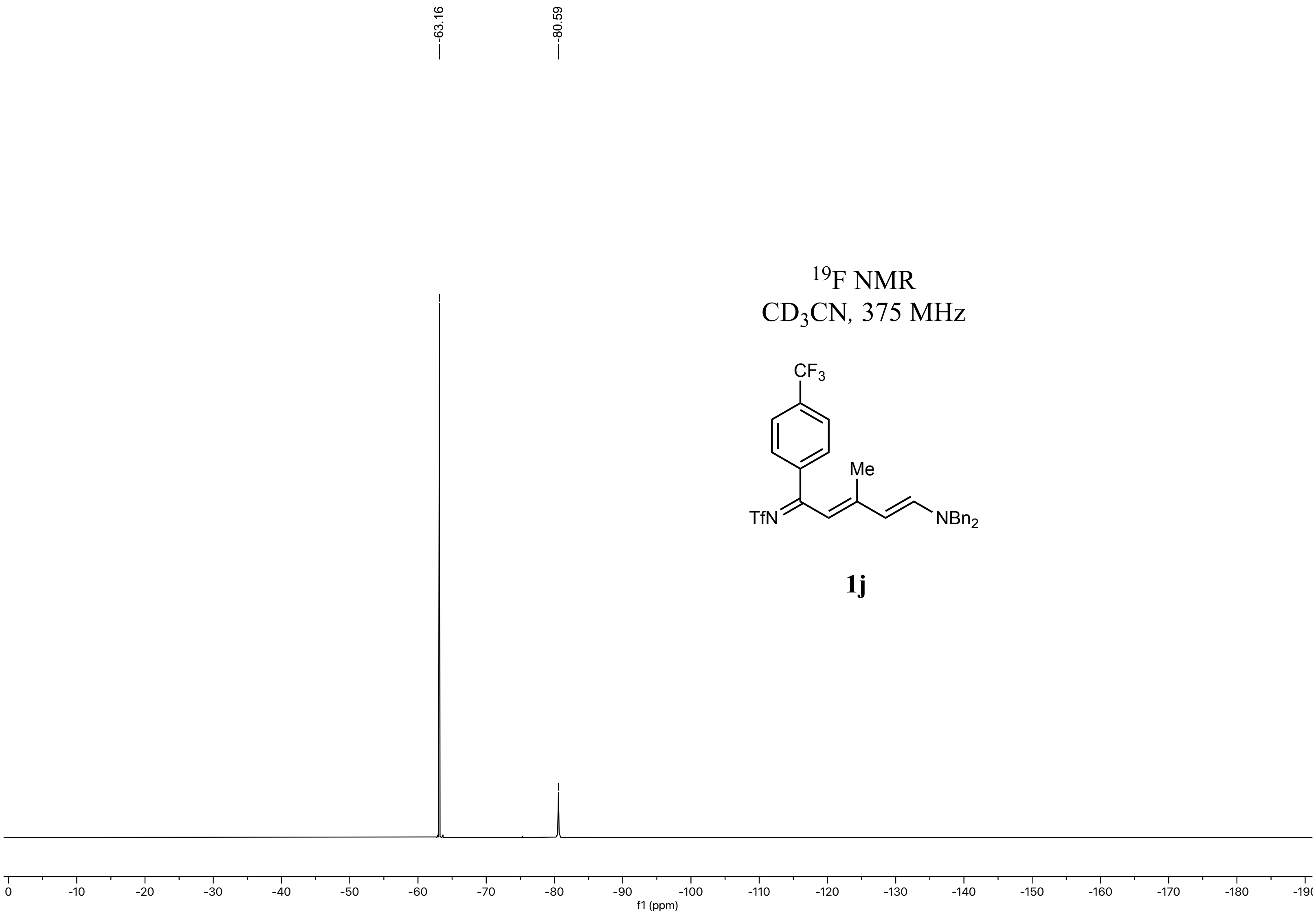

^{19}F NMR
CD_3CN, 375 MHz

1j

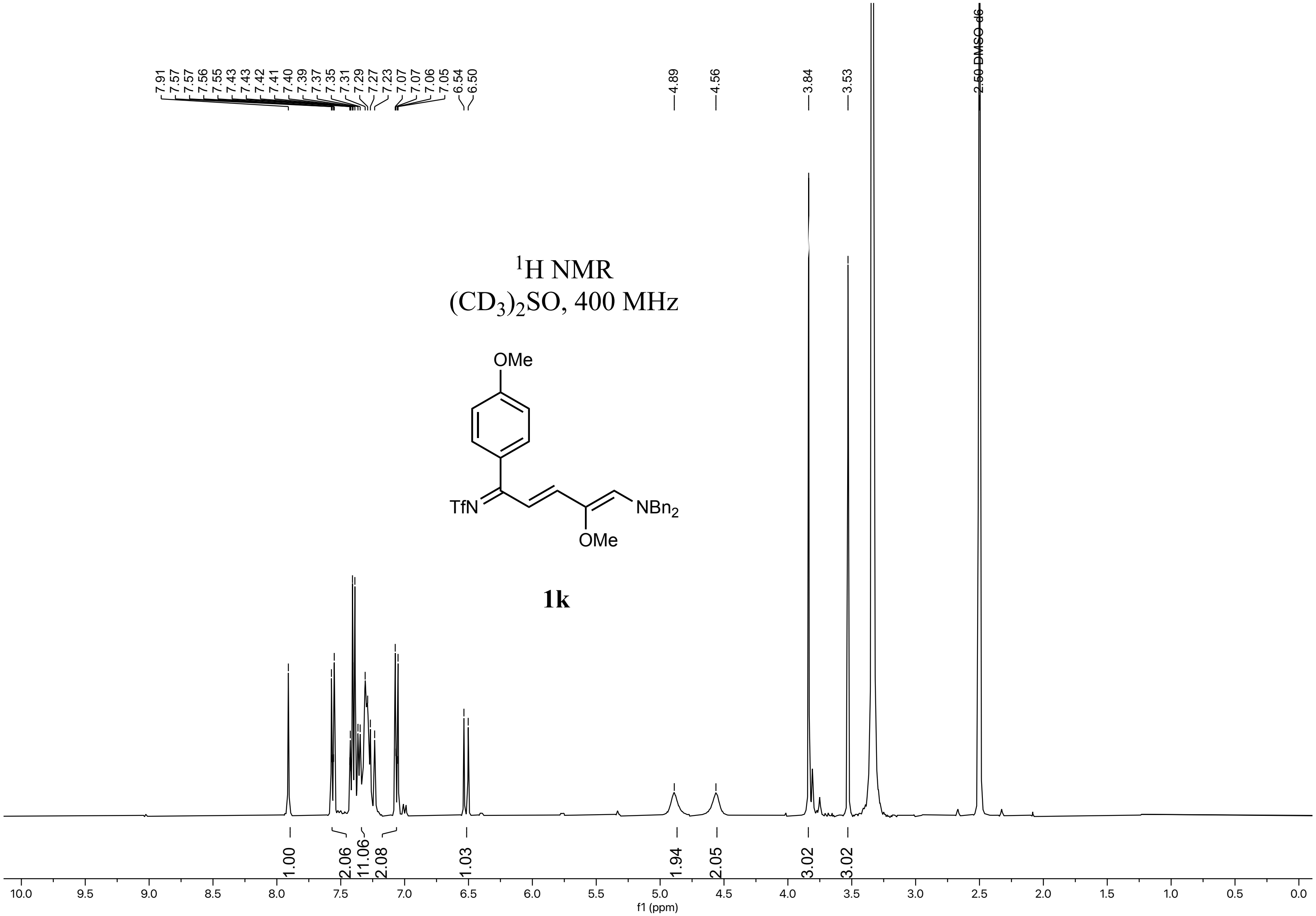

7.91
7.57
7.57
7.56
7.55
7.43
7.43
7.42
7.41
7.40
7.39
7.37
7.35
7.31
7.29
7.27
7.23
7.07
7.07
7.06
7.05
6.54
6.50
4.89
4.56
3.84
3.53
2.50 DMSO d6
^{1}H NMR
$(CD_3)_2SO$, 400 MHz
OMe
TfN
NBn$_2$
OMe
1k
1.00
2.06
11.06
2.08
1.03
1.94
2.05
3.02
3.02
10.0
9.5
9.0
8.5
8.0
7.5
7.0
6.5
6.0
5.5
5.0
4.5
4.0
3.5
3.0
2.5
2.0
1.5
1.0
0.5
0.0
f1 (ppm)

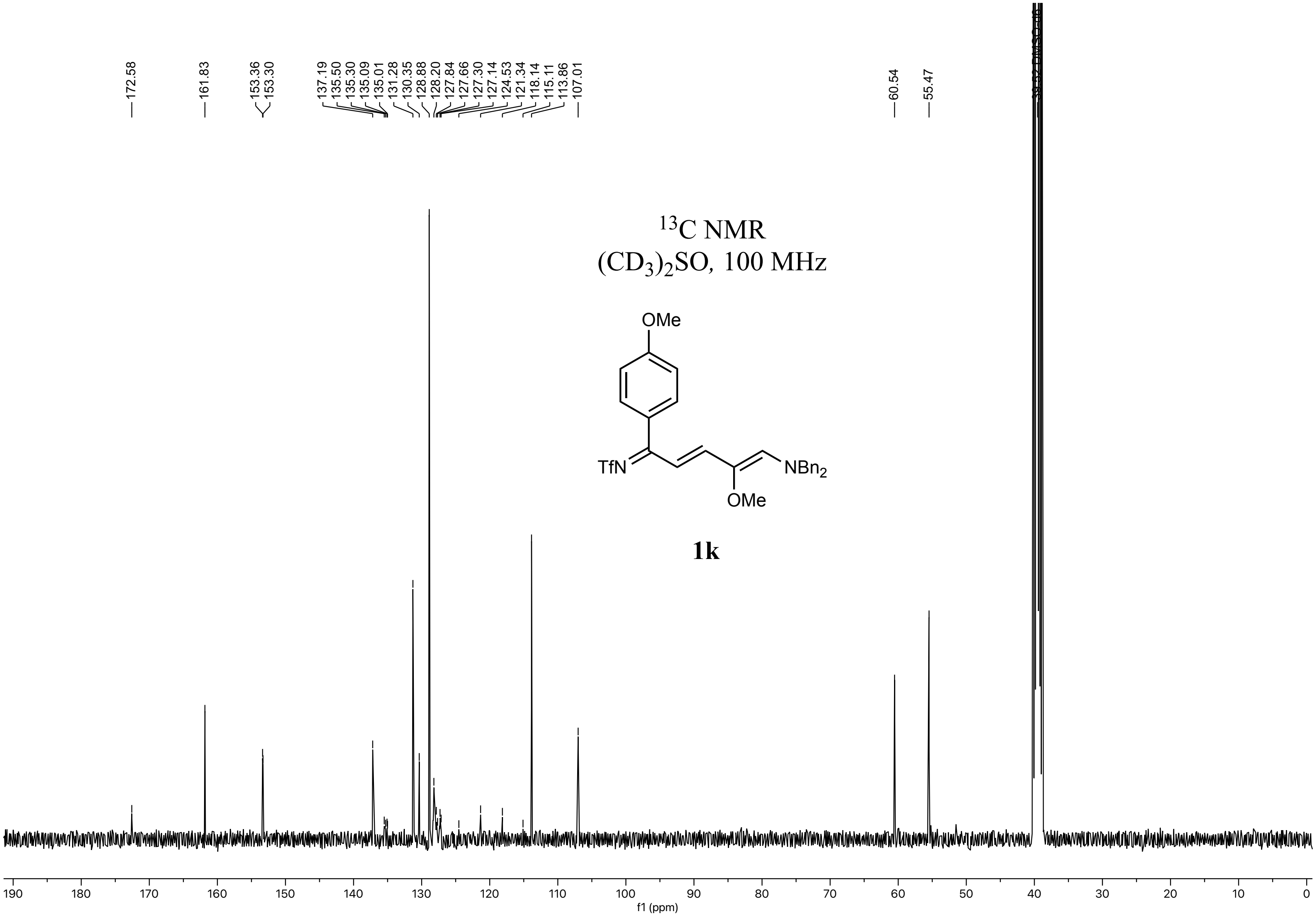
172.58
161.83
153.36
153.30
137.19
135.50
135.30
135.09
135.01
131.28
130.35
128.88
128.20
127.84
127.66
127.30
127.14
124.53
121.34
118.14
115.11
113.86
107.01
60.54
55.47
^{13}C NMR
$(CD_3)_2SO$, 100 MHz
OMe
TfN
NBn$_2$
OMe
1k
190
180
170
160
150
140
130
120
110
100
90
80
70
60
50
40
30
20
10
0
f1 (ppm)

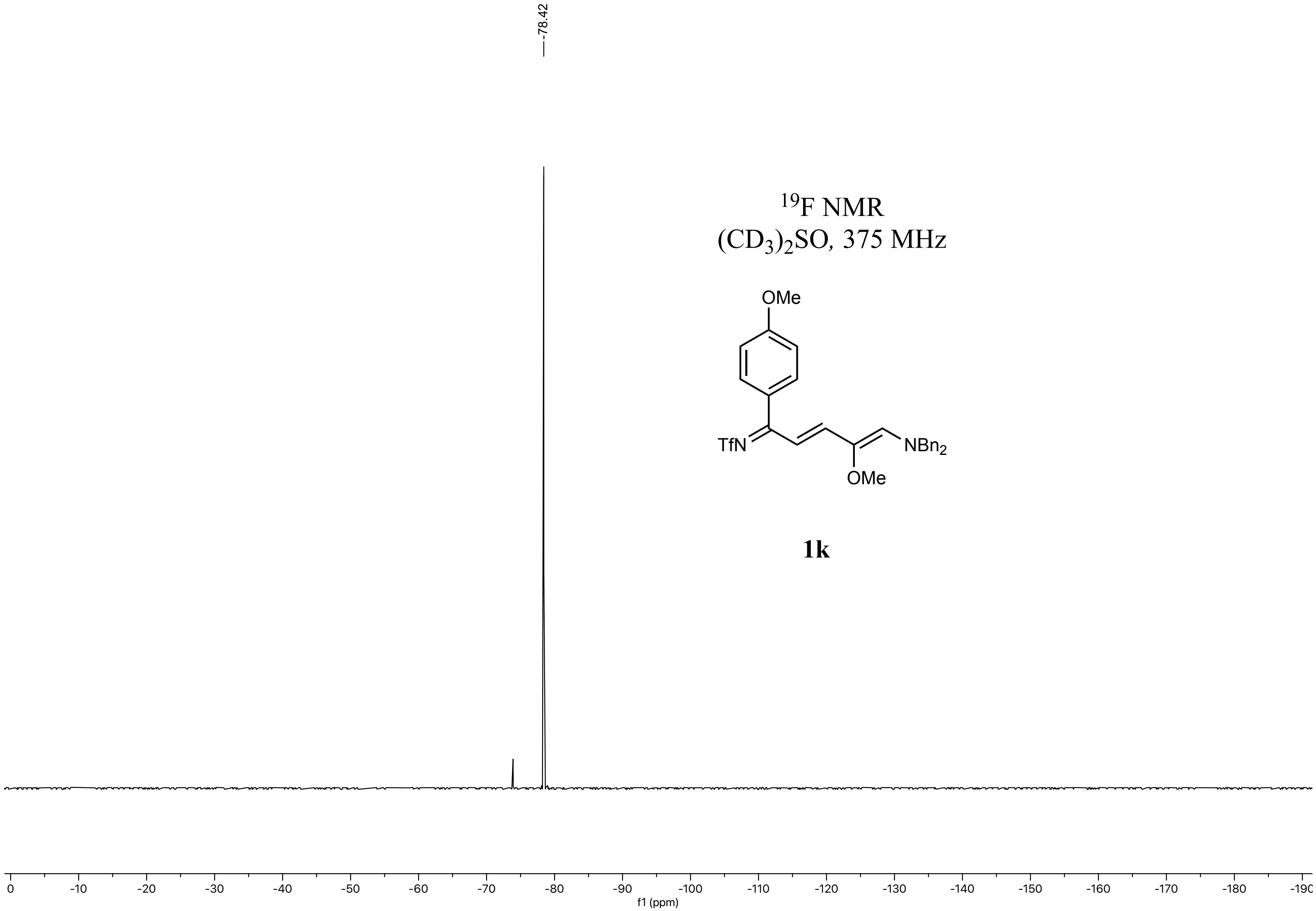

^{19}F NMR
$(CD_3)_2SO$, 375 MHz

1k

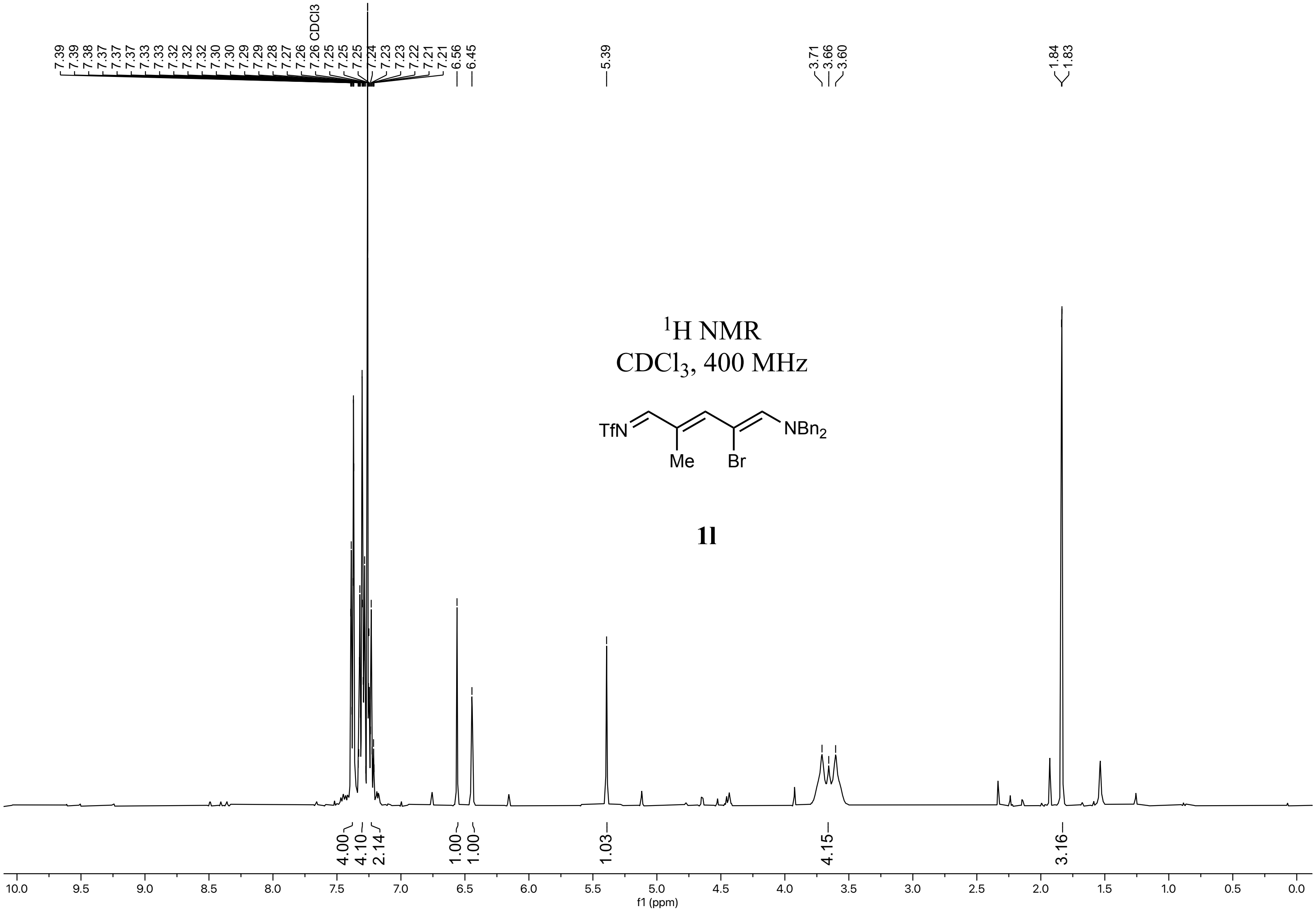

7.39 7.39 7.38 7.37 7.37 7.37 7.33 7.33 7.32 7.32 7.32 7.30 7.30 7.29 7.29 7.28 7.27 7.26 7.26 CDCl3 7.25 7.25 7.25 7.24 7.23 7.23 7.22 7.21 7.21 6.56 6.45
5.39
3.71 3.66 3.60
1.84 1.83
^{1}H NMR
$CDCl_3$, 400 MHz
TfN
Me
Br
NBn_2
1l
4.00 4.10 2.14 1.00 1.00 1.03 4.15 3.16
10.0 9.5 9.0 8.5 8.0 7.5 7.0 6.5 6.0 5.5 5.0 4.5 4.0 3.5 3.0 2.5 2.0 1.5 1.0 0.5 0.0
f1 (ppm)

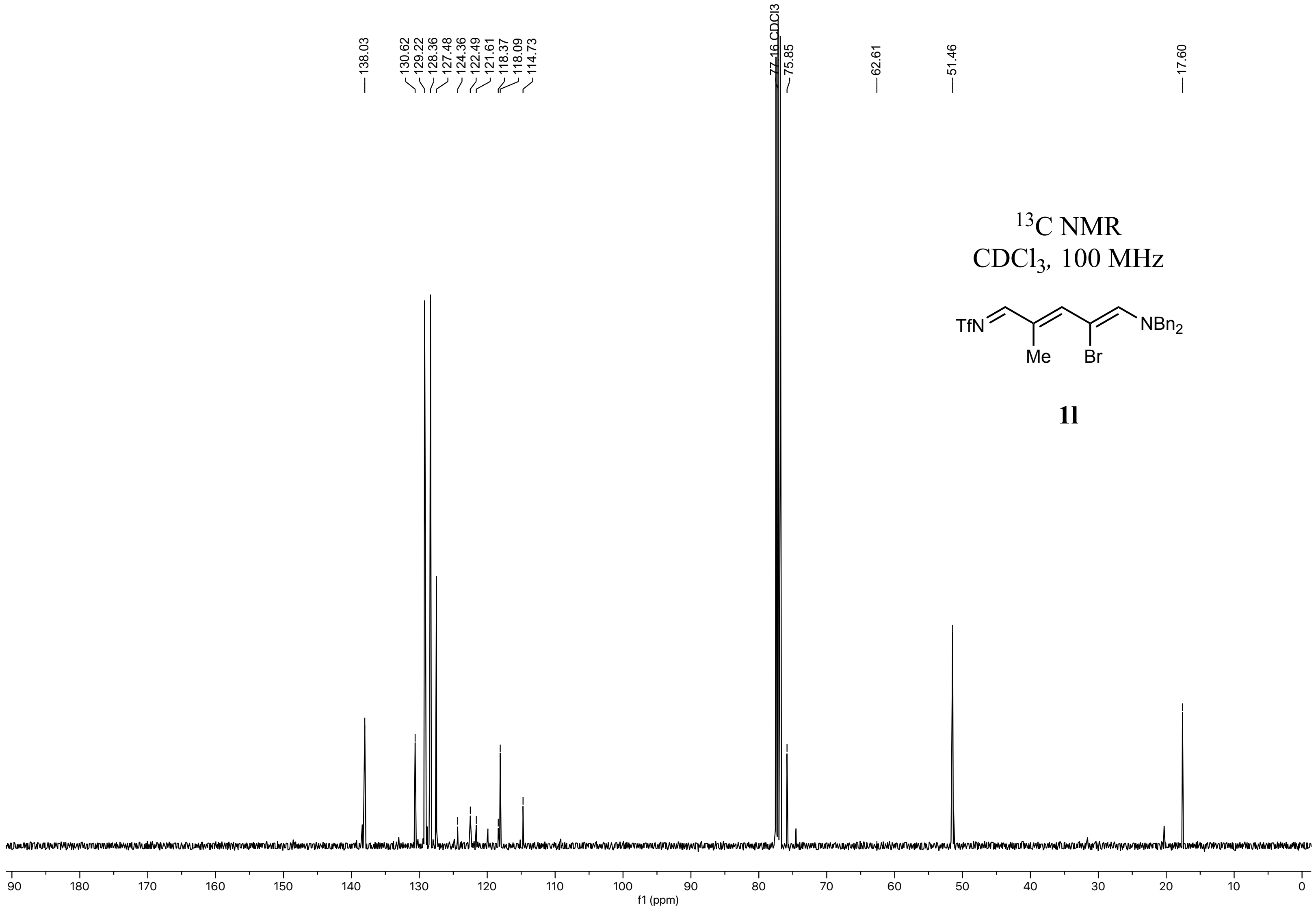
138.03
130.62
129.22
128.36
127.48
124.36
122.49
121.61
118.37
118.09
114.73
77.16 CDCl3
75.85
62.61
51.46
17.60
13C NMR
CDCl3, 100 MHz
TfN
NBn2
Me
Br
1l
f1 (ppm)

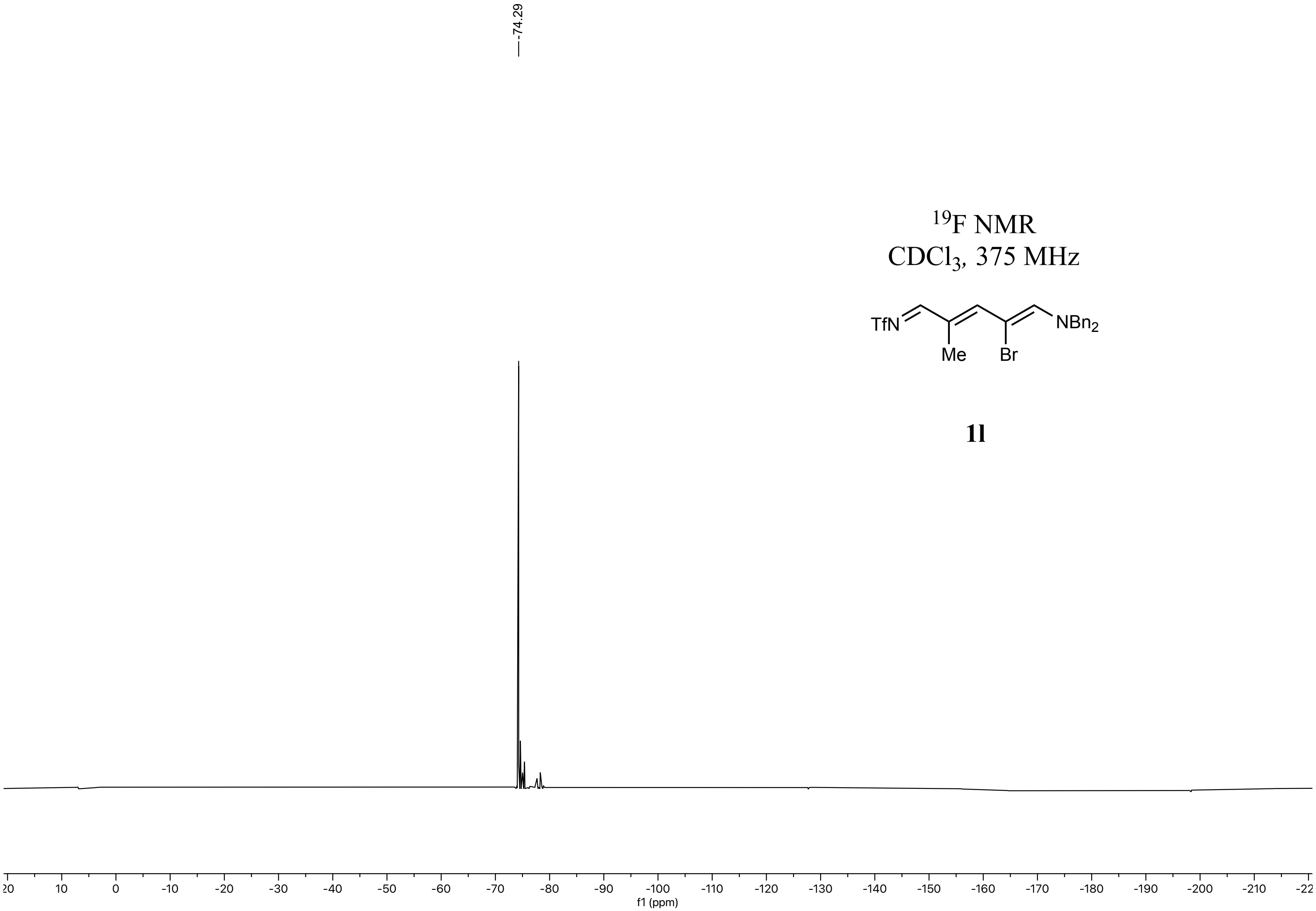

^{19}F NMR

$CDCl_3$, 375 MHz

1l

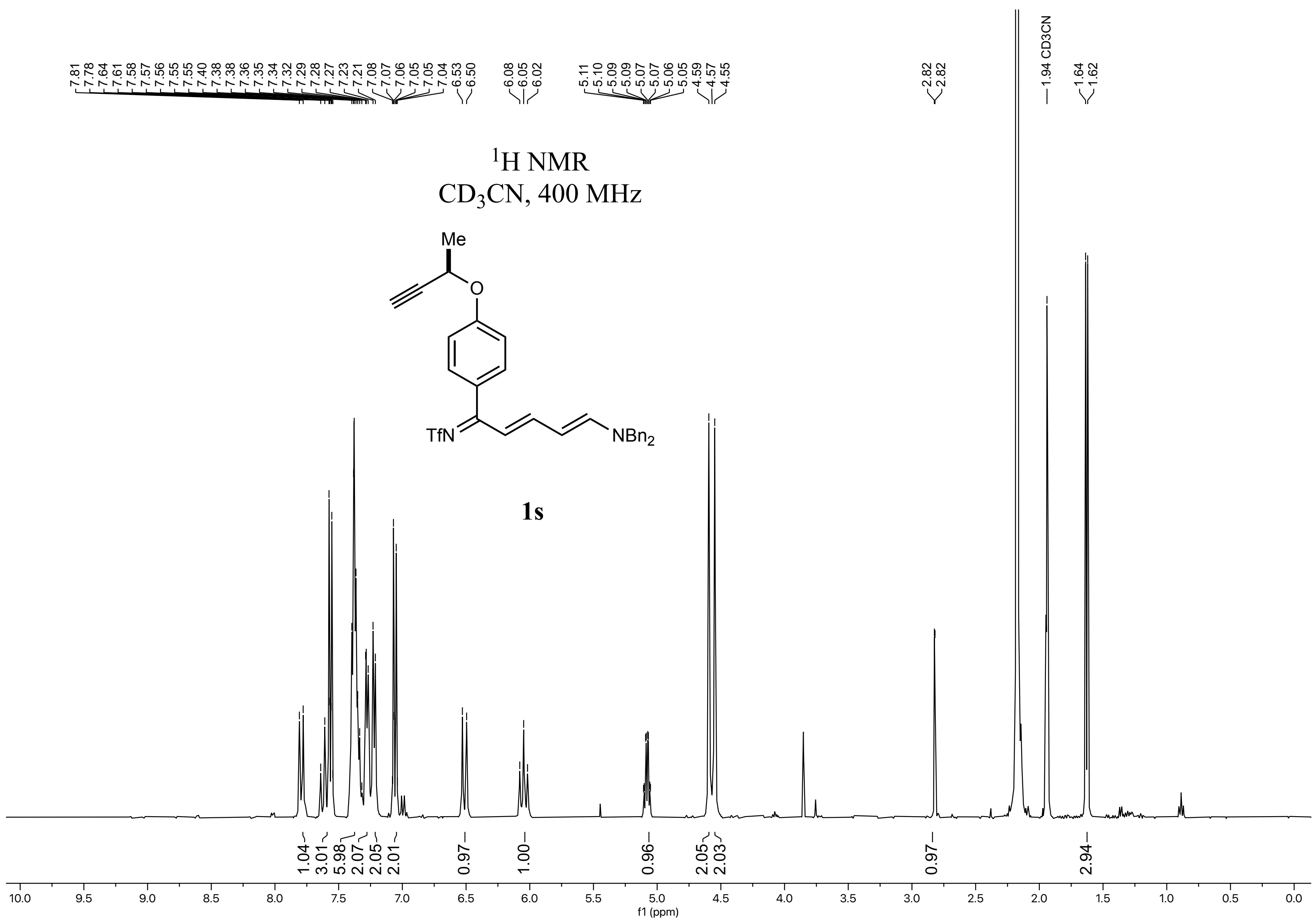

^{1}H NMR
CD_3CN, 400 MHz
Me
O
TfN
NBn_2
1s
1.94 CD3CN
f1 (ppm)

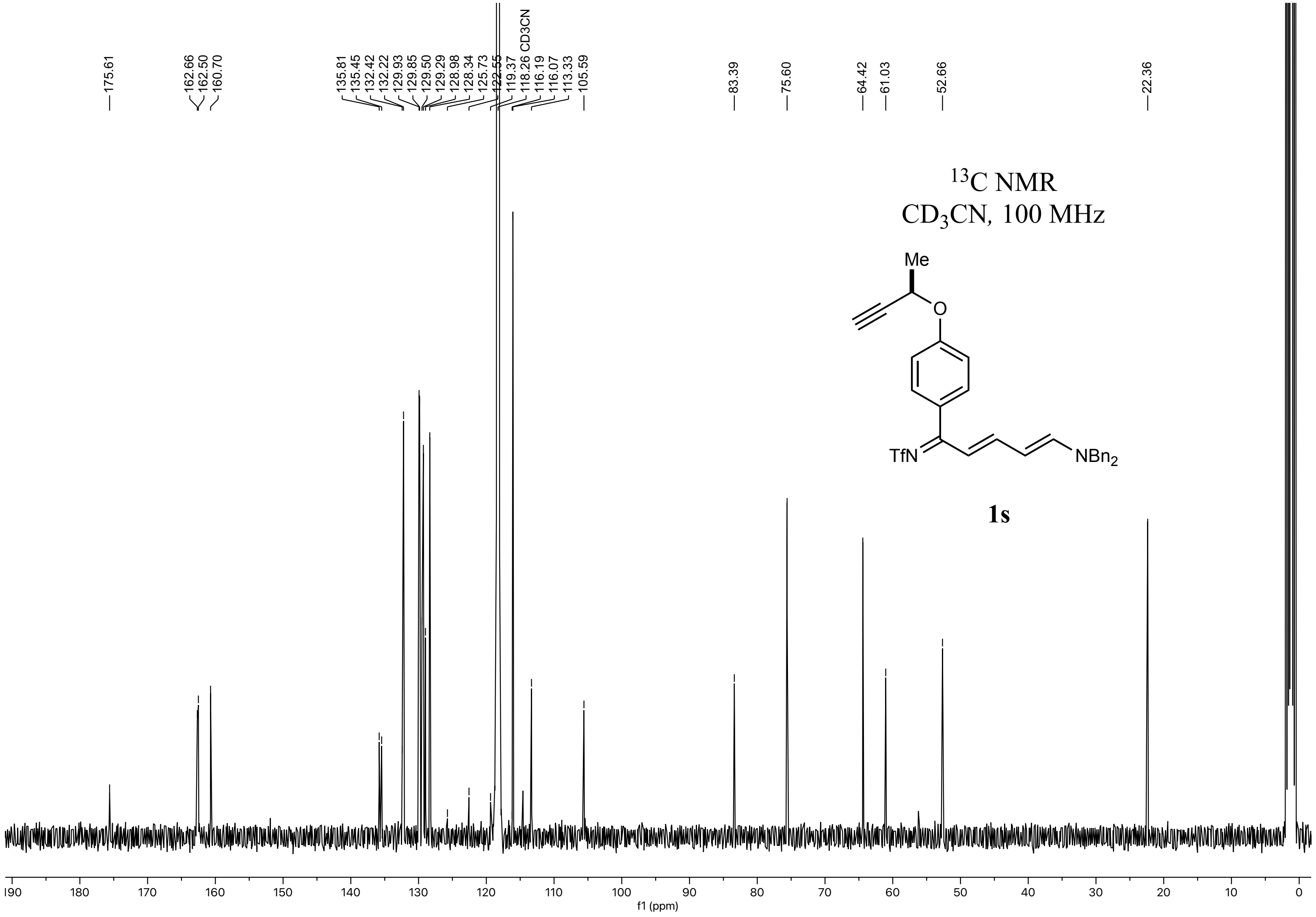
175.61
162.66
162.50
160.70
135.81
135.45
132.42
132.22
129.93
129.85
129.50
129.29
128.98
128.34
125.73
122.55
119.37
118.26 CD3CN
116.19
116.07
113.33
105.59
83.39
75.60
64.42
61.03
52.66
22.36
^{13}C NMR
CD_3CN, 100 MHz
Me
O
TfN
NBn_2
1s
f1 (ppm)

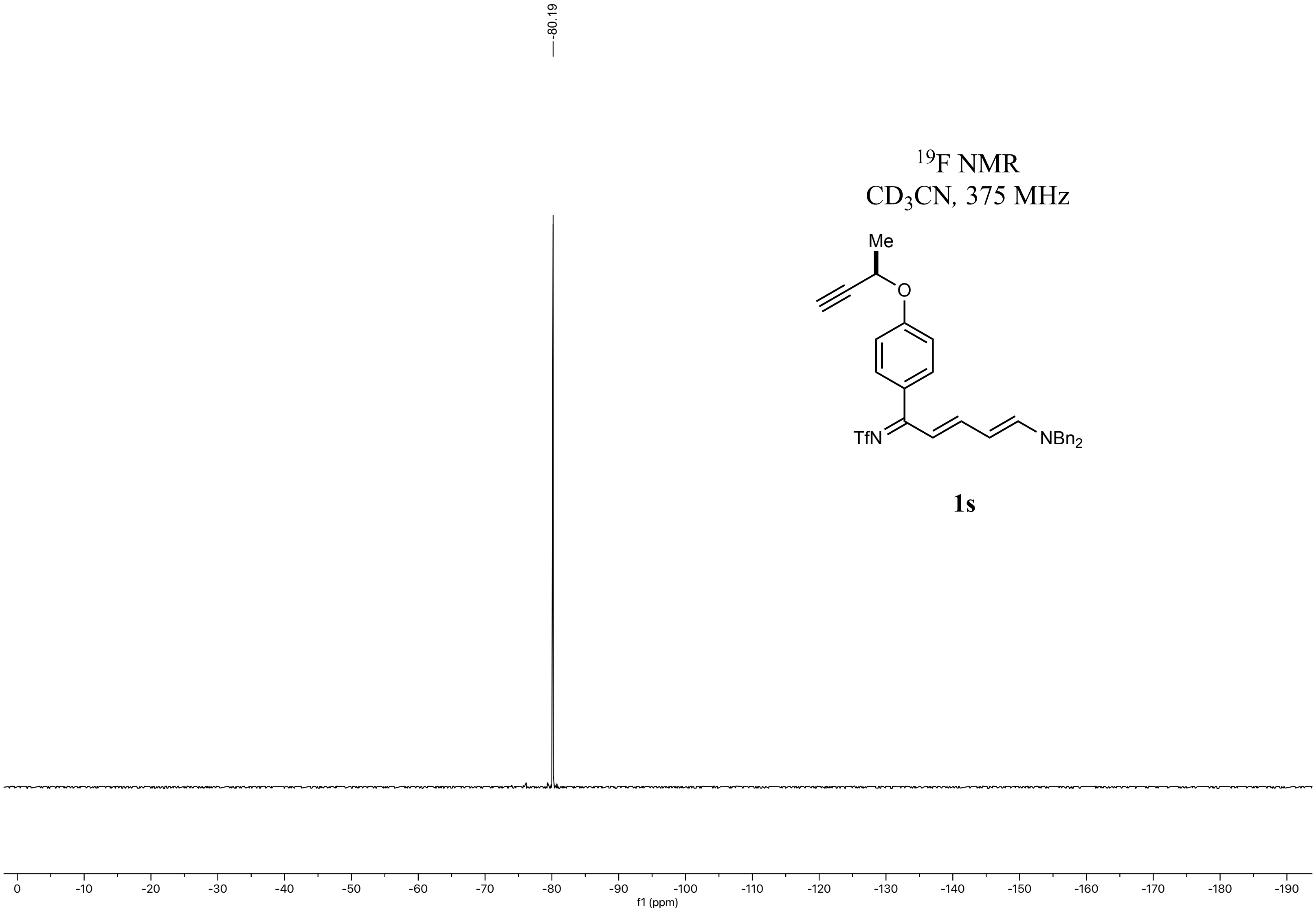
-80.19
^{19}F NMR
CD_3CN, 375 MHz
Me
O
TfN
NBn$_2$
1s
0 -10 -20 -30 -40 -50 -60 -70 -80 -90 -100 -110 -120 -130 -140 -150 -160 -170 -180 -190
f1 (ppm)

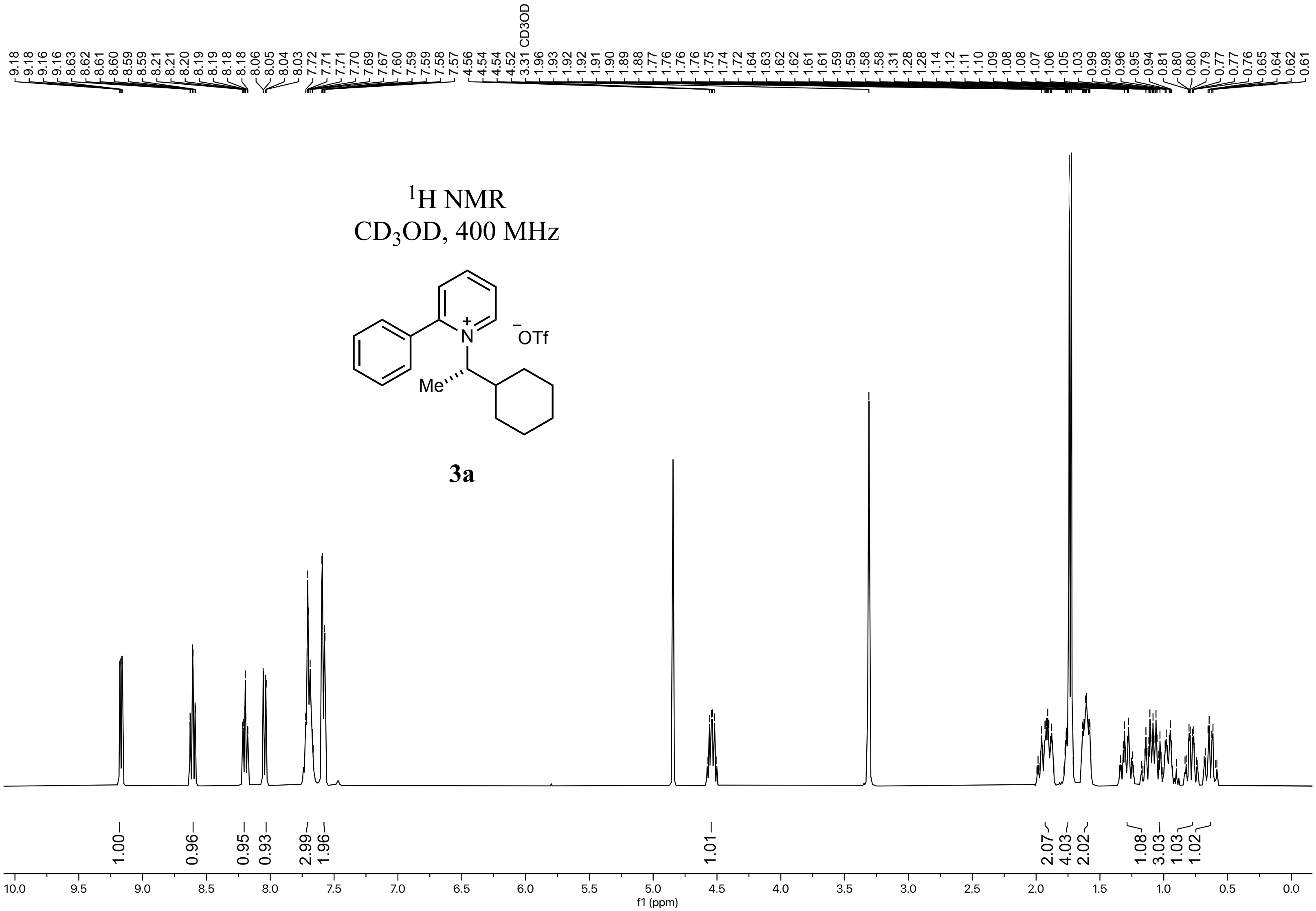

^{1}H NMR
CD$_3$OD, 400 MHz
OTf
N
Me
3a
CD3OD
9.18 9.18 9.16 8.63 8.62 8.61 8.60 8.59 8.59 8.21 8.20 8.19 8.19 8.18 8.18 8.06 8.05 8.04 8.03 7.72 7.71 7.71 7.70 7.69 7.67 7.60 7.59 7.59 7.58 7.57 4.56 4.54 4.54 4.52 3.31 1.96 1.93 1.92 1.92 1.91 1.90 1.89 1.88 1.77 1.76 1.76 1.75 1.74 1.72 1.64 1.63 1.62 1.62 1.61 1.61 1.59 1.59 1.58 1.58 1.31 1.28 1.28 1.14 1.12 1.11 1.10 1.09 1.08 1.08 1.07 1.06 1.05 1.03 0.99 0.98 0.96 0.95 0.94 0.81 0.80 0.80 0.79 0.77 0.76 0.65 0.64 0.62 0.61
1.00 0.96 0.95 0.93 2.99 1.96 1.01 2.07 4.03 2.02 1.08 3.03 1.03 1.02
10.0 9.5 9.0 8.5 8.0 7.5 7.0 6.5 6.0 5.5 5.0 4.5 4.0 3.5 3.0 2.5 2.0 1.5 1.0 0.5 0.0
f1 (ppm)

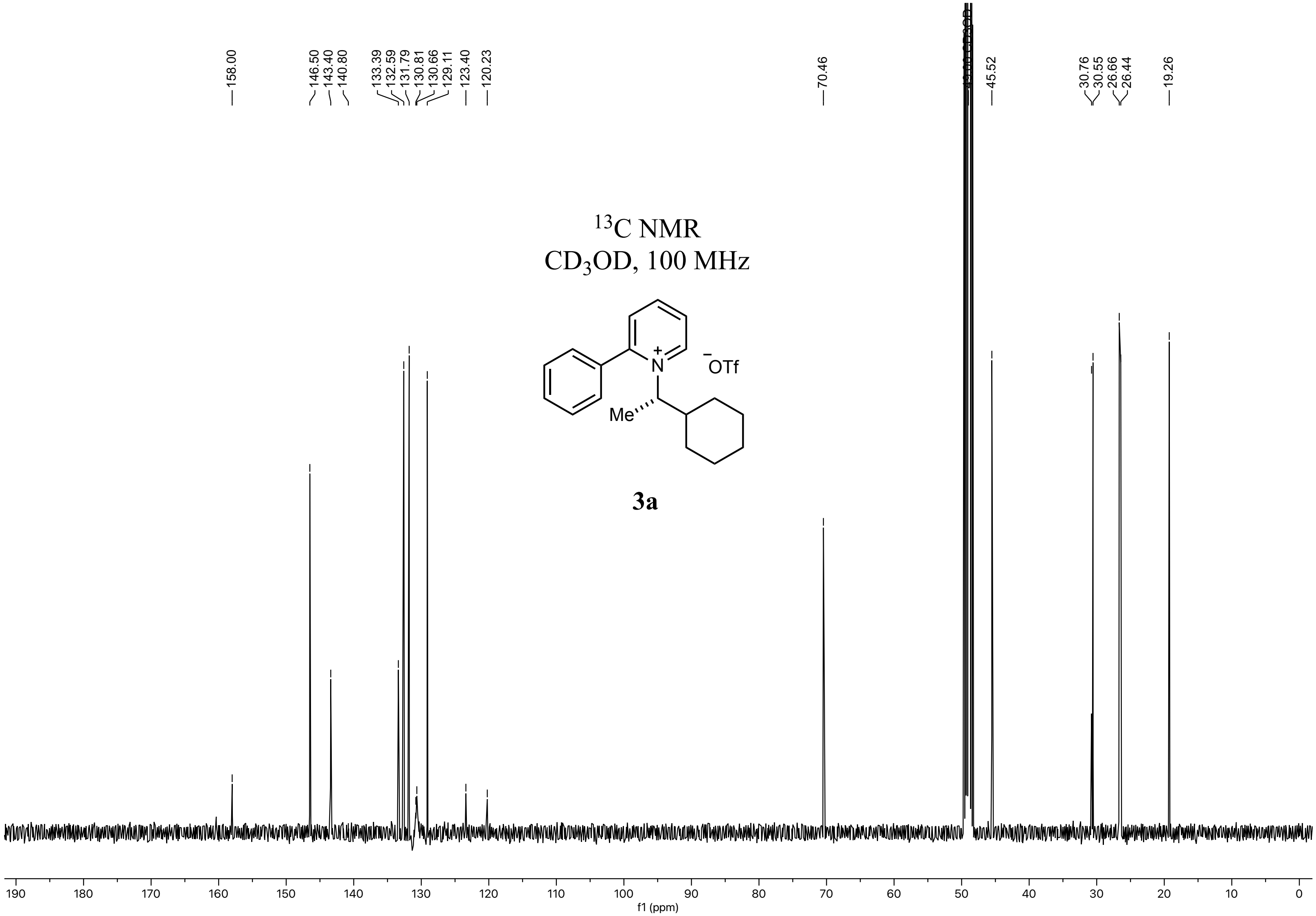
158.00
146.50
143.40
140.80
133.39
132.59
131.79
130.81
130.66
129.11
123.40
120.23
70.46
45.52
30.76
30.55
26.66
26.44
19.26
13C NMR
CD3OD, 100 MHz
OTf
Me
3a
190
180
170
160
150
140
130
120
110
100
90
80
70
60
50
40
30
20
10
0
f1 (ppm)

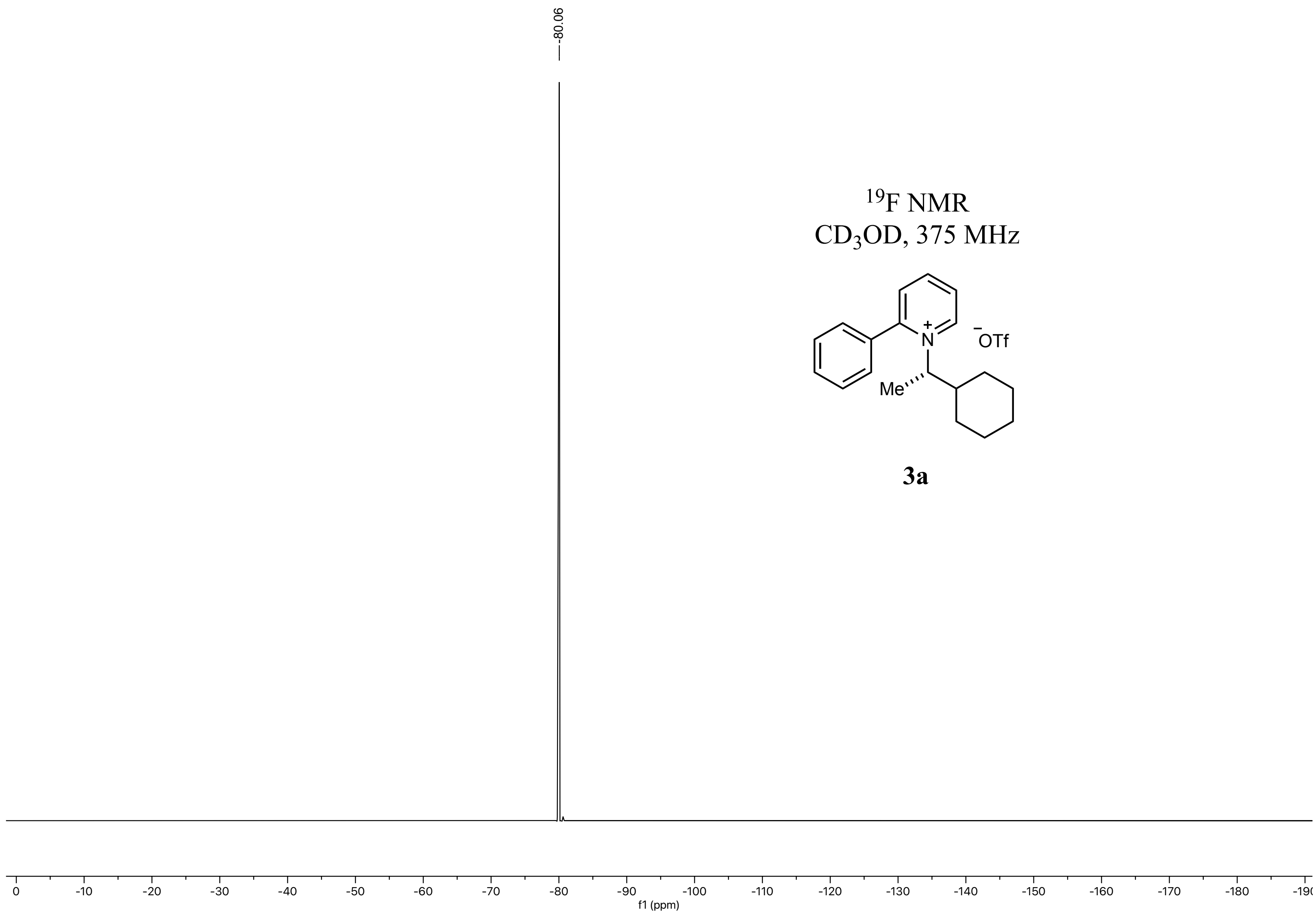
-80.06
^{19}F NMR
CD$_3$OD, 375 MHz
OTf
N
Me
3a
0
-10
-20
-30
-40
-50
-60
-70
-80
-90
-100
-110
-120
-130
-140
-150
-160
-170
-180
f1 (ppm)

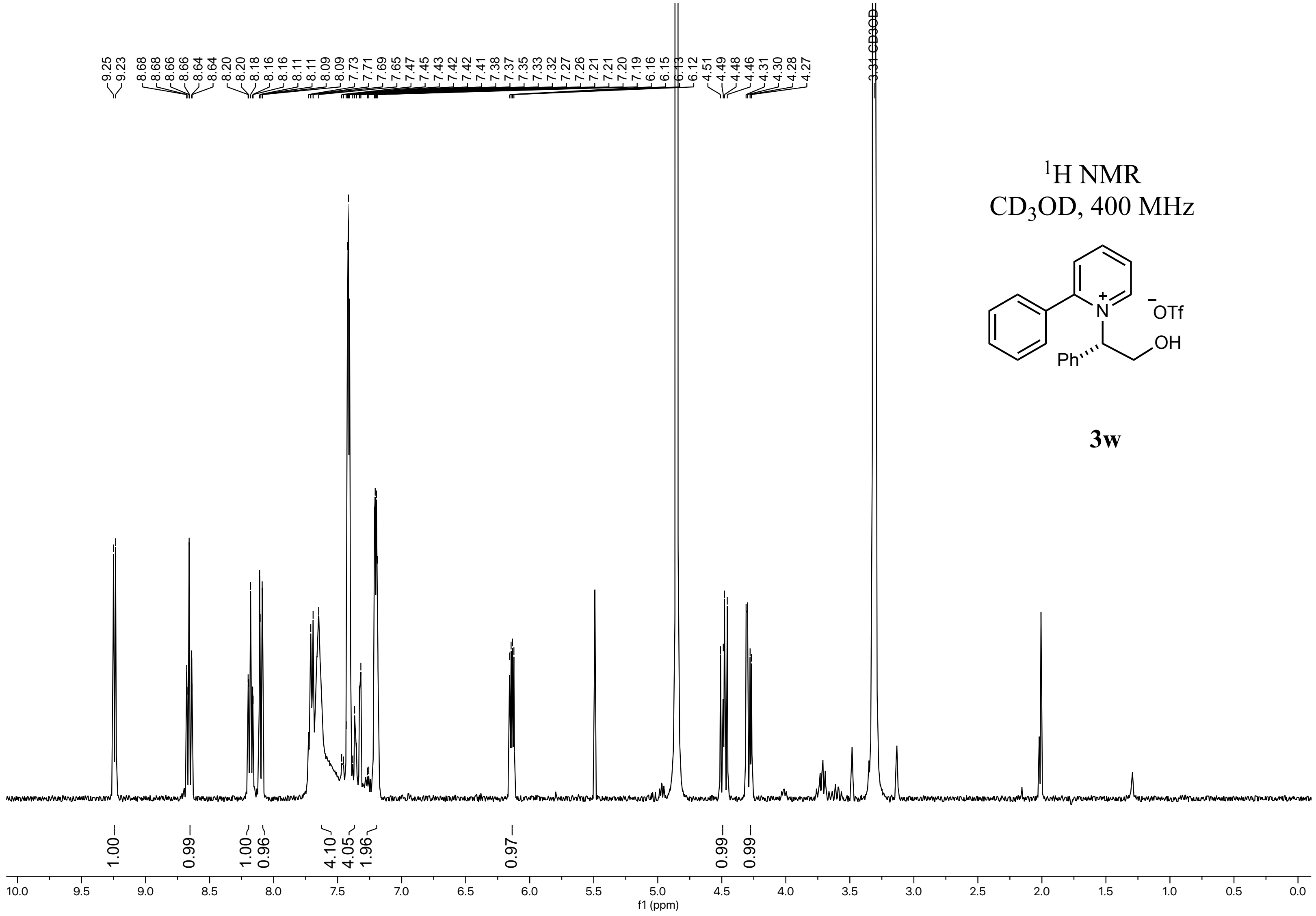

1H NMR
CD3OD, 400 MHz
OTf
OH
Ph
3w
f1 (ppm)

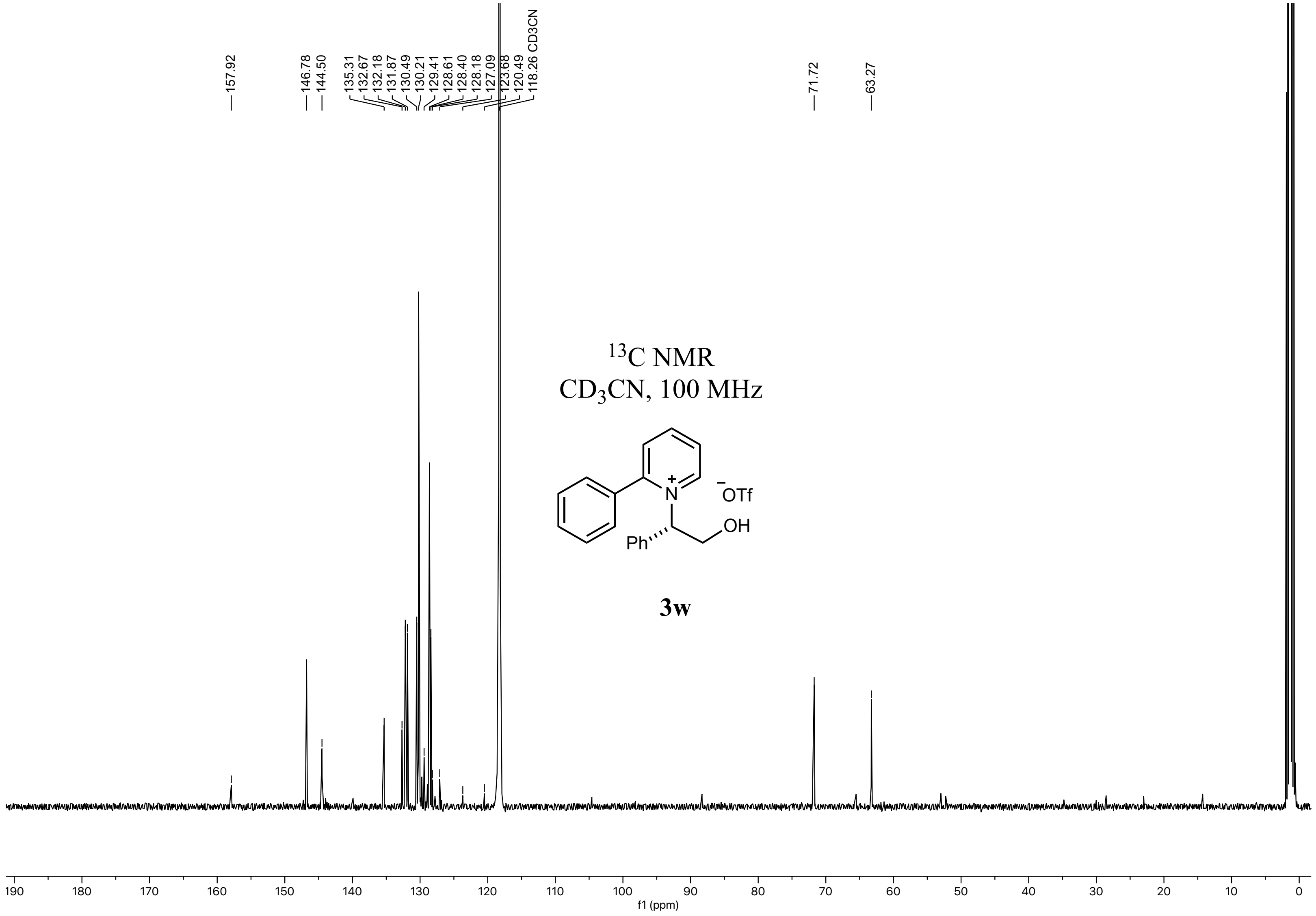

157.92
146.78
144.50
135.31
132.67
132.18
131.87
130.49
130.21
129.41
128.61
128.40
128.18
127.09
123.68
120.49
118.26 CD3CN
71.72
63.27
13C NMR
CD3CN, 100 MHz
OTf
OH
Ph
3w
f1 (ppm)

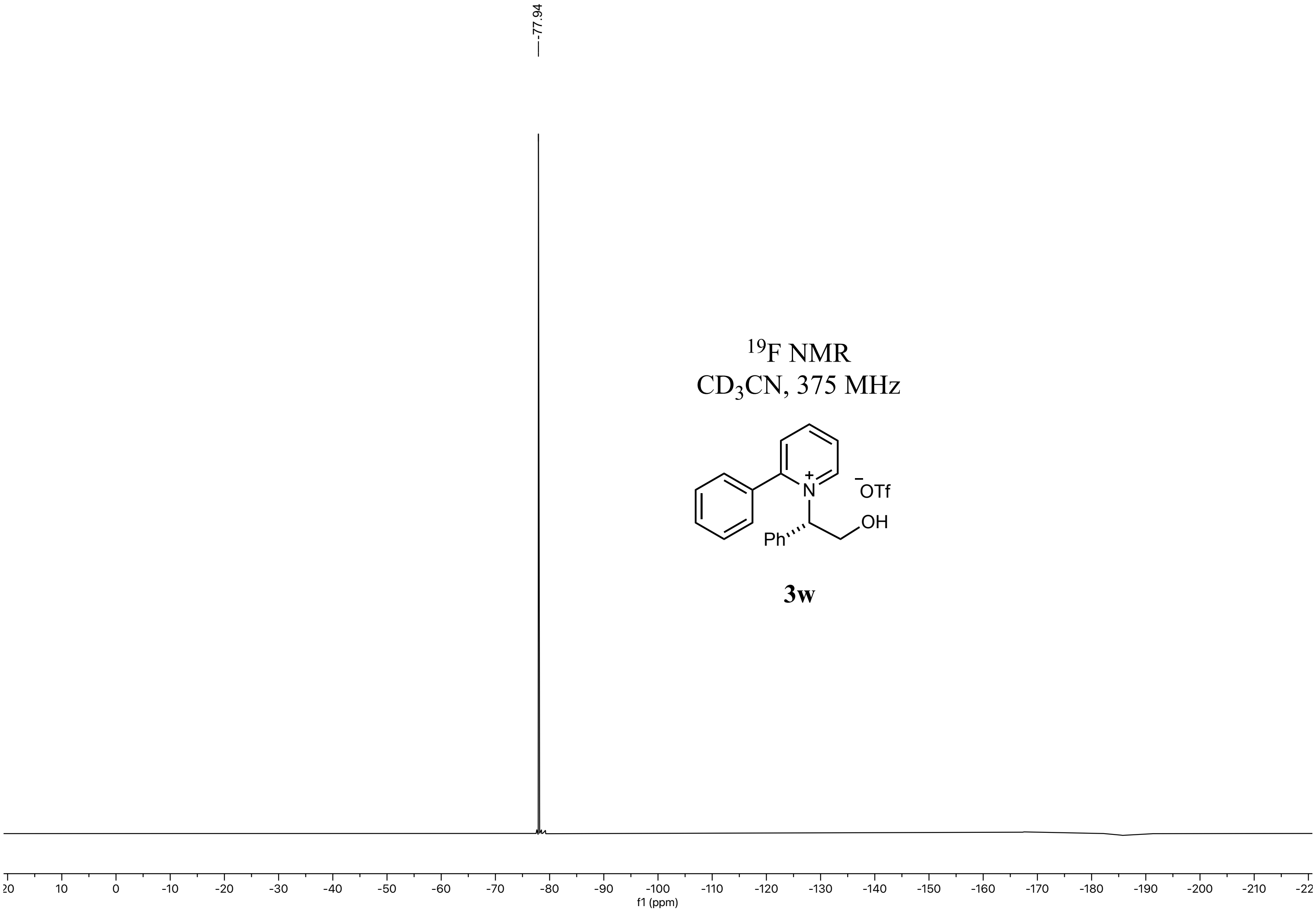

^{19}F NMR
CD_3CN, 375 MHz

3w

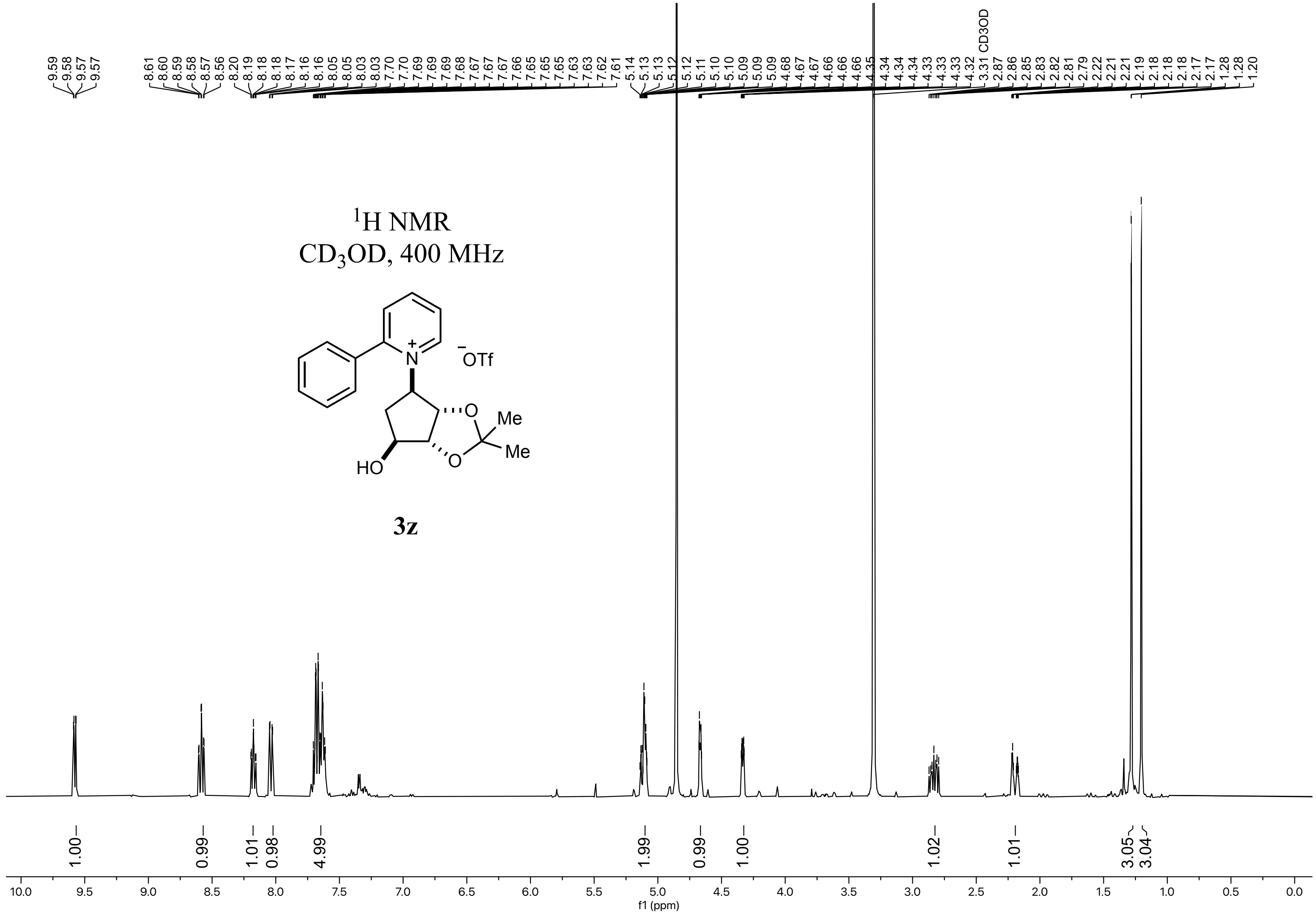

9.59 9.58 9.57 9.57 8.61 8.60 8.59 8.58 8.57 8.56 8.20 8.19 8.18 8.18 8.17 8.16 8.16 8.05 8.05 8.03 8.03 7.70 7.70 7.69 7.69 7.68 7.67 7.67 7.66 7.65 7.65 7.63 7.62 7.61 5.14 5.13 5.13 5.12 5.12 5.11 5.10 5.10 5.09 5.09 4.68 4.67 4.67 4.66 4.66 4.66 4.35 4.34 4.34 4.34 4.33 4.33 4.33 4.32 3.31 CD3OD 2.87 2.86 2.85 2.83 2.82 2.81 2.79 2.22 2.21 2.21 2.19 2.18 2.18 2.17 2.17 1.28 1.28 1.20
1H NMR
CD3OD, 400 MHz
N
+
−OTf
O
O
Me
Me
HO
3z
1.00 0.99 1.01 0.98 4.99 1.99 0.99 1.00 1.02 1.01 3.05 3.04
10.0 9.5 9.0 8.5 8.0 7.5 7.0 6.5 6.0 5.5 5.0 4.5 4.0 3.5 3.0 2.5 2.0 1.5 1.0 0.5 0.0
f1 (ppm)

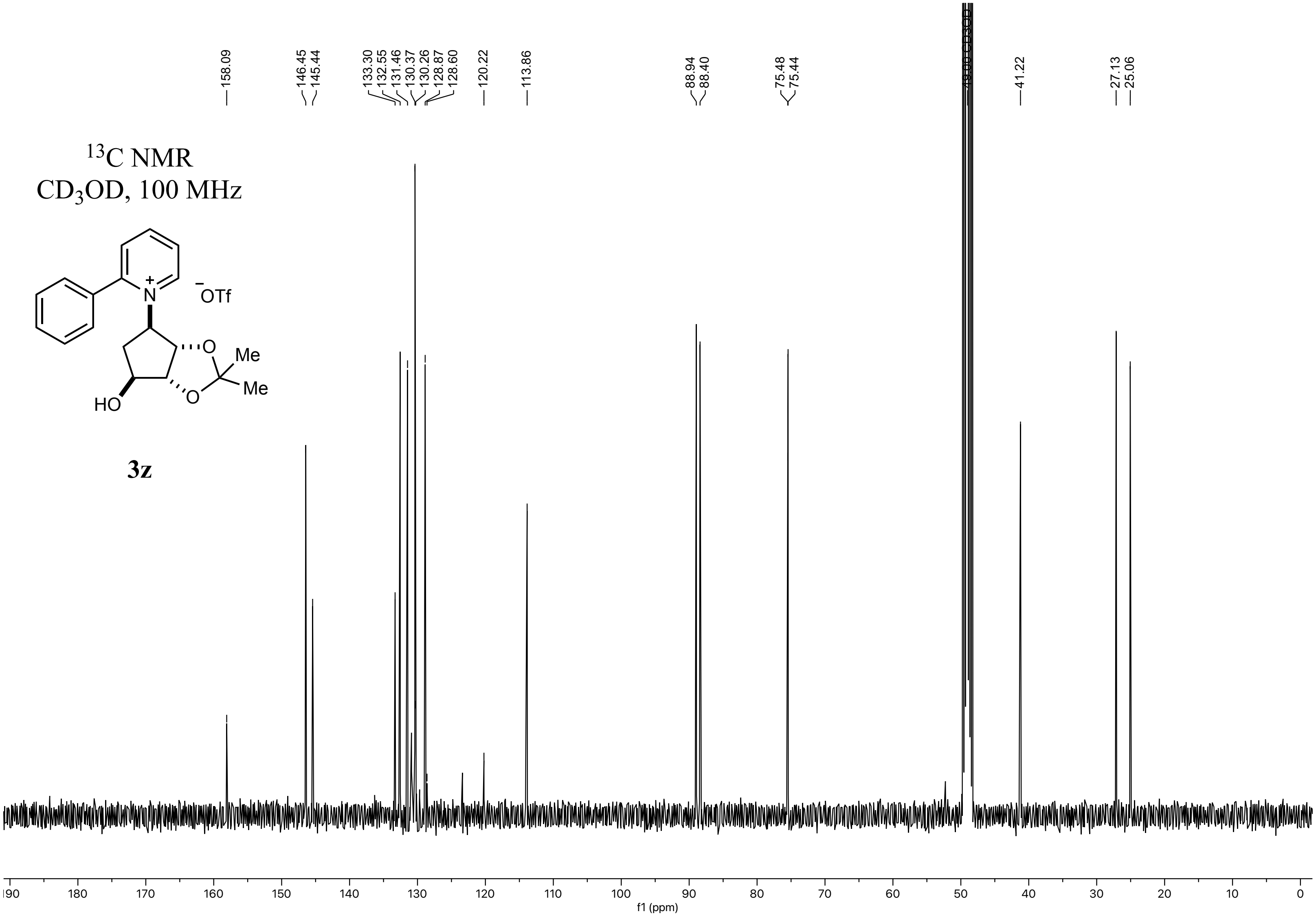

158.09
146.45
145.44
133.30
132.55
131.46
130.37
130.26
128.87
128.60
120.22
113.86
88.94
88.40
75.48
75.44
CD3OD
41.22
27.13
25.06
^{13}C NMR
CD_3OD, 100 MHz
OTf
N
Me
Me
O
O
HO
3z
190
180
170
160
150
140
130
120
110
100
90
80
70
60
50
40
30
20
10
0
f1 (ppm)

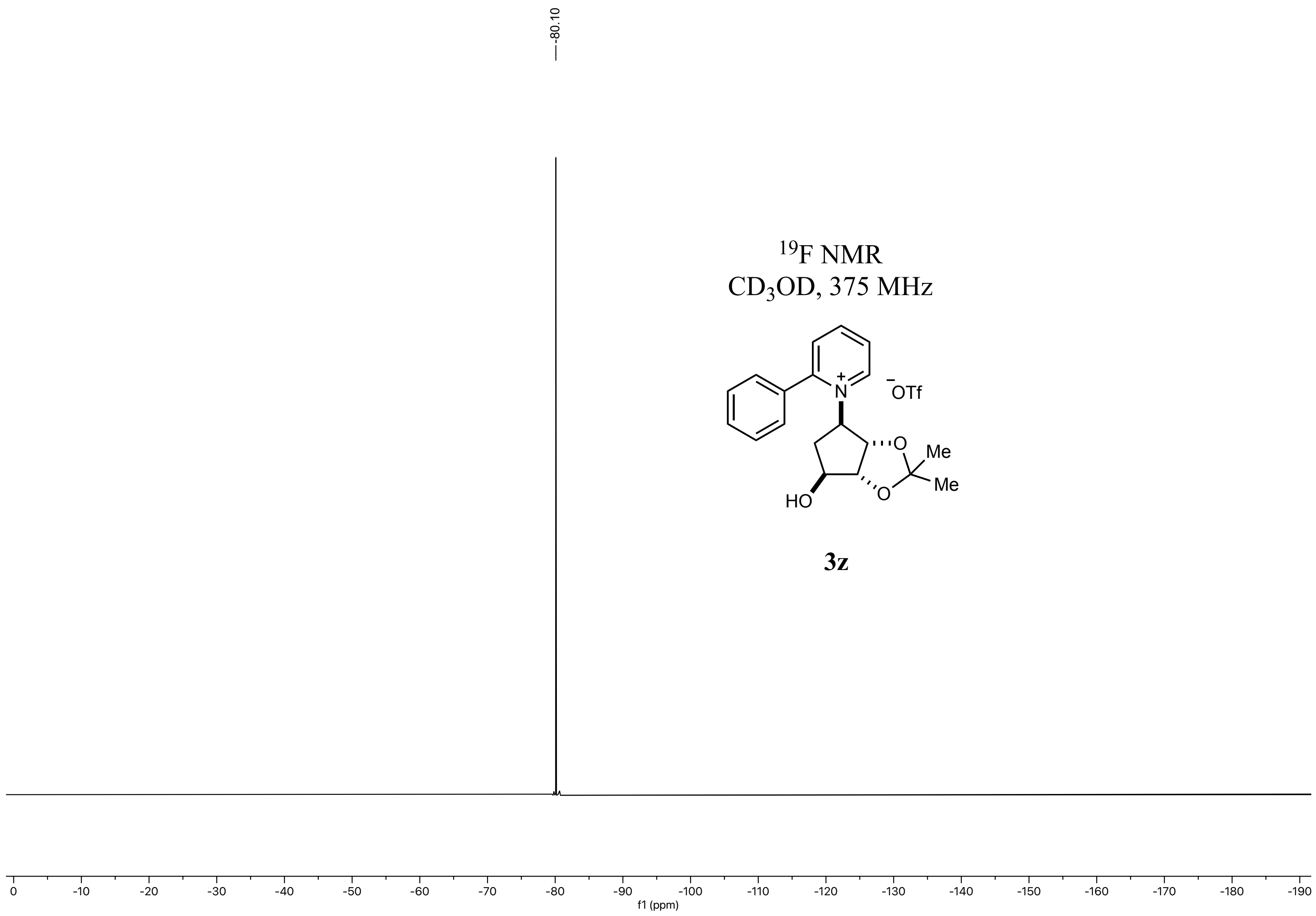

^{19}F NMR
CD_3OD, 375 MHz

3z

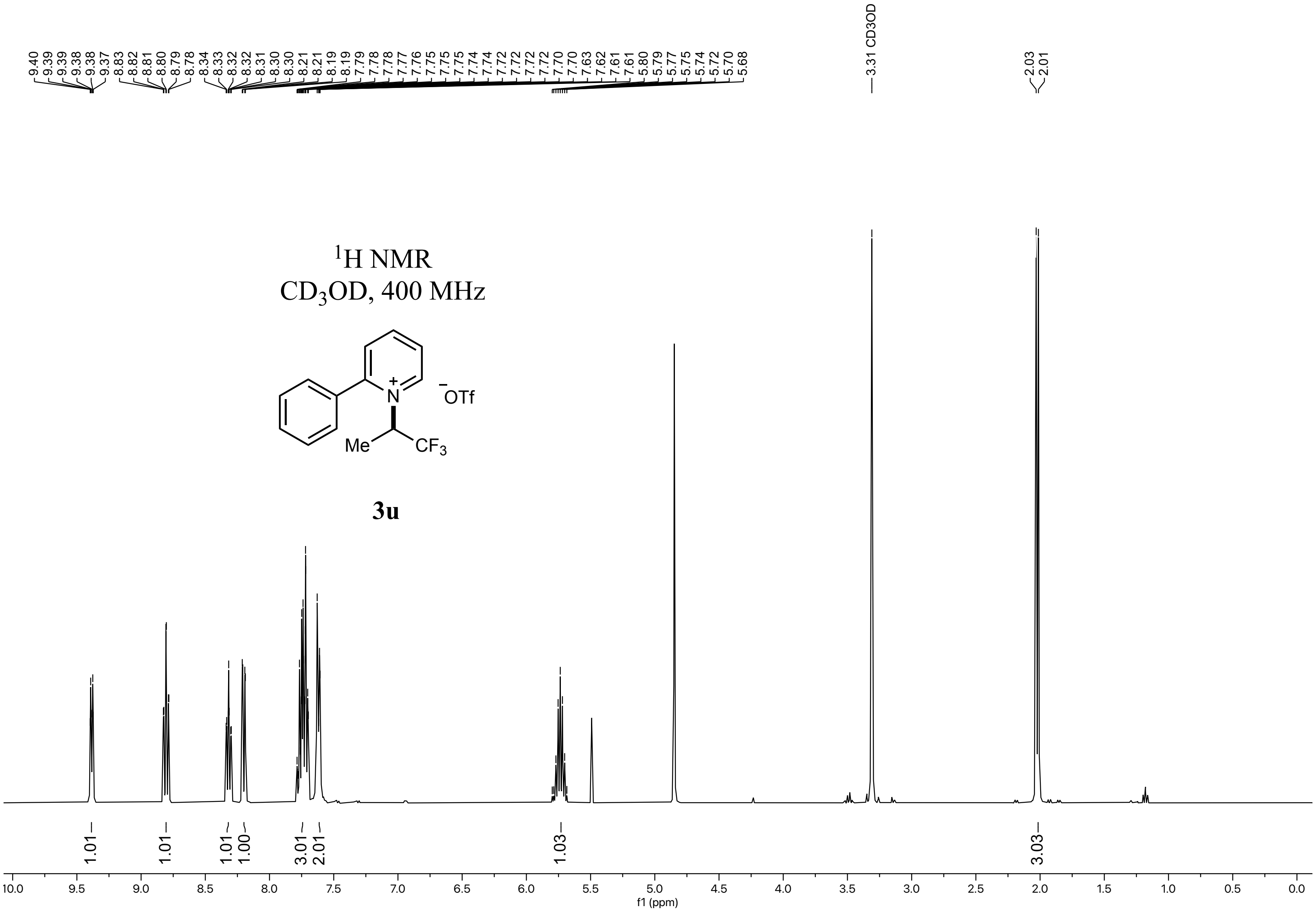

9.40 9.39 9.39 9.38 9.38 9.37 8.83 8.82 8.81 8.80 8.79 8.78 8.34 8.33 8.32 8.32 8.31 8.30 8.30 8.21 8.21 8.19 8.19 7.79 7.78 7.78 7.77 7.76 7.75 7.75 7.75 7.74 7.74 7.72 7.72 7.72 7.72 7.70 7.70 7.63 7.62 7.61 7.61 5.80 5.79 5.77 5.75 5.74 5.72 5.70 5.68
3.31 CD3OD
2.03 2.01
^{1}H NMR
CD_3OD, 400 MHz
OTf
N
Me
CF_3
3u
1.01 1.01 1.01 1.00 3.01 2.01 1.03 3.03
10.0 9.5 9.0 8.5 8.0 7.5 7.0 6.5 6.0 5.5 5.0 4.5 4.0 3.5 3.0 2.5 2.0 1.5 1.0 0.5 0.0
f1 (ppm)

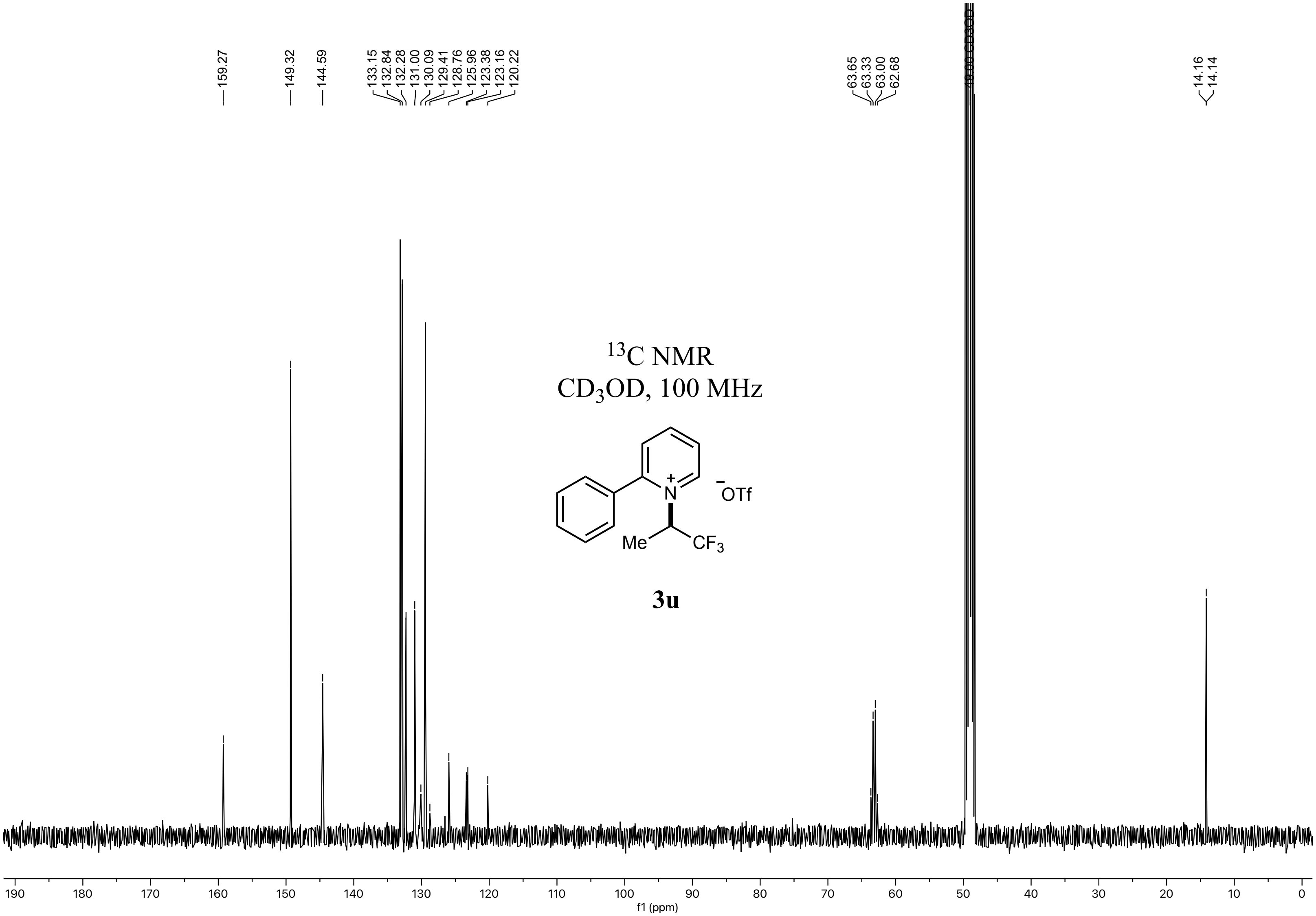
159.27
149.32
144.59
133.15
132.84
132.28
131.00
130.09
129.41
128.76
125.96
123.38
123.16
120.22
63.65
63.33
63.00
62.68
14.16
14.14
^{13}C NMR
CD_3OD, 100 MHz
OTf
N
Me
CF_3
f1 (ppm)
190 180 170 160 150 140 130 120 110 100 90 80 70 60 50 40 30 20 10 0

3u

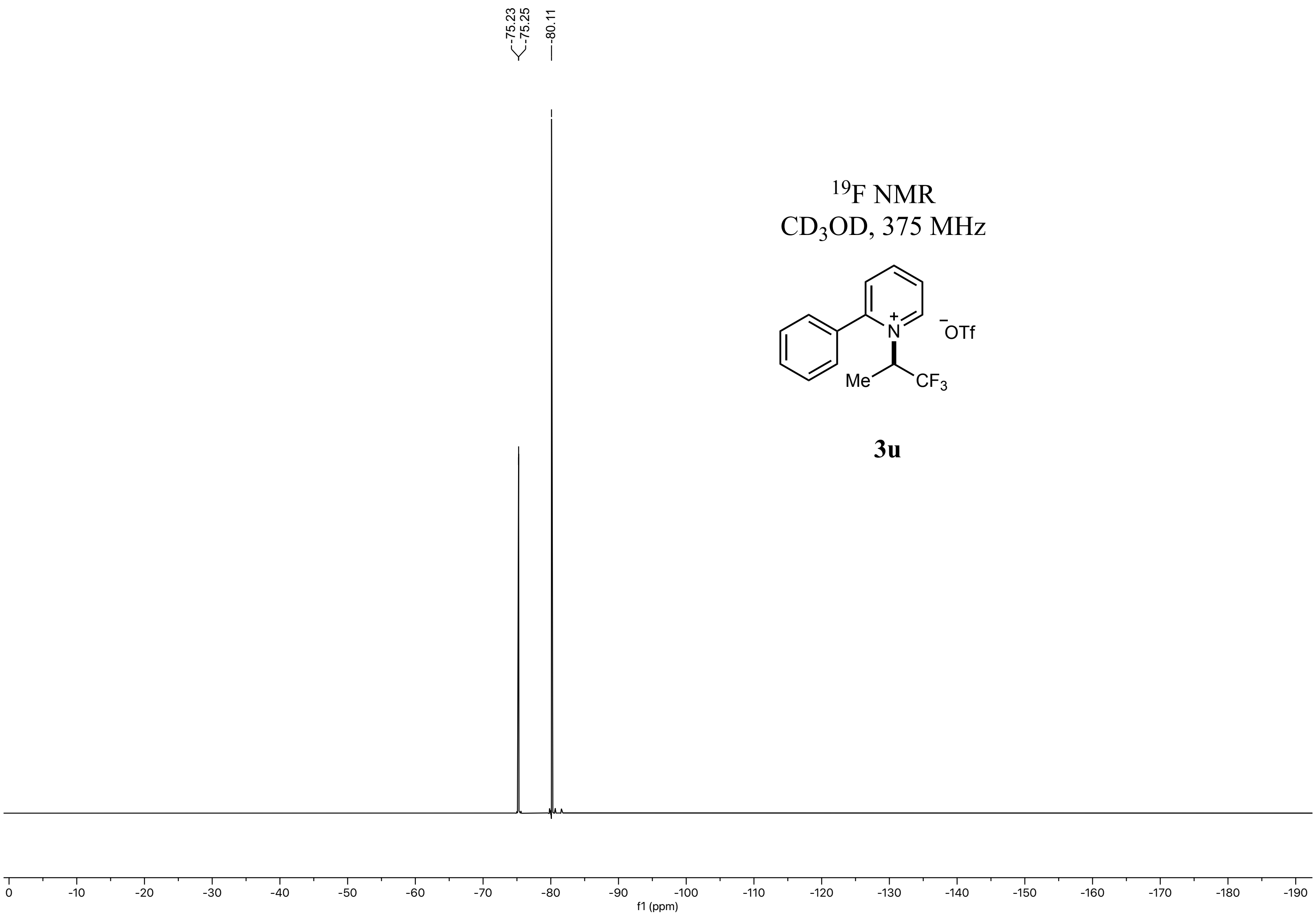
-75.23
-75.25
-80.11
^{19}F NMR
CD_3OD, 375 MHz
OTf
N
Me
CF_3
0
-10
-20
-30
-40
-50
-60
-70
-80
-90
-100
-110
-120
-130
-140
-150
-160
-170
-180
-190
f1 (ppm)

3u

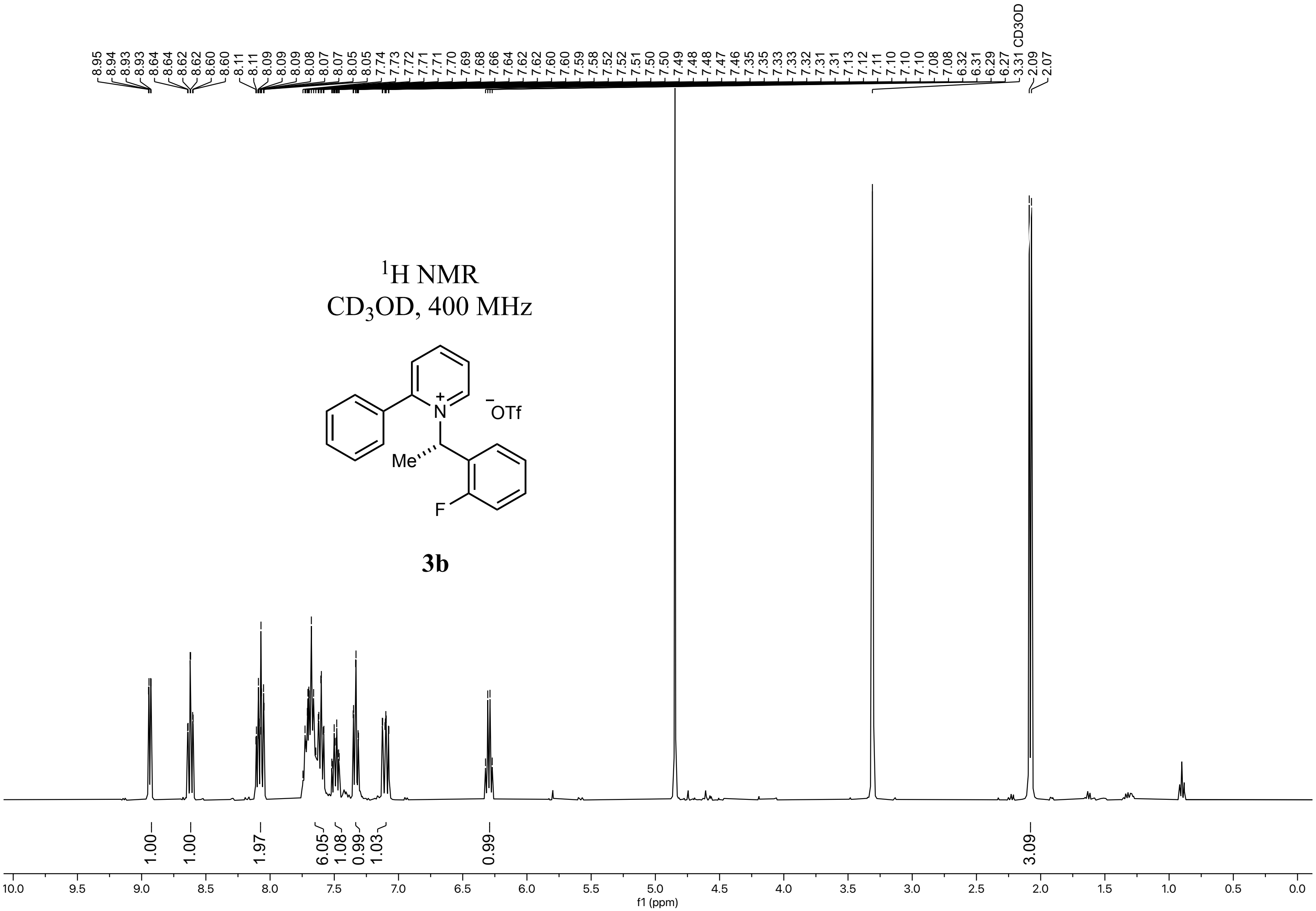

8.95
8.94
8.93
8.93
8.64
8.64
8.62
8.62
8.60
8.60
8.11
8.11
8.09
8.09
8.08
8.07
8.07
8.05
7.74
7.73
7.72
7.71
7.70
7.69
7.68
7.66
7.64
7.62
7.62
7.60
7.60
7.59
7.58
7.52
7.52
7.51
7.50
7.50
7.49
7.48
7.48
7.47
7.46
7.35
7.35
7.33
7.32
7.31
7.31
7.13
7.12
7.11
7.10
7.10
7.08
6.32
6.31
6.29
6.27
3.31 CD3OD
2.09
2.07
1H NMR
CD3OD, 400 MHz
OTf
Me
F
3b
1.00
1.00
1.97
6.05
1.08
0.99
1.03
0.99
3.09
10.0
9.5
9.0
8.5
8.0
7.5
7.0
6.5
6.0
5.5
5.0
4.5
4.0
3.5
3.0
2.5
2.0
1.5
1.0
0.5
0.0
f1 (ppm)

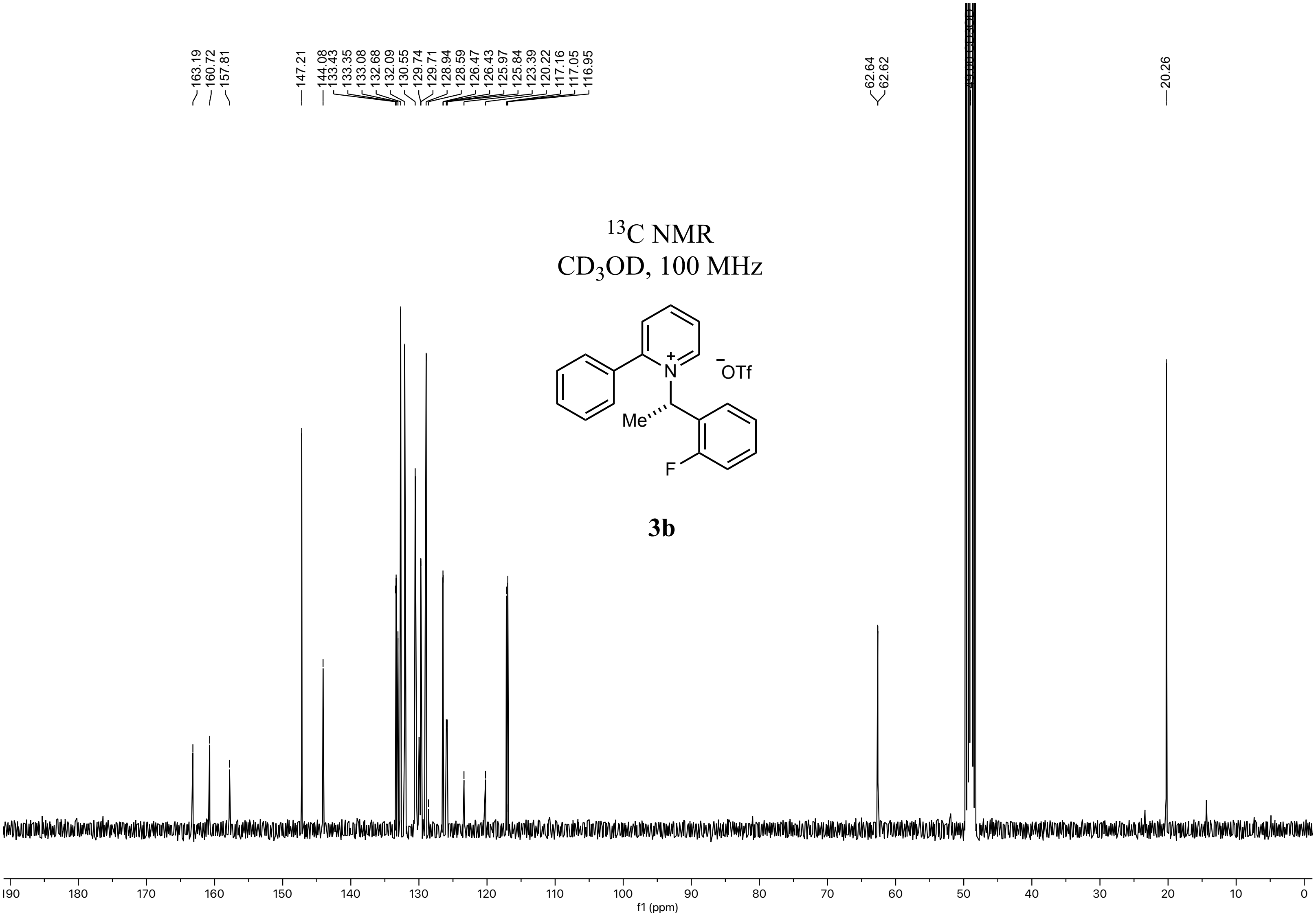
163.19
160.72
157.81
147.21
144.08
133.43
133.35
133.08
132.68
132.09
130.55
129.74
129.71
128.94
128.59
126.47
126.43
125.97
125.84
123.39
120.22
117.16
117.05
116.95
62.64
62.62
20.26
^{13}C NMR
CD_3OD, 100 MHz
OTf
Me
F
3b
f1 (ppm)

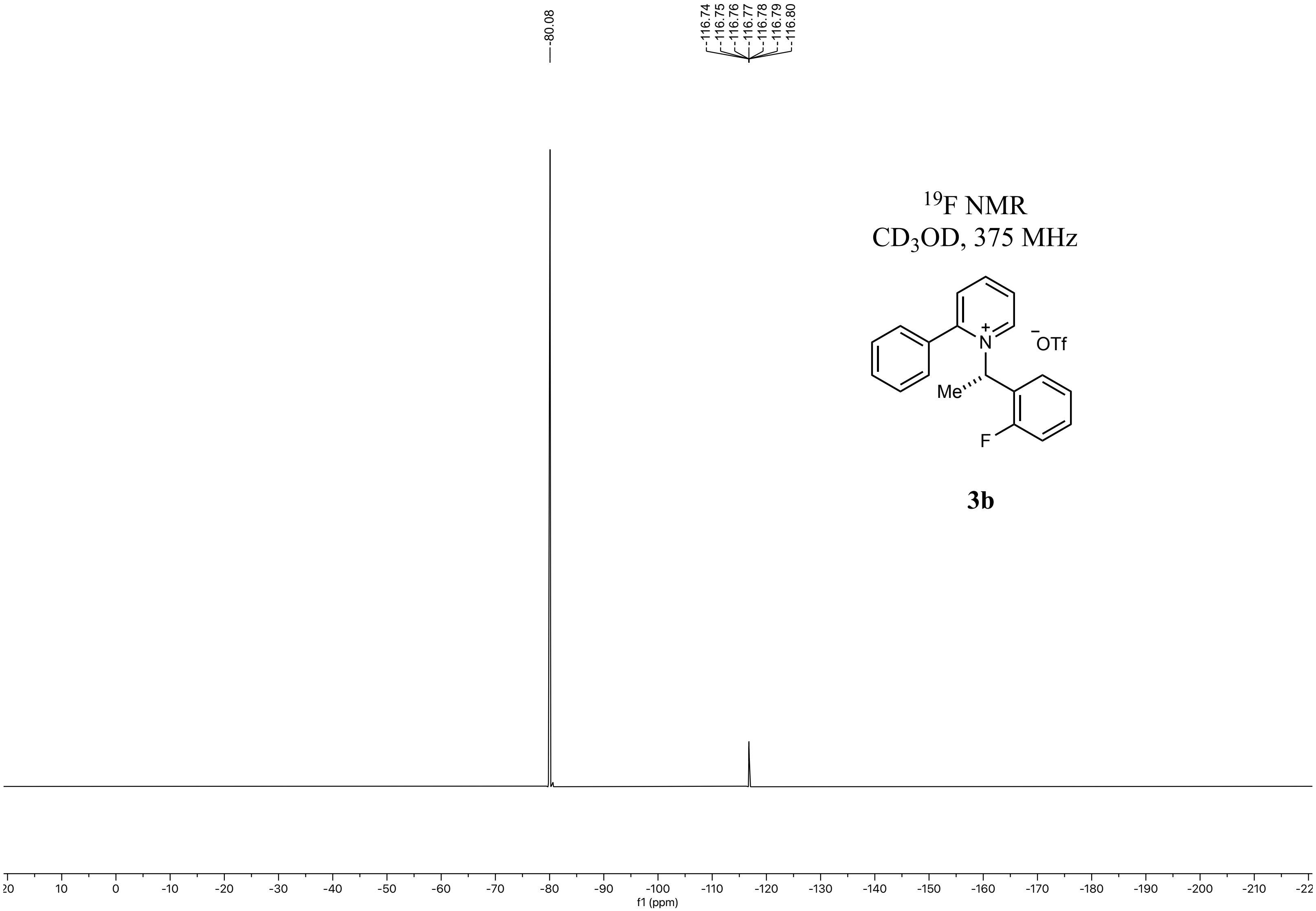

^{19}F NMR
CD_3OD, 375 MHz

3b

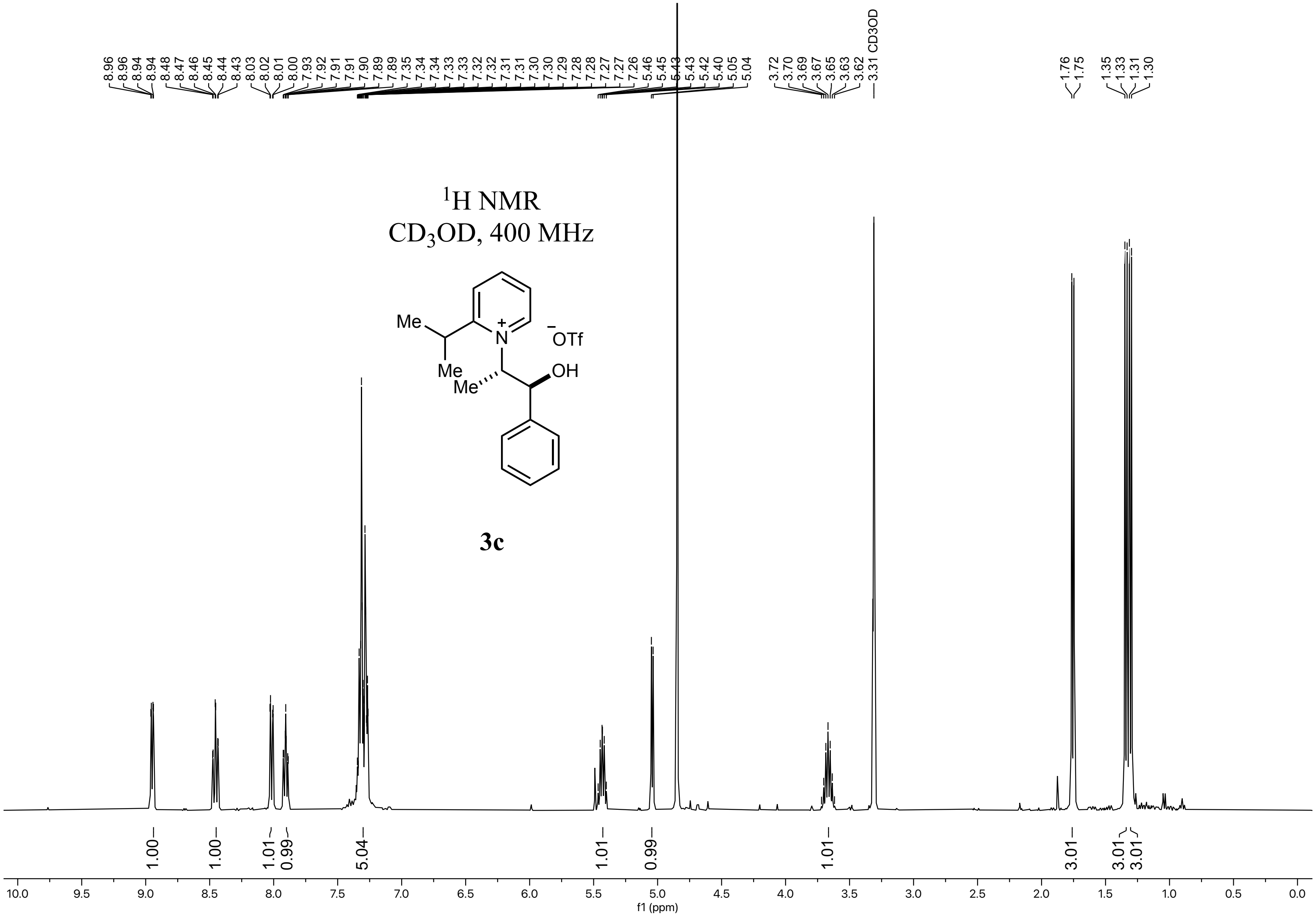

8.96
8.96
8.94
8.94
8.48
8.47
8.46
8.45
8.44
8.43
8.03
8.02
8.01
8.00
7.93
7.92
7.91
7.91
7.90
7.89
7.89
7.35
7.34
7.34
7.33
7.33
7.32
7.32
7.31
7.31
7.30
7.30
7.29
7.28
7.28
7.27
7.27
7.26
5.46
5.45
5.43
5.43
5.42
5.40
5.05
5.04
3.72
3.70
3.69
3.67
3.65
3.63
3.62
3.31 CD3OD
1.76
1.75
1.35
1.33
1.31
1.30
^{1}H NMR
CD_3OD, 400 MHz
Me
Me
Me
N
OH
OTf
3c
1.00
1.00
1.01
0.99
5.04
1.01
0.99
1.01
3.01
3.01
3.01
f1 (ppm)

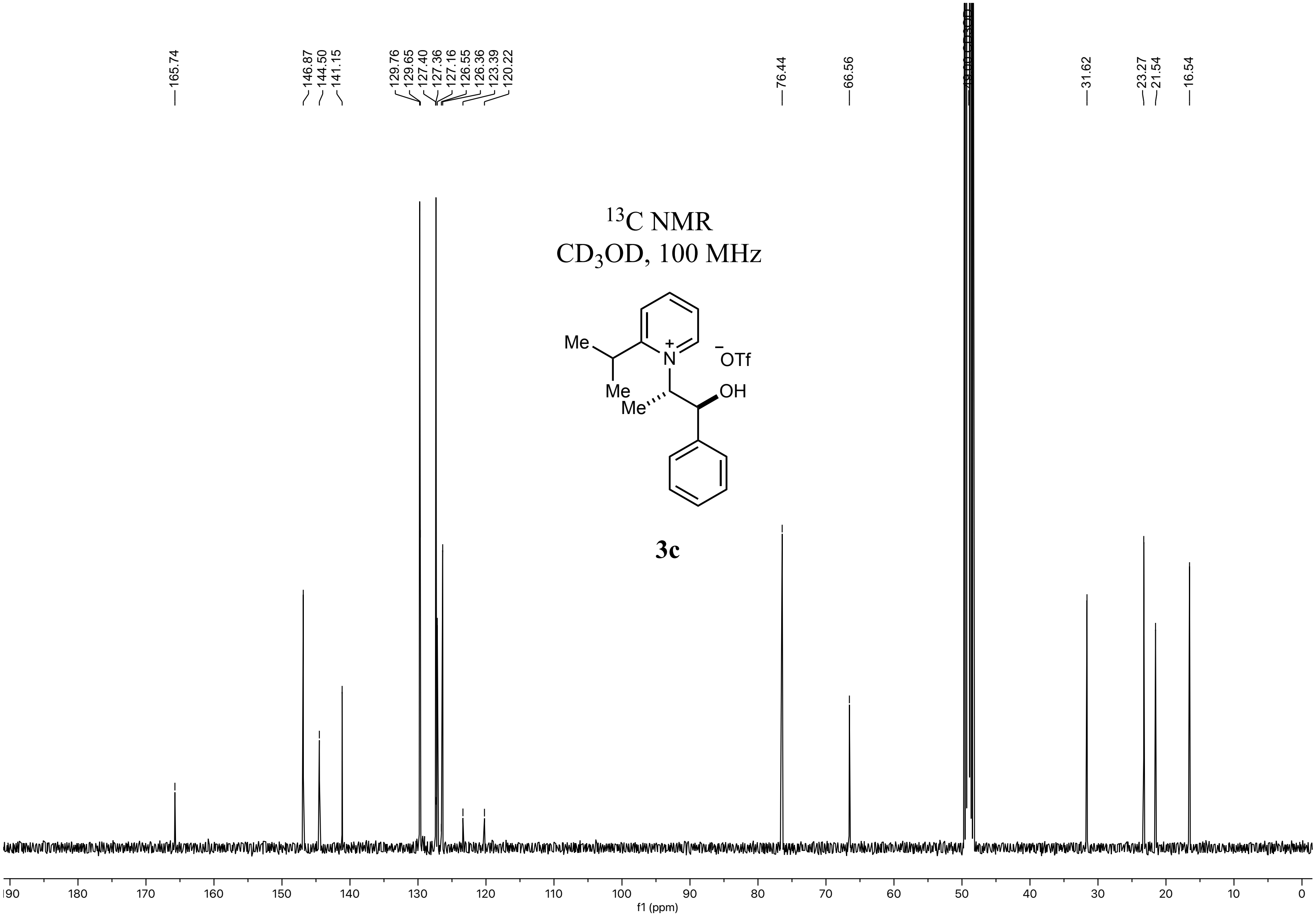

165.74
146.87
144.50
141.15
129.76
129.65
127.40
127.36
127.16
126.55
126.36
123.39
120.22
76.44
66.56
31.62
23.27
21.54
16.54
^{13}C NMR
CD_3OD, 100 MHz
Me
Me
Me
N
OTf
OH
3c
f1 (ppm)

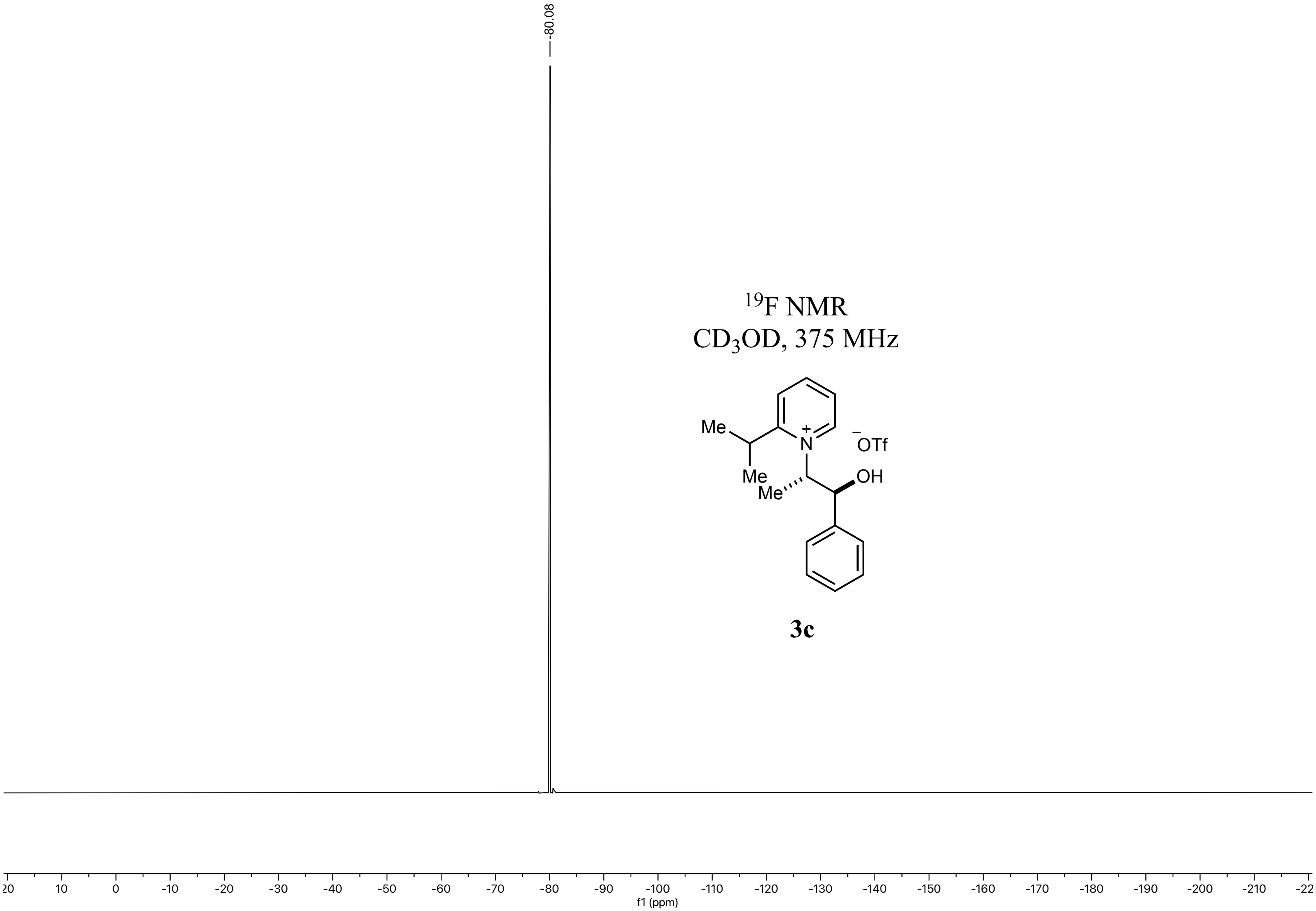

-80.08
19F NMR
CD3OD, 375 MHz
Me
Me
Me
N
+
−
OTf
OH
3c
20 10 0 -10 -20 -30 -40 -50 -60 -70 -80 -90 -100 -110 -120 -130 -140 -150 -160 -170 -180 -190 -200 -210 -220
f1 (ppm)

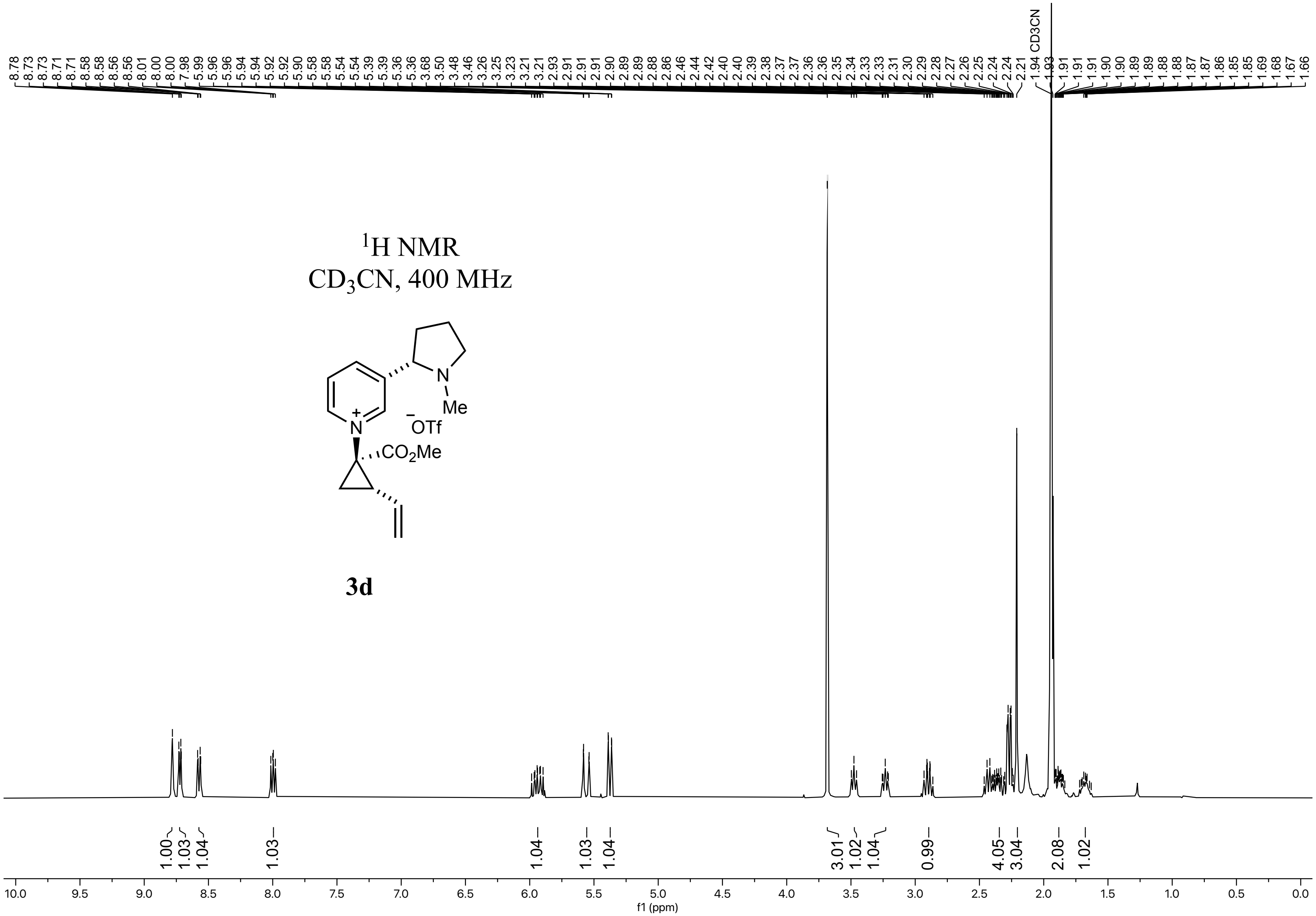
^{1}H NMR
CD_3CN, 400 MHz
3d
CD3CN
f1 (ppm)

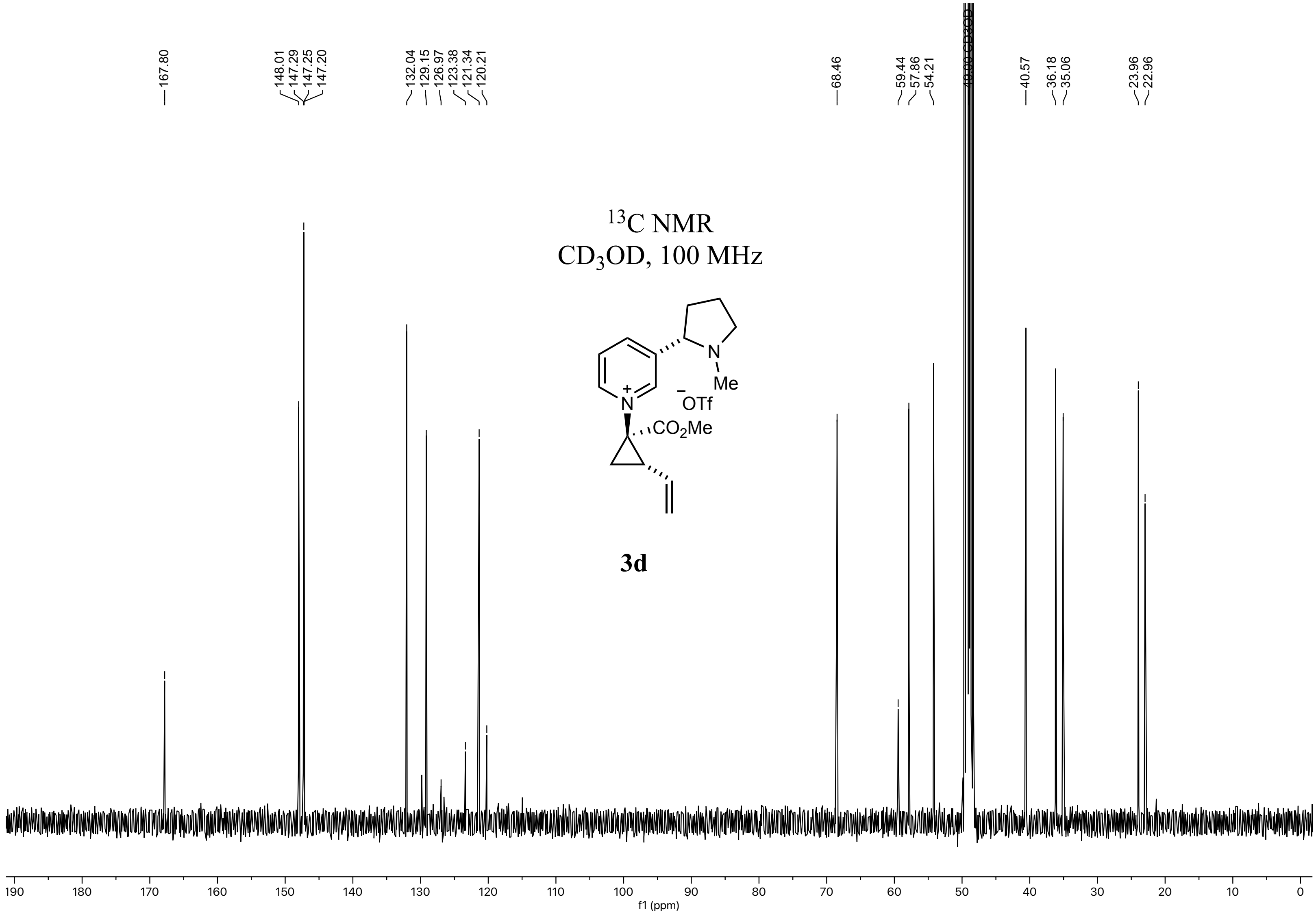
167.80
148.01
147.29
147.25
147.20
132.04
129.15
126.97
123.38
121.34
120.21
68.46
59.44
57.86
54.21
CD3OD
40.57
36.18
35.06
23.96
22.96
13C NMR
CD3OD, 100 MHz
N
Me
OTf
CO2Me
3d
190 180 170 160 150 140 130 120 110 100 90 80 70 60 50 40 30 20 10 0
f1 (ppm)

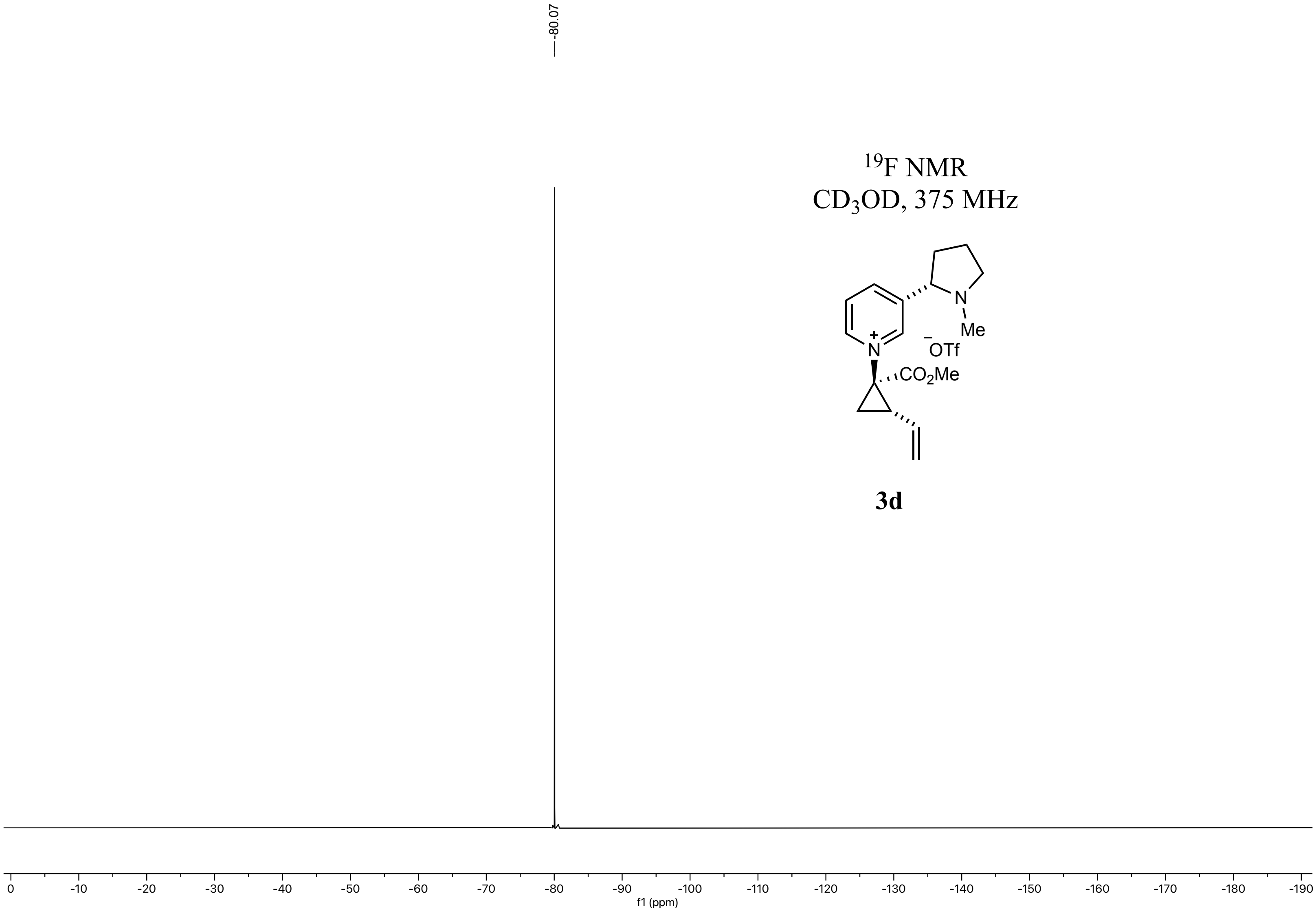
-80.07
^{19}F NMR
CD_3OD, 375 MHz
N
Me
OTf
N
CO2Me
3d
0
-10
-20
-30
-40
-50
-60
-70
-80
-90
-100
-110
-120
-130
-140
-150
-160
-170
-180
-190
f1 (ppm)

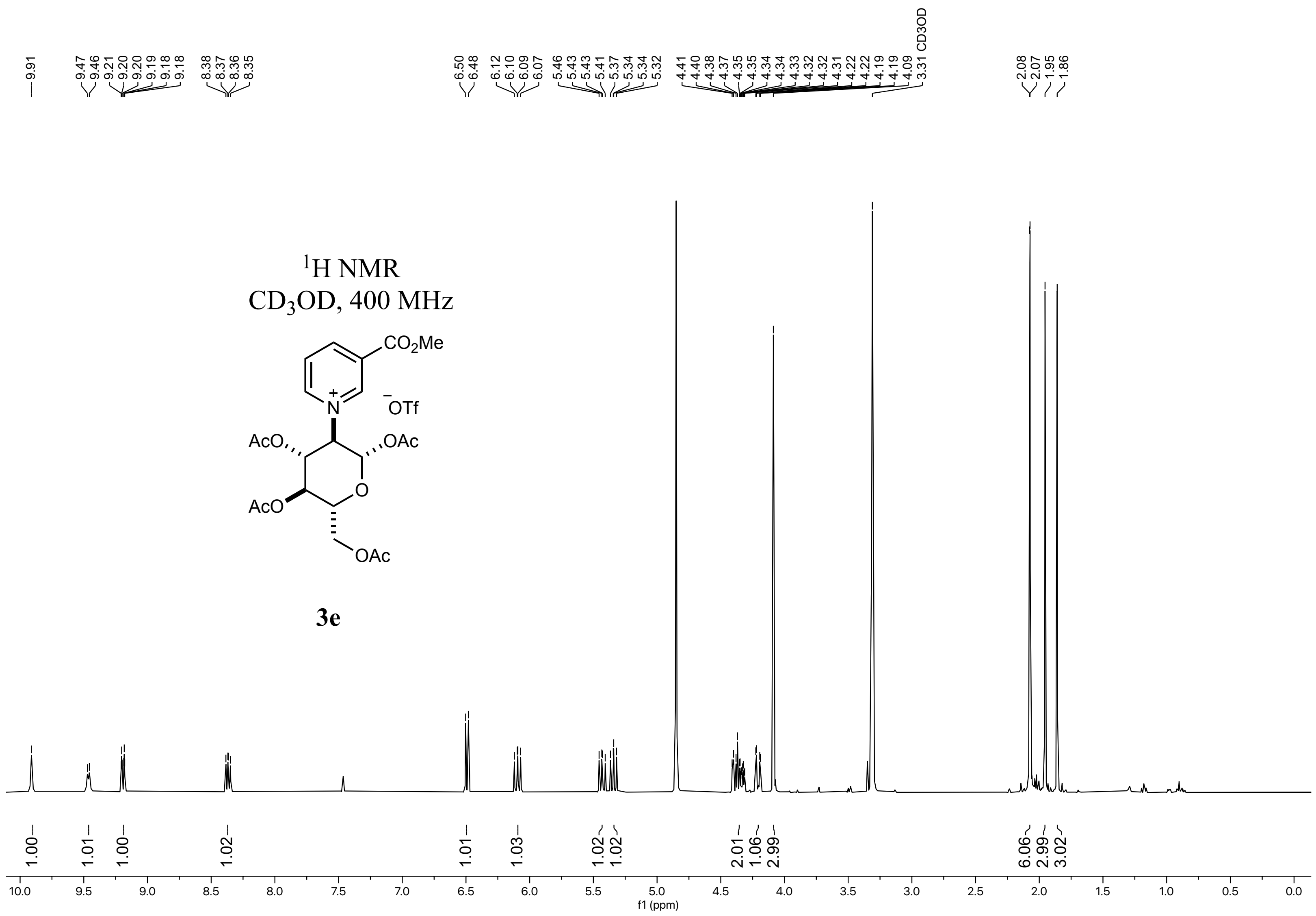

1H NMR
CD3OD, 400 MHz
3e
9.91
9.47 9.46 9.21 9.20 9.20 9.19 9.18 9.18
8.38 8.37 8.36 8.35
6.50 6.48
6.12 6.10 6.09 6.07
5.46 5.43 5.43 5.41 5.37 5.34 5.34 5.32
4.41 4.40 4.38 4.37 4.35 4.35 4.34 4.34 4.33 4.32 4.32 4.31 4.22 4.22 4.19 4.19 4.09
3.31 CD3OD
2.08 2.07 1.95 1.86
1.00 1.01 1.00 1.02 1.01 1.03 1.02 1.02 2.01 1.06 2.99 6.06 2.99 3.02
f1 (ppm)

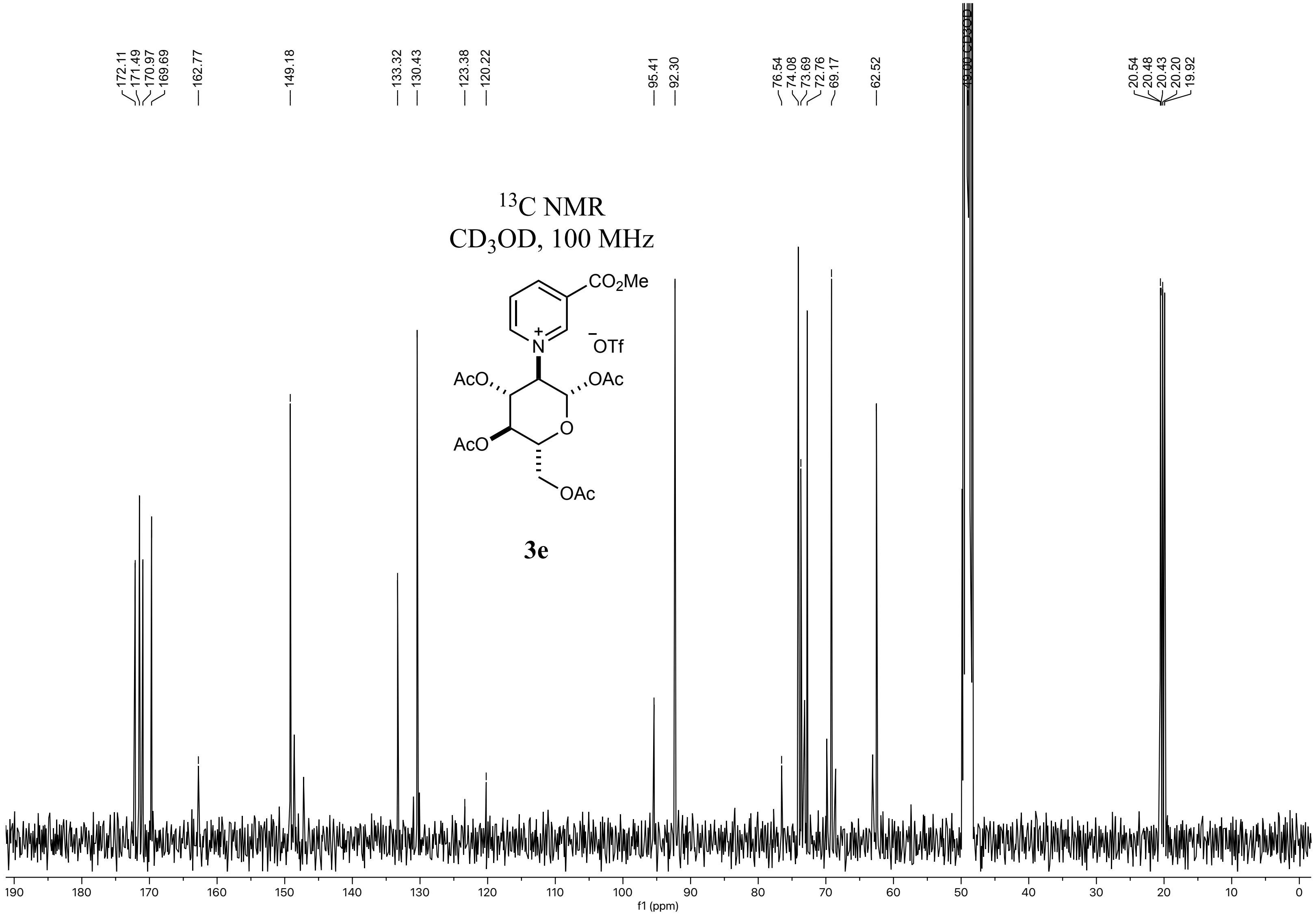
172.11
171.49
170.97
169.69
162.77
149.18
133.32
130.43
123.38
120.22
95.41
92.30
76.54
74.08
73.69
72.76
69.17
62.52
49.00 CD3OD
20.54
20.48
20.43
20.20
19.92
^{13}C NMR
CD_3OD, 100 MHz
CO_2Me
^-OTf
AcO
OAc
AcO
O
OAc
3e
190
180
170
160
150
140
130
120
110
100
90
80
70
60
50
40
30
20
10
0
f1 (ppm)

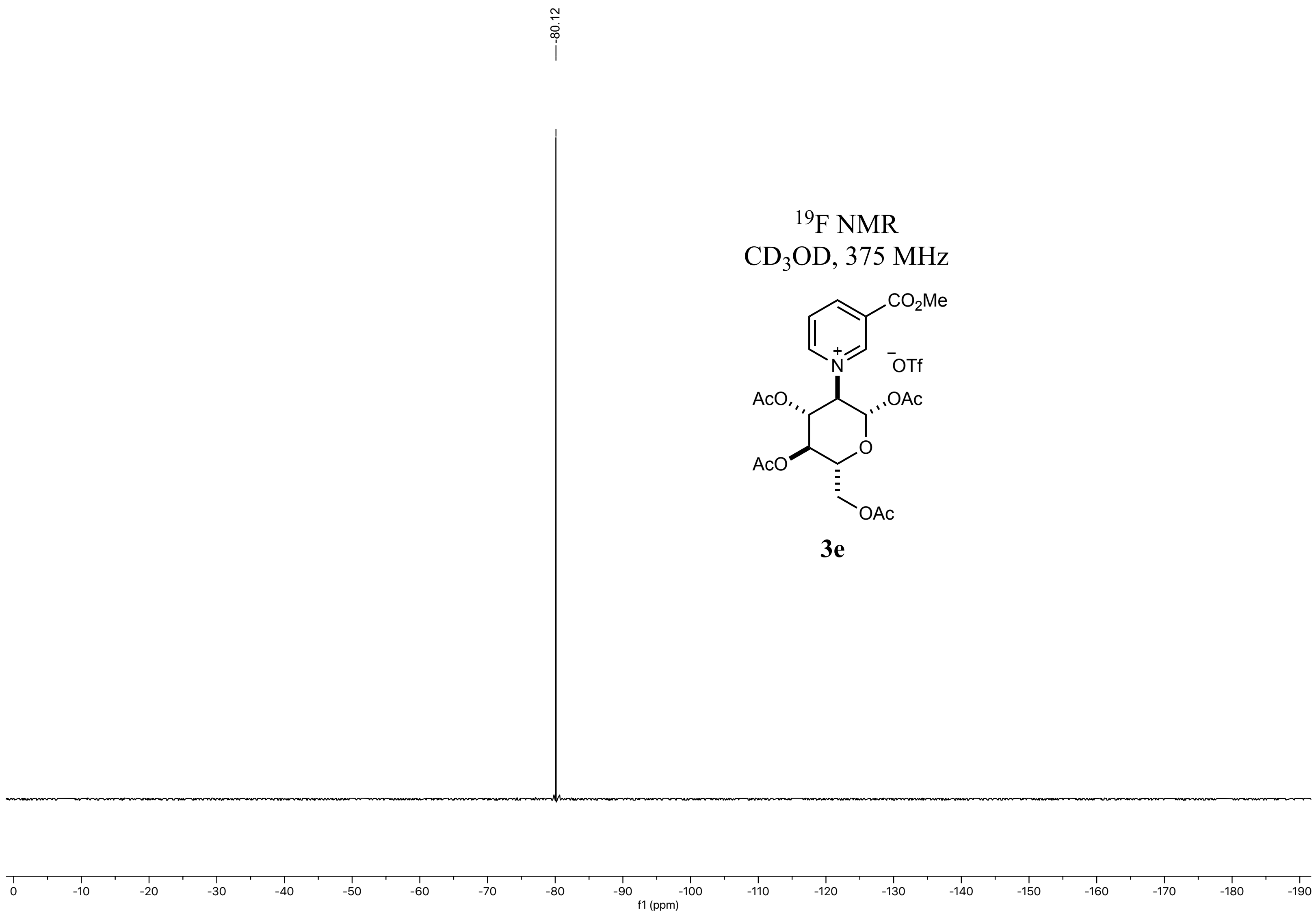
-80.12
19F NMR
CD3OD, 375 MHz
CO2Me
N
OTf
AcO
OAc
AcO
O
OAc
3e
0
-10
-20
-30
-40
-50
-60
-70
-80
-90
-100
-110
-120
-130
-140
-150
-160
-170
-180
-190
f1 (ppm)

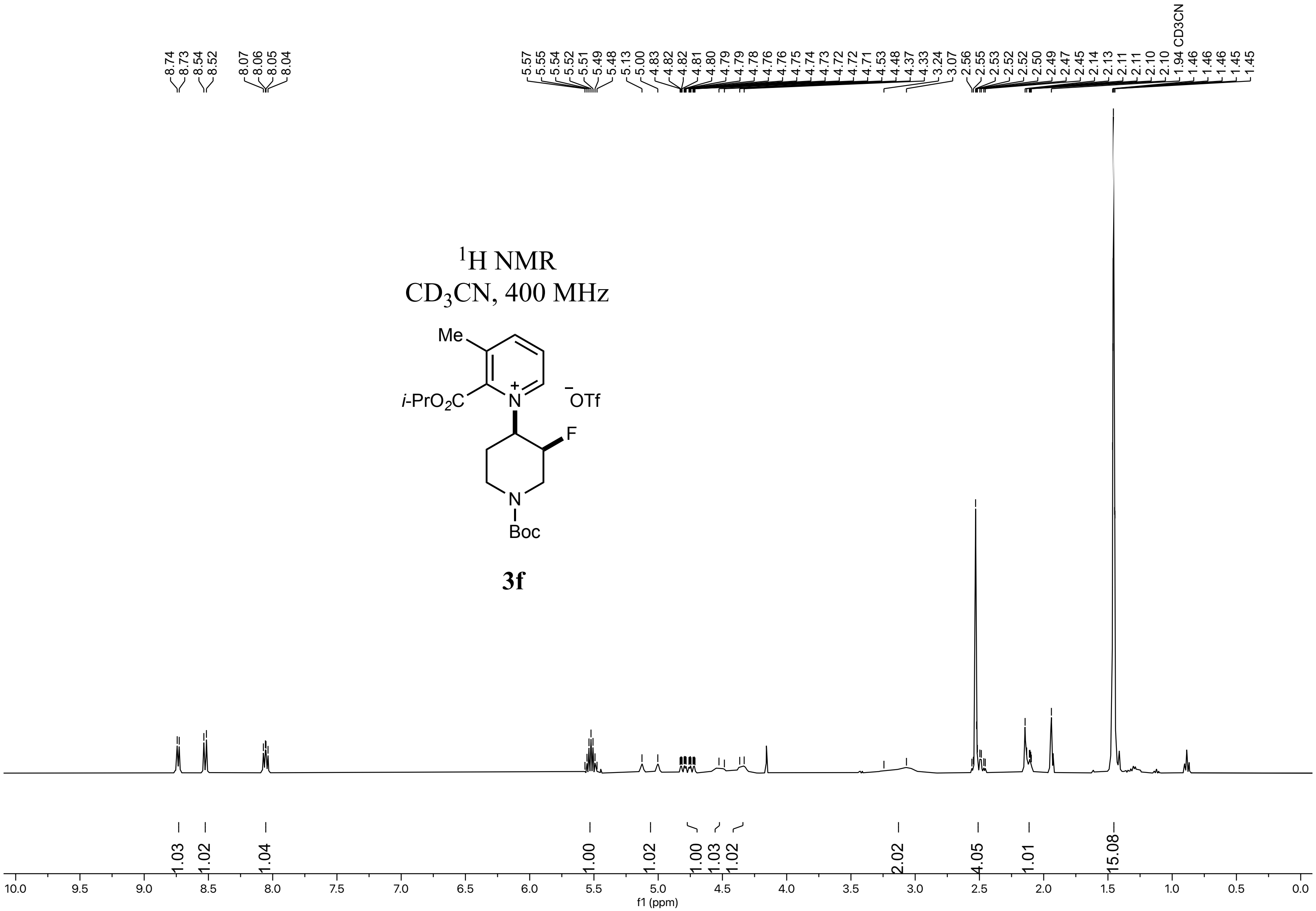
8.74
8.73
8.54
8.52
8.07
8.06
8.05
8.04
5.57
5.55
5.54
5.52
5.51
5.49
5.48
5.13
5.00
4.83
4.82
4.82
4.81
4.80
4.79
4.79
4.78
4.76
4.76
4.75
4.74
4.73
4.72
4.72
4.71
4.53
4.48
4.37
4.33
3.24
3.07
2.56
2.55
2.53
2.52
2.52
2.50
2.49
2.47
2.45
2.14
2.13
2.11
2.11
2.10
1.94 CD3CN
1.46
1.46
1.46
1.45
1.45
1H NMR
CD3CN, 400 MHz
Me
i-PrO2C
N
+
−
OTf
F
N
Boc
3f
1.03
1.02
1.04
1.00
1.02
1.00
1.03
1.02
2.02
4.05
1.01
15.08
f1 (ppm)

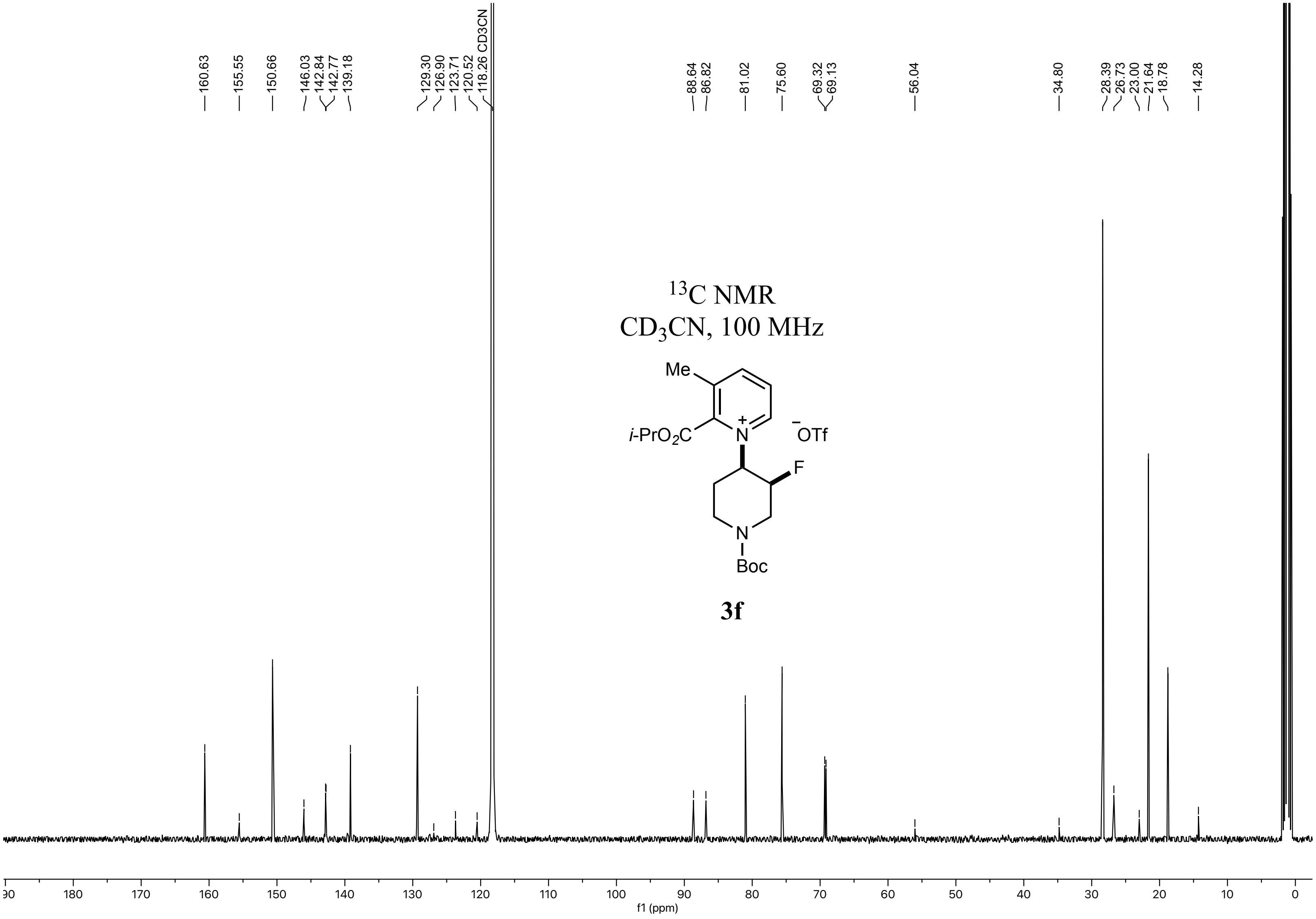
160.63
155.55
150.66
146.03
142.84
142.77
139.18
129.30
126.90
123.71
120.52
118.26 CD3CN
88.64
86.82
81.02
75.60
69.32
69.13
56.04
34.80
28.39
26.73
23.00
21.64
18.78
14.28
^{13}C NMR
CD_3CN, 100 MHz
Me
i-PrO_2C
N
$^-$OTf
F
N
Boc
3f
f1 (ppm)

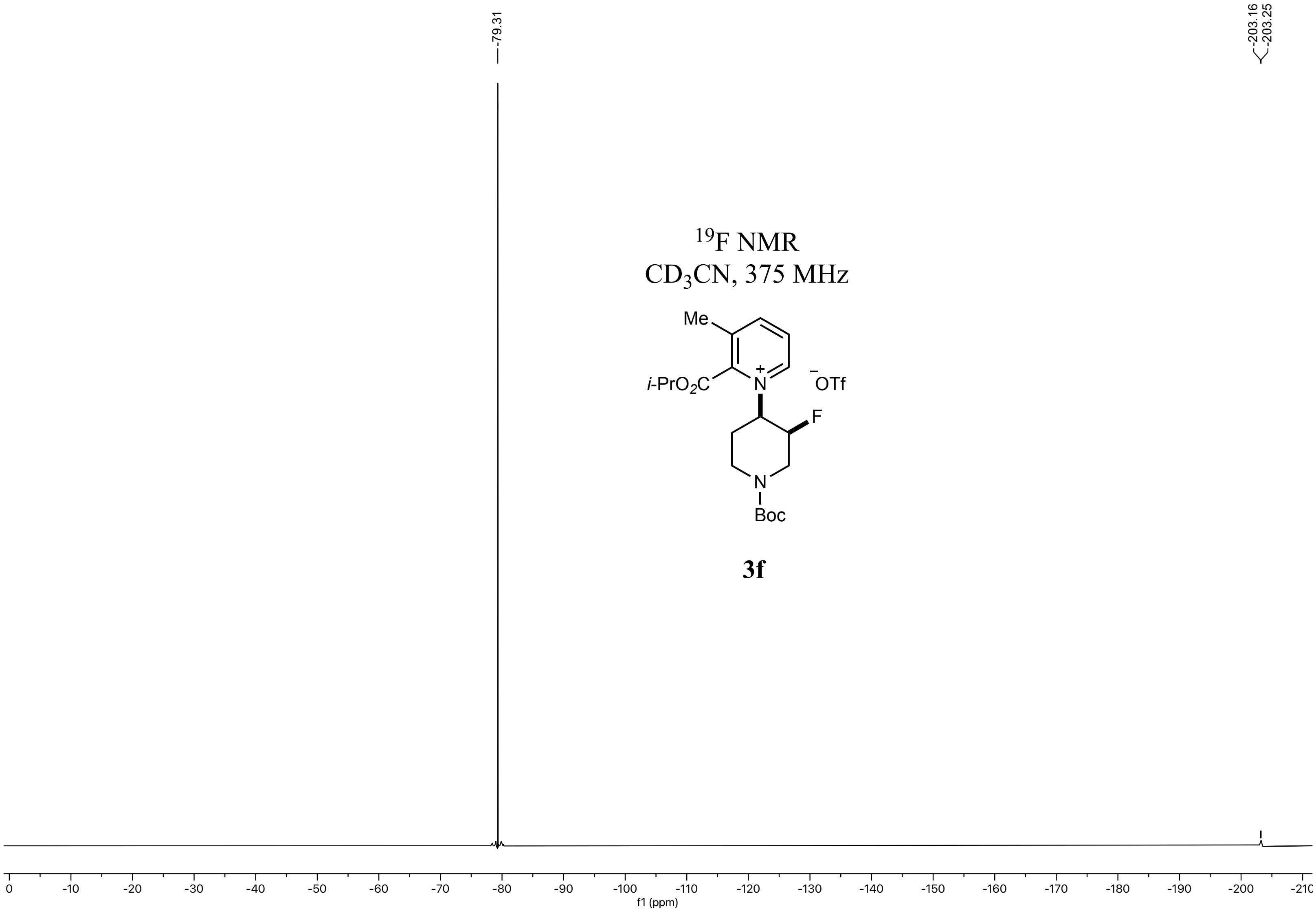

-79.31
-203.16
-203.25
^{19}F NMR
CD_3CN, 375 MHz
Me
i-PrO2C
OTf
F
N
Boc
3f
0
-10
-20
-30
-40
-50
-60
-70
-80
-90
-100
-110
-120
-130
-140
-150
-160
-170
-180
-190
-200
-210
f1 (ppm)

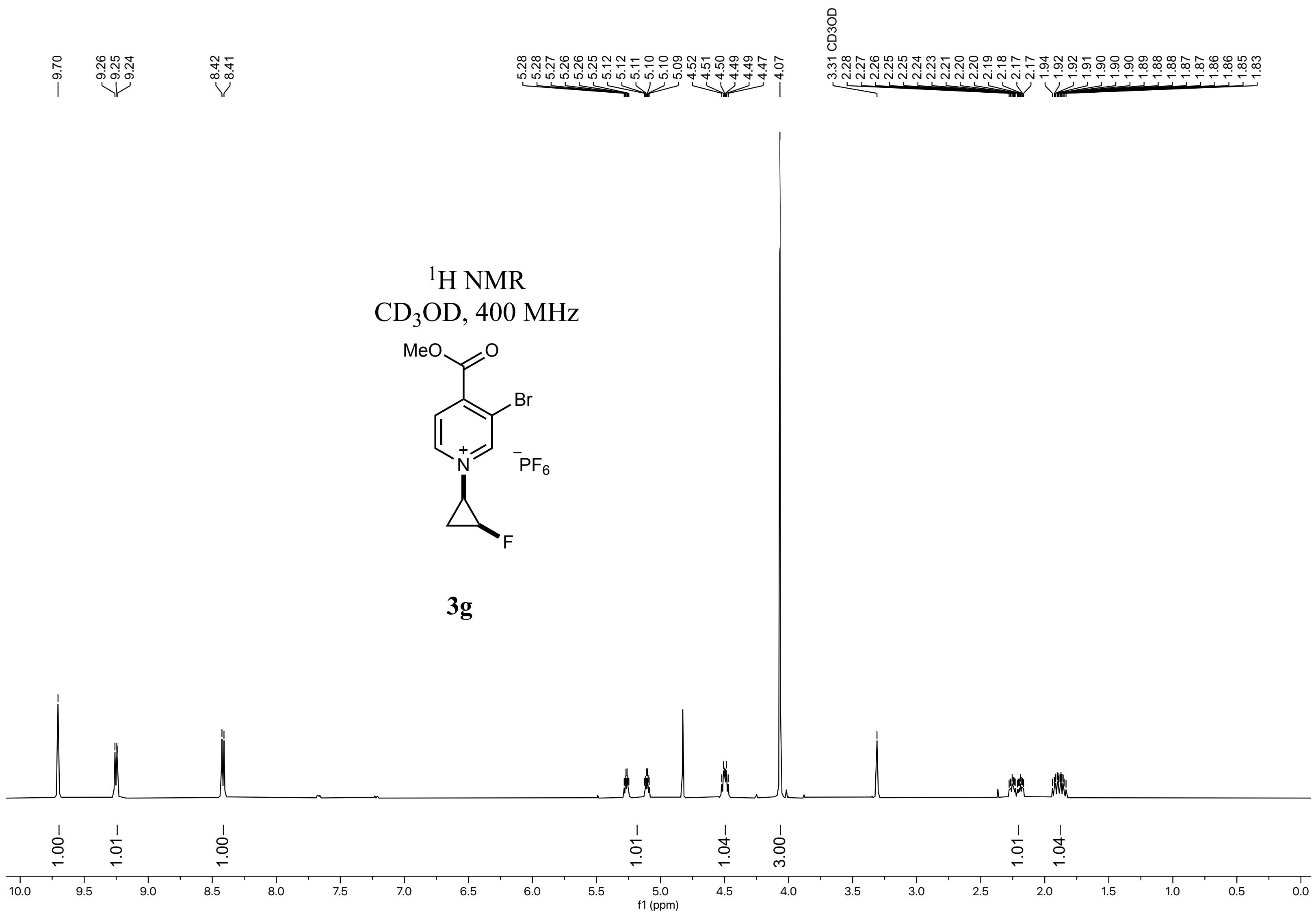

^{1}H NMR
CD_3OD, 400 MHz
MeO
O
Br
N
PF6
F
3g
f1 (ppm)

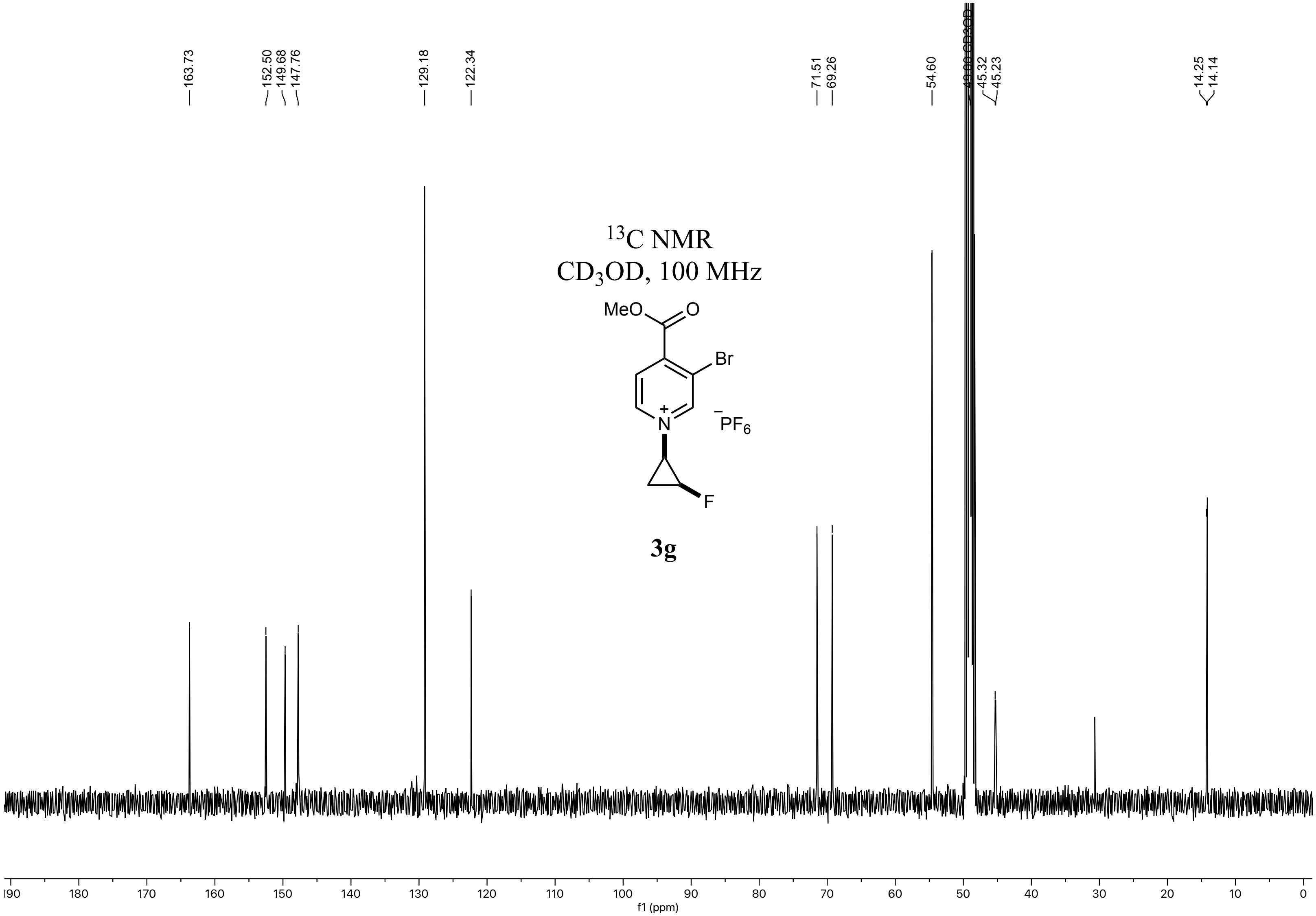

163.73
152.50
149.68
147.76
129.18
122.34
71.51
69.26
54.60
45.32
45.23
14.25
14.14
^{13}C NMR
CD_3OD, 100 MHz
MeO
O
Br
N
+
−
PF_6
F
3g
190
180
170
160
150
140
130
120
110
100
90
80
70
60
50
40
30
20
10
0
f1 (ppm)

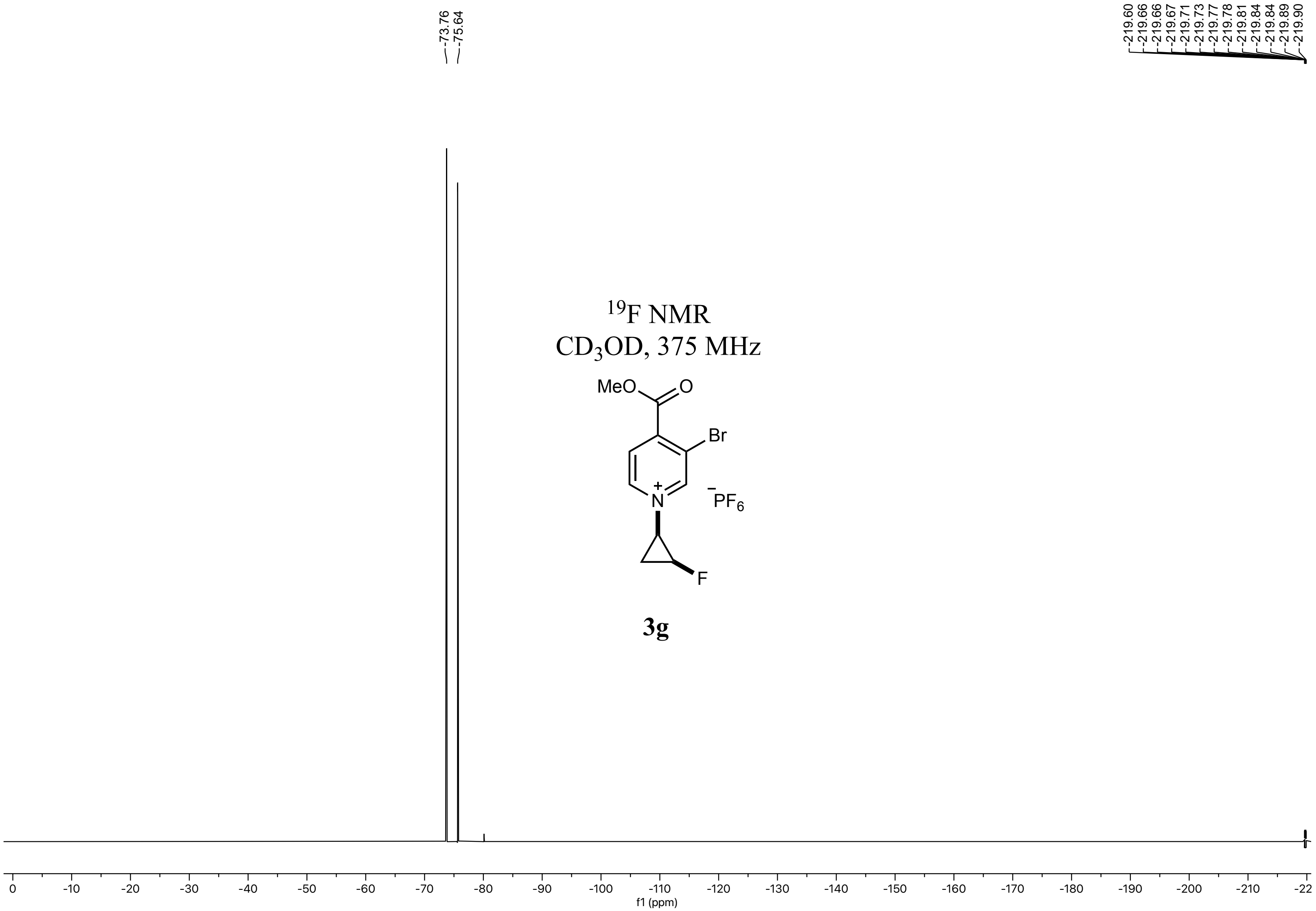
-73.76
-75.64
-219.60
-219.66
-219.66
-219.67
-219.71
-219.73
-219.77
-219.78
-219.81
-219.84
-219.84
-219.89
-219.90
^{19}F NMR
CD_3OD, 375 MHz
MeO
O
Br
N
$^-PF_6$
F
f1 (ppm)

3g

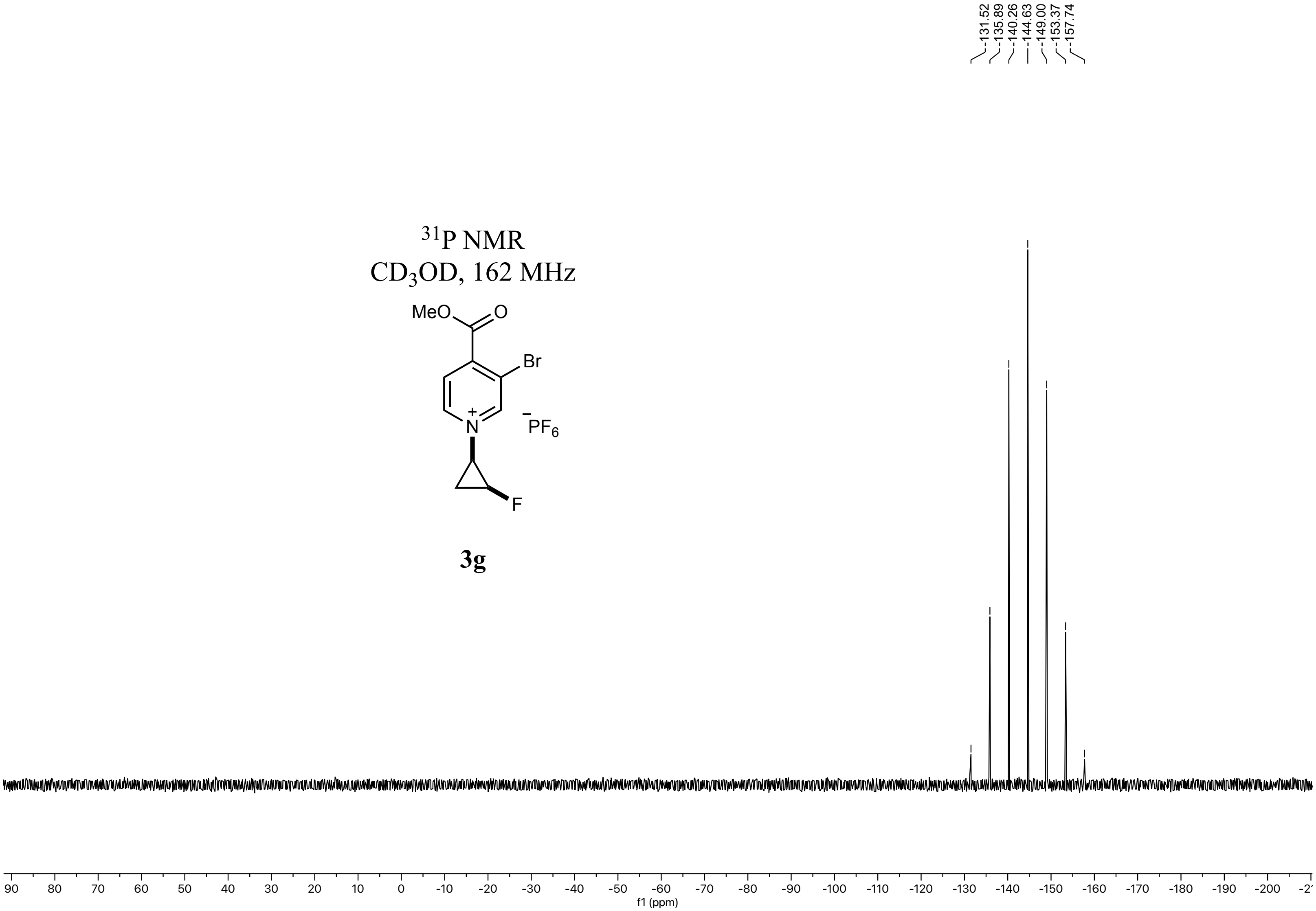

-131.52
-135.89
-140.26
-144.63
-149.00
-153.37
-157.74
^{31}P NMR
CD$_3$OD, 162 MHz
MeO
O
Br
N
PF6
F
3g
90
80
70
60
50
40
30
20
10
0
-10
-20
-30
-40
-50
-60
-70
-80
-90
-100
-110
-120
-130
-140
-150
-160
-170
-180
-190
-200
f1 (ppm)

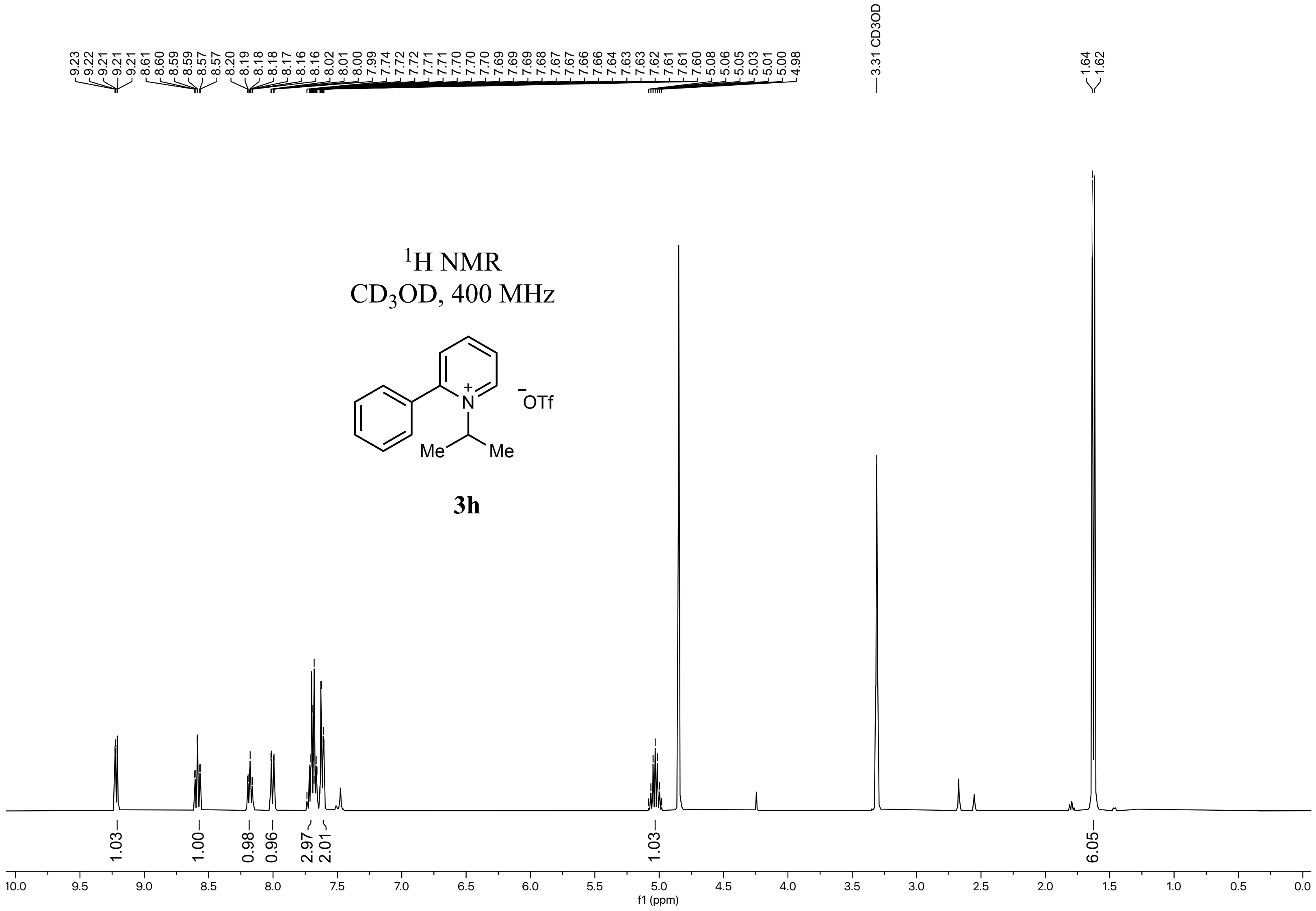

^{1}H NMR
CD_3OD, 400 MHz
OTf
N
Me
Me
3h
3.31 CD3OD
f1 (ppm)

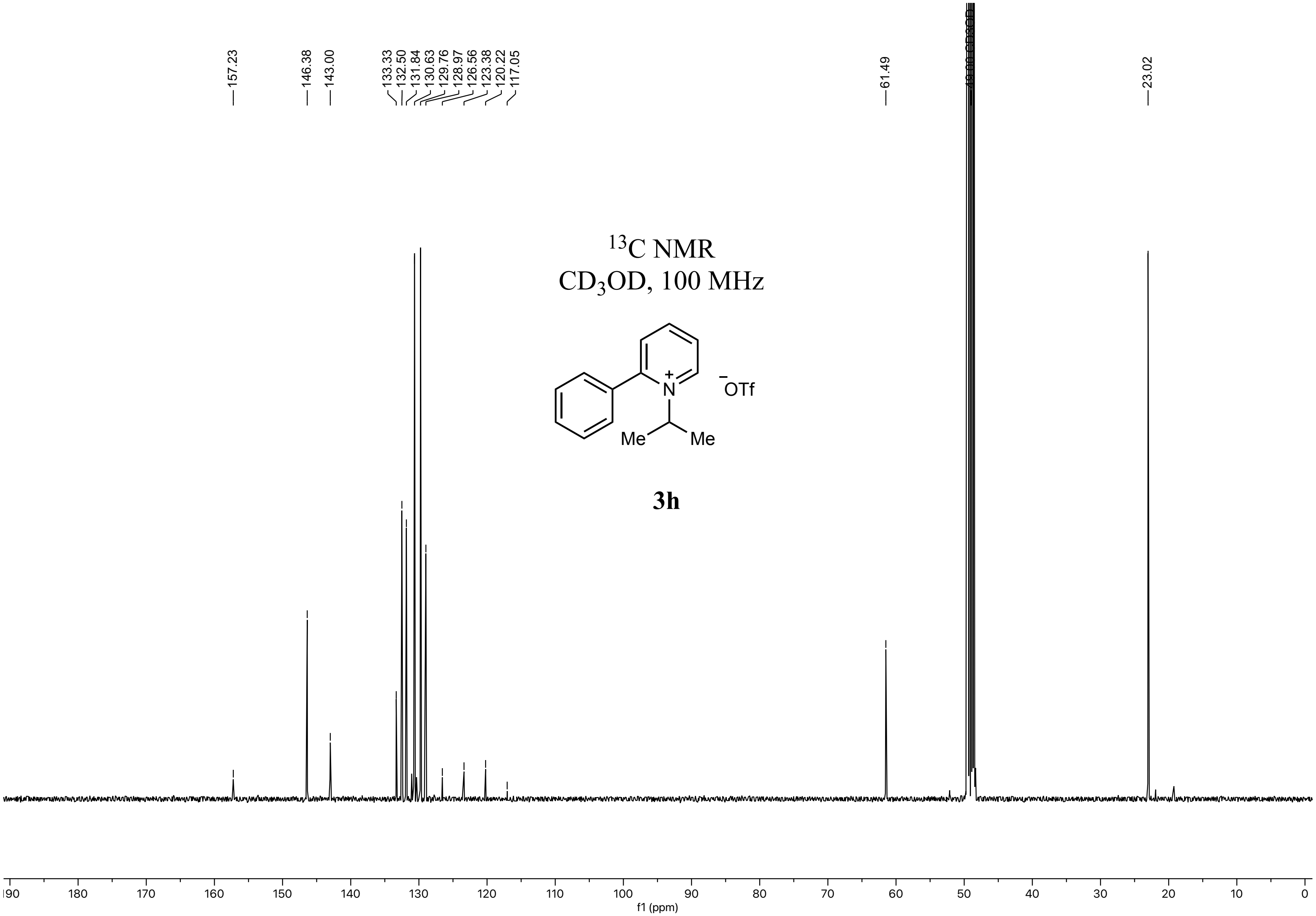
157.23
146.38
143.00
133.33
132.50
131.84
130.63
129.76
128.97
126.56
123.38
120.22
117.05
61.49
23.02
^{13}C NMR
CD_3OD, 100 MHz
N
+
−OTf
Me
Me
3h
190
180
170
160
150
140
130
120
110
100
90
80
70
60
50
40
30
20
10
0
f1 (ppm)

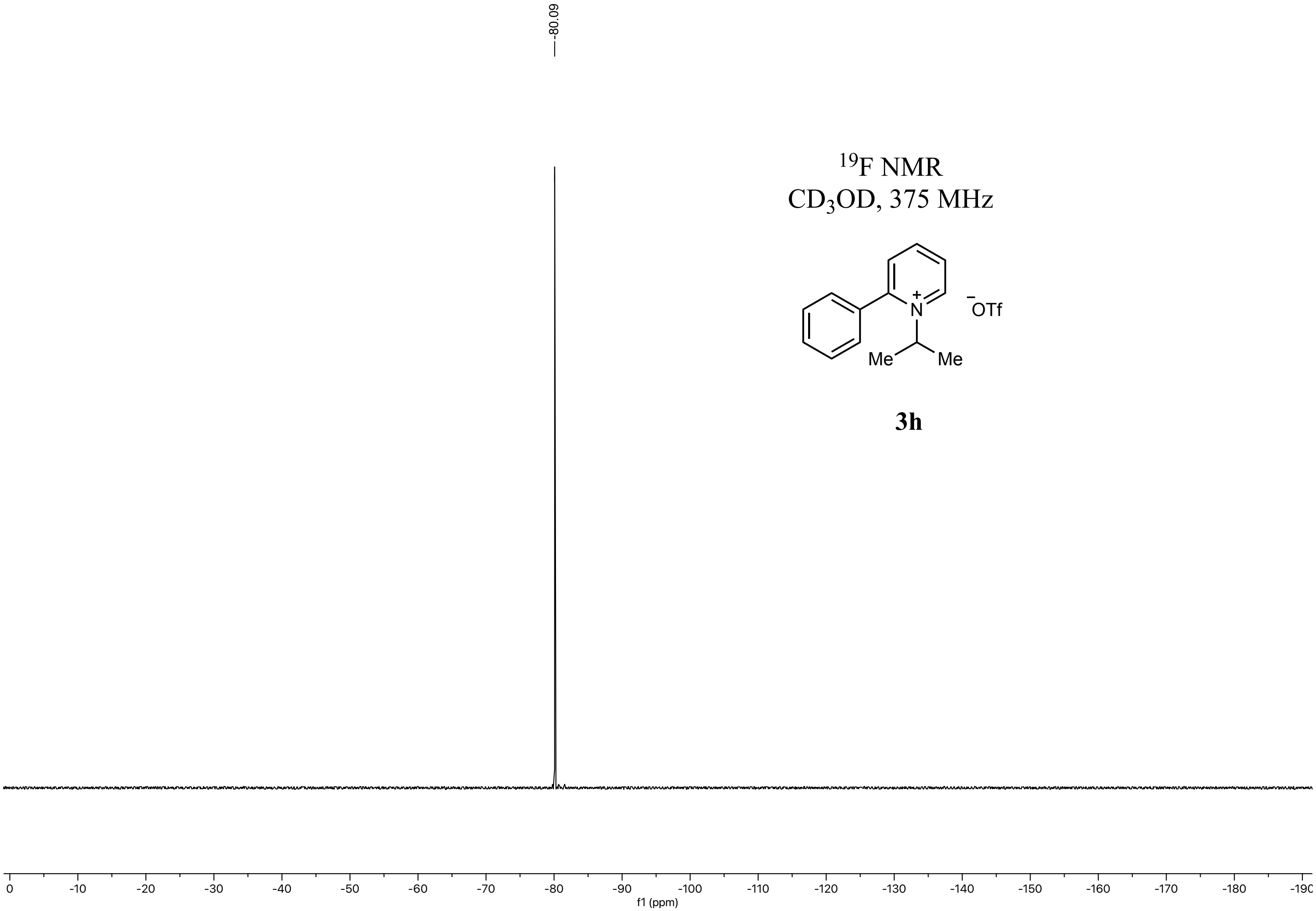
-80.09
^{19}F NMR
CD_3OD, 375 MHz
N
OTf
Me
Me
0
-10
-20
-30
-40
-50
-60
-70
-80
-90
-100
-110
-120
-130
-140
-150
-160
-170
-180
-190
f1 (ppm)

3h

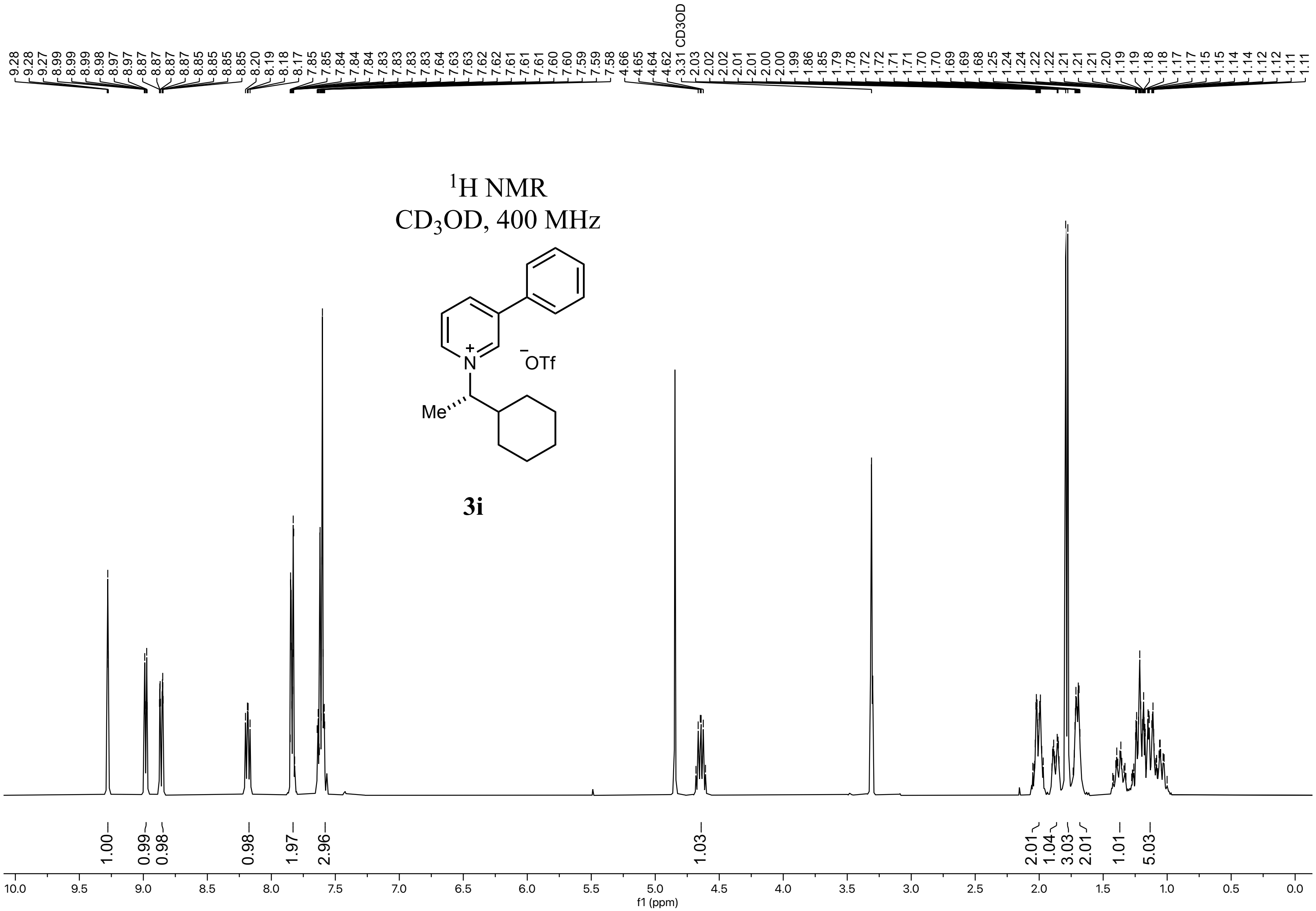

CD3OD
^{1}H NMR
CD_3OD, 400 MHz
N
+
−OTf
Me
3i
1.00
0.99
0.98
0.98
1.97
2.96
1.03
2.01
1.04
3.03
2.01
1.01
5.03
f1 (ppm)

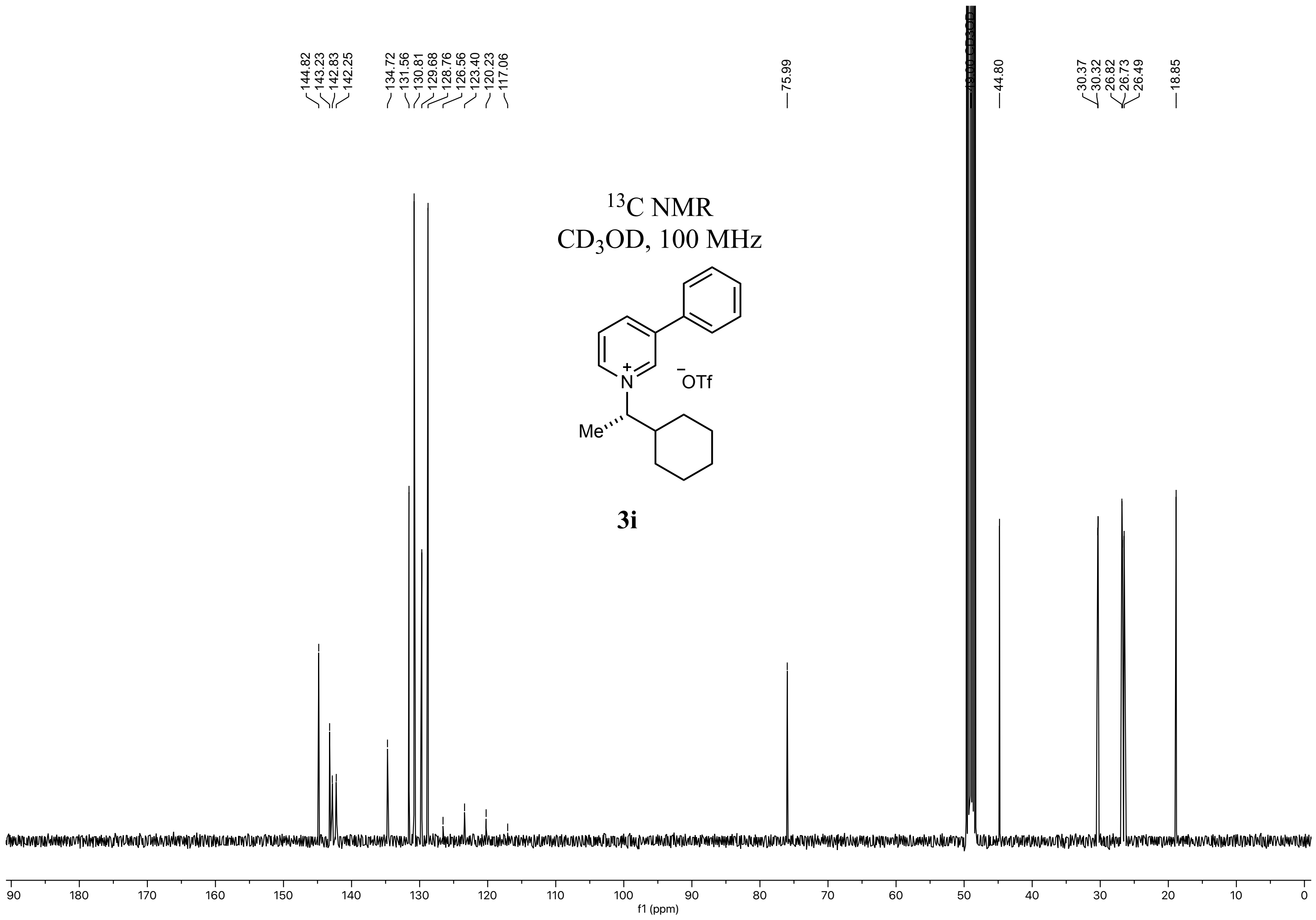
144.82
143.23
142.83
142.25
134.72
131.56
130.81
129.68
128.76
126.56
123.40
120.23
117.06
75.99
44.80
30.37
30.32
26.82
26.73
26.49
18.85
^{13}C NMR
CD_3OD, 100 MHz
OTf
Me
3i
f1 (ppm)

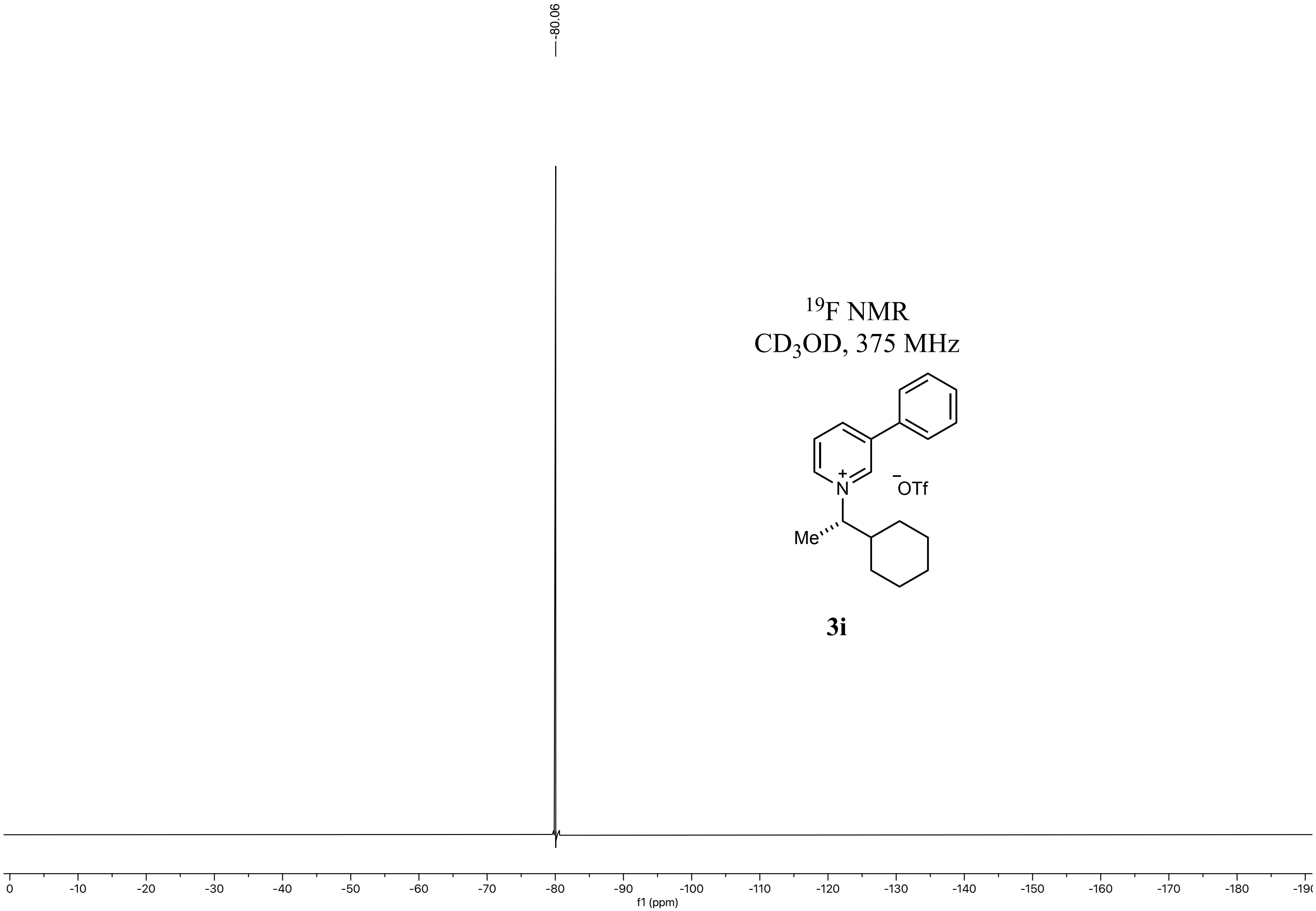
-80.06
^{19}F NMR
CD_3OD, 375 MHz
OTf
Me
N
0
-10
-20
-30
-40
-50
-60
-70
-80
-90
-100
-110
-120
-130
-140
-150
-160
-170
-180
-190
f1 (ppm)

3i

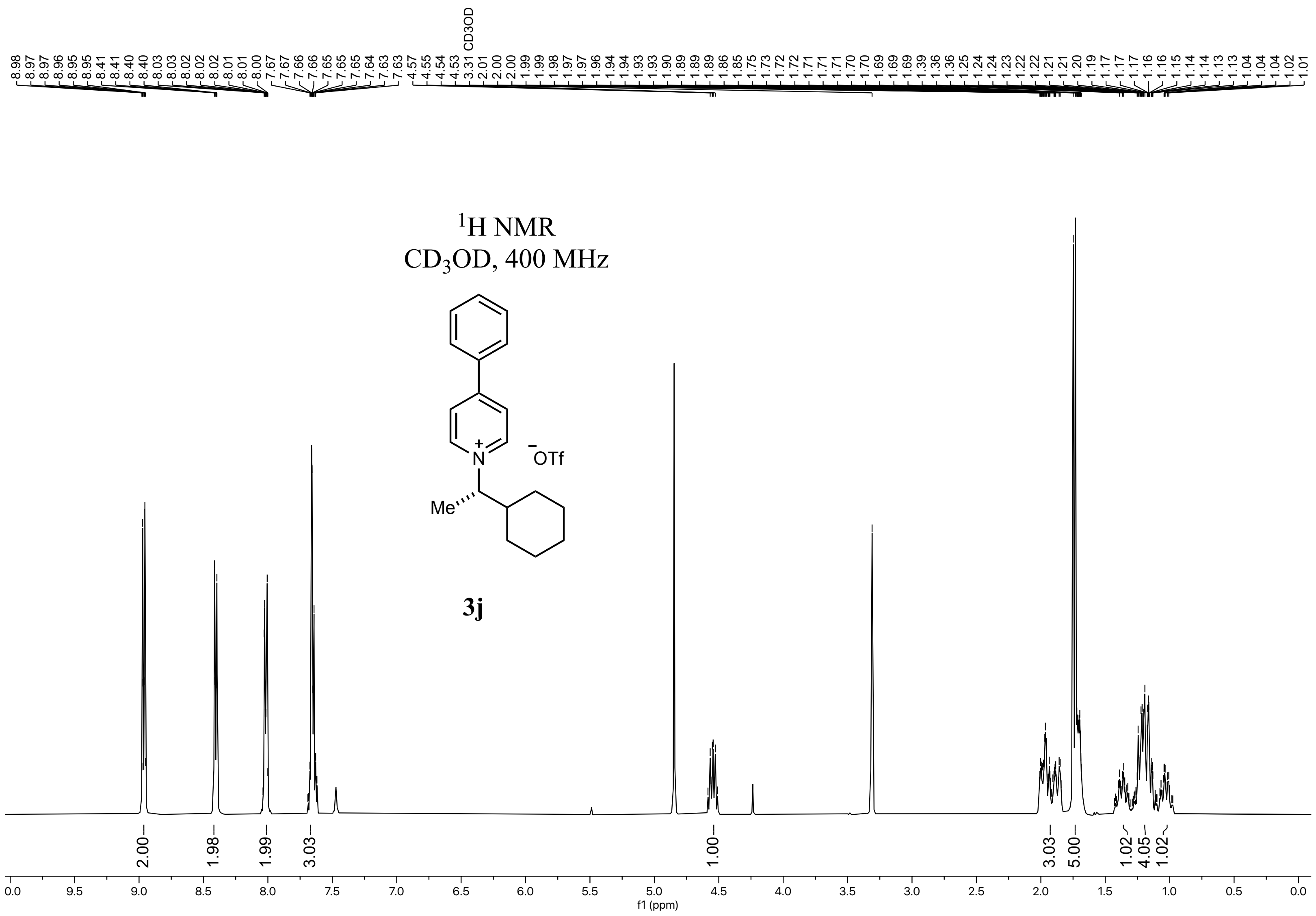

^{1}H NMR
CD$_3$OD, 400 MHz
OTf
Me
3j
f1 (ppm)

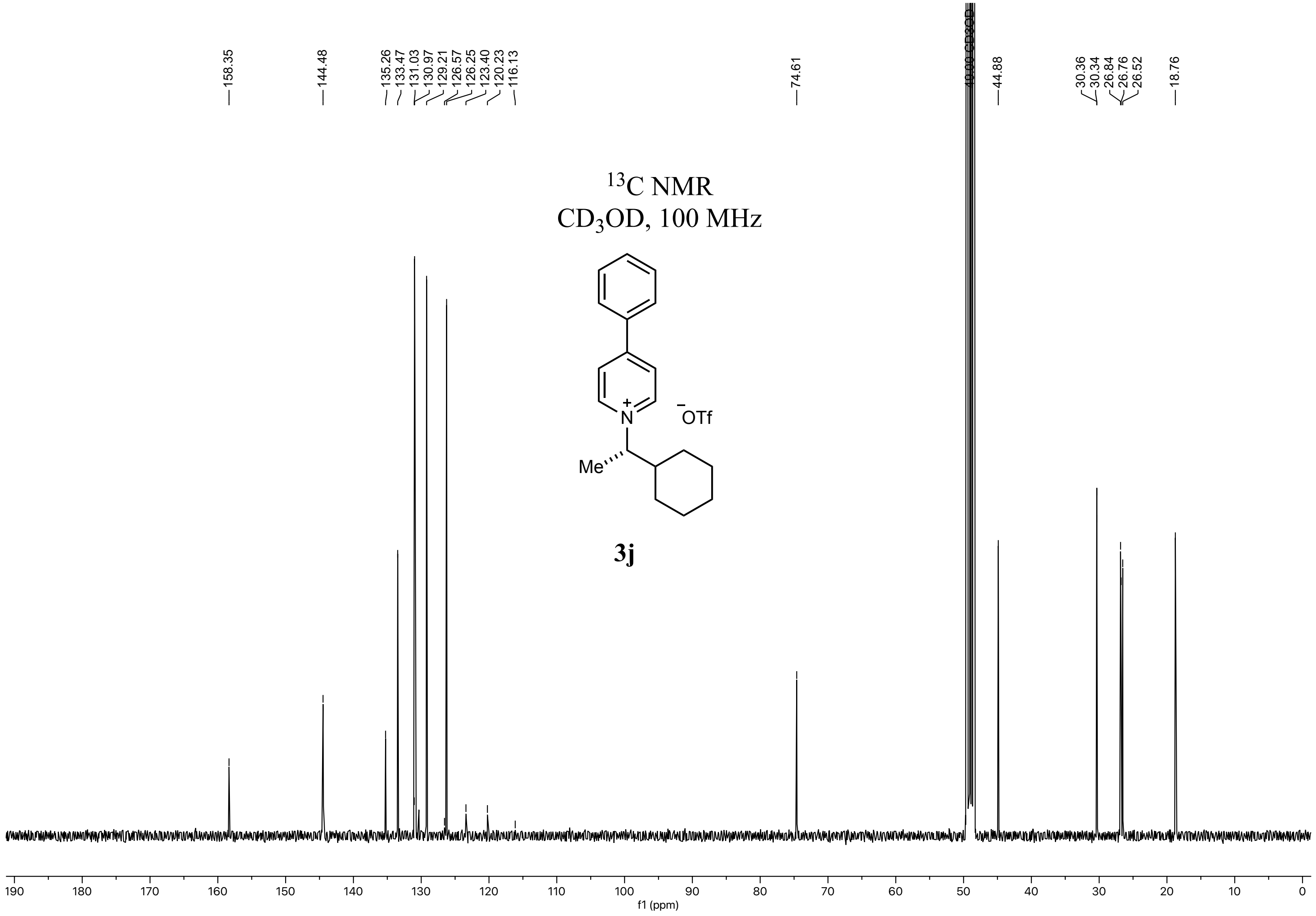

158.35
144.48
135.26
133.47
131.03
130.97
129.21
126.57
126.25
123.40
120.23
116.13
74.61
44.88
30.36
30.34
26.84
26.76
26.52
18.76
^{13}C NMR
CD_3OD, 100 MHz
OTf
N
Me
3j
190
180
170
160
150
140
130
120
110
100
90
80
70
60
50
40
30
20
10
0
f1 (ppm)

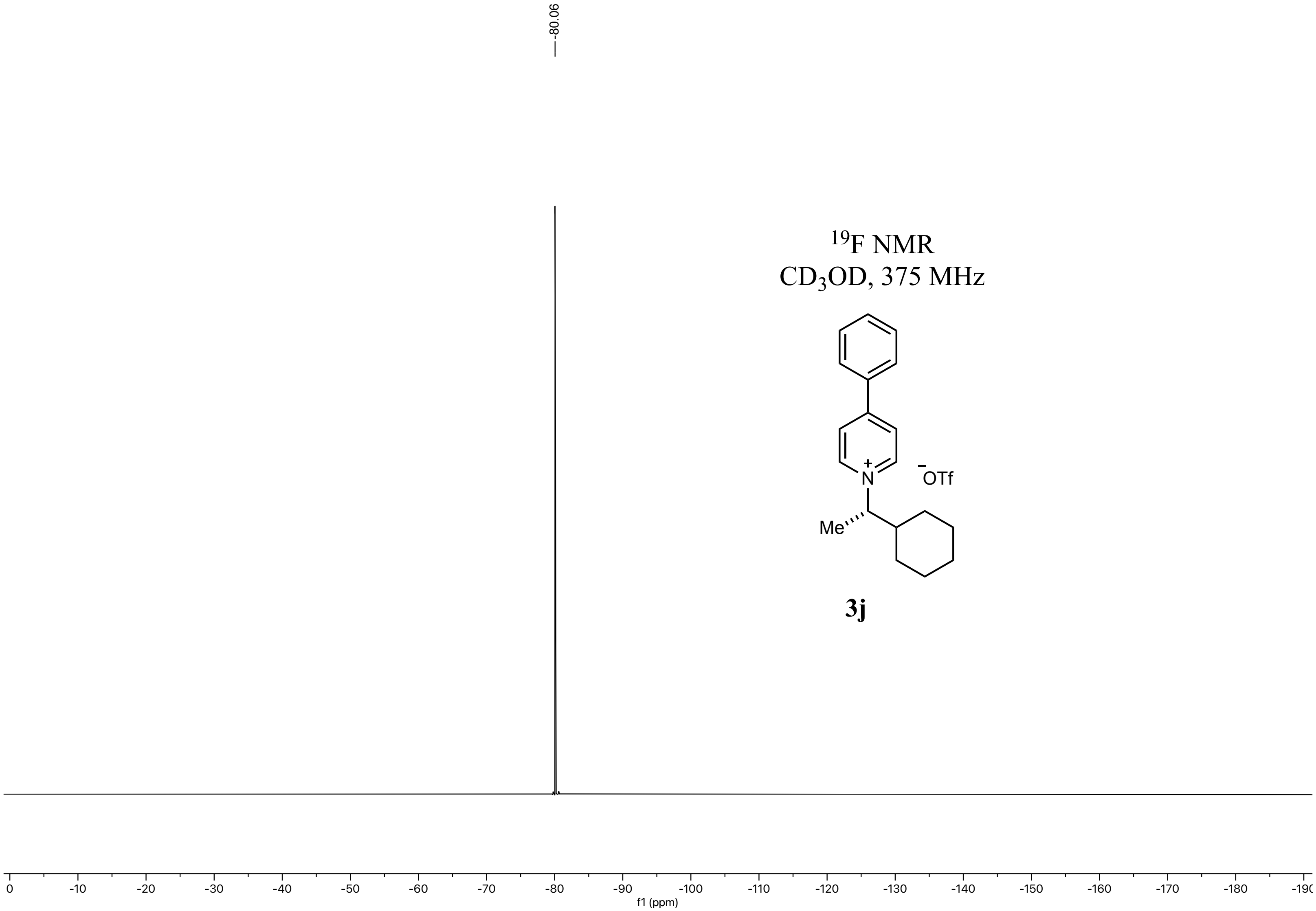

^{19}F NMR
CD_3OD, 375 MHz

3j

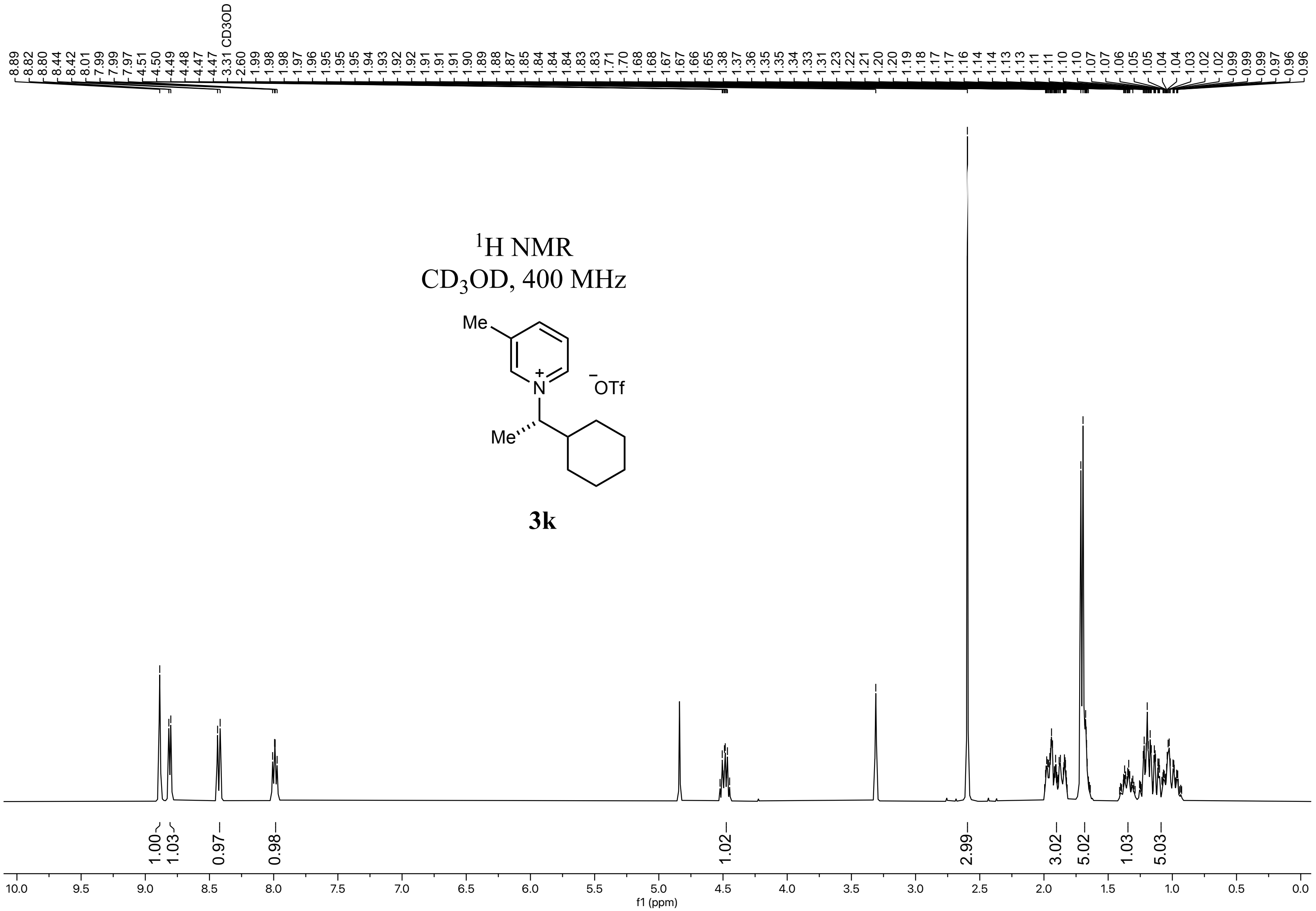
8.89 8.82 8.80 8.44 8.42 8.01 7.99 7.99 7.97 4.51 4.50 4.49 4.48 4.47 3.31 CD3OD 2.60 1.99 1.98 1.98 1.97 1.96 1.95 1.95 1.95 1.94 1.93 1.92 1.92 1.91 1.91 1.91 1.90 1.89 1.88 1.87 1.85 1.84 1.84 1.84 1.83 1.83 1.71 1.70 1.68 1.67 1.67 1.66 1.65 1.38 1.37 1.36 1.35 1.35 1.34 1.33 1.31 1.23 1.22 1.21 1.20 1.19 1.18 1.17 1.17 1.16 1.14 1.14 1.13 1.13 1.11 1.11 1.10 1.07 1.07 1.06 1.05 1.05 1.04 1.04 1.03 1.02 1.02 0.99 0.99 0.97 0.96 0.96
1H NMR
CD3OD, 400 MHz
Me
N
OTf
Me
3k
1.00 1.03 0.97 0.98 1.02 2.99 3.02 5.02 1.03 5.03
10.0 9.5 9.0 8.5 8.0 7.5 7.0 6.5 6.0 5.5 5.0 4.5 4.0 3.5 3.0 2.5 2.0 1.5 1.0 0.5 0.0
f1 (ppm)

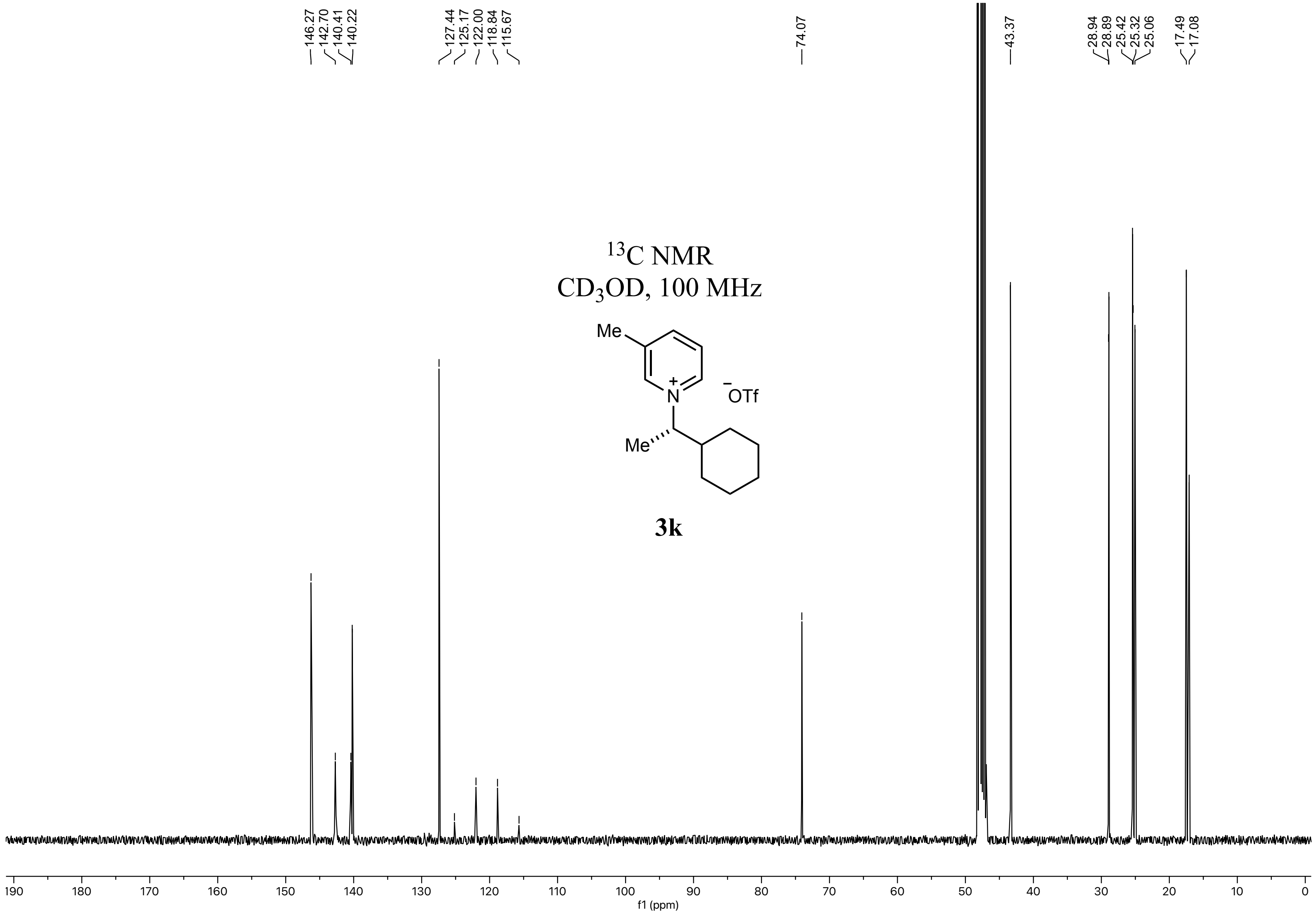

146.27
142.70
140.41
140.22
127.44
125.17
122.00
118.84
115.67
74.07
43.37
28.94
28.89
25.42
25.32
25.06
17.49
17.08
^{13}C NMR
CD_3OD, 100 MHz
Me
N
OTf
Me
3k
190 180 170 160 150 140 130 120 110 100 90 80 70 60 50 40 30 20 10 0
f1 (ppm)

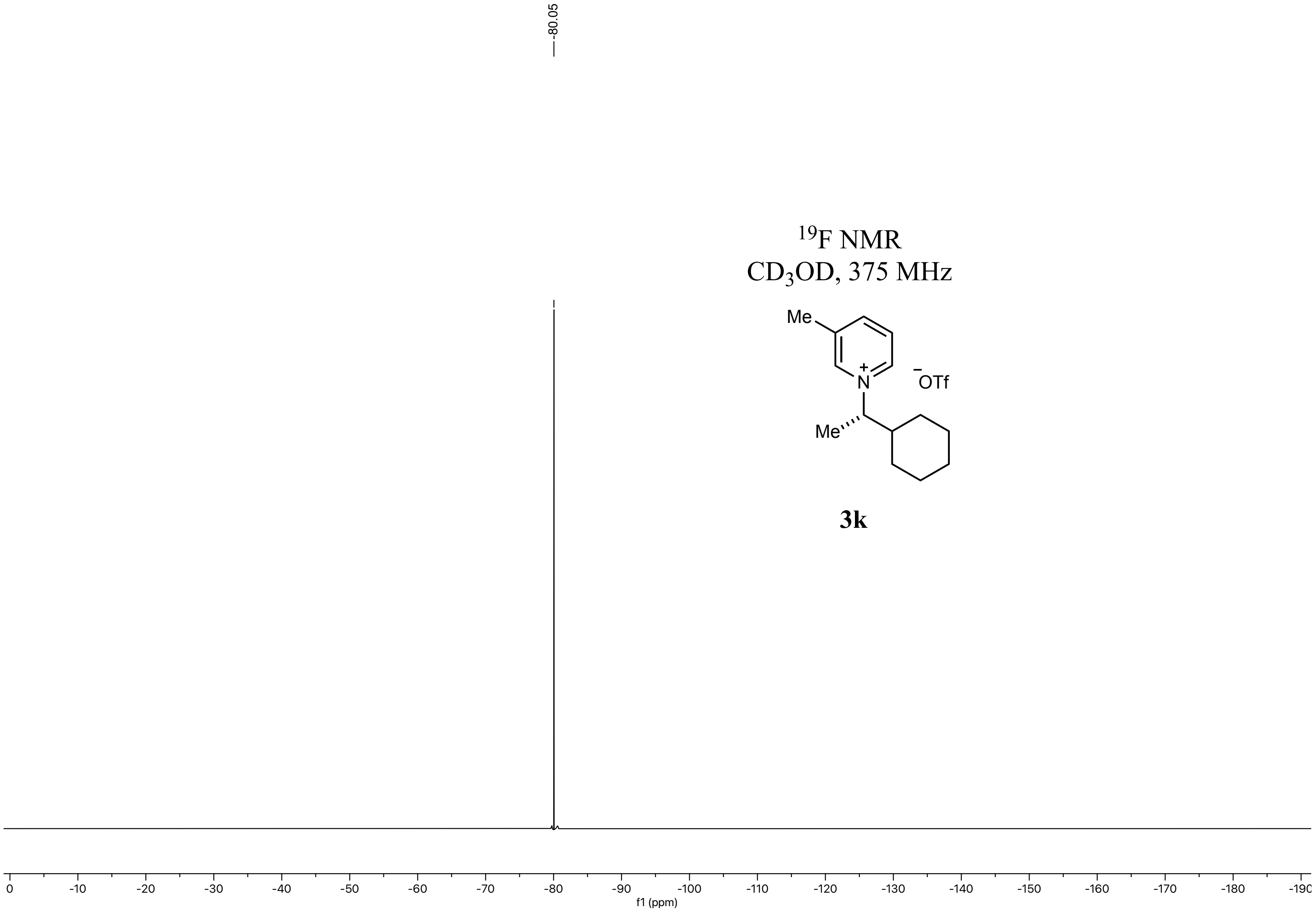

^{19}F NMR
CD_3OD, 375 MHz

3k

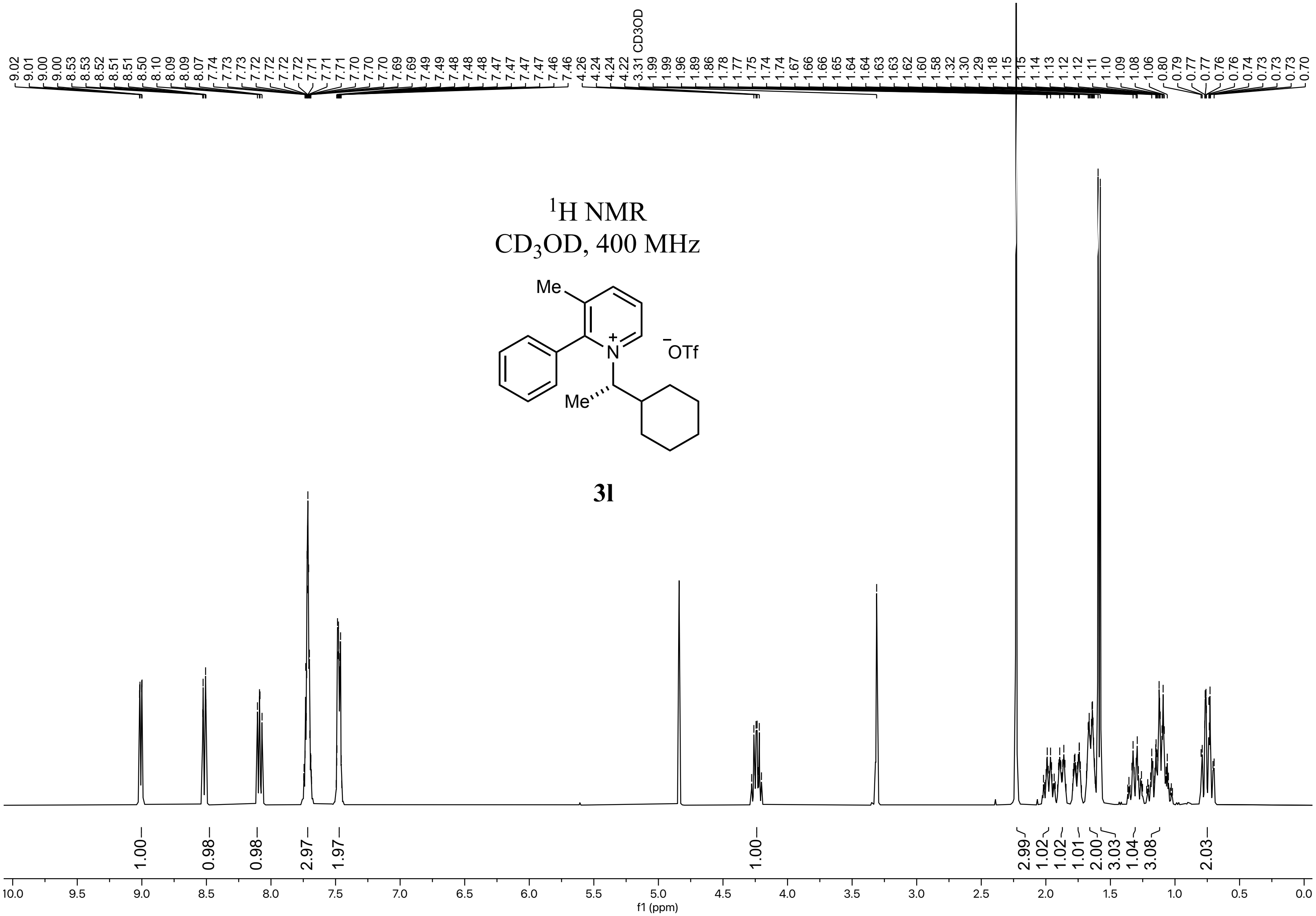
9.02 9.01 9.00 8.53 8.52 8.51 8.51 8.50 8.10 8.09 8.09 8.07 7.74 7.73 7.73 7.72 7.72 7.72 7.71 7.71 7.71 7.70 7.70 7.70 7.69 7.69 7.49 7.48 7.48 7.47 7.47 7.47 7.46 4.26 4.24 4.24 4.22 3.31 CD3OD 1.99 1.99 1.96 1.89 1.86 1.78 1.77 1.75 1.74 1.74 1.67 1.66 1.66 1.65 1.64 1.64 1.63 1.63 1.62 1.60 1.58 1.32 1.30 1.29 1.18 1.15 1.15 1.14 1.13 1.12 1.12 1.11 1.10 1.09 1.08 1.06 0.80 0.79 0.77 0.77 0.76 0.74 0.73 0.73 0.73 0.70
1H NMR
CD3OD, 400 MHz
Me
OTf
Me
3l
1.00 0.98 0.98 2.97 1.97 1.00 2.99 1.02 1.02 1.01 2.00 3.03 1.04 3.08 2.03
10.0 9.5 9.0 8.5 8.0 7.5 7.0 6.5 6.0 5.5 5.0 4.5 4.0 3.5 3.0 2.5 2.0 1.5 1.0 0.5 0.0
f1 (ppm)

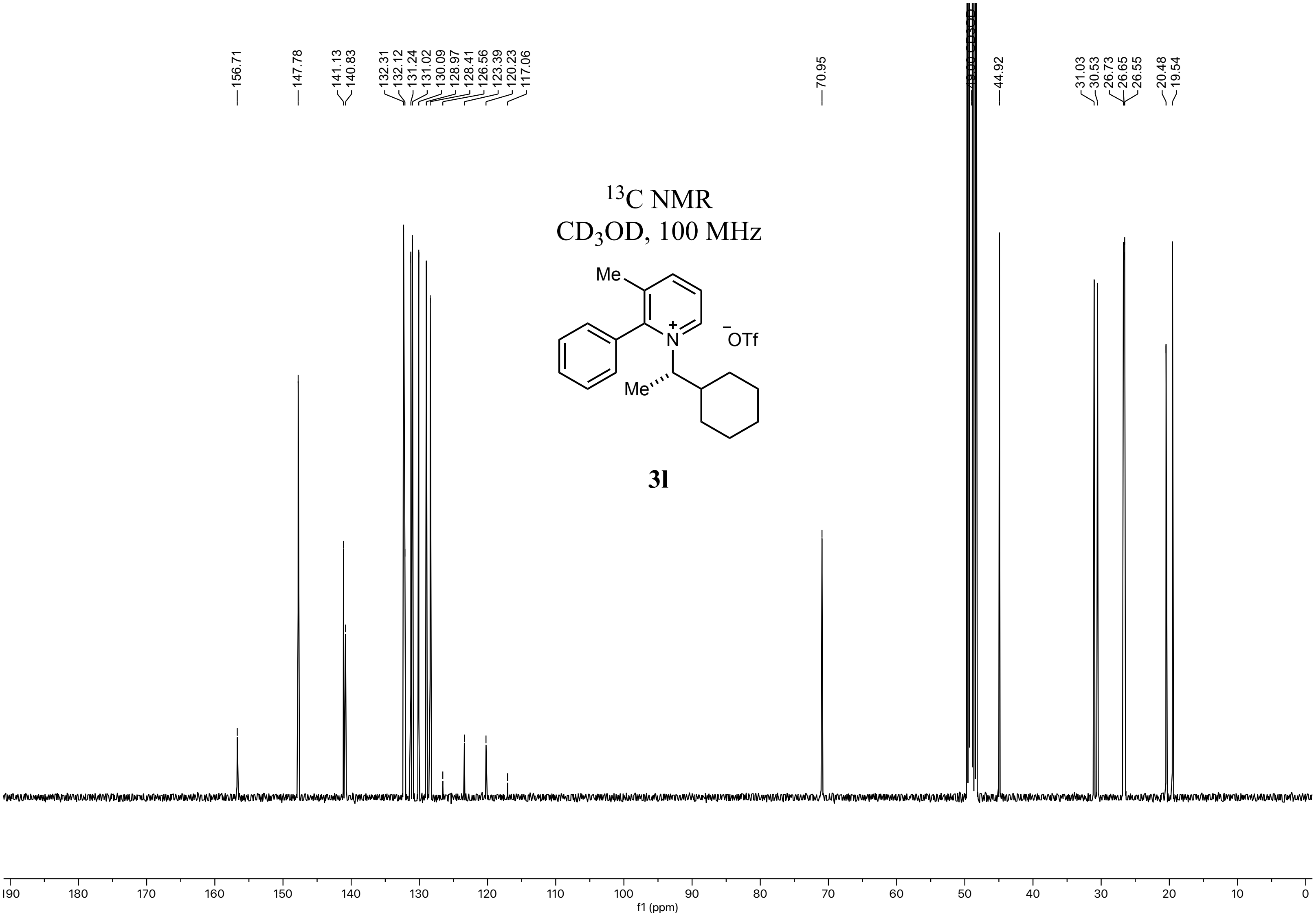
156.71
147.78
141.13
140.83
132.31
132.12
131.24
131.02
130.09
128.97
128.41
126.56
123.39
120.23
117.06
70.95
44.92
31.03
30.53
26.73
26.65
26.55
20.48
19.54
^{13}C NMR
CD_3OD, 100 MHz
Me
N
OTf
Me
3l
f1 (ppm)

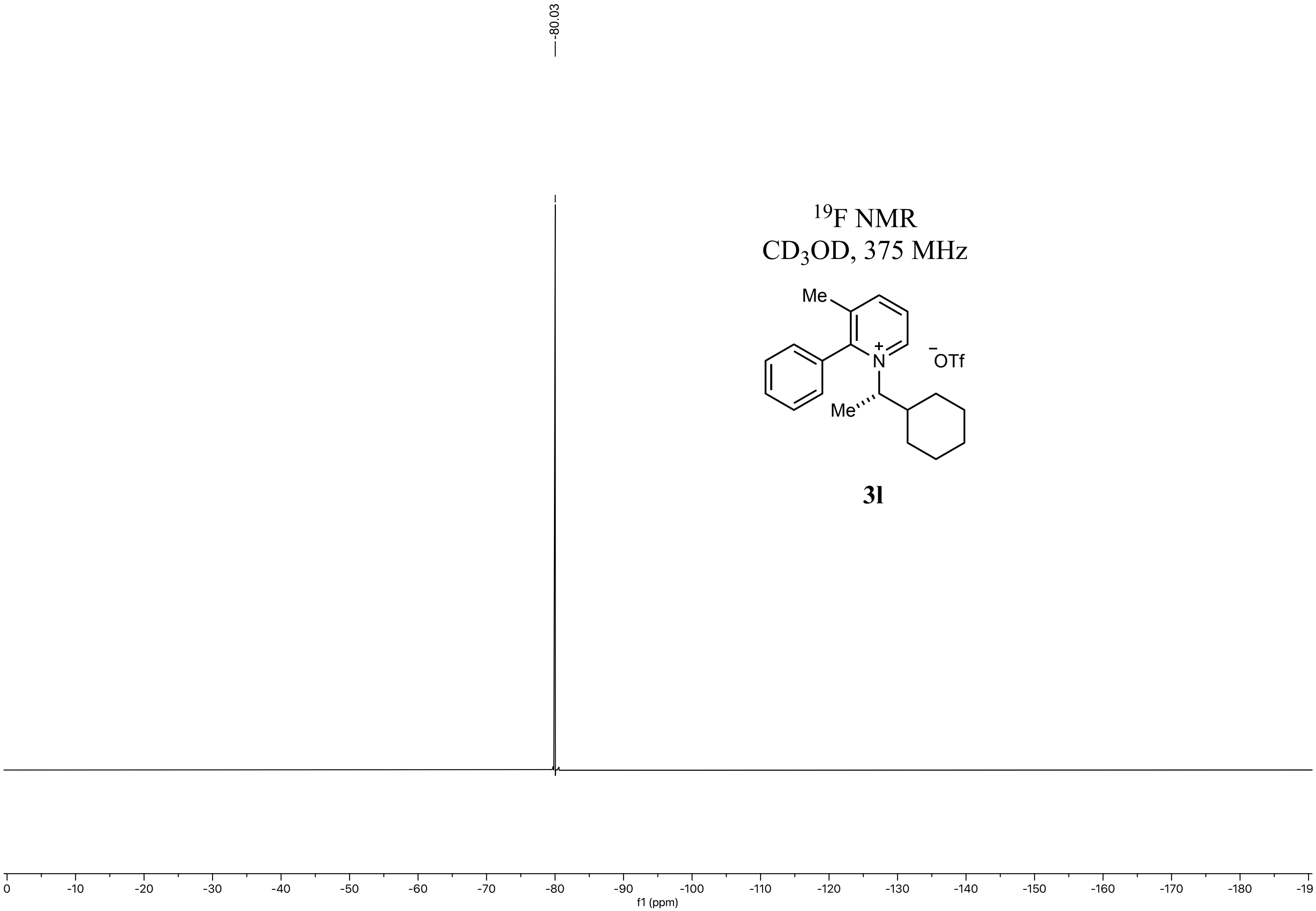

^{19}F NMR
CD_3OD, 375 MHz

3l

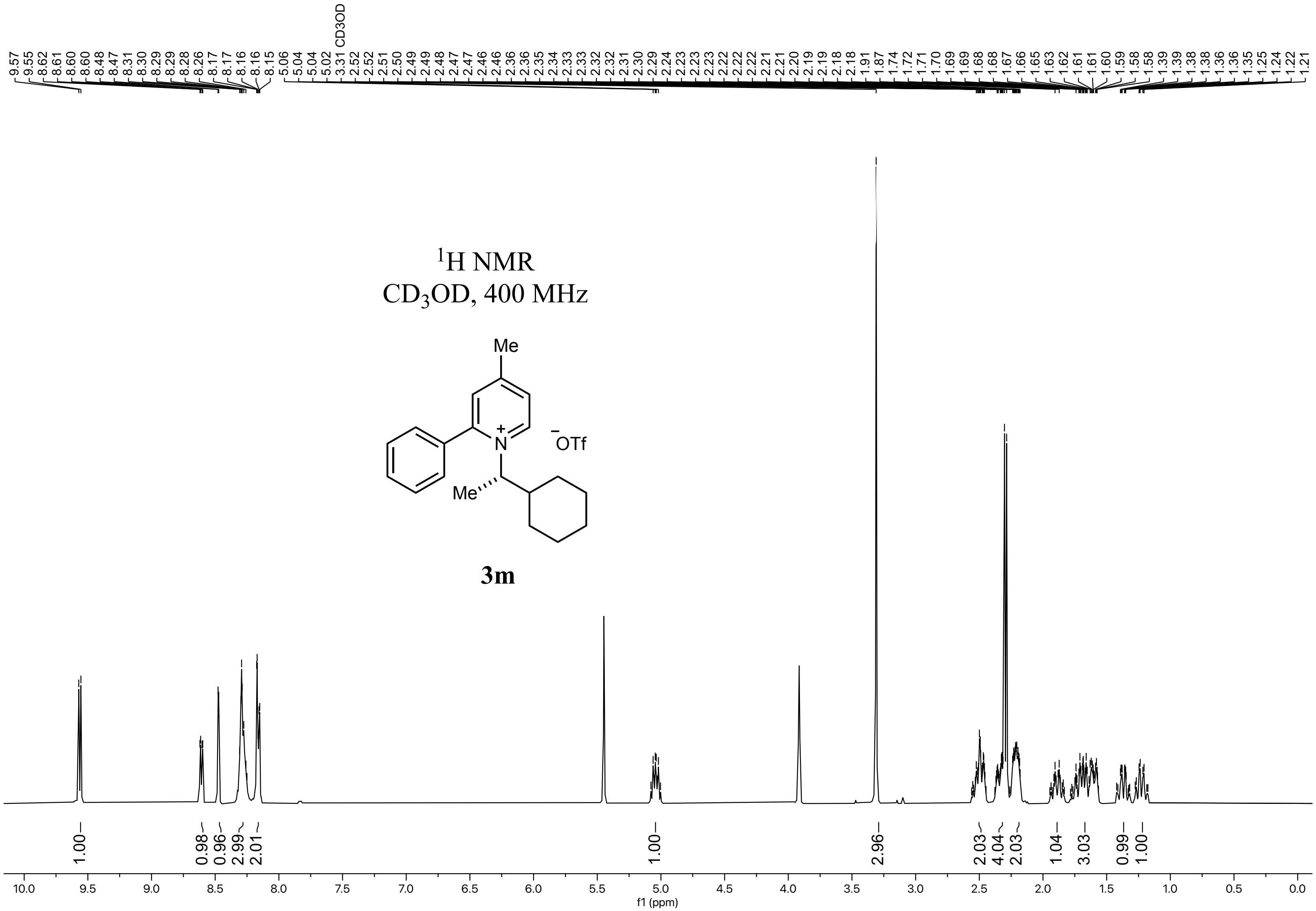

1H NMR
CD3OD, 400 MHz
Me
N
OTf
Me
3m
CD3OD
f1 (ppm)

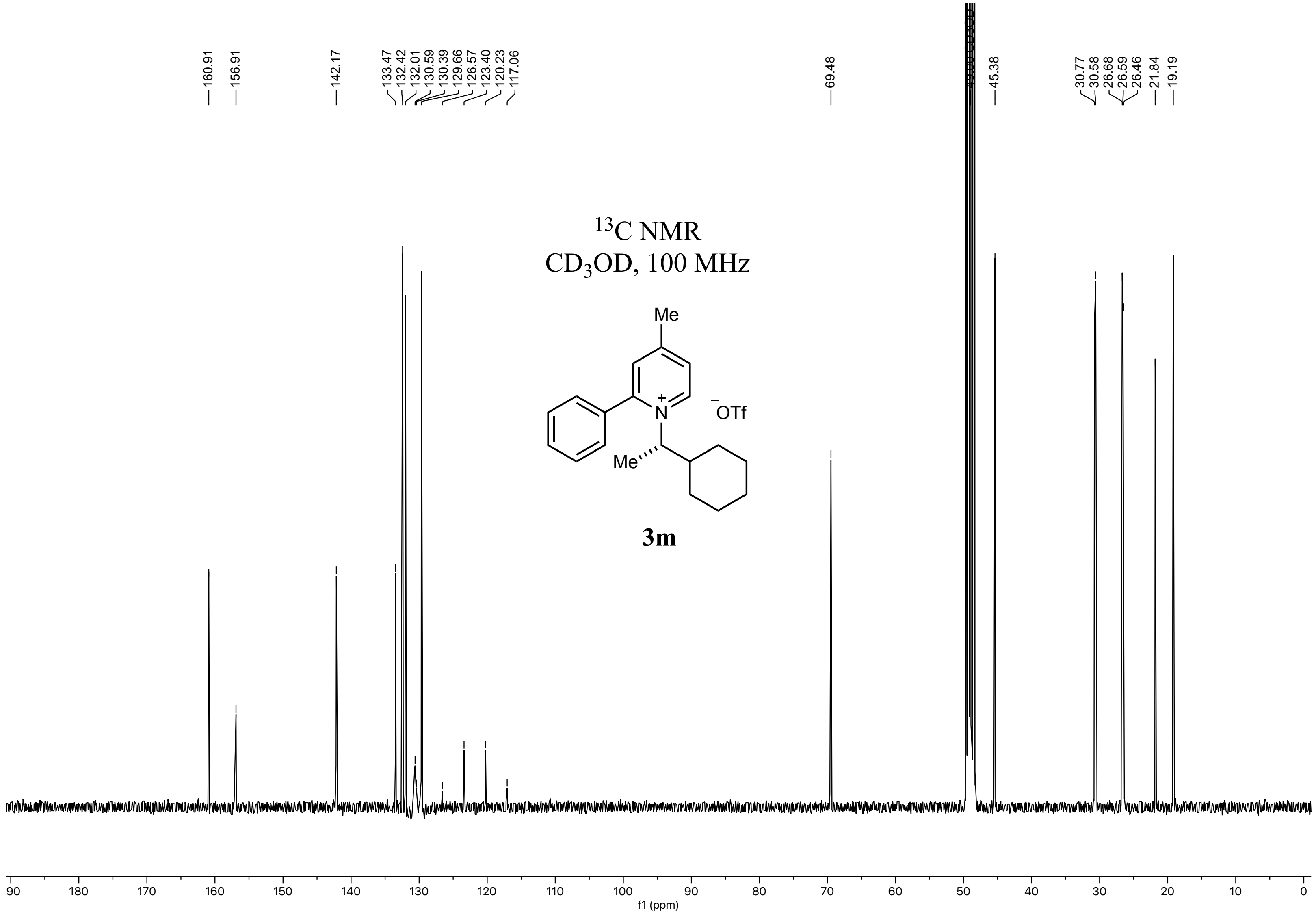
160.91
156.91
142.17
133.47
132.42
132.01
130.59
130.39
129.66
126.57
123.40
120.23
117.06
69.48
45.38
30.77
30.58
26.68
26.59
26.46
21.84
19.19
13C NMR
CD3OD, 100 MHz
Me
N
+
OTf
Me
f1 (ppm)

3m

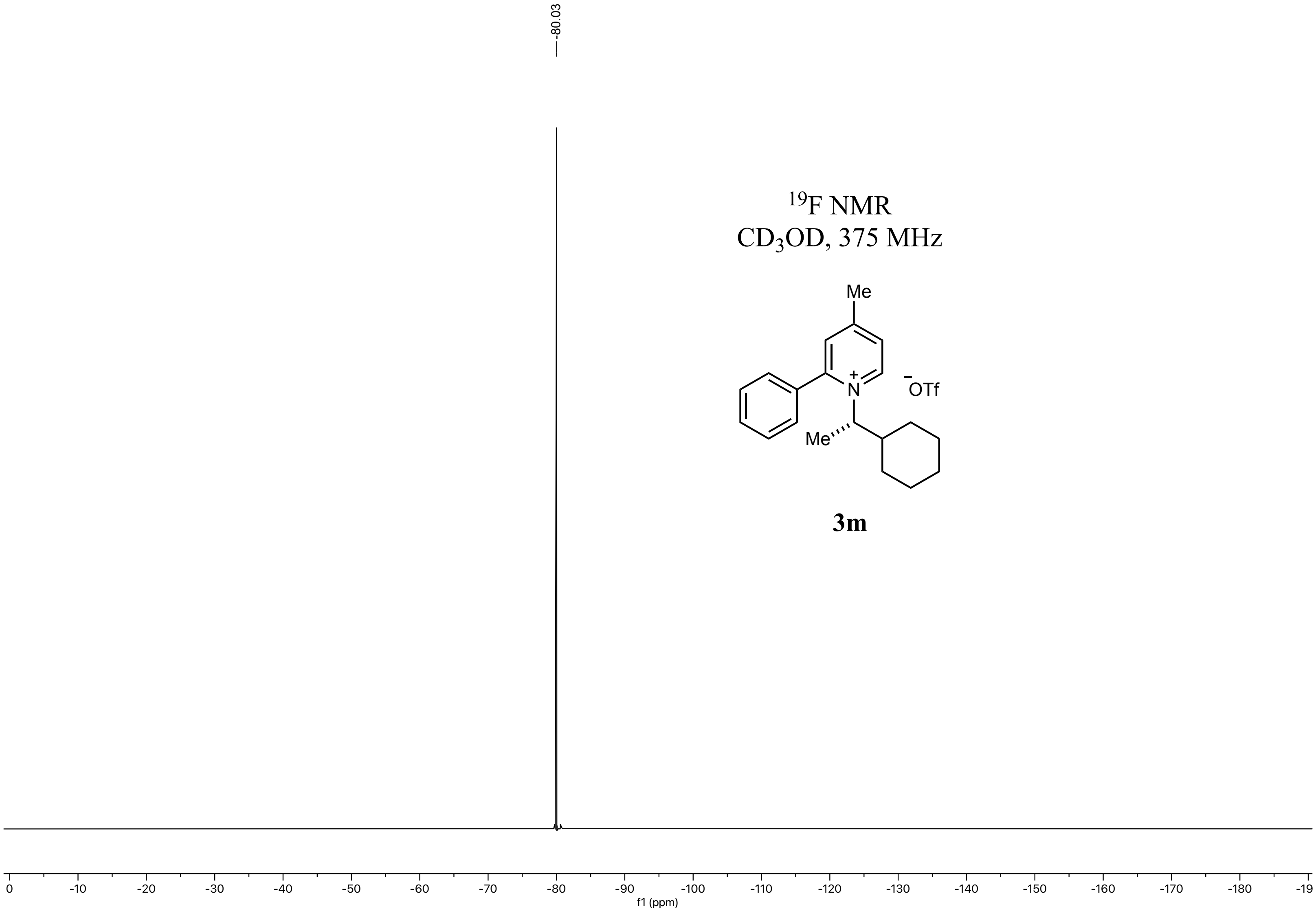

^{19}F NMR
CD_3OD, 375 MHz

3m

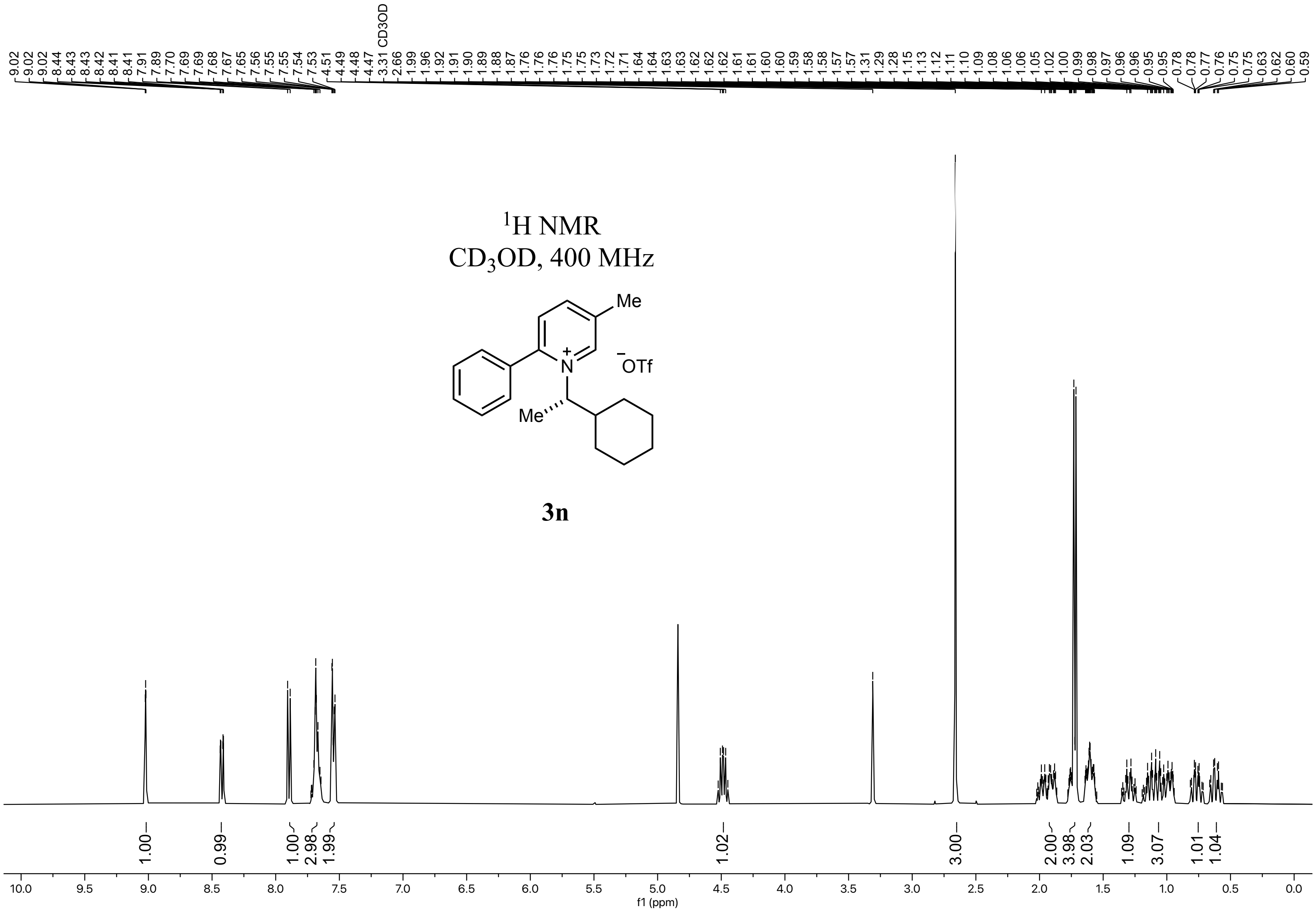

1H NMR
CD3OD, 400 MHz
Me
OTf
Me
3n
f1 (ppm)

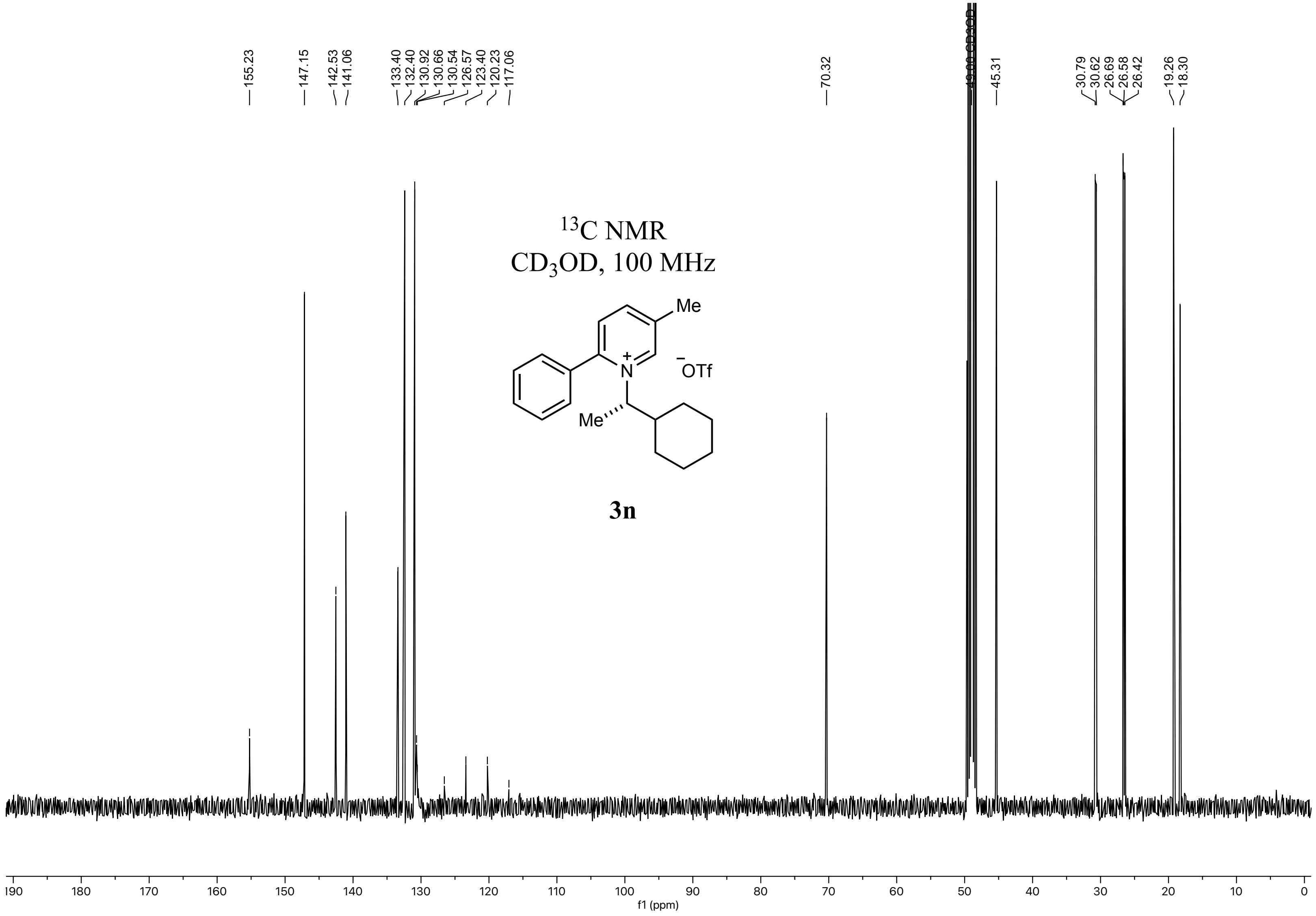

155.23
147.15
142.53
141.06
133.40
132.40
130.92
130.66
130.54
126.57
123.40
120.23
117.06
70.32
45.31
30.79
30.62
26.69
26.58
26.42
19.26
18.30
^{13}C NMR
CD_3OD, 100 MHz
Me
OTf
Me
190
180
170
160
150
140
130
120
110
100
90
80
70
60
50
40
30
20
10
0
f1 (ppm)

3n

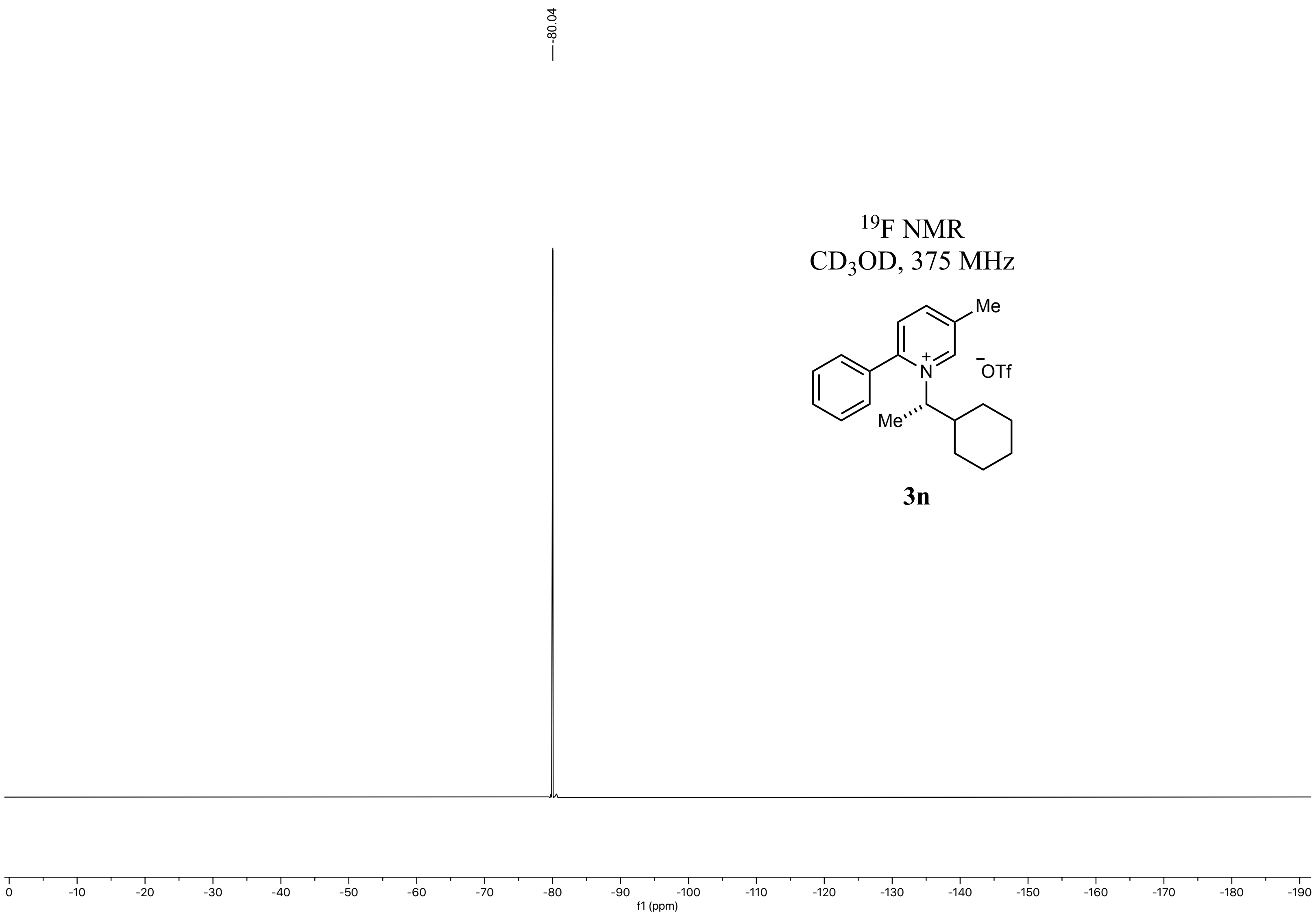
-80.04
19F NMR
CD3OD, 375 MHz
Me
OTf
N
Me
3n
0 -10 -20 -30 -40 -50 -60 -70 -80 -90 -100 -110 -120 -130 -140 -150 -160 -170 -180 -190
f1 (ppm)

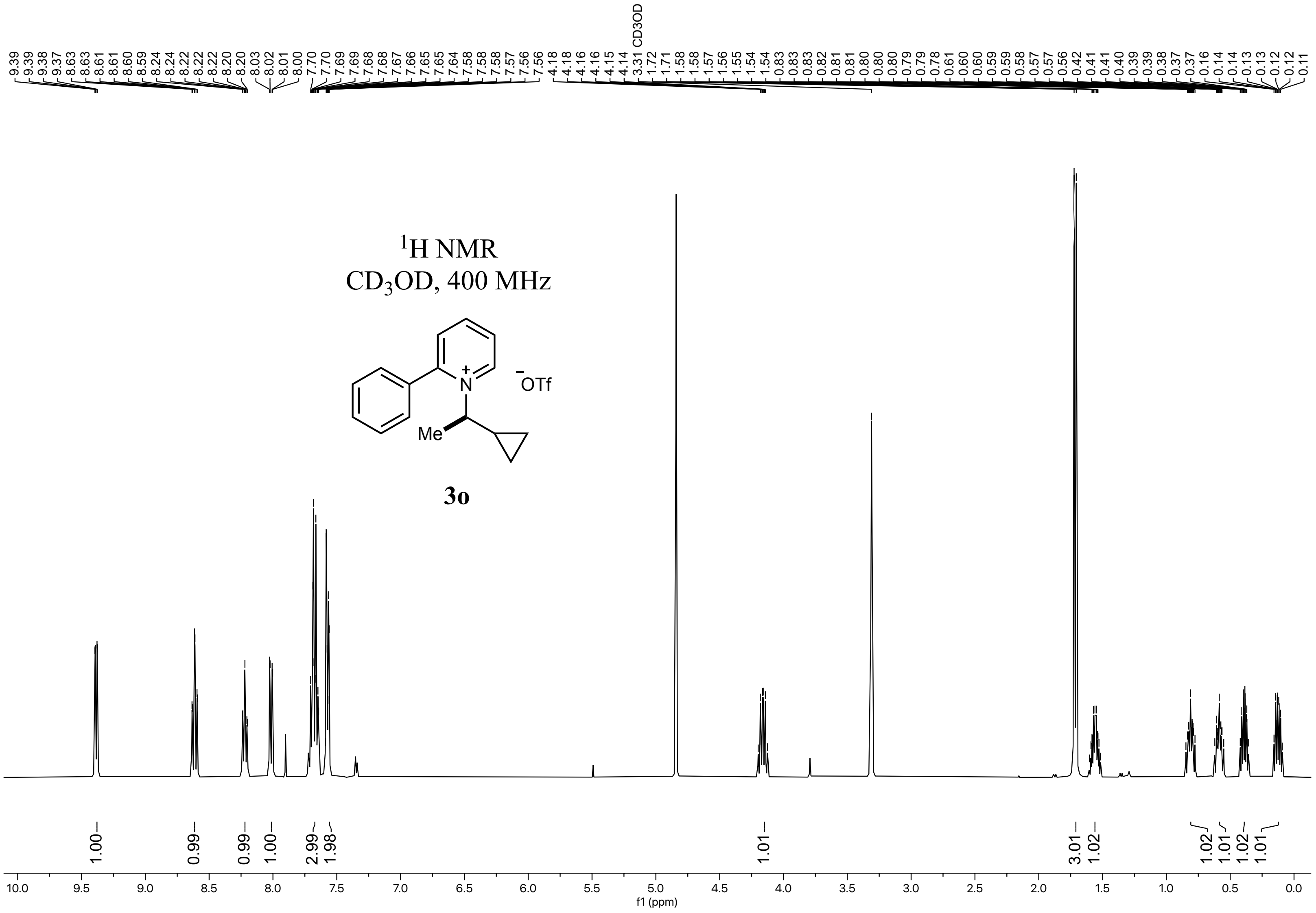
9.39 9.39 9.38 9.37 8.63 8.61 8.61 8.60 8.59 8.24 8.22 8.22 8.22 8.20 8.20 8.03 8.02 8.01 8.00 7.70 7.70 7.69 7.69 7.68 7.67 7.66 7.65 7.64 7.58 7.58 7.58 7.57 7.56 7.56 4.18 4.18 4.16 4.16 4.15 4.14 3.31 CD3OD 1.72 1.71 1.58 1.58 1.57 1.56 1.55 1.54 1.54 0.83 0.83 0.83 0.82 0.81 0.81 0.80 0.80 0.80 0.79 0.79 0.78 0.61 0.60 0.60 0.59 0.59 0.58 0.57 0.57 0.56 0.42 0.41 0.41 0.40 0.39 0.39 0.38 0.37 0.37 0.16 0.14 0.14 0.13 0.13 0.12 0.12 0.11
^{1}H NMR
CD_3OD, 400 MHz
N
+
$^-$OTf
Me
3o
1.00 0.99 0.99 1.00 2.99 1.98 1.01 3.01 1.02 1.02 1.01 1.02 1.01
10.0 9.5 9.0 8.5 8.0 7.5 7.0 6.5 6.0 5.5 5.0 4.5 4.0 3.5 3.0 2.5 2.0 1.5 1.0 0.5 0.0
f1 (ppm)

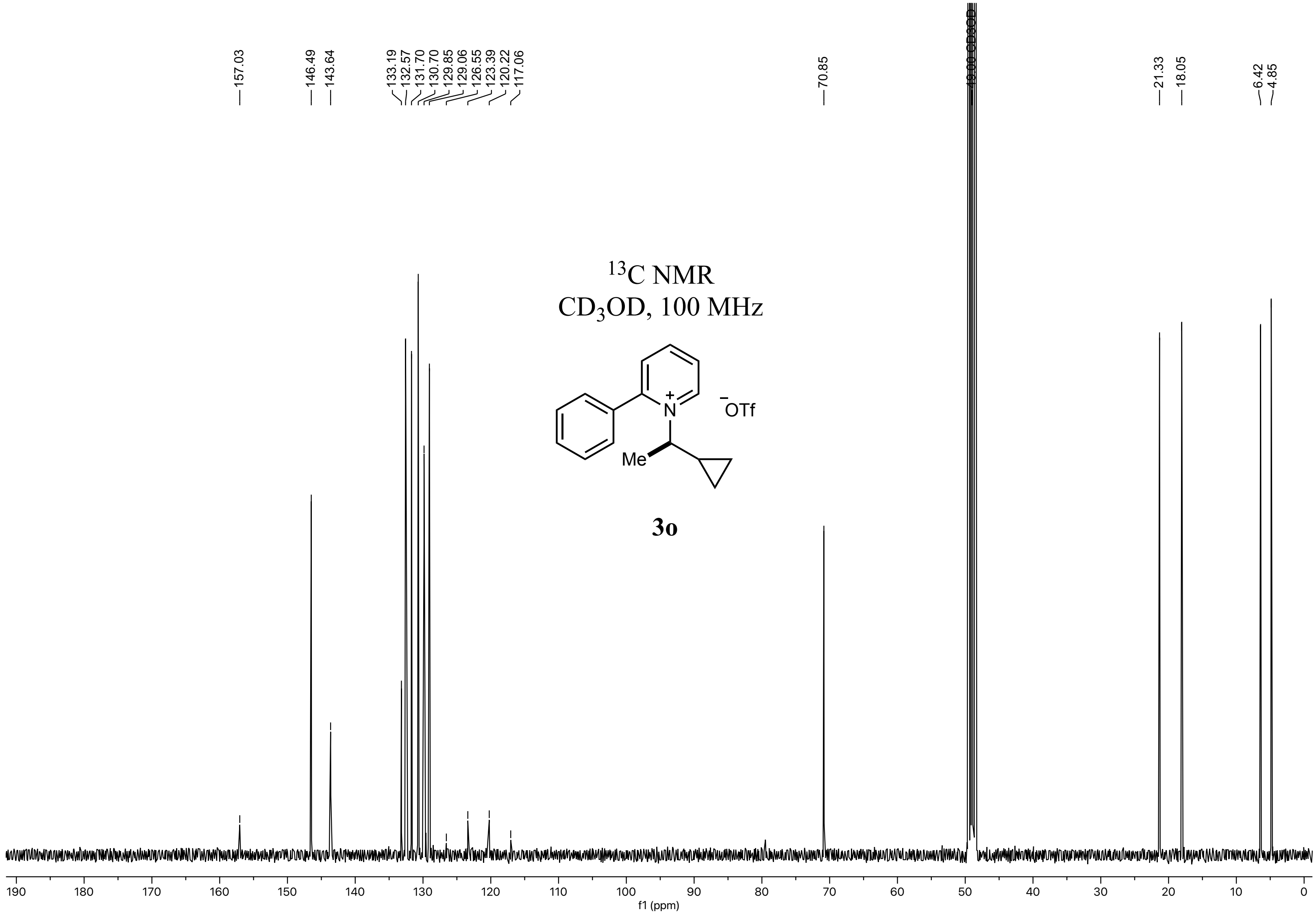
157.03
146.49
143.64
133.19
132.57
131.70
130.70
129.85
129.06
126.55
123.39
120.22
117.06
70.85
49.00 CD3OD
21.33
18.05
6.42
4.85
13C NMR
CD3OD, 100 MHz
OTf
Me
3o
190
180
170
160
150
140
130
120
110
100
90
80
70
60
50
40
30
20
10
0
f1 (ppm)

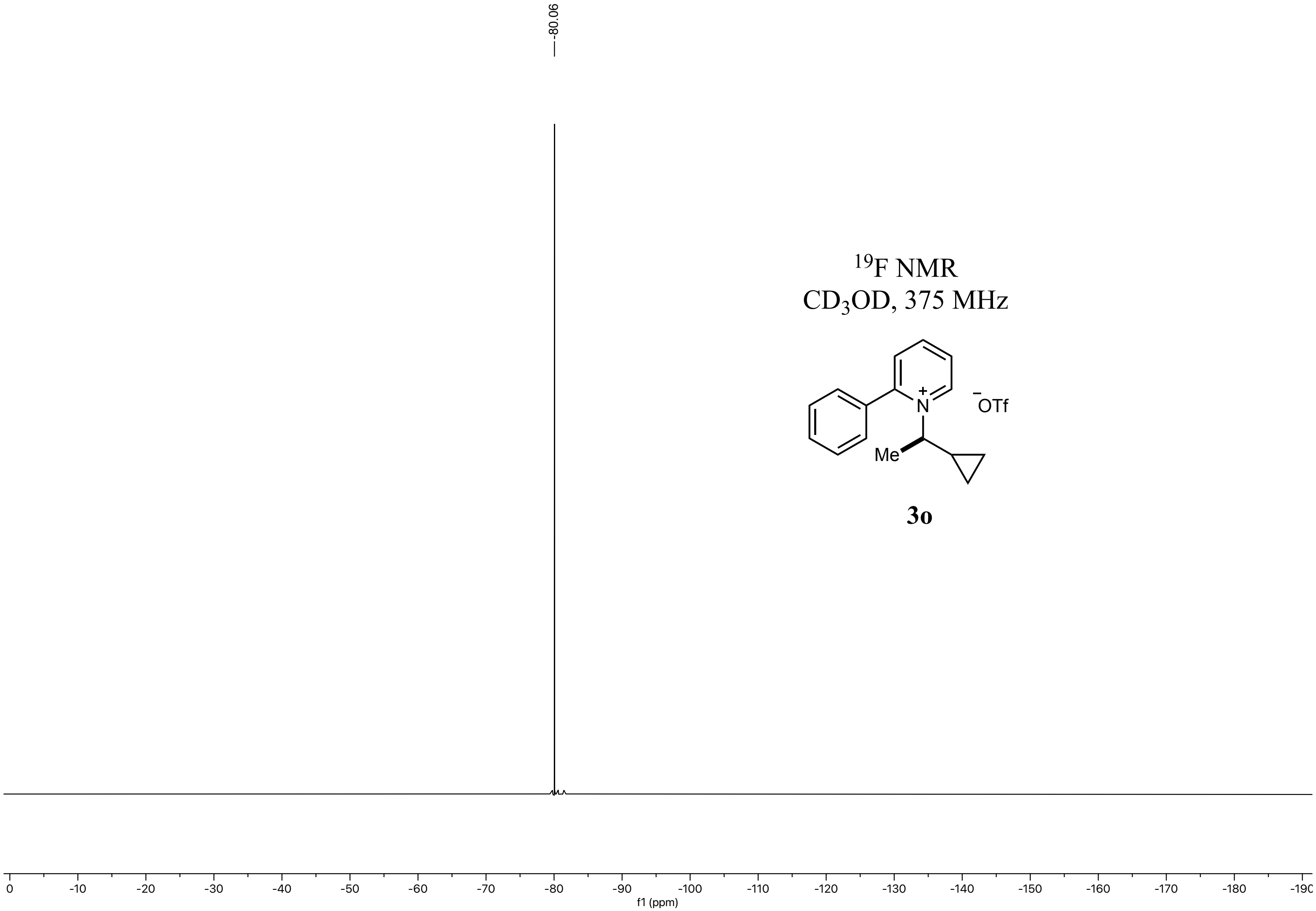

-80.06
19F NMR
CD3OD, 375 MHz
OTf
N
Me
0
-10
-20
-30
-40
-50
-60
-70
-80
-90
-100
-110
-120
-130
-140
-150
-160
-170
-180
-190
f1 (ppm)

3o

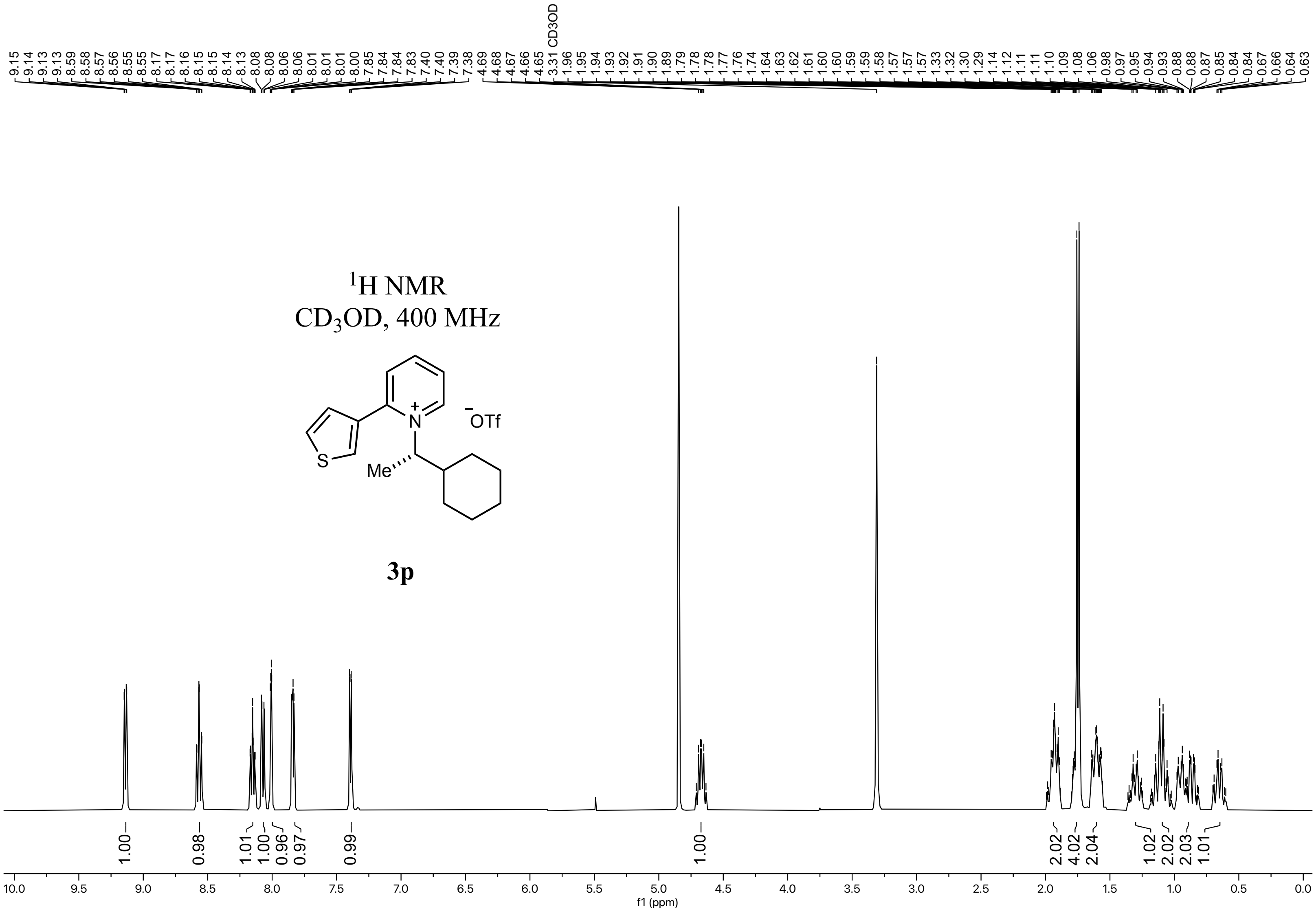
CD3OD
1H NMR
CD3OD, 400 MHz
S
N
Me
OTf
3p
f1 (ppm)

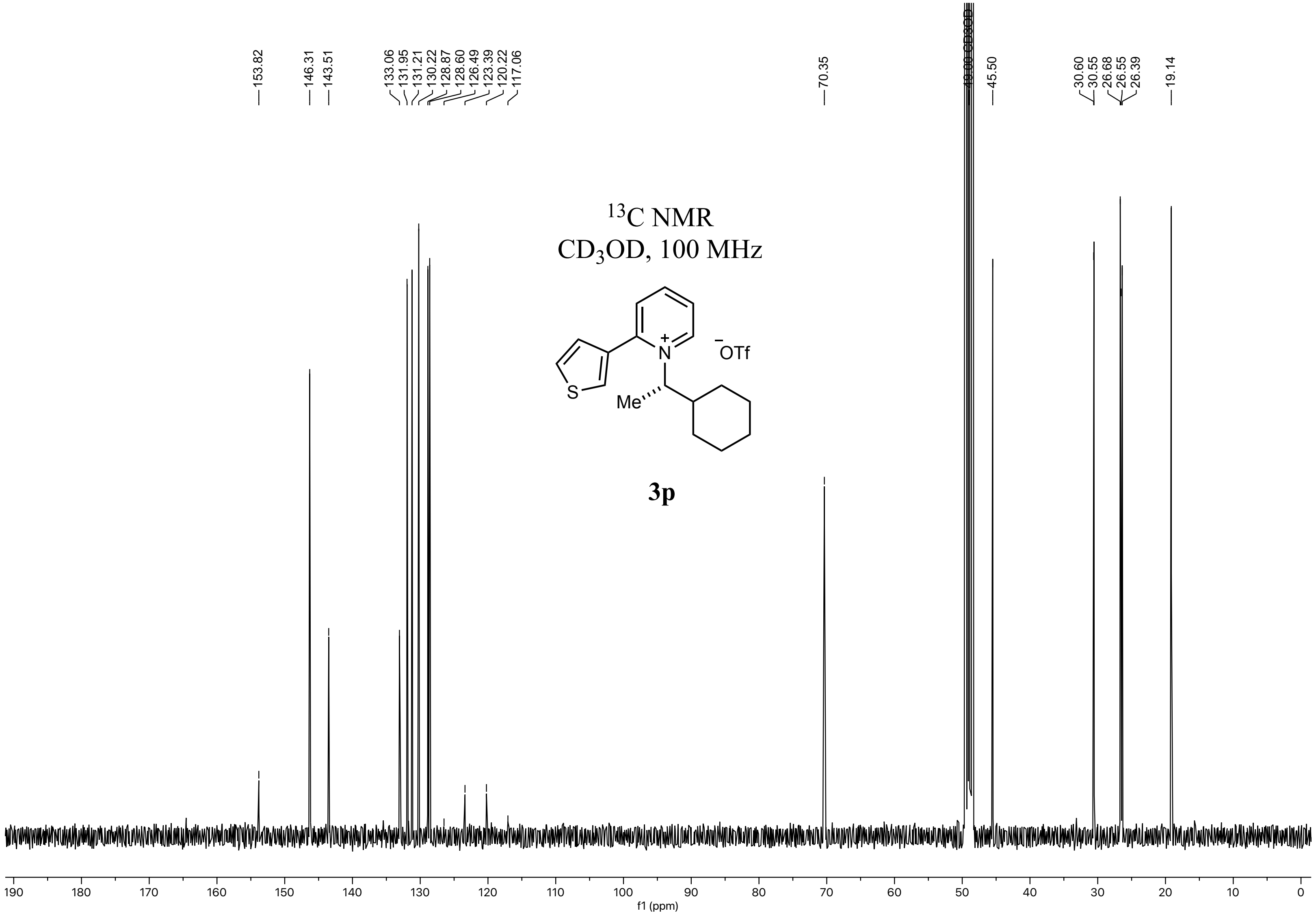
153.82
146.31
143.51
133.06
131.95
131.21
130.22
128.87
128.60
126.49
123.39
120.22
117.06
70.35
49.00 CD3OD
45.50
30.60
30.55
26.68
26.55
26.39
19.14
13C NMR
CD3OD, 100 MHz
OTf
S
N
Me
3p
190
180
170
160
150
140
130
120
110
100
90
80
70
60
50
40
30
20
10
0
f1 (ppm)

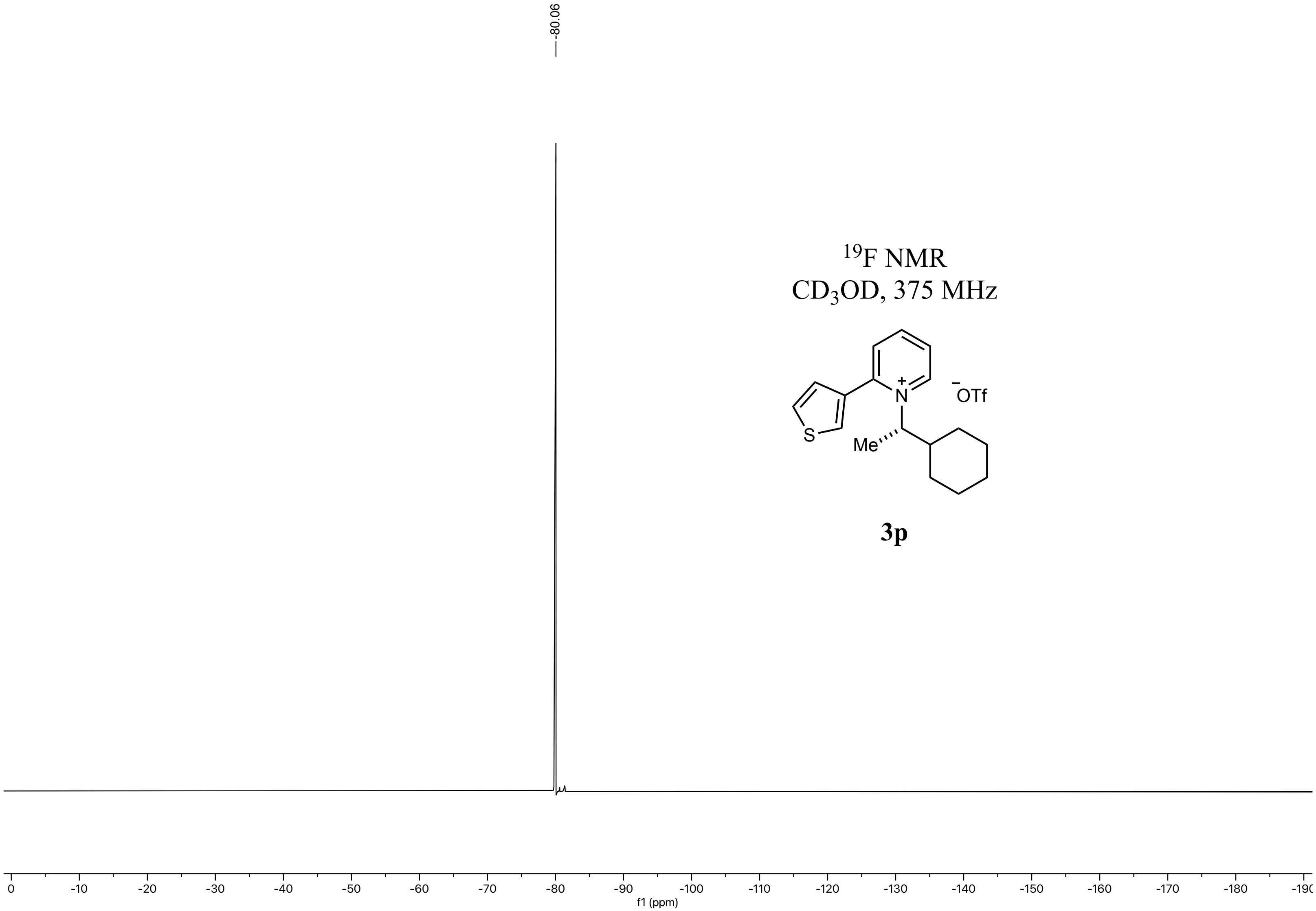
-80.06
^{19}F NMR
CD_3OD, 375 MHz
OTf
N
S
Me
3p
0 -10 -20 -30 -40 -50 -60 -70 -80 -90 -100 -110 -120 -130 -140 -150 -160 -170 -180 -190
f1 (ppm)

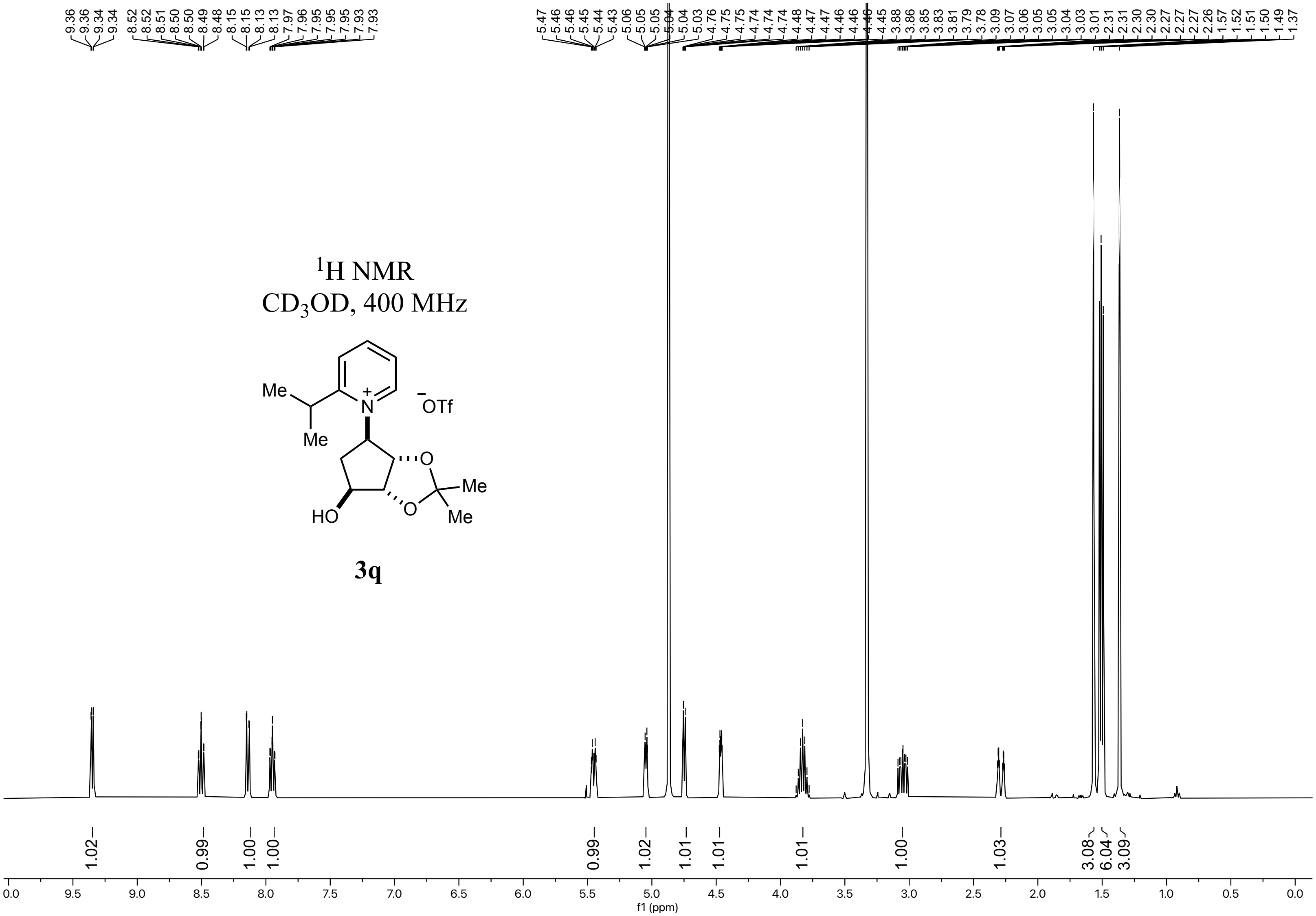

^{1}H NMR
CD_3OD, 400 MHz
3q
f1 (ppm)

^{13}C NMR
CD_3OD, 100 MHz

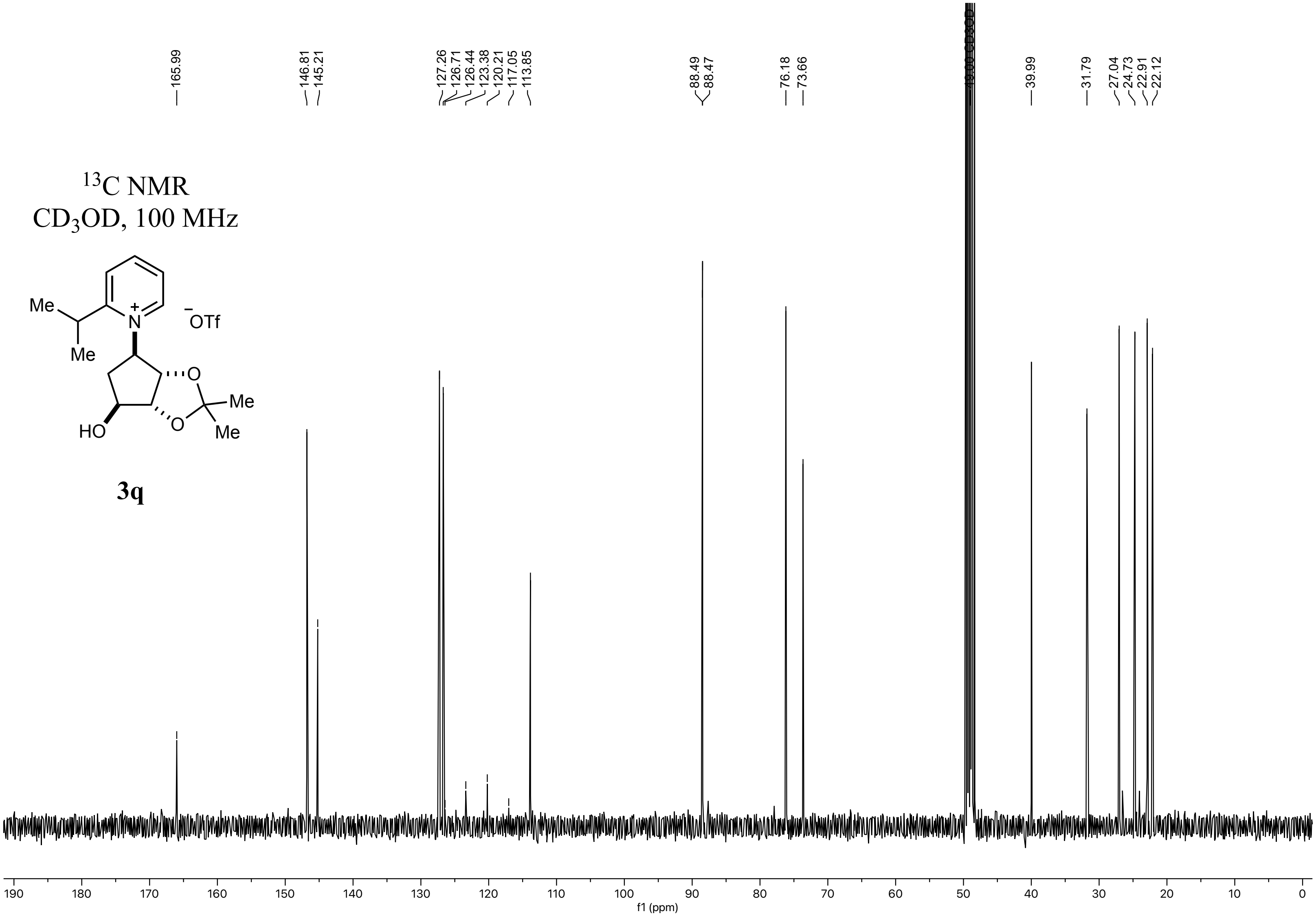

3q

^{19}F NMR
CD_3OD, 375 MHz

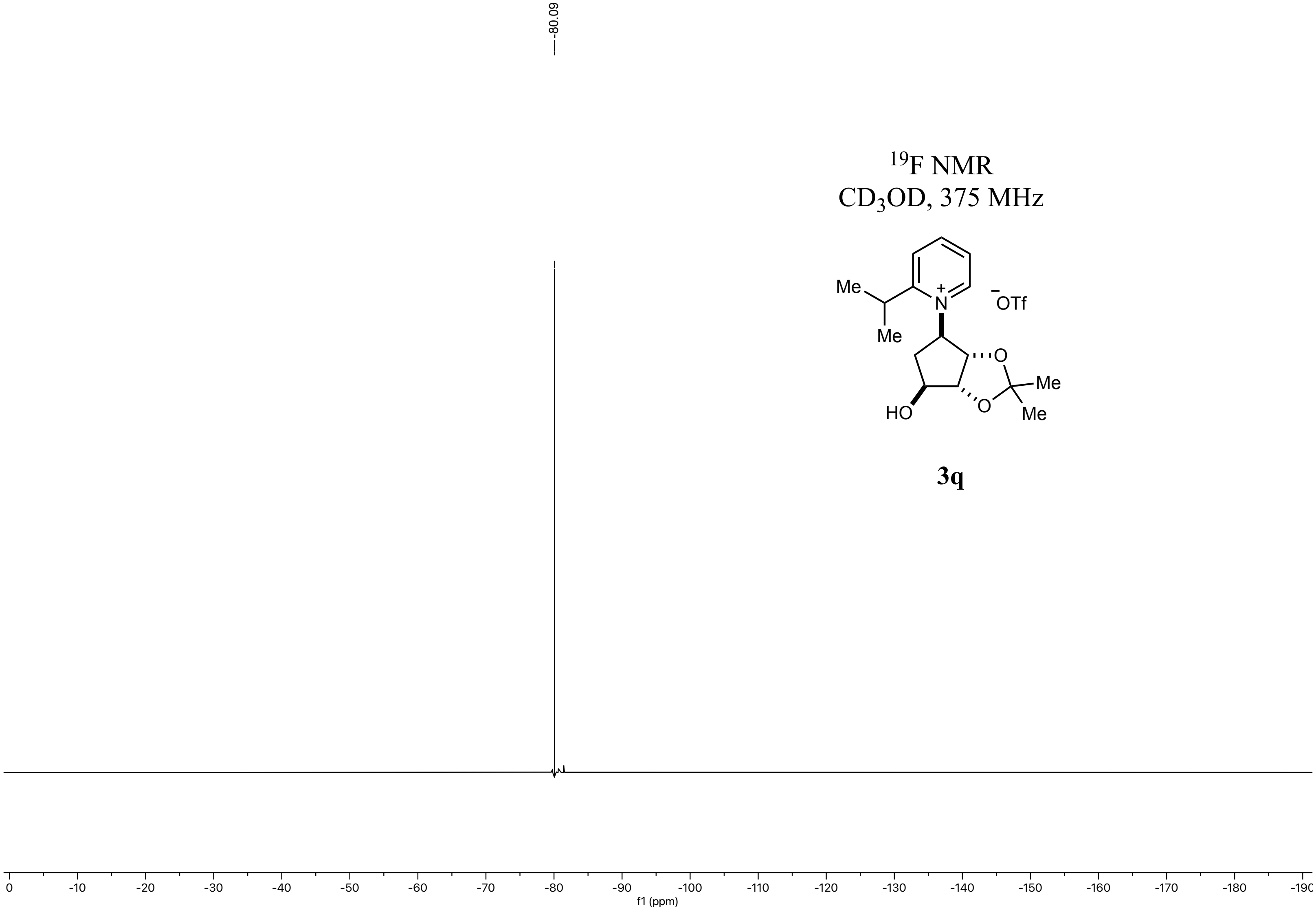

3q

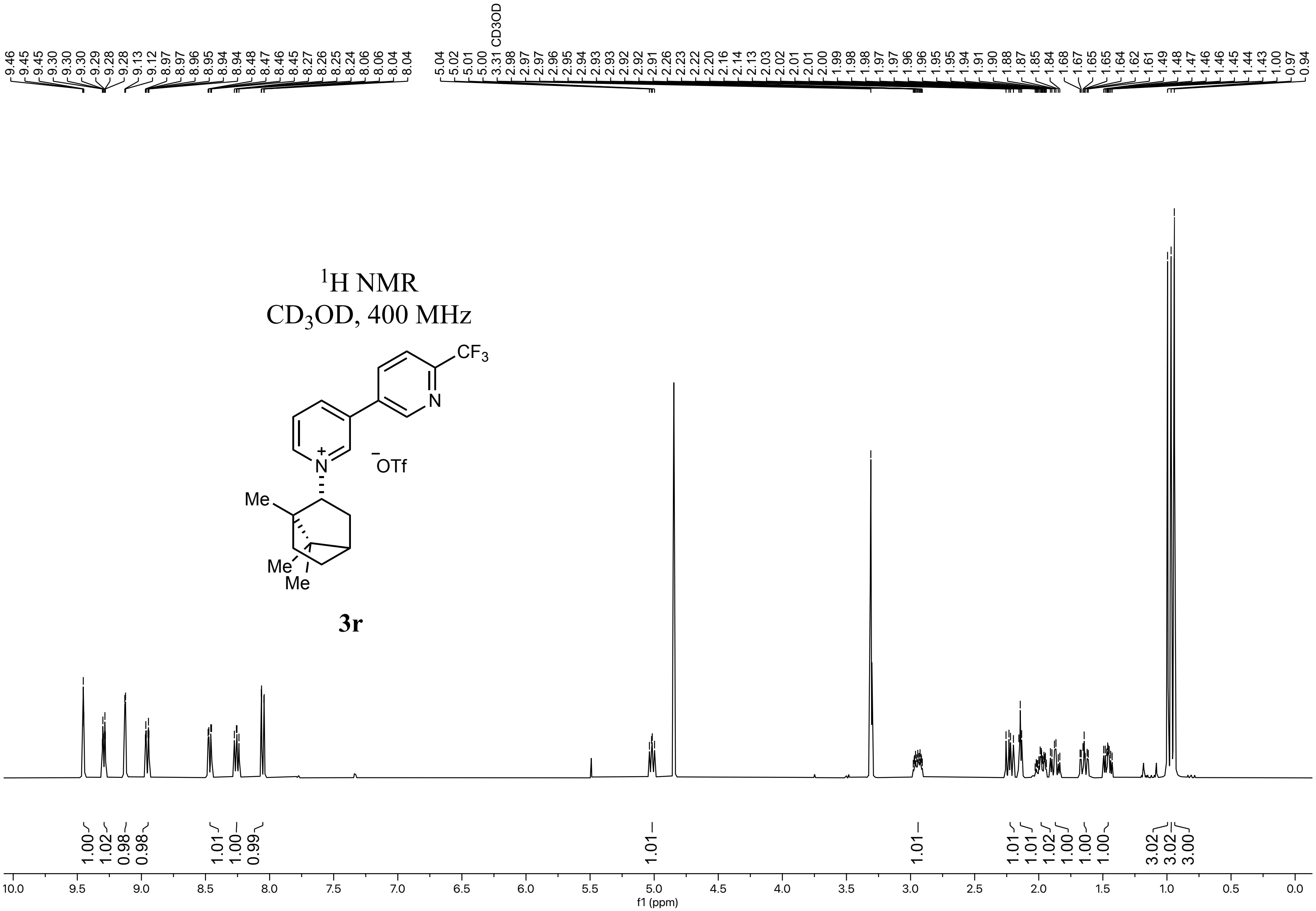
9.46 9.45 9.30 9.30 9.29 9.28 9.13 9.12 8.97 8.97 8.96 8.95 8.94 8.48 8.47 8.46 8.45 8.27 8.26 8.25 8.24 8.06 8.06 8.04 8.04
5.04 5.02 5.01 5.00 3.31 CD3OD 2.98 2.97 2.97 2.96 2.95 2.94 2.93 2.93 2.92 2.92 2.91 2.26 2.23 2.22 2.20 2.16 2.14 2.13 2.03 2.02 2.01 2.01 2.00 1.99 1.98 1.98 1.97 1.97 1.96 1.96 1.95 1.95 1.94 1.91 1.90 1.88 1.87 1.85 1.84 1.68 1.67 1.65 1.65 1.64 1.62 1.61 1.49 1.48 1.47 1.46 1.46 1.45 1.44 1.43 1.00 0.97 0.94
^{1}H NMR
CD$_3$OD, 400 MHz
CF$_3$
N
N
+
–OTf
Me
Me
Me
3r
1.00 1.02 0.98 0.98 1.01 1.00 0.99 1.01 1.01 1.01 1.01 1.02 1.00 1.00 1.00 3.02 3.02 3.00
10.0 9.5 9.0 8.5 8.0 7.5 7.0 6.5 6.0 5.5 5.0 4.5 4.0 3.5 3.0 2.5 2.0 1.5 1.0 0.5 0.0
f1 (ppm)

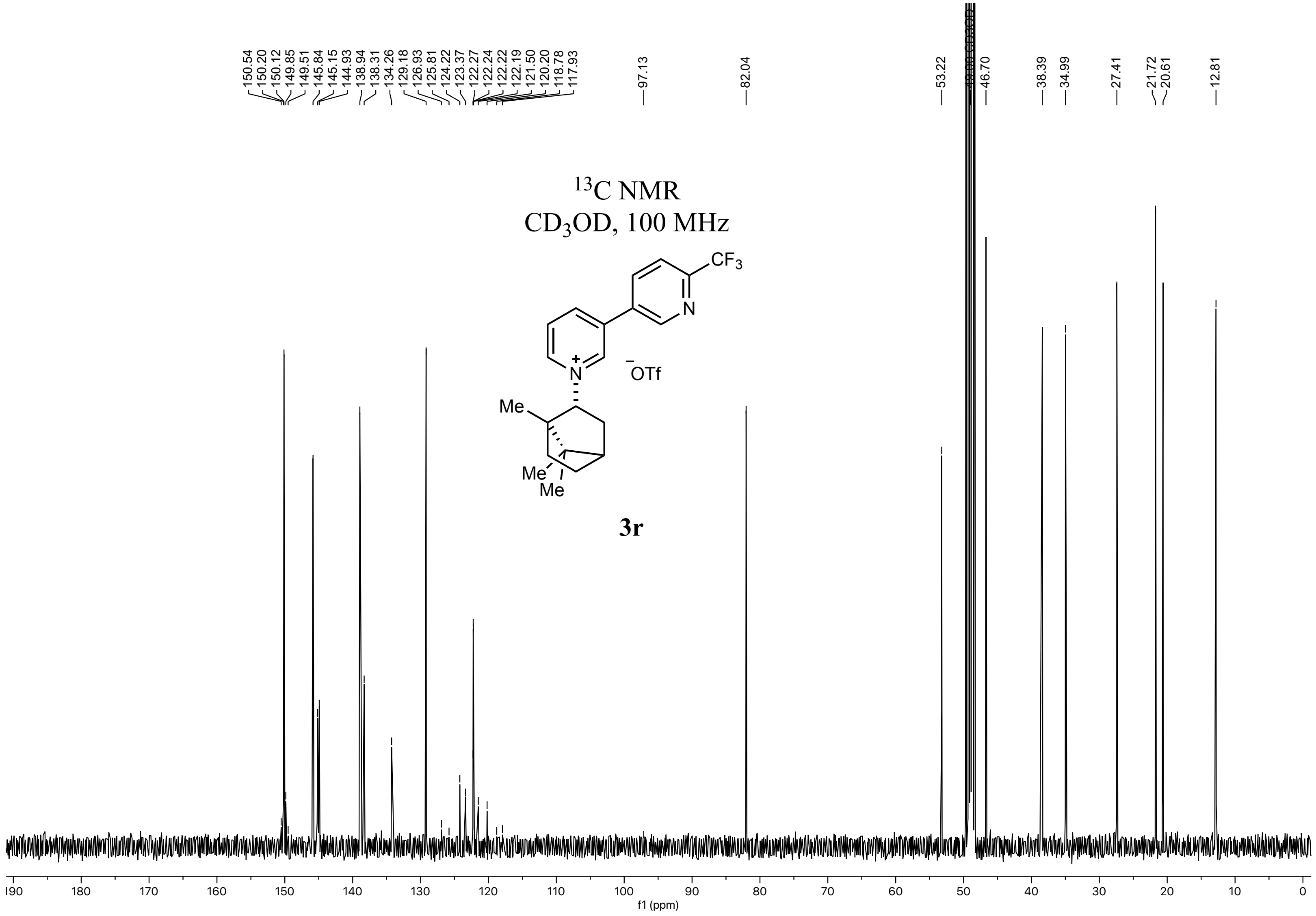

150.54
150.20
150.12
149.85
149.51
145.84
145.15
144.93
138.94
138.31
134.26
129.18
126.93
125.81
124.22
123.37
122.27
122.24
122.22
122.19
121.50
120.20
118.78
117.93
97.13
82.04
53.22
49.00 CD3OD
46.70
38.39
34.99
27.41
21.72
20.61
12.81
^{13}C NMR
CD_3OD, 100 MHz
CF_3
N
N
+
$^-$OTf
Me
Me
Me
3r
190
180
170
160
150
140
130
120
110
100
90
80
70
60
50
40
30
20
10
0
f1 (ppm)

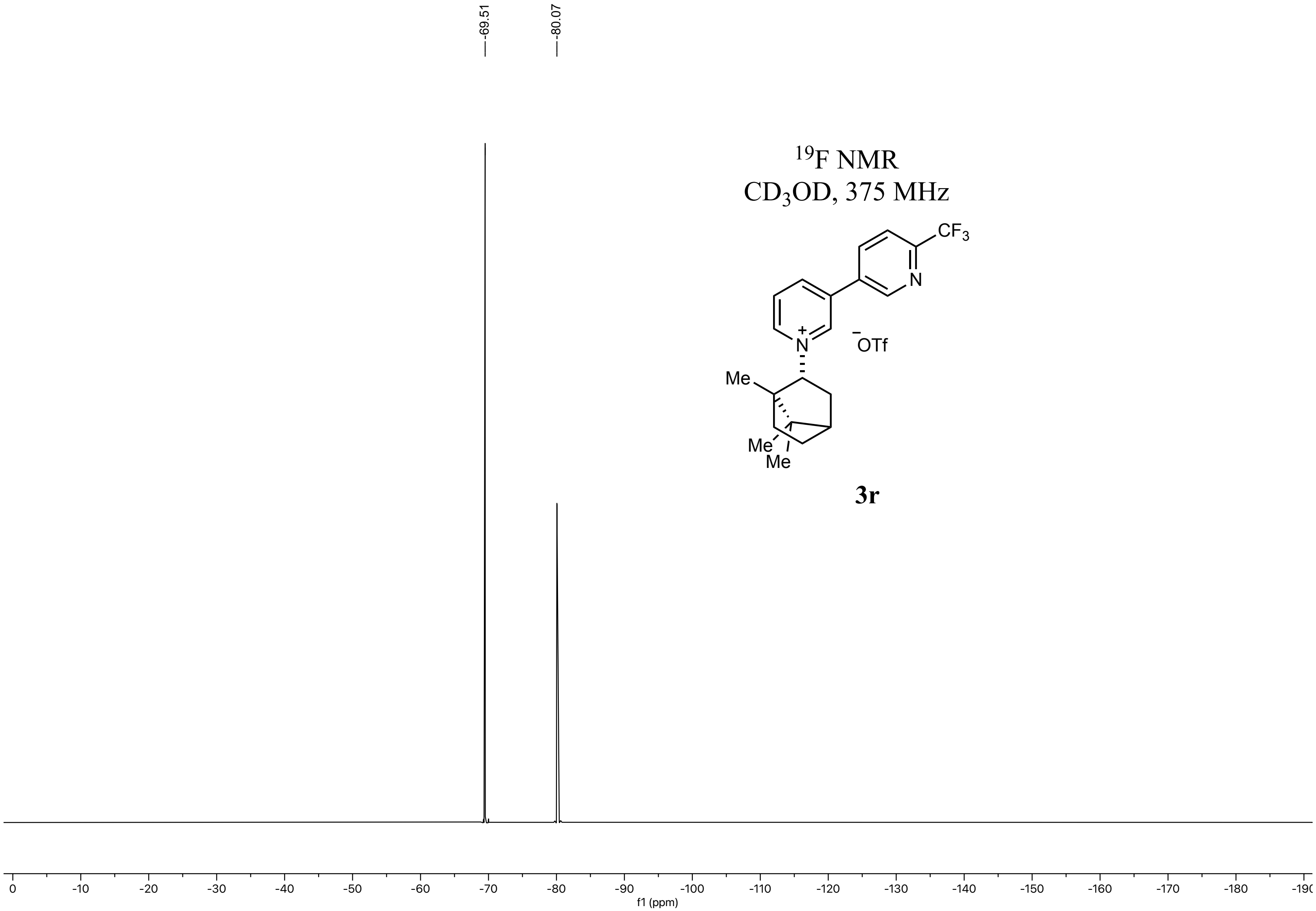
-69.51
-80.07
^{19}F NMR
CD_3OD, 375 MHz
CF_3
N
N
+
−OTf
Me
Me
Me
3r
0 -10 -20 -30 -40 -50 -60 -70 -80 -90 -100 -110 -120 -130 -140 -150 -160 -170 -180
f1 (ppm)

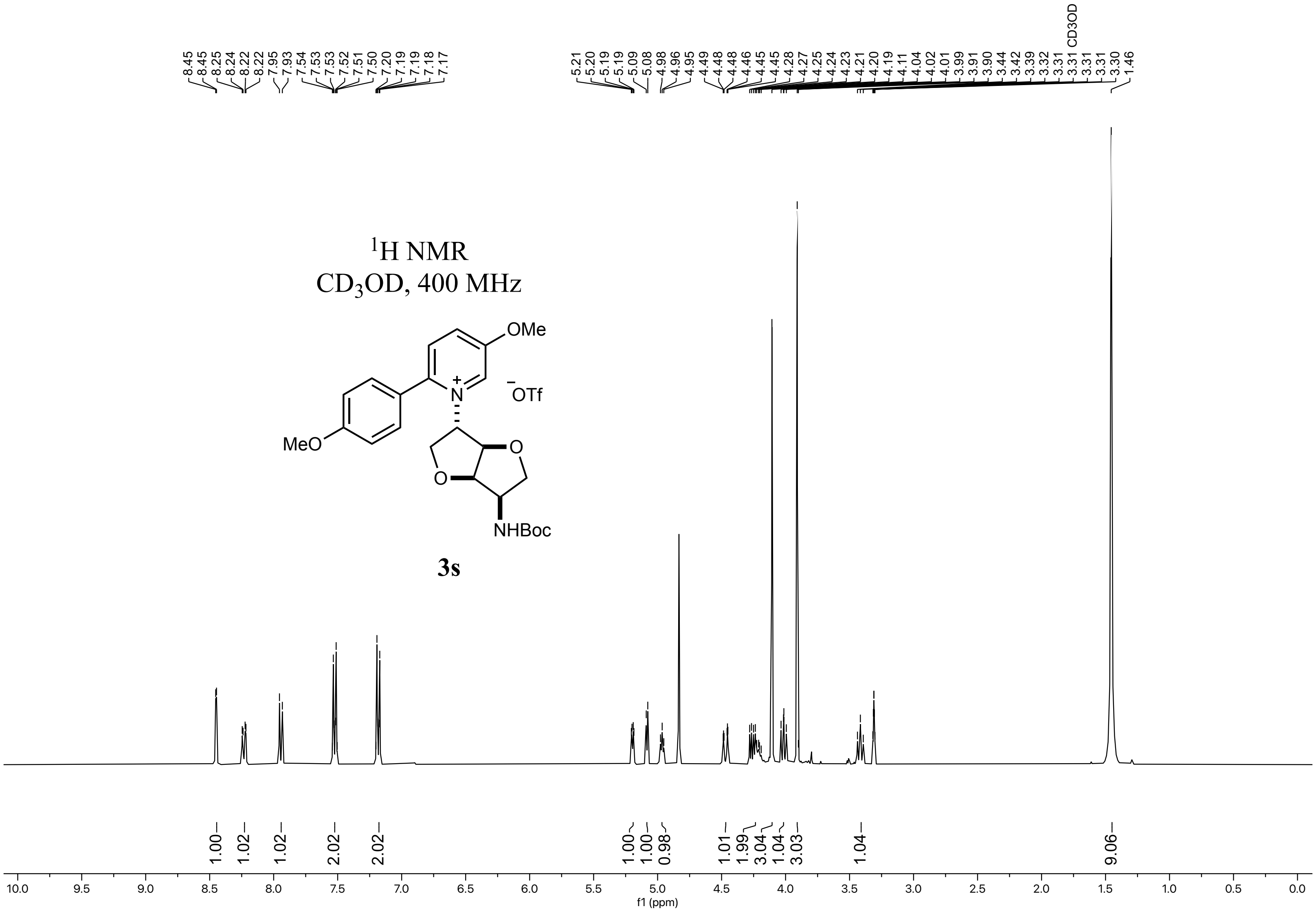

8.45
8.45
8.25
8.24
8.22
8.22
7.95
7.93
7.54
7.53
7.53
7.52
7.51
7.50
7.20
7.19
7.19
7.18
7.17
5.21
5.20
5.19
5.19
5.09
5.08
4.98
4.96
4.95
4.49
4.48
4.48
4.46
4.45
4.45
4.28
4.27
4.25
4.24
4.23
4.21
4.20
4.19
4.11
4.04
4.02
4.01
3.99
3.91
3.90
3.44
3.42
3.39
3.32
3.31
3.31 CD3OD
3.31
3.31
3.30
1.46
^{1}H NMR
CD_3OD, 400 MHz
OMe
OTf
MeO
N
O
O
NHBoc
3s
1.00
1.02
1.02
2.02
2.02
1.00
1.00
0.98
1.01
1.99
3.04
1.04
3.03
1.04
9.06
10.0
9.5
9.0
8.5
8.0
7.5
7.0
6.5
6.0
5.5
5.0
4.5
4.0
3.5
3.0
2.5
2.0
1.5
1.0
0.5
0.0
f1 (ppm)

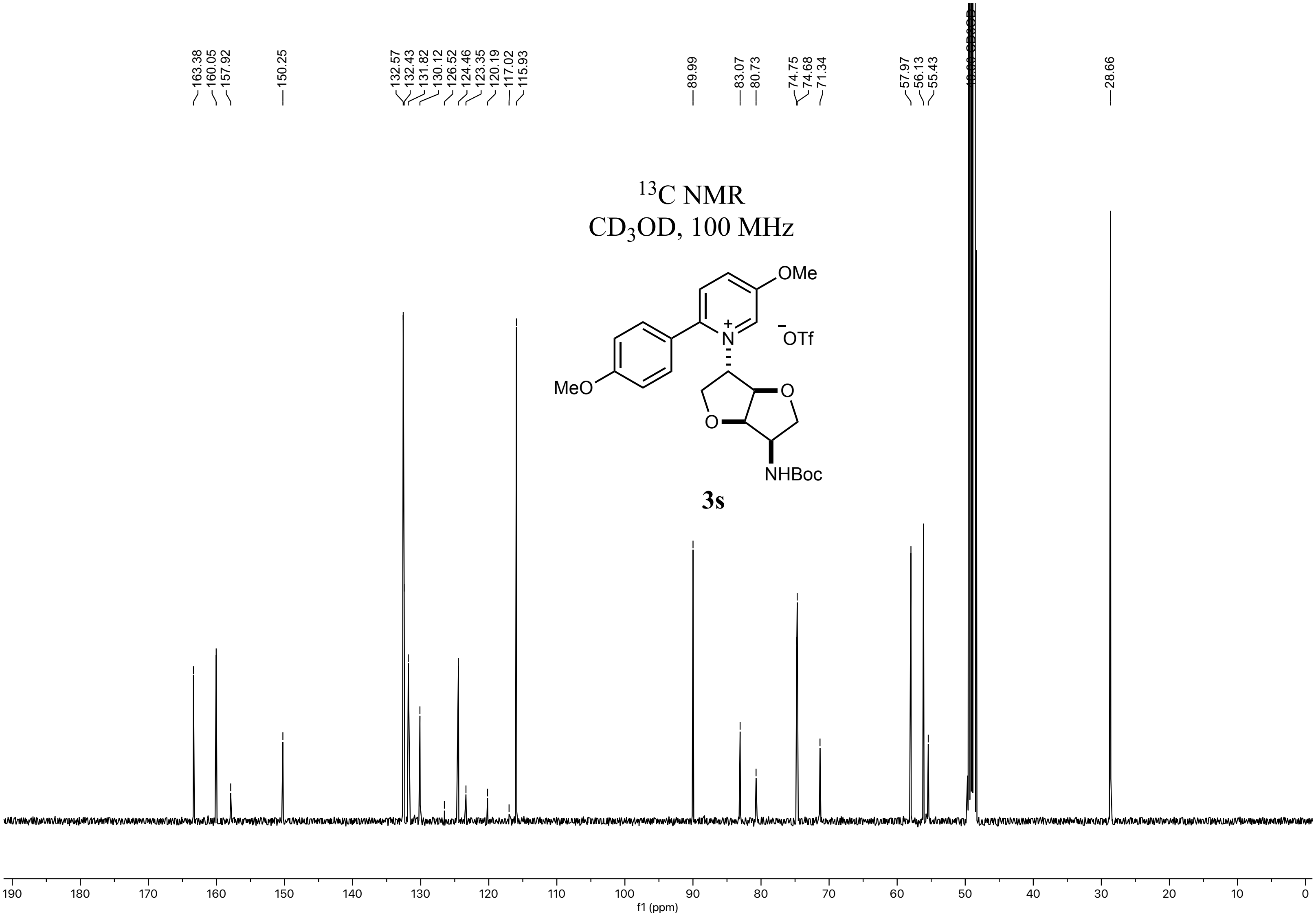
163.38
160.05
157.92
150.25
132.57
132.43
131.82
130.12
126.52
124.46
123.35
120.19
117.02
115.93
89.99
83.07
80.73
74.75
74.68
71.34
57.97
56.13
55.43
28.66
^{13}C NMR
CD_3OD, 100 MHz
OMe
OTf
MeO
O
O
NHBoc
3s
190
180
170
160
150
140
130
120
110
100
90
80
70
60
50
40
30
20
10
0
f1 (ppm)

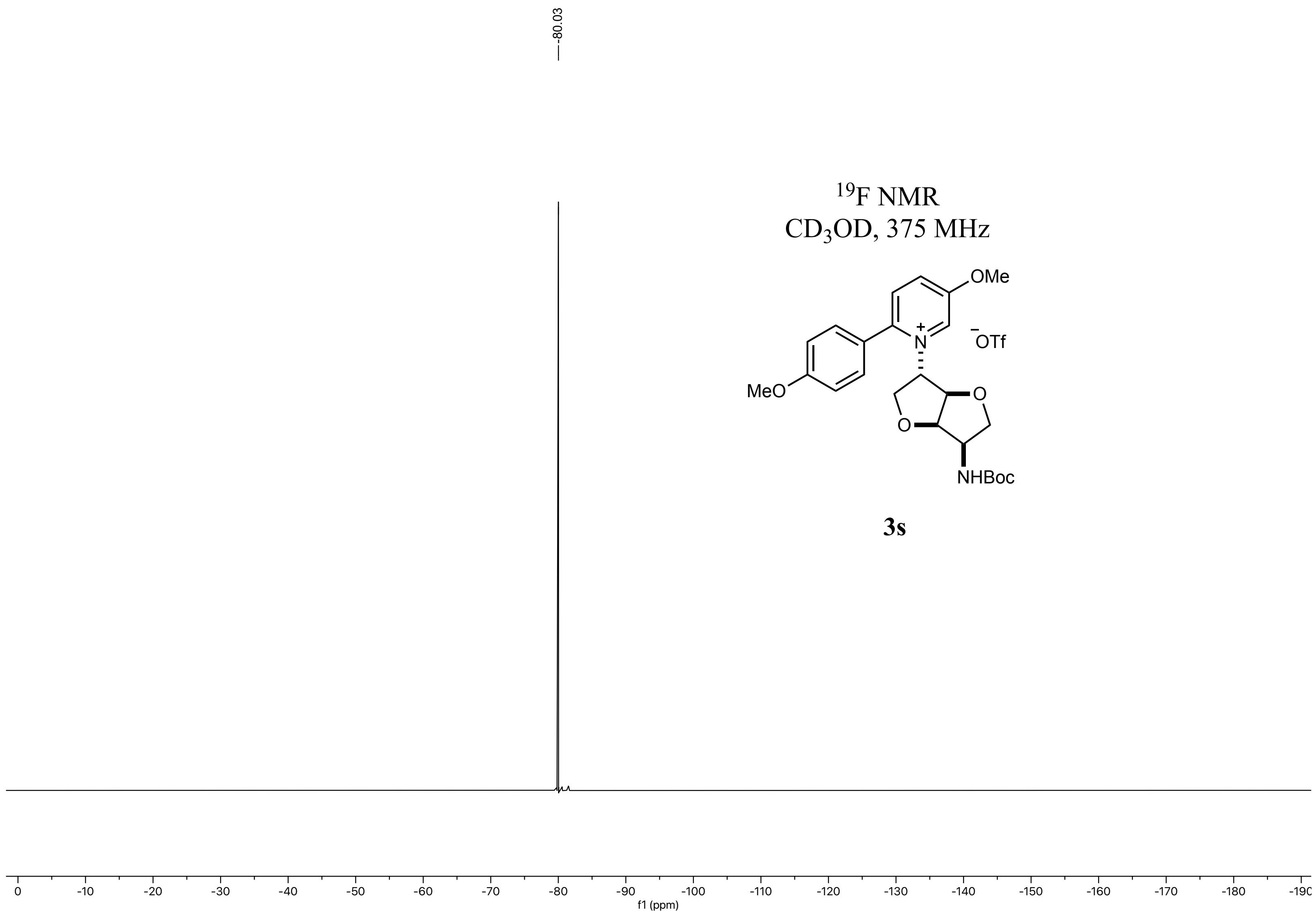
-80.03
^{19}F NMR
CD_3OD, 375 MHz
OMe
OTf
MeO
NHBoc
3s
0 -10 -20 -30 -40 -50 -60 -70 -80 -90 -100 -110 -120 -130 -140 -150 -160 -170 -180 -190
f1 (ppm)

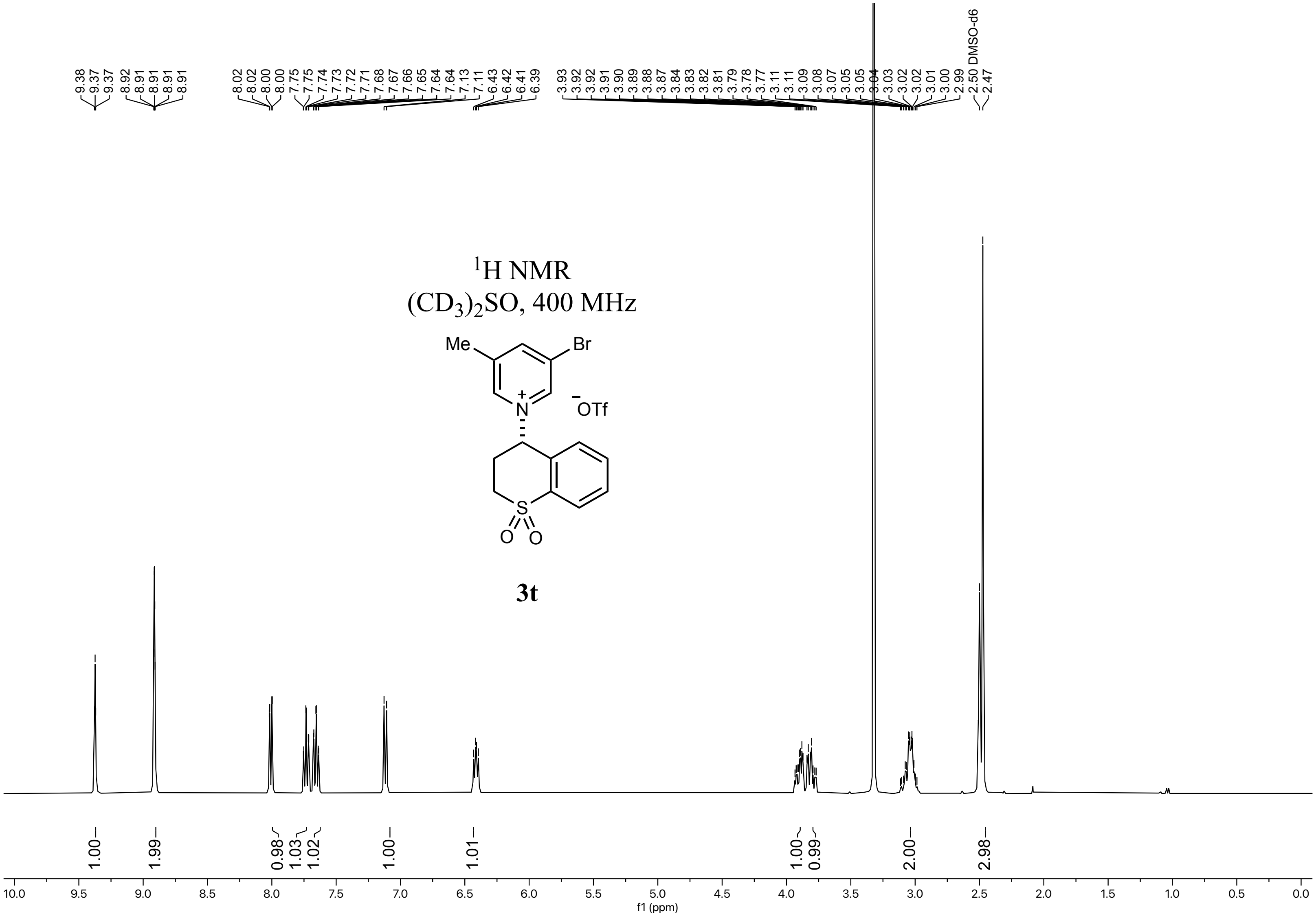

^{1}H NMR
$(CD_3)_2SO$, 400 MHz

3t

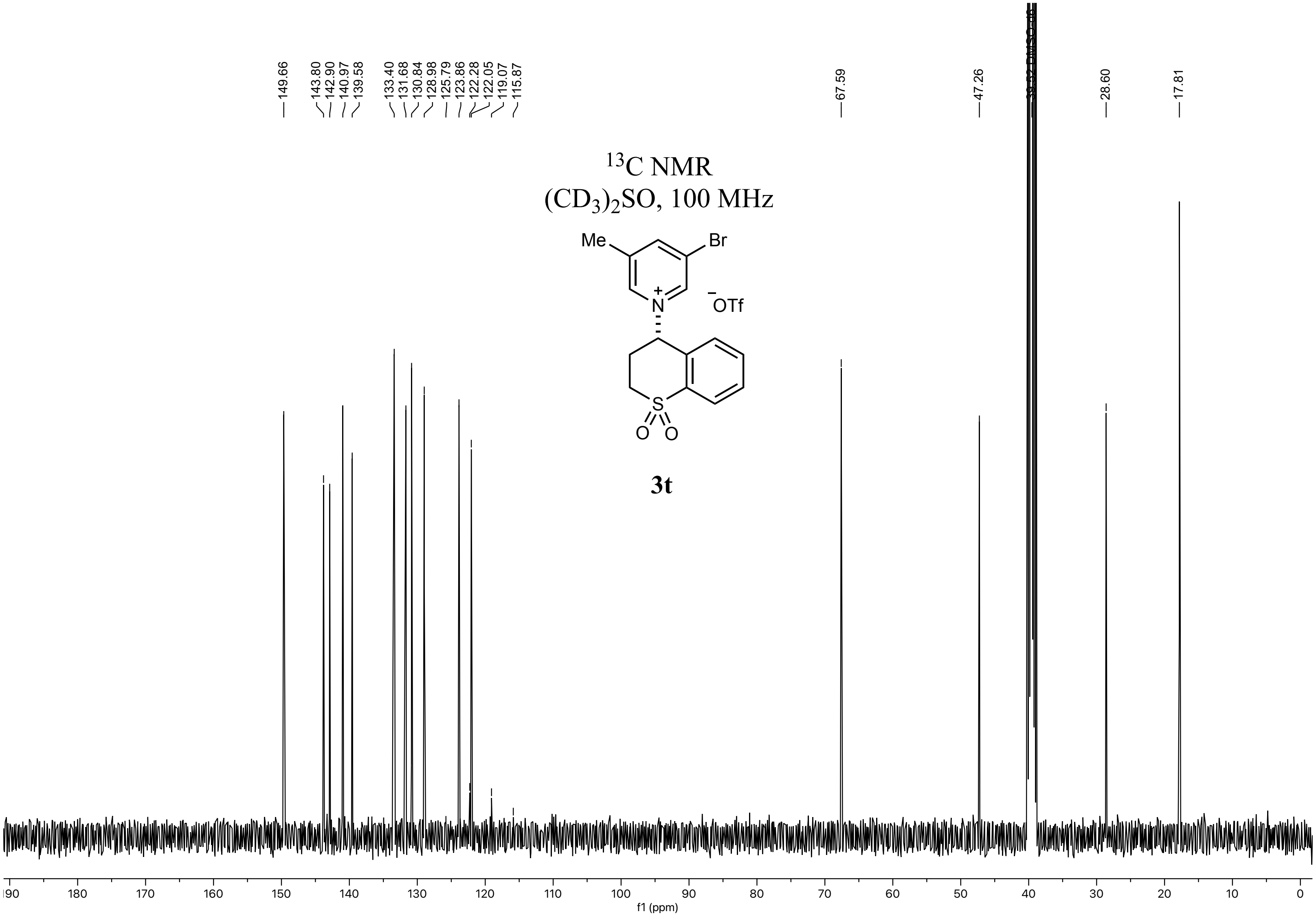
149.66
143.80
142.90
140.97
139.58
133.40
131.68
130.84
128.98
125.79
123.86
122.28
122.05
119.07
115.87
67.59
47.26
28.60
17.81
^{13}C NMR
$(CD_3)_2SO$, 100 MHz
Me
Br
N
OTf
S
O
O
3t
190
180
170
160
150
140
130
120
110
100
90
80
70
60
50
40
30
20
10
0
f1 (ppm)

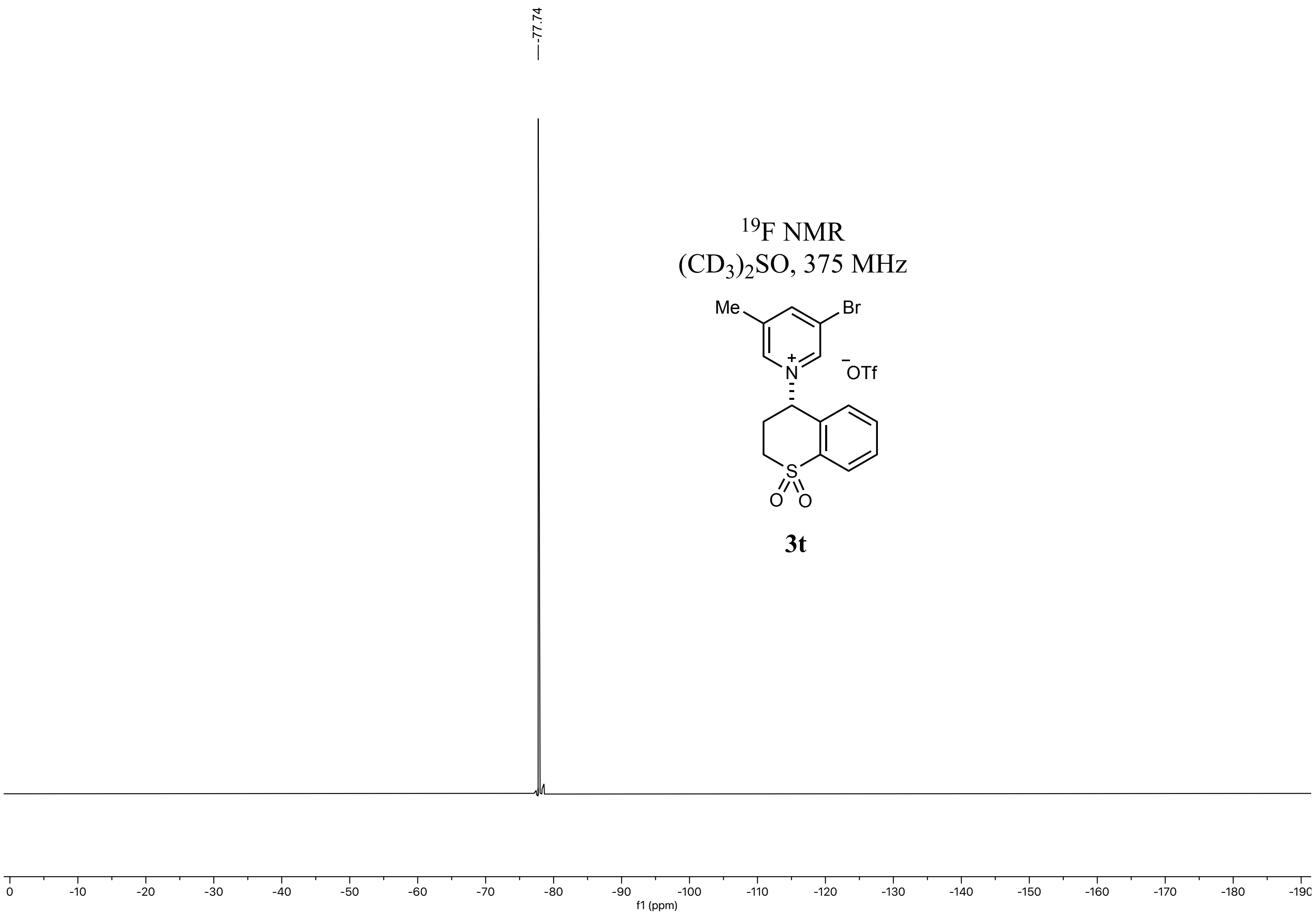

^{19}F NMR
$(CD_3)_2SO$, 375 MHz

3t

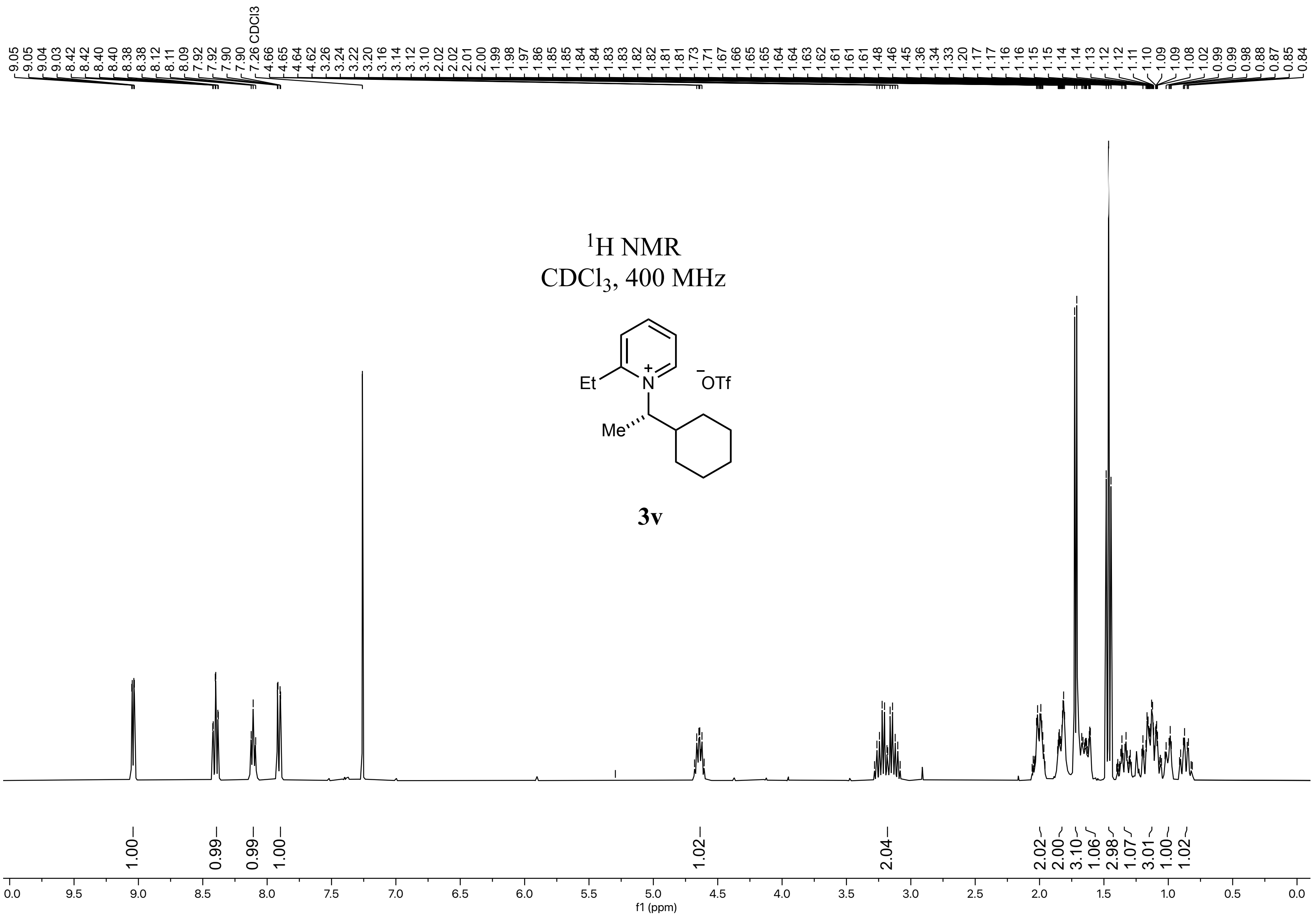

1H NMR
CDCl3, 400 MHz
Et
N
OTf
Me
3v
f1 (ppm)

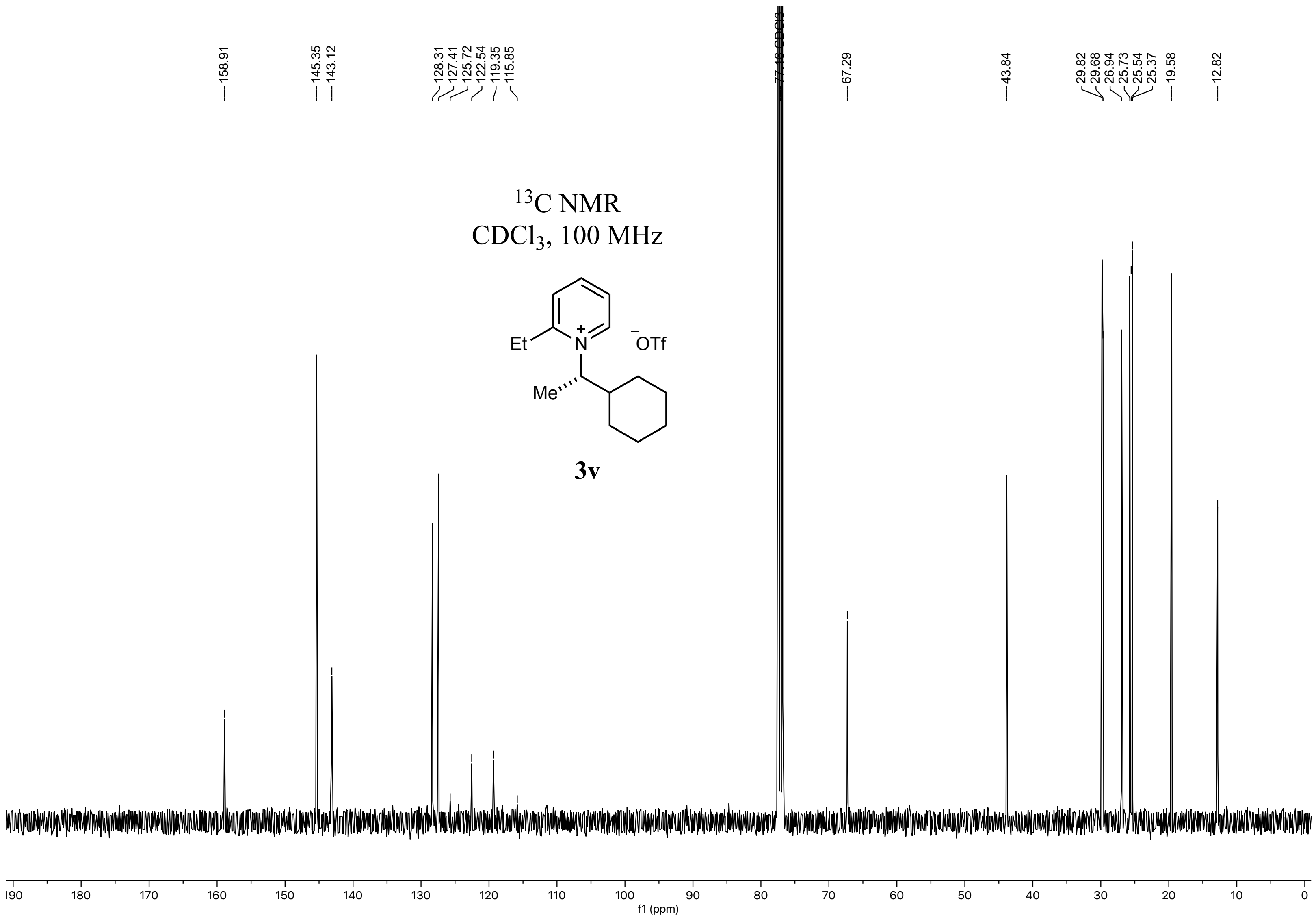

158.91
145.35
143.12
128.31
127.41
125.72
122.54
119.35
115.85
77.16 CDCl3
67.29
43.84
29.82
29.68
26.94
25.73
25.54
25.37
19.58
12.82
13C NMR
CDCl3, 100 MHz
Et
N
+
−OTf
Me
3v
f1 (ppm)

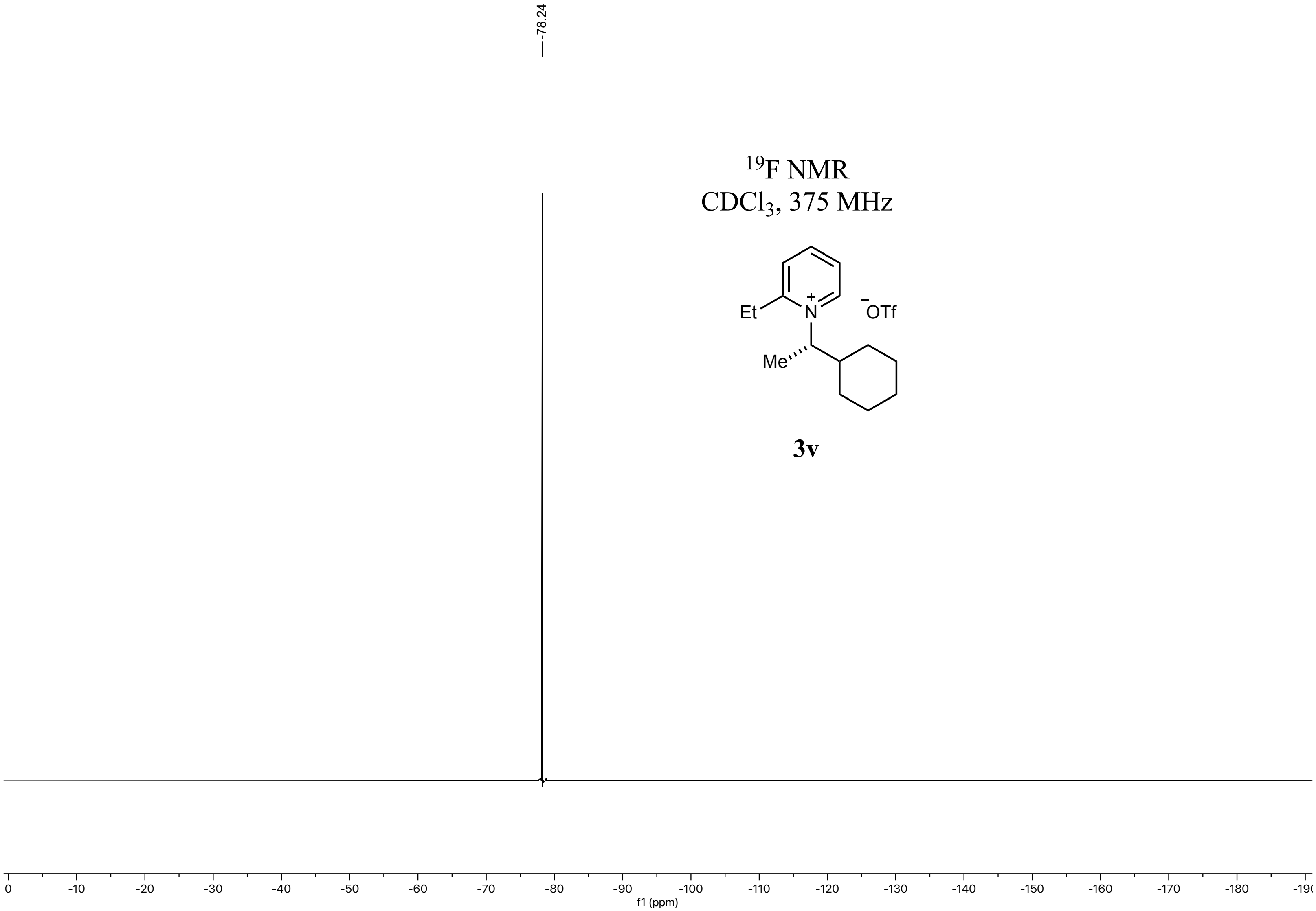
-78.24
19F NMR
CDCl3, 375 MHz
Et
N
+
−
OTf
Me
3v
0
-10
-20
-30
-40
-50
-60
-70
-80
-90
-100
-110
-120
-130
-140
-150
-160
-170
-180
f1 (ppm)

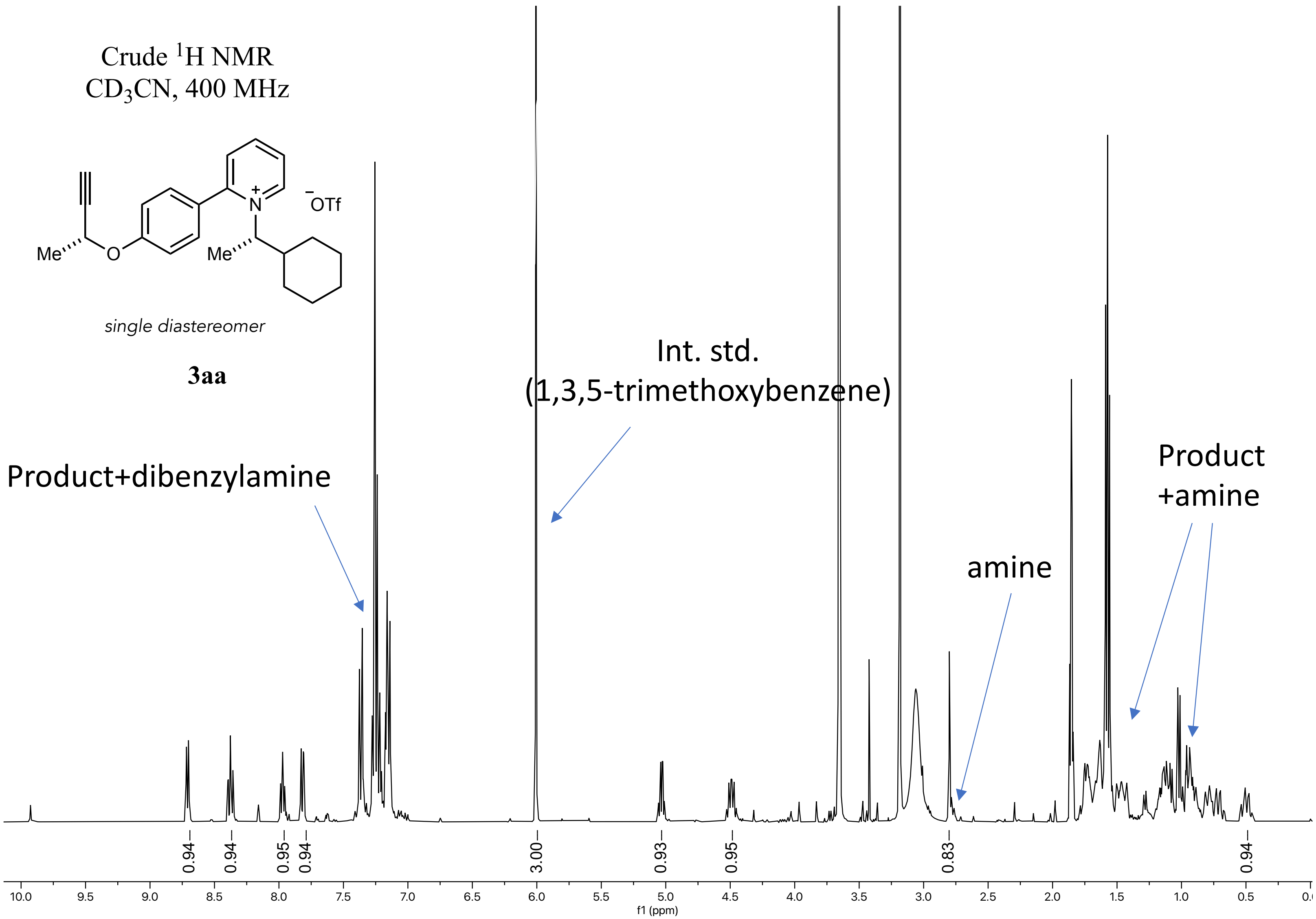

Crude ^{1}H NMR
CD_3CN, 400 MHz
OTf
Me
O
N
Me
single diastereomer
3aa
Int. std.
(1,3,5-trimethoxybenzene)
Product+dibenzylamine
amine
Product
+amine
0.94
0.94
0.95
0.94
3.00
0.93
0.95
0.83
0.94
10.0
9.5
9.0
8.5
8.0
7.5
7.0
6.5
6.0
5.5
5.0
4.5
4.0
3.5
3.0
2.5
2.0
1.5
1.0
0.5
f1 (ppm)

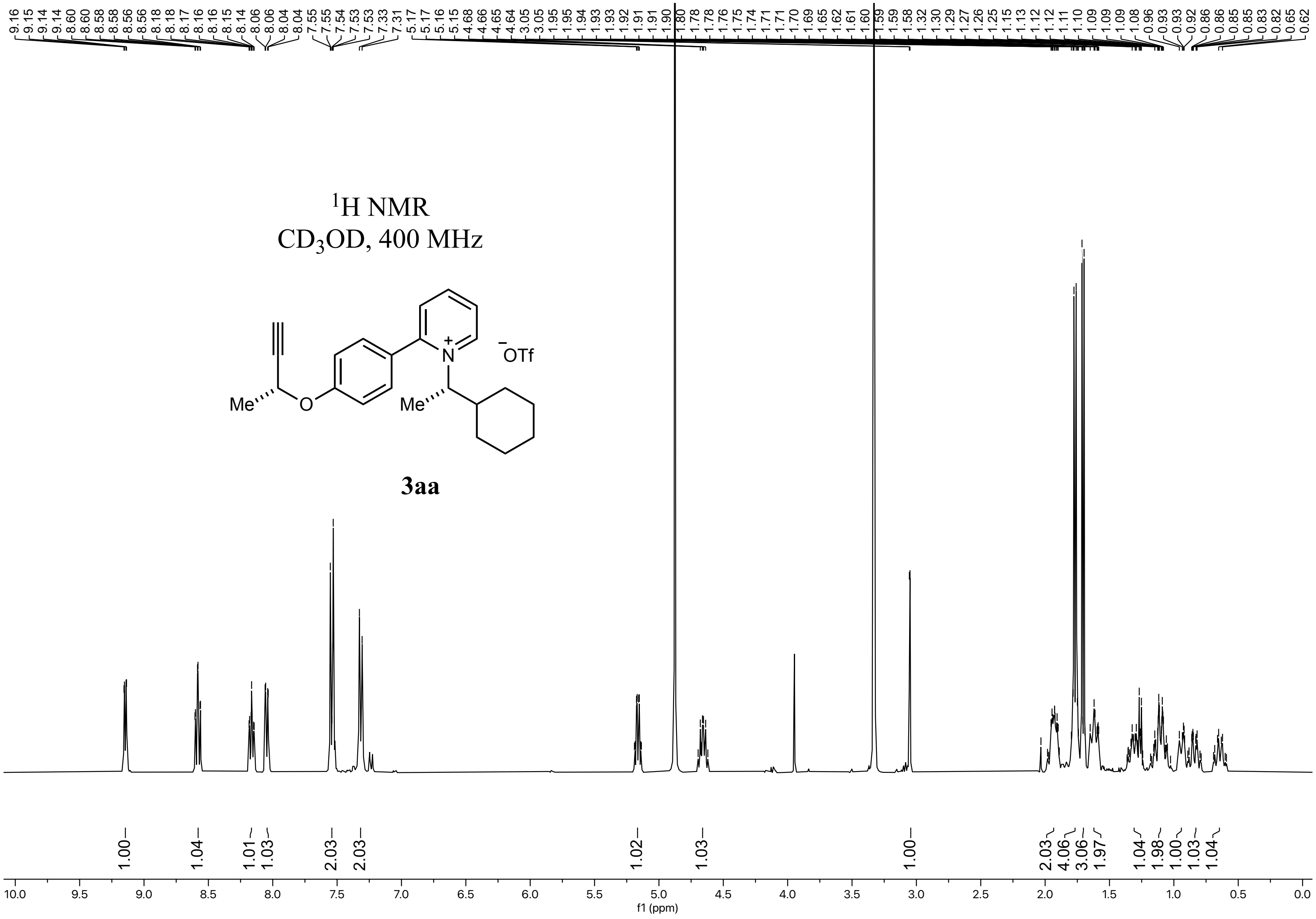

^{1}H NMR
CD_3OD, 400 MHz
Me
O
N
+
−OTf
Me
3aa
f1 (ppm)

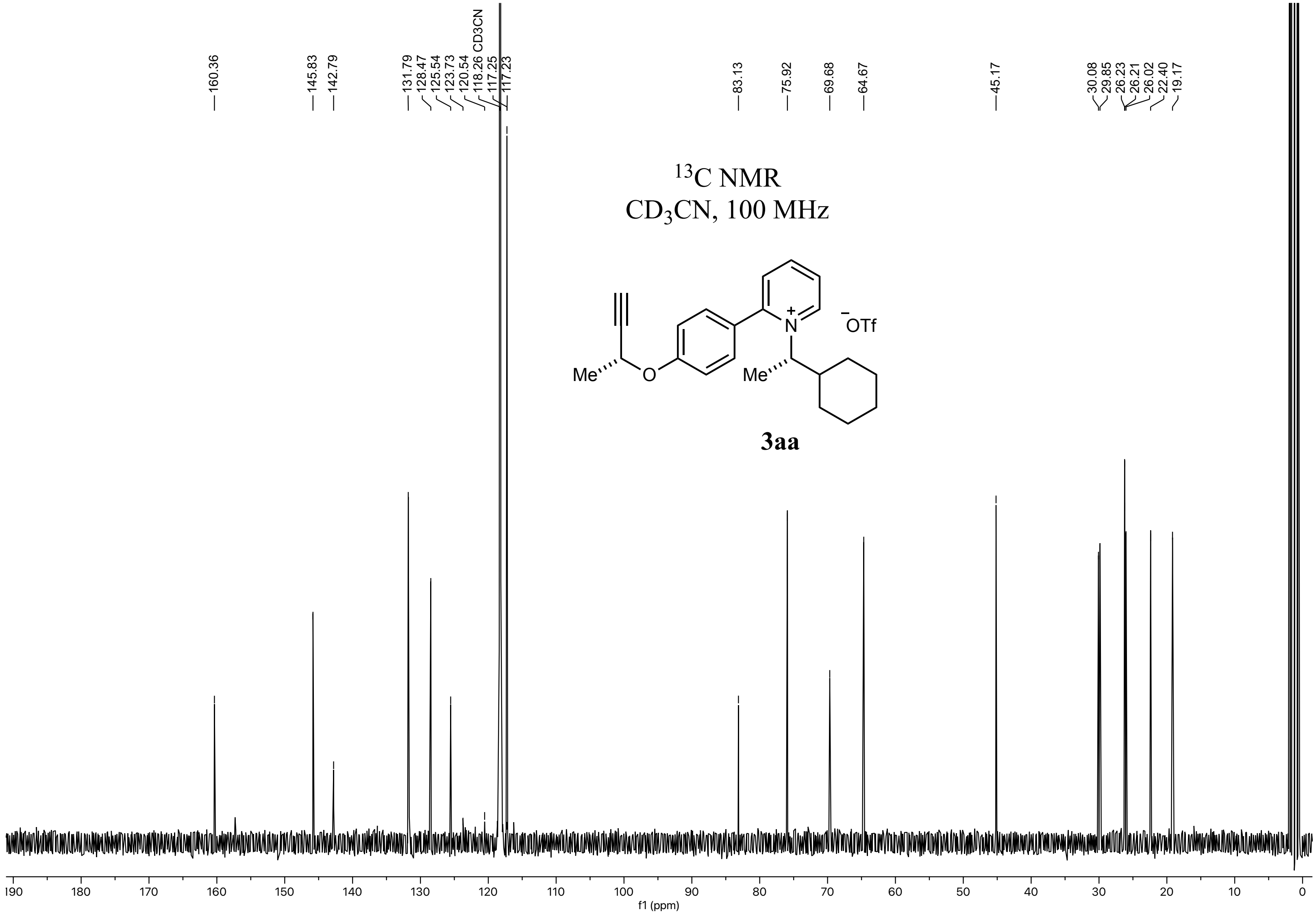

160.36
145.83
142.79
131.79
128.47
125.54
123.73
120.54
118.26 CD3CN
117.25
117.23
83.13
75.92
69.68
64.67
45.17
30.08
29.85
26.23
26.21
26.02
22.40
19.17
^{13}C NMR
CD_3CN, 100 MHz
Me
O
N
+
−
OTf
Me
3aa
f1 (ppm)

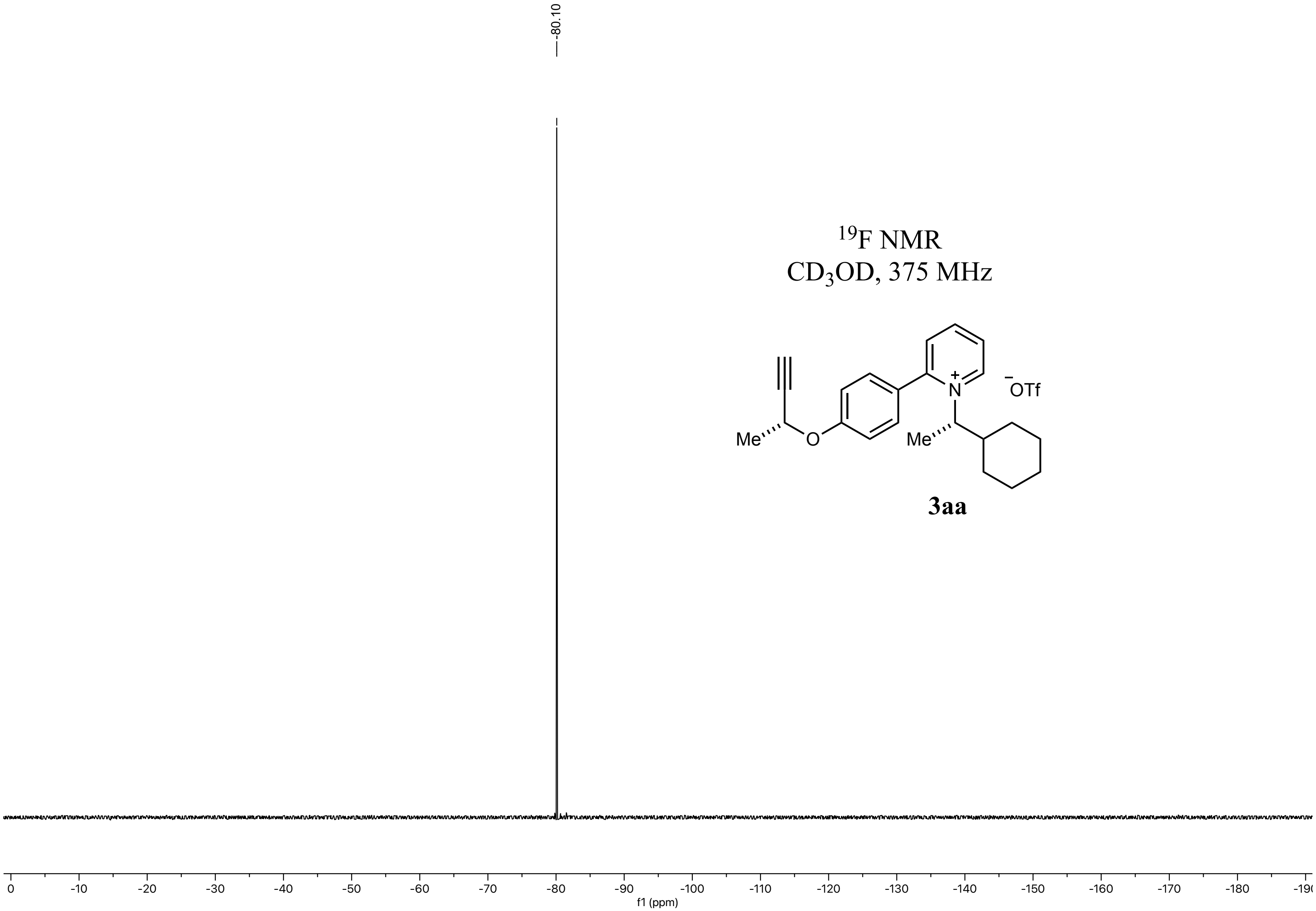

^{19}F NMR
CD_3OD, 375 MHz

3aa

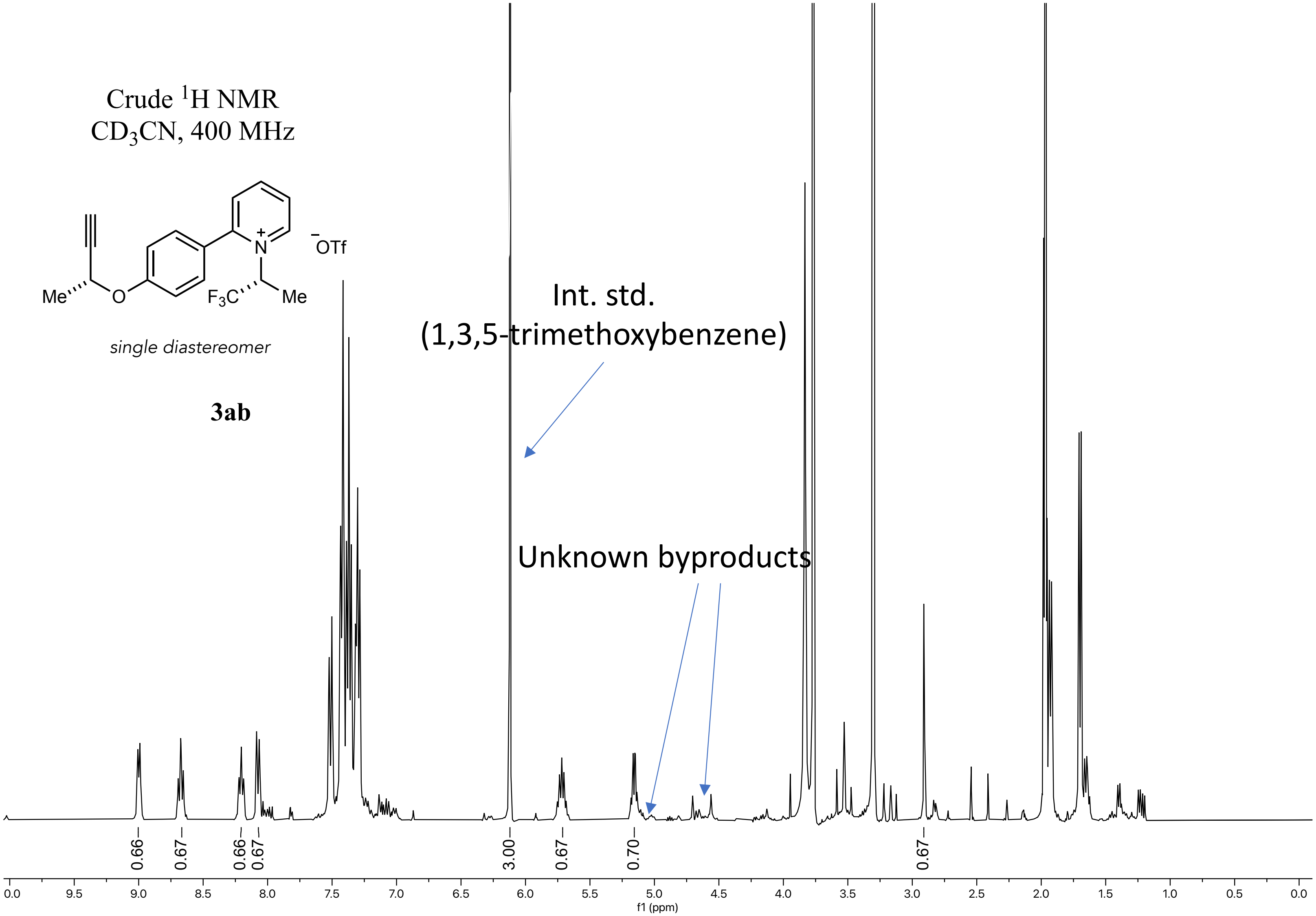

Crude 1H NMR
CD3CN, 400 MHz
OTf
Me
O
F3C
Me
N
single diastereomer
3ab
Int. std.
(1,3,5-trimethoxybenzene)
Unknown byproducts
0.66
0.67
0.66
0.67
3.00
0.67
0.70
0.67
f1 (ppm)

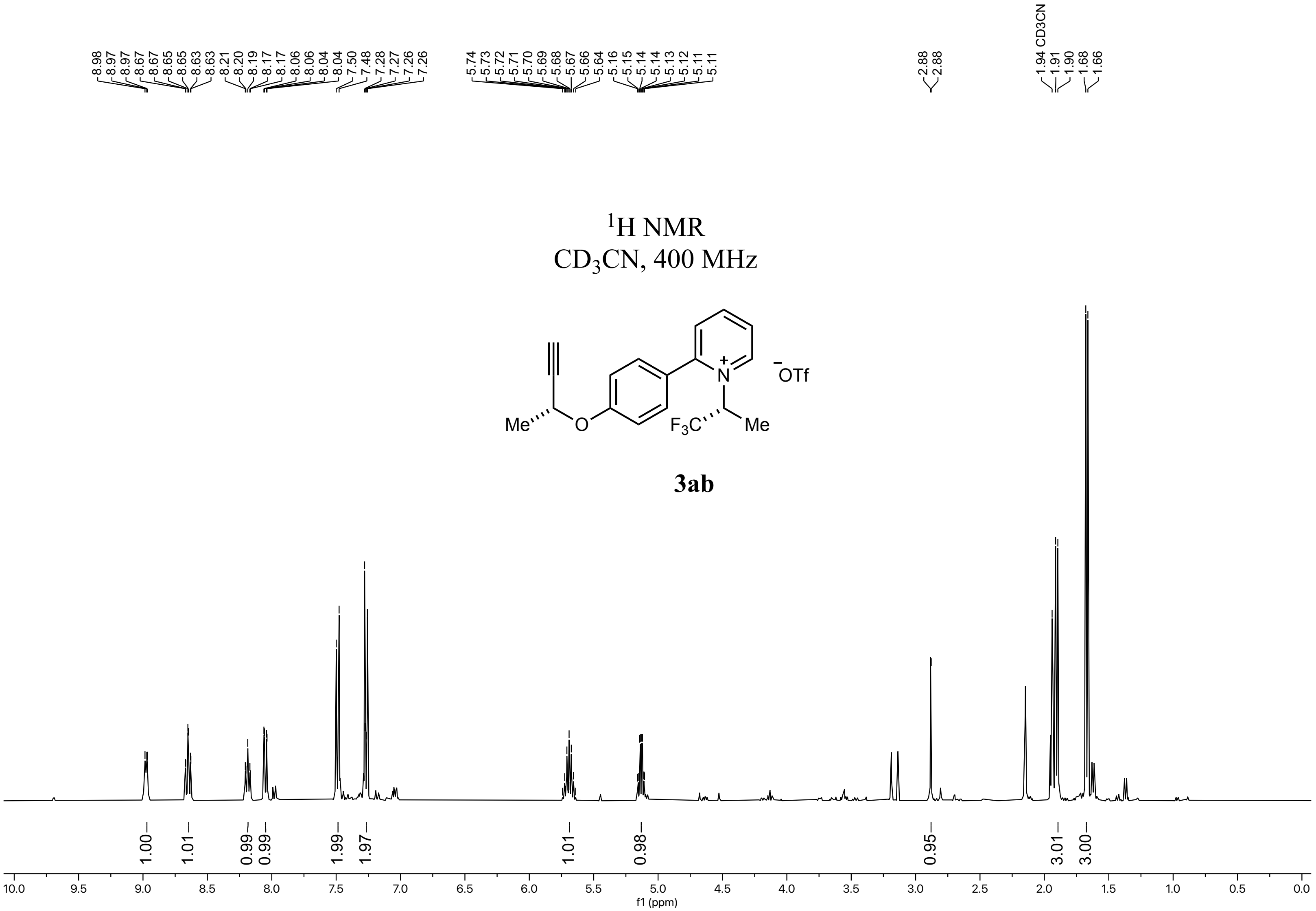

^{1}H NMR

CD_3CN, 400 MHz

3ab

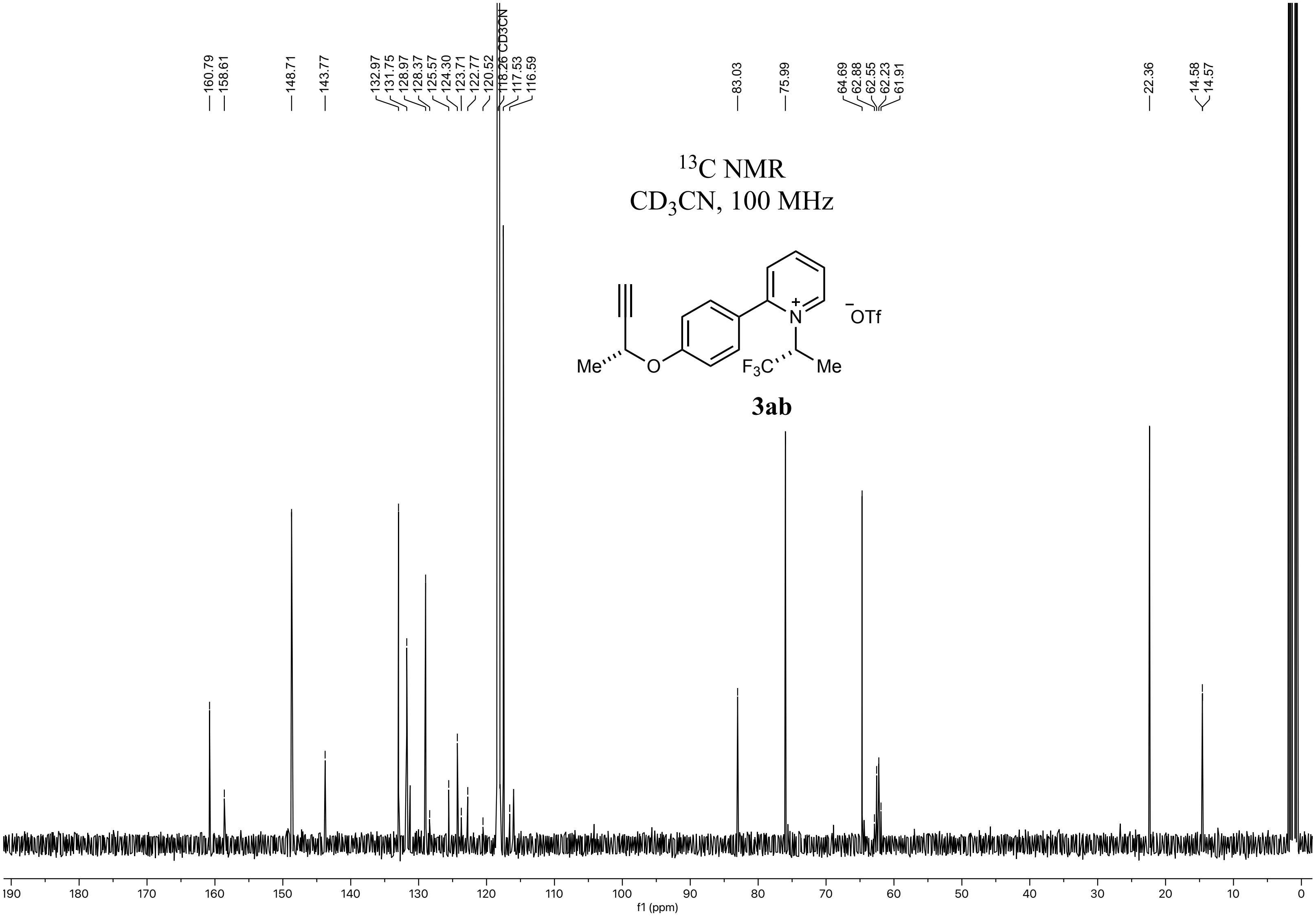
160.79
158.61
148.71
143.77
132.97
131.75
128.97
128.37
125.57
124.30
123.71
122.77
120.52
118.26 CD3CN
117.53
116.59
83.03
75.99
64.69
62.88
62.55
62.23
61.91
22.36
14.58
14.57
13C NMR
CD3CN, 100 MHz
OTf
Me
O
F3C
Me
N
3ab
190
180
170
160
150
140
130
120
110
100
90
80
70
60
50
40
30
20
10
0
f1 (ppm)

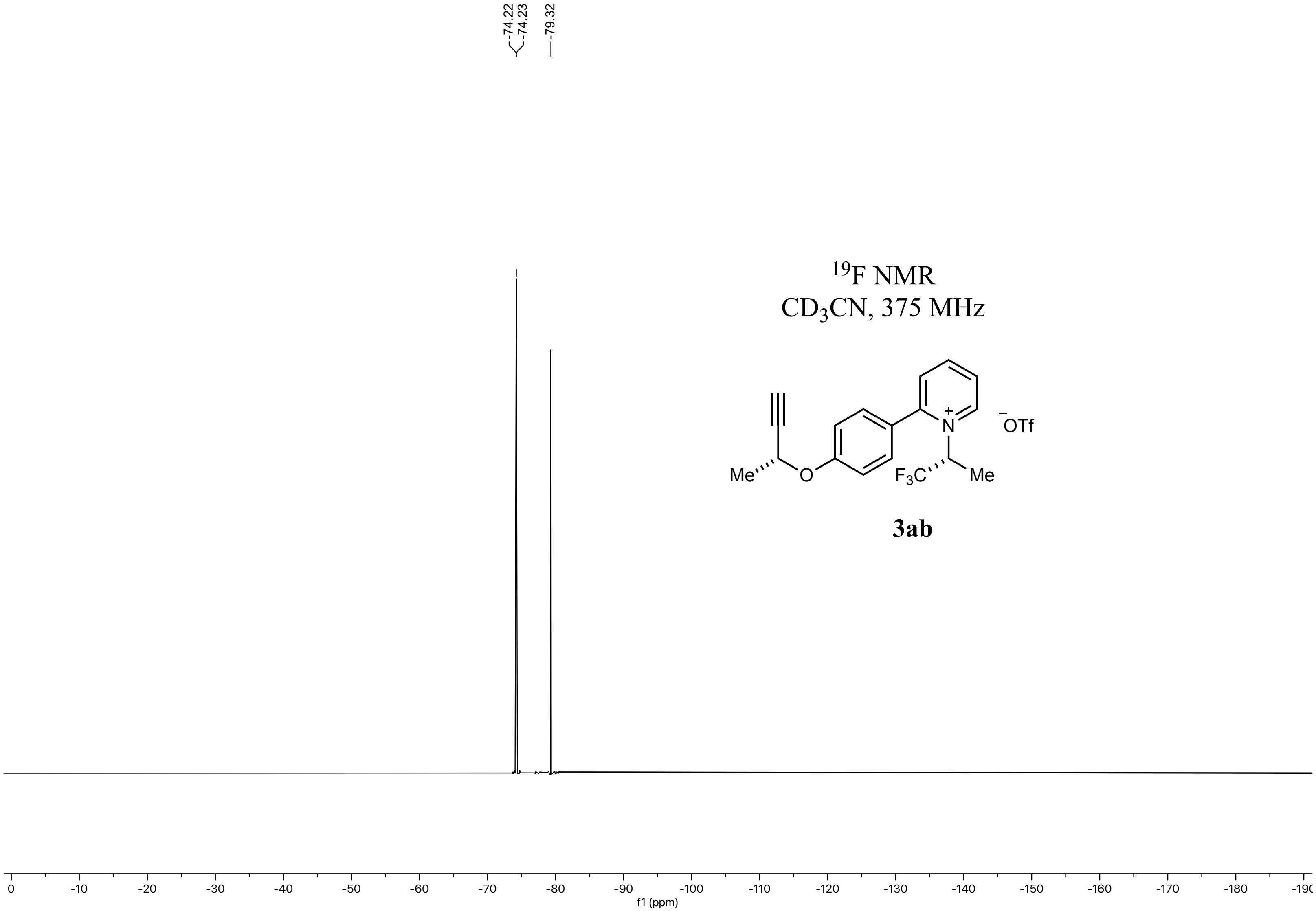

-74.22
-74.23
-79.32
19F NMR
CD3CN, 375 MHz
Me
O
N
+
OTf
F3C
Me
0
-10
-20
-30
-40
-50
-60
-70
-80
-90
-100
-110
-120
-130
-140
-150
-160
-170
-180
-190
f1 (ppm)

3ab

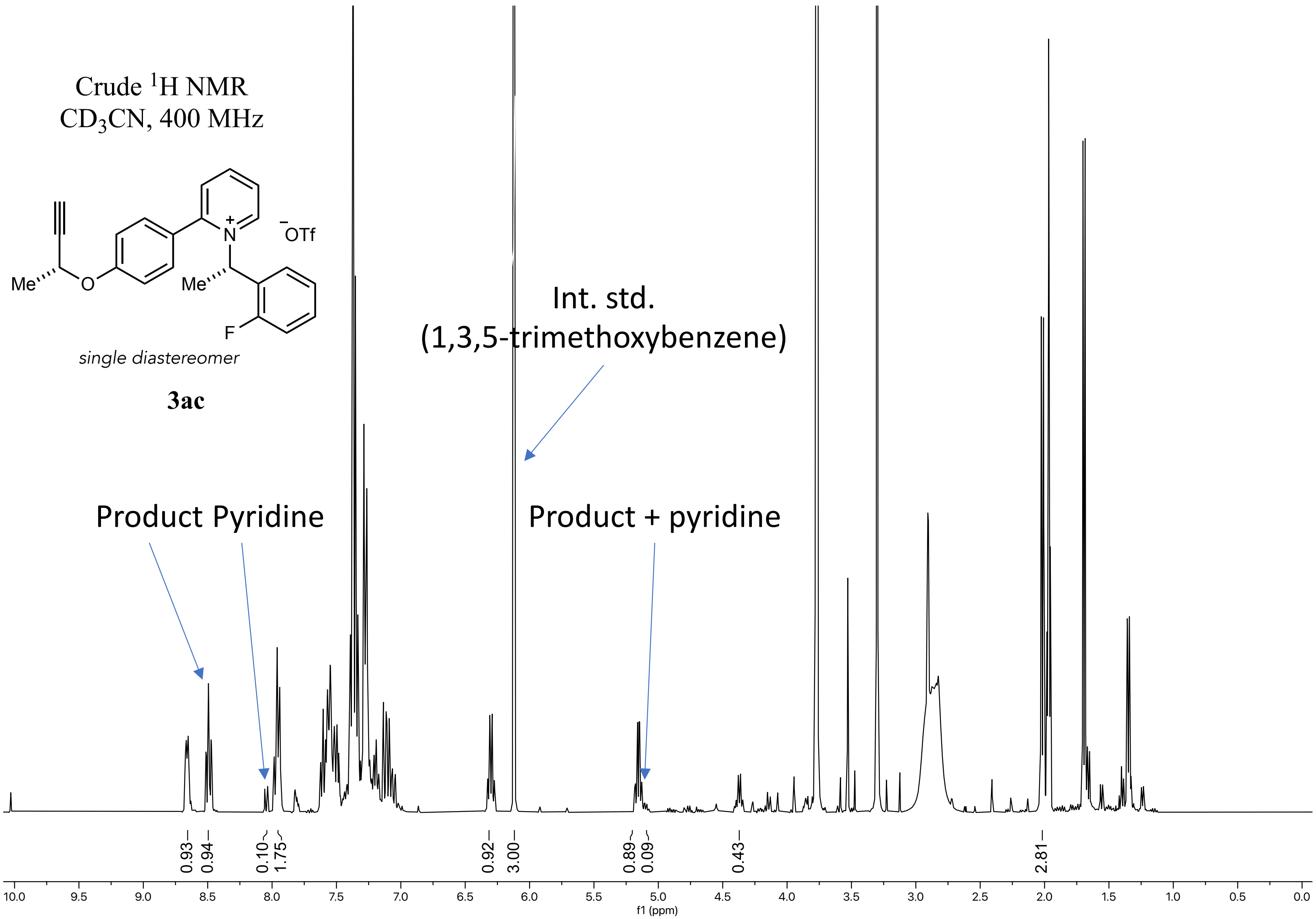
Crude ^{1}H NMR
CD_3CN, 400 MHz
OTf
Me
O
N
Me
F
single diastereomer
3ac
Int. std.
(1,3,5-trimethoxybenzene)
Product Pyridine
Product + pyridine
0.93
0.94
0.10
1.75
0.92
3.00
0.89
0.09
0.43
2.81
10.0
9.5
9.0
8.5
8.0
7.5
7.0
6.5
6.0
5.5
5.0
4.5
4.0
3.5
3.0
2.5
2.0
1.5
1.0
0.5
0.0
f1 (ppm)

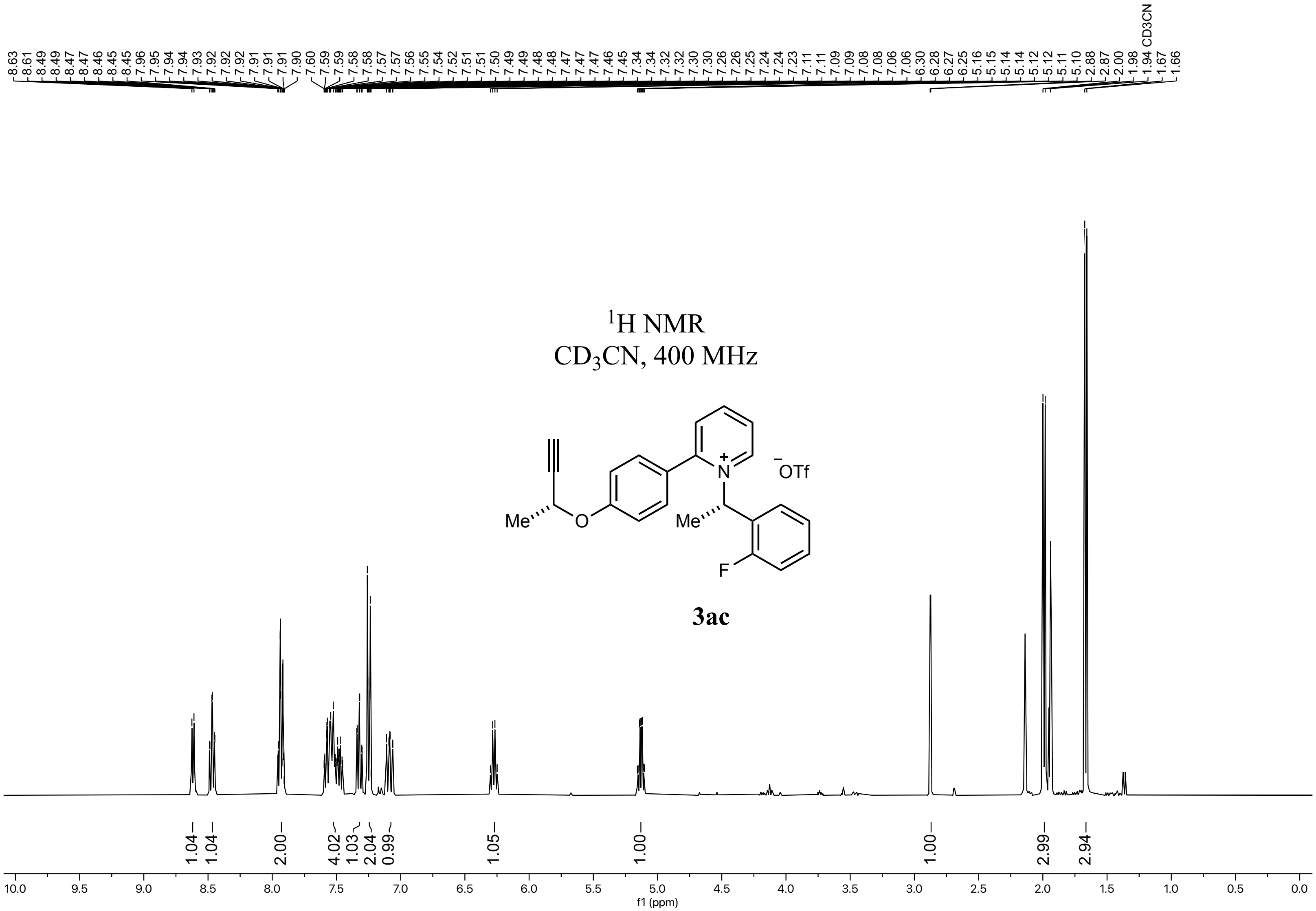

1H NMR
CD3CN, 400 MHz
OTf
Me
O
N
F
3ac
CD3CN
f1 (ppm)

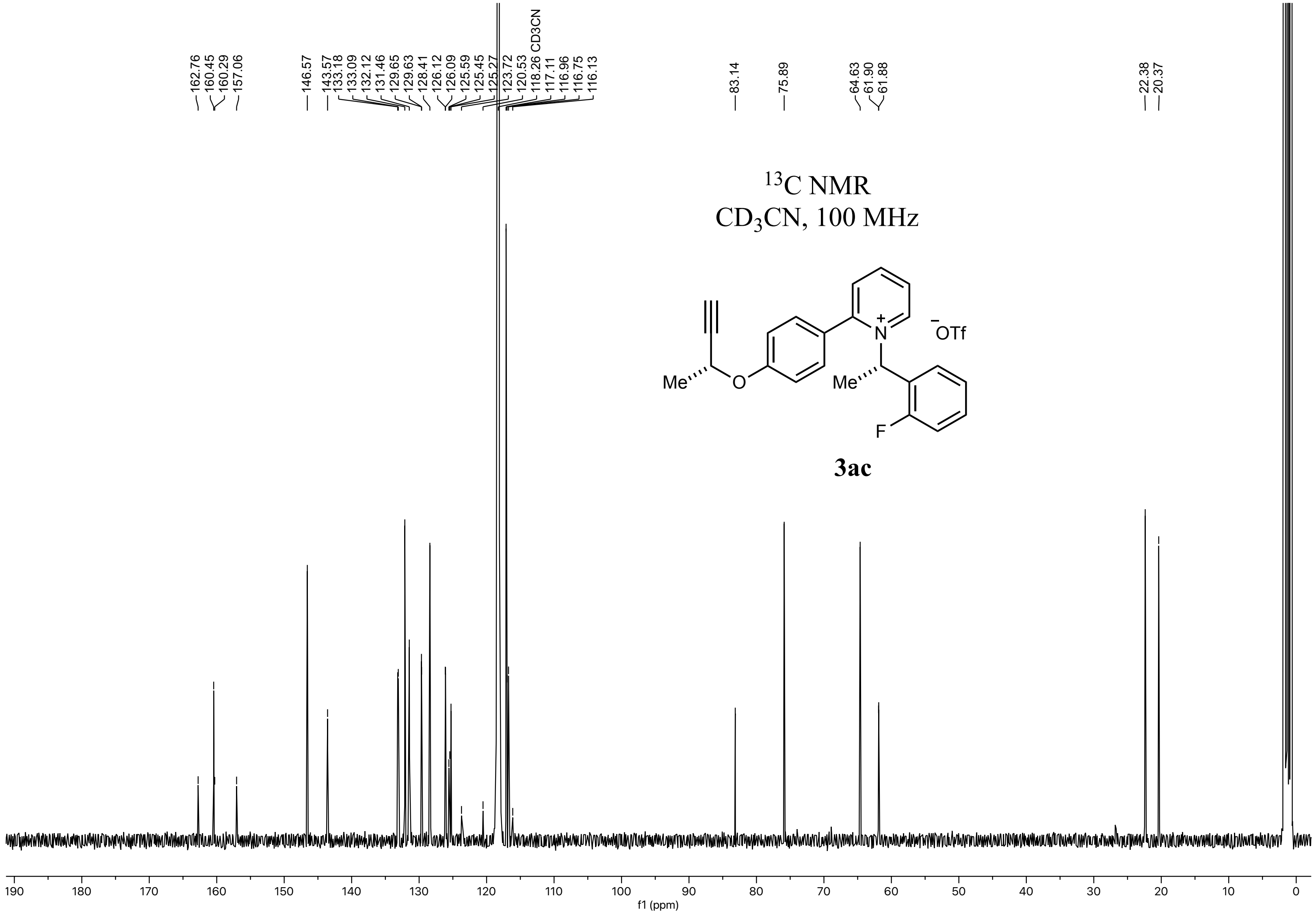
162.76
160.45
160.29
157.06
146.57
143.57
133.18
133.09
132.12
131.46
129.65
129.63
128.41
126.12
126.09
125.59
125.45
125.27
123.72
120.53
118.26 CD3CN
117.11
116.96
116.75
116.13
83.14
75.89
64.63
61.90
61.88
22.38
20.37
^{13}C NMR
CD_3CN, 100 MHz
OTf
N
Me
O
Me
F
f1 (ppm)
3ac

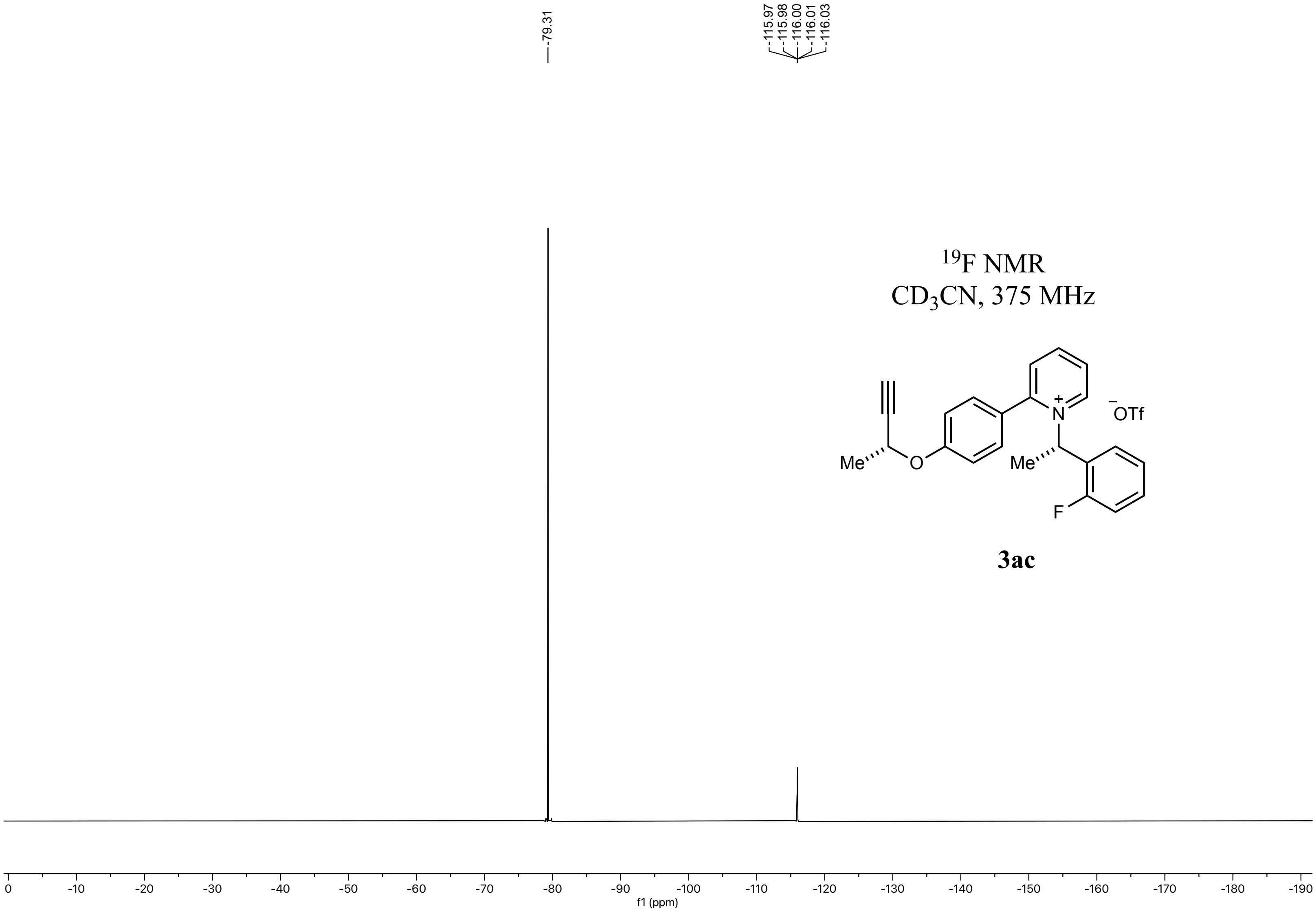

^{19}F NMR
CD_3CN, 375 MHz

3ac

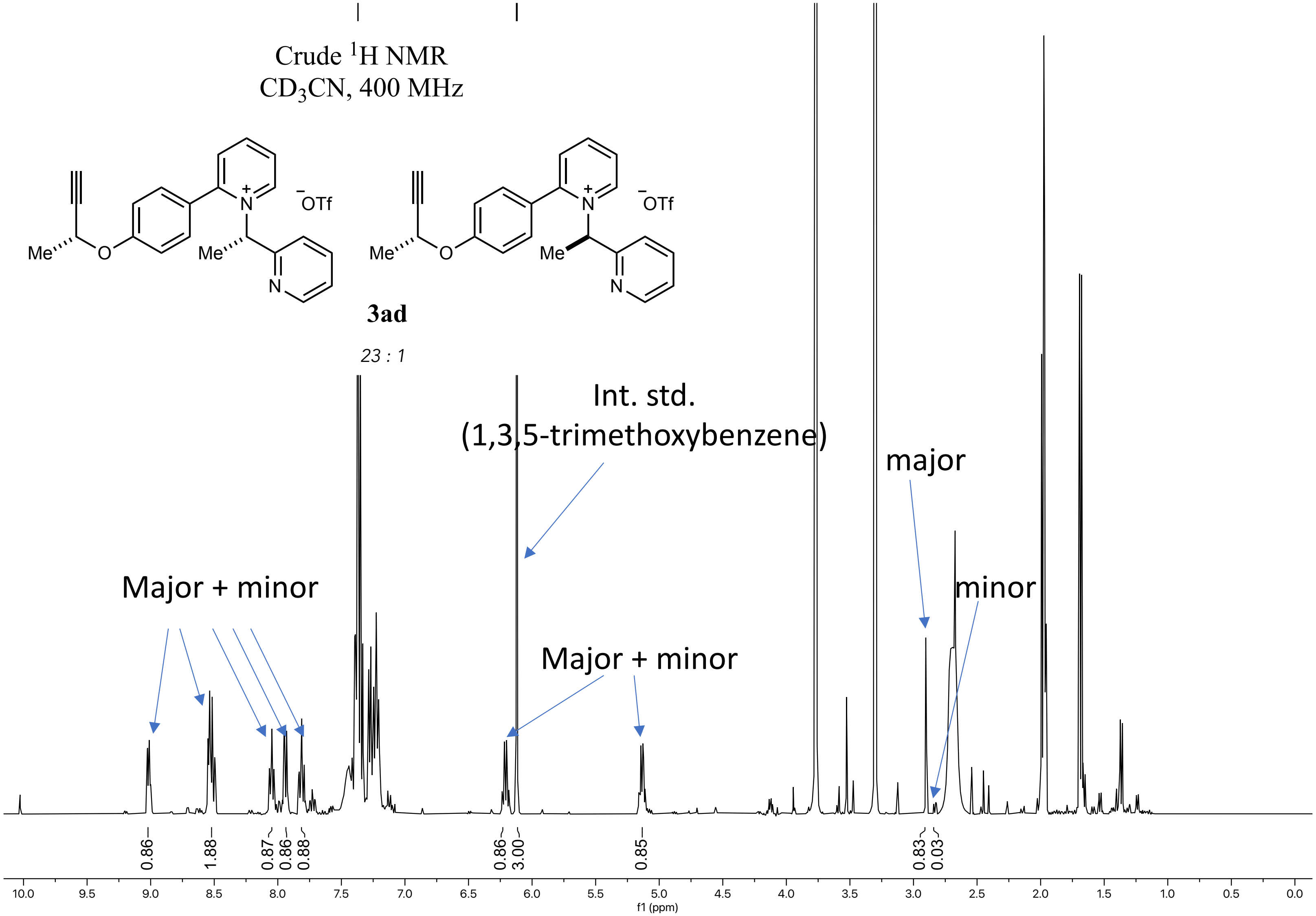

Crude 1H NMR
CD3CN, 400 MHz
OTf
Me
O
N
3ad
23 : 1
Int. std.
(1,3,5-trimethoxybenzene)
major
minor
Major + minor
Major + minor
0.86
1.88
0.87
0.86
0.88
0.86
3.00
0.85
0.83
0.03
f1 (ppm)

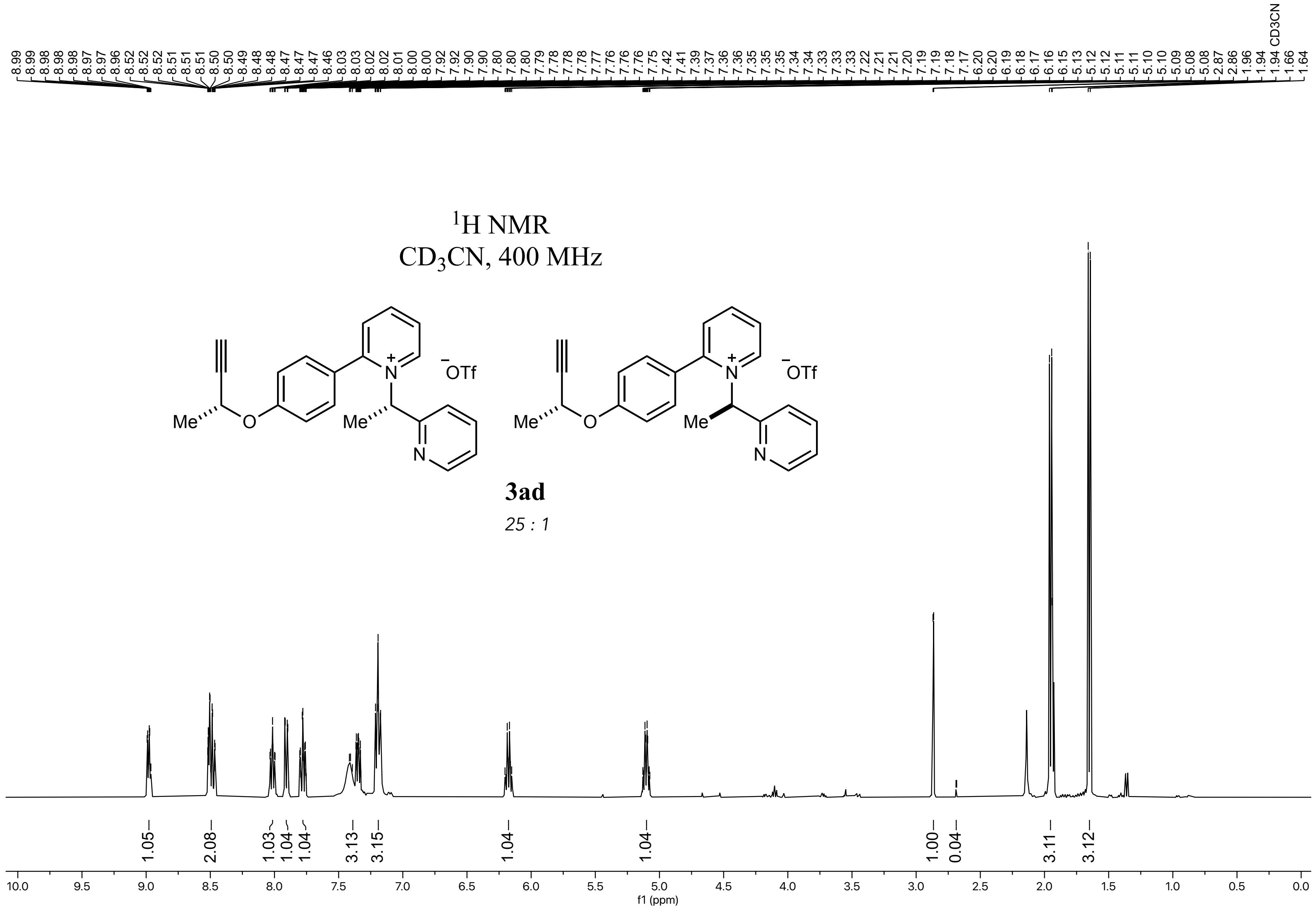
8.99 8.99 8.98 8.98 8.97 8.97 8.96 8.52 8.52 8.51 8.51 8.50 8.50 8.49 8.48 8.48 8.47 8.47 8.46 8.03 8.03 8.02 8.02 8.01 8.00 8.00 7.92 7.92 7.90 7.90 7.80 7.80 7.79 7.78 7.78 7.77 7.76 7.76 7.76 7.75 7.42 7.41 7.39 7.37 7.36 7.35 7.35 7.34 7.33 7.33 7.22 7.21 7.21 7.20 7.19 7.19 7.18 7.17 6.20 6.20 6.19 6.18 6.17 6.16 6.15 5.13 5.12 5.12 5.11 5.10 5.10 5.09 5.08 2.87 2.86 1.96 1.94 CD3CN 1.66 1.64
^{1}H NMR
CD_3CN, 400 MHz
OTf
Me
O
N
OTf
Me
O
N
3ad
25 : 1
1.05 2.08 1.03 1.04 1.04 3.13 3.15 1.04 1.04 1.00 0.04 3.11 3.12
10.0 9.5 9.0 8.5 8.0 7.5 7.0 6.5 6.0 5.5 5.0 4.5 4.0 3.5 3.0 2.5 2.0 1.5 1.0 0.5 0.0
f1 (ppm)

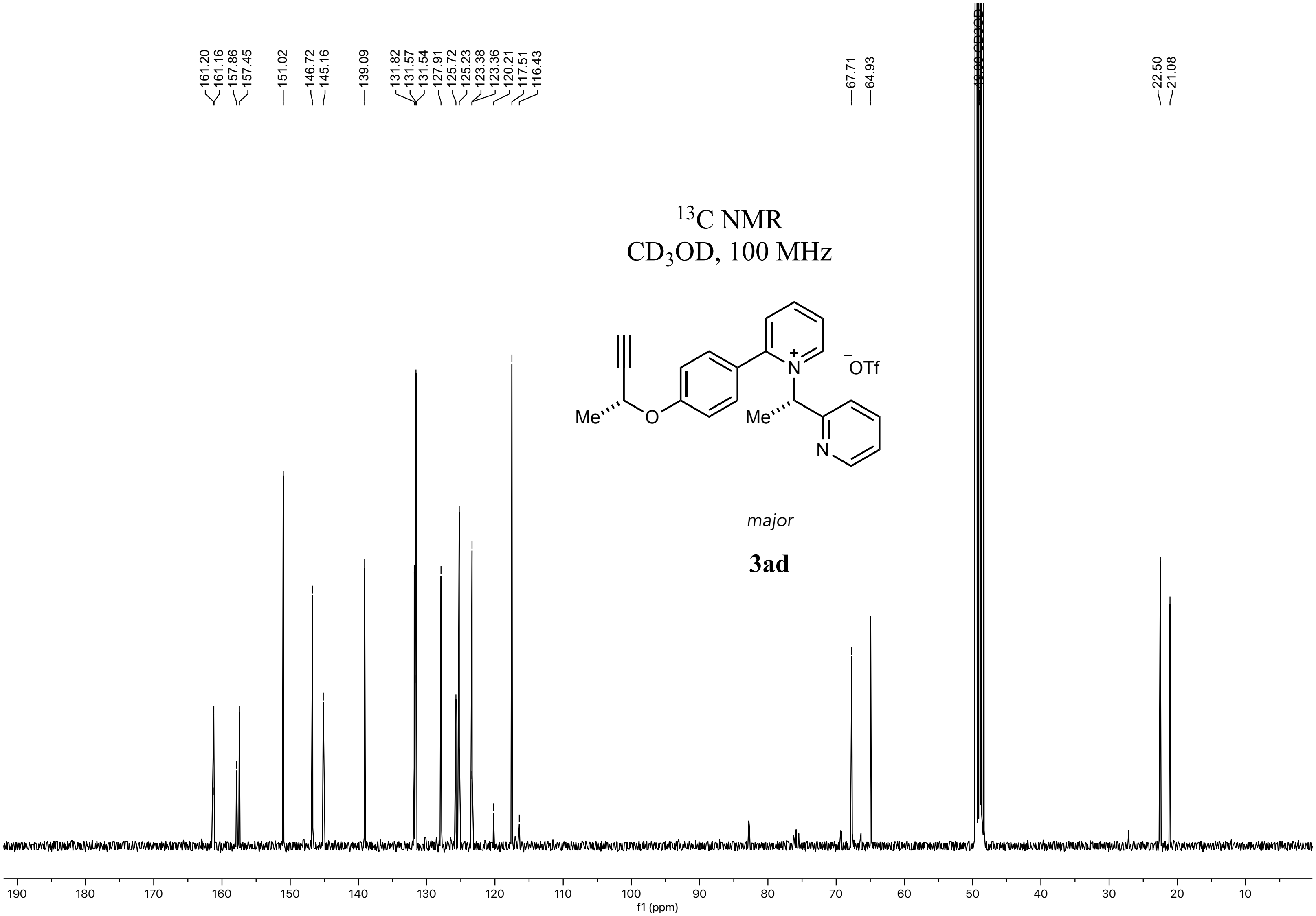
161.20
161.16
157.86
157.45
151.02
146.72
145.16
139.09
131.82
131.57
131.54
127.91
125.72
125.23
123.38
123.36
120.21
117.51
116.43
67.71
64.93
49.00 CD3OD
22.50
21.08
13C NMR
CD3OD, 100 MHz
OTf
Me
O
N
N
major
3ad
f1 (ppm)

^{19}F NMR
CD_3CN, 375 MHz

3ad

25 : 1

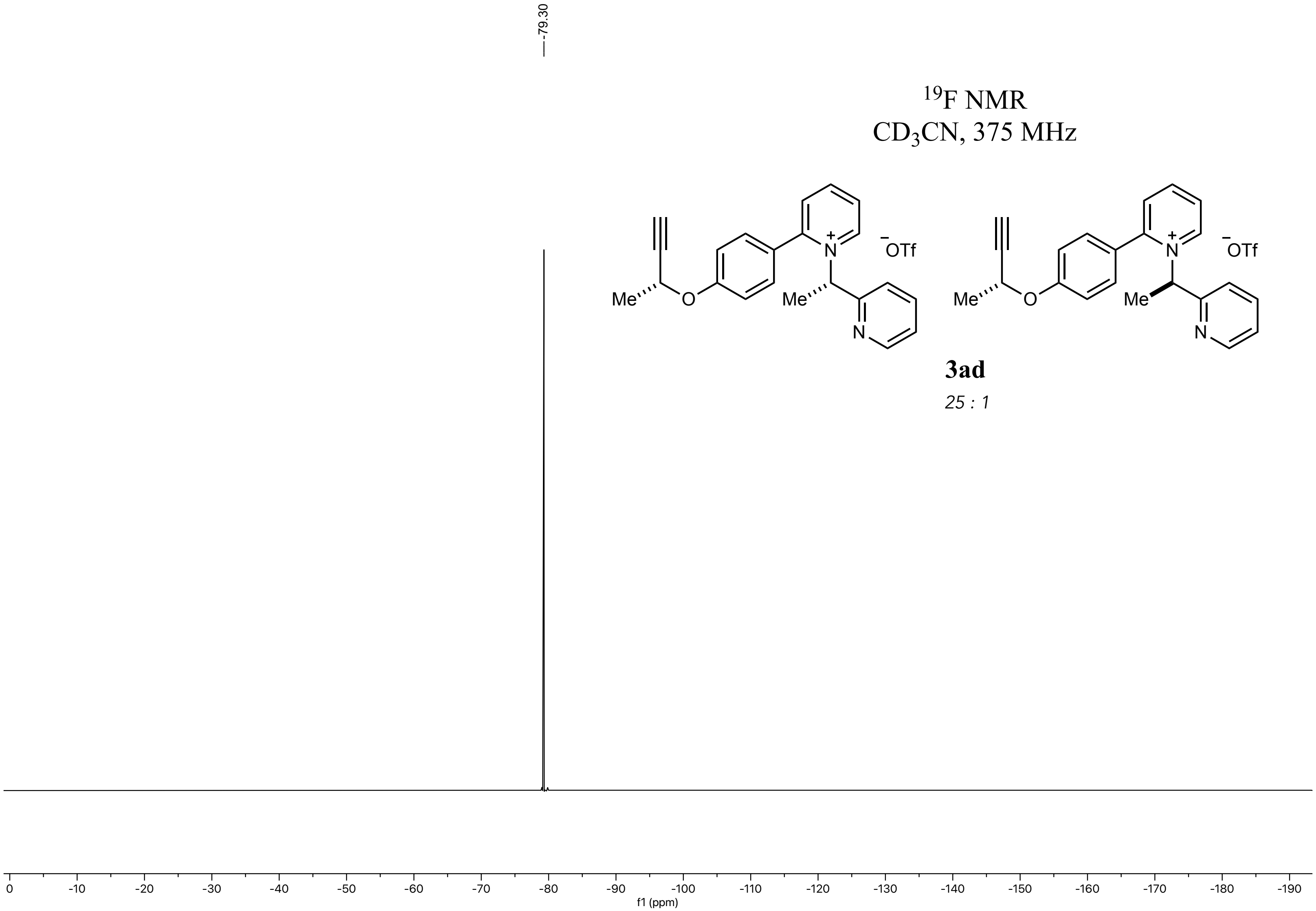

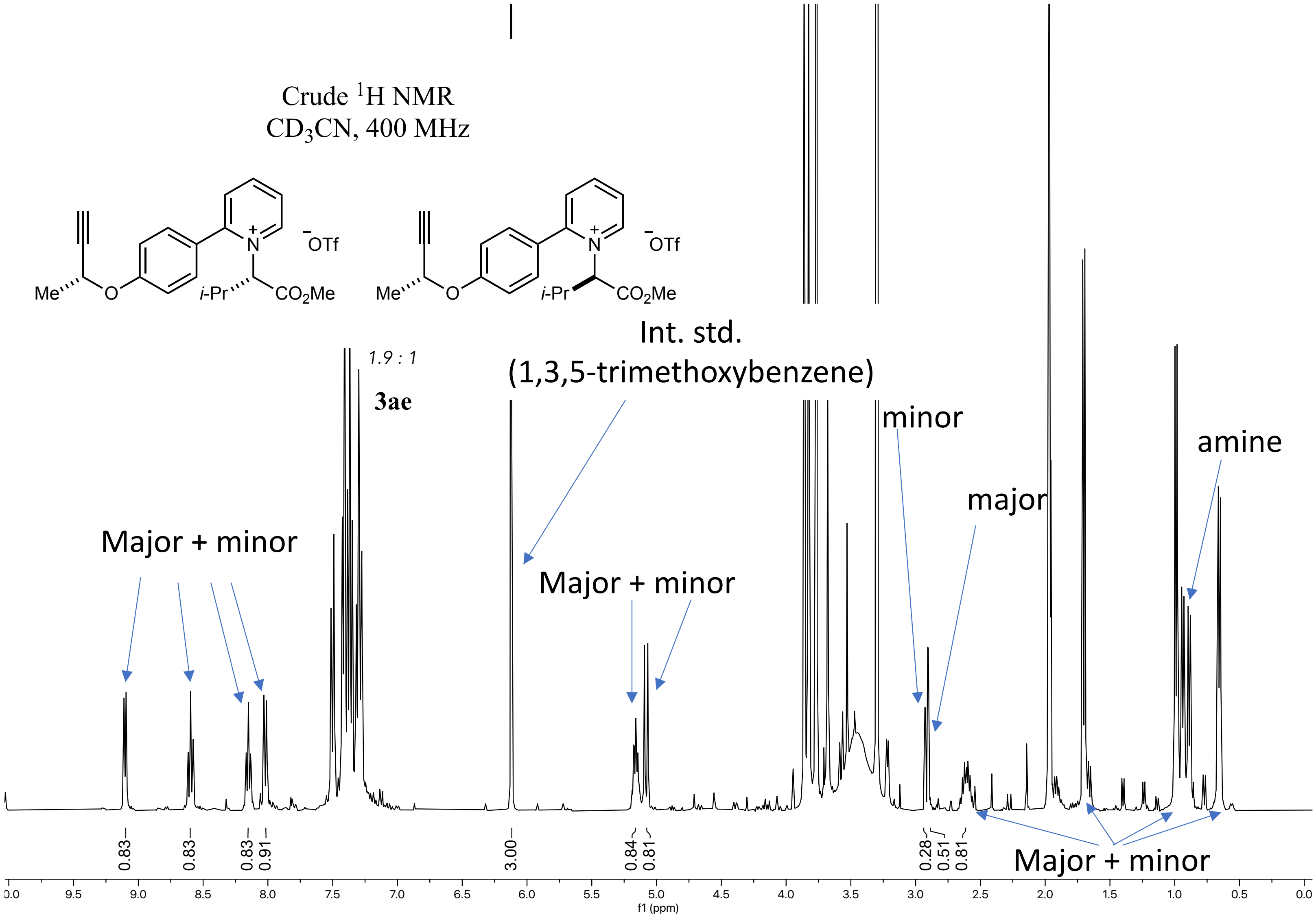
Crude ^{1}H NMR
CD_3CN, 400 MHz
OTf
Me
O
N
i-Pr
CO_2Me
1.9 : 1
3ae
Int. std.
(1,3,5-trimethoxybenzene)
minor
major
amine
Major + minor
Major + minor
Major + minor
0.83
0.83
0.83
0.91
3.00
0.84
0.81
0.28
0.51
0.81
f1 (ppm)

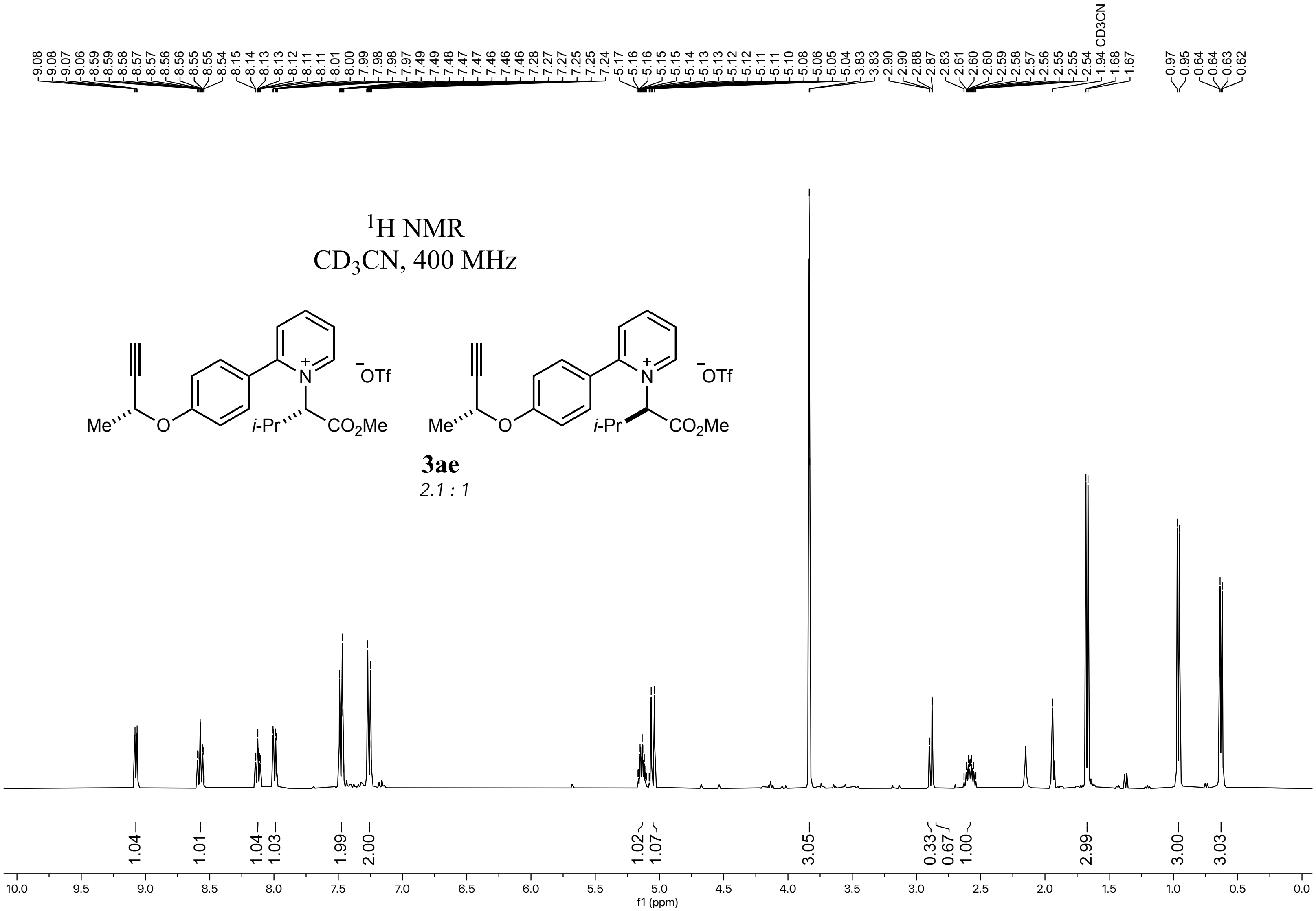

3ae
2.1 : 1

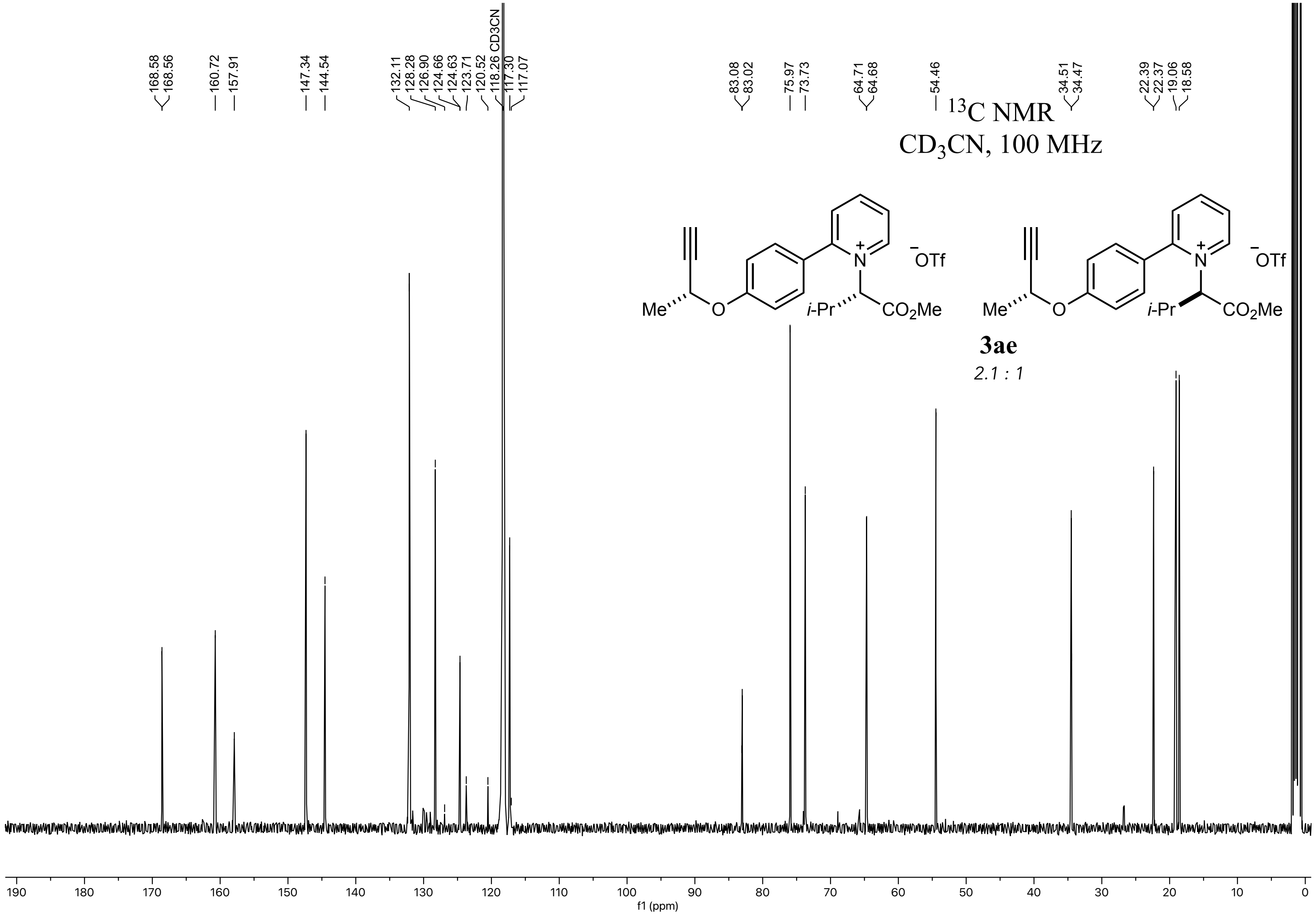
168.58
168.56
160.72
157.91
147.34
144.54
132.11
128.28
126.90
124.66
124.63
123.71
120.52
118.26 CD3CN
117.30
117.07
83.08
83.02
75.97
73.73
64.71
64.68
54.46
34.51
34.47
22.39
22.37
19.06
18.58
^{13}C NMR
CD_3CN, 100 MHz
Me
O
N
OTf
i-Pr
CO_2Me
3ae
2.1 : 1
190 180 170 160 150 140 130 120 110 100 90 80 70 60 50 40 30 20 10 0
f1 (ppm)

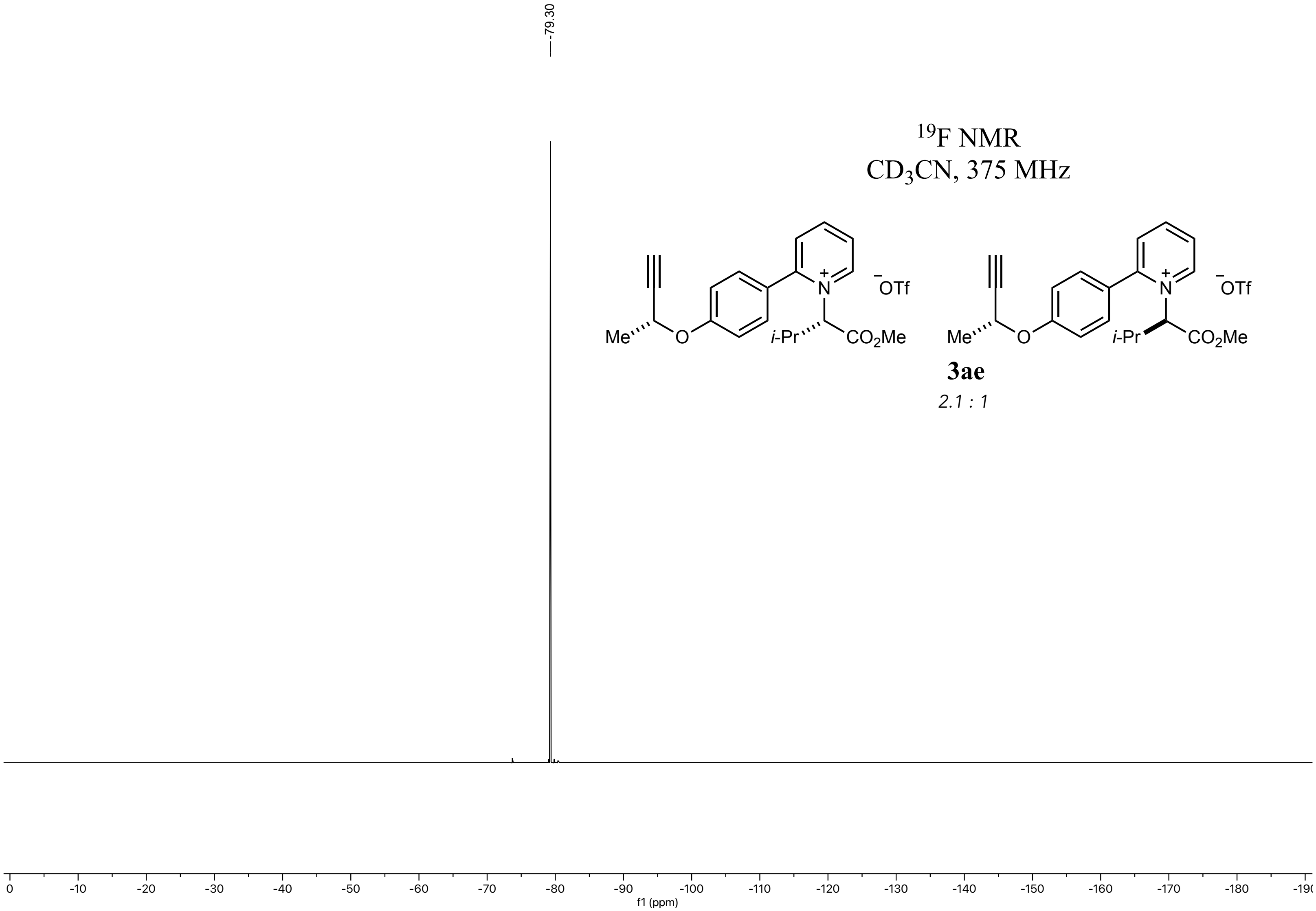

^{19}F NMR
CD_3CN, 375 MHz

3ae

2.1 : 1

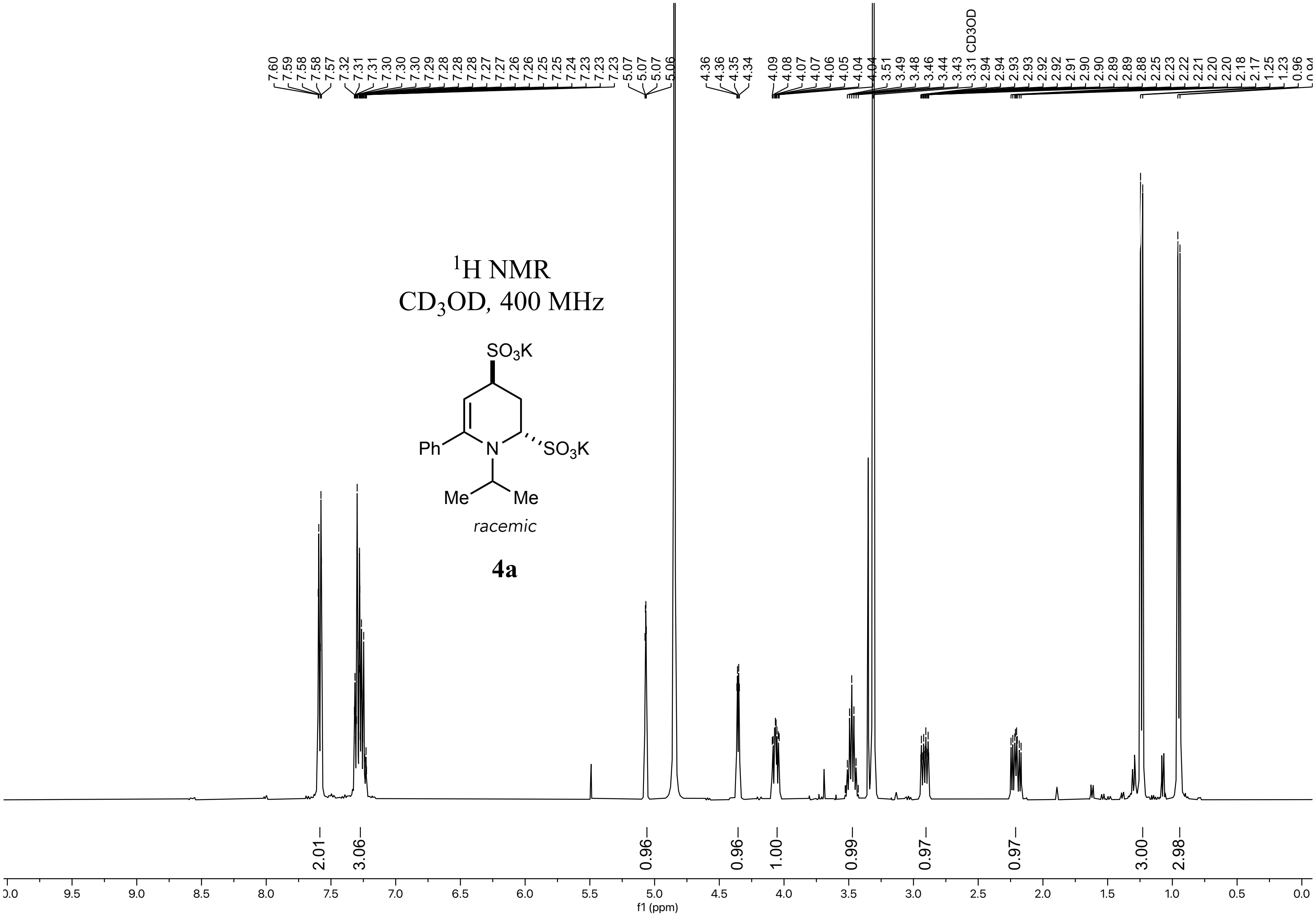

^{1}H NMR
CD_3OD, 400 MHz
SO_3K
Ph
N
SO_3K
Me
Me
racemic
CD3OD
2.01
3.06
0.96
0.96
1.00
0.99
0.97
0.97
3.00
2.98
f1 (ppm)

4a

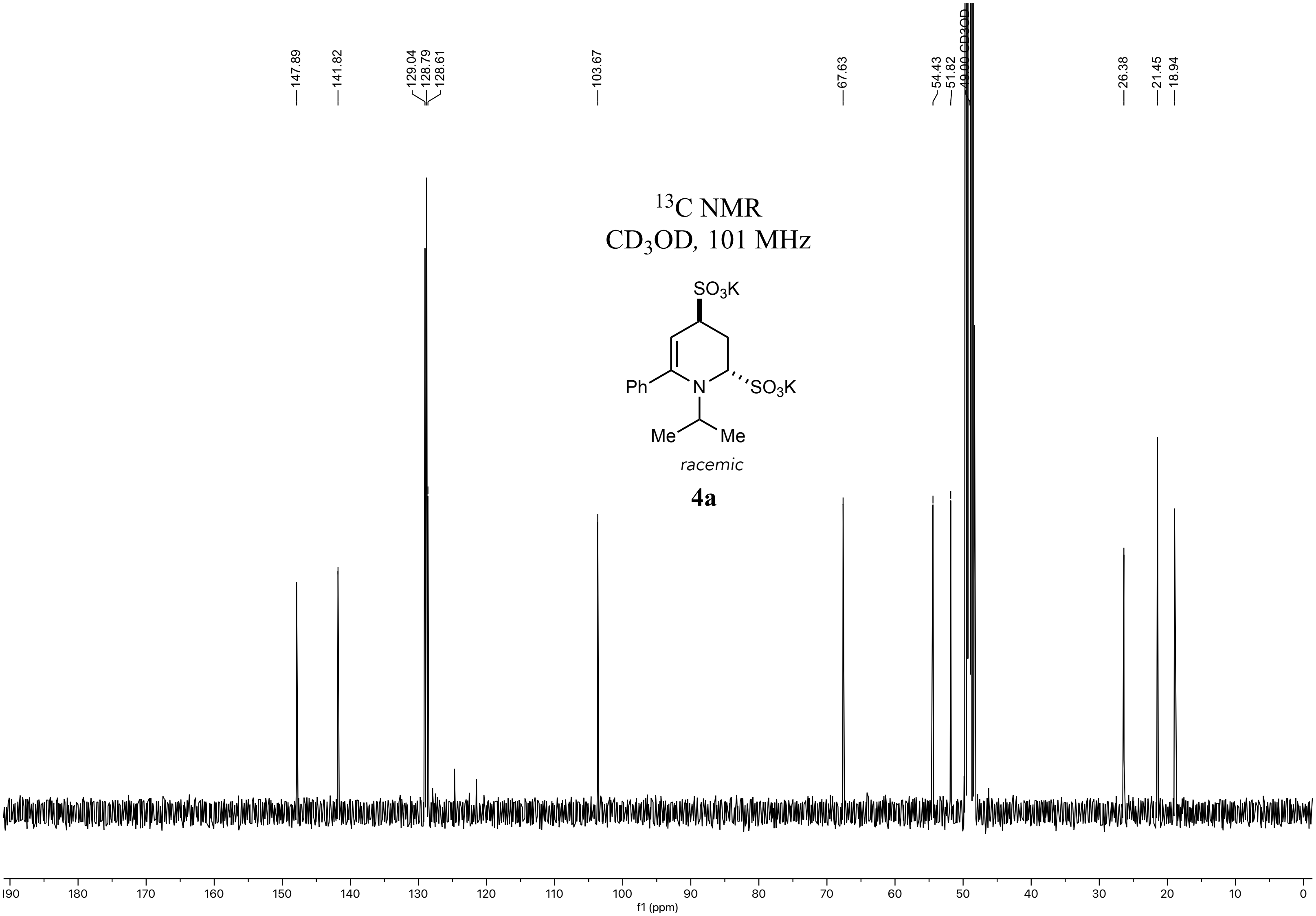

147.89
141.82
129.04
128.79
128.61
103.67
67.63
54.43
51.82
49.00 CD3OD
26.38
21.45
18.94
^{13}C NMR
CD$_3$OD, 101 MHz
SO$_3$K
Ph
N
SO$_3$K
Me
Me
racemic
4a
190
180
170
160
150
140
130
120
110
100
90
80
70
60
50
40
30
20
10
0
f1 (ppm)

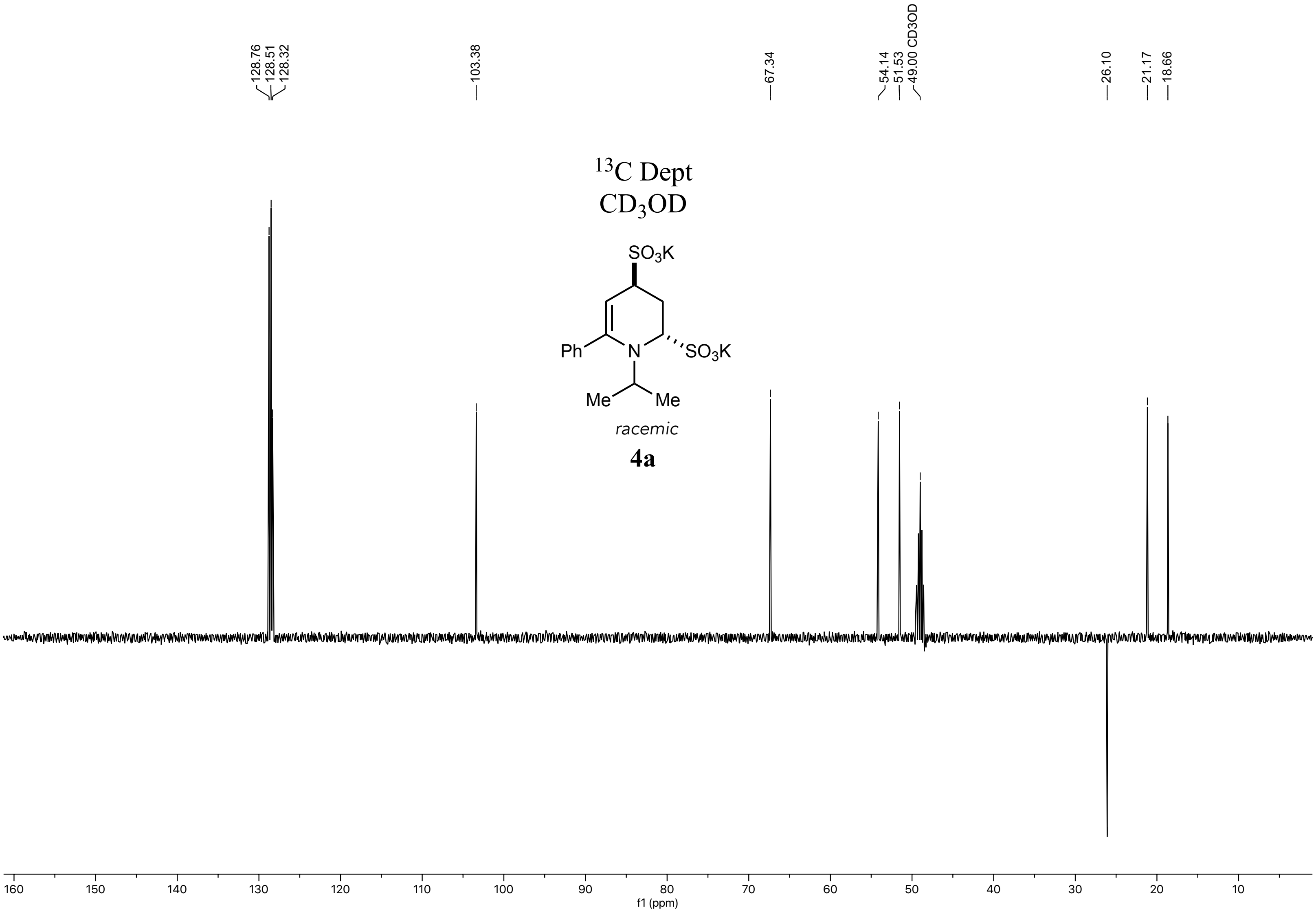
128.76
128.51
128.32
103.38
67.34
54.14
51.53
49.00 CD3OD
26.10
21.17
18.66
^{13}C Dept
CD_3OD
SO_3K
Ph
N
SO_3K
Me
Me
racemic
4a
160
150
140
130
120
110
100
90
80
70
60
50
40
30
20
10
f1 (ppm)

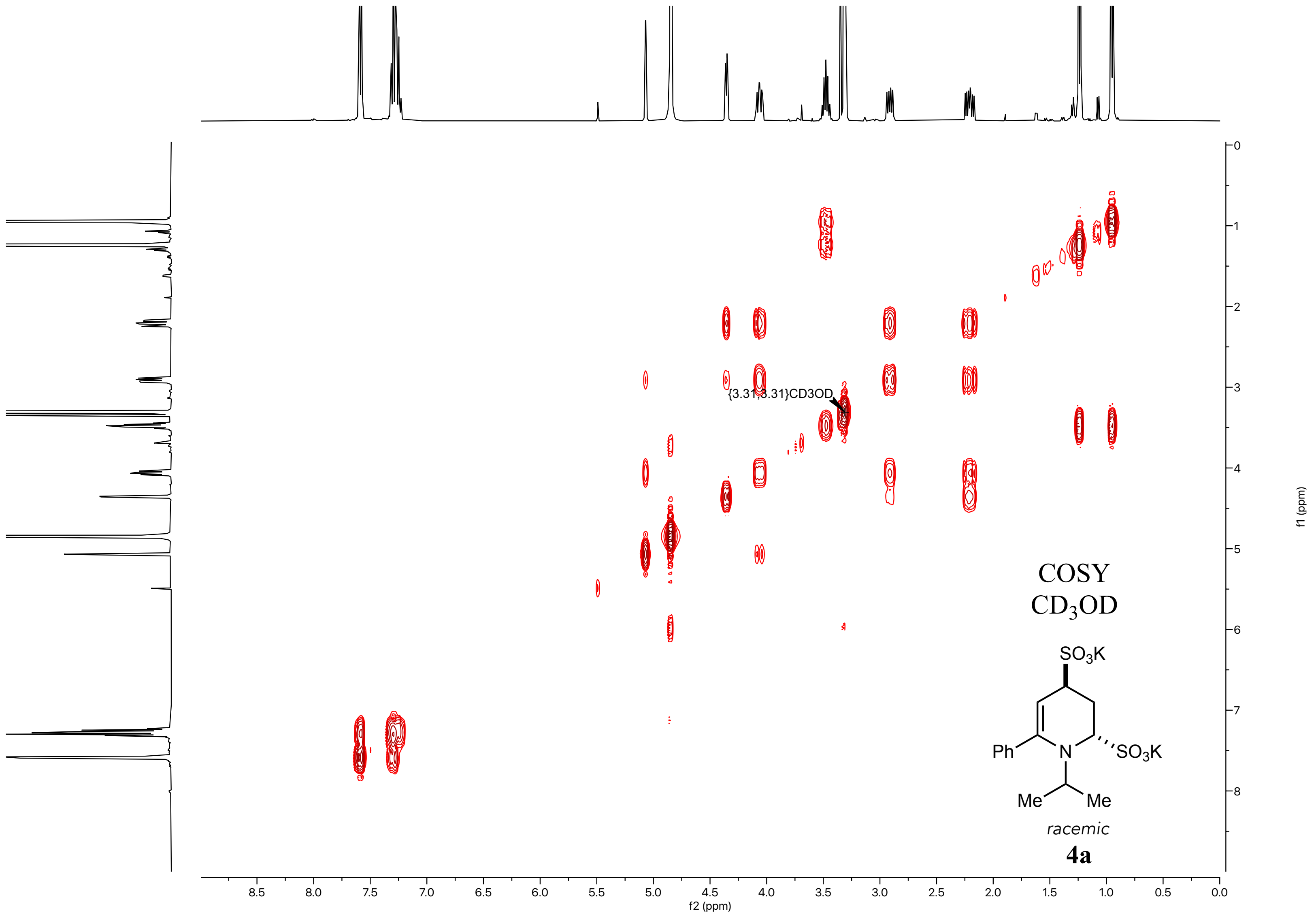

{3.31,3.31}CD3OD
f2 (ppm)
f1 (ppm)
COSY
CD3OD
SO3K
Ph
N
SO3K
Me
Me
racemic
4a

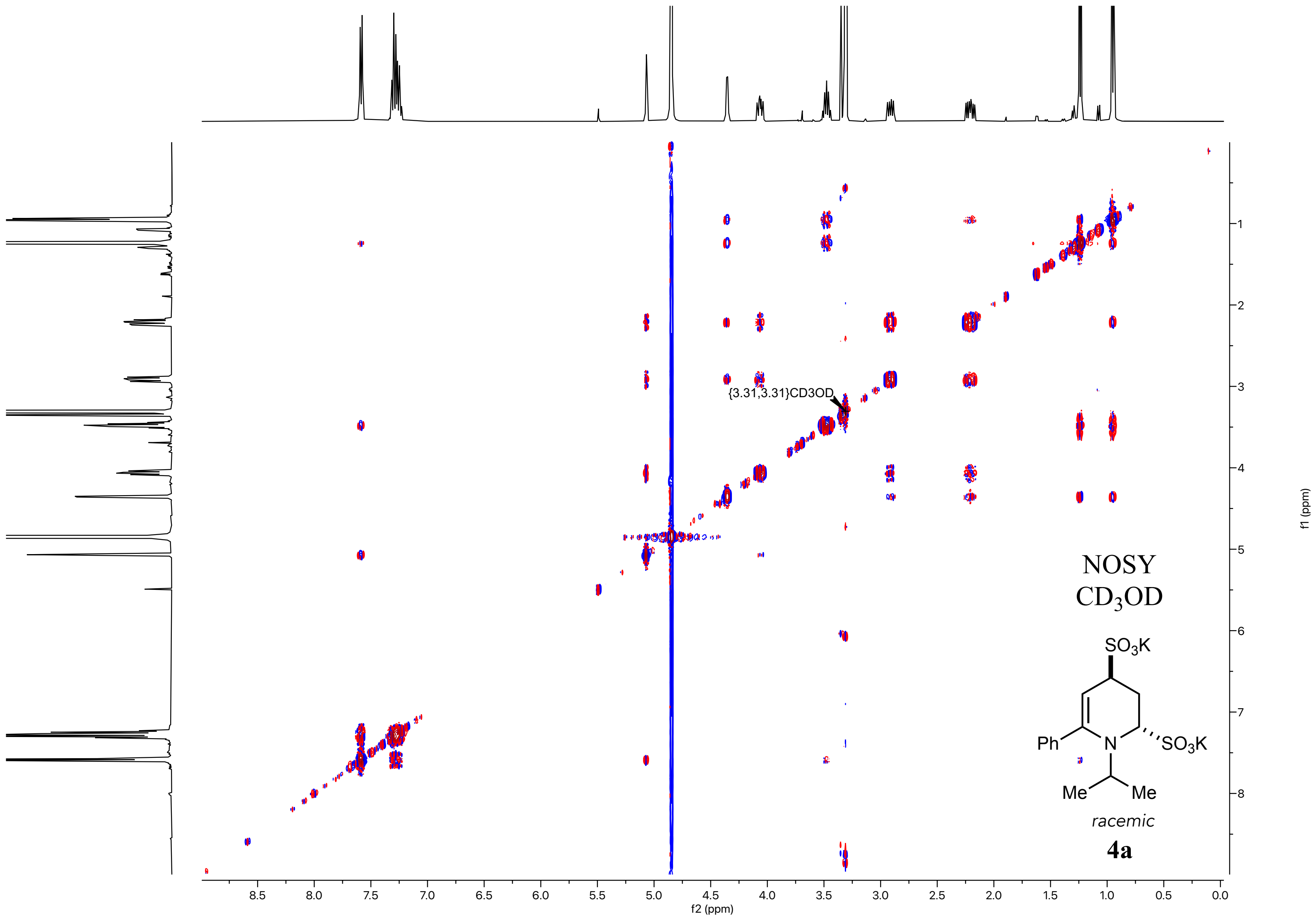

{3.31,3.31}CD3OD
NOSY
CD_3OD
SO_3K
Ph
N
SO_3K
Me
Me
racemic
4a
f2 (ppm)
f1 (ppm)

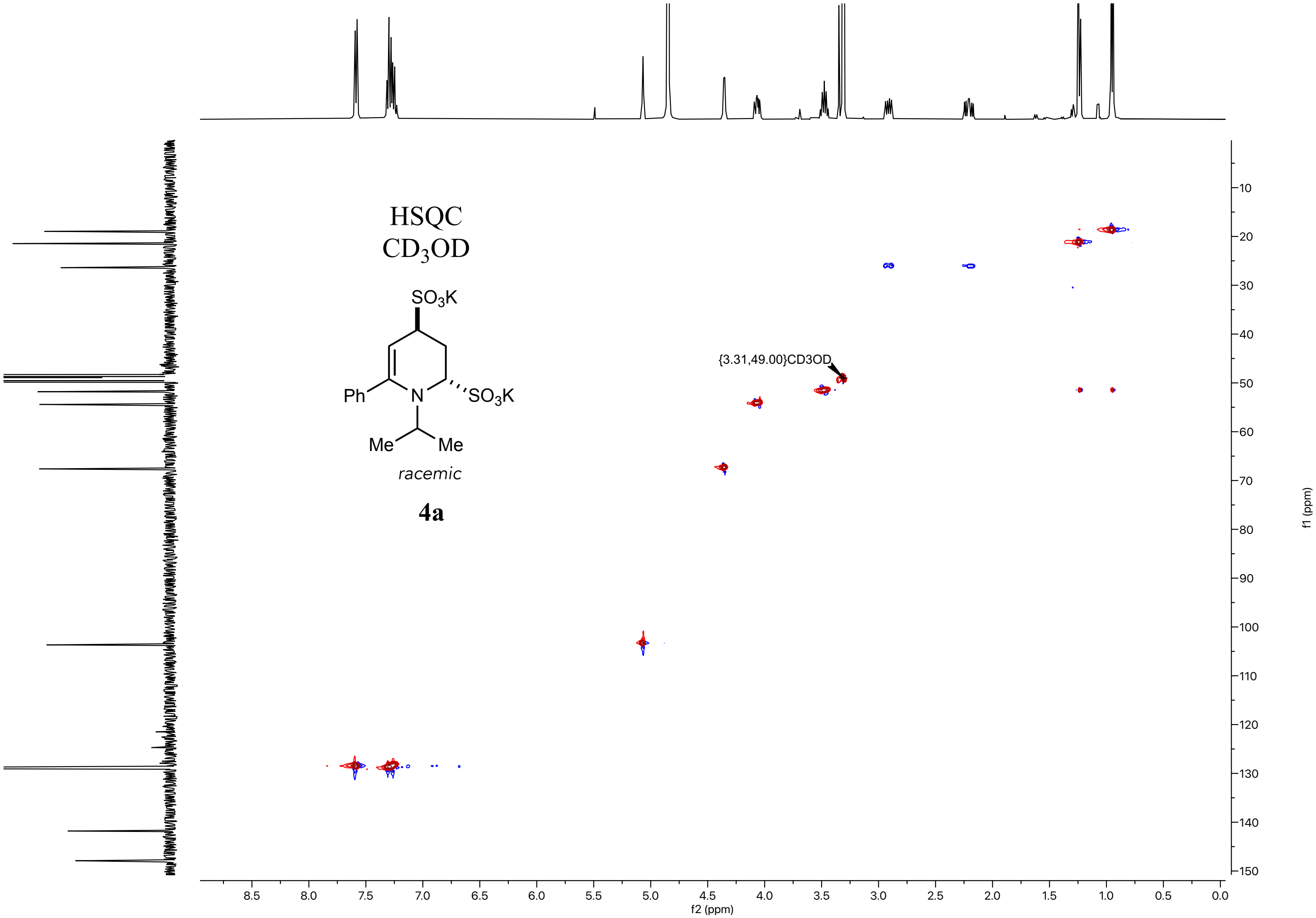

HSQC
CD_3OD
SO_3K
Ph
N
SO_3K
Me
Me
racemic
4a
{3.31,49.00}CD3OD
f2 (ppm)
f1 (ppm)

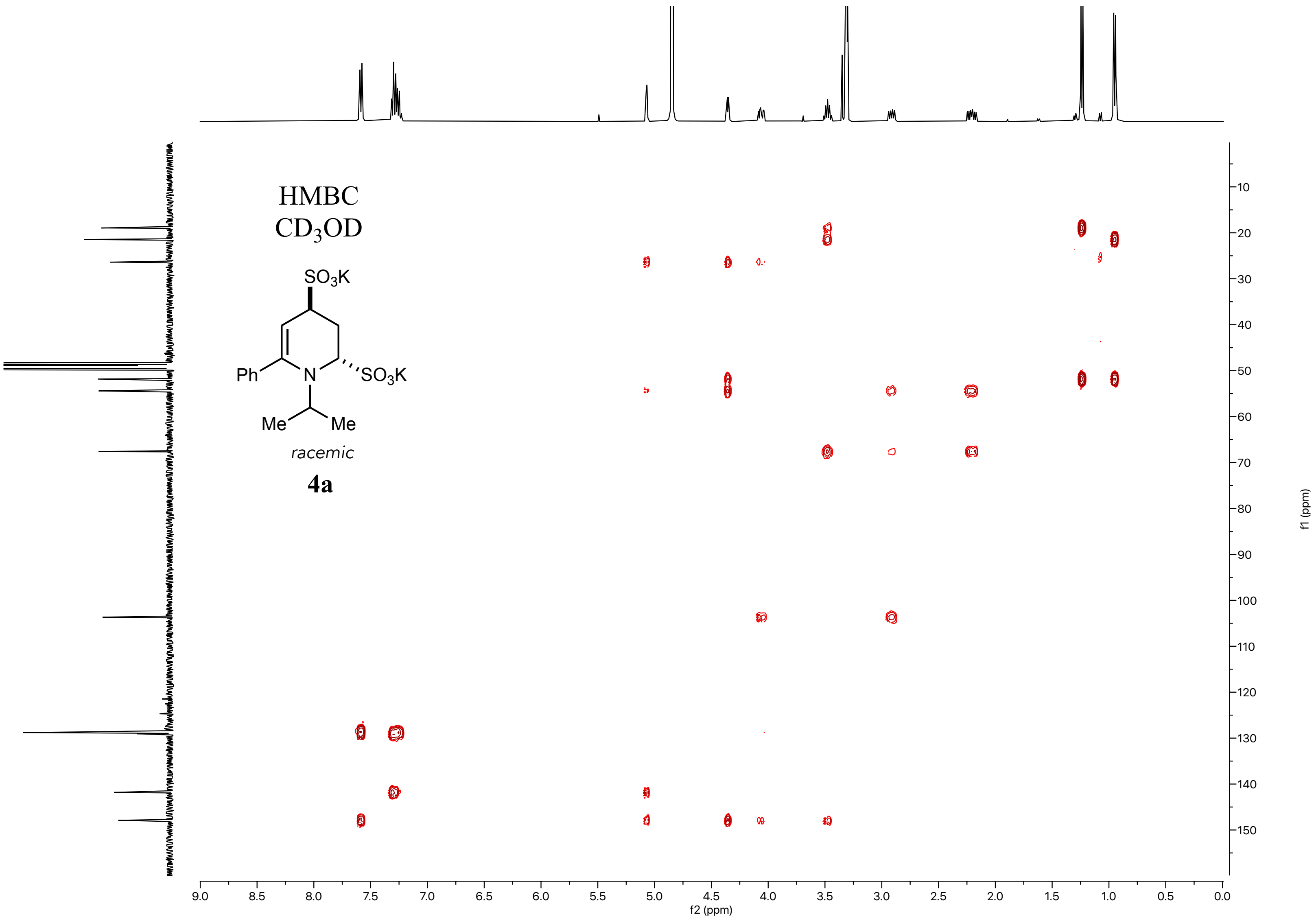
HMBC
CD3OD
SO3K
Ph
N
SO3K
Me
Me
racemic
4a
f2 (ppm)
f1 (ppm)

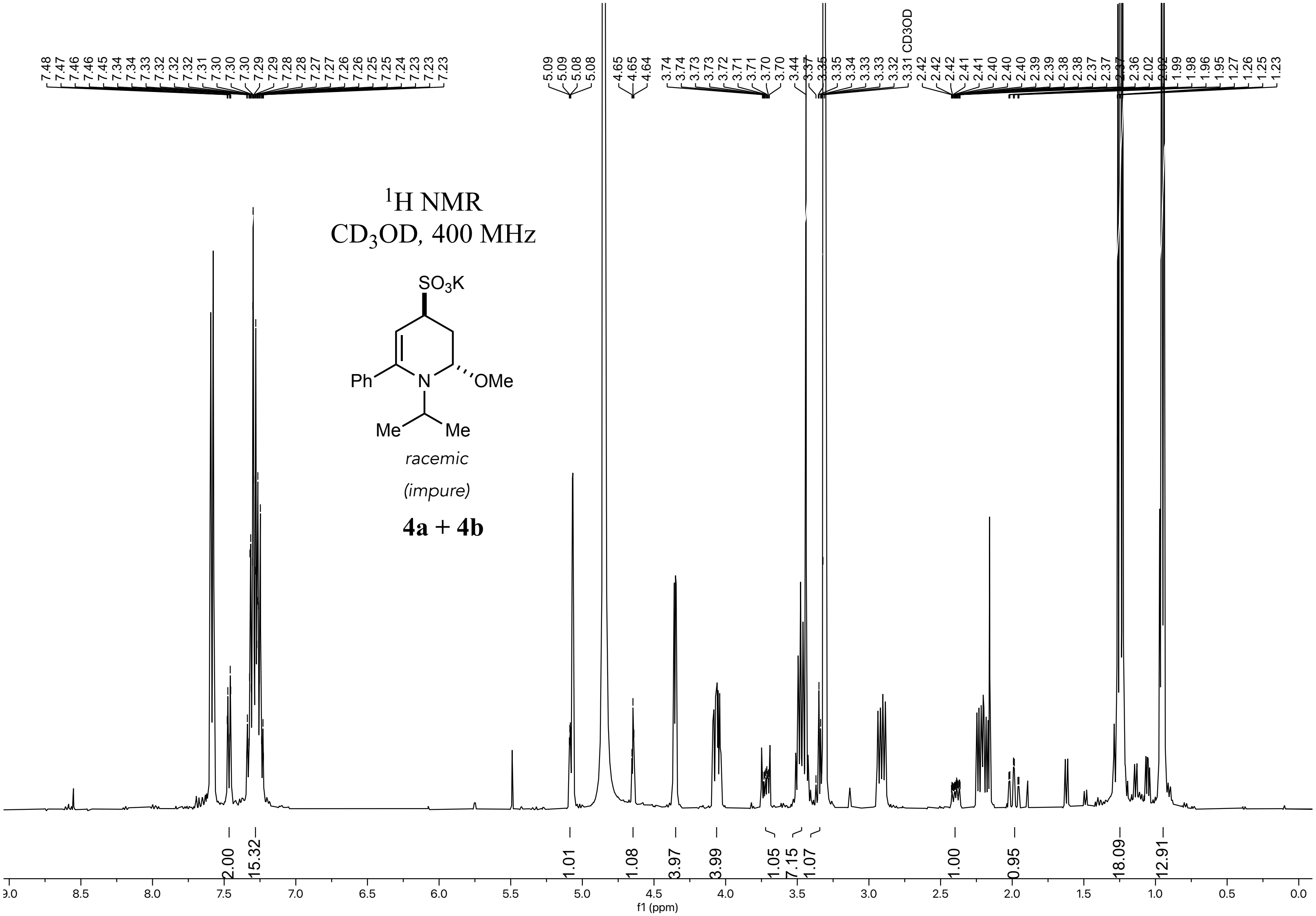

^{1}H NMR
CD_3OD, 400 MHz
SO_3K
Ph
N
OMe
Me
Me
racemic
(impure)
4a + 4b
f1 (ppm)
CD3OD

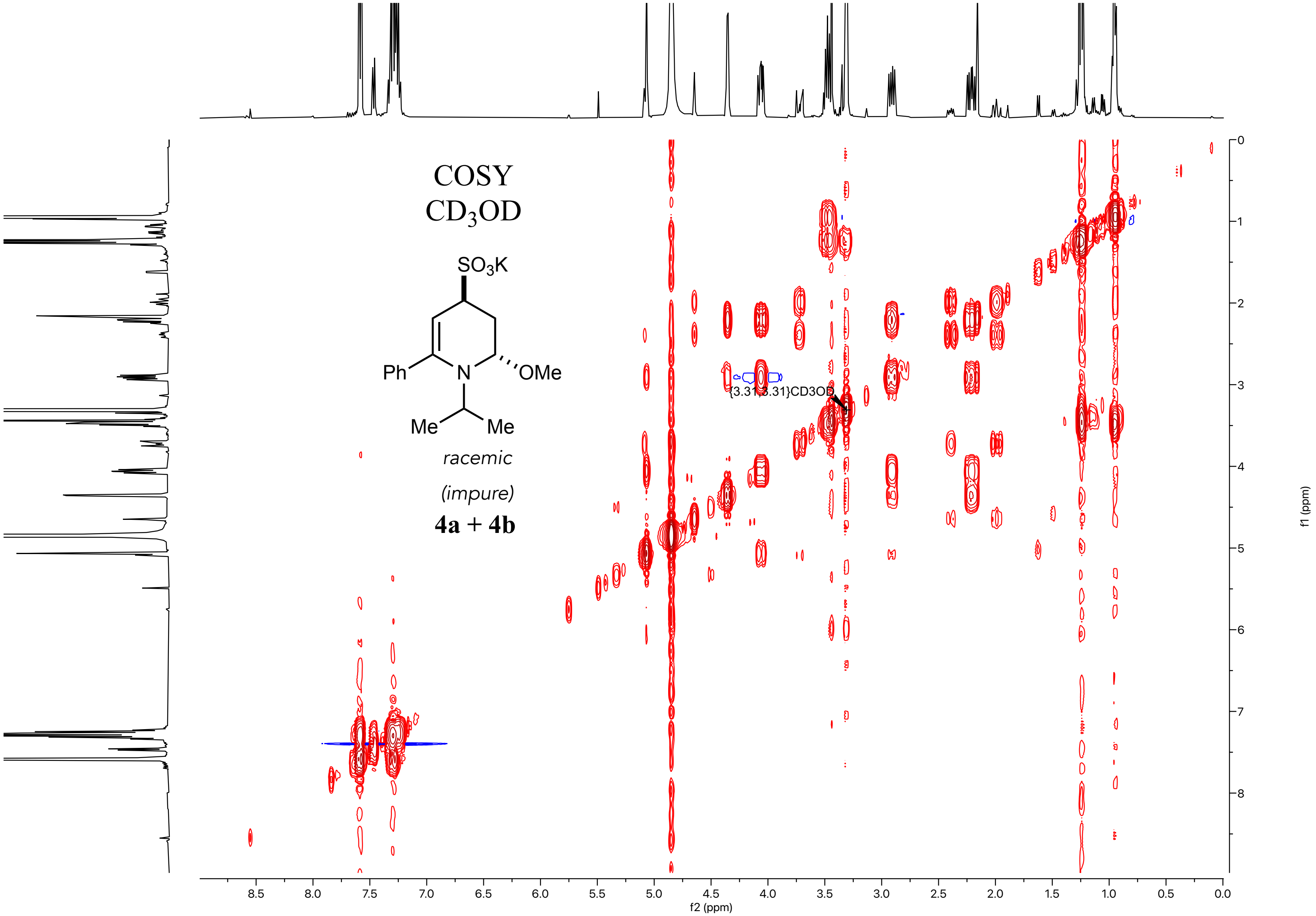
COSY
CD_3OD
SO_3K
Ph
N
OMe
Me
Me
racemic
(impure)
4a + 4b
{3.31,3.31}CD3OD
f2 (ppm)
f1 (ppm)

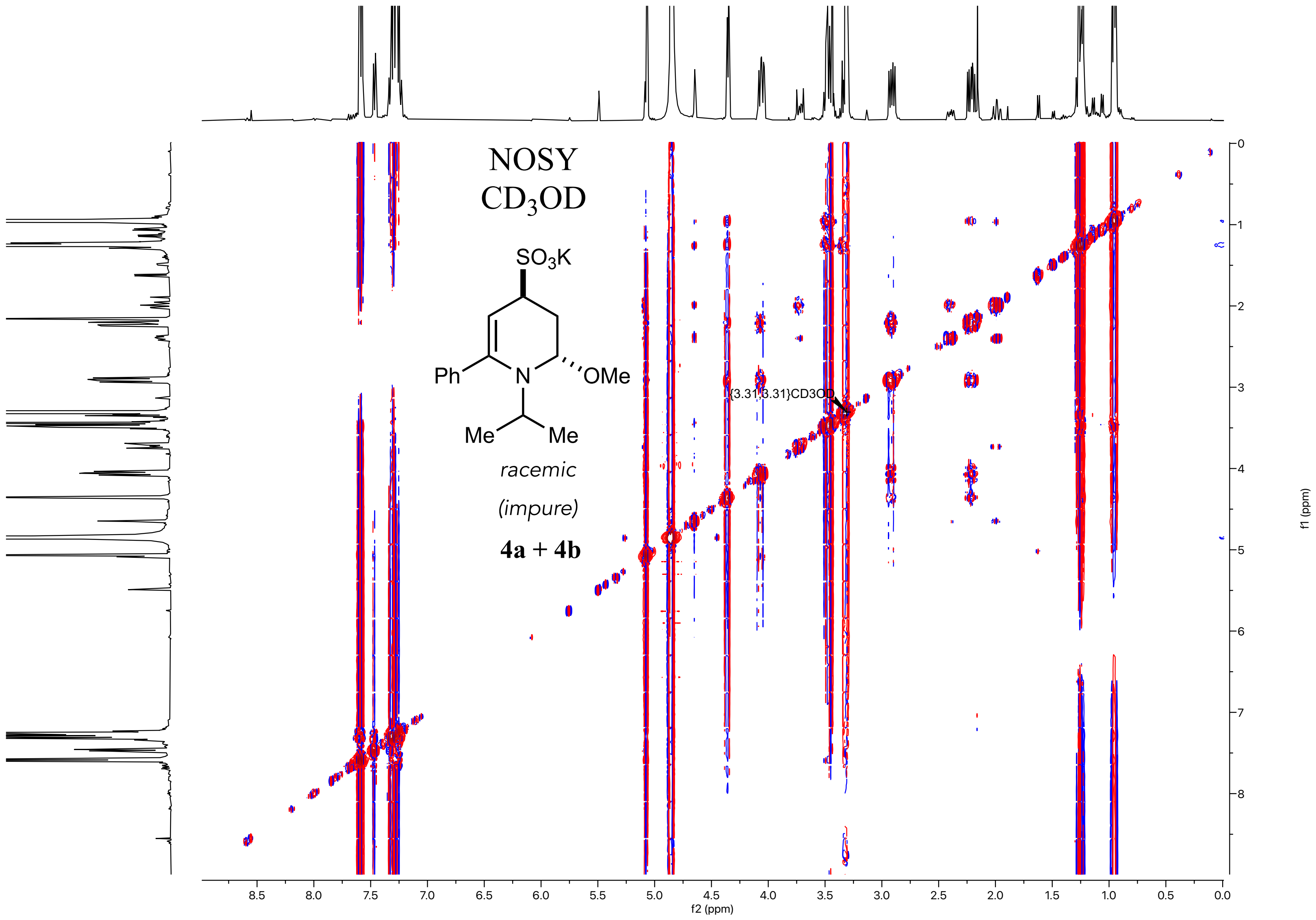
NOSY
CD3OD
SO3K
Ph
N
OMe
Me
Me
racemic
(impure)
4a + 4b
{3.31,3.31}CD3OD
f2 (ppm)
f1 (ppm)

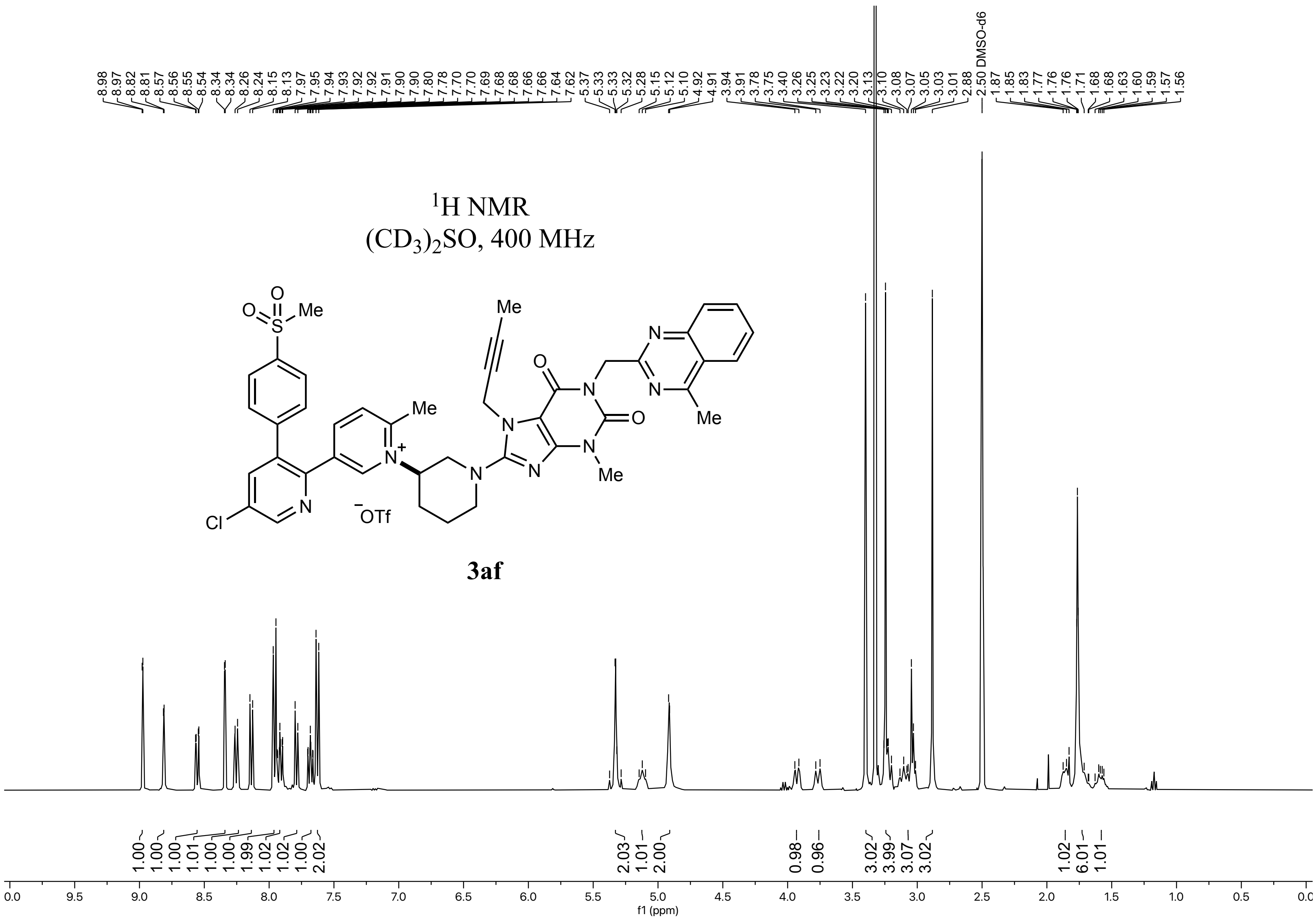

1H NMR
(CD3)2SO, 400 MHz
3af
2.50 DMSO-d6
f1 (ppm)

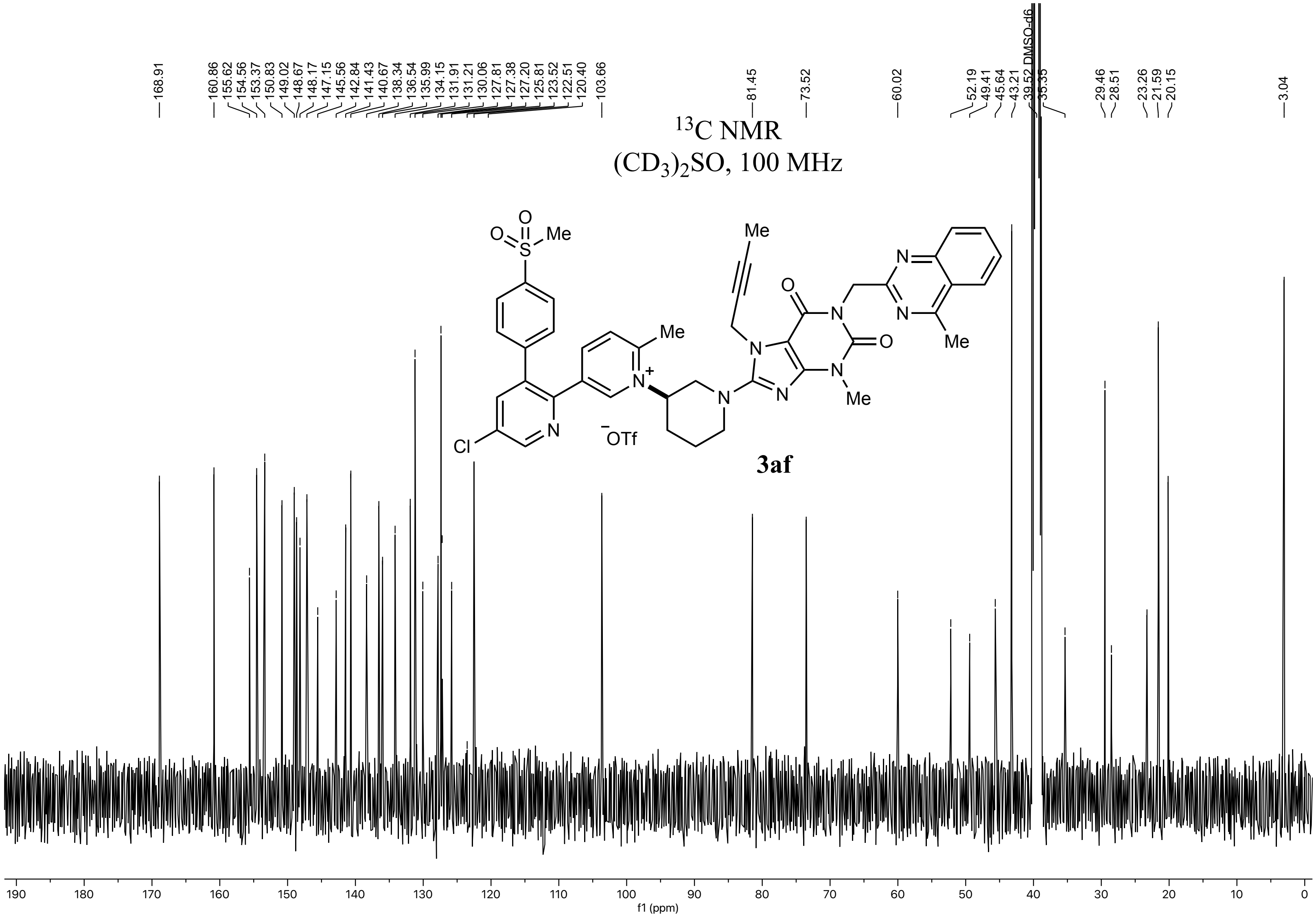
168.91
160.86
155.62
154.56
153.37
150.83
149.02
148.67
148.17
147.15
145.56
142.84
141.43
140.67
138.34
136.54
135.99
134.15
131.91
131.21
130.06
127.81
127.38
127.20
125.81
123.52
122.51
120.40
103.66
81.45
73.52
60.02
52.19
49.41
45.64
43.21
39.52 DMSO-d6
35.35
29.46
28.51
23.26
21.59
20.15
3.04
^{13}C NMR
$(CD_3)_2SO$, 100 MHz
3af
f1 (ppm)

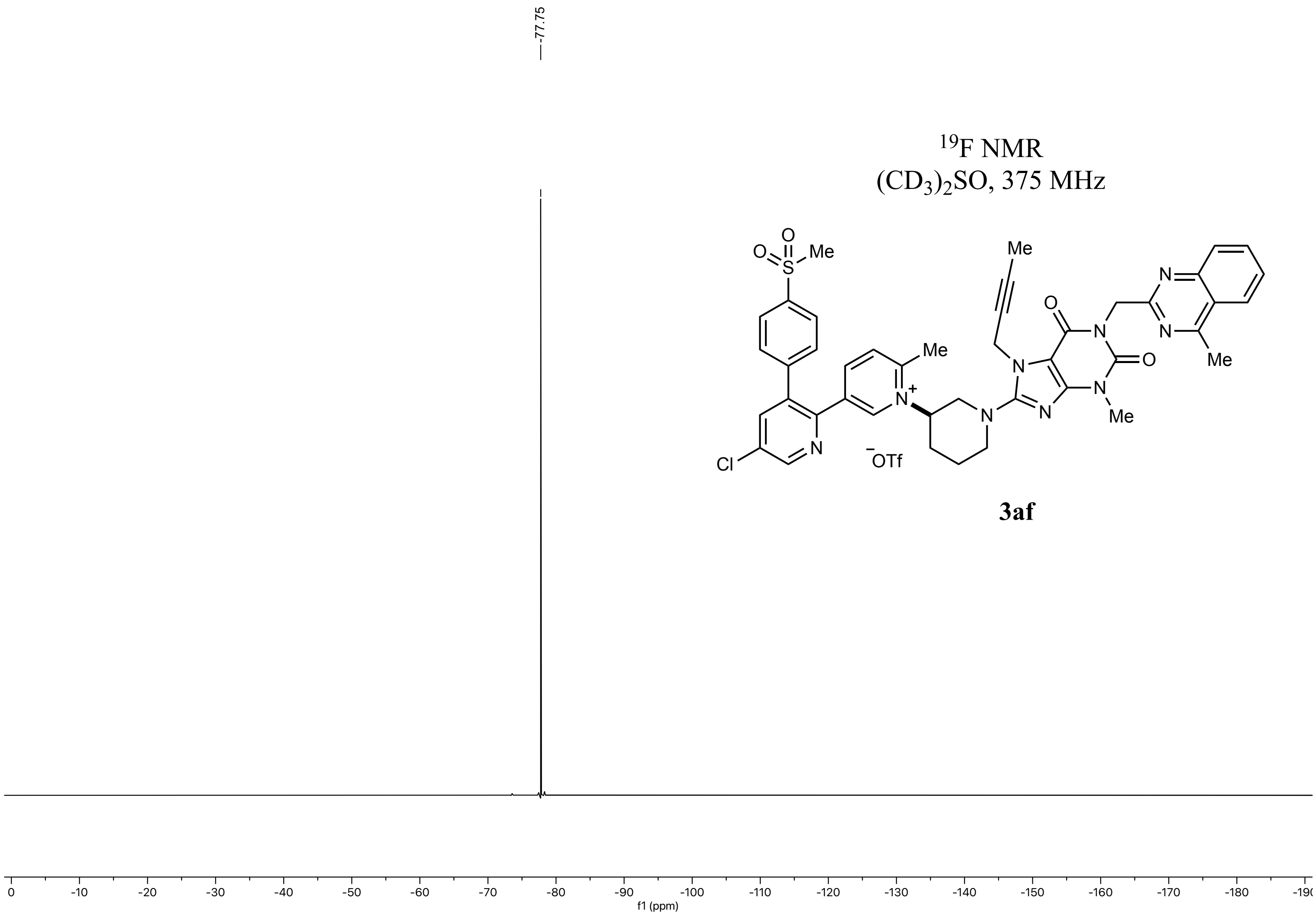

-77.75
^{19}F NMR
$(CD_3)_2SO$, 375 MHz
O
O
S
Me
Me
Me
N
N
O
N
O
Me
N
N
Me
N
N
N
N
Cl
N
$^{-}$OTf
3af
0
-10
-20
-30
-40
-50
-60
-70
-80
-90
-100
-110
-120
-130
-140
-150
-160
-170
-180
-190
f1 (ppm)

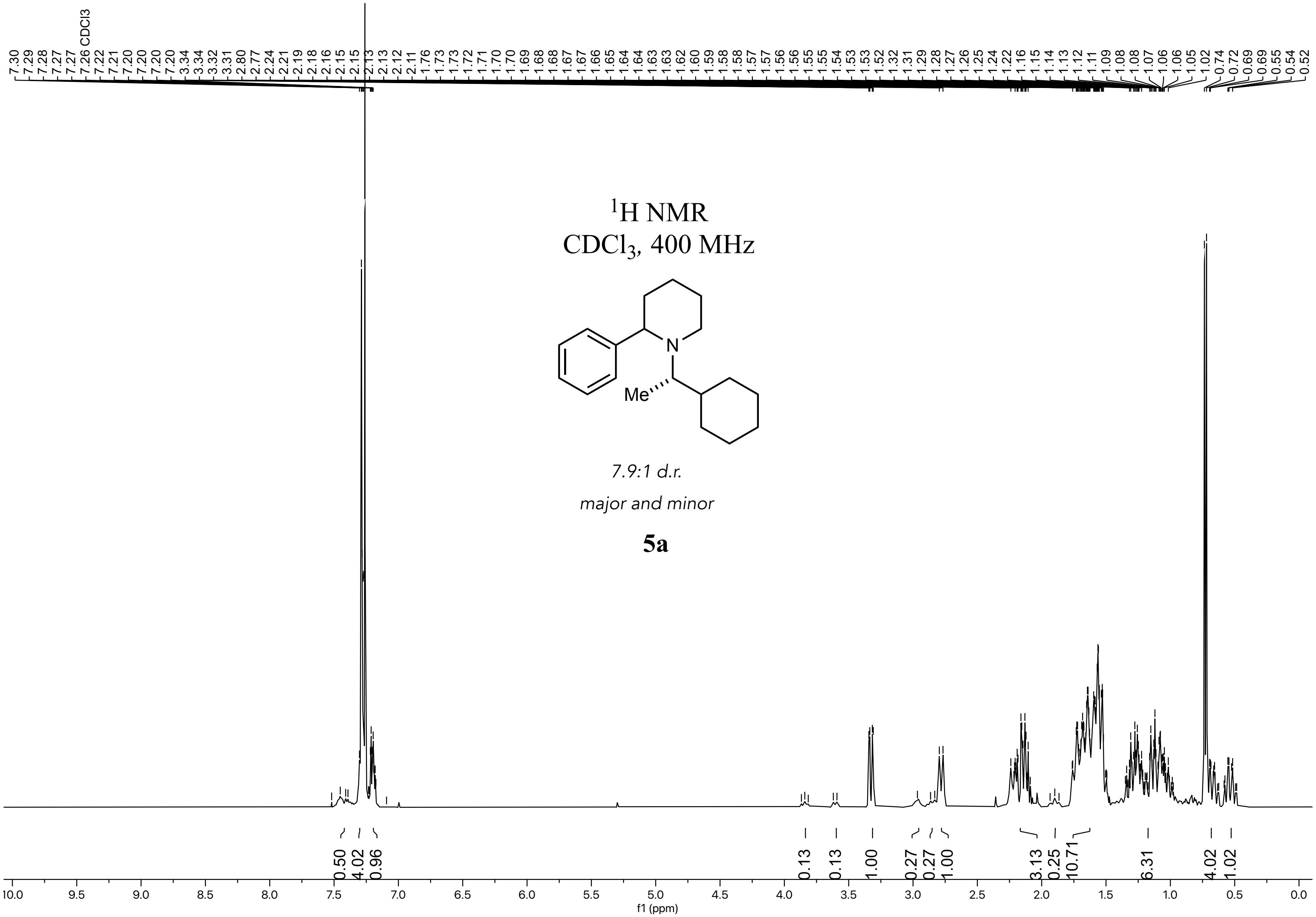

5a

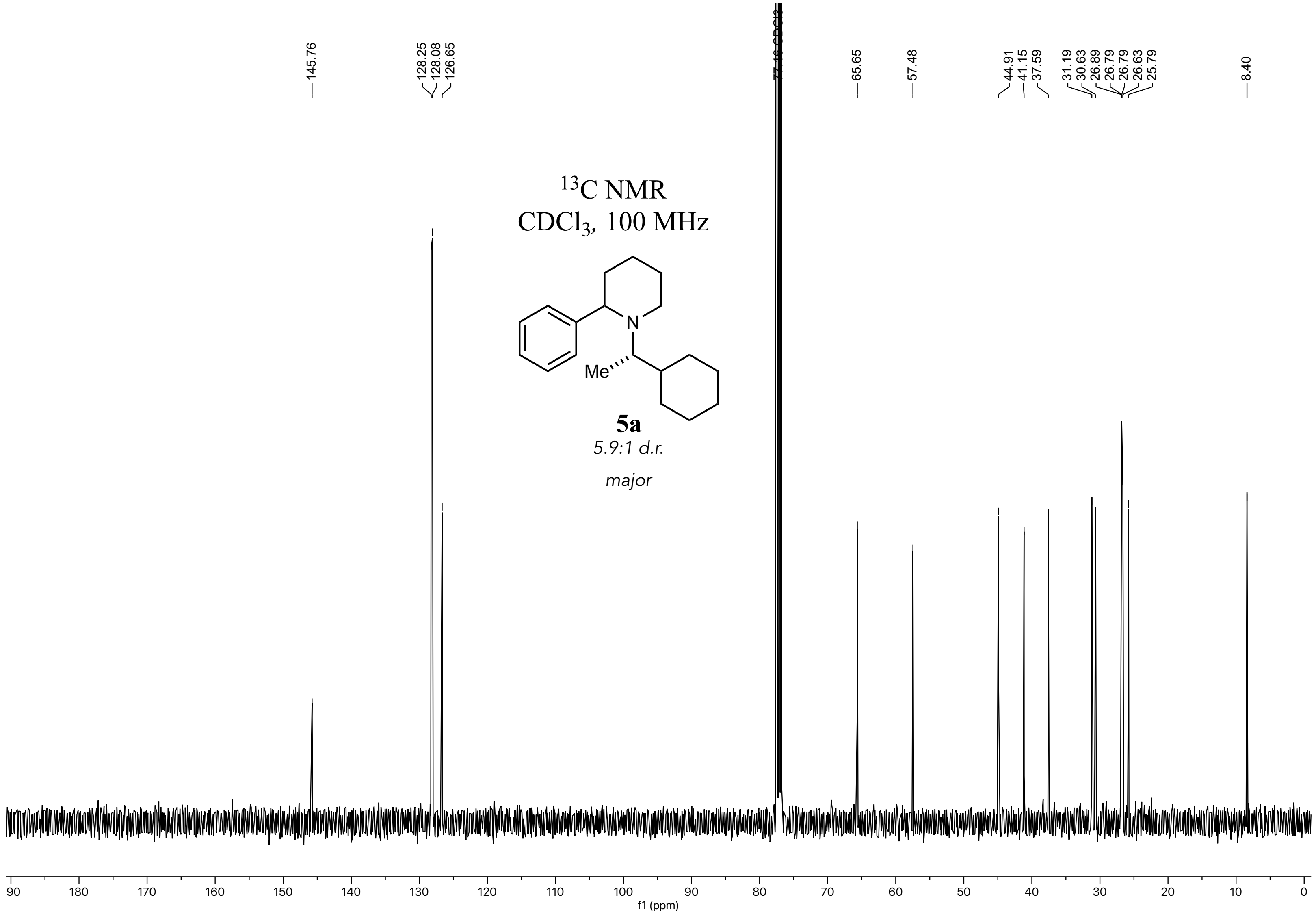
145.76
128.25
128.08
126.65
77.16 CDCl3
65.65
57.48
44.91
41.15
37.59
31.19
30.63
26.89
26.79
26.79
26.63
25.79
8.40
13C NMR
CDCl3, 100 MHz
N
Me
5a
5.9:1 d.r.
major
f1 (ppm)

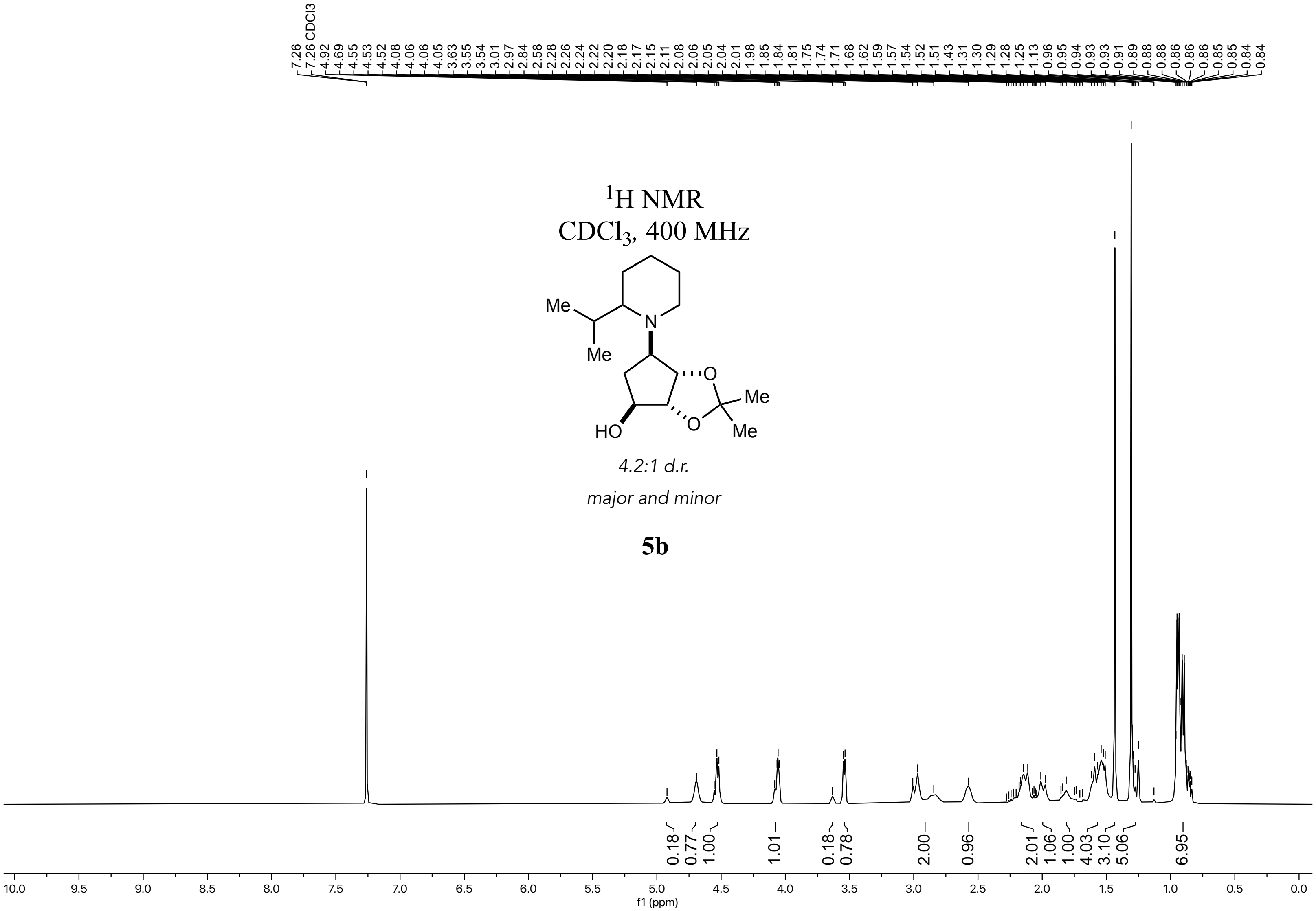

1H NMR
CDCl3, 400 MHz
Me
Me
N
O
O
Me
Me
HO
4.2:1 d.r.
major and minor
5b
f1 (ppm)

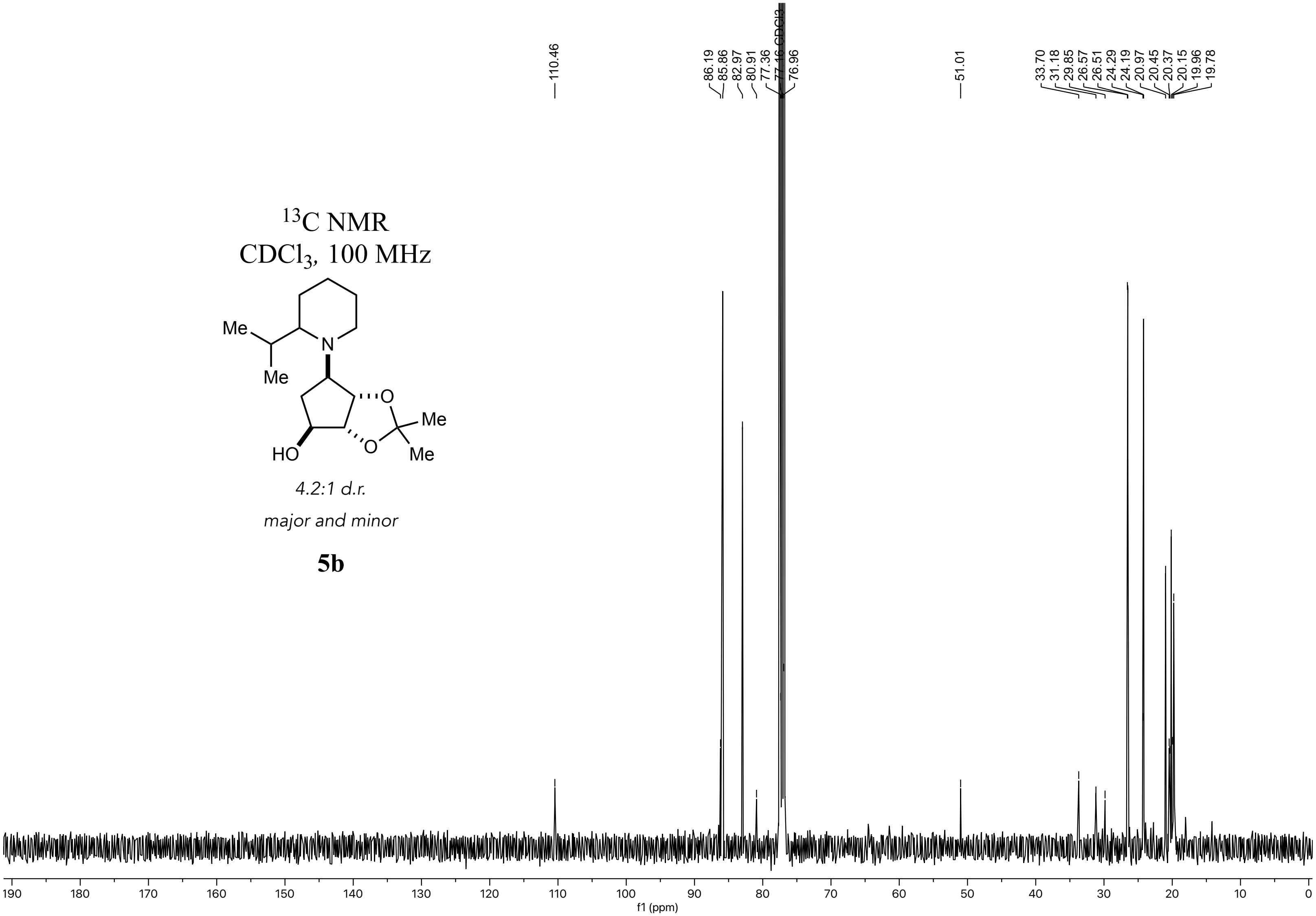

^{13}C NMR
$CDCl_3$, 100 MHz

4.2:1 d.r.

major and minor

5b

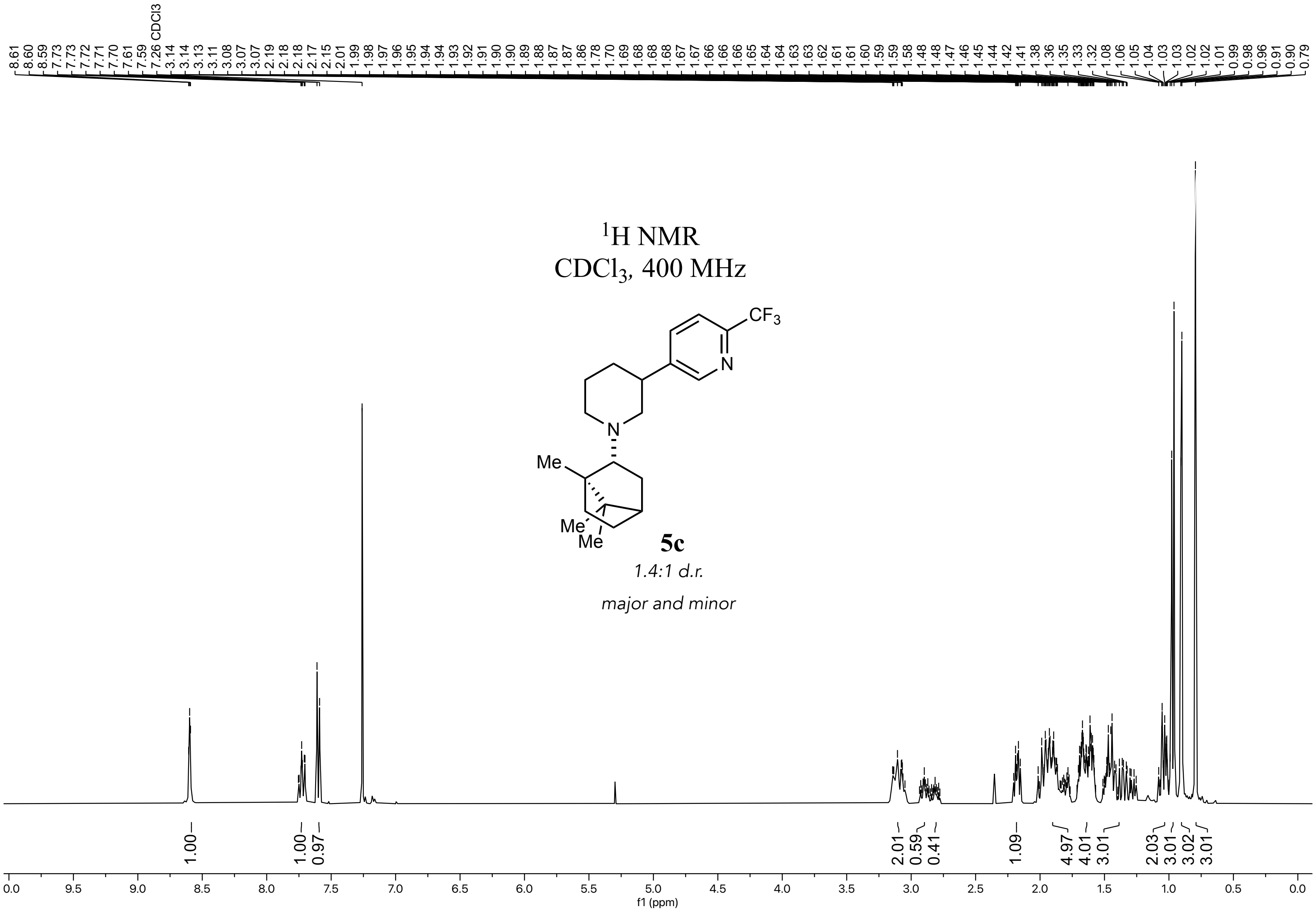
8.61 8.60 8.59 7.73 7.73 7.72 7.71 7.70 7.61 7.59 7.26 CDCl3 3.14 3.14 3.13 3.11 3.08 3.07 3.07 2.19 2.18 2.17 2.15 2.01 1.99 1.98 1.97 1.96 1.95 1.94 1.93 1.92 1.91 1.90 1.89 1.88 1.87 1.87 1.86 1.78 1.70 1.69 1.68 1.68 1.68 1.67 1.67 1.66 1.66 1.66 1.65 1.64 1.64 1.63 1.63 1.62 1.61 1.61 1.60 1.59 1.59 1.58 1.48 1.48 1.47 1.46 1.45 1.44 1.42 1.41 1.38 1.36 1.35 1.33 1.32 1.08 1.06 1.05 1.04 1.03 1.03 1.02 1.02 1.01 0.99 0.98 0.96 0.91 0.90 0.79
1H NMR
CDCl3, 400 MHz
CF3
N
N
Me
Me
Me
5c
1.4:1 d.r.
major and minor
1.00 1.00 0.97 2.01 0.59 0.41 1.09 4.97 4.01 3.01 2.03 3.01 3.02 3.01
0.0 9.5 9.0 8.5 8.0 7.5 7.0 6.5 6.0 5.5 5.0 4.5 4.0 3.5 3.0 2.5 2.0 1.5 1.0 0.5 0.0
f1 (ppm)

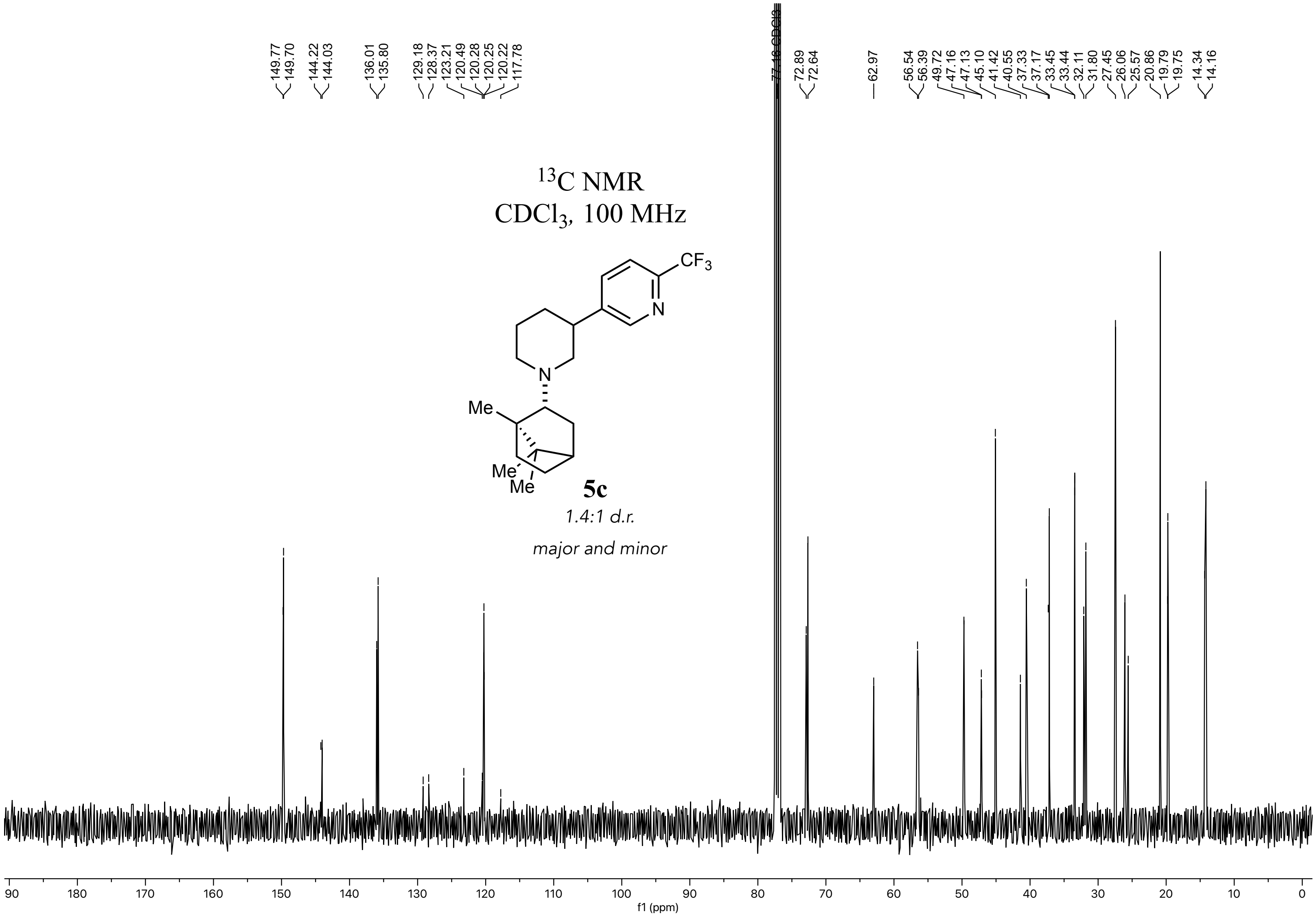

13C NMR
CDCl3, 100 MHz
CF3
N
Me
Me
Me
5c
1.4:1 d.r.
major and minor
149.77
149.70
144.22
144.03
136.01
135.80
129.18
128.37
123.21
120.49
120.28
120.25
120.22
117.78
77.16 CDCl3
72.89
72.64
62.97
56.54
56.39
49.72
47.16
47.13
45.10
41.42
40.55
37.33
37.17
33.45
33.44
32.11
31.80
27.45
26.06
25.57
20.86
19.79
19.75
14.34
14.16
f1 (ppm)

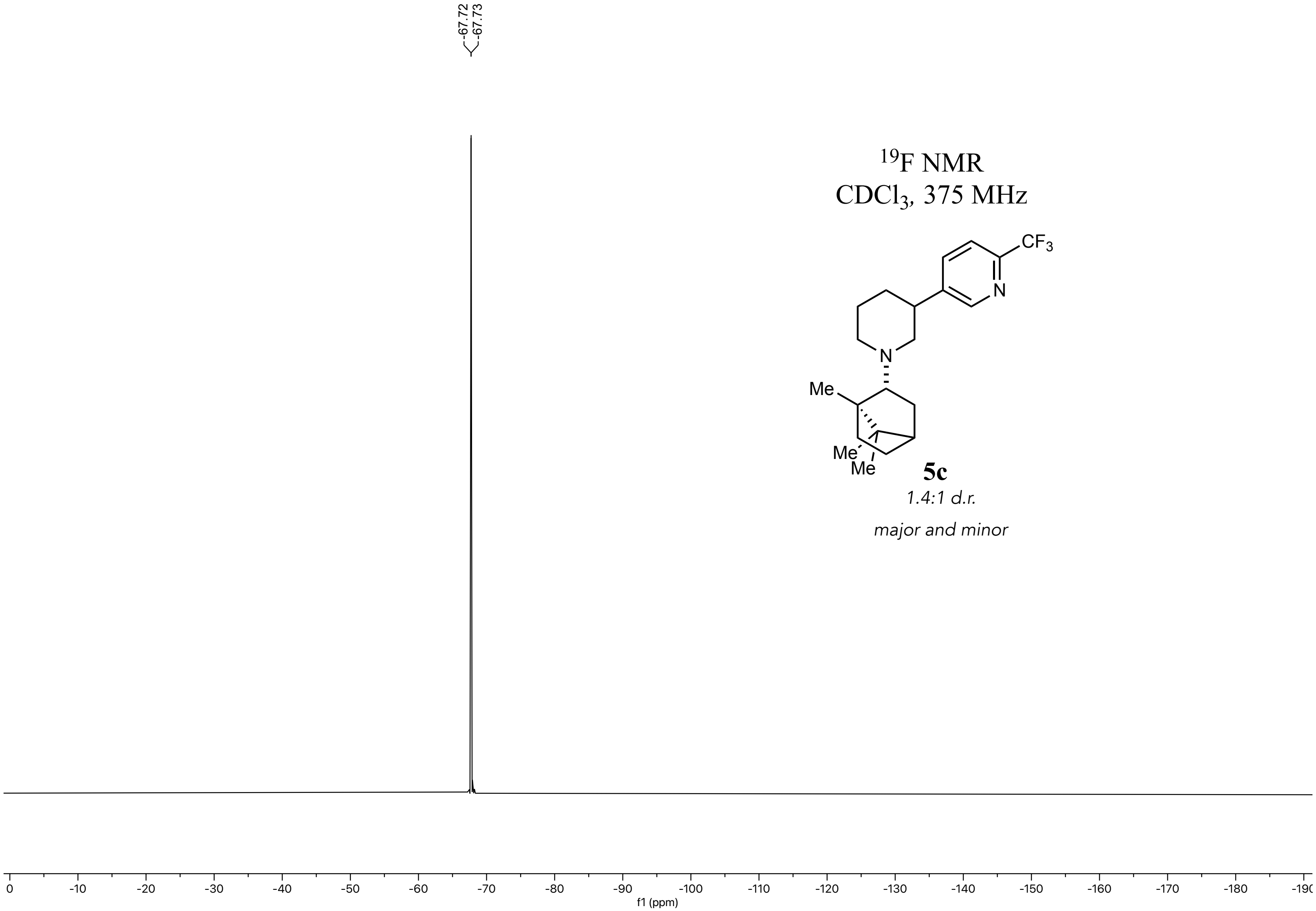

^{19}F NMR
$CDCl_3$, 375 MHz

5c

1.4:1 d.r.

major and minor
